# Development of Fragment-Based Inhibitors of the Bacterial Deacetylase LpxC with Low Nanomolar Activity

**DOI:** 10.1021/acs.jmedchem.4c01262

**Published:** 2024-09-20

**Authors:** Sebastian Mielniczuk, Katharina Hoff, Fady Baselious, Yunqi Li, Jörg Haupenthal, Andreas M. Kany, Maria Riedner, Holger Rohde, Katharina Rox, Anna K. H. Hirsch, Isabelle Krimm, Wolfgang Sippl, Ralph Holl

**Affiliations:** † Institute of Organic Chemistry, Universität Hamburg, Martin-Luther-King-Platz 6, 20146 Hamburg, Germany; ‡ German Center for Infection Research (DZIF), Partner Site Hamburg-Lübeck-Borstel-Riems, 20146 Hamburg, Germany; § Institute of Pharmacy, Martin-Luther-University of Halle-Wittenberg, Kurt-Mothes-Straße 3, 06120 Halle (Saale), Germany; ∥ Team “Small Molecules for Biological Targets”, Institut Convergence Plascan, Centre de Recherche en Cancérologie de Lyon, INSERM U1052-CNRS UMR5286, Centre Léon Bérard, Université de Lyon, Université Claude Bernard Lyon1, 69008 Lyon, France; ⊥ Shanghai Key Laboratory of Regulatory Biology, The Institute of Biomedical Sciences & School of Life Sciences, East China Normal University, 200241 Shanghai, China; # Helmholtz Institute for Pharmaceutical Research Saarland (HIPS), Helmholtz Centre for Infection Research (HZI), Campus E8.1, 66123 Saarbrücken, Germany; ¶ Technology Platform Mass Spectrometry, Universität Hamburg, Mittelweg 177, 20148 Hamburg, Germany; ∇ Institute of Medical Microbiology, Virology and Hygiene, University Medical Center Hamburg-Eppendorf, Martinistr. 52, 20246 Hamburg, Germany; ○ Department of Chemical Biology, Helmholtz Centre for Infection Research (HZI), Inhoffenstr. 7, 38124 Braunschweig, Germany; ⧫ German Center for Infection Research (DZIF), Partner Site Hannover-Braunschweig, 38124 Braunschweig, Germany; †† Helmholtz International Lab for Anti-infectives, Campus E8.1, 66123 Saarbrücken, Germany; ‡‡ Department of Pharmacy, Saarland University, Campus E8.1, 66123 Saarbrücken, Germany

## Abstract

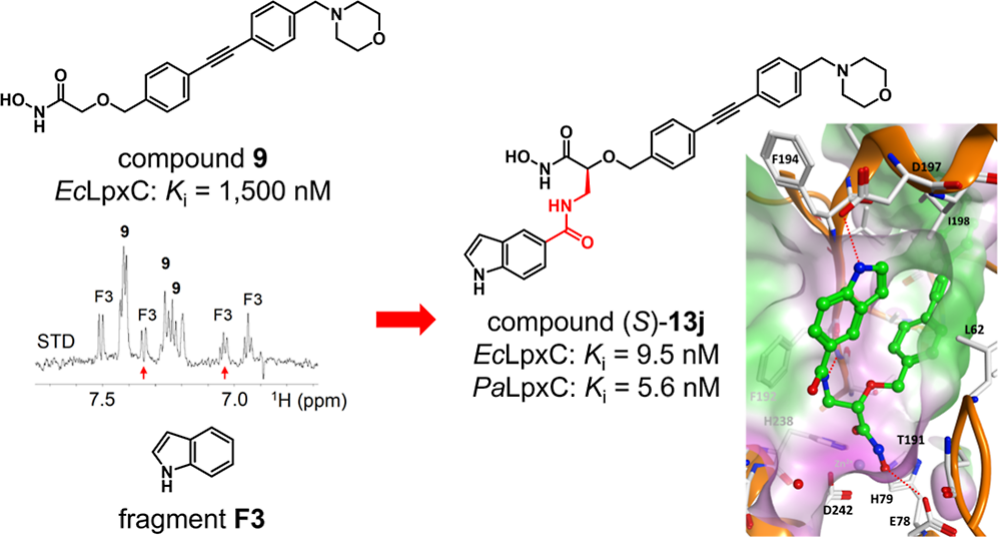

In a fragment-based approach using NMR spectroscopy, benzyloxyacetohydroxamic acid-derived inhibitors of the bacterial deacetylase LpxC bearing a substituent to target the uridine diphosphate-binding site of the enzyme were developed. By appending privileged fragments via a suitable linker, potent LpxC inhibitors with promising antibacterial activities could be obtained, like the one-digit nanomolar LpxC inhibitor (*S*)-**13j** [*K*_*i*_ (*Ec*LpxC C63A) = 9.5 nM; *K*_*i*_ (*Pa*LpxC): 5.6 nM]. To rationalize the observed structure–activity relationships, molecular docking and molecular dynamics studies were performed. Initial in vitro absorption–distribution–metabolism–excretion–toxicity (ADMET) studies of the most potent compounds have paved the way for multiparameter optimization of our newly developed isoserine-based amides.

## Introduction

Due to the emergence of pathogenic bacteria being resistant to most or all of the currently available antibiotics and the low success rate of drug development in this field, the need for new molecular frameworks is particularly crucial for antibiotics.^1−6^ Following the golden age of antibiotic research (1940–1960), the development of new antibiotic scaffolds for Gram-negative pathogens dramatically decreased.^1,7^ The target-based high-throughput screening campaigns carried out against a number of bacterial enzymes all failed to deliver candidates, and very low hit rates were obtained in comparison to what was typically observed with nonbacterial targets. Whole-cell high-throughput screening also yielded low hit rates.^1,8^ Alternative approaches and/or novel therapeutic targets are therefore necessary for the generation of novel antibiotics.

The inhibition of the biosynthesis of lipid A is a promising but hitherto clinically unexploited strategy for the development of antibiotics selectively combating Gram-negative bacteria.^9^ Lipid A acts as the hydrophobic membrane anchor of the lipopolysaccharides (LPS), which represent the main component of the outer monolayer of the outer membrane of Gram-negative bacteria, thus being essential for growth and viability of nearly all Gram-negative bacteria.^10^

**Figure 1:**
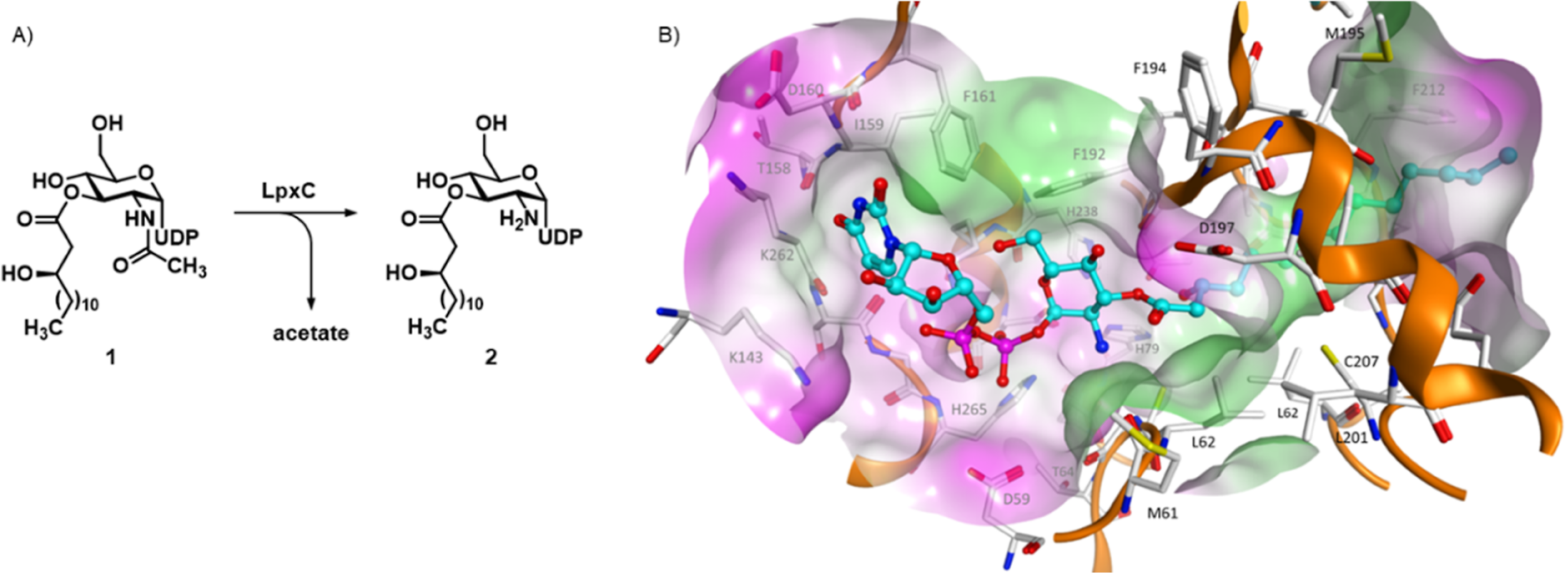
(A) LpxC-catalyzed deacetylation of UDP-3-*O*-[(*R*)-3-hydroxymyristoyl]-*N*-acetylglucosamine (**1**). (B) Molecular surface (colored according to the hydrophobicity, polar regions are colored magenta, hydrophobic regions are colored green) of *E. coli* LpxC near the deacetylated natural product **2** (PDB ID: 4MDT).^13^ UDP-3-*O*-[(*R*)-3-hydroxymyristoyl]-glucosamine (**2**) is shown as a cyan colored ball and stick model.

In Gram-negative bacteria, the biosynthesis of Kdo_2_-lipid A comprises a conserved pathway including nine enzymes.^10^ Its second step, in *Escherichia coli* the irreversible deacetylation of uridine diphosphate (UDP)-3-*O*-[(*R*)-3-hydroxymyristoyl]-*N*-acetylglucosamine (**1**, Figure 1A), is considered as the committed step of lipid A biosynthesis.^11^ This step is catalyzed by the Zn^2+^-dependent deacetylase LpxC. The enzyme is present in virtually all Gram-negative bacteria, is highly conserved among them, and possesses no mammalian counterpart, which makes LpxC an excellent target for the development of novel antibiotics.^12^

Structural studies revealed that LpxC displays a “β–α–α–β sandwich” fold, being formed by two domains with similar topologies.^14^ The catalytic Zn^2+^-ion is complexed by one aspartate and two histidine residues at the bottom of a conical active-site cleft, which is located at one side of the sandwich at the interface of the two domains (Figure 1B).^15^ A hydrophobic tunnel leads out of the active-site pocket, which binds the 3-*O*-[(*R*)-3-hydroxyacyl] substituent of the enzyme’s natural substrate **1** during catalysis.^16^

**Figure 2:**
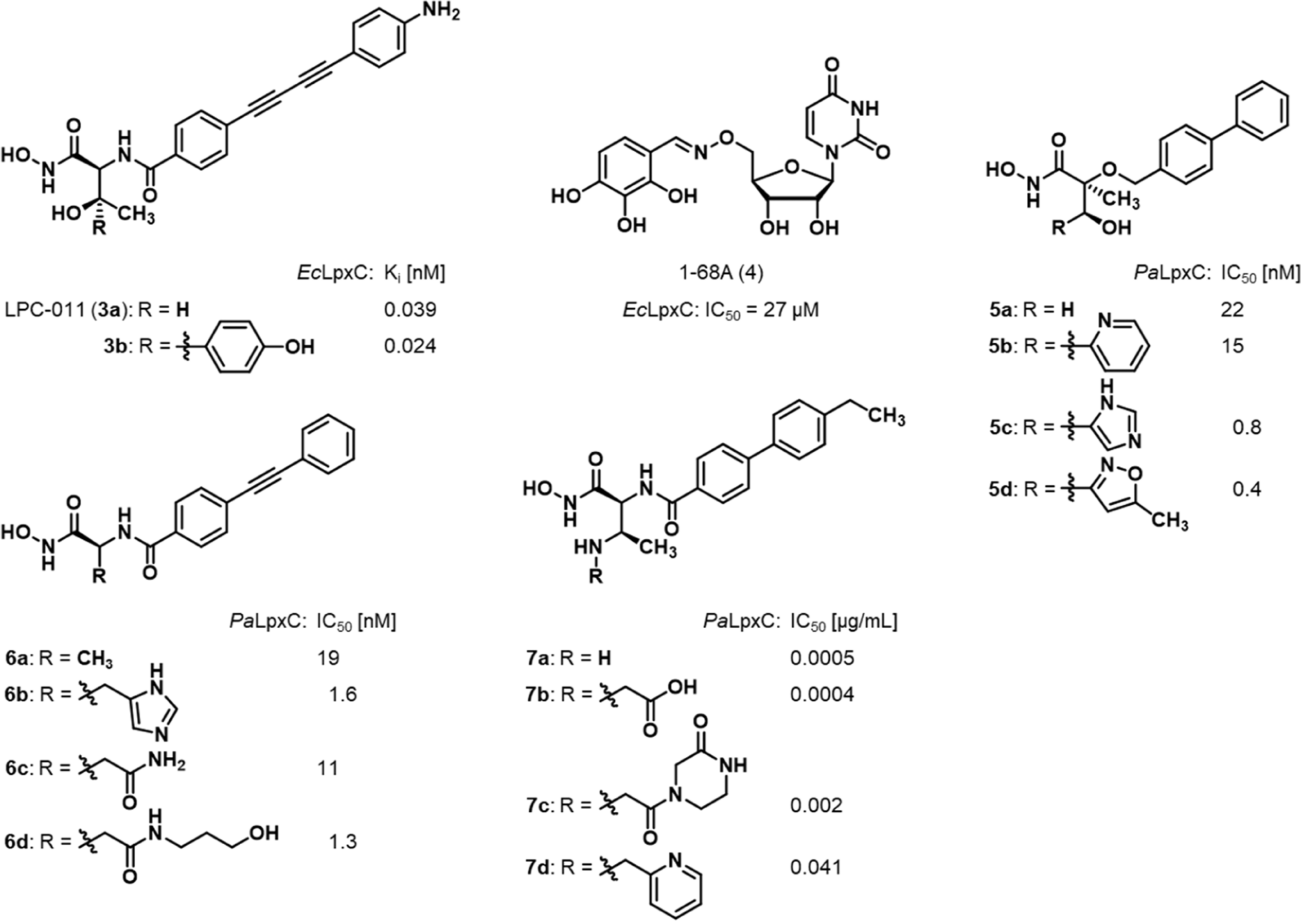
Structures of the described LpxC inhibitors targeting the enzyme’s UDP-binding site. Reported inhibitory activities toward *E. coli* LpxC (*Ec*LpxC) and *P. aeruginosa* LpxC (*Pa*LpxC) are given.^17−21^

Hitherto, various structural classes of small-molecule LpxC inhibitors have been described.^22−27^ Like the *N*-aroyl-l-threonine derivatives CHIR-090 (Figure S1) and LPC-011 (**3a**, Figure 2),^16,28^ most of these inhibitors exhibit a Zn^2+^-chelating hydroxamate moiety, which is linked to a lipophilic side chain addressing the enzyme’s hydrophobic tunnel. Although extensive structure–activity relationship (SAR) studies around the lipophilic side chain as well as the linker region have been performed, only a few LpxC inhibitors addressing the binding site of the UDP moiety of the enzyme’s natural substrate **1** have been reported so far.^22,23^ The uridine-based compound **1–68A** (**4**, Figure 2), which weakly inhibits LpxC, is a rare example of an inhibitor targeting the UDP-binding site of LpxC.^17^ Additionally, some attempts were undertaken to expand known inhibitors into the UDP-binding site. For instance, the attachment of a hydroxyphenyl group to the β-carbon atom of the threonyl moiety of LPC-011 (**3a**) led to a 1.6-fold improvement in inhibitory activity toward LpxC.^18^ In case of ether **5a** and amide **6a**, the attachment of suitable substituents also led to an increase in binding affinity over the respective parent compound.^19,20^ Finally, analogues of biphenyl derivative **7a** bearing substituents on the β-amino group retained potent inhibitory activity against LpxC, although they exhibited weaker antimicrobial activity.^21^ Altogether, these examples clearly show the feasibility of such attempts. However, although the fragment-based discovery of LpxC inhibitors containing a nonhydroxamate Zn^2+^-chelating motif has been reported recently,^29^ no systematic fragment-based studies aiming to find suitable substituents addressing the UDP-binding site have been described in the literature so far.

**Figure 3:**
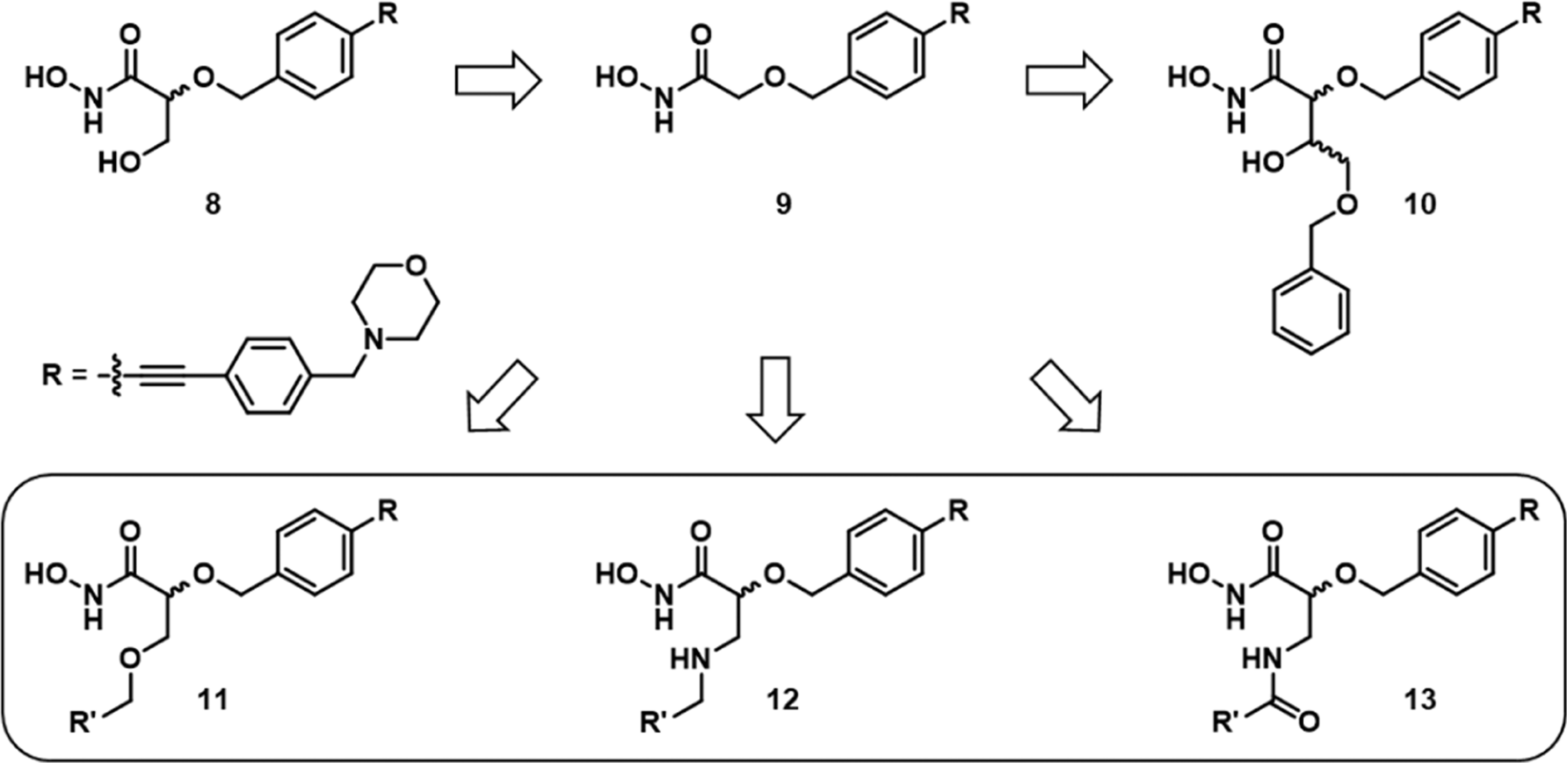
Structures of the described benzyloxyacetohydroxamic acid-based LpxC inhibitors and the envisaged linked ligands (encircled).^30−32^

**Figure 4:**
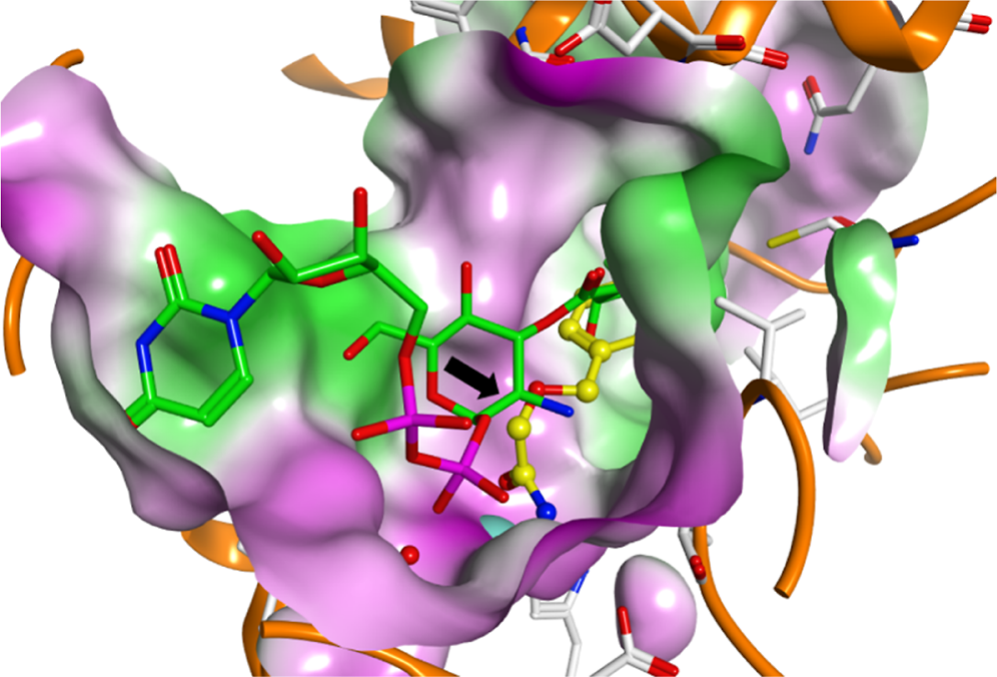
Docking pose of inhibitor **9** (colored yellow) at the LpxC UDP-binding pocket superimposed with the reaction product UDP-3-*O*-[(*R*)-3-hydroxymyristoyl]-glucosamine (**2**, colored green) crystallized with LpxC (PDB ID: 4MDT).^13^ The molecular surface is displayed and colored according to the polarity. Polar regions are colored magenta, and hydrophobic regions are colored green. The zinc ion is shown as a cyan sphere. The black arrow marks the site where the inhibitors of the current study were substituted to target the UDP-binding pocket.

Previously, we reported on the design, synthesis, and biological evaluation of a series of benzyloxyacetohydroxamic acid derivatives like glyceric acid derivatives (*S*)-**8** and (*R*)-**8** as well as the α-position-unsubstituted benzyloxyacetic acid derivative **9** (Figure 3, Table 1).^30,31^ While glyceric acid derivatives (*S*)-**8** and (*R*)-**8** were found to exhibit promising LpxC inhibitory and antibacterial activities, removal of the hydroxymethyl group in α-position, leading to compound **9**, caused a decrease in inhibitory activity. As these compounds leave the binding pocket for the UDP moiety of the natural substrate of LpxC unoccupied (Figure 4), substituents addressing the UDP-binding pocket should be introduced in the α-position of the hydroxamate moiety in order to gain further favorable interactions with the enzyme, thus improving the affinity of the compounds toward LpxC. To demonstrate the feasibility of this approach, we previously synthesized aldotetronic acid derivatives **10**.^32^ Even though the LpxC inhibitory activity of the most potent stereoisomer (2*S*,3*S*)-**10** only slightly exceeded the one of glyceric acid derivative (*S*)-**8** (Table 1), the observation that the elongation of the substituent in α-position did not diminish LpxC inhibitory activity shows that it is possible to grow our inhibitors into the UDP-binding site of LpxC.

**Table 1:**
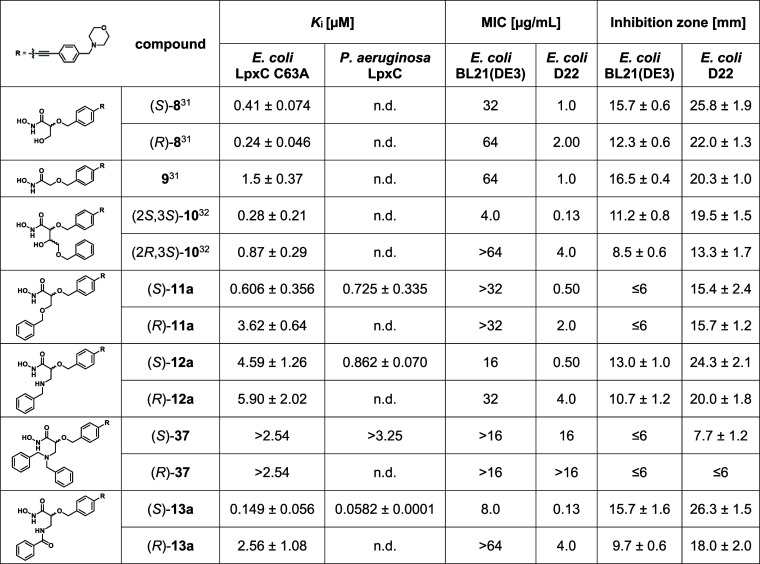
Antibacterial and LpxC Inhibitory Activities of Reported and Newly Synthesized Hydroxamic Acids^a^

a n.d.: not determined.

Having shown that the introduction of a substituent addressing the UDP-binding pocket of LpxC in the α-position of the hydroxamate moiety is feasible, in this paper we report how we pursued this attractive strategy to further optimize our benzyloxyacetohydroxamic acid derivatives. In order to generate LpxC inhibitors that address the UDP-binding pocket, various linkers were investigated. Thus, besides the tetrose-derived ethers **10**, glyceric acid-derived ethers **11** as well as isoserine-based amines **12** and amides **13** were synthesized. In parallel, to identify chemical structures capable of binding into the available UDP pocket, we have performed a fragment screen against LpxC in the presence of compound **9**. We screened a library of 650 fragments to identify small substructures that bind the protein target through a minimal recognition motif. Fragments, small compounds (MW < 250 Da) with low complexity, typically bind proteins with weak affinities (usually *K*_D_ > 100 μM), nevertheless exhibiting high ligand efficiency (binding energy per heavy atom) due to high-quality interactions.^33,34^ Saturation transfer difference (STD)-NMR, WaterLogsy, and subsequent NMR-interligand nuclear Overhauser effect (ILOE) experiments^35−37^ were performed to identify fragments that bind into the enzyme’s UDP-binding pocket near the methylene group in the α-position of the hydroxamate moiety of compound **9**. After the identification of an optimal linker, substituents inspired by the identified fragments were connected with the benzyloxyacetohydroxamic acids, and the biological activities of the obtained compounds were investigated. Additionally, the effect of the stereochemistry of the newly synthesized compounds on their biological activities was studied.

## Results and Discussion

### Synthesis of Compounds with Ether, Amine, and Amide Linkers

In order to find an optimal linker, a phenyl ring as an exemplary substituent should be connected to the scaffold of the benzyloxyacetohydroxamic acid derivatives via structural elements of different structures.

**Scheme 1:**
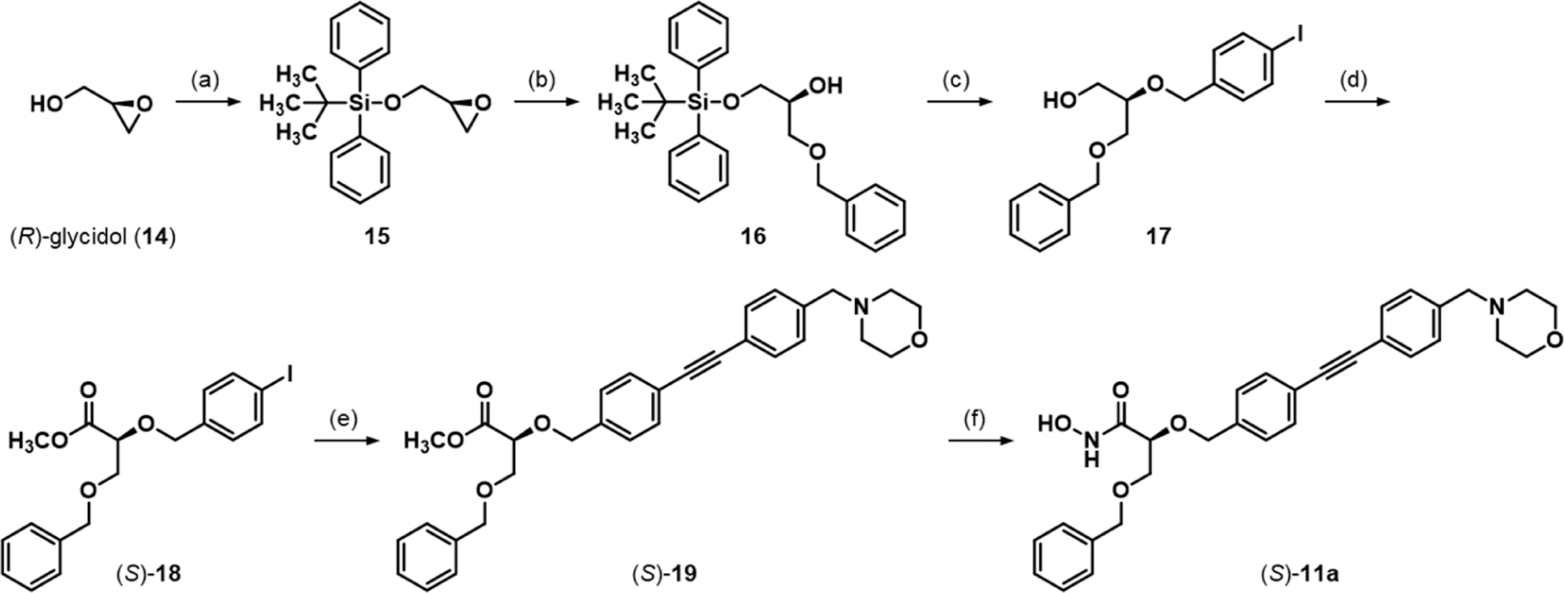
Synthesis of Benzyl Ether (*S*)-**11a** Reagents and Conditions: (a) TBDPSCl, imidazole, CH_2_Cl_2_, 0 °C → rt, 86%; (b) BnOH, Er(OTf)_3_, rt, 73%; (c) (1) NaH, 4-iodobenzyl bromide, THF, 0 °C → rt, (2) TBAF, THF, rt, 71%; (d) (1) CrO_3_/H_5_IO_6_, ACN, H_2_O, 0 °C → rt, (2) H_2_SO_4_, MeOH, Δ, 48%; (e) 4-(4-ethynylbenzyl)morpholine, [PdCl_2_(PPh_3_)_2_], CuI, diisopropylamine, THF, rt, 88%; (f) aq. NH_2_OH, THF/*i*-PrOH (1:1), 0 °C → rt, 67%.

Thus, to vary the length and structure of the linker region compared to the previously investigated aldotetronic acid-based LpxC inhibitors, benzyl ethers (*S*)-**11a** and (*R*)-**11a** were synthesized. Ether (*S*)-**11a** was obtained from (*R*)-glycidol (**14**, Scheme 1). After the protection of the primary alcohol of **14** with *tert*-butyldiphenylsilyl chloride, the oxirane ring of the resulting silyl ether **15** was opened with benzyl alcohol in the presence of erbium(III) triflate, yielding secondary alcohol **16**.^38^ Then, a Williamson ether synthesis with 4-iodobenzyl bromide was performed, and the silyl protective group was cleaved with tetrabutylammonium fluoride to give primary alcohol **17**. Subsequently, the alcohol was oxidized using an oxidant solution, which was composed of periodic acid and catalytic amounts of chromium(VI) oxide in wet acetonitrile, and the resulting carboxylic acid was esterified with methanol in the presence of sulfuric acid. The thereby obtained ester (*S*)-**18** was subjected to a Sonogashira coupling with 4-(4-ethynylbenzyl)morpholine to yield diphenylacetylene derivative (*S*)-**19**. Finally, ester (*S*)-**19** was converted into the desired hydroxamic acid (*S*)-**11a** by performing an aminolysis with hydroxylamine.^39,40^

**Scheme 2:**
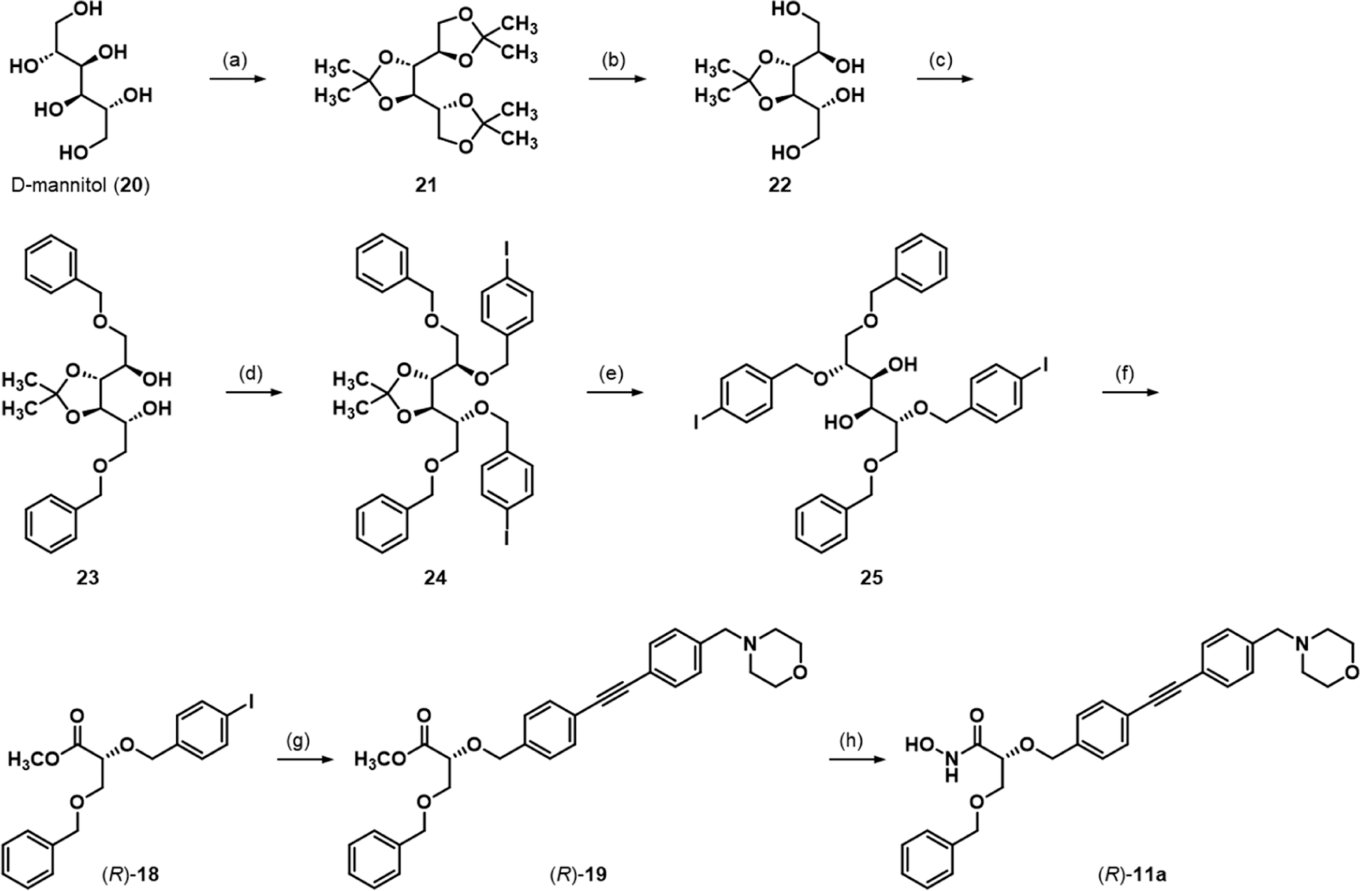
Synthesis of Benzyl Ether (*R*)-**11a** Reagents and Conditions: (a) acetone, H_2_SO_4_, rt, 32%; (b) 70% HOAc, 40 °C, 69%; (c) (1) Bu_2_SnO, toluene, Δ, (2) benzyl bromide, Bu_4_NI, toluene, 70 °C, 92%; (d) NaH, 4-iodobenzyl bromide, THF, rt, 85%; (e) 80% TFA, 0 °C, 97%; (f) (1) NaIO_4_, MeOH, rt, (2) Br_2_, NaHCO_3_, MeOH/H_2_O (9:1), rt, 69%; (g) 4-(4-ethynylbenzyl)morpholine, [PdCl_2_(PPh_3_)_2_], CuI, diisopropylamine, THF, Δ, then rt, 64%; (h) aq. NH_2_OH, THF/*i*-PrOH (1:1), 0 °C → rt, 65%.

The enantiomeric ether (*R*)-**11a** was accessed in a chiral pool synthesis starting from d-mannitol (**20**, Scheme 2). After the conversion of d-mannitol (**20**) into trisacetonide **21** with acetone in the presence of sulfuric acid, **21** was stirred at 40 °C in 70% aqueous acetic acid to yield 3,4-*O*-isopropylidene-d-mannitol (**22**).^41,42^ Subsequently, the primary alcohols of tetrol **22** were selectively alkylated with benzyl bromide to give diether **23**, which was subjected to a Williamson ether synthesis with 4-iodobenzyl bromide, yielding tetraether **24**. Then, the remaining acetonide moiety was hydrolyzed, and the C–C bond between C-3 and C-4 of the resulting glycol **25** was cleaved with sodium periodate to yield two identical (*R*)-configured aldehydes, which were directly transformed into glyceric acid ester (*R*)-**18** via an oxidation with bromine in a 9:1 mixture of methanol and water in the presence of sodium bicarbonate.^43^ Like its enantiomer, hydroxamic acid (*R*)-**11a** was finally obtained from aryl iodide (*R*)-**18** via a Sonogashira coupling and a subsequent aminolysis with hydroxylamine.

**Scheme 3:**
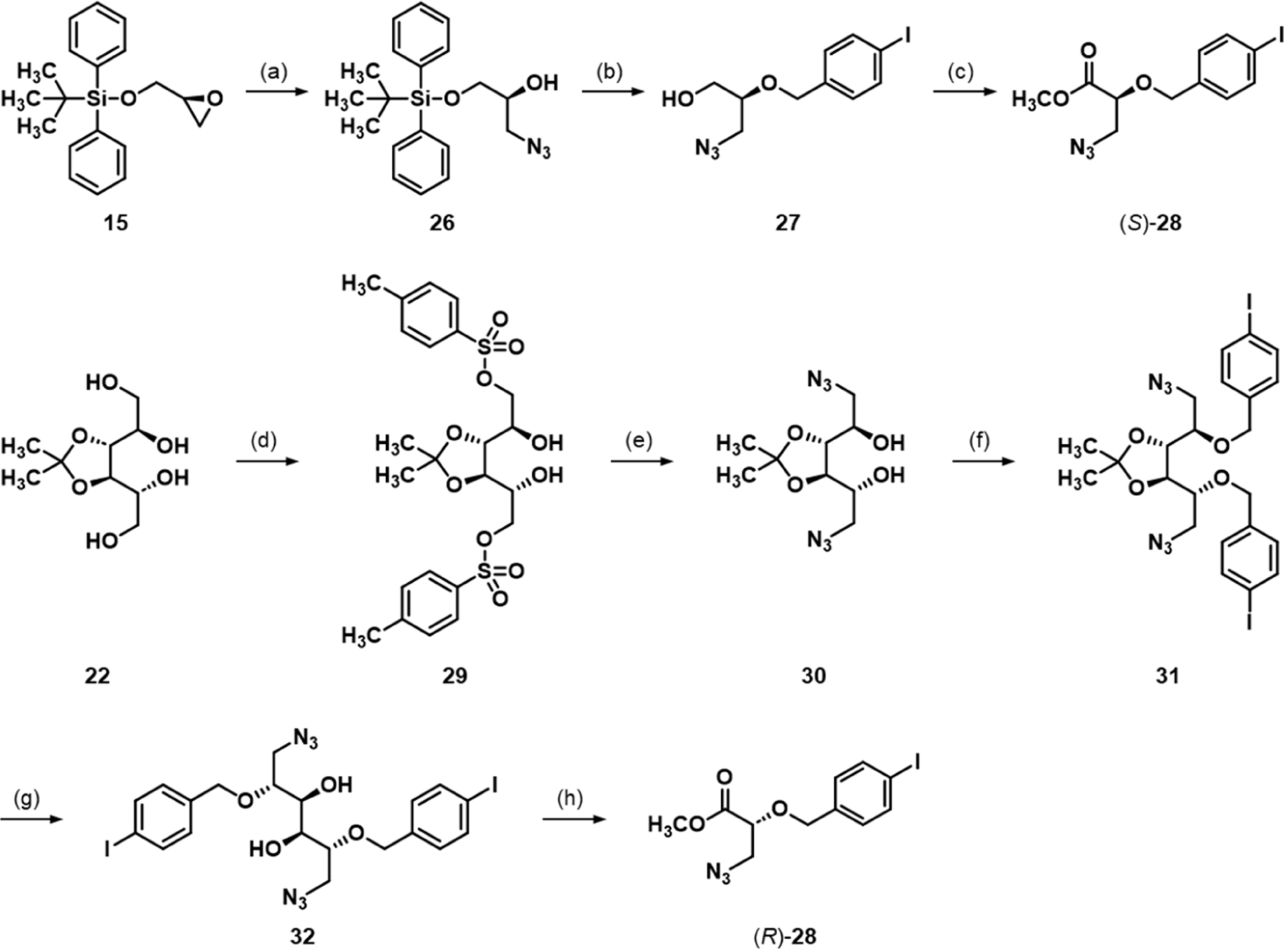
Synthesis of Azides (*S*)-**28** and (*R*)-**28** Reagents and Conditions: (a) NaN_3_, NH_4_Cl, MeOH, H_2_O, 65 °C, 70%; (b) (1) NaH, 4-iodobenzyl bromide, THF, 0 °C → rt, (2) TBAF, THF, rt, 78%; (c) (1) CrO_3_/H_5_IO_6_, ACN, H_2_O, 0 °C → rt, (2) H_2_SO_4_, MeOH, Δ, 65%; (d) (1) Bu_2_SnO, toluene, Δ, (2) *p*-TsCl, CHCl_3_, 0 °C → rt, 94%; (e) NaN_3_, DMSO, 80 °C, 89%; (f) NaH, 4-iodobenzyl bromide, THF, rt, 82%; (g) 80% HOAc, 0 °C, 90%; (h) (1) NaIO_4_, MeOH, rt, (2) Br_2_, NaHCO_3_, MeOH/H_2_O (9:1), rt, 62%.

The envisaged isoserine-derived amines and amides were synthesized from azides (*S*)-**28** and (*R*)-**28**. To obtain azide (*S*)-**28**, epoxide **15** was subjected to a ring-opening reaction with sodium azide in the presence of ammonium chloride to yield secondary alcohol **26** as the preferred regioisomer (Scheme 3).^44^ A Williamson ether synthesis with 4-iodobenzyl bromide and the subsequent cleavage of the silyl group afforded primary alcohol **27**, which was oxidized and thereafter reacted with methanol in the presence of sulfuric acid to give the desired ester (*S*)-**28**.

The enantiomeric azide (*R*)-**28** could have been synthesized in the same way starting from commercially available (*S*)-glycidol. However, an alternative synthetic route starting from d-mannitol (**20**) was established. Thus, the primary alcohols of d-mannitol-derived tetrol **22** were selectively tosylated via the intermediate formation of the respective stannylidene acetals (Scheme 3). The thereby obtained bistosylate **29** was subjected to a nucleophilic substitution with sodium azide to give diazide **30**. Subsequently, the remaining hydroxy groups were alkylated with 4-iodobenzyl bromide yielding diether **31**, followed by an acetal cleavage to obtain glycol **32**. As for the synthesis of the (*R*)-configured benzyl ethers, diol **32** was subjected to a glycol cleavage with sodium periodate and the obtained identical (*R*)-configured aldehydes were oxidized to ester (*R*)-**28** with bromine in a mixture of methanol and water (9:1) buffered with sodium bicarbonate.

While the chiral pool synthesis starting from d-mannitol required more steps and gave (*R*)-**28** in a considerably lower overall yield compared to the synthesis of (*S*)-**28** starting from (*R*)-glycidol [8.5% for (*R*)-**28** vs 32% for (*S*)-**28**], this synthetic route yielded (*R*)-**28** in an enantiomerically pure form [*ee* of (*R*)-**28**: 100% vs *ee* of (*S*)-**28**: 99.2%].

**Scheme 4:**
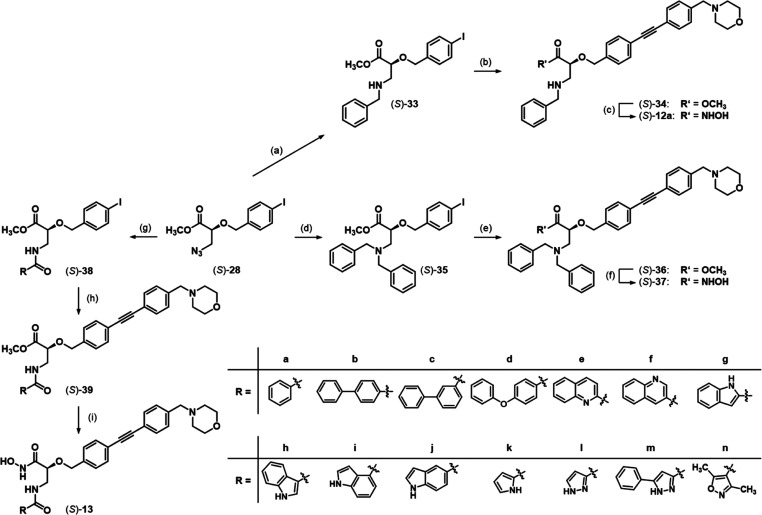
Synthesis of Hydroxamic Acids (*S*)-**12a**, (*S*)-**37**, and (*S*)-**13a-n** Reagents and Conditions: (a) (1) P(OEt)_3_, toluene, 0 °C → rt, (2) benzaldehyde, 0 °C → rt, (3) NaBH_4_, MeOH, 0 °C → rt, 67%; (b) 4-(4-ethynylbenzyl)morpholine, [PdCl_2_(PPh_3_)_2_], CuI, diisopropylamine, THF, rt, 68%; (c) aq. NH_2_OH, THF/*i*-PrOH (1:1), 0 °C → rt, 70%; (d) (1) PS–PPh_3_, THF, H_2_O, 40 °C, (2) benzaldehyde, NaBH(OAc)_3_, dichloroethane, 0 °C → rt, (3) H_2_SO_4_, MeOH, 80 °C, 50%; (e) 4-(4-ethynylbenzyl)morpholine, [PdCl_2_(PPh_3_)_2_], CuI, diisopropylamine, THF, rt, 87%; (f) aq. NH_2_OH, THF/*i*-PrOH (1:1), 0 °C → rt, 47%; (g) P(CH_3_)_3_, R–CO_2_H, 2,2′-dithiodipyridine, toluene, 0 °C → rt, (*S*)-**38a** 83%, (*S*)-**38b** 74%, (*S*)-**38c** 76%, (*S*)-**38d** 77%, (*S*)-**38e** 92%, (*S*)-**38f** 82%, (*S*)-**38g** 76%, (*S*)-**38h** 65%, (*S*)-**38i** 64%, (*S*)-**38j** 34%, (*S*)-**38k** 33%, (*S*)-**38l** 50%, (*S*)-**38m** 66%, (*S*)-**38n** 83%; (h) 4-(4-ethynylbenzyl)morpholine, [PdCl_2_(PPh_3_)_2_], CuI, diisopropylamine, THF, rt, (*S*)-**39a** 94%, (*S*)-**39b** 50%, (*S*)-**39c** 88%, (*S*)-**39d** 90%, (*S*)-**39e** 99%, (*S*)-**39f** 78%, (*S*)-**39g** 87%, (*S*)-**39h** 98%, (*S*)-**39i** 64%, (*S*)-**39j** 83%, (*S*)-**39k** 78%, (*S*)-**39l** 78%, (*S*)-**39m** 88%, (*S*)-**39n** 87%; (i) aq. NH_2_OH, THF/*i*-PrOH (1:1), 0 °C → rt, (*S*)-**13a** 41%, (*S*)-**13b** 82%, (*S*)-**13c** 70%, (*S*)-**13d** 77%, (*S*)-**13e** 68%, (*S*)-**13f** 79%, (*S*)-**13g** 82%, (*S*)-**13h** 67%, (*S*)-**13i** 63%, (*S*)-**13j** 86%, (*S*)-**13k** 75%, (*S*)-**13l** 48%, (*S*)-**13m** 76%, (*S*)-**13n** 84%.

Starting from azide (*S*)-**28**, secondary amine (*S*)-**33** could be obtained via a Staudinger/aza-Wittig reaction, followed by the reduction of the resultant imine intermediate (Scheme 4). Thus, azide (*S*)-**28** was reacted with triethyl phosphite, and the resulting triethoxyiminophosphorane intermediate was converted into an imine with benzaldehyde, which was then reduced with sodium borohydride.^45^ In contrast, a Staudinger reduction of azide (*S*)-**28** with polymer-bound triphenylphosphine in the presence of water and subsequent reductive alkylation of the resultant primary amine with an excess of benzaldehyde and sodium triacetoxyborohydride yielded tertiary amine (*S*)-**35**.^46^ Finally, benzamide (*S*)-**38a** could be accessed via a Staudinger–Vilarrasa reaction by reacting azide (*S*)-**28** with benzoic acid in the presence of trimethylphosphane and 2,2′-dithiodipyridine.^47^ Subsequently, the three isoserine-derived aryl iodides (*S*)-**33**, (*S*)-**35**, and (*S*)-**38a** were subjected to Sonogashira couplings with 4-(4-ethynylbenzyl)morpholine and final aminolyses with hydroxylamine to yield hydroxamic acids (*S*)-**12a**, (*S*)-**37**, and (*S*)-**13a**.

The respective enantiomers (*R*)-**12a**, (*R*)-**37**, and (*R*)-**13a** were obtained in principally the same way starting from azide (*R*)-**28**.

### Identification of the Most Suitable Linker

In order to determine the inhibitory activities of the compounds of interest toward *E. coli* LpxC, a fluorescence-based enzyme assay was deployed, in which the formed deacetylated natural product UDP-3-*O*-[(*R*)-3-hydroxymyristoyl]glucosamine (**2**) is transformed into a fluorescent isoindole with *o*-phthaldialdehyde and 2-mercaptoethanol.^48,49^ In the assay, *E. coli* LpxC C63A was used, as this mutant is significantly less susceptible to being inhibited by high concentrations of Zn^2+^ compared to the wild-type enzyme.^50,51^ The *K*_M_ of *E. coli* LpxC C63A was determined experimentally using a mass spectrometry-based LpxC assay (Figure S8, Supporting Information, vide infra). The *K*_M_ was found to be 3.6 μM, thus being in the same range as the reported *K*_M_ of wild-type *E. coli* LpxC (4.0 μM).^52^

In order to identify the optimal linker to connect a phenyl ring with the benzyloxyacetic acid scaffold, the LpxC inhibitory activities of the tetrose-derived ethers (2*S*,3*S*)-**10** and (2*R*,3*S*)-**10**, glyceric acid-derived ethers (*S*)-**11a** and (*R*)-**11a**, isoserine-based secondary amines (*S*)-**12a** and (*R*)-**12a**, tertiary amines (*S*)-**37** and (*R*)-**37**, and amides (*S*)-**13a** and (*R*)-**13a** were compared (Table 1). As reported previously, the inhibitory activity of tetrose-derived ether (2*S*,3*S*)-**10** slightly exceeded the one of glyceric acid derivative (*S*)-**8**.^32^ And while the configuration in α-position had only a minor effect on the inhibitory activities of glyceric acid-derived hydroxamic acids (*S*)-**8** and (*R*)-**8**,^31^ with a slight superiority of the (*R*)-configured enantiomer, the (2*S*)-configured aldotetronic acid derivative (2*S*,3*S*)-**10** exhibited an about 3-fold higher inhibitory activity compared to its (2*R*)-configured diastereomer (2*R*,3*S*)-**10**.^32^ The newly synthesized glyceric acid-derived ether (*S*)-**11a** was found to be a slightly less potent LpxC inhibitor than glyceric acid derivative (*S*)-**8** and tetrose-derived ether (2*S*,3*S*)-**10**. In case of ethers (*S*)-**11a** and (*R*)-**11a**, the inhibitory activity of the (*S*)-configured stereoisomer exceeds the one of its (*R*)-configured enantiomer by a factor of 6. While the isoserine-based amines (*S*)-**12a**, (*R*)-**12a**, (*S*)-**37**, and (*R*)-**37** showed considerably reduced LpxC inhibitory activities, the isoserine-based amide (*S*)-**13a** exhibited a promising *K*_*i*_ value of 0.15 μM, thus exceeding the inhibitory activities of glyceric acid derivative (*S*)-**8** and tetrose-derived ether (2*S*,3*S*)-**10**. Additionally, in case of benzamides (*S*)-**13a** and (*R*)-**13a**, the eudysmic ratio was the highest in the examined series of compounds, with (*S*)-**13a** being a 17-fold more potent LpxC inhibitor than its enantiomer (*R*)-**13a**.

Therefore, the amide linker turned out to be the most favorable with respect to inhibitory activity toward LpxC.

Besides measuring the inhibitory activities toward *E. coli* LpxC C63A, the antibacterial activities of the synthesized hydroxamic acids were evaluated by determining their minimal inhibitory concentrations (MIC) in broth dilution tests as well as by performing disc diffusion assays (Table 1). Thus, the compounds were tested against *E. coli* BL21(DE3) (*lpxC*^+^) and the defective *E. coli* D22 (*lpxC101*) strain,^53^ exhibiting reduced LpxC activity.

A comparison of the benzyloxyacetohydroxamic acids bearing a phenyl substituent connected via different linker regions revealed that the tetrose-derived ether (2*S*,3*S*)-**10** as well as the isoserine-based amide (*S*)-**13a** exhibit the lowest MIC values against the two investigated *E. coli* strains, which agrees with the low *K*_*i*_ values of the two compounds. Surprisingly, although showing only negligible inhibitory activity against *E. coli* LpxC C63A, notable antibacterial activity was found for secondary amine (*S*)-**12a**.

### Fragment Screening Using NMR

In order to find fragments specifically addressing the UDP-binding pocket, which can be linked to the LpxC inhibitors, a fragment screen was performed against LpxC bound to **9**. First, 650 fragments in cocktails of five were tested for binding to the LpxC–**9** complex using STD-NMR and WaterLogsy experiments. Then, fragment hits identified as potential binders were tested one by one against the LpxC–**9** complex by STD-NMR and WaterLogsy experiments performed under identical experimental conditions. A total of 97 fragment hits were identified as binders, leading to a hit rate of 15%.

**Figure 5:**
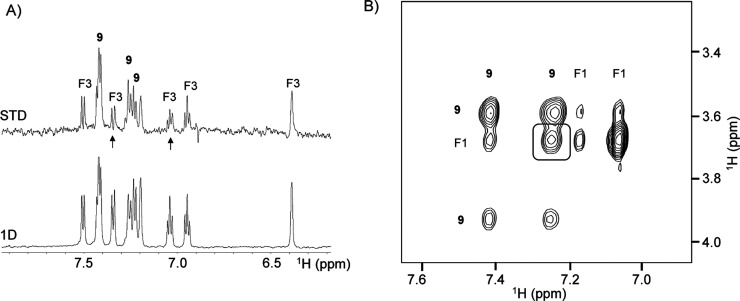
NMR experiments for the identification of fragments bound to the complex LpxC–**9**. (A) STD spectrum of fragment F3 in the presence of the complex LpxC–**9**. Arrows highlight two resonances of fragment F3 exhibiting the lowest STD factors, indicating that the corresponding protons are more solvent-exposed than the other protons. (B) Nuclear Overhauser effect spectroscopy (NOESY) spectrum showing transferred NOESY peaks observed for compounds **9** and F1 bound to LpxC. The largest interligand NOESY peak between **9** and F1 is shown in the square.

High hit rates are typically observed in fragment screening due to the low complexity and small size of the fragments as well as artifacts. Also, the highly hydrophobic nature of the fragments (mostly aromatic) gives them a high ability to bind to protein pockets. Such fragments can exhibit multiple binding modes when binding to proteins, which give rise to STD factors that are averaged and can therefore have similar values for each proton of the fragment.^54^ By contrast, fragments that bind proteins with a privileged binding mode should exhibit a STD-based epitope mapping,^54,55^ where the STD factor value for each proton reflects the proximity between the fragment proton to the protein protons. Here, due to the high rate of fragment hits, we decided to select fragments that displayed a STD-based epitope mapping. We acknowledge that this approach does not guarantee a unique binding mode for the fragments, and that some interesting fragments can be missed. We considered that this strategy would help in the selection of the most promising ligands. Finally, 19 fragment hits were selected as potentially interesting LpxC binders, which correspond to a hit rate of 2.9% (Table 2 and Figure 5A).

**Table 2:**
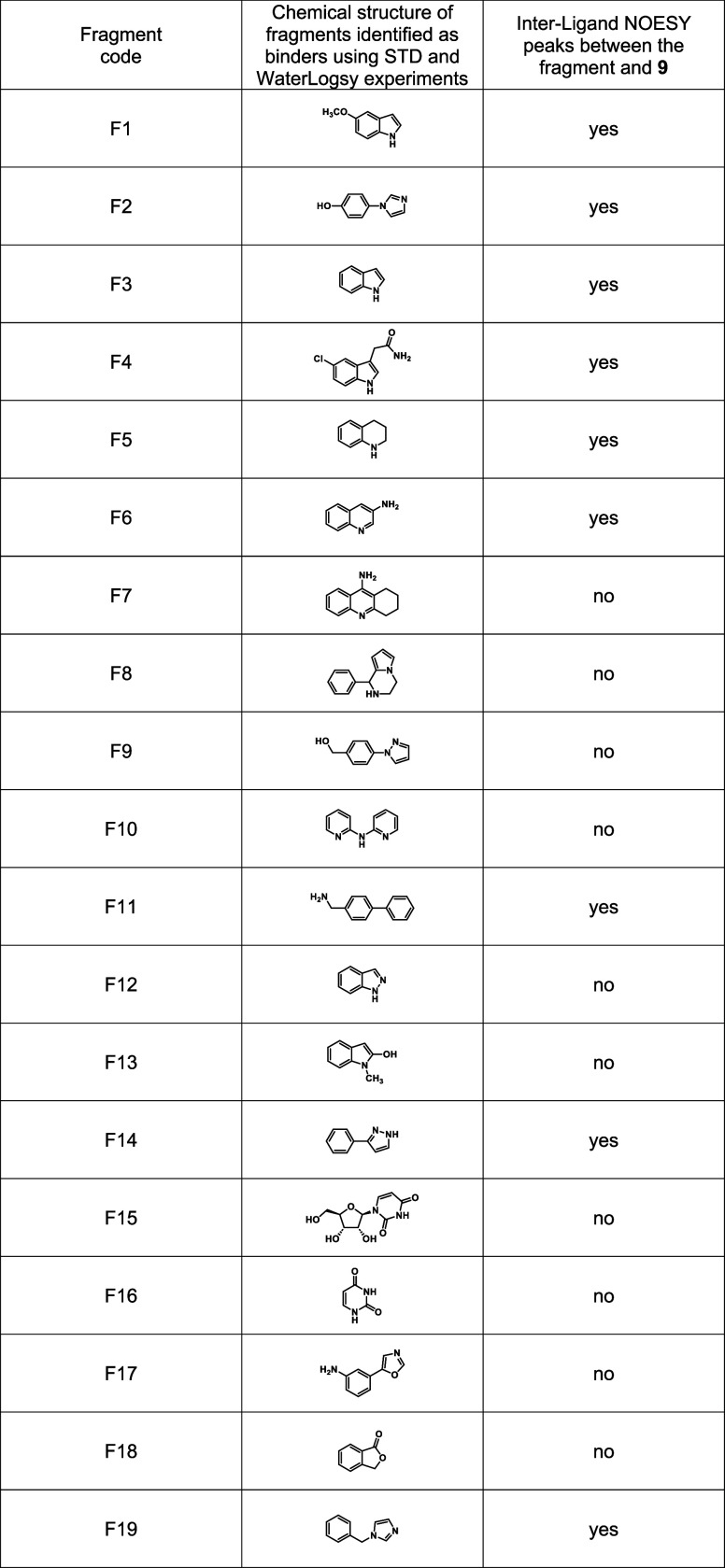
Fragment Hits Identified as Binders of the LpxC–**9** Complex

Then, NMR-ILOE experiments were performed with selected fragment hits in the presence of LpxC and the unsubstituted hydroxamic acid **9**. This allowed the identification of fragments that bind in the enzyme’s UDP-binding pocket near the methylene group in the α-position of the inhibitor’s hydroxamate moiety. Weak interligand NOESY peaks were observed between inhibitor **9** and nine fragments. By contrast, no interligand NOESY peaks were observed between compound **9** and the other ten fragments. The nine fragments exhibited weak interligand NOESY peaks between protons of the fragment and protons of the CH_2_ groups near the hydroxamate moiety as well as the aromatic resonances of **9** (see, for example, Figure 5B, showing the NOESY spectrum between **9** and F1). Unexpectedly, we also observed interligand NOESY between the fragment protons and protons of the morpholino moiety.

Using the data from the STD, WaterLogsy, and NOESY experiments, we identified nine fragments that were capable of binding LpxC in the UDP-binding pocket near the inhibitor’s hydroxamate moiety (Table 2). The interpretation of the NOESY was rather ambiguous, and no further structural information (such as the relative orientation of the fragment and compound **9**) was inferred from these experiments. Thus, from these chemical structures, we selected four substructures (biphenyl, indole, quinoline, and pyrazole) to be linked to the benzyloxyacetic acid scaffold of hydroxamic acid **9**.

### Synthesis and LpxC Inhibitory Activity of Linked Compounds

As the amide linker had turned out to be the most favorable with respect to inhibitory activity toward LpxC and the Staudinger–Vilarrasa reaction provided convenient access to the desired amides, the selected biphenyl-, indole-, quinoline-, and pyrazole-based substituents were linked via an amide linker to the scaffold of hydroxamic acid **9**. Additionally, to broaden the SAR, diphenyl ether-, pyrrole-, and isoxazole-based substituents were introduced. Thus, azide (*S*)-**28** was ligated with the respective carboxylic acids to finally obtain hydroxamic acids (*S*)-**13b**–**n** (Scheme 4). The enantiomeric amides (*R*)-**13b**–**h**,**j**,**m**,**n** were obtained in principally the same way starting from azide (*R*)-**28**.

The synthesized amide-based hydroxamic acids **13** were tested for inhibitory activity toward *E. coli* LpxC C63A. The results are reported in Table 3.

**Table 3:**
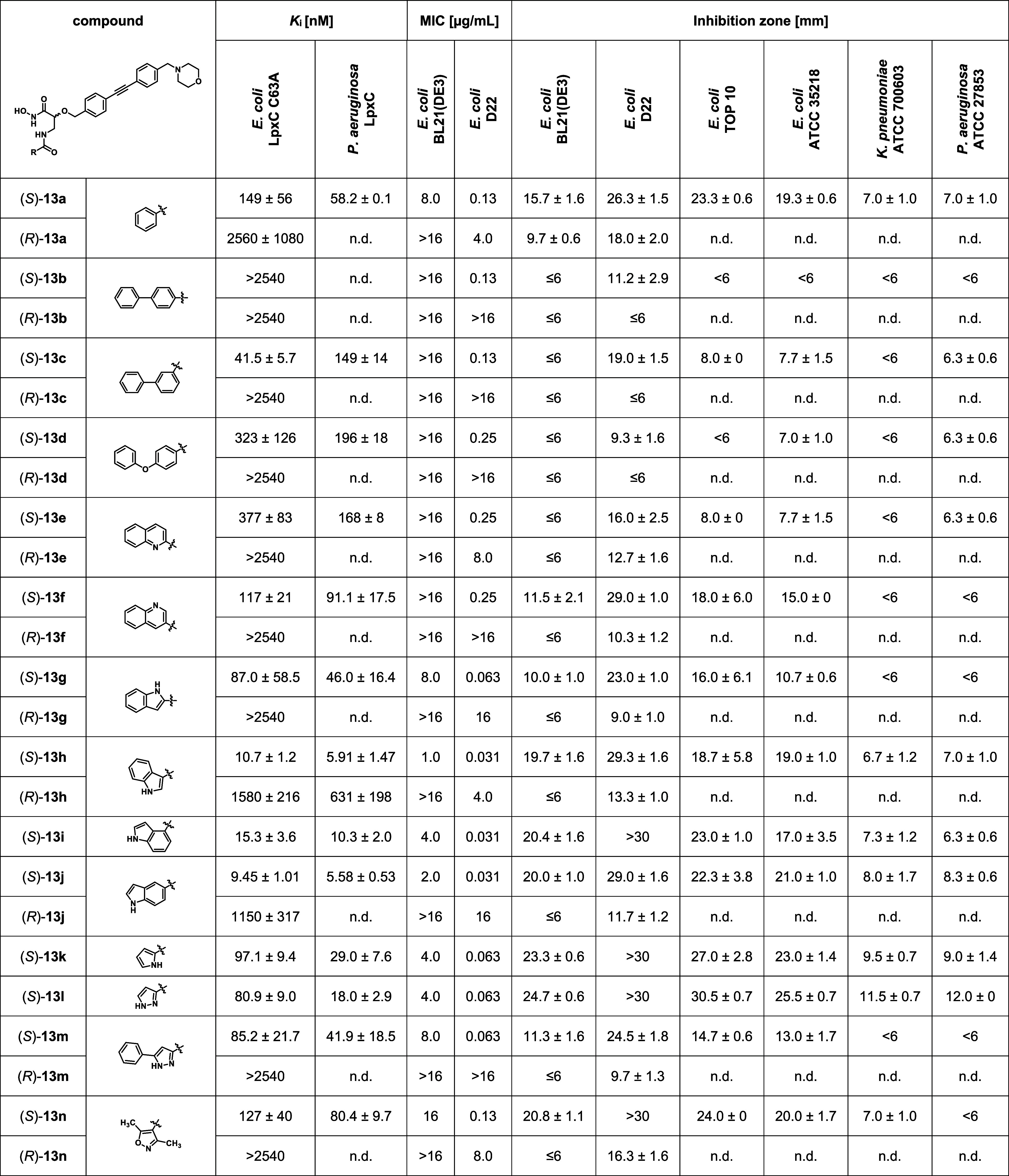
Antibacterial and LpxC Inhibitory Activities of Isoserine-Based Hydroxamic Acids^a^

a n.d.: not determined.

Generally, as observed for benzamides (*S*)-**13a** and (*R*)-**13a**, the (*S*)-configured compound was found to be the eutomer of all investigated pairs of enantiomeric amides. In case of the synthesized benzamide derivatives, the introduction of another phenyl ring in position 4 of the benzoyl moiety, leading to biphenyl derivative (*S*)-**13b**, was found to be detrimental to inhibitory activity. While the introduction of a 4-phenoxy substituent, leading to diphenyl ether (*S*)-**13d**, caused only a slight reduction of inhibitory activity compared to (*S*)-**13a**, the introduction of a 3-phenyl substituent led to a pronounced increase in inhibitory activity. Thus, the relatively bent biphenyl derivative (*S*)-**13c** was found to exhibit a *K*_*i*_ value of 42 nM. The investigated acylamides containing a five-membered heterocycle, namely pyrrole (*S*)-**13k**, pyrazole derivatives (*S*)-**13l** and (*S*)-**13m**, as well as isoxazole (*S*)-**13n**, exhibited inhibitory activities being slightly superior to the one of benzamide (*S*)-**13a**. In case of the synthesized acylamides containing benzannelated heterocycles, quinoline derivatives (*S*)-**13f** and (*S*)-**13e** were found to be equally or slightly less active compared to (*S*)-**13a**. However, among the investigated indole derivatives, superior inhibitory activities were observed. While indole-2-carboxamide (*S*)-**13g** was found to be the least potent indole derivative with a *K*_*i*_ value of 87 nM, indole-3-carboxamide (*S*)-**13h**, indole-4-carboxamide (*S*)-**13i**, and indole-5-carboxamide (*S*)-**13j** exhibited *K*_*i*_ values between 9 and 15 nM, thus representing the most potent LpxC inhibitors of the synthesized series of acylamides.

As *Pseudomonas aeruginosa* is one of the so-called ESKAPE pathogens,^56^ being the leading cause of nosocomial infections throughout the world, and carbapenem-resistant *P. aeruginosa* strains were assigned the highest priority (priority 1: critical) within the “WHO priority pathogens list for R&D of new antibiotics” (WHO 2017),^57^ new antibacterial agents combating these bacteria are urgently required. Thus, our newly developed LpxC inhibitors should be tested for inhibitory activity toward *P. aeruginosa* LpxC, which shares 57% sequence identity with *E. coli* LpxC.^28^ Therefore, a liquid chromatography with tandem mass spectrometry (LC–MS/MS)-based *P. aeruginosa* LpxC assay was established. Even though UDP-3-*O*-[(*R*)-3-hydroxydecanoyl]-*N*-acetylglucosamine is the natural substrate of *P. aeruginosa* LpxC,^58^ the *E. coli* LpxC substrate UDP-3-*O*-[(*R*)-3-hydroxymyristoyl]-*N*-acetylglucosamine (**1**) was employed, as LpxC had been found to be relatively nonspecific with respect to the acyl chain length of the substrate.^59−61^ After incubation and subsequent termination of the enzymatic reaction, the samples were subjected to LC–MS/MS analysis. Following the chromatographic separation of substrate **1** and deacetylated product **2**, the eluted compounds were ionized and subjected to fragmentation. Three multiple reaction monitoring (MRM) transitions were detected per compound [precursor ions: *m*/*z* (substrate **1**) 832; *m*/*z* (deacetylated product **2**) 790; product ions: *m*/*z* (product 1) 385, collision energy = −60 V; *m*/*z* (product 2) 159, collision energy = −80 V; *m*/*z* (product 3) 79, collision energy = −140 V]. The MRM transitions 832 → 79 of substrate **1** and 790 → 79 of product **2** were used as quantifiers; the other mass transitions were used as qualifiers.

Generally, the LC–MS/MS-based method represents an alternative to analyze compounds, which cannot be tested in the fluorescence-based assay, like primary amines and intrinsically fluorescent compounds. Additionally, it offers the advantage that lower substrate concentrations (10%) are used in the established protocol compared to the fluorescence-based assay, in which the detection of respectively low product concentrations would be difficult to detect due to inherently high background fluorescence. Thus, the conditions of the established LC–MS/MS-based enzyme assay were employed to determine the *K*_M_ value for the *P. aeruginosa* LpxC-catalyzed deacetylation of UDP-3-*O*-[(*R*)-3-hydroxymyristoyl]-*N*-acetylglucosamine (**1**) (Figure S9, Supporting Information), which was found to be 4.7 μM. Additionally, the *K*_M_ of *E. coli* LpxC C63A could be determined using the LC–MS/MS-based method (Figure S8, Supporting Information, vide supra).

With respect to their inhibitory activities toward *P. aeruginosa* LpxC, the investigated amides showed the same trends as previously observed against *E. coli* LpxC C63A (Table 3). Remarkably, the determined *K*_*i*_ values against *P. aeruginosa* LpxC were generally lower by a factor of around 1.3 to 4.5 than the ones against *E. coli* LpxC C63A. Only biphenyl derivative (*S*)-**13c** was found to exhibit a higher *K*_*i*_ value against *P. aeruginosa* LpxC than against *E. coli* LpxC C63A. Again, indole-3-carboxamide (*S*)-**13h**, indole-4-carboxamide (*S*)-**13i**, and indole-5-carboxamide (*S*)-**13j** were found to be the most potent LpxC inhibitors of the investigated series of acylamides, with compounds (*S*)-**13h** and (*S*)-**13j** exhibiting *K*_*i*_ values against *P. aeruginosa* LpxC in the single-digit nanomolar range. As in accordance with the results of the *E. coli* LpxC C63A assay, the (*R*)-configured indole-3-carboxamide (*R*)-**13h** showed considerably lower inhibitory activity toward *P. aeruginosa* LpxC than its enantiomer (*S*)-**13h**, the investigation of the other (*R*)-configured acylamides in the *P. aeruginosa* LpxC assay was omitted.

The isoserine-based amides were additionally tested for antibacterial activity (Table 3). Also in case of these compounds, the most potent inhibitors of *E. coli* LpxC C63A, namely indole derivatives (*S*)-**13h**, (*S*)-**13i**, and (*S*)-**13j**, were found to exhibit the lowest MIC values against *E. coli* BL21(DE3) (1–4 μg/mL) and *E. coli* D22 (0.031 μg/mL). Among the moderately active LpxC inhibitors (*S*)-**13c**, (*S*)-**13g**, (*S*)-**13k**, (*S*)-**13l**, and (*S*)-**13m**, biphenyl derivative (*S*)-**13c** showed no antibacterial activity against *E. coli* BL21(DE3). In contrast, indole-2-carboxamide (*S*)-**13g**, pyrrole (*S*)-**13k**, as well as pyrazole derivatives (*S*)-**13l** and (*S*)-**13m** were found to be only one serial dilution step less active against *E. coli* D22 (0.063 μg/mL) and about one to two serial dilution steps less active against *E. coli* BL21(DE3) (4–8 μg/mL) compared to indole derivatives (*S*)-**13h**, (*S*)-**13i**, and (*S*)-**13j**. Particularly, pyrrole derivative (*S*)-**13k** and pyrazole derivative (*S*)-**13l** caused the largest zones of growth inhibition in the performed disc diffusion assays against *E. coli* BL21(DE3) and *E. coli* D22.

As the (*S*)-configured isoserine-based amides exhibited higher antibacterial activities than their (*R*)-configured enantiomers, these compounds were further characterized by testing them against *E. coli* TOP10 and a series of Gram-negative wild-type strains representing clinically relevant species, namely *E. coli* ATCC 35218, *Klebsiella pneumoniae* ATCC 700603, and *P. aeruginosa* ATCC 27853. In the performed disc diffusion assays, indole derivatives (*S*)-**13h**, (*S*)-**13i**, and (*S*)-**13j** caused promising halos of growth inhibition. However, again the largest zones of growth inhibition were found for pyrrole derivative (*S*)-**13k** and pyrazole derivative (*S*)-**13l**, which showed considerable activity against *K. pneumoniae* ATCC 700603 and *P. aeruginosa* ATCC 27853.

### Molecular Docking Studies

**Figure 6:**
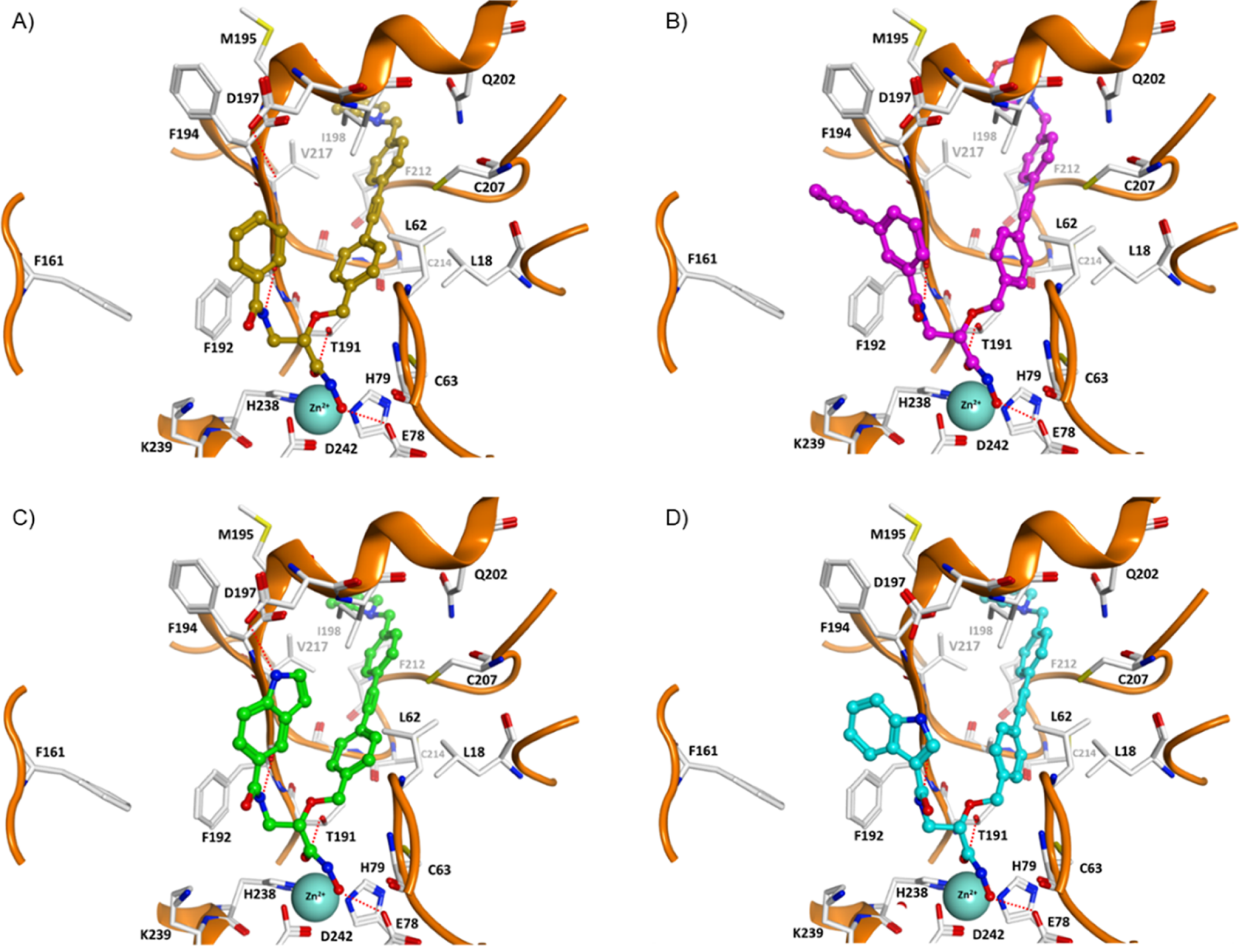
Docking poses of potent LpxC inhibitors from the current study (PDB ID 4MQY). (A) (*S*)-**13a**, (B) (*S*)-**13c**, (C) (*S*)-**13j**, (D) (*S*)-**13h**. Inhibitors are shown in ball-and-stick mode. The zinc ion is shown as a cyan sphere (van der Waals radius), and hydrogen bonds are shown as red-colored dashed lines.

To rationalize the SAR of the inhibitors synthesized, molecular docking into the *E. coli* and *P. aeruginosa* LpxC structures (PDB ID: 4MQY and 5VWM) was carried out using the program Glide (Figure 6, Table S1, for details see Computational Methods). The docked inhibitors showed a similar binding mode in both enzymes, which resembled the commonly observed binding mode of known LpxC inhibitors, such as CHIR-090 (Figure S1) and LPC-011 (**3a**, Figure 2). Since more in vitro data were obtained for the *E. coli* LpxC orthologue and since the observed inhibition data for both forms show high correlation, only the docking results observed for the *E. coli* orthologue are discussed here. Generally, the hydroxamic acid moiety of the inhibitors coordinates to the Zn^2+^-ion in a bidentate fashion. The hydroxamic acid group further exhibits hydrogen bond interactions with E78 and T191. The lipophilic side chain is located in a hydrophobic tunnel comprising L18, L62, I198, C207, F212, and V217. The substituents introduced at the α-carbon atom of hydroxamic acid **9** (indicated by the arrow in Figure 4) protrude into the UDP pocket of LpxC and can be used for adding pocket-filling substituents. From the first series of synthesized inhibitors (Table 1), the (2*S*)-configured isoserine-based amide (*S*)-**13a** demonstrated the most favorable LpxC inhibitory activity. The docking pose of (*S*)-**13a** showed an additional hydrogen bond between the amide group of the side chain and the backbone CO of F192, whereas the terminal phenyl group is involved in aromatic interactions with the side chain of F192 (Figure 6A). These interactions might contribute to the enhanced inhibitory activity compared to the analogues that do not show this hydrogen bond [e.g., ether (*S*)-**11a**]. Due to the favorable hydrogen bond of the amide linker, it was retained for further optimization of the derivatives (Table 3). The size of the aromatic substituent is another option to improve the binding in the UDP pocket, as can be seen in the interaction of (*S*)-**13c** (Figure 6B). The additional aromatic substituent in the meta-position forms aromatic interactions with F192 and F194, which is not the case with the para-substituted isomer (*S*)-**13b**. The phenyl ring located in the para-position of (*S*)-**13b** does not fit properly into the UDP-binding pocket and results in a loss of the correct bidentate chelation of the hydroxamic acid group.

In case of the most active inhibitor (*S*)-**13j**, the terminal indole ring is involved in a hydrogen bond with D197 (Figure 6C). In case of the (*R*)-configured isomers [e.g., (*R*)-**13j**, (*R*)-**13c**], the binding is less favorable, and in all cases, a hydrogen bond between the amide and the backbone CO of F192 was not observed. In addition, the interaction of the aromatic substituents is less favorable indicated by weaker docking scores. For the second and third most potent inhibitors (*S*)-**13h** and (*S*)-**13i**, a direct hydrogen bond between the indole and D197 was not observed. However, the distance is short enough that a water molecule could mediate a hydrogen bond. The aromatic interaction with F192 is similar to that observed for (*S*)-**13j** (shown exemplarily for (*S*)-**13h** in Figure 6D).

In order to test the stability and the dynamic behavior of potent inhibitors and their less active enantiomers, we selected the two pairs (*S*)-**13c**/(*R*)-**13c** and (*S*)-**13j**/(*R*)-**13j** and analyzed them by means of molecular dynamics (MD) simulations. For comparison, we also simulated the crystal structure of LpxC in complex with the inhibitor LPC-138 (Figure S1, PDB structure 4MQY).^62^ The MD simulations of the crystal structure (PDB ID: 4MQY) and the two potent inhibitors (*S*)-**13c** and (*S*)-**13j** showed that the complexes and the interactions of the inhibitors are very stable (Figures S2–S7). This is reflected in small root-mean-square deviation (RMSD) changes for the ligand, the chelation of the zinc ion, and the stable hydrogen bonds. In contrast, simulations of the less active stereoisomers (*R*)-**13c** and (*R*)-**13j** showed that although the protein structure was stable, the inhibitors showed significant changes in the interactions, including loss of chelation in case of (*R*)-**13c**.

### Further Biological Evaluation

#### Inhibition of the Virulence Factor LasB from *P. aeruginosa*

The elastase LasB of *P. aeruginosa* is a secreted metalloprotease, which actively controls the infection process and is considered an important virulence factor in this pathogen.^63,64^ Thus, inhibitors of LasB are described to reduce pathogenicity without being bactericidal.^65,66^ These inhibitors could be applied together with a standard-of-care antibiotic in combination therapy. Hence, an additional antivirulence effect via LasB inhibition could be beneficial for the LpxC inhibitors, leading to, e.g., a dual LpxC/LasB inhibitor.

As aldotetronic acid derivative (2*S*,3*S*)-**10** as well as its stereoisomer (2*S*,3*R*)-**10** had been found to exhibit promising inhibitory activities against LasB,^32^ the newly synthesized hydroxamic acids were also evaluated for inhibitory activity toward the zinc metalloprotease (Table 4). As in case of the aldotetronic acid derivatives (2*S*)-configuration had been found to be crucial for inhibitory activity toward LasB, generally only the (*S*)-enantiomers of the newly synthesized compounds were investigated.

**Table 4:**
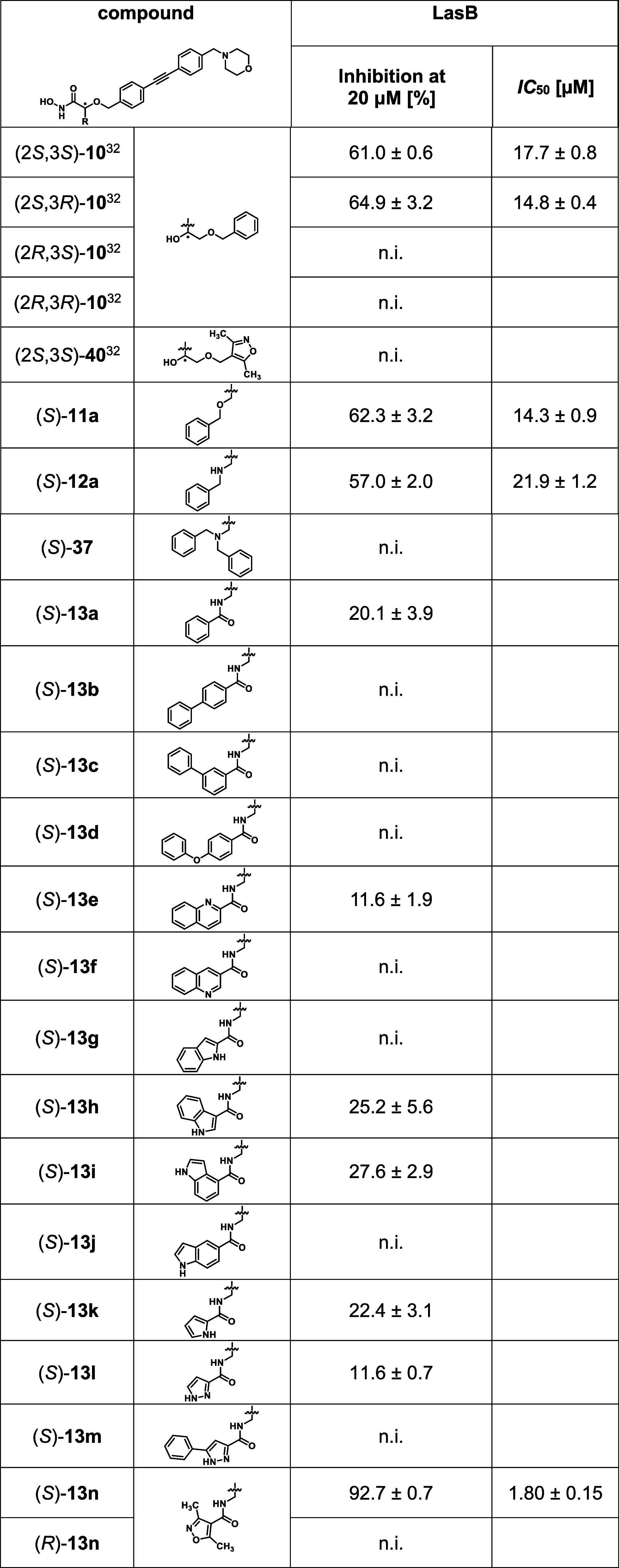
In Vitro Activities of the Newly Synthesized Hydroxamic Acids against LasB^a^

a n.i.: <10% inhibition.

In case of benzyl ether (*S*)-**11a** and benzylamine (*S*)-**12a**, IC_50_ values of 14.3 and 21.9 μM were found, respectively. These were in the same range as those determined for aldotetronic acid derivatives (2*S*,3*S*)-**10** and (2*S*,3*R*)-**10**. In contrast, tertiary amine (*S*)-**37** exhibited no inhibitory activity.

While benzamide (*S*)-**13a** showed moderate inhibition of the enzymatic activity of LasB at a concentration of 20 μM, being similarly potent as indole derivatives (*S*)-**13h** and (*S*)-**13i** as well as pyrrole derivative (*S*)-**13k** (20–30% inhibition at 20 μM), little to no inhibitory activity was found for biphenyl derivatives (*S*)-**13b** and (*S*)-**13c**, diphenyl ether (*S*)-**13d**, quinoline derivatives (*S*)-**13e** and (*S*)-**13f**, indole derivatives (*S*)-**13g** and (*S*)-**13j**, as well as pyrazole derivatives (*S*)-**13l** and (*S*)-**13m**. While in case of the previously investigated aldotetronic acid derivatives, the replacement of the benzyl group by a (3,5-dimethylisoxazol-4-yl)methyl substituent was found to be detrimental for LasB inhibition [cf. (2*S*,3*S*)-**40**], the exchange of the benzoyl moiety of isoserine-based benzamide (*S*)-**13a** by a 3,5-dimethylisoxazole-4-carbonyl group, leading to hydroxamic acid (*S*)-**13n**, caused a considerable increase in inhibitory activity. With an IC_50_ value of 1.8 μM, isoxazole (*S*)-**13n** is the most potent LasB inhibitor of the investigated series of hydroxamic acids and represents a promising starting point for further optimization steps.

To confirm the superiority of the stereoisomers with (*S*)-configuration in the α-position of the hydroxamate moiety, isoxazole derivative (*R*)-**13n**, the enantiomer of (*S*)-**13n**, was evaluated for inhibitory activity toward LasB. Just like in case of the aldotetronic acid derivatives, the inversion of configuration in the α-position of isoserine-based amide (*S*)-**13n** was detrimental for LasB inhibition, with enantiomer (*R*)-**13n** exhibiting no inhibitory activity.

#### In Vitro ADMET Properties and Off-Targets

As indole derivatives (*S*)-**13h** and (*S*)-**13j** as well as pyrazole derivative (*S*)-**13l** were found to exhibit promising antibacterial as well as LpxC inhibitory activities, the three frontrunner compounds were further investigated regarding their in vitro absorption–distribution–metabolism–excretion–toxicity (ADMET) profile (Table 5).

**Table 5:**
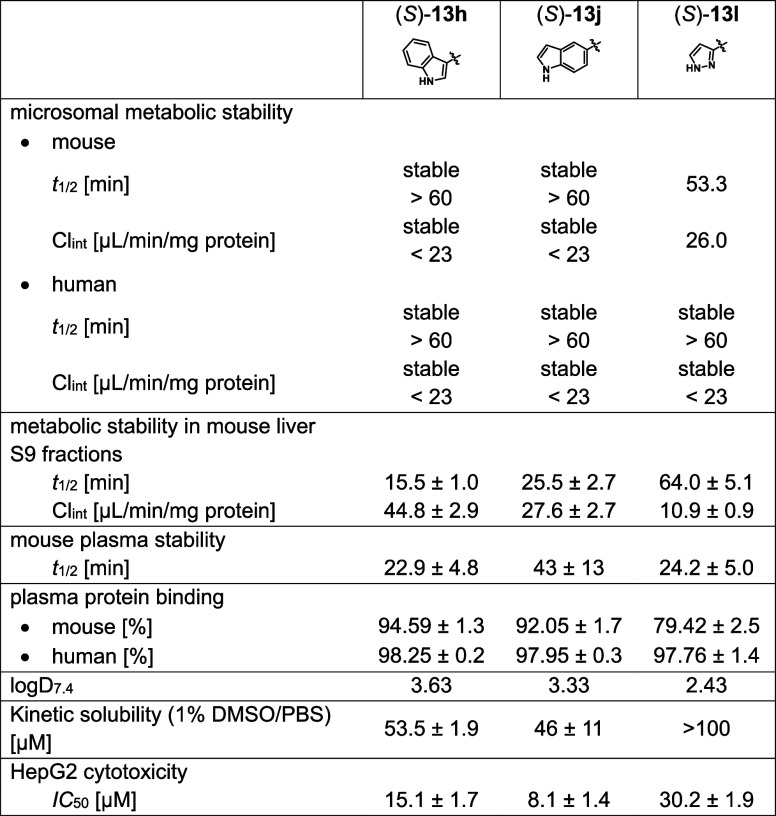
In Vitro ADMET Profile of (*S*)-**13h**, (*S*)-**13j**, and (*S*)-**13l**

Metabolism with liver microsomes did show that the compounds were generally stable in humans and mice, with pyrazole derivative (*S*)-**13l** being the least stable with a half-life of around 53 min in the presence of mouse liver microsomes. In contrast to the metabolism with liver microsomes, the metabolism in liver S9 fractions revealed lower stability of indole derivative (*S*)-**13h** with a clearance of around 45 μL/min/mg protein, whereas indole derivative (*S*)-**13j** had a significantly lower clearance similar to the one observed in microsomes. Superior metabolic stability was found for the pyrazole-substituted compound (*S*)-**13l** with a moderate half-life of more than 60 min. Also, the plasma stability of the compounds in mouse plasma was generally low with half-lives between 22.9 min [(*S*)-**13h**] and 43.1 min [(*S*)-**13j**].

In general, plasma protein binding in humans was high with the compounds having a plasma protein binding above 97%. Interestingly, the compounds exhibited lower plasma protein binding in mice compared to humans, which was considerably pronounced in case of pyrazole derivative (*S*)-**13l** (<80%), resulting in a much higher unbound fraction.

With a log *D*_7.4_ of 2.43, the pyrazole-substituted compound (*S*)-**13l** is also the least lipophilic of the three compounds. And while the two indole derivatives showed similar kinetic solubility around 50 μM, pyrazole-substituted (*S*)-**13l** was found to be soluble up to 100 μM. This compound was also the least cytotoxic among the ones tested with an IC_50_ value of 30.2 μM (vs 15.1 and 8.1 μM).

Since LpxC and LasB are zinc-dependent enzymes, we investigated potential off-target activity of the three frontrunners on mammalian zinc-dependent enzymes. Our panel comprised three representative matrix-metalloproteases (MMP1–3) and tumor necrosis factor-α converting enzyme (TACE, also known as ADAM17) (Table 6). MMP inhibition was mostly below 20% at 100 μM, except for MMP2: here, (*S*)-**13j** resulted in ∼50% inhibition at 100 μM and (*S*)-**13l** gave an IC_50_ value of 67 μM. Both compounds were also active against TACE, again with stronger inhibition by (*S*)-**13l** [IC_50_ 5.4 μM vs 38% inhibition at 100 μM by (*S*)-**13j**]. Considering the potent activity on LpxC, this compound still shows excellent to moderate selectivity (selectivity factor 105 for MMP2 and 8.4 for TACE).

**Table 6:**
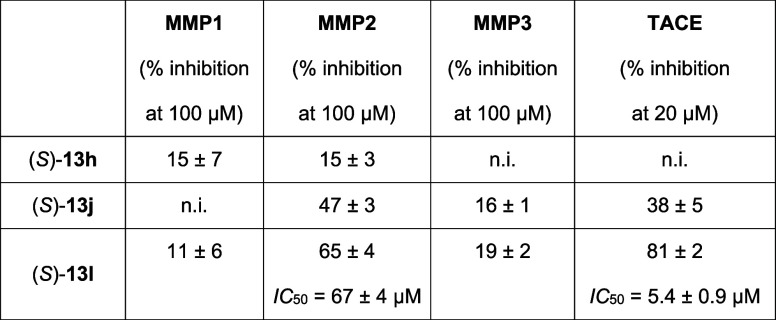
In Vitro Activities of Hydroxamic Acids (*S*)-**13l**, (*S*)-**13h**, and (*S*)-**13j** against MMPs 1–3 and TACE (ADAM-17)^a^

a n.i.: <10% inhibition.

## Conclusions

In this study, we aimed to generate LpxC inhibitors that address the UDP-binding pocket, which is located in close proximity to the Zn^2+^-chelating hydroxamate moiety of our previously reported inhibitors.

Thus, fragment screening was utilized as the strategy to optimize our benzyloxyacetohydroxamic acid derivatives. We used STD-NMR, WaterLogsy, and subsequent NMR-ILOE experiments to identify nine out of 650 fragments, which bind into the UDP-binding pocket of LpxC near the methylene group in the α-position of the hydroxamate moiety of LpxC inhibitor **9**, and four substructures (biphenyl, indole, quinoline, and pyrazole) were selected to be linked to the benzyloxyacetic acid scaffold of hydroxamic acid **9**.

In parallel, in order to find an optimal linker to connect the structures of interest with the benzyloxyacetohydroxamic acid derivatives, a phenyl ring as an exemplary substituent was connected to the scaffold of hydroxamic acid **9** via structural elements of different lengths and structures. The biological evaluation of the investigated tetrose- and glyceric acid-derived ethers as well as the isoserine-based amides and secondary and tertiary amines revealed the amide linker of isoserine derivative (*S*)-**13a** to be the most favorable with respect to inhibitory activity toward LpxC (Table 1). Besides holding the introduced fragment in an appropriate distance to the benzyloxyacetohydroxamic acid scaffold, molecular docking studies indicate that the amide linker undergoes favorable hydrogen bonding with the enzyme (backbone CO of F192).

Among the synthesized amides exhibiting fragment-derived substituents as well as further acyl residues, the (*S*)-configured indole derivatives (*S*)-**13h**, (*S*)-**13i**, and (*S*)-**13j** as well as pyrazole derivatives (*S*)-**13l** and (*S*)-**13m** exhibited highest inhibitory activities toward *E. coli* LpxC C63A as well as *P. aeruginosa* LpxC (Table 3). Thus, linking these two substructures, which were identified through fragment screening using NMR spectroscopy, to the scaffold of hydroxamic acid **9** resulted in more potent LpxC inhibitors than linking the other investigated residues. Consequently, by appending a privileged fragment via a suitable linker to the scaffold of hydroxamic acid **9**, this micromolar LpxC inhibitor could be converted into the one-digit nanomolar LpxC inhibitor (*S*)-**13j** [*K*_*i*_ (*Ec*LpxC C63A) = 9.5 nM; *K*_*i*_ (*Pa*LpxC): 5.6 nM]. Therefore, NMR-based fragment screening proved to be an appropriate strategy for the fragment-based drug discovery of LpxC inhibitors specifically addressing the enzyme’s UDP-binding site.

Molecular docking into an *E. coli* LpxC structure indicated that the substituents introduced at the α-carbon atom of hydroxamic acid **9** protrude into the UDP pocket of LpxC with the amide linker of the (*S*)-configured isomers undergoing hydrogen bonding with the backbone CO of F192. The aromatic substituents enter the substrate-ribose region of the UDP-binding pocket through the amide linker (Figure 4). In case of the most active inhibitor (*S*)-**13j**, besides undergoing aromatic interactions with the side chain of F192, the indole ring is involved in a hydrogen bond with D197 being indicative of its high inhibitory activity (Figure 6).

Indole derivatives (*S*)-**13h**, (*S*)-**13i**, and (*S*)-**13j**, the most potent LpxC inhibitors of the investigated isoserine-based amides, were found to exhibit the lowest MIC values against *E. coli* BL21(DE3) (1–4 μg/mL) and *E. coli* D22 (0.031 μg/mL) (Table 3). In the performed disc diffusion assays against the Gram-negative wild-type strains *E. coli* ATCC 35218, *K. pneumoniae* ATCC 700603, and *P. aeruginosa* ATCC 27853, indole derivatives (*S*)-**13h**, (*S*)-**13i**, and (*S*)-**13j** caused promising halos of growth inhibition. However, the largest zones of growth inhibition were found for pyrrole derivative (*S*)-**13k** and pyrazole derivative (*S*)-**13l**.

As our previously reported benzyloxyacetohydroxamic acid derivatives had shown inhibitory activity toward the elastase LasB of *P. aeruginosa*,^32^ the newly developed isoserine-based amides were also tested against this important virulence factor (Table 4). While indole derivatives (*S*)-**13g**, (*S*)-**13h**, (*S*)-**13i**, and (*S*)-**13j** as well as pyrazole derivatives (*S*)-**13l** and (*S*)-**13m** exhibited little to no inhibitory activity toward LasB, the (*S*)-configured 3,5-dimethylisoxazol-4-yl derivative (*S*)-**13n** was found to be the most potent LasB inhibitor of the investigated series of hydroxamic acids, exhibiting an IC_50_ value of 1.8 μM. Thus, the isoserine-based amides represent a promising starting point for further optimization steps, as depending on the introduced acyl residue, the compounds can be developed into selective or dual inhibitors of the two Zn^2+^-dependent enzymes.

Additionally, the three frontrunner compounds (*S*)-**13h**, (*S*)-**13j**, and (*S*)-**13l** were tested for inhibitory activity toward several mammalian zinc-dependent enzymes (MMP1–3 and TACE), representing potential off-targets (Table 6). The compounds exhibited good selectivities for LpxC, with pyrazole derivative (*S*)-**13l** being the least selective compound, particularly with respect to MMP2 and TACE inhibition.

The three frontrunner compounds, exhibiting promising antibacterial as well as LpxC inhibitory activities, were additionally investigated regarding their in vitro ADMET profile (Table 5). While the metabolic stability of the compounds in the presence of human and mouse liver microsomes was generally high, particularly the stability of indole derivative (*S*)-**13h** in the presence of mouse liver S9 fractions, including phase I and phase II reactions, was low. These findings indicate, that the metabolism of these compounds might not be mainly CYP-mediated, as S9 fractions harbor additional non-CYP-enzymes, or might be a cause of phase II metabolism.^67,68^ As plasma stability of the compounds in mouse plasma was also found to be low, their stability needs to be improved in further optimization steps.

Among the three frontrunner compounds, the pyrazole-substituted compound (*S*)-**13l** was the least lipophilic with a log *D*_7.4_ of 2.43. This is in line with the higher kinetic solubility and the considerably lower plasma protein binding in mice of the compound compared to the two indole derivatives. In comparison to marketed Gram-negative antibacterials, which exhibit an average *c* log *D* of −2.8,^69^ all of our compounds are substantially more lipophilic. However, the whole class of LpxC inhibitors proved to be an exception to the trend that primarily hydrophilic compounds exhibit potent *P. aeruginosa* and *E. coli* activity.^70^ Nonetheless, as the UDP-binding site of LpxC is solvent-exposed, in further optimization steps of the developed isoserine-based amides, additional polar functional groups could be introduced at the substituent, addressing the UDP-binding site to reduce the lipophilicity of the inhibitors. Additionally, having found compounds tightly binding to the UDP-binding site, their lipophilic side chain could be shortened to reduce the impact of this hydrophobic moiety on the polarity of the compounds.

To date, a lot of preclinical work has been done on LpxC inhibitors with promising results, showing the feasibility of developing hydroxamate-based LpxC inhibitors into clinical candidates, which in contrast to ACHN-975 (Figure S1), the first LpxC inhibitor to reach human clinical trials, do not exhibit cardiovascular toxicity.^22−24,71,72^ Altogether, our initial screening revealed some ADMET parameters that need to be considered during the further development of our newly developed isoserine-based amides, paving the way for multiparameter optimization with the aim to merge compound features favorable for activity with those improving the in vitro ADMET profile as well as selectivity.

## Experimental Section

### Chemistry, General

All experiments involving water- or air-sensitive compounds were carried out under anhydrous conditions (N_2_ atmosphere). Reagents were purchased from various suppliers and were used without further purification unless otherwise noted. Anhydrous solvents were purchased from Acros Organics (extra dry over molecular sieves). Solvents for flash column chromatography were purchased in technical grade and distilled prior to use. Ultrapure water for reversed-phase chromatography was purified using a Sartorius Arium pro system (Sartopore 0.2 μm, UV). Acetonitrile (ACN) for reversed-phase chromatography was purchased from VWR (HPLC grade). Flash column chromatography on silica gel was performed using Macherey Nagel silica gel 60 M (0.040–0.063 mm). Parentheses include the diameter of the column, fraction size, eluent, and *R*_*f*_ value. Thin-layer chromatography was performed on Macherey Nagel precoated TLC sheets (ALUGRAM Xtra SIL G/UV_254_). Visualization was achieved by heat-staining using a cerium molybdate dipping bath [Ce(SO_4_)_2_ (1.8 g), (NH_4_)_6_Mo_7_O_24_ × 4H_2_O (45 g), conc. H_2_SO_4_ (45 g), H_2_O (900 mL)]. Automatic reversed-phase flash column chromatography was performed using Biotage SNAP Ultra C18 columns on an Isolera One (Biotage). Product-containing fractions were combined and lyophilized using a Christ Alpha 2–4 LDplus freeze-dryer. Automatic normal-phase flash column chromatography was performed using Biotage SNAP Ultra HP-Sphere columns on an Interchim puriFlash XS 420 system. Product-containing fractions were combined, and the solvent was removed in vacuo. Melting points were measured with a Büchi Melting Point M-565 and are uncorrected. Optical rotation α [deg] was determined with a P8000 polarimeter (A. Krüss Optronic GmbH); path length 1 dm, wavelength 589 nm (sodium D line); the unit of the specific rotation [α]_D_^20^ (deg mL dm^–1^ g^–1^) is omitted; the concentration of the sample c (mg mL^–1^) and the solvent used are given in brackets. IR spectra were recorded on a Bruker Alpha FT-IR Platinum ATR spectrophotometer. NMR spectra were recorded at ambient temperature on Bruker Avance I 400, DRX 500, and Avance III 600 instruments. High-resolution mass spectrometry was performed using an Agilent 6224 ESI-TOF instrument via flow injection analysis in 50:50 water + 0.1% formic acid/acetonitrile + 0.1% formic acid at a flow rate of 0.3 mL/min via electrospray ionization. HPLC methods for the determination of product purity: method 1: VWR Hitachi equipment; UV/vis detector: 5420; autosampler: 5260; pump: 5160; column: LiChrospher 60 RP-select B (5 μm); LiChroCART 250-4 mm cartridge; flow rate: 1.00 mL/min; injection volume: 5.0 μL; detection at λ = 210 nm for 30 min; solvents: (A) water with 0.05% (V/V) trifluoroacetic acid, (B) acetonitrile with 0.05% (V/V) trifluoroacetic acid; gradient elution: (A %): 0–4 min: 90%, 4–29 min: gradient from 90 to 0%, 29–31 min: 0%, 31–31.5 min: gradient from 0 to 90%, 31.5–40 min: 90%; data were collected and evaluated by Chromaster software. Method 2: VWR Hitachi equipment; UV/vis detector: 5420; autosampler: 5260; pump: 5160; column: Phenomenex Gemini 5 μm C6-Phenyl 110 Å; LC Column 250 × 4.6 mm; flow rate: 1.00 mL/min; injection volume: 5.0 μL; detection at λ = 254 nm for 20 min; solvents: (A) acetonitrile/10 mM ammonium formate = 10:90 with 0.1% formic acid, (B) acetonitrile/10 mM ammonium formate = 90:10 with 0.1% formic acid; gradient elution: (A %): 0–5 min: 100%, 5–12 min: gradient from 100 to 0%, 12–20 min: 0%, 20–22 min: gradient from 0 to 100%, 22–30 min: 100%; data were collected and evaluated by Chromaster software. Method 3: VWR Hitachi equipment; UV/vis detector: 5420; autosampler: 5260; pump: 5160; column: LiChrospher 60 RP-select B (5 μm); LiChroCART 250-4 mm cartridge; flow rate: 1.00 mL/min; injection volume: 5.0 μL; detection at λ = 210 nm for 40 min; solvents: (A) water with 0.05% (V/V) trifluoroacetic acid, (B) acetonitrile with 0.05% (V/V) trifluoroacetic acid: gradient elution: (A %): 0–4 min: 90%, 4–29 min: gradient from 90 to 0%, 29–41 min: 0%, 41–41.5 min: gradient from 0 to 90%, 41.5–50 min: 90%; data were collected and evaluated by Chromaster software. Method 4: KNAUER equipment; UV/vis detector: Azura UVD 2.1S; pump: Azura P4.1S; column: Daicel Chiralpak IA; flow rate: 1.00 mL/min; injection: manual, injection valve; injection volume: 300 μL; detection at λ = 230 nm for 20 min; solvent: isohexane/isopropanol = 97.5:2.5; data were collected and evaluated by the software ClarityChrom Preparativ Version 5.0.5.98. The purity of all test compounds was ≥95%.

### Synthetic Procedures

#### (*S*)-*tert*-Butyl(oxiran-2-ylmethoxy)diphenylsilane (**15**)

Under a N_2_ atmosphere, *tert*-butyldiphenylsilyl chloride (9.5 mL, 10 g, 37 mmol) was added to an ice-cooled solution of (*R*)-glycidol (2.0 mL, 2.3 g, 31 mmol) and imidazole (3.0 g, 43 mmol) in dry dichloromethane (60 mL). After stirring the reaction mixture for 10 min at 0 °C, the ice-bath was removed and the mixture was stirred for 24 h at ambient temperature. Afterward, the mixture was washed with water and brine. The organic layer was dried (Na_2_SO_4_), filtered, and the solvent was removed in vacuo. The residue was purified by flash column chromatography (Ø = 6 cm, *h* = 22 cm, *V* = 50 mL, petroleum ether/ethyl acetate = 10:1, *R*_*f*_ = 0.59) to give **15** as a colorless oil (8.3 g, 27 mmol, 86%). [α]_D_^20^ = +2.2 (1.9, methanol); ^1^H NMR (DMSO-*d*_6_): δ [ppm] = 1.00 (s, 9H, SiC(C*H*_3_)_3_), 2.57 (dd, *J* = 5.2/2.7 Hz, 1H, C*H*_2_CHCH_2_OSi), 2.71 (dd, *J* = 5.2/4.2 Hz, 1H, C*H*_2_CHCH_2_OSi), 3.10–3.17 (m, 1H, CH_2_C*H*CH_2_OSi), 3.59 (dd, *J* = 11.9/5.3 Hz, 1H, CH_2_CHC*H*_2_OSi), 3.91 (dd, *J* = 11.9/2.7 Hz, 1H, CH_2_CHC*H*_2_OSi), 7.39–7.50 (m, 6H, 3′-H_diphenylsilyl_, 4′-H_diphenylsilyl_, 5′-H_diphenylsilyl_), and 7.60–7.67 (m, 4H, 2′-H_diphenylsilyl_, 6′-H_diphenylsilyl_); ^13^C NMR (DMSO-*d*_6_): δ [ppm] = 18.8 (1C, Si*C*(CH_3_)_3_), 26.5 (3C, SiC(*C*H_3_)_3_), 43.4 (1C, *C*H_2_CHCH_2_OSi), 51.7 (1C, CH_2_*C*HCH_2_OSi), 64.4 (1C, CH_2_*C*HCH_2_OSi), 127.9 (4C, C-3′_diphenylsilyl_, C-5′_diphenylsilyl_), 129.9 (2C, C-4′_diphenylsilyl_), 132.76 (1C, C-1′_diphenylsilyl_), 132.79 (1C, C-1′_diphenylsilyl_), 135.0 (2C, C-2′_diphenylsilyl_, C-6′_diphenylsilyl_), and 135.1 (2C, C-2′_diphenylsilyl_, C-6′_diphenylsilyl_); IR (neat): ν̃ [cm^–1^] = 3071, 3050, 2998, 2958, 2930, 2893, 2857, 1472, 1427, 1390, 1361, 1254, 1159, 1106, 1089, 980, 917, 823, 738, 699, 612, 503, 486, and 425; HRMS (*m*/*z*): [M + Na]^+^ calcd for C_19_H_24_NaO_2_Si, 335.1438; found, 335.1461; HPLC (method 3): *t*_R_ = 28.3 min, purity 99.5%.

#### (*S*)-1-(Benzyloxy)-3-[(*tert*-butyldiphenylsilyl)oxy]propan-2-ol (**16**)

A mixture of **15** (380 mg, 1.2 mmol), erbium(III) triflate (75 mg, 0.12 mmol), and benzyl alcohol (0.15 mL, 160 mg, 1.4 mmol) was stirred at ambient temperature for 24 h. Then, a saturated aqueous solution of NaHCO_3_ (25 mL) was added, and the mixture was extracted with dichloromethane (3×). The combined organic layers were dried (Na_2_SO_4_), filtered, and the solvent was removed in vacuo. The residue was purified by flash column chromatography (Ø = 4 cm, *h* = 24 cm, *V* = 30 mL, petroleum ether/ethyl acetate = 4:1, *R*_*f*_ = 0.53) to give **16** as a colorless oil (380 mg, 0.89 mmol, 73%). [α]_D_^20^ = +4.1 (2.2, methanol); ^1^H NMR (DMSO-*d*_6_): δ [ppm] = 0.97 (s, 9H, SiC(C*H*_3_)_3_), 3.46 (dd, *J* = 9.7/5.6 Hz, 1H, C*H*_2_CHCH_2_OSi), 3.57 (dd, *J* = 9.7/4.8 Hz, 1H, C*H*_2_CHCH_2_OSi), 3.60 (dd, *J* = 10.1/5.2 Hz, 1H, CH_2_CHC*H*_2_OSi), 3.63 (dd, *J* = 10.1/5.8 Hz, 1H, CH_2_CHC*H*_2_OSi), 3.73–3.79 (m, 1H, CH_2_C*H*CH_2_OSi), 4.49 (s, 2H, OC*H*_2_Ph), 4.87 (d, *J* = 5.3 Hz, 1H, CHO*H*), 7.26–7.29 (m, 1H, 4′-H_benzyl_), 7.29–7.35 (m, 4H, 2′-H_benzyl_, 3′-H_benzyl_, 5′-H_benzyl_, 6′-H_benzyl_), 7.37–7.43 (m, 4H, 3″-H_diphenylsilyl_, 5″-H_diphenylsilyl_), 7.43–7.47 (m, 2H, 4″-H_diphenylsilyl_), and 7.60–7.65 (m, 4H, 2″-H_diphenylsilyl_, 6″-H_diphenylsilyl_); ^13^C NMR (DMSO-*d*_6_): δ [ppm] = 18.8 (1C, Si*C*(CH_3_)_3_), 26.6 (3C, SiC(*C*H_3_)_3_), 65.1 (1C, CH_2_CH*C*H_2_OSi), 70.0 (1C, CH_2_*C*HCH_2_OSi), 71.3 (1C, *C*H_2_CHCH_2_OSi), 72.3 (1C, O*C*H_2_Ph), 127.3 (1C, C-4′_benzyl_), 127.4 (2C, C-2′_benzyl_, C-6′_benzyl_), 127.80 (2C, C-3″_diphenylsilyl_, C-5″_diphenylsilyl_), 127.81 (2C, C-3″_diphenylsilyl_, C-5″_diphenylsilyl_), 128.2 (2C, C-3′_benzyl_, C-5′_benzyl_), 129.75 (1C, C-4″_diphenylsilyl_), 129.77 (1C, C-4″_diphenylsilyl_), 133.08 (1C, C-1″_diphenylsilyl_), 133.10 (1C, C-1″_diphenylsilyl_), 135.06 (2C, C-2″_diphenylsilyl_, C-6″_diphenylsilyl_), 135.08 (2C, C-2″_diphenylsilyl_, C-6″_diphenylsilyl_), and 138.5 (1C, C-1′_benzyl_); IR (neat): ν̃ [cm^–1^] = 3070, 2930, 2857, 1472, 1454, 1427, 1390, 1361, 1105, 1028, 998, 823, 738, 697, 611, 503, and 487; HRMS (*m*/*z*): [M + Na]^+^ calcd for C_26_H_32_NaO_3_Si, 443.2013; found, 443.2013; HPLC (method 3): *t*_R_ = 28.8 min, purity 98.3%.

#### (*R*)-3-(Benzyloxy)-2-[(4-iodobenzyl)oxy]propan-1-ol (**17**)

Under a N_2_ atmosphere, sodium hydride (60% suspension in paraffin oil, 700 mg, 18 mmol) was added to an ice-cooled solution of **16** (1.8 g, 4.2 mmol) in anhydrous THF (40 mL). After stirring the reaction mixture for 20 min at 0 °C, 4-iodobenzyl bromide (1.5 g, 5.0 mmol) was added, and the reaction mixture was stirred for an additional 15 min at 0 °C. Then, the ice-bath was removed, and the mixture was stirred for 24 h at ambient temperature. Afterward, methanol was added under ice-cooling, and the solvent was removed in vacuo. The residue was dissolved in THF (60 mL), tetrabutylammonium fluoride trihydrate (1.8 g, 5.8 mmol) was added, and the reaction mixture was stirred for 6 h at ambient temperature. Then, the solvent was removed in vacuo, water was added, and the mixture was extracted with ethyl acetate (3×). The combined organic layers were dried (Na_2_SO_4_), filtered, and the solvent was removed in vacuo. The residue was purified by flash column chromatography (Ø = 6 cm, *h* = 23 cm, *V* = 50 mL, petroleum ether/ethyl acetate = 2:1, *R*_*f*_ = 0.24) to give **17** as a colorless oil (1.2 g, 2.9 mmol, 71%). [α]_D_^20^ = −2.1 (2.0, methanol); ^1^H NMR (DMSO-*d*_6_): δ [ppm] = 3.44–3.61 (m, 5H, C*H*_2_C*H*C*H*_2_OH), 4.48 (s, 2H, CH_2_OC*H*_2_Ph), 4.55 (d, *J* = 13.0 Hz, 1H, CHOC*H*_2_Ar), 4.59 (d, *J* = 13.0 Hz, 1H, CHOC*H*_2_Ar), 4.68 (t, *J* = 5.5 Hz, 1H, CH_2_O*H*), 7.13–7.19 (m, 2H, 2″-H_4-iodophenyl_, 6″-H_4-iodophenyl_), 7.24–7.39 (m, 5H, 2′-H_phenyl_, 3′-H_phenyl_, 4′-H_phenyl_, 5′-H_phenyl_, 6′-H_phenyl_), and 7.65–7.71 (m, 2H, 3″-H_4-iodophenyl_, 5″-H_4-iodophenyl_); ^13^C NMR (DMSO-*d*_6_): δ [ppm] = 60.8 (1C, CH_2_CH*C*H_2_OH), 70.1 (1C, *C*H_2_CHCH_2_OH), 70.2 (1C, CHO*C*H_2_Ar), 72.3 (1C, CH_2_O*C*H_2_Ph), 79.1 (1C, CH_2_*C*HCH_2_OH), 93.1 (1C, C-4″_4-iodophenyl_), 127.38 (1C, C-4′_phenyl_), 127.40 (2C, C-2′_phenyl_, C-6′_phenyl_), 128.2 (2C, C-3′_phenyl_, C-5′_phenyl_), 129.6 (2C, C-2″_4-iodophenyl_, C-6″_4-iodophenyl_), 136.9 (2C, C-3″_4-iodophenyl_, C-5″_4-iodophenyl_), 138.5 (1C, C-1′_phenyl_), and 139.0 (1C, C-1″_4-iodophenyl_); IR (neat): ν̃ [cm^–1^] = 3413, 2862, 1589, 1483, 1453, 1401, 1364, 1205, 1058, 1006, 799, 735, 696, 609, and 469; HRMS (*m*/*z*): [M + Na]^+^ calcd for C_17_H_19_INaO_3_, 421.0271; found, 421.0263; HPLC (method 1): *t*_R_ = 23.8 min, purity 98.6%.

#### (4*R*,4′*R*,4″*R*,5′*R*)-2,2,2′,2′,2″,2″-Hexamethyl-4,4′:5′,4″-ter(1,3-dioxolane) (**21**)

The compound was synthesized according to the literature with minor variations:^41^ Concentrated sulfuric acid (1 mL) was added to a suspension of d-mannitol (10 g, 55 mmol) in acetone (250 mL), and the reaction mixture was stirred for 48 h at ambient temperature. Then, the mixture was neutralized with a 25% aqueous solution of NH_4_OH (4.7 mL) and Na_2_CO_3_ (6.2 g). The solvent was removed in vacuo, and the residue was recrystallized from ethanol to give **21** as a colorless solid (5.3 g, 18 mmol, 32%). mp 70 °C; [α]_D_^20^ = +16.3 (6.7, methanol); ^1^H NMR (DMSO-*d*_6_): δ [ppm] = 1.27 (s, 6H, CH_2_OC(C*H*_3_)_2_), 1.32 (s, 6H, (CHO)_2_OC(C*H*_3_)_2_), 1.33 (s, 6H, CH_2_OC(C*H*_3_)_2_), 3.83 (dd, *J* = 8.3/5.7 Hz, 2H, OC*H*_2_CHCH), 3.85–3.90 (m, 2H, HOCH_2_CHC*H*), 4.03 (dd, *J* = 8.3/6.6 Hz, 2H, OC*H*_2_CHCH), and 4.11–4.18 (m, 2H, OCH_2_C*H*CH); ^13^C NMR (DMSO-*d*_6_): δ [ppm] = 25.2 (2C, CH_2_OC(*C*H_3_)_2_), 26.3 (2C, CH_2_OC(*C*H_3_)_2_), 27.2 (2C, (CHO)_2_C(*C*H_3_)_2_), 65.4 (2C, O*C*H_2_CHCH), 75.7 (2C, OCH_2_*C*HCH), 78.6 (2C, OCH_2_CH*C*H), 108.7 (2C, CH_2_O*C*(CH_3_)_2_), and 109.4 (1C, (CHO)_2_*C*(CH_3_)_2_); IR (neat): ν̃ [cm^–1^] = 2991, 2958, 2936, 2880, 1368, 1257, 1211, 1147, 1063, 969, 844, 787, 509, and 408; HRMS (*m*/*z*): [M + Na]^+^ calcd for C_15_H_26_NaO_6_, 325.1622; found, 325.1613.

#### (1*R*,1′*R*)-1,1′-[(4*R*,5*R*)-2,2-Dimethyl-1,3-dioxolane-4,5-diyl]bis(ethane-1,2-diol) (**22**)

The compound was synthesized according to the literature with minor variations:^41,42^ After stirring **21** (1.0 g, 3.3 mmol) in 70% aqueous acetic acid (20 mL) for 90 min at 40 °C, the solvent was removed in vacuo and the residue was suspended in acetone (30 mL). The suspension was sonicated for 2 min, filtered, and the solvent was removed in vacuo. The residue was purified by flash column chromatography (Ø = 3 cm, *h* = 20 cm, *V* = 20 mL, dichloromethane/methanol = 20:1, *R*_*f*_ = 0.13) to give **22** as a colorless solid (510 mg, 2.3 mmol, 69%). mp 86 °C; [α]_D_^20^ = +27.0 (5.7, methanol); ^1^H NMR (DMSO-*d*_6_): δ [ppm] = 1.28 (s, 6H, C(C*H*_3_)_2_), 3.32–3.39 (m, 2H, HOC*H*_2_CHCH), 3.44–3.51 (m, 2H, HOCH_2_C*H*CH), 3.54 (ddd, *J* = 11.1/5.6/3.2 Hz, 2H, HOC*H*_2_CHCH), 3.84–3.88 (m, 2H, HOCH_2_CHC*H*), 4.44 (t, *J* = 5.7 Hz, 2H, CH_2_O*H*), and 5.06 (d, *J* = 4.5 Hz, 2H, CHO*H*); ^13^C NMR (DMSO-*d*_6_): δ [ppm] = 27.2 (2C, C(*C*H_3_)_2_), 63.0 (2C, HO*C*H_2_CHCH), 72.9 (2C, HOCH_2_*C*HCH), 79.1 (2C, HOCH_2_CH*C*H), and 108.3 (1C, *C*(CH_3_)_2_); IR (neat): ν̃ [cm^–1^] = 3312, 2936, 2884, 1433, 1371, 1335, 1221, 1192, 1166, 1116, 1068, 1035, 1011, 978, 875, 712, and 505; HRMS (*m*/*z*): [M + Na]^+^ calcd for C_9_H_18_NaO_6_, 245.0996; found, 245.0996.

#### (1*R*,1′*R*)-1,1′-[(4*R*,5*R*)-2,2-Dimethyl-1,3-dioxolane-4,5-diyl]bis[2-(benzyloxy)ethan-1-ol] (**23**)

A mixture of **22** (400 mg, 1.8 mmol) and dibutyltin oxide (970 mg, 3.9 mmol) was heated to reflux in toluene (20 mL) using a Dean–Stark trap for 16 h. Then, the solvent was removed in vacuo, and the residue was suspended in toluene (10 mL). Benzyl bromide (0.85 mL, 1.2 g, 7.2 mmol) and tetrabutylammonium iodide (660 mg, 1.8 mmol) were added and the mixture was heated to 70 °C for 36 h. Then, the solvent was removed in vacuo and the residue was purified by flash column chromatography (Ø = 6 cm, *h* = 18 cm, *V* = 50 mL, petroleum ether/ethyl acetate = 2:1, *R*_*f*_ = 0.29) to give **23** as a colorless oil (670 mg, 1.7 mmol, 92%). [α]_D_^20^ = +26.4 (4.9, methanol); ^1^H NMR (DMSO-*d*_6_): δ [ppm] = 1.28 (s, 6H, C(C*H*_3_)_2_), 3.41 (dd, *J* = 10.1/6.5 Hz, 2H, OC*H*_2_CHCH), 3.59 (dd, *J* = 10.1/3.2 Hz, 2H, OC*H*_2_CHCH), 3.66–3.75 (m, 2H, OCH_2_C*H*CH), 3.89–3.94 (m, 2H, OCH_2_CHC*H*), 4.48 (d, *J* = 12.3 Hz, 2H, OC*H*_2_Ph), 4.52 (d, *J* = 12.3 Hz, 2H, OC*H*_2_Ph), 5.24 (d, *J* = 5.0 Hz, 2H, CHO*H*), and 7.24–7.37 (m, 10H, 2′-H_phenyl_, 3′-H_phenyl_, 4′-H_phenyl_, 5′-H_phenyl_, 6′-H_phenyl_); ^13^C NMR (DMSO-*d*_6_): δ [ppm] = 27.2 (2C, C(*C*H_3_)_2_), 70.9 (2C, OCH_2_*C*HCH), 71.9 (2C, O*C*H_2_CHCH), 72.3 (2C, O*C*H_2_Ph), 79.3 (2C, OCH_2_CH*C*H), 108.5 (1C, *C*(CH_3_)_2_), 127.3 (2C, C-4′_phenyl_), 127.4 (4C, C-2′_phenyl_, C-6′_phenyl_), 128.2 (4C, C-3′_phenyl_, C-5′_phenyl_), and 138.6 (2C, C-1′_phenyl_); IR (neat): ν̃ [cm^–1^] = 3363, 2985, 2912, 2866, 1496, 1453, 1370, 1239, 1211, 1166, 1066, 1027, 907, 872, 734, 696, 595, 509, and 466; HRMS (*m*/*z*): [M + Na]^+^ calcd for C_23_H_30_NaO_6_, 425.1935; found, 425.1929; HPLC (method 1): *t*_R_ = 22.9 min, purity 96.8%.

#### (4*R*,5*R*)-4,5-Bis{(*R*)-2-(benzyloxy)-1-[(4-iodobenzyl)oxy]ethyl}-2,2-dimethyl-1,3-dioxolane (**24**)

Under a N_2_ atmosphere and ice-cooling, sodium hydride (60% suspension in paraffin oil, 390 mg, 9.7 mmol) was added to a solution of **23** (620 mg, 1.5 mmol) in THF (15 mL). After stirring the mixture for 10 min at 0 °C, stirring was continued for 1 h at ambient temperature. Then, 4-iodobenzyl bromide (1.4 g, 4.6 mmol) was added, and the reaction mixture was stirred for 72 h at ambient temperature. Afterward, methanol and water were added, and the mixture was extracted with ethyl acetate (3×). The combined organic layers were dried (Na_2_SO_4_), filtered, and the solvent was removed in vacuo. The residue was purified by flash column chromatography (Ø = 6 cm, *h* = 17 cm, *V* = 50 mL, petroleum ether/ethyl acetate = 10:1, *R*_*f*_ = 0.27) to give **24** as a colorless oil (1.1 g, 1.3 mmol, 85%). [α]_D_^20^ = +18.2 (10.4, acetonitrile); ^1^H NMR (DMSO-*d*_6_): δ [ppm] = 1.28 (s, 6H, C(C*H*_3_)_2_), 3.51 (dd, *J* = 10.5/5.7 Hz, 2H, OC*H*_2_CHCH), 3.63–3.74 (m, 4H, OCH_2_C*H*CH, OC*H*_2_CHCH (2H)), 4.08–4.14 (m, 2H, OCH_2_CHC*H*), 4.43 (s, 4H, CH_2_OC*H*_2_Ph), 4.48 (d, *J* = 12.1 Hz, 2H, CHOC*H*_2_Ar), 4.58 (d, *J* = 12.1 Hz, 2H, CHOC*H*_2_Ar), 7.04–7.10 (m, 4H, 2″-H_4-iodophenyl_, 6″-H_4-iodophenyl_), 7.24–7.35 (m, 10H, 2′-H_phenyl_, 3′-H_phenyl_, 4′-H_phenyl_, 5′-H_phenyl_, 6′-H_phenyl_), and 7.59–7.65 (m, 4H, 3″-H_4-iodophenyl_, 5″-H_4-iodophenyl_); ^13^C NMR (DMSO-*d*_6_): δ [ppm] = 27.1 (2C, C(*C*H_3_)_2_), 69.6 (2C, O*C*H_2_CHCH), 70.9 (2C, CHO*C*H_2_Ar), 72.3 (2C, CH_2_O*C*H_2_Ph), 77.6 (2C, OCH_2_CH*C*H), 79.0 (2C, OCH_2_*C*HCH), 93.3 (2C, C-4″_4-iodophenyl_), 109.0 (1C, *C*(CH_3_)_2_), 127.38 (4C, C-2′_phenyl_, C-6′_phenyl_), 127.40 (2C, C-4′_phenyl_), 128.2 (4C, C-3′_phenyl_, C-5′_phenyl_), 129.7 (4C, C-2″_4-iodophenyl_, C-6″_4-iodophenyl_), 136.9 (4C, C-3″_4-iodophenyl_, C-5″_4-iodophenyl_), 138.3 (2C, C_arom._), and 138.4 (2C, C_arom._); IR (neat): ν̃ [cm^–1^] = 2861, 1484, 1453, 1368, 1239, 1209, 1073, 1006, 872, 798, 733, 696, 610, and 470; HRMS (*m*/*z*): [M + Na]^+^ calcd for C_37_H_40_I_2_NaO_6_, 857.0806; found, 857.0780; HPLC (method 3): *t*_R_ = 32.1 min, purity 95.6%.

#### (2*R*,3*S*,4*S*,5*R*)-1,6-Bis(benzyloxy)-2,5-bis[(4-iodobenzyl)oxy]hexane-3,4-diol (**25**)

After stirring **24** (870 mg, 1.0 mmol) in 80% aqueous trifluoroacetic acid (5 mL) for 2 h at 0 °C, the reaction mixture was diluted with toluene, and the solvent was removed in vacuo. The residue was purified by flash column chromatography (Ø = 3 cm, *h* = 17 cm, *V* = 20 mL, petroleum ether/ethyl acetate = 2:1, *R*_*f*_ = 0.31) to give **25** as a colorless solid (800 mg, 1.0 mmol, 97%). mp 80 °C; [α]_D_^20^ = +23.4 (5.9, methanol); ^1^H NMR (DMSO-*d*_6_): δ [ppm] = 3.59–3.68 (m, 4H, OCH_2_C*H*CHOH, OC*H*_2_CHCHOH (2H)), 3.71–3.79 (m, 2H, OCH_2_CHC*H*OH), 3.82–3.90 (m, 2H, OC*H*_2_CHCHOH), 4.50 (d, *J* = 12.0 Hz, 2H, CHOC*H*_2_Ar), 4.51 (s, 4H, CH_2_OC*H*_2_Ph), 4.53–4.61 (m, 2H, OCH_2_CHCHO*H*), 4.64 (d, *J* = 12.0 Hz, 2H, CHOC*H*_2_Ar), 7.08–7.15 (m, 4H, 2″-H_4-iodophenyl_, 6″-H_4-iodophenyl_), 7.24–7.36 (m, 10H, 2′-H_phenyl_, 3′-H_phenyl_, 4′-H_phenyl_, 5′-H_phenyl_, 6′-H_phenyl_), and 7.59–7.66 (m, 4H, 3″-H_4-iodophenyl_, 5″-H_4-iodophenyl_); ^13^C NMR (DMSO-*d*_6_): δ [ppm] = 68.1 (2C, OCH_2_CH*C*HOH), 70.6 (2C, O*C*H_2_CHCHOH), 71.0 (2C, CHO*C*H_2_Ar), 72.4 (2C, CH_2_O*C*H_2_Ph), 78.6 (2C, OCH_2_*C*HCHOH), 93.0 (2C, C-4″_4-iodophenyl_), 127.28 (2C, C-4′_phenyl_), 127.31 (4C, C-2′_phenyl_, C-6′_phenyl_), 128.2 (4C, C-3′_phenyl_, C-5′_phenyl_), 129.6 (4C, C-2″_4-iodophenyl_, C-6″_4-iodophenyl_), 136.8 (4C, C-3″_4-iodophenyl_, C-5″_4-iodophenyl_), 138.7 (2C, C-1′_phenyl_), and 138.9 (2C, C-1″_4-iodophenyl_); IR (neat): ν̃ [cm^–1^] = 3420, 3261, 2929, 2866, 1484, 1453, 1401, 1373, 1344, 1318, 1298, 1215, 1100, 1077, 1056, 1006, 875, 794, 748, 732, 693, 476, and 456; HRMS (*m*/*z*): [M + Na]^+^ calcd for C_34_H_36_I_2_NaO_6_, 817.0493; found, 817.0495; HPLC (method 3): *t*_R_ = 29.4 min, purity 96.4%.

#### Methyl (*S*)-3-(benzyloxy)-2-[(4-iodobenzyl)oxy]propanoate ((*S*)-**18**)

An oxidant solution was prepared by dissolving H_5_IO_6_ (11.4 g, 50 mmol) and CrO_3_ (23 mg, 0.23 mmol) in wet acetonitrile (114 mL, 0.75% water V/V) overnight. Under ice-cooling, the oxidant solution (8 mL) was added to a solution of **17** (590 mg, 1.5 mmol) in acetonitrile (10 mL). After stirring the reaction mixture for 30 min at 0 °C, stirring was continued for 16 h at ambient temperature. Then, the solvent was concentrated in vacuo, water was added, and the mixture was extracted with diethyl ether (3×). The combined organic layers were dried (Na_2_SO_4_), filtered, and the solvent was removed in vacuo. The residue was dissolved in methanol (15 mL) and concentrated sulfuric acid (0.1 mL) was added. After heating the reaction mixture to reflux for 16 h, the solvent was concentrated in vacuo. Then, the mixture was diluted with ethyl acetate and washed with ice-cold water, a saturated aqueous solution of NaHCO_3_, and brine. The combined organic layers were dried (Na_2_SO_4_), filtered, and the solvent was removed in vacuo. The residue was purified by flash column chromatography (Ø = 4 cm, *h* = 24 cm, *V* = 30 mL, petroleum ether/ethyl acetate = 6:1, *R*_*f*_ = 0.31) to give (*S*)-**18** as a colorless oil (300 mg, 0.71 mmol, 48%). [α]_D_^20^ = −25.6 (1.3, methanol); HPLC (method 1): *t*_R_ = 26.0 min, purity 94.5%.

#### Methyl (*R*)-3-(benzyloxy)-2-[(4-iodobenzyl)oxy]propanoate ((*R*)-**18**)

NaIO_4_ (320 mg, 1.5 mmol) was added to a solution of **25** (770 mg, 0.97 mmol) in methanol (40 mL), and the mixture was stirred at ambient temperature for 48 h. Then, the solvent was concentrated in vacuo, brine was added, and the mixture was extracted with ethyl acetate (3×). The combined organic layers were dried (Na_2_SO_4_), filtered, and the solvent was removed in vacuo. The residue was dissolved in a mixture of methanol and water (9:1, 40 mL) and NaHCO_3_ (2.5 g, 29 mmol) was added. Then, Br_2_ (0.15 mL, 0.46 g, 2.9 mmol) was added, and the reaction mixture was stirred at ambient temperature for 48 h in a flask protected from ordinary lighting. Afterward, sodium thiosulfate and water were added and the mixture was extracted with ethyl acetate (3×). The combined organic layers were dried (Na_2_SO_4_), filtered, and the solvent was removed in vacuo. The residue was purified by flash column chromatography (Ø = 6 cm, *h* = 19 cm, *V* = 50 mL, petroleum ether/ethyl acetate = 4:1, *R*_*f*_ = 0.51) to give (*R*)-**18** as a colorless oil (570 mg, 1.3 mmol, 69%). [α]_D_^20^ = +29.8 (8.4, methanol); HPLC (method 1): *t*_R_ = 26.1 min, purity 97.8%.

##### Spectroscopic Data of (*S*)-**18** and (*R*)-**18**

^1^H NMR (DMSO-*d*_6_): δ [ppm] = 3.67 (s, 3H, CO_2_C*H*_3_), 3.71 (dd, *J* = 10.7/3.8 Hz, 1H, OCHC*H*_2_O), 3.73 (dd, *J* = 10.7/5.1 Hz, 1H, OCHC*H*_2_O), 4.29 (dd, *J* = 5.0/4.0 Hz, 1H, OC*H*CH_2_O), 4.41 (d, *J* = 12.1 Hz, 1H, CHOC*H*_2_Ar), 4.48 (d, *J* = 12.2 Hz, 1H, CH_2_OC*H*_2_Ph), 4.53 (d, *J* = 12.2 Hz, 1H, CH_2_OC*H*_2_Ph), 4.60 (d, *J* = 12.1 Hz, 1H, CHOC*H*_2_Ar), 7.15–7.18 (m, 2H, 2″-H_4-iodophenyl_, 6″-H_4-iodophenyl_), 7.26–7.32 (m, 3H, 2′-H_phenyl_, 4′-H_phenyl_, 6′-H_phenyl_), 7.32–7.37 (m, 2H, 3′-H_phenyl_, 5′-H_phenyl_), and 7.69–7.73 (m, 2H, 3″-H_4-iodophenyl_, 5″-H_4-iodophenyl_); ^13^C NMR (DMSO-*d*_6_): δ [ppm] = 51.8 (1C, CO_2_*C*H_3_), 70.1 (1C, OCH*C*H_2_O), 70.8 (1C, CHO*C*H_2_Ar), 72.3 (1C, CH_2_O*C*H_2_Ph), 77.7 (1C, O*C*HCH_2_O), 93.6 (1C, C-4″_4-iodophenyl_), 127.47 (2C, C-2′_phenyl_, C-6′_phenyl_), 127.50 (1C, C-4′_phenyl_), 128.2 (2C, C-3′_phenyl_, C-5′_phenyl_), 129.8 (2C, C-2″_4-iodophenyl_, C-6″_4-iodophenyl_), 137.0 (2C, C-3″_4-iodophenyl_, C-5″_4-iodophenyl_), 137.7 (1C, C-1″_4-iodophenyl_), 138.0 (1C, C-1′_phenyl_), and 170.5 (1C, *C*O_2_CH_3_); IR (neat): ν̃ [cm^–1^] = 3070, 2930, 2857, 1472, 1427, 1390, 1361, 1105, 1028, 857, 738, 697, 611, 503, and 487; HRMS (*m*/*z*): [M + H]^+^ calcd for C_18_H_19_INaO_4_, 449.0220; found, 449.0219.

#### Methyl (*S*)-3-(benzyloxy)-2-[(4-{[4-(morpholinomethyl)phenyl]ethynyl}benzyl)oxy]propanoate ((*S*)-**19**)

Under a N_2_ atmosphere, copper(I) iodide (11 mg, 0.058 mmol), bis(triphenylphosphine)palladium(II) chloride (38 mg, 0.054 mmol), and diisopropylamine (3 mL) were added to a solution of (*S*)-**18** (200 mg, 0.48 mmol) in dry THF (10 mL) at ambient temperature, and the mixture was stirred for 20 min. Then, 4-(4-ethynylbenzyl)morpholine (220 mg, 1.1 mmol) was added in two portions at an interval of 30 min. After stirring the reaction mixture for 24 h at ambient temperature, the solvent was removed in vacuo. The residue was dissolved in a mixture of petroleum ether and ethyl acetate (1:1) and filtered through a short silica gel column. The solvent was removed in vacuo, and the residue was purified by flash column chromatography (Ø = 4 cm, *h* = 11 cm, *V* = 20 mL, petroleum ether/ethyl acetate = 1:1, *R*_*f*_ = 0.17) to give (*S*)-**19** as a yellow oil (210 mg, 0.42 mmol, 88%). [α]_D_^20^ = −21.3 (1.2, methanol); HPLC (method 1): *t*_R_ = 21.7 min, purity 93.2%.

#### Methyl (*R*)-3-(benzyloxy)-2-[(4-{[4-(morpholinomethyl)phenyl]ethynyl}benzyl)oxy]propanoate ((*R*)-**19**)

Under a N_2_ atmosphere, copper(I) iodide (52 mg, 0.27 mmol), bis(triphenylphosphine)palladium(II) chloride (140 mg, 0.20 mmol), and diisopropylamine (10 mL) were added to a solution of (*R*)-**18** (430 mg, 1.0 mmol) in dry THF (5 mL) at ambient temperature, and the mixture was stirred for 10 min. Then, 4-(4-ethynylbenzyl)morpholine (300 mg, 1.5 mmol) was added, and the reaction mixture was heated to reflux for 24 h. After stirring the reaction mixture for an additional 12 h at ambient temperature, the solvent was removed in vacuo. The residue was dissolved in a mixture of petroleum ether and ethyl acetate (1:1) and filtered through a short silica gel column. The solvent was removed in vacuo, and the residue was purified by flash column chromatography (Ø = 4 cm, *h* = 10 cm, *V* = 30 mL, petroleum ether/ethyl acetate = 1:1, *R*_*f*_ = 0.17) to give (*R*)-**19** as a yellow oil (320 mg, 0.65 mmol, 64%). [α]_D_^20^ = +23.9 (8.0, methanol); HPLC (method 1): *t*_R_ = 21.8 min, purity 95.5%.

##### Spectroscopic Data of (*S*)-**19** and (*R*)-**19**

^1^H NMR (DMSO-*d*_6_): δ [ppm] = 2.31–2.40 (m, 4H, N(C*H*_2_CH_2_)_2_O), 3.49 (s, 2H, NC*H*_2_Ar), 3.55–3.60 (m, 4H, N(CH_2_C*H*_2_)_2_O), 3.68 (s, 3H, CO_2_C*H*_3_), 3.73 (dd, *J* = 10.7/4.0 Hz, 1H, OCHC*H*_2_O), 3.75 (dd, *J* = 10.7/5.1 Hz, 1H, OCHC*H*_2_O), 4.32 (dd, *J* = 5.0/4.1 Hz, 1H, OC*H*CH_2_O), 4.49 (d, *J* = 12.2 Hz, 1H, CH_2_OC*H*_2_Ph), 4.52–4.56 (m, 2H, CHOC*H*_2_Ar (1H), CH_2_OC*H*_2_Ph (1H)), 4.68 (d, *J* = 12.2 Hz, 1H, CHOC*H*_2_Ar), 7.27–7.31 (m, 3H, 2′-H_phenyl_, 4′-H_phenyl_, 6′-H_phenyl_), 7.33–7.38 (m, 4H, 3′-H_phenyl_, 5′-H_phenyl_, 3‴-H_4-(morpholinomethyl)phenyl_, 5‴-H_4-(morpholinomethyl)phenyl_), 7.38–7.42 (m, 2H, 2″-H_4-{[4-(morpholinomethyl)phenyl]ethynyl}phenyl_, 6″-H_4-{[4-(morpholinomethyl)phenyl]ethynyl}phenyl_), and 7.49–7.54 (m, 4H, 3″-H_4-{[4-(morpholinomethyl)phenyl]ethynyl}phenyl_, 5″-H_4-{[4-(morpholinomethyl)phenyl]ethynyl}phenyl_, 2‴-H_4-(morpholinomethyl)phenyl_, 6‴-H_4-(morpholinomethyl)phenyl_); ^13^C NMR (DMSO-*d*_6_): δ [ppm] = 51.8 (1C, CO_2_*C*H_3_), 53.2 (2C, N(*C*H_2_CH_2_)_2_O), 62.0 (1C, Ar*C*H_2_N), 66.2 (2C, N(CH_2_*C*H_2_)_2_O), 70.1 (1C, OCH*C*H_2_O), 71.1 (1C, CHO*C*H_2_Ar), 72.3 (1C, CH_2_O*C*H_2_Ph), 77.8 (1C, O*C*HCH_2_O), 89.0 (1C, C≡C), 89.3 (1C, C≡C), 120.8 (1C, C-1‴_4-(morpholinomethyl)phenyl_), 121.4 (1C, C-4″_4-{[4-(morpholinomethyl)phenyl]ethynyl}phenyl_), 127.48 (2C, C-2′_phenyl_, C-6′_phenyl_), 127.51 (1C, C-4′_phenyl_), 127.8 (2C, C-2″_4-{[4-(morpholinomethyl)phenyl]ethynyl}phenyl_, C-6″_4-{[4-(morpholinomethyl)phenyl]ethynyl}phenyl_), 128.3 (2C, C-3′_phenyl_, C-5′_phenyl_), 129.2 (2C, C-3‴_4-(morpholinomethyl)phenyl_, C-5‴_4-(morpholinomethyl)phenyl_), 131.2 (4C, C-3″_4-{[4-(morpholinomethyl)phenyl]ethynyl}phenyl_, C-5″_4-{[4-(morpholinomethyl)phenyl]ethynyl}phenyl_, C-2‴_4-(morpholinomethyl)phenyl_, C-6‴_4-(morpholinomethyl)phenyl_), 138.0 (1C, C-1′_phenyl_), 138.6 (1C, C-1″_4-{[4-(morpholinomethyl)phenyl]ethynyl}phenyl_), 138.9 (1C, C-4‴_4-(morpholinomethyl)phenyl_), and 170.5 (1C, *C*O_2_CH_3_); IR (neat): ν̃ [cm^–1^] = 3030, 2951, 2855, 2807, 1749, 1517, 1453, 1349, 1290, 1204, 1114, 1069, 1007, 913, 865, 819, 794, 736, 697, 540, and 515; HRMS (*m*/*z*): [M + H]^+^ calcd for C_31_H_34_NO_5_, 500.2431; found, 500.2449.

#### (*S*)-3-(Benzyloxy)-*N*-hydroxy-2-[(4-{[4-(morpholinomethyl)phenyl]ethynyl}benzyl)oxy]propanamide ((*S*)-**11a**)

Under ice-cooling, an aqueous solution of hydroxylamine (50 wt %, 2.5 mL) was added to a solution of (*S*)-**19** (120 mg, 0.24 mmol) in a mixture of THF (4 mL) and isopropanol (4 mL). After stirring the reaction mixture for 5 min at 0 °C, stirring was continued for 36 h at ambient temperature. Then, the solvent was removed in vacuo, and the residue was purified by automatic flash column chromatography using a Biotage Isolera One system (10% → 80% ACN in H_2_O, Biotage SNAP Ultra C18 30 g). Fractions containing the desired product were combined and subjected to lyophilization to give (*S*)-**11a** as a colorless solid (80 mg, 0.16 mmol, 67%). mp 65 °C; [α]_D_^20^ = −22.5 (1.5, methanol); HPLC (method 2): *t*_R_ = 13.7 min, purity 100%.

#### (*R*)-3-(Benzyloxy)-*N*-hydroxy-2-[(4-{[4-(morpholinomethyl)phenyl]ethynyl}benzyl)oxy]propanamide ((*R*)-**11a**)

Under ice-cooling, an aqueous solution of hydroxylamine (50 wt %, 6 mL) was added to a solution of (*R*)-**19** (240 mg, 0.48 mmol) in a mixture of THF (5 mL) and isopropanol (5 mL). After stirring the reaction mixture for 5 min at 0 °C, stirring was continued for 72 h at ambient temperature. Then, the solvent was removed in vacuo, water was added, and the mixture was extracted with ethyl acetate (3×). The combined organic layers were dried (Na_2_SO_4_), filtered, and the solvent was removed in vacuo. The residue was purified by automatic flash column chromatography using a Biotage Isolera One system (10% → 100% ACN in H_2_O, Biotage SNAP Ultra C18 12 g). Fractions containing the desired product were combined and subjected to lyophilization to give (*R*)-**11a** as a colorless solid (160 mg, 0.32 mmol, 65%). mp 65 °C; [α]_D_^20^ = +22.6 (1.9, methanol); HPLC (method 2): *t*_R_ = 13.7 min, purity 97.1%.

##### Spectroscopic Data of (*S*)-**11a** and (*R*)-**11a**

^1^H NMR (DMSO-*d*_6_): δ [ppm] = 2.30–2.40 (m, 4H, N(C*H*_2_CH_2_)_2_O), 3.49 (s, 2H, NC*H*_2_Ar), 3.55–3.61 (m, 4H, N(CH_2_C*H*_2_)_2_O), 3.65 (d, *J* = 5.3 Hz, 2H, OCHC*H*_2_O), 4.01 (t, *J* = 5.3 Hz, 1H, OC*H*CH_2_O), 4.46–4.53 (m, 3H, CHOC*H*_2_Ar (1H), CH_2_OC*H*_2_Ph), 4.62 (d, *J* = 12.6 Hz, 1H, CHOC*H*_2_Ar), 7.27–7.33 (m, 3H, 2′-H_phenyl_, 4′-H_phenyl_, 6′-H_phenyl_), 7.33–7.38 (m, 4H, 3′-H_phenyl_, 5′-H_phenyl_, 3‴-H_4-(morpholinomethyl)phenyl_, 5‴-H_4-(morpholinomethyl)phenyl_), 7.39–7.43 (m, 2H, 2″-H_4-{[4-(morpholinomethyl)phenyl]ethynyl}phenyl_, 6″-H_4-{[4-(morpholinomethyl)phenyl]ethynyl}phenyl_), 7.49–7.54 (m, 4H, 3″-H_4-{[4-(morpholinomethyl)phenyl]ethynyl}phenyl_, 5″-H_4-{[4-(morpholinomethyl)phenyl]ethynyl}phenyl_, 2‴-H_4-(morpholinomethyl)phenyl_, 6‴-H_4-(morpholinomethyl)phenyl_), 8.96 (s br, 1H, CON*H*OH), and 10.77 (s br, 1H, CONHO*H*); ^13^C NMR (DMSO-*d*_6_): δ [ppm] = 53.2 (2C, N(*C*H_2_CH_2_)_2_O), 62.0 (1C, Ar*C*H_2_N), 66.2 (2C, N(CH_2_*C*H_2_)_2_O), 70.3 (1C, OCH*C*H_2_O), 70.7 (1C, CHO*C*H_2_Ar), 72.2 (1C, CH_2_O*C*H_2_Ph), 77.7 (1C, O*C*HCH_2_O), 89.1 (1C, C≡C), 89.3 (1C, C≡C), 120.8 (1C, C-1‴_4-(morpholinomethyl)phenyl_), 121.4 (1C, C-4″_4-{[4-(morpholinomethyl)phenyl]ethynyl}phenyl_), 127.45 (1C, C-4′_phenyl_), 127.50 (2C, C-2′_phenyl_, C-6′_phenyl_), 127.8 (2C, C-2″_4-{[4-(morpholinomethyl)phenyl]ethynyl}phenyl_, C-6″_4-{[4-(morpholinomethyl)phenyl]ethynyl}phenyl_), 128.2 (2C, C-3′_phenyl_, C-5′_phenyl_), 129.2 (2C, C-3‴_4-(morpholinomethyl)phenyl_, C-5‴_4-(morpholinomethyl)phenyl_), 131.17 (2C, C_arom._), 131.23 (2C, C_arom._), 138.1 (1C, C-1′_phenyl_), 138.7 (1C, C-1″_4-{[4-(morpholinomethyl)phenyl]ethynyl}phenyl_), 138.8 (1C, C-4‴_4-(morpholinomethyl)phenyl_), and 165.7 (1C, *C*ONHOH); IR (neat): ν̃ [cm^–1^] = 3209, 3030, 2857, 2810, 1664, 1517, 1453, 1350, 1113, 1006, 914, 864, 820, 792, 736, 697, 539, and 514; HRMS (*m*/*z*): [M + H]^+^ calcd for C_30_H_33_N_2_O_5_, 501.2384; found, 501.2395.

#### (*S*)-1-Azido-3-[(*tert*-butyldiphenylsilyl)oxy]propan-2-ol (**26**)

Sodium azide (2.5 g, 38 mmol) was added to a solution of **15** (2.4 g, 7.6 mmol) and ammonium chloride (890 mg, 17 mmol) in a mixture of methanol (120 mL) and water (15 mL). After stirring the reaction mixture at 65 °C for 16 h, the mixture was diluted with diethyl ether (120 mL), a saturated aqueous solution of NaHCO_3_ (100 mL) was added, and the mixture was extracted with diethyl ether (3×). The combined organic layers were washed with brine, dried (Na_2_SO_4_), filtered, and the solvent was removed in vacuo. The residue was purified by automatic flash column chromatography using an Interchim puriFlash XS 420 system (0% → 10% ethyl acetate in petroleum ether, Biotage SNAP Ultra HP-Sphere 50 g) to give **26** as a colorless oil (1.9 g, 5.3 mmol, 70%). *R*_*f*_ = 0.36 (petroleum ether/ethyl acetate = 10:1); [α]_D_^20^ = −15.6 (2.3, methanol); ^1^H NMR (DMSO-*d*_6_): δ [ppm] = 0.99 (s, 9H, SiC(C*H*_3_)_3_), 3.32 (dd, *J* = 12.6/6.3 Hz, 1H, N_3_C*H*_2_CHCH_2_), 3.41 (dd, *J* = 12.6/3.6 Hz, 1H, N_3_C*H*_2_CHCH_2_), 3.55 (dd, *J* = 10.0/7.0 Hz, 1H, N_3_CH_2_CHC*H*_2_), 3.60 (dd, *J* = 10.0/5.0 Hz, 1H, N_3_CH_2_CHC*H*_2_), 3.73–3.85 (m, 1H, N_3_CH_2_C*H*CH_2_), 5.27 (d, *J* = 5.2 Hz, 1H, CHO*H*), 7.39–7.51 (m, 6H, 3′-H_diphenylsilyl_, 4′-H_diphenylsilyl_, 5′-H_diphenylsilyl_), and 7.58–7.67 (m, 4H, 2′-H_diphenylsilyl_, 6′-H_diphenylsilyl_);

^13^C NMR (DMSO-*d*_6_): δ [ppm] = 18.8 (1C, Si*C*(CH_3_)_3_), 26.6 (3C, Si*C*(CH_3_)_3_), 53.2 (1C, N_3_*C*H_2_CHCH_2_), 65.1 (1C, N_3_CH_2_CH*C*H_2_), 70.2 (1C, N_3_CH_2_*C*HCH_2_), 127.9 (4C, C-3′_diphenylsilyl_, C-5′_diphenylsilyl_), 129.9 (2C, C-4′_diphenylsilyl_), 132.8 (1C, C-1′_diphenylsilyl_), 132.9 (1C, C-1′_diphenylsilyl_), 135.0 (2C, C-2′_diphenylsilyl_, C-6′_diphenylsilyl_), and 135.1 (2C, C-2′_diphenylsilyl_, C-6′_diphenylsilyl_); IR (neat): ν̃ [cm^–1^] = 3430, 3071, 2930, 2858, 2098, 1472, 1427, 1288, 1106, 998, 937, 823, 800, 740, 699, 613, 503, and 486; HRMS (*m*/*z*): [M + Na]^+^ calcd for C_19_H_25_N_3_NaO_2_Si, 378.1608; found, 378.1574; HPLC (method 1): *t*_R_ = 27.6 min, purity 99.8%.

#### (*S*)-3-Azido-2-[(4-iodobenzyl)oxy]propan-1-ol (**27**)

Under a N_2_ atmosphere, sodium hydride (60% suspension in paraffin oil, 970 mg, 24 mmol) was added to an ice-cooled solution of **26** (1.8 g, 5.1 mmol) in anhydrous THF (75 mL). After stirring the reaction mixture for 15 min at 0 °C, 4-iodobenzyl bromide (1.9 g, 6.5 mmol) was added, and the reaction mixture was stirred for additional 15 min at 0 °C. Then, the ice-bath was removed, and the mixture was stirred for 72 h at ambient temperature. Afterward, methanol was added under ice-cooling, the solvent was removed in vacuo, water was added, and the mixture was extracted with ethyl acetate (3×). The combined organic layers were dried (Na_2_SO_4_), filtered, and the solvent was removed in vacuo. The residue was dissolved in THF (80 mL), tetrabutylammonium fluoride trihydrate (3.5 g, 11 mmol) was added, and the reaction mixture was stirred for 36 h at ambient temperature. Then, the solvent was removed in vacuo, diethyl ether was added, and the mixture was washed with a saturated aqueous solution of ammonium chloride, water, and brine. The organic layer was dried (Na_2_SO_4_), filtered, and the solvent was removed in vacuo. The residue was purified by automatic flash column chromatography using an Interchim puriFlash XS 420 system (20% → 33% ethyl acetate in petroleum ether, Biotage SNAP Ultra HP-Sphere 50 g) to give **27** as a yellow oil (1.3 g, 3.9 mmol, 78%). *R*_*f*_ = 0.38 (petroleum ether/ethyl acetate = 2:1); [α]_D_^20^ = +7.8 (2.6, methanol); ^1^H NMR (DMSO-*d*_6_): δ [ppm] = 3.33 (dd, *J* = 13.1/6.1 Hz, 1H, N_3_C*H*_2_CHCH_2_OH), 3.41–3.61 (m, 4H, N_3_C*H*_2_CHCH_2_OH (1H), N_3_CH_2_C*H*CH_2_OH, N_3_CH_2_CHC*H*_2_OH), 4.55 (d, *J* = 12.2 Hz, 1H, OC*H*_2_Ar), 4.61 (d, *J* = 12.2 Hz, 1H, OC*H*_2_Ar), 4.81 (t, *J* = 5.5 Hz, 1H, CH_2_O*H*), 7.14–7.21 (m, 2H, 2′-H_4-iodophenyl_, 6′-H_4-iodophenyl_), and 7.67–7.74 (m, 2H, 3′-H_4-iodophenyl_, 5′-H_4-iodophenyl_); ^13^C NMR (DMSO-*d*_6_): δ [ppm] = 50.9 (1C, N_3_*C*H_2_CHCH_2_OH), 60.3 (1C, N_3_CH_2_CH*C*H_2_OH), 70.1 (1C, O*C*H_2_Ar), 78.9 (1C, N_3_CH_2_*C*HCH_2_OH), 93.3 (1C, C-4′_4-iodophenyl_), 129.7 (2C, C-2′_4-iodophenyl_, C-6′_4-iodophenyl_), 136.9 (2C, C-3′_4-iodophenyl_, C-5′_4-iodophenyl_), and 138.4 (1C, C-1′_4-iodophenyl_); IR (neat): ν̃ [cm^–1^] = 3399, 2927, 2871, 2093, 1590, 1484, 1402, 1344, 1273, 1099, 1057, 1006, 798, 627, 554, and 470; HRMS (*m*/*z*): [M + Na]^+^ calcd for C_10_H_12_IN_3_NaO_2_, 355.9866; found, 355.9875; HPLC (method 1): *t*_R_ = 21.7 min, purity 99.9%.

#### (2*R*,2′*R*)-[(4*R*,5*R*)-2,2-Dimethyl-1,3-dioxolane-4,5-diyl]bis(2-hydroxyethane-2,1-diyl) bis(4-methylbenzenesulfonate) (**29**)

A mixture of **22** (1.2 g, 5.3 mmol) and dibutyltin oxide (2.8 g, 11 mmol) was heated to reflux in toluene (250 mL) using a Dean–Stark trap for 48 h. Then, the solvent was removed in vacuo and the residue was suspended in chloroform (50 mL). Under ice-cooling, *p*-toluenesulfonyl chloride (2.1 g, 11 mmol) was added, and the reaction mixture was stirred for 5 min at 0 °C and subsequently for 7 d at ambient temperature. Then, the solvent was removed in vacuo and the residue was purified by flash column chromatography (Ø = 6 cm, *h* = 21 cm, *V* = 50 mL, petroleum ether/ethyl acetate = 1:1, *R*_*f*_ = 0.49) to give **29** as a colorless oil (2.6 g, 5.0 mmol, 94%). [α]_D_^20^ = +26.0 (2.9, methanol); ^1^H NMR (DMSO-*d*_6_): δ [ppm] = 1.14 (s, 6H, C(C*H*_3_)_2_), 2.42 (s, 6H, ArC*H*_3_), 3.62–3.68 (m, 2H, OCH_2_C*H*CH), 3.75–3.79 (m, 2H, OCH_2_CHC*H*), 3.89 (dd, *J* = 10.3/6.6 Hz, 2H, OC*H*_2_CHCH), 4.05 (dd, *J* = 10.3/2.5 Hz, 2H, OC*H*_2_CHCH), 5.58 (d, *J* = 5.5 Hz, 2H, CHO*H*), 7.45–7.49 (m, 4H, 3′-H_4-methylbenzenesulfonate_, 5′-H_4-methylbenzenesulfonate_), and 7.75–7.79 (m, 4H, 2′-H_4-methylbenzenesulfonate_, 6′-H_4-methylbenzenesulfonate_); ^13^C NMR (DMSO-*d*_6_): δ [ppm] = 21.1 (2C, Ar*C*H_3_), 27.2 (2C, C(*C*H_3_)_2_), 69.3 (2C, OCH_2_*C*HCH), 72.0 (2C, O*C*H_2_CHCH), 78.3 (2C, OCH_2_CH*C*H), 109.3 (1C, *C*(CH_3_)_2_), 127.7 (4C, C-2′_4-methylbenzenesulfonate_, C-6′_4-methylbenzenesulfonate_), 130.1 (4C, C-3′_4-methylbenzenesulfonate_, C-5′_4-methylbenzenesulfonate_), 132.2 (2C, C-1′_4-methylbenzenesulfonate_), and 144.9 (2C, C-4′_4-methylbenzenesulfonate_); IR (neat): ν̃ [cm^–1^] = 3391, 2987, 1735, 1598, 1453, 1356, 1242, 1172, 1075, 974, 937, 902, 812, 664, and 551; HRMS (*m*/*z*): [M + H]^+^ calcd for C_23_H_31_O_10_S_2_, 531.1353; found, 531.1348; HPLC (method 1): *t*_R_ = 23.6 min, purity 99.7%.

#### (1*R*,1′*R*)-1,1′-[(4*R*,5*R*)-2,2-Dimethyl-1,3-dioxolane-4,5-diyl]bis(2-azidoethan-1-ol) (**30**)

Sodium azide (2.2 g, 34 mmol) was added to a solution of **29** (5.7 g, 11 mmol) in DMSO (150 mL), and the reaction mixture was heated to 80 °C for 24 h. Under ice-cooling, water was added and the mixture was extracted with ethyl acetate (3×). The combined organic layers were dried (Na_2_SO_4_), filtered, and the solvent was removed in vacuo. The residue was purified by flash column chromatography (Ø = 6 cm, *h* = 24 cm, *V* = 50 mL, petroleum ether/ethyl acetate = 1:1, *R*_*f*_ = 0.78) to give **30** as a colorless oil (2.6 g, 9.5 mmol, 89%). [α]_D_^20^ = +39.9 (6.3, methanol); ^1^H NMR (DMSO-*d*_6_): δ [ppm] = 1.29 (s, 6H, C(C*H*_3_)_2_), 3.28 (dd, *J* = 12.8/6.7 Hz, 2H, N_3_C*H*_2_CHCH), 3.35 (dd, *J* = 12.8/3.0 Hz, 2H, N_3_C*H*_2_CHCH), 3.66–3.74 (m, 2H, N_3_CH_2_C*H*CH), 3.85–3.91 (m, 2H, N_3_CH_2_CHC*H*), and 5.64 (d, *J* = 5.3 Hz, 2H, CHO*H*); ^13^C NMR (DMSO-*d*_6_): δ [ppm] = 27.3 (2C, C(*C*H_3_)_2_), 53.4 (2C, N_3_*C*H_2_CHCH), 71.2 (2C, N_3_CH_2_*C*HCH), 79.5 (2C, N_3_CH_2_CH*C*H), and 109.1 (1C, *C*(CH_3_)_2_); IR (neat): ν̃ [cm^–1^] = 3354, 2989, 2934, 2096, 1441, 1373, 1214, 1164, 1069, 870, 656, 555, and 504; HRMS (*m*/*z*): [M + Na]^+^ calcd for C_9_H_16_N_6_NaO_4_, 295.1125; found, 295.1118.

#### (4*R*,5*R*)-4,5-bis{(*R*)-2-Azido-1-[(4-iodobenzyl)oxy]ethyl}-2,2-dimethyl-1,3-dioxolane (**31**)

Under a N_2_ atmosphere and ice-cooling, sodium hydride (60% suspension in paraffin oil, 210 mg, 5.3 mmol) was added to a solution of **30** (270 mg, 0.98 mmol) in THF (15 mL). After stirring the mixture for 10 min at 0 °C, stirring was continued for 1 h at ambient temperature. Then, 4-iodobenzyl bromide (870 mg, 2.9 mmol) was added, and the reaction mixture was stirred for 72 h at ambient temperature. Afterward, methanol and water were added, and the mixture was extracted with ethyl acetate (3×). The combined organic layers were dried (Na_2_SO_4_), filtered, and the solvent was removed in vacuo. The residue was purified by flash column chromatography (Ø = 4 cm, *h* = 12 cm, *V* = 30 mL, petroleum ether/ethyl acetate = 4:1, *R*_*f*_ = 0.49) to give **31** as a colorless oil (570 mg, 0.81 mmol, 82%). [α]_D_^20^ = +17.7 (6.8, methanol); ^1^H NMR (DMSO-*d*_6_): δ [ppm] = 1.31 (s, 6H, C(C*H*_3_)_2_), 3.29–3.35 (m, 2H, N_3_C*H*_2_CHCH), 3.66–3.73 (m, 4H, N_3_C*H*_2_CHCH (2H), N_3_CH_2_C*H*CH), 4.09–4.13 (m, 2H, N_3_CH_2_CHC*H*), 4.53 (d, *J* = 11.8 Hz, 2H, OC*H*_2_Ar), 4.58 (d, *J* = 11.8 Hz, 2H, OC*H*_2_Ar), 7.08–7.13 (m, 4H, 2′-H_4-iodophenyl_, 6′-H_4-iodophenyl_), and 7.63–7.67 (m, 4H, 3′-H_4-iodophenyl_, 5′-H_4-iodophenyl_); ^13^C NMR (DMSO-*d*_6_): δ [ppm] = 27.1 (2C, C(*C*H_3_)_2_), 49.7 (2C, N_3_*C*H_2_CHCH), 70.5 (2C, O*C*H_2_Ar), 77.4 (2C, N_3_CH_2_CH*C*H), 78.9 (2C, N_3_CH_2_*C*HCH), 93.5 (2C, C-4′_4-iodophenyl_), 109.5 (1C, *C*(CH_3_)_2_), 129.8 (4C, C-2′_4-iodophenyl_, C-6′_4-iodophenyl_), 137.0 (4C, C-3′_4-iodophenyl_, C-5′_4-iodophenyl_), and 137.7 (2C, C-1′_4-iodophenyl_); IR (neat): ν̃ [cm^–1^] = 2986, 2933, 2869, 2095, 1590, 1484, 1371, 1238, 1210, 1081, 1006, 867, 828, 797, 656, 629, and 471; HRMS (*m*/*z*): [M + Na]^+^ calcd for C_23_H_26_I_2_N_6_NaO_4_, 726.9997; found, 726.9993; HPLC (method 1): *t*_R_ = 30.1 min, purity 97.0%.

#### (2*R*,3*S*,4*S*,5*R*)-1,6-Diazido-2,5-bis[(4-iodobenzyl)oxy]hexane-3,4-diol (**32**)

After stirring **31** (4.4 g, 6.3 mmol) in 80% aqueous acetic acid (40 mL) for 5 h at 0 °C, the reaction mixture was diluted with toluene and the solvent was removed in vacuo. The residue was purified by flash column chromatography (Ø = 6 cm, *h* = 12 cm, *V* = 50 mL, petroleum ether/ethyl acetate = 1:1, *R*_*f*_ = 0.51) to give **32** as a colorless oil (3.8 g, 5.6 mmol, 90%). [α]_D_^20^ = +50.5 (6.5, methanol); ^1^H NMR (DMSO-*d*_6_): δ [ppm] = 3.40 (dd, *J* = 13.1/4.3 Hz, 2H, N_3_C*H*_2_CHCH), 3.61–3.80 (m, 6H, N_3_C*H*_2_CHCH (2H), N_3_CH_2_C*H*CH, N_3_CH_2_CHC*H*), 4.51 (d, *J* = 11.7 Hz, 2H, OC*H*_2_Ar), 4.62 (d, *J* = 11.7 Hz, 2H, OC*H*_2_Ar), 4.85 (d, *J* = 7.9 Hz, 2H, CHO*H*), 7.12–7.19 (m, 4H, 2′-H_4-iodophenyl_, 6′-H_4-iodophenyl_), and 7.67–7.74 (m, 4H, 3′-H_4-iodophenyl_, 5′-H_4-iodophenyl_); ^13^C NMR (DMSO-*d*_6_): δ [ppm] = 50.5 (2C, N_3_*C*H_2_CHCH), 68.2 (2C, N_3_CH_2_CH*C*H), 70.7 (2C, O*C*H_2_Ar), 78.2 (2C, N_3_CH_2_*C*HCH), 93.4 (2C, C-4′_4-iodophenyl_), 129.8 (4C, C-2′_4-iodophenyl_, C-6′_4-iodophenyl_), 137.0 (4C, C-3′_4-iodophenyl_, C-5′_4-iodophenyl_), and 138.2 (2C, C-1′_4-iodophenyl_); IR (neat): ν̃ [cm^–1^] = 3451, 2929, 2873, 2095, 1588, 1483, 1433, 1403, 1365, 1330, 1292, 1260, 1081, 1038, 1004, 885, 854, 829, 793, 745, 618, 595, 525, and 469; HRMS (*m*/*z*): [M + Na]^+^ calcd for C_20_H_22_I_2_N_6_NaO_4_, 686.9684; found, 686.9641; HPLC (method 1): *t*_R_ = 26.7 min, purity 96.7%.

#### Methyl (*S*)-3-azido-2-[(4-iodobenzyl)oxy]propanoate ((*S*)-**28**)

An oxidant solution was prepared by dissolving H_5_IO_6_ (11.4 g, 50 mmol) and CrO_3_ (23 mg, 0.23 mmol) in wet acetonitrile (114 mL, 0.75% water V/V) overnight. Under ice-cooling, the oxidant solution (21 mL) was added to a solution of **27** (1.2 g, 3.6 mmol) in acetonitrile (20 mL). After stirring the reaction mixture for 30 min at 0 °C, stirring was continued for 16 h at ambient temperature. Then, Na_2_HPO_4_ (1.1 g, 7.6 mmol) and water (20 mL) were added under ice-cooling. Afterward, the mixture was diluted with diethyl ether and stirred for 15 min at ambient temperature. Then, 1.0 M HCl (8 mL) was added and the mixture was extracted with diethyl ether (3×). The combined organic layers were dried (Na_2_SO_4_), filtered, and the solvent was removed in vacuo. The residue was dissolved in methanol (60 mL) and concentrated sulfuric acid (0.2 mL) was added. After heating the reaction mixture to reflux for 24 h, the solvent was concentrated in vacuo. Then, the mixture was diluted with dichloromethane and washed with ice-cold water, a saturated aqueous solution of NaHCO_3_, and brine. The combined organic layers were dried (Na_2_SO_4_), filtered, and the solvent was removed in vacuo. The residue was purified by automatic flash column chromatography using an Interchim puriFlash XS 420 system (20% → 33% ethyl acetate in petroleum ether, Biotage SNAP Ultra HP-Sphere 50 g) to give (*S*)-**28** as a colorless oil (850 mg, 2.4 mmol, 65%). *R*_*f*_ = 0.47 (petroleum ether/ethyl acetate = 4:1); [α]_D_^20^ = −47.2 (7.1, methanol); enantiomeric ratio (HPLC method 4): *t*_R_ = 11.2 min, (*S*)/(*R*) = 99.6/0.4; HPLC (method 1): *t*_R_ = 24.3 min, purity 98.9%.

#### Methyl (*R*)-3-azido-2-[(4-iodobenzyl)oxy]propanoate ((*R*)-**28**)

NaIO_4_ (130 mg, 0.62 mmol) was added to a solution of **32** (250 mg, 0.37 mmol) in methanol (15 mL), and the mixture was stirred at ambient temperature for 16 h. Then, the solvent was concentrated in vacuo, brine was added, and the mixture was extracted with ethyl acetate (3×). The combined organic layers were dried (Na_2_SO_4_), filtered, and the solvent was removed in vacuo. The residue was dissolved in a mixture of methanol and water (9:1, 40 mL) and NaHCO_3_ (960 mg, 11 mmol) was added. Then, Br_2_ (0.06 mL, 190 mg, 1.2 mmol) was added, and the reaction mixture was stirred at ambient temperature for 16 h in a flask protected from ordinary lighting. Afterward, sodium thiosulfate and water were added and the mixture was extracted with ethyl acetate (3×). The combined organic layers were dried (Na_2_SO_4_), filtered, and the solvent was removed in vacuo. The residue was purified by flash column chromatography (Ø = 3 cm, *h* = 20 cm, *V* = 20 mL, petroleum ether/ethyl acetate = 4:1, *R*_*f*_ = 0.47) to give (*R*)-**28** as a colorless oil (160 mg, 0.45 mmol, 62%). [α]_D_^20^ = +46.9 (8.2, methanol); enantiomeric ratio (HPLC method 4): *t*_R_ = 10.2 min, (*R*)/(*S*) = 100/0; HPLC (method 1): *t*_R_ = 24.4 min, purity 95.1%.

##### Spectroscopic Data of (*S*)-**28** and (*R*)-**28**

^1^H NMR (DMSO-*d*_6_): δ [ppm] = 3.53 (dd, *J* = 13.3/5.9 Hz, 1H, CHC*H*_2_N_3_), 3.66 (dd, *J* = 13.3/3.5 Hz, 1H, CHC*H*_2_N_3_), 3.70 (s, 3H, CO_2_C*H*_3_), 4.36 (dd, *J* = 5.9/3.5 Hz, 1H, C*H*CH_2_N_3_), 4.52 (d, *J* = 12.0 Hz, 1H, OC*H*_2_Ar), 4.66 (d, *J* = 12.0 Hz, 1H, OC*H*_2_Ar), 7.15–7.22 (m, 2H, 2′-H_4-iodophenyl_, 6′-H_4-iodophenyl_), and 7.70–7.76 (m, 2H, 3′-H_4-iodophenyl_, 5′-H_4-iodophenyl_); ^13^C NMR (DMSO-*d*_6_): δ [ppm] = 51.7 (1C, CH*C*H_2_N_3_), 52.1 (1C, CO_2_*C*H_3_), 71.0 (1C, O*C*H_2_Ar), 77.1 (1C, *C*HCH_2_N_3_), 93.7 (1C, C-4′_4-iodophenyl_), 129.8 (2C, C-2′_4-iodophenyl_, C-6′_4-iodophenyl_), 137.0 (2C, C-3′_4-iodophenyl_, C-5′_4-iodophenyl_), 137.4 (1C, C-1′_4-iodophenyl_), and 170.0 (1C, *C*O_2_CH_3_); IR (neat): ν̃ [cm^–1^] = 2952, 2871, 2098, 1748, 1590, 1484, 1436, 1264, 1203, 1122, 1006, 798, 649, 556, and 474; HRMS (*m*/*z*): [M + Na]^+^ calcd for C_11_H_12_IN_3_NaO_3_, 383.9816; found, 383.9819.

#### Methyl (*S*)-3-(benzylamino)-2-[(4-iodobenzyl)oxy]propanoate ((*S*)-**33**)

Under ice-cooling, triethyl phosphite (0.22 mL, 220 mg, 1.3 mmol) was added to a solution of (*S*)-**28** (440 mg, 1.2 mmol) in toluene (2 mL). After stirring the mixture at ambient temperature overnight, freshly distilled benzaldehyde (0.15 mL, 160 mg, 1.5 mmol) was added slowly under ice-cooling. After stirring the reaction mixture at ambient temperature for 72 h, the solvent was removed in high vacuum. The residue was taken up in methanol (2 mL). Under ice-cooling, NaBH_4_ (59 mg, 1.6 mmol) was added, and the mixture was stirred at ambient temperature overnight. Then, water was added and the mixture was extracted with ethyl acetate (3×). The combined organic layers were dried (Na_2_SO_4_), filtered, and the solvent was removed in vacuo. The residue was purified by flash column chromatography (Ø = 4 cm, *h* = 26 cm, *V* = 30 mL, ethyl acetate, *R*_*f*_ = 0.60) to give (*S*)-**33** as a colorless oil (350 mg, 0.82 mmol, 67%). [α]_D_^20^ = −45.7 (2.5, methanol); HPLC (method 1): *t*_R_ = 20.4 min, purity 99.3%.

#### Methyl (*R*)-3-(benzylamino)-2-[(4-iodobenzyl)oxy]propanoate ((*R*)-**33**)

Under ice-cooling, triethyl phosphite (0.13 mL, 130 mg, 0.76 mmol) was added to a solution of (*R*)-**28** (270 mg, 0.75 mmol) in toluene (1 mL). After stirring the mixture at ambient temperature overnight, freshly distilled benzaldehyde (0.1 mL, 100 mg, 0.98 mmol) was added slowly under ice-cooling. After stirring the reaction mixture at ambient temperature for 72 h, the solvent was removed in high vacuum. The residue was taken up in methanol (1 mL). Under ice-cooling, NaBH_4_ (38 mg, 1.0 mmol) was added, and the mixture was stirred at ambient temperature overnight. Then, water was added and the mixture was extracted with ethyl acetate (3×). The combined organic layers were dried (Na_2_SO_4_), filtered, and the solvent was removed in vacuo. The residue was purified by flash column chromatography (Ø = 4 cm, *h* = 22 cm, *V* = 30 mL, ethyl acetate, *R*_*f*_ = 0.60) to give (*R*)-**33** as a colorless oil (220 mg, 0.52 mmol, 69%). [α]_D_^20^ = +46.7 (1.2, methanol); HPLC (method 1): *t*_R_ = 20.4 min, purity 97.1%.

##### Spectroscopic Data of (*S*)-**33** and (*R*)-**33**

^1^H NMR (DMSO-*d*_6_): δ [ppm] = 2.23 (s br, 1H, OCHCH_2_N*H*), 2.77 (dd, *J* = 12.6/6.5 Hz, 1H, OCHC*H*_2_NH), 2.81 (dd, *J* = 12.6/4.4 Hz, 1H, OCHC*H*_2_NH), 3.65 (s, 3H, CO_2_C*H*_3_), 3.66 (d, *J* = 13.7 Hz, 1H, NHC*H*_2_Ph), 3.69 (d, *J* = 13.7 Hz, 1H, NHC*H*_2_Ph), 4.15 (dd, *J* = 6.4/4.4 Hz, 1H, OC*H*CH_2_NH), 4.41 (d, *J* = 12.1 Hz, 1H, OC*H*_2_Ar), 4.57 (d, *J* = 12.1 Hz, 1H, OC*H*_2_Ar), 7.15–7.19 (m, 2H, 2′-H_4-iodophenyl_, 6′-H_4-iodophenyl_), 7.19–7.23 (m, 1H, 4″-H_phenyl_), 7.25–7.32 (m, 4H, 2″-H_phenyl_, 3″-H_phenyl_, 5″-H_phenyl_, 6″-H_phenyl_), and 7.69–7.72 (m, 2H, 3′-H_4-iodophenyl_, 5′-H_4-iodophenyl_); ^13^C NMR (DMSO-*d*_6_): δ [ppm] = 50.1 (1C, OCH*C*H_2_NH), 51.6 (1C, CO_2_*C*H_3_), 52.4 (1C, NH*C*H_2_Ph), 70.8 (1C, O*C*H_2_Ar), 77.8 (1C, O*C*HCH_2_NH), 93.4 (1C, C-4′_4-iodophenyl_), 126.6 (1C, C-4″_phenyl_), 127.8 (2C, C_arom._), 128.1 (2C, C_arom._), 129.9 (2C, C-2′_4-iodophenyl_, C-6′_4-iodophenyl_), 137.0 (2C, C-3′_4-iodophenyl_, C-5′_4-iodophenyl_), 137.7 (1C, C-1′_4-iodophenyl_), 140.5 (1C, C-1″_phenyl_), and 171.6 (1C, *C*O_2_CH_3_); IR (neat): ν̃ [cm^–1^] = 3026, 2949, 2843, 1746, 1590, 1484, 1453, 1435, 1272, 1199, 1120, 1006, 798, 734, 698, and 472; HRMS (*m*/*z*): [M + H]^+^ calcd for C_18_H_21_INO_3_, 426.0561; found, 426.0549.

#### Methyl (*S*)-3-(benzylamino)-2-[(4-{[4-(morpholinomethyl)phenyl]ethynyl}benzyl)oxy]propanoate ((*S*)-**34**)

Under a N_2_ atmosphere, copper(I) iodide (9 mg, 0.047 mmol), bis(triphenylphosphine)palladium(II) chloride (35 mg, 0.050 mmol), and diisopropylamine (2.5 mL) were added to a solution of (*S*)-**33** (200 mg, 0.46 mmol) in dry THF (12 mL) at ambient temperature, and the mixture was stirred for 20 min. Then, 4-(4-ethynylbenzyl)morpholine (180 mg, 0.87 mmol) was added in two portions at an interval of 30 min. After stirring the reaction mixture for 24 h at ambient temperature, the solvent was removed in vacuo. The residue was dissolved in ethyl acetate and filtered through a short silica gel column. The solvent was removed in vacuo, and the residue was purified by flash column chromatography (Ø = 3 cm, *h* = 15 cm, *V* = 20 mL, ethyl acetate, *R*_*f*_ = 0.22) to give (*S*)-**34** as a colorless oil (160 mg, 0.32 mmol, 68%). [α]_D_^20^ = −22.0 (1.0, methanol); HPLC (method 1): *t*_R_ = 16.9 min, purity 96.5%.

#### Methyl (*R*)-3-(benzylamino)-2-[(4-{[4-(morpholinomethyl)phenyl]ethynyl}benzyl)oxy]propanoate ((*R*)-**34**)

Under a N_2_ atmosphere, copper(I) iodide (8 mg, 0.042 mmol), bis(triphenylphosphine)palladium(II) chloride (22 mg, 0.031 mmol), and diisopropylamine (2.5 mL) were added to a solution of (*R*)-**33** (130 mg, 0.30 mmol) in dry THF (10 mL) at ambient temperature, and the mixture was stirred for 20 min. Then, 4-(4-ethynylbenzyl)morpholine (120 mg, 0.60 mmol) was added in two portions at an interval of 30 min. After stirring the reaction mixture for 24 h at ambient temperature, the solvent was removed in vacuo. The residue was dissolved in ethyl acetate and filtered through a short silica gel column. The solvent was removed in vacuo and the residue was purified by flash column chromatography (Ø = 3 cm, *h* = 16 cm, *V* = 20 mL, ethyl acetate, *R*_*f*_ = 0.22) to give (*R*)-**34** as a colorless oil (100 mg, 0.21 mmol, 69%). [α]_D_^20^ = +23.6 (1.2, methanol); HPLC (method 1): *t*_R_ = 16.7 min, purity 99.1%.

##### Spectroscopic Data of (*S*)-**34** and (*R*)-**34**

^1^H NMR (DMSO-*d*_6_): δ [ppm] = 2.24 (s br, 1H, OCHCH_2_N*H*), 2.31–2.39 (m, 4H, N(C*H*_2_CH_2_)_2_O), 2.79 (dd, *J* = 12.6/6.4 Hz, 1H, OCHC*H*_2_NH), 2.83 (dd, *J* = 12.6/4.4 Hz, 1H, OCHC*H*_2_NH), 3.49 (s, 2H, NC*H*_2_Ar), 3.55–3.60 (m, 4H, N(CH_2_C*H*_2_)_2_O), 3.66 (s, 3H, CO_2_C*H*_3_), 3.67 (d, *J* = 13.8 Hz, 1H, NHC*H*_2_Ph), 3.71 (d, *J* = 13.8 Hz, 1H, NHC*H*_2_Ph), 4.18 (dd, *J* = 6.4/4.4 Hz, 1H, OC*H*CH_2_NH), 4.49 (d, *J* = 12.3 Hz, 1H, OC*H*_2_Ar), 4.65 (d, *J* = 12.3 Hz, 1H, OC*H*_2_Ar), 7.20–7.24 (m, 1H, 4‴-H_phenyl_), 7.26–7.32 (m, 4H, 2‴-H_phenyl_, 3‴-H_phenyl_, 5‴-H_phenyl_, 6‴-H_phenyl_), 7.34–7.38 (m, 2H, 3″-H_4-(morpholinomethyl)phenyl_, 5″-H_4-(morpholinomethyl)phenyl_), 7.39–7.43 (m, 2H, 2′-H_4-{[4-(morpholinomethyl)phenyl]ethynyl}phenyl_, 6′-H_4-{[4-(morpholinomethyl)phenyl]ethynyl}phenyl_), and 7.49–7.54 (m, 4H, 3′-H_4-{[4-(morpholinomethyl)phenyl]ethynyl}phenyl_, 5′-H_4-{[4-(morpholinomethyl)phenyl]ethynyl}phenyl_, 2″-H_4-(morpholinomethyl)phenyl_, 6″-H_4-(morpholinomethyl)phenyl_); ^13^C NMR (DMSO-*d*_6_): δ [ppm] = 50.1 (1C, OCH*C*H_2_NH), 51.6 (1C, CO_2_*C*H_3_), 52.4 (1C, NH*C*H_2_Ph), 53.2 (2C, N(*C*H_2_CH_2_)_2_O), 62.0 (1C, Ar*C*H_2_N), 66.2 (2C, N(CH_2_*C*H_2_)_2_O), 71.0 (1C, O*C*H_2_Ar), 77.9 (1C, O*C*HCH_2_NH), 89.0 (1C, C≡C), 89.3 (1C, C≡C), 120.8 (1C, C-1″_4-(morpholinomethyl)phenyl_), 121.4 (1C, C-4′_4-{[4-(morpholinomethyl)phenyl]ethynyl}phenyl_), 126.6 (1C, C-4‴_phenyl_), 127.9 (4C, C-2′_4-{[4-(morpholinomethyl)phenyl]ethynyl}phenyl_, C-6′_4-{[4-(morpholinomethyl)phenyl]ethynyl}phenyl_, C-2‴_phenyl_, C-6‴_phenyl_), 128.1 (2C, C-3‴_phenyl_, C-5‴_phenyl_), 129.2 (2C, C-3″_4-(morpholinomethyl)phenyl_, C-5″_4-(morpholinomethyl)phenyl_), 131.23 (2C, C_arom._), 131.24 (2C, C_arom._), 138.7 (1C, C-1′_4-{[4-(morpholinomethyl)phenyl]ethynyl}phenyl_), 138.9 (1C, C-4″_4-(morpholinomethyl)phenyl_), 140.6 (1C, C-1‴_phenyl_), and 171.6 (1C, CO_2_CH_3_); IR (neat): ν̃ [cm^–1^] = 2951, 2852, 2808, 1748, 1517, 1453, 1349, 1201, 1114, 1007, 914, 866, 819, 794, 735, 698, 541, and 515; HRMS (*m*/*z*): [M + H]^+^ calcd for C_31_H_35_N_2_O_4_, 499.2591; found, 499.2605.

#### (*S*)-3-(Benzylamino)-*N*-hydroxy-2-[(4-{[4-(morpholinomethyl)phenyl]ethynyl}benzyl)oxy]propanamide ((*S*)-**12a**)

Under ice-cooling, an aqueous solution of hydroxylamine (50 wt %, 2 mL) was added to a solution of (*S*)-**34** (93 mg, 0.19 mmol) in a mixture of THF (4 mL) and isopropanol (4 mL). After stirring the reaction mixture for 5 min at 0 °C, stirring was continued for 48 h at ambient temperature. Then, the solvent was removed in vacuo and the residue was purified by automatic flash column chromatography using a Biotage Isolera One system (20% → 100% ACN in H_2_O, Biotage SNAP Ultra C18 30 g). Fractions containing the desired product were combined and subjected to lyophilization to give (*S*)-**12a** as a colorless solid (65 mg, 0.13 mmol, 70%). mp 59 °C; [α]_D_^20^ = −29.3 (1.5, methanol); HPLC (method 2): *t*_R_ = 12.6 min, purity 98.8%.

#### (*R*)-3-(Benzylamino)-*N*-hydroxy-2-[(4-{[4-(morpholinomethyl)phenyl]ethynyl}benzyl)oxy]propanamide ((*R*)-**12a**)

Under ice-cooling, an aqueous solution of hydroxylamine (50 wt %, 2 mL) was added to a solution of (*R*)-**34** (58 mg, 0.12 mmol) in a mixture of THF (4 mL) and isopropanol (4 mL). After stirring the reaction mixture for 5 min at 0 °C, stirring was continued for 48 h at ambient temperature. Then, the solvent was removed in vacuo and the residue was purified by automatic flash column chromatography using a Biotage Isolera One system (20% → 100% ACN in H_2_O, Biotage SNAP Ultra C18 30 g). Fractions containing the desired product were combined and subjected to lyophilization to give (*R*)-**12a** as a colorless solid (36 mg, 0.072 mmol, 62%). mp 59 °C; [α]_D_^20^ = +32.5 (2.0, methanol); HPLC (method 2): *t*_R_ = 12.6 min, purity 99.2%.

##### Spectroscopic Data of (*S*)-**12a** and (*R*)-**12a**

^1^H NMR (DMSO-*d*_6_): δ [ppm] = 2.30–2.40 (m, 4H, N(C*H*_2_CH_2_)_2_O), 2.72 (dd, *J* = 12.5/4.9 Hz, 1H, OCHC*H*_2_NH), 2.77 (dd, *J* = 12.5/6.9 Hz, 1H, OCHC*H*_2_NH), 3.49 (s, 2H, NC*H*_2_Ar), 3.55–3.61 (m, 4H, N(CH_2_C*H*_2_)_2_O), 3.68 (s, 2H, NHC*H*_2_Ph), 3.89 (dd, *J* = 6.8/4.9 Hz, 1H, OC*H*CH_2_NH), 4.43 (d, *J* = 12.5 Hz, 1H, OC*H*_2_Ar), 4.59 (d, *J* = 12.5 Hz, 1H, OC*H*_2_Ar), 7.17–7.26 (m, 1H, 4‴-H_phenyl_), 7.26–7.33 (m, 4H, 2‴-H_phenyl_, 3‴-H_phenyl_, 5‴-H_phenyl_, 6‴-H_phenyl_), 7.33–7.39 (m, 2H, 3″-H_4-(morpholinomethyl)phenyl_, 5″-H_4-(morpholinomethyl)phenyl_), 7.39–7.45 (m, 2H, 2′-H_4-{[4-(morpholinomethyl)phenyl]ethynyl}phenyl_, 6′-H_4-{[4-(morpholinomethyl)phenyl]ethynyl}phenyl_), 7.48–7.55 (m, 4H, 3′-H_4-{[4-(morpholinomethyl)phenyl]ethynyl}phenyl_, 5′-H_4-{[4-(morpholinomethyl)phenyl]ethynyl}phenyl_, 2″-H_4-(morpholinomethyl)phenyl_, 6″-H_4-(morpholinomethyl)phenyl_), and 8.88 (s br, 1H, CON*H*OH), the signals for OCHCH_2_N*H* and CONHO*H* cannot be observed in the spectrum; ^13^C NMR (DMSO-*d*_6_): δ [ppm] = 50.5 (1C, OCH*C*H_2_NH), 52.6 (1C, NH*C*H_2_Ph), 53.2 (2C, N(*C*H_2_CH_2_)_2_O), 62.0 (1C, Ar*C*H_2_N), 66.2 (2C, N(CH_2_*C*H_2_)_2_O), 70.6 (1C, O*C*H_2_Ar), 77.8 (1C, O*C*HCH_2_NH), 89.1 (1C, C≡C), 89.3 (1C, C≡C), 120.8 (1C, C-1″_4-(morpholinomethyl)phenyl_), 121.4 (1C, C-4′_4-{[4-(morpholinomethyl)phenyl]ethynyl}phenyl_), 126.6 (1C, C-4‴_phenyl_), 127.85 (2C, C_arom._), 127.90 (2C, C_arom._), 128.1 (2C, C-3‴_phenyl_, C-5‴_phenyl_), 129.2 (2C, C-3″_4-(morpholinomethyl)phenyl_, C-5″_4-(morpholinomethyl)phenyl_), 131.18 (2C, C_arom._), 131.23 (2C, C_arom._), 138.78 (1C, C_arom._), 138.84 (1C, C_arom._), 140.6 (1C, C-1‴_phenyl_), and 167.0 (1C, *C*ONHOH); IR (neat): ν̃ [cm^–1^] = 3179, 3028, 2853, 2808, 1660, 1517, 1453, 1349, 1332, 1308, 1291, 1114, 1007, 914, 865, 820, 792, 741, 698, 539, and 517; HRMS (*m*/*z*): [M + H]^+^ calcd for C_30_H_34_N_3_O_4_, 500.2544; found, 500.2555.

#### Methyl (*S*)-3-(dibenzylamino)-2-[(4-iodobenzyl)oxy]propanoate ((*S*)-**35**)

Under a N_2_ atmosphere, polymer-bound triphenylphosphine (∼3 mmol/g triphenylphosphine loading, 360 mg, 1.1 mmol) was added to a solution of (*S*)-**28** (200 mg, 0.55 mmol) in a mixture of tetrahydrofuran (12 mL) and water (8 mL). After stirring the reaction mixture for 24 h at 40 °C, the mixture was filtered and the solvent was removed under high vacuum. The residue was taken up in 1,2-dichloroethane (10 mL) and freshly distilled benzaldehyde (0.12 mL, 130 mg, 1.2 mmol) was added to the suspension. Under ice-cooling, NaBH(OAc)_3_ (250 mg, 1.2 mmol) was added to the reaction mixture. After stirring the reaction mixture for 5 min at 0 °C, stirring was continued for 72 h at ambient temperature. Then, a saturated aqueous solution of NaHCO_3_ was added and the mixture was extracted with ethyl acetate (3×). The combined organic layers were dried (Na_2_SO_4_), filtered, and the solvent was removed in vacuo. The residue was taken up in methanol (10 mL) and concentrated sulfuric acid (0.03 mL, 55 mg, 0.56 mmol) was added. After stirring the reaction mixture for 72 h at 80 °C, the solvent was concentrated in vacuo, water was added, and the mixture was extracted with ethyl acetate (3×). The combined organic layers were dried (Na_2_SO_4_), filtered, and the solvent was removed in vacuo. The residue was purified by flash column chromatography (Ø = 3 cm, *h* = 16 cm, *V* = 20 mL, petroleum ether/ethyl acetate = 10:1 → 4:1) to give (*S*)-**35** as a colorless oil (140 mg, 0.28 mmol, 50%). *R*_*f*_ = 0.44 (petroleum ether/ethyl acetate = 10:1); [α]_D_^20^ = −7.9 (1.6, methanol); HPLC (method 1): *t*_R_ = 23.6 min, purity 98.9%.

#### Methyl (*R*)-3-(dibenzylamino)-2-[(4-iodobenzyl)oxy]propanoate ((*R*)-**35**)

Under a N_2_ atmosphere, polymer-bound triphenylphosphine (∼3 mmol/g triphenylphosphine loading, 430 mg, 1.3 mmol) was added to a solution of (*R*)-**28** (170 mg, 0.46 mmol) in a mixture of tetrahydrofuran (12 mL) and water (8 mL). After stirring the reaction mixture for 24 h at 40 °C, the mixture was filtered and the solvent was removed in high vacuum. The residue was taken up in 1,2-dichloroethane (10 mL) and freshly distilled benzaldehyde (0.10 mL, 100 mg, 0.98 mmol) was added to the suspension. Under ice-cooling, NaBH(OAc)_3_ (210 mg, 0.97 mmol) was added to the reaction mixture. After stirring the reaction mixture for 5 min at 0 °C, stirring was continued for 72 h at ambient temperature. Then, a saturated aqueous solution of NaHCO_3_ was added and the mixture was extracted with ethyl acetate (3×). The combined organic layers were dried (Na_2_SO_4_), filtered, and the solvent was removed in vacuo. The residue was taken up in methanol (10 mL) and concentrated sulfuric acid (0.03 mL, 55 mg, 0.56 mmol) was added. After stirring the reaction mixture for 72 h at 80 °C, the solvent was concentrated in vacuo, water was added, and the mixture was extracted with ethyl acetate (3×). The combined organic layers were dried (Na_2_SO_4_), filtered, and the solvent was removed in vacuo. The residue was purified by flash column chromatography (Ø = 3 cm, *h* = 24 cm, *V* = 20 mL, petroleum ether/ethyl acetate = 10:1 → 4:1) to give (*R*)-**35** as a colorless oil (100 mg, 0.20 mmol, 43%). *R*_*f*_ = 0.44 (petroleum ether/ethyl acetate = 10:1); [α]_D_^20^ = +9.4 (1.2, methanol); HPLC (method 1): *t*_R_ = 23.6 min, purity 100%.

##### Spectroscopic Data of (*S*)-**35** and (*R*)-**35**

^1^H NMR (DMSO-*d*_6_): δ [ppm] = 2.72 (dd, *J* = 13.4/4.8 Hz, 1H, OCHC*H*_2_N), 2.77 (dd, *J* = 13.4/6.2 Hz, 1H, OCHC*H*_2_N), 3.48 (d, *J* = 13.7 Hz, 2H, N(C*H*_2_Ph)_2_), 3.60 (s, 3H, CO_2_C*H*_3_), 3.65 (d, *J* = 13.7 Hz, 2H, N(C*H*_2_Ph)_2_), 4.27 (dd, *J* = 6.2/4.8 Hz, 1H, OC*H*CH_2_N), 4.38 (d, *J* = 12.0 Hz, 1H, OC*H*_2_Ar), 4.50 (d, *J* = 12.0 Hz, 1H, OC*H*_2_Ar), 7.11–7.15 (m, 2H, 2′-H_4-iodophenyl_, 6′-H_4-iodophenyl_), 7.20–7.25 (m, 2H, 4″-H_phenyl_), 7.25–7.29 (m, 4H, 2″-H_phenyl_, 6″-H_phenyl_), 7.29–7.33 (m, 4H, 3″-H_phenyl_, 5″-H_phenyl_), and 7.68–7.73 (m, 2H, 3′-H_4-iodophenyl_, 5′-H_4-iodophenyl_); ^13^C NMR (DMSO-*d*_6_): δ [ppm] = 51.5 (1C, CO_2_*C*H_3_), 54.7 (1C, OCH*C*H_2_N), 57.9 (2C, N(*C*H_2_Ph)_2_), 70.8 (1C, O*C*H_2_Ar), 77.6 (1C, O*C*HCH_2_N), 93.6 (1C, C-4′_4-iodophenyl_), 126.9 (2C, C-4″_phenyl_), 128.1 (4C, C-3″_phenyl_, C-5″_phenyl_), 128.6 (4C, C-2″_phenyl_, C-6″_phenyl_), 129.8 (2C, C-2′_4-iodophenyl_, C-6′_4-iodophenyl_), 137.0 (2C, C-3′_4-iodophenyl_, C-5′_4-iodophenyl_), 137.6 (1C, C-1′_4-iodophenyl_), 139.0 (2C, C-1″_phenyl_), and 171.3 (1C, *C*O_2_CH_3_); IR (neat): ν̃ [cm^–1^] = 3026, 2799, 1747, 1589, 1484, 1452, 1201, 1096, 1006, 798, 745, 697, and 472; HRMS (*m*/*z*): [M + H]^+^ calcd for C_25_H_27_INO_3_, 516.1030; found, 516.1031.

#### Methyl (*S*)-3-(dibenzylamino)-2-[(4-{[4-(morpholinomethyl)phenyl]ethynyl}benzyl)oxy]propanoate ((*S*)-**36**)

Under a N_2_ atmosphere, copper(I) iodide (5 mg, 0.026 mmol), bis(triphenylphosphine)palladium(II) chloride (18 mg, 0.026 mmol), and diisopropylamine (3 mL) were added to a solution of (*S*)-**35** (120 mg, 0.23 mmol) in dry THF (12 mL) at ambient temperature, and the mixture was stirred for 20 min. Then, 4-(4-ethynylbenzyl)morpholine (130 mg, 0.66 mmol) was added in two portions at an interval of 30 min. After stirring the reaction mixture for 24 h at ambient temperature, the solvent was removed in vacuo. The residue was dissolved in a mixture of petroleum ether and ethyl acetate (1:1) and filtered through a short silica gel column. The solvent was removed in vacuo and the residue was purified by flash column chromatography (Ø = 3 cm, *h* = 22 cm, *V* = 20 mL, petroleum ether/ethyl acetate = 1:1, *R*_*f*_ = 0.44) to give (*S*)-**36** as a yellow oil (120 mg, 0.21 mmol, 87%). [α]_D_^20^ = −12.1 (1.1, methanol); HPLC (method 1): *t*_R_ = 19.4 min, purity 98.1%.

#### Methyl (*R*)-3-(dibenzylamino)-2-[(4-{[4-(morpholinomethyl)phenyl]ethynyl}benzyl)oxy]propanoate ((*R*)-**36**)

Under a N_2_ atmosphere, copper(I) iodide (4 mg, 0.021 mmol), bis(triphenylphosphine)palladium(II) chloride (14 mg, 0.020 mmol), and diisopropylamine (2.5 mL) were added to a solution of (*R*)-**35** (100 mg, 0.19 mmol) in dry THF (12 mL) at ambient temperature, and the mixture was stirred for 20 min. Then, 4-(4-ethynylbenzyl)morpholine (120 mg, 0.61 mmol) was added in two portions at an interval of 30 min. After stirring the reaction mixture for 24 h at ambient temperature, the solvent was removed in vacuo. The residue was dissolved in a mixture of petroleum ether and ethyl acetate (1:1) and filtered through a short silica gel column. The solvent was removed in vacuo and the residue was purified by flash column chromatography (Ø = 2 cm, *h* = 19 cm, *V* = 10 mL, petroleum ether/ethyl acetate = 1:1, *R*_*f*_ = 0.44) to give (*R*)-**36** as a yellow oil (92 mg, 0.16 mmol, 81%). [α]_D_^20^ = +10.0 (1.1, methanol); HPLC (method 1): *t*_R_ = 19.4 min, purity 100%.

##### Spectroscopic Data of (*S*)-**36** and (*R*)-**36**

^1^H NMR (DMSO-*d*_6_): δ [ppm] = 2.31–2.39 (m, 4H, N(C*H*_2_CH_2_)_2_O), 2.75 (dd, *J* = 13.4/4.8 Hz, 1H, OCHC*H*_2_N), 2.80 (dd, *J* = 13.4/6.2 Hz, 1H, OCHC*H*_2_N), 3.49 (s, 2H, NC*H*_2_Ar), 3.51 (d, *J* = 13.7 Hz, 2H, N(C*H*_2_Ph)_2_), 3.55–3.60 (m, 4H, N(CH_2_C*H*_2_)_2_O), 3.61 (s, 3H, CO_2_C*H*_3_), 3.67 (d, *J* = 13.7 Hz, 2H, N(C*H*_2_Ph)_2_), 4.29–4.32 (m, 1H, OC*H*CH_2_N), 4.47 (d, *J* = 12.2 Hz, 1H, OC*H*_2_Ar), 4.58 (d, *J* = 12.2 Hz, 1H, OC*H*_2_Ar), 7.21–7.26 (m, 2H, 4‴-H_phenyl_), 7.27–7.34 (m, 8H, 2‴-H_phenyl_, 3‴-H_phenyl_, 5‴-H_phenyl_, 6‴-H_phenyl_), 7.34–7.39 (m, 4H, 2′-H_4-{[4-(morpholinomethyl)phenyl]ethynyl}phenyl_, 6′-H_4-{[4-(morpholinomethyl)phenyl]ethynyl}phenyl_, 3″-H_4-(morpholinomethyl)phenyl_, 5″-H_4-(morpholinomethyl)phenyl_), and 7.48–7.55 (m, 4H, 3′-H_4-{[4-(morpholinomethyl)phenyl]ethynyl}phenyl_, 5′-H_4-{[4-(morpholinomethyl)phenyl]ethynyl}phenyl_, 2″-H_4-(morpholinomethyl)phenyl_, 6″-H_4-(morpholinomethyl)phenyl_); ^13^C NMR (DMSO-*d*_6_): δ [ppm] = 51.5 (1C, CO_2_*C*H_3_), 53.2 (2C, N(*C*H_2_CH_2_)_2_O), 54.7 (1C, OCH*C*H_2_N), 58.0 (2C, N(*C*H_2_Ph)_2_), 62.0 (1C, Ar*C*H_2_N), 66.2 (2C, N(CH_2_*C*H_2_)_2_O), 71.1 (1C, O*C*H_2_Ar), 77.7 (1C, O*C*HCH_2_N), 89.0 (1C, C≡C), 89.3 (1C, C≡C), 120.8 (1C, C-1″_4-(morpholinomethyl)phenyl_), 121.5 (1C, C-4′_4-{[4-(morpholinomethyl)phenyl]ethynyl}phenyl_), 126.9 (2C, C-4‴_phenyl_), 127.8 (2C, C-2′_4-{[4-(morpholinomethyl)phenyl]ethynyl}phenyl_, C-6′_4-{[4-(morpholinomethyl)phenyl]ethynyl}phenyl_), 128.1 (4C, C-3‴_phenyl_, C-5‴_phenyl_), 128.6 (4C, C-2‴_phenyl_, C-6‴_phenyl_), 129.2 (2C, C-3″_4-(morpholinomethyl)phenyl_, C-5″_4-(morpholinomethyl)phenyl_), 131.2 (4C, C-3′_4-{[4-(morpholinomethyl)phenyl]ethynyl}phenyl_, C-5′_4-{[4-(morpholinomethyl)phenyl]ethynyl}phenyl_, C-2″_4-(morpholinomethyl)phenyl_, C-6″_4-(morpholinomethyl)phenyl_), 138.5 (1C, C-1′_4-{[4-(morpholinomethyl)phenyl]ethynyl}phenyl_), 138.9 (1C, C-4″_4-(morpholinomethyl)phenyl_), 139.0 (2C, C-1‴_phenyl_), and 171.4 (1C, *C*O_2_CH_3_); IR (neat): ν̃ [cm^–1^] = 3027, 2950, 2852, 2803, 1748, 1517, 1494, 1453, 1349, 1290, 1261, 1203, 1114, 1098, 1007, 978, 913, 866, 819, 746, 698, 540, and 515; HRMS (*m*/*z*): [M + H]^+^ calcd for C_38_H_41_N_2_O_4_, 589.3061; found, 589.3081.

#### (*S*)-3-(Dibenzylamino)-*N*-hydroxy-2-[(4-{[4-(morpholinomethyl)phenyl]ethynyl}benzyl)oxy]propanamide ((*S*)-**37**)

Under ice-cooling, an aqueous solution of hydroxylamine (50 wt %, 2 mL) was added to a solution of (*S*)-**36** (78 mg, 0.13 mmol) in a mixture of THF (4 mL) and isopropanol (4 mL). After stirring the reaction mixture for 5 min at 0 °C, stirring was continued for 48 h at ambient temperature. Then, the solvent was removed in vacuo and the residue was purified by automatic flash column chromatography using a Biotage Isolera One system (10% → 90% ACN in H_2_O, Biotage SNAP Ultra C18 30 g). Fractions containing the desired product were combined and subjected to lyophilization to give (*S*)-**37** as a colorless solid (37 mg, 0.063 mmol, 47%). mp 79 °C; [α]_D_^20^ = −22.0 (1.3, methanol); HPLC (method 2): *t*_R_ = 13.5 min, purity 100%.

#### (*R*)-3-(Dibenzylamino)-*N*-hydroxy-2-[(4-{[4-(morpholinomethyl)phenyl]ethynyl}benzyl)oxy]propanamide ((*R*)-**37**)

Under ice-cooling, an aqueous solution of hydroxylamine (50 wt %, 2 mL) was added to a solution of (*R*)-**36** (70 mg, 0.12 mmol) in a mixture of THF (4 mL) and isopropanol (4 mL). After stirring the reaction mixture for 5 min at 0 °C, stirring was continued for 48 h at ambient temperature. Then, the solvent was removed in vacuo and the residue was purified by automatic flash column chromatography using a Biotage Isolera One system (20% → 100% ACN in H_2_O, Biotage SNAP Ultra C18 30 g). Fractions containing the desired product were combined and subjected to lyophilization to give (*R*)-**37** as a colorless solid (35 mg, 0.059 mmol, 50%). mp 79 °C; [α]_D_^20^ = +28.5 (1.1, methanol); HPLC (method 2): *t*_R_ = 13.6 min, purity 100%.

##### Spectroscopic Data of (*S*)-**37** and (*R*)-**37**

^1^H NMR (DMSO-*d*_6_): δ [ppm] = 2.31–2.40 (m, 4H, N(C*H*_2_CH_2_)_2_O), 2.67 (dd, *J* = 13.6/4.4 Hz, 1H, OCHC*H*_2_N), 2.74 (dd, *J* = 13.6/7.3 Hz, 1H, OCHC*H*_2_N), 3.49 (s, 2H, NC*H*_2_Ar), 3.53 (d, *J* = 14.0 Hz, 2H, N(C*H*_2_Ph)_2_), 3.55–3.61 (m, 4H, N(CH_2_C*H*_2_)_2_O), 3.64 (d, *J* = 14.0 Hz, 2H, N(C*H*_2_Ph)_2_), 4.04 (dd, *J* = 7.3/4.4 Hz, 1H, OC*H*CH_2_N), 4.41 (d, *J* = 12.4 Hz, 1H, OC*H*_2_Ar), 4.55 (d, *J* = 12.4 Hz, 1H, OC*H*_2_Ar), 7.20–7.25 (m, 2H, 4‴-H_phenyl_), 7.27–7.34 (m, 8H, 2‴-H_phenyl_, 3‴-H_phenyl_, 5‴-H_phenyl_, 6‴-H_phenyl_), 7.35–7.38 (m, 2H, 3″-H_4-(morpholinomethyl)phenyl_, 5″-H_4-(morpholinomethyl)phenyl_), 7.38–7.42 (m, 2H, 2′-H_4-{[4-(morpholinomethyl)phenyl]ethynyl}phenyl_, 6′-H_4-{[4-(morpholinomethyl)phenyl]ethynyl}phenyl_), 7.49–7.55 (m, 4H, 3′-H_4-{[4-(morpholinomethyl)phenyl]ethynyl}phenyl_, 5′-H_4-{[4-(morpholinomethyl)phenyl]ethynyl}phenyl_, 2″-H_4-(morpholinomethyl)phenyl_, 6″-H_4-(morpholinomethyl)phenyl_), 8.93 (s br, 1H, CON*H*OH), and 10.78 (s br, 1H, CONHO*H*); ^13^C NMR (DMSO-*d*_6_): δ [ppm] = 53.1 (2C, N(*C*H_2_CH_2_)_2_O), 55.6 (1C, OCH*C*H_2_N), 57.5 (2C, N(*C*H_2_Ph)_2_), 62.0 (1C, Ar*C*H_2_N), 66.2 (2C, N(CH_2_*C*H_2_)_2_O), 70.5 (1C, O*C*H_2_Ar), 76.8 (1C, O*C*HCH_2_N), 89.1 (1C, C≡C), 89.3 (1C, C≡C), 120.8 (1C, C-1″_4-(morpholinomethyl)phenyl_), 121.4 (1C, C-4′_4-{[4-(morpholinomethyl)phenyl]ethynyl}phenyl_), 126.8 (2C, C-4‴_phenyl_), 127.8 (2C, C-2′_4-{[4-(morpholinomethyl)phenyl]ethynyl}phenyl_, C-6′_4-{[4-(morpholinomethyl)phenyl]ethynyl}phenyl_), 128.1 (4C, C-3‴_phenyl_, C-5‴_phenyl_), 128.6 (4C, C-2‴_phenyl_, C-6‴_phenyl_), 129.2 (2C, C-3″_4-(morpholinomethyl)phenyl_, C-5″_4-(morpholinomethyl)phenyl_), 131.17 (2C, C_arom._), 131.23 (2C, C_arom._), 138.7 (1C, C-1′_4-{[4-(morpholinomethyl)phenyl]ethynyl}phenyl_), 138.8 (1C, C-4″_4-(morpholinomethyl)phenyl_), 139.0 (2C, C-1‴_phenyl_), and 166.8 (1C, *C*ONHOH); IR (neat): ν̃ [cm^–1^] = 3181, 3027, 2854, 2806, 1666, 1517, 1494, 1453, 1349, 1112, 1006, 914, 864, 819, 792, 746, 698, 539, and 517; HRMS (*m*/*z*): [M + H]^+^ calcd for C_37_H_40_N_3_O_4_, 590.3013; found, 590.3019.

#### Methyl (*S*)-3-benzamido-2-[(4-iodobenzyl)oxy]propanoate ((*S*)-**38a**)

Under a N_2_ atmosphere, a 1.0 M solution of trimethylphosphane in toluene (1.5 mL, 1.5 mmol) was added to an ice-cooled mixture of benzoic acid (82 mg, 0.67 mmol), (*S*)-**28** (220 mg, 0.6 mmol), and 2,2′-dithiodipyridine (75 mg, 0.34 mmol). After stirring the reaction mixture for 20 min at 0 °C, the ice-bath was removed, and the mixture was stirred for 24 h at ambient temperature. Then, water (1.5 mL) was added, and the mixture was stirred for 20 min. Afterward, the mixture was diluted with dichloromethane and washed with a saturated aqueous solution of NaHCO_3_, an ice-cold solution of 1.0 M HCl, and water. The combined organic layers were dried (Na_2_SO_4_), filtered, and the solvent was removed in vacuo. The residue was purified by flash column chromatography (Ø = 3 cm, *h* = 21 cm, *V* = 20 mL, petroleum ether/ethyl acetate = 2:1, *R*_*f*_ = 0.37) to give (*S*)-**38a** as a colorless solid (220 mg, 0.51 mmol, 83%). mp 117 °C; [α]_D_^20^ = −39.6 (2.7, methanol); HPLC (method 1): *t*_R_ = 22.7 min, purity 99.5%.

#### Methyl (*R*)-3-benzamido-2-[(4-iodobenzyl)oxy]propanoate ((*R*)-**38a**)

Under a N_2_ atmosphere, a 1.0 M solution of trimethylphosphane in toluene (1.7 mL, 1.7 mmol) was added to an ice-cooled mixture of benzoic acid (82 mg, 0.67 mmol), (*R*)-**28** (240 mg, 0.66 mmol), and 2,2′-dithiodipyridine (74 mg, 0.34 mmol). After stirring the reaction mixture for 20 min at 0 °C, the ice-bath was removed, and the mixture was stirred for 24 h at ambient temperature. Then, water (1.0 mL) was added, and the mixture was stirred for 20 min. Afterward, the mixture was diluted with dichloromethane and washed with a saturated aqueous solution of NaHCO_3_, an ice-cold solution of 1.0 M HCl, and water. The combined organic layers were dried (Na_2_SO_4_), filtered, and the solvent was removed in vacuo. The residue was purified by flash column chromatography (Ø = 3 cm, *h* = 26 cm, *V* = 20 mL, petroleum ether/ethyl acetate = 2:1, *R*_*f*_ = 0.37) to give (*R*)-**38a** as a colorless solid (190 mg, 0.43 mmol, 65%). mp 117 °C; [α]_D_^20^ = +38.3 (6.8, methanol); HPLC (method 1): *t*_R_ = 22.8 min, purity 99.4%.

##### Spectroscopic Data of (*S*)-**38a** and (*R*)-**38a**

^1^H NMR (DMSO-*d*_6_): δ [ppm] = 3.45–3.54 (m, 1H, OCHC*H*_2_NH), 3.60–3.71 (m, 4H, OCHC*H*_2_NH (1H), CO_2_C*H*_3_), 4.19 (dd, *J* = 7.3/5.0 Hz, 1H, OC*H*CH_2_NH), 4.41 (d, *J* = 12.3 Hz, 1H, OC*H*_2_Ar), 4.58 (d, *J* = 12.3 Hz, 1H, OC*H*_2_Ar), 7.10–7.17 (m, 2H, 2′-H_4-iodophenyl_, 6′-H_4-iodophenyl_), 7.43–7.50 (m, 2H, 3″-H_benzoyl_, 5″-H_benzoyl_), 7.50–7.57 (m, 1H, 4″-H_benzoyl_), 7.58–7.65 (m, 2H, 3′-H_4-iodophenyl_, 5′-H_4-iodophenyl_), 7.77–7.84 (m, 2H, 2″-H_benzoyl_, 6″-H_benzoyl_), and 8.67 (t, *J* = 5.9 Hz, 1H, CON*H*); ^13^C NMR (DMSO-*d*_6_): δ [ppm] = 41.4 (1C, OCH*C*H_2_NH), 51.9 (1C, CO_2_*C*H_3_), 70.7 (1C, O*C*H_2_Ar), 76.5 (1C, O*C*HCH_2_NH), 93.6 (1C, C-4′_4-iodophenyl_), 127.2 (2C, C-2″_benzoyl_, C-6″_benzoyl_), 128.3 (2C, C-3″_benzoyl_, C-5″_benzoyl_), 129.9 (2C, C-2′_4-iodophenyl_, C-6′_4-iodophenyl_), 131.3 (1C, C-4″_benzoyl_), 134.2 (1C, C-1″_benzoyl_), 136.9 (2C, C-3′_4-iodophenyl_, C-5′_4-iodophenyl_), 137.5 (1C, C-1′_4-iodophenyl_), 166.5 (1C, *C*ONH), and 171.0 (1C, *C*O_2_CH_3_); IR (neat): ν̃ [cm^–1^] = 3279, 2948, 1741, 1635, 1532, 1485, 1257, 1205, 1139, 1121, 1007, 796, 694, and 433; HRMS (*m*/*z*): [M + Na]^+^ calcd for C_18_H_18_INNaO_4_, 462.0173; found, 462.0162.

#### Methyl (*S*)-3-benzamido-2-[(4-{[4-(morpholinomethyl)phenyl]ethynyl}benzyl)oxy]propanoate ((*S*)-**39a**)

Under a N_2_ atmosphere, copper(I) iodide (6.8 mg, 0.036 mmol), bis(triphenylphosphine)palladium(II) chloride (26 mg, 0.037 mmol), and diisopropylamine (3 mL) were added to a solution of (*S*)-**38a** (160 mg, 0.36 mmol) in dry THF (15 mL) at ambient temperature and the mixture was stirred for 20 min. Then, 4-(4-ethynylbenzyl)morpholine (170 mg, 0.82 mmol) was added in two portions at an interval of 30 min. After stirring the reaction mixture for 24 h at ambient temperature, the solvent was removed in vacuo. The residue was dissolved in a mixture of petroleum ether and ethyl acetate (1:4) and filtered through a short silica gel column. The solvent was removed in vacuo and the residue was purified by flash column chromatography (Ø = 4 cm, *h* = 13 cm, *V* = 30 mL, petroleum ether/ethyl acetate = 1:4, *R*_*f*_ = 0.24) to give (*S*)-**39a** as a yellow oil (170 mg, 0.34 mmol, 94%). [α]_D_^20^ = −31.5 (3.4, methanol); HPLC (method 2): *t*_R_ = 14.1 min, purity 95.9%.

#### Methyl (*R*)-3-benzamido-2-[(4-{[4-(morpholinomethyl)phenyl]ethynyl}benzyl)oxy]propanoate ((*R*)-**39a**)

Under a N_2_ atmosphere, copper(I) iodide (27 mg, 0.14 mmol), bis(triphenylphosphine)palladium(II) chloride (65 mg, 0.093 mmol), and diisopropylamine (10 mL) were added to a solution of (*R*)-**38a** (190 mg, 0.44 mmol) in dry THF (5 mL) at ambient temperature and the mixture was stirred for 20 min. Then, 4-(4-ethynylbenzyl)morpholine (130 mg, 0.65 mmol) was added in two portions at an interval of 30 min. After stirring the reaction mixture for 24 h at ambient temperature, the solvent was removed in vacuo. The residue was dissolved in a mixture of petroleum ether and ethyl acetate (1:4) and filtered through a short silica gel column. The solvent was removed in vacuo and the residue was purified by flash column chromatography (Ø = 3 cm, *h* = 21 cm, *V* = 20 mL, petroleum ether/ethyl acetate = 1:2 → 1:4) to give (*R*)-**39a** as a yellow oil (150 mg, 0.29 mmol, 66%). *R*_*f*_ = 0.24 (petroleum ether/ethyl acetate = 1:4); [α]_D_^20^ = +29.1 (3.5, methanol); HPLC (method 2): *t*_R_ = 14.1 min, purity 96.1%.

##### Spectroscopic Data of (*S*)-**39a** and (*R*)-**39a**

^1^H NMR (DMSO-*d*_6_): δ [ppm] = 2.30–2.41 (m, 4H, N(C*H*_2_CH_2_)_2_O), 3.57–3.61 (m, 7H, NC*H*_2_Ar, N(CH_2_C*H*_2_)_2_O, OCHC*H*_2_NH (1H)), 3.61–3.72 (m, 4H, OCHC*H*_2_NH (1H), CO_2_C*H*_3_), 4.24 (dd, *J* = 7.2/5.1 Hz, 1H, OC*H*CH_2_NH), 4.50 (d, *J* = 12.5 Hz, 1H, OC*H*_2_Ar), 4.66 (d, *J* = 12.5 Hz, 1H, OC*H*_2_Ar), 7.32–7.42 (m, 4H, 2′-H_4-{[4-(morpholinomethyl)phenyl]ethynyl}phenyl_, 6′-H_4-{[4-(morpholinomethyl)phenyl]ethynyl}phenyl_, 3″-H_4-(morpholinomethyl)phenyl_, 5″-H_4-(morpholinomethyl)phenyl_), 7.42–7.55 (m, 7H, 3′-H_4-{[4-(morpholinomethyl)phenyl]ethynyl}phenyl_, 5′-H_4-{[4-(morpholinomethyl)phenyl]ethynyl}phenyl_, 2″-H_4-(morpholinomethyl)phenyl_, 6″-H_4-(morpholinomethyl)phenyl_, 3‴-H_benzoyl_, 4‴-H_benzoyl_, 5‴-H_benzoyl_), 7.79–7.86 (m, 2H, 2‴-H_benzoyl_, 6‴-H_benzoyl_), and 8.68 (t, *J* = 5.9 Hz, 1H, CON*H*); ^13^C NMR (DMSO-*d*_6_): δ [ppm] = 41.4 (1C, OCH*C*H_2_NH), 51.9 (1C, CO_2_*C*H_3_), 53.1 (2C, N(*C*H_2_CH_2_)_2_O), 62.0 (1C, Ar*C*H_2_N), 66.2 (2C, N(CH_2_*C*H_2_)_2_O), 70.9 (1C, O*C*H_2_Ar), 76.6 (1C, O*C*HCH_2_NH), 89.0 (1C, C≡C), 89.3 (1C, C≡C), 120.8 (1C, C-1″_4-(morpholinomethyl)phenyl_), 121.5 (1C, C-4′_4-{[4-(morpholinomethyl)phenyl]ethynyl}phenyl_), 127.2 (2C, C-2‴_benzoyl_, C-6‴_benzoyl_), 127.8 (2C, C-2′_4-{[4-(morpholinomethyl)phenyl]ethynyl}phenyl_, C-6′_4-{[4-(morpholinomethyl)phenyl]ethynyl}phenyl_), 128.3 (2C, C-3‴_benzoyl_, C-5‴_benzoyl_), 129.2 (2C, C-3″_4-(morpholinomethyl)phenyl_, C-5″_4-(morpholinomethyl)phenyl_), 131.15 (2C, C_arom._), 131.21 (3C, C-4‴_benzoyl_, C_arom._), 134.2 (1C, C-1‴_benzoyl_), 138.4 (1C, C-1′_4-{[4-(morpholinomethyl)phenyl]ethynyl}phenyl_), 138.9 (1C, C-4″_4-(morpholinomethyl)phenyl_), 166.5 (1C, *C*ONH), and 171.0 (1C, *C*O_2_CH_3_); IR (neat): ν̃ [cm^–1^] = 3336, 2951, 2855, 2808, 1745, 1645, 1518, 1290, 1205, 1113, 1006, 914, 864, 819, 693, and 540; HRMS (*m*/*z*): [M + H]^+^ calcd for C_31_H_33_N_2_O_5_, 513.2384; found, 513.2351.

#### (*S*)-*N*-{3-(Hydroxyamino)-2-[(4-{[4-(morpholinomethyl)phenyl]ethynyl}benzyl)oxy]-3-oxopropyl}benzamide ((*S*)-**13a**)

Under ice-cooling, an aqueous solution of hydroxylamine (50 wt %, 3 mL) was added to a solution of (*S*)-**39a** (140 mg, 0.27 mmol) in a mixture of THF (5 mL) and isopropanol (5 mL). After stirring the reaction mixture for 5 min at 0 °C, stirring was continued for 24 h at ambient temperature. Then, the solvent was removed in vacuo and the residue was purified by automatic flash column chromatography using a Biotage Isolera One system (10% → 80% ACN in H_2_O, Biotage SNAP Ultra C18 12 g). Fractions containing the desired product were combined and subjected to lyophilization to give (*S*)-**13a** as a colorless solid (56 mg, 0.11 mmol, 41%). mp 86 °C; [α]_D_^20^ = −47.0 (1.0, methanol); HPLC (method 2): *t*_R_ = 12.9 min, purity 100%.

#### (*R*)-*N*-{3-(Hydroxyamino)-2-[(4-{[4-(morpholinomethyl)phenyl]ethynyl}benzyl)oxy]-3-oxopropyl}benzamide ((*R*)-**13a**)

Under ice-cooling, an aqueous solution of hydroxylamine (50 wt %, 2.5 mL) was added to a solution of (*R*)-**39a** (100 mg, 0.20 mmol) in a mixture of THF (4 mL) and isopropanol (4 mL). After stirring the reaction mixture for 5 min at 0 °C, stirring was continued for 36 h at ambient temperature. Then, the solvent was removed in vacuo and the residue was purified by automatic flash column chromatography using a Biotage Isolera One system (10% → 75% ACN in H_2_O, Biotage SNAP Ultra C18 12 g). Fractions containing the desired product were combined and subjected to lyophilization to give (*R*)-**13a** as a colorless solid (70 mg, 0.14 mmol, 69%). mp 86 °C; [α]_D_^20^ = +44.7 (1.7, methanol); HPLC (method 2): *t*_R_ = 13.0 min, purity 99.6%.

##### Spectroscopic Data of (*S*)-**13a** and (*R*)-**13a**

^1^H NMR (DMSO-*d*_6_): δ [ppm] = 2.30–2.40 (m, 4H, N(C*H*_2_CH_2_)_2_O), 3.45–3.51 (m, 3H, NC*H*_2_Ar, OCHC*H*_2_NH (1H)), 3.54–3.60 (m, 5H, N(CH_2_C*H*_2_)_2_O, OCHC*H*_2_NH (1H)), 4.01 (dd, *J* = 7.9/4.8 Hz, 1H, OC*H*CH_2_NH), 4.43 (d, *J* = 12.6 Hz, 1H, OC*H*_2_Ar), 4.61 (d, *J* = 12.6 Hz, 1H, OC*H*_2_Ar), 7.34–7.40 (m, 4H, 2′-H_4-{[4-(morpholinomethyl)phenyl]ethynyl}phenyl_, 6′-H_4-{[4-(morpholinomethyl)phenyl]ethynyl}phenyl_, 3″-H_4-(morpholinomethyl)phenyl_, 5″-H_4-(morpholinomethyl)phenyl_), 7.40–7.44 (m, 2H, 3′-H_4-{[4-(morpholinomethyl)phenyl]ethynyl}phenyl_, 5′-H_4-{[4-(morpholinomethyl)phenyl]ethynyl}phenyl_), 7.44–7.49 (m, 2H, 3‴-H_benzoyl_, 5‴-H_benzoyl_), 7.49–7.51 (m, 2H, 2″-H_4-(morpholinomethyl)phenyl_, 6″-H_4-(morpholinomethyl)phenyl_), 7.51–7.55 (m, 1H, 4‴-H_benzoyl_), 7.81–7.85 (m, 2H, 2‴-H_benzoyl_, 6‴-H_benzoyl_), 8.60 (t, *J* = 5.7 Hz, 1H, CON*H*CH_2_), 8.98 (s br, 1H, CON*H*OH), and 10.89 (s br, 1H, CON*H*OH); ^13^C NMR (DMSO-*d*_6_): δ [ppm] = 41.7 (1C, OCH*C*H_2_NH), 53.2 (2C, N(*C*H_2_CH_2_)_2_O), 62.0 (1C, Ar*C*H_2_N), 66.2 (2C, N(CH_2_*C*H_2_)_2_O), 70.6 (1C, O*C*H_2_Ar), 76.7 (1C, O*C*HCH_2_NH), 89.0 (1C, C≡C), 89.3 (1C, C≡C), 120.8 (1C, C-1″_4-(morpholinomethyl)phenyl_), 121.4 (1C, C-4′_4-{[4-(morpholinomethyl)phenyl]ethynyl}phenyl_), 127.2 (2C, C-2‴_benzoyl_, C-6‴_benzoyl_), 127.7 (2C, C-2′_4-{[4-(morpholinomethyl)phenyl]ethynyl}phenyl_, C-6′_4-{[4-(morpholinomethyl)phenyl]ethynyl}phenyl_), 128.2 (2C, C-3‴_benzoyl_, C-5‴_benzoyl_), 129.2 (2C, C-3″_4-(morpholinomethyl)phenyl_, C-5″_4-(morpholinomethyl)phenyl_), 131.10 (2C, C-3′_4-{[4-(morpholinomethyl)phenyl]ethynyl}phenyl_, C-5′_4-{[4-(morpholinomethyl)phenyl]ethynyl}phenyl_), 131.18 (1C, C-4‴_benzoyl_), 131.22 (2C, C-2″_4-(morpholinomethyl)phenyl_, C-6″_4-(morpholinomethyl)phenyl_), 134.3 (1C, C-1‴_benzoyl_), 138.7 (1C, C-1′_4-{[4-(morpholinomethyl)phenyl]ethynyl}phenyl_), 138.8 (1C, C-4″_4-(morpholinomethyl)phenyl_), 166.2 (1C, *C*ONHOH), and 166.5 (1C, *C*ONHCH_2_); IR (neat): ν̃ [cm^–1^] = 3245, 2860, 2807, 1644, 1529, 1292, 1113, 1006, 864, 692, and 541; HRMS (*m*/*z*): [M + H]^+^ calcd for C_30_H_32_N_3_O_5_, 514.2336; found, 514.2333.

#### NMR Experiments

Standard 1D and STD NMR spectra were acquired at 20 °C with a Bruker 600 MHz NMR spectrometer equipped with a 5 mm cryoprobe. Parameters for the STD experiments (saturation frequency and saturation time) were identical for all samples. Selective saturation of the protein NMR spectrum was achieved with the decoupler offset at 0.5 ppm, and nonsaturation control was performed at 15,000 Hz downfield. STD saturation time was 2 s. WaterLOGSY mixing time was 1.5 s. Two NOESY experiments were recorded with mixing times of 0.6 and 0.3 s. STD and WaterLOGSY spectra were recorded with the same NMR tubes containing 3 μM LpxC, compound **9** at 500 μM, and fragments at 500 μM. For NOESY experiments, NMR tubes contained 6 μM LpxC.

Temperature was set to 293 K for all NMR experiments. Water suppression was achieved with the excitation sculpting sequence in all experiments.

STD signals were measured for protons in the aromatic region only. The STD effects were measured as the ratio between the intensities of the STD signal and the 1D signal (*I*_STD_/*I*_1D_). STD effects were then normalized by setting the largest STD effect to 100%.

### Biological Evaluation

#### Disk Diffusion assay

The disc diffusion assays against *E. coli* BL21(DE3) and the defective strain *E. coli* D22 were performed as follows: liquid cultures of the bacteria were grown overnight in lysogeny broth (LB)^73^ at 37 °C and 200 rpm. 150 μL of an overnight cell suspension was spread evenly onto LB agar plates. 0.15 μmol of each compound (dissolved in 10 or 15 μL DMSO) were applied onto circular filter paper (Ø = 6 mm, Cytiva). Pure DMSO, serving as a negative control, and CHIR-090, serving as a positive control, were also spotted. The agar plates were incubated overnight at 37 °C, and the diameter of the zone of growth inhibition was measured for each compound. Each assay was performed at least three times on separate days.

The disc diffusion assays against *E. coli* TOP10 (Thermo Fisher, Waltham, USA), *E. coli* ATCC 35218, *K. pneumoniae* ATCC 700603, and *P. aeruginosa* ATCC 27853 were performed as follows: the bacteria were grown overnight on a Columbia blood agar plate (Oxoid, Basingstoke, UK), and one colony was suspended in sterile saline to yield a suspension of 0.5 McFarland standard. Using a sterile swab, the suspension was spread evenly onto Mueller–Hinton agar (Oxoid, Basingstoke, UK). 0.15 μmol of each compound (dissolved in 10 μL DMSO) was applied onto circular 6 mm diameter filter paper disks, which were then placed on the agar. After incubating the agar plates for 20 h at 37 °C, the diameter of the zone of growth inhibition was measured for each compound.

#### Minimum Inhibitory Concentration (MIC)

The MIC values of the compounds were determined by means of the microdilution method using 96-well plates.

To determine the MIC values against *E. coli* BL21(DE3) and *E. coli* D22, the bacteria were grown overnight in LB at 37 °C and 200 rpm. The overnight suspension was diluted 1:1000 in fresh LB. 10 μL of a 2-fold dilution series of the compounds in DMSO and 90 μL of LB were dispensed to each well of a 96-well plate. Then, 100 μL of the inoculated medium was added, resulting in 5 × 10^5^ cfu mL^–1^, 5% DMSO, and a final concentration range of the test compounds between 64 and 0.016 μg mL^–1^. The plates were incubated for 20 h at 37 °C. The MIC was defined as the lowest concentration of the compounds that prevented visible growth after incubation. Each assay was performed at least three times on separate days.

### LpxC Enzyme Assays

#### Protein Expression

##### *E. coli* LpxC C63A

The expression of *E. coli* LpxC C63A was performed essentially as previously described.^74^ The C63A mutation lowers the undesired influence of the Zn^2+^-concentration on the enzymatic activity.^50^

The plasmid pET11EcLpxCC63A, which was kindly provided by Carol Fierke,^50^ was transformed into *E. coli* BL21(DE3) cells. The overnight culture was prepared by growing a single colony in 50 mL of LB supplemented with carbenicillin (0.1 mM) and glucose (0.5%) at 37 °C and 200 rpm. The next day, 2 mL of this culture was used to inoculate 400 mL of fresh LB containing carbenicillin (0.1 mM) and glucose (0.5%). After reaching an OD_600_ of 0.6–0.8, the culture was cooled to 30 °C and induced with isopropyl β-d-1-thiogalactopyranoside (IPTG, 1 mM) and ZnCl_2_ (100 μM). After being grown for an additional 4 h at 30 °C, the cells were cooled on ice for 20 min and then harvested by centrifugation (4 °C, 5000*g*, 15 min) and stored at −20 °C.

##### *P. aeruginosa* LpxC

The expression of *P. aeruginosa* LpxC was based on the protocol for the expression of *E. coli* LpxC C63A. The plasmid pWY427, which was kindly provided by Ning Gao,^20^ was transformed into *E. coli* BL21(DE3) cells. The overnight culture was prepared by growing a single colony in 50 mL of LB supplemented with kanamycin (0.1 mM) and glucose (0.5%) at 37 °C and 200 rpm. The next day, 2 mL of this culture was used to inoculate 400 mL of fresh LB containing kanamycin (0.1 mM) and glucose (0.5%). After reaching an OD_600_ of 0.3–0.4, the culture was induced with IPTG (500 μM) and ZnCl_2_ (100 μM). After being grown for an additional 2 h at 37 °C, the cells were cooled on ice for 20 min and then harvested by centrifugation (4 °C, 5000*g*, 15 min) and stored at −20 °C.

#### Protein Purification

Unless otherwise specified, all steps were carried out at 4 °C.

##### *E. coli* LpxC C63A

The harvested cells were thawed on ice and resuspended in 50 mL of anion exchange (AEX) buffer [25 mM HEPES (pH = 7.0), 2 mM dithiothreitol (DTT)], containing benzamidine (15 μg mL^–1^) and phenylmethylsulfonyl fluoride (PMSF, 1 mM) as protease inhibitors. Afterward, the cells were disrupted by sonication (5 × 40 s). Then, the cellular debris were removed by centrifugation (4 °C, 5000*g*, 90 min), and the supernatant was filtered (0.2 μm).

The cleared lysate was loaded onto a 20 mL AEX column (HiPrep Q HP 16/10, GE Healthcare) and eluted at a flow rate of 0.5 mL min^–1^ using a linear potassium chloride gradient (0 M → 0.5 M) in AEX-buffer. The fractions containing LpxC were concentrated using molecular weight cut off (MWCO) spin columns (10 kDa), loaded onto a 120 mL size exclusion (SEC) column (HiLoad 16/600 Superdex 200, GE Healthcare) and eluted at a flow rate of 0.5 mL min^–1^ in SEC buffer [50 mM Bis/Tris (pH = 6.0), 150 mM NaCl].

##### *P. aeruginosa* LpxC

The harvested cells were thawed on ice and resuspended in 50 mL of AEX buffer [25 mM Tris–HCl (pH = 8.0), 2 mM DTT, 5% glycerol] containing benzamidine (15 μg mL^–1^) and PMSF (1 mM) as protease inhibitors. Afterward, the cells were disrupted by sonication (5× 40 s). Then, cellular debris were removed by centrifugation (4 °C, 5000*g*, 90 min), and the supernatant was filtered (0.2 μm).

The cleared lysate was loaded onto a 20 mL AEX column (HiPrep Q HP 16/10, GE Healthcare) and eluted at a flow rate of 0.5 mL min^–1^ using a linear sodium chloride gradient (0 M → 0.5 M) in AEX buffer. The fractions containing LpxC were concentrated using MWCO spin columns (10 kDa), loaded onto a 120 mL SEC column (HiLoad 16/600 Superdex 200, GE Healthcare) and eluted at a flow rate of 0.5 mL min^–1^ in SEC buffer [25 mM HEPES (pH = 8.0), 2 mM DTT, 5% glycerol].

The presence of the enzyme during the purification process was confirmed by sodium dodecyl sulfate-polyacrylamide gel electrophoresis (SDS-PAGE) with Coomassie brilliant blue staining. The purified enzyme was quantified by use of a Nanodrop 2000C, diluted with SEC buffer to 0.5 mg mL^–1^ and stored at −80 °C.

#### Enzyme Inhibition Assays

##### *E. coli* LpxC C63A

A fluorescence-based microplate assay for LpxC activity was performed as described by Clements et al.^48^ The wells in a black, nonbinding, 96-well fluorescence microplate (Greiner Bio One, Frickenhausen) were filled with 93 μL of 26.9 μM UDP-3-*O*-[(*R*)-3-hydroxymyristoyl]-*N*-acetylglucosamine in assay buffer [40 mM sodium morpholinoethanesulfonic acid (pH 6.0), 80 μM dithiothreitol, 0.02% Brij 35]. In order to assay the inhibitors at final concentrations from 20 nM up to 20 μM, 2 μL of a respective dilution of the compounds in DMSO were added. The addition of 5 μL of a solution of purified LpxC (10 μg mL^–1^) in assay buffer led to final concentrations of 25 μM UDP-3-*O*-[(*R*)-3-hydroxymyristoyl]-*N*-acetylglucosamine, 15 nM *E. coli* LpxC C63A, 2% DMSO, and from 20 nM up to 20 μM inhibitor. The microplate was incubated for 30 min at 37 °C in a plate shaker. Then, the biochemical reaction was stopped by adding 40 μL of 0.625 M sodium hydroxide. The reaction mixture was further incubated for 10 min and neutralized by adding 40 μL of 0.625 M acetic acid. The deacetylated product UDP-3-*O*-[(*R*)-3-hydroxymyristoyl]glucosamine was converted into a fluorescing isoindole by adding 120 μL of an *o*-phthaldialdehyde-2-mercaptoethanol solution, which was prepared by dissolving 10 mg of *o*-phthaldialdehyde in 1 mL of methanol, diluting the mixture with 24 mL of a sodium borate buffer (0.1 M), and finally adding 2.5 μL of 2-mercaptoethanol.^49^ Fluorescence was measured with a TriStar^2^ S LB 942 plate reader (Berthold, Bad Wildbad) at 340 nm excitation and 460 nm emission wavelengths. Each assay was performed at least three times on separate days. The IC_50_ values were calculated via Probit-log concentration graphs with the aid of the software Origin and were subsequently converted into *K*_*i*_ values using the Cheng–Prusoff equation.^75,76^ The *K*_M_ value of *E. coli* LpxC C63A was determined experimentally using the LC–MS/MS-based LpxC assay (Supporting Information) and was found to be 3.6 μM (Figure S8).

##### *P. aeruginosa* LpxC

The protocol of the LC–MS/MS-based *P. aeruginosa* LpxC assay was based on the *E. coli* LpxC C63A enzyme assay. Compared to the fluorescence-based enzyme assay, the substrate was diluted 1:10 and the inhibitors were diluted 1:4.

The wells in a black, nonbinding, 96-well fluorescence microplate (Greiner Bio One, Frickenhausen) were filled with 93 μL of 2.69 μM UDP-3-*O*-[(*R*)-3-hydroxymyristoyl]-*N*-acetylglucosamine in assay buffer [50 mM KH_2_PO_4_/K_2_HPO_4_ (pH = 7.5), 80 μM dithiothreitol, 0.02% Brij 35]. In order to assay the inhibitors at final concentrations from 5 nM up to 5 μM, 2 μL of a respective dilution of the compounds in DMSO were added. The addition of 5 μL of a solution of purified *P. aeruginosa* LpxC (5 μg mL^–1^) in assay buffer led to final concentrations of 2.5 μM UDP-3-*O*-[(*R*)-3-hydroxymyristoyl]-*N*-acetylglucosamine, 7.5 nM *P. aeruginosa* LpxC, 2% DMSO, and from 5 nM up to 5 μM inhibitor. The microplate was incubated for 30 min at 37 °C in a plate shaker. Then, the biochemical reaction was stopped by adding 40 μL of 0.625 M hydrochloric acid. The reaction mixtures were further incubated for 10 min, sealed, and stored at −80 °C until analysis.

*LC–MS/MS-Analysis*. The reaction mixtures were separated by ultrahigh performance liquid-chromatography (1290 II Infinity UHPLC, Agilent Technologies), and the eluted compounds were analyzed by mass spectrometry using electrospray ionization in negative ion mode with a triple quadrupole linear ion trap mass spectrometer (QTRAP 5500, AB Sciex LLC).

UHPLC method: column: Nucleodur C18 Gravity-SB (Ø = 3 mm, *h* = 100 mm, Macherey-Nagel), coupled to a Universal RP-guard column (Ø = 2 mm, *h* = 4 mm, Macherey-Nagel); flow rate: 0.3 mL · min^–1^; injection volume: 3.0 μL; solvents: (A) 20 mM ammonium formate in water; (B) 1 mM ammonium formate in acetonitrile/isopropanol/water (47.5:42.75:9.75); gradient elution: (B %): 0–1 min: 30%, 1–16 min: gradient from 30 to 90%, 16–17 min: 90%, 17–17.5 min: gradient from 90 to 30%, 17.5–21.5 min: 30%; detection: 12–19 min; *t*_R_ (**1**) = 12.2 min, *t*_R_ (**2**) = 13.0 min.

To analyze the eluted compounds by mass spectrometry, a MRM method was applied. The specific parameters of this method are given in Table S2 (Supporting Information). After detection and selection of the precursor ions (**1**: *m*/*z* 832; **2**: *m*/*z* 790), both analytes were fragmented, leading to three identical product ions [*m*/*z* (product 1) 385, collision energy = −60 V; *m*/*z* (product 2) 159, collision energy = −80 V; *m*/*z* (product 3) 79, collision energy = −140 V]. The mass transitions 832 → 79 (substrate **1**) and 790 → 79 (product **2**) were used as quantifiers; the other mass transitions were used as qualifiers. The ratio between substrate **1** and product **2** was quantified by comparing the peak areas of the quantifiers. The percentual inhibition caused by each inhibitor concentration was determined with respect to the amount of the product formed in the noninhibited reaction after 30 min.

Each assay was performed at least two times on separate days. The IC_50_ values were calculated via Probit-log concentration graphs with the aid of the software Origin and were subsequently converted into *K*_*i*_ values using the Cheng–Prusoff equation.^75,76^ The *K*_M_ value for the *P. aeruginosa* LpxC-catalyzed deacetylation of **1** was determined experimentally (Supporting Information) and found to be 4.7 μM (Figure S9).

### Assays to Determine the In Vitro Inhibition of LasB, MMPs, and TACE

Purification of LasB from *P. aeruginosa* PA14 supernatant and the subsequent performance of the FRET-based in vitro inhibition assay was performed as described previously.^66^ The TACE (ADAM-17) inhibitor screening kit was purchased from Sigma-Aldrich (Saint Louis, MO). MMPs 1–3 along with the SensoLyte 520 Generic MMP Activity Kit Fluorimetric were purchased from AnaSpec (Fremont, CA, USA). The assays were performed according to the guidelines of the respective manufacturer. Fluorescence signals were measured using a CLARIOstar plate reader (BMG Labtech, Ortenberg, Germany).

### Cytotoxicity Assay

An MTT-based assay was employed to evaluate the viability of HepG2 cells after challenge with selected inhibitors and performed as described previously.^77^

### Kinetic Turbidimetric Solubility

The desired compounds were sequentially diluted in DMSO in a 96-well plate. 1.5 μL from each well was transferred into another 96-well plate and mixed with 148.5 μL of PBS. Plates were shaken for 5 min at 600 rpm at room temperature, and the absorbance at 620 nm was measured. Absorbance values were normalized by blank subtraction and plotted using GraphPad Prism 8.4.2 (GraphPad Software, San Diego, CA, USA). Solubility (*S*) was determined based on the First X value of AUC function using a threshold of 0.005.

### Log *D*_7.4_

Log *D*_7.4_ was analyzed using an HPLC-based method. The UV retention time of reference compounds with known log *D*_7.4_ was determined and plotted toward their log *D*_7.4_. Linear regression was used to determine the log *D*_7.4_ of unknown compounds. Analysis was performed using a Vanquish Flex HPLC system with a variable wavelength detector (Thermo Fisher, Dreieich, Germany) with the following conditions: EC150/2 NUCLEODUR C18 Pyramid column, 5 μM (Macherey Nagel, Düren, Germany); eluent A: 50 mM NH_4_OAc pH 7.4, eluent B: acetonitrile, and flow: 0.6 mL/min; gradient elution: (B %): 0–2.5 min: gradient from 0 to 100%, 2.5–3.0 min: 100%, 3.0–3.2 min: gradient from 100 to 0%, 3.2–5.0 min: 0%.

### ADME In Vitro Studies

The microsomal metabolic stability assay as well as the plasma protein binding assay were conducted as described previously.^78^

#### HPLC–MS/MS Analysis

Samples were analyzed using an Agilent 1290 Infinity II HPLC system coupled to an AB Sciex QTrap 6500plus mass spectrometer. LC conditions were as follows: column: Agilent Zorbax Eclipse Plus C18, 50 × 2.1 mm, 1.8 μm; temperature: 30 °C; injection volume: 5 μL per sample; flow rate: 700 μL min^–1^. Samples were run under acidic conditions. Solvents: (A) water + 0.1% formic acid; (B) 95% acetonitrile/5% H_2_O + 0.1% formic acid. Gradient elution: (A %): 0–0.1 min: 99%, 0.1–3.5 min: gradient from 99 to 50%, 3.5–3.8 min: gradient from 50 to 0%, 3.8–4.7 min: gradient from 0 to 99%. Mass transitions for controls and compounds are depicted in Table S3.

### Metabolic Stability in Liver S9 Fractions

For the evaluation of combined phase I and phase II metabolic stability, the compound (1 μM) was incubated with 1 mg/mL pooled mouse liver S9 fraction (Xenotech, Kansas City, USA), 2 mM nicotinamide adenine dinucleotide phosphate hydrogen (NADPH), 1 mM UDPGA, 10 mM MgCl_2_, 5 mM glutathione (GSH), and 0.1 mM 3′-phosphoadenosine **5**′-phosphosulfate (PAPS) at 37 °C for 120 min. The metabolic stability of testosterone, verapamil, and ketoconazole were determined in parallel to confirm the enzymatic activity of mouse S9 fractions. The incubation was stopped after defined time points by precipitation of aliquots of S9 enzymes with 2 volumes of cold acetonitrile containing internal standard (150 nM diphenhydramine). Samples were stored on ice until the end of the incubation and the precipitated protein was removed by centrifugation (15 min, 4 °C, 4000*g*). Concentration of the remaining test compound at the different time points was analyzed by HPLC–MS/MS (TSQ Quantum Access Max, Thermo Fisher, Dreieich, Germany) and used to determine half-life (*t*_1/2_).

### Stability in Mouse Plasma

To determine stability in mouse plasma, the compound (1 μM) was incubated with pooled CD-1 mouse plasma (Neo Biotech, Nanterre, France). Samples were taken at defined time points by mixing aliquots with 4 volumes of acetonitrile containing internal standard (125 nM diphenhydramine). Samples were stored on ice until the end of the incubation, and the precipitated protein was removed by centrifugation (15 min, 4 °C, 4000*g*, 2 centrifugation steps). Concentration of the remaining test compound at the different time points was analyzed by HPLC–MS/MS (TSQ Quantum Access MAX, Thermo Fisher, Dreieich, Germany). The plasma stability of procain, propantheline, and diltiazem were determined in parallel to confirm enzymatic activity.

### Computational Methods

Molecular docking was performed using a recently developed and evaluated protocol as reported in our previous studies.^32,79^ This docking protocol was successful in redocking the cocrystallized inhibitors. The crystal structure of *E. coli* LpxC (PDB ID: 4MQY) in complex with LPC-138 (Figure S1) was retrieved from the Protein Data Bank (https://www.rcsb.org).^80^ The LpxC protein structure was chosen due to the similarity of LPC-138 to the compounds developed in the current work. The LpxC protein was prepared using Protein Preparation Wizard by adding the hydrogen atoms and missing side chains in the Schrödinger suite.^81^ Solvent molecules were removed. Tautomeric states and protonation states of the amino acids were adjusted with the PROPKA tool at pH 7.0. OPLS3e force field was applied to minimize the complex to remove the steric clashes, bad contacts, and unsuitable torsional angles. The inhibitor structures were prepared using the Ligprep tool by applying the OPLS3e force field. Subsequently, 64 conformers per ligand were generated using the Confgen tool with force field minimization on output conformers. Molecular docking studies were performed in Glide from the Schrödinger suite. Grid files were prepared with default settings by applying box-size as 15 Å × 15 Å × 15 Å. Standard Precision mode with flexible ligand sampling and enhanced planarity of conjugated π groups were used for docking studies. The validation of the docking protocol was done by redocking. The root-mean-square deviation (RMSD) value of the redocked ligand from 4MQY compared to its observed binding mode in the crystal structure was 0.78 Å. The docking results were visually analyzed in MOE.^82^

#### MD Simulation

The selected docking poses in complex with LpxC as well as the original crystal structure cocrystallized with the inhibitor LPC-138 (PDB ID: 4MQY) were subjected to MD simulation using Desmond (Schrödinger Suite 2019).^82,83^ LpxC–inhibitor complexes were simulated for two independent runs each of 50 ns. The System Builder panel was used to build the complexes. The complexes were solvated using a SPC water model and an orthorhombic box with 10 Å distance between the solute structures and the simulation box boundary. The box volume was then minimized. The whole system was neutralized by adding sodium ions that were placed 10 Å away from the inhibitor structure. The MD panel in Desmond was used to set the simulation parameters. The prepared system was relaxed using the default Desmond relaxation protocol for *NPT* ensemble followed by a production run utilizing the *NPT* ensemble at the temperature of 300 K using a Nosé–Hoover chain thermostat and a pressure of 1.01325 bar using a Martyna–Tobias–Klein barostat. The progress of the simulation was recorded every 100 ps. The Simulation Interaction Diagram panel was used for analyzing the RMSD, RMSF, and the interaction persistence of the ligands. The Simulation Event Analysis panel was used for calculating protein–inhibitor distances and hydrogen bond occupancies.

## Supporting Information Available

Molecular formula strings (CSV)
Determination of enzymatic parameters, synthetic procedures and analytical data of compounds (*S*)-**38b**–**n**, (*R*)-**38b**–**h**,**j**,**m**,**n**, (*S*)-**39b**–**n**, (*R*)-**39**–**h**,**j**,**m**,**n**, (*S*)-**13b**–**n**, and (*R*)-**13b**–**h**,**j**,**m**,**n**, ^1^H and ^13^C NMR spectra of representative compounds, and HPLC chromatograms of the test compounds (PDF)

## Abbreviations

ILOE: interligand nuclear Overhauser effect
LB: lysogeny broth
MRM: multiple reaction monitoring
RMSF: root-mean-square fluctuation
STD: saturation-transfer difference
TACE: tumor necrosis factor-α converting enzyme
UDPGA: uridine diphosphate glucuronic acid
WaterLOGSY: Water-Ligand Observed via Gradient SpectroscopY

## References

[ref1] Walsh C. T.; Wencewicz T. A. Prospects for new antibiotics: a molecule-centered perspective. J. Antibiot. 2014, 67 (1), 7–22. 10.1038/ja.2013.49. 23756684

[ref2] Miethke M.; Pieroni M.; Weber T.; Brönstrup M.; Hammann P.; Halby L.; Arimondo P. B.; Glaser P.; Aigle B.; Bode H. B.; Moreira R.; Li Y.; Luzhetskyy A.; Medema M. H.; Pernodet J. L.; Stadler M.; Tormo J. R.; Genilloud O.; Truman A. W.; Weissman K. J.; Takano E.; Sabatini S.; Stegmann E.; Brötz-Oesterhelt H.; Wohlleben W.; Seemann M.; Empting M.; Hirsch A. K. H.; Loretz B.; Lehr C. M.; Titz A.; Herrmann J.; Jaeger T.; Alt S.; Hesterkamp T.; Winterhalter M.; Schiefer A.; Pfarr K.; Hoerauf A.; Graz H.; Graz M.; Lindvall M.; Ramurthy S.; Karlen A.; van Dongen M.; Petkovic H.; Keller A.; Peyrane F.; Donadio S.; Fraisse L.; Piddock L. J. V.; Gilbert I. H.; Moser H. E.; Müller R. Towards the sustainable discovery and development of new antibiotics. Nat. Rev. Chem. 2021, 5 (10), 726–749. 10.1038/s41570-021-00313-1. PMC8374425 34426795

[ref3] Hegemann J. D.; Birkelbach J.; Walesch S.; Müller R. Current developments in antibiotic discovery: Global microbial diversity as a source for evolutionary optimized anti-bacterials. EMBO Rep. 2023, 24 (1), e56184 10.15252/embr.202256184. 36541849 PMC9827545

[ref4] Murray C. J. L.; Ikuta K. S.; Sharara F.; Swetschinski L.; Robles Aguilar G.; Gray A.; Han C.; Bisignano C.; Rao P.; Wool E.; et al. Global burden of bacterial antimicrobial resistance in 2019: a systematic analysis. Lancet 2022, 399 (10325), 629–655. 10.1016/S0140-6736(21)02724-0. 35065702 PMC8841637

[ref5] Ikuta K. S.; Swetschinski L. R.; Robles Aguilar G.; Sharara F.; Mestrovic T.; Gray A. P.; Davis Weaver N.; Wool E. E.; Han C.; Gershberg Hayoon A.; et al. Global mortality associated with 33 bacterial pathogens in 2019: a systematic analysis for the Global Burden of Disease Study 2019. Lancet 2022, 400 (10369), 2221–2248. 10.1016/S0140-6736(22)02185-7. 36423648 PMC9763654

[ref6] de Kraker M. E.; Stewardson A. J.; Harbarth S. Will 10 Million People Die a Year due to Antimicrobial Resistance by 2050?. PLoS Med. 2016, 13 (11), e1002184 10.1371/journal.pmed.1002184. 27898664 PMC5127510

[ref7] Newman D. J.; Cragg G. M. Natural Products as Sources of New Drugs over the Nearly Four Decades from 01/1981 to 09/2019. J. Nat. Prod. 2020, 83 (3), 770–803. 10.1021/acs.jnatprod.9b01285. 32162523

[ref8] Spry C.; Coyne A. G. Fragment-Based Discovery of Antibacterials. In Fragment-Based Drug Discovery; Steven H., Chris A., Eds.; Royal Society of Chemistry, 2015; pp 177–213.

[ref9] Wyckoff T. J. O.; Raetz C. R. H.; Jackman J. E. Antibacterial and anti-inflammatory agents that target endotoxin. Trends Microbiol. 1998, 6 (4), 154–159. 10.1016/S0966-842X(98)01230-X. 9587193

[ref10] Raetz C. R. H.; Reynolds C. M.; Trent M. S.; Bishop R. E. Lipid a modification systems in gram-negative bacteria. Annu. Rev. Biochem. 2007, 76, 295–329. 10.1146/annurev.biochem.76.010307.145803. 17362200 PMC2569861

[ref11] Zhou P.; Barb A. Mechanism and inhibition of LpxC: an essential zinc-dependent deacetylase of bacterial lipid A synthesis. Curr. Pharm. Biotechnol. 2008, 9 (1), 9–15. 10.2174/138920108783497668. 18289052 PMC3022321

[ref12] Raetz C. R. H.; Whitfield C. Lipopolysaccharide endotoxins. Annu. Rev. Biochem. 2002, 71, 635–700. 10.1146/annurev.biochem.71.110601.135414. 12045108 PMC2569852

[ref13] Clayton G. M.; Klein D. J.; Rickert K. W.; Patel S. B.; Kornienko M.; Zugay-Murphy J.; Reid J. C.; Tummala S.; Sharma S.; Singh S. B.; Miesel L.; Lumb K. J.; Soisson S. M. Structure of the bacterial deacetylase LpxC bound to the nucleotide reaction product reveals mechanisms of oxyanion stabilization and proton transfer. J. Biol. Chem. 2013, 288 (47), 34073–34080. 10.1074/jbc.M113.513028. 24108127 PMC3837148

[ref14] Whittington D. A.; Rusche K. M.; Shin H.; Fierke C. A.; Christianson D. W. Crystal structure of LpxC, a zinc-dependent deacetylase essential for endotoxin biosynthesis. Proc. Natl. Acad. Sci. U.S.A. 2003, 100 (14), 8146–8150. 10.1073/pnas.1432990100. 12819349 PMC166197

[ref15] Clayton G. M.; Klein D. J.; Rickert K. W.; Patel S. B.; Kornienko M.; Zugay-Murphy J.; Reid J. C.; Tummala S.; Sharma S.; Singh S. B.; Miesel L.; Lumb K. J.; Soisson S. M. Structure of the Bacterial Deacetylase LpxC Bound to the Nucleotide Reaction Product Reveals Mechanisms of Oxyanion Stabilization and Proton Transfer. J. Biol. Chem. 2013, 288 (47), 34073–34080. 10.1074/jbc.M113.513028. 24108127 PMC3837148

[ref16] Barb A. W.; Jiang L.; Raetz C. R.; Zhou P. Structure of the deacetylase LpxC bound to the antibiotic CHIR-090: Time-dependent inhibition and specificity in ligand binding. Proc. Natl. Acad. Sci. U.S.A. 2007, 104 (47), 18433–18438. 10.1073/pnas.0709412104. 18025458 PMC2141794

[ref17] Barb A. W.; Leavy T. M.; Robins L. I.; Guan Z.; Six D. A.; Zhou P.; Bertozzi C. R.; Raetz C. R. H.; Raetz C. R. Uridine-based inhibitors as new leads for antibiotics targeting Escherichia coli LpxC. Biochemistry 2009, 48 (14), 3068–3077. 10.1021/bi900167q. 19256534 PMC2709817

[ref18] Liang X.; Lee C. J.; Zhao J.; Toone E. J.; Zhou P. Synthesis, structure, and antibiotic activity of aryl-substituted LpxC inhibitors. J. Med. Chem. 2013, 56 (17), 6954–6966. 10.1021/jm4007774. 23914798 PMC3941642

[ref19] Warmus J. S.; Quinn C. L.; Taylor C.; Murphy S. T.; Johnson T. A.; Limberakis C.; Ortwine D.; Bronstein J.; Pagano P.; Knafels J. D.; Lightle S.; Mochalkin I.; Brideau R.; Podoll T. Structure based design of an in vivo active hydroxamic acid inhibitor of P. aeruginosa LpxC. Bioorg. Med. Chem. Lett. 2012, 22 (7), 2536–2543. 10.1016/j.bmcl.2012.01.140. 22401863

[ref20] Hale M. R.; Hill P.; Lahiri S.; Miller M. D.; Ross P.; Alm R.; Gao N.; Kutschke A.; Johnstone M.; Prince B.; Thresher J.; Yang W. Exploring the UDP pocket of LpxC through amino acid analogs. Bioorg. Med. Chem. Lett. 2013, 23 (8), 2362–2367. 10.1016/j.bmcl.2013.02.055. 23499237

[ref21] Piizzi G.; Parker D. T.; Peng Y.; Dobler M.; Patnaik A.; Wattanasin S.; Liu E.; Lenoir F.; Nunez J.; Kerrigan J.; McKenney D.; Osborne C.; Yu D.; Lanieri L.; Bojkovic J.; Dzink-Fox J.; Lilly M. D.; Sprague E. R.; Lu Y.; Wang H.; Ranjitkar S.; Xie L.; Wang B.; Glick M.; Hamann L. G.; Tommasi R.; Yang X.; Dean C. R. Design, Synthesis, and Properties of a Potent Inhibitor of Pseudomonas aeruginosa Deacetylase LpxC. J. Med. Chem. 2017, 60 (12), 5002–5014. 10.1021/acs.jmedchem.7b00377. 28549219

[ref22] Kalinin D. V.; Holl R. Insights into the Zinc-Dependent Deacetylase LpxC: Biochemical Properties and Inhibitor Design. Curr. Top. Med. Chem. 2016, 16 (21), 2379–2430. 10.2174/1568026616666160413135835. 27072691

[ref23] Kalinin D. V.; Holl R. LpxC inhibitors: a patent review (2010–2016). Expert Opin. Ther. Pat. 2017, 27 (11), 1227–1250. 10.1080/13543776.2017.1360282. 28742403

[ref24] Cohen F.; Aggen J. B.; Andrews L. D.; Assar Z.; Boggs J.; Choi T.; Dozzo P.; Easterday A. N.; Haglund C. M.; Hildebrandt D. J.; Holt M. C.; Joly K.; Jubb A.; Kamal Z.; Kane T. R.; Konradi A. W.; Krause K. M.; Linsell M. S.; Machajewski T. D.; Miroshnikova O.; Moser H. E.; Nieto V.; Phan T.; Plato C.; Serio A. W.; Seroogy J.; Shakhmin A.; Stein A. J.; Sun A. D.; Sviridov S.; Wang Z.; Wlasichuk K.; Yang W.; Zhou X.; Zhu H.; Cirz R. T. Optimization of LpxC Inhibitors for Antibacterial Activity and Cardiovascular Safety. ChemMedChem 2019, 14 (16), 1560–1572. 10.1002/cmdc.201900287. 31283109

[ref25] Liang X.; Lee C. J.; Chen X.; Chung H. S.; Zeng D.; Raetz C. R.; Li Y.; Zhou P.; Toone E. J. Syntheses, structures and antibiotic activities of LpxC inhibitors based on the diacetylene scaffold. Bioorg. Med. Chem. 2011, 19 (2), 852–860. 10.1016/j.bmc.2010.12.017. 21194954 PMC3035996

[ref26] Niu Z.; Lei P.; Wang Y.; Wang J.; Yang J.; Zhang J. Small molecule LpxC inhibitors against gram-negative bacteria: Advances and future perspectives. Eur. J. Med. Chem. 2023, 253, 115326 10.1016/j.ejmech.2023.115326. 37023679

[ref27] Kumar Pal S.; Kumar S. LpxC (UDP-3-O-(R-3-hydroxymyristoyl)-*N*-acetylglucosamine deacetylase) inhibitors: A long path explored for potent drug design. Int. J. Biol. Macromol. 2023, 234, 122960 10.1016/j.ijbiomac.2022.12.179. 36565833

[ref28] Lee C. J.; Liang X.; Chen X.; Zeng D.; Joo S. H.; Chung H. S.; Barb A. W.; Swanson S. M.; Nicholas R. A.; Li Y.; Toone E. J.; Raetz C. R.; Zhou P. Species-specific and inhibitor-dependent conformations of LpxC: implications for antibiotic design. Chem. Biol. 2011, 18 (1), 38–47. 10.1016/j.chembiol.2010.11.011. 21167751 PMC3149848

[ref29] Yamada Y.; Takashima H.; Walmsley D. L.; Ushiyama F.; Matsuda Y.; Kanazawa H.; Yamaguchi-Sasaki T.; Tanaka-Yamamoto N.; Yamagishi J.; Kurimoto-Tsuruta R.; Ogata Y.; Ohtake N.; Angove H.; Baker L.; Harris R.; Macias A.; Robertson A.; Surgenor A.; Watanabe H.; Nakano K.; Mima M.; Iwamoto K.; Okada A.; Takata I.; Hitaka K.; Tanaka A.; Fujita K.; Sugiyama H.; Hubbard R. E. Fragment-Based Discovery of Novel Non-Hydroxamate LpxC Inhibitors with Antibacterial Activity. J. Med. Chem. 2020, 63 (23), 14805–14820. 10.1021/acs.jmedchem.0c01215. 33210531

[ref30] Szermerski M.; Melesina J.; Wichapong K.; Löppenberg M.; Jose J.; Sippl W.; Holl R. Synthesis, biological evaluation and molecular docking studies of benzyloxyacetohydroxamic acids as LpxC inhibitors. Bioorg. Med. Chem. 2014, 22 (3), 1016–1028. 10.1016/j.bmc.2013.12.057. 24412340

[ref31] Tangherlini G.; Torregrossa T.; Agoglitta O.; Köhler J.; Melesina J.; Sippl W.; Holl R. Synthesis and biological evaluation of enantiomerically pure glyceric acid derivatives as LpxC inhibitors. Bioorg. Med. Chem. 2016, 24 (5), 1032–1044. 10.1016/j.bmc.2016.01.029. 26827141

[ref32] Wimmer S.; Hoff K.; Martin B.; Grewer M.; Denni L.; Lascorz Massanet R.; Raimondi M. V.; Bülbül E. F.; Melesina J.; Hotop S. K.; Haupenthal J.; Rohde H.; Heisig P.; Hirsch A. K. H.; Brönstrup M.; Sippl W.; Holl R. Synthesis, biological evaluation, and molecular docking studies of aldotetronic acid-based LpxC inhibitors. Bioorg. Chem. 2023, 131, 106331 10.1016/j.bioorg.2022.106331. 36587505

[ref33] Erlanson D. A. Introduction to fragment-based drug discovery. Top. Curr. Chem. 2011, 317, 1–32. 10.1007/128_2011_180. 21695633

[ref34] Murray C. W.; Erlanson D. A.; Hopkins A. L.; Keseru G. M.; Leeson P. D.; Rees D. C.; Reynolds C. H.; Richmond N. J. Validity of ligand efficiency metrics. ACS Med. Chem. Lett. 2014, 5 (6), 616–618. 10.1021/ml500146d. 24944729 PMC4060940

[ref35] Meyer B.; Peters T. NMR spectroscopy techniques for screening and identifying ligand binding to protein receptors. Angew. Chem., Int. Ed. Engl. 2003, 42 (8), 864–890. 10.1002/anie.200390233. 12596167

[ref36] Dalvit C.; Fogliatto G.; Stewart A.; Veronesi M.; Stockman B. WaterLOGSY as a method for primary NMR screening: practical aspects and range of applicability. J. Biomol. NMR 2001, 21 (4), 349–359. 10.1023/A:1013302231549. 11824754

[ref37] Leone M.; Freeze H. H.; Chan C. S.; Pellecchia M. The Nuclear Overhauser Effect in the lead identification process. Curr. Drug Discovery Technol. 2006, 3 (2), 91–100. 10.2174/157016306778108884. 16925517

[ref38] Nardi M.; Dalpozzo R.; Oliverio M.; Paonessa R.; Procopio A. Erbium(III) Triflate is a Highly Efficient Catalyst for the Synthesis of β-Alkoxy Alcohols, 1,2-Diols and β-Hydroxy Sulfides by Ring Opening of Epoxides. Synthesis 2009, 2009 (20), 3433–3438. 10.1055/s-0029-1216956.

[ref39] Kawai T.; Kazuhiko I.; Takaya N.; Yamaguchi Y.; Kishii R.; Kohno Y.; Kurasaki H. Sulfonamide-based non-alkyne LpxC inhibitors as Gram-negative antibacterial agents. Bioorg. Med. Chem. Lett. 2017, 27 (4), 1045–1049. 10.1016/j.bmcl.2016.12.059. 28082037

[ref40] Kurasaki H.; Tsuda K.; Shinoyama M.; Takaya N.; Yamaguchi Y.; Kishii R.; Iwase K.; Ando N.; Nomura M.; Kohno Y. LpxC Inhibitors: Design, Synthesis, and Biological Evaluation of Oxazolidinones as Gram-negative Antibacterial Agents. ACS Med. Chem. Lett. 2016, 7 (6), 623–628. 10.1021/acsmedchemlett.6b00057. 27326338 PMC4904260

[ref41] Morpain C.; Tisserand M. A Possible Model for a New Chiral Glyceride Synthesis. Part 1. Synthesis of 1-O-Aroyl-2-O-tosyl-sn-glycerols. J. Chem. Soc., Perkin Trans. 1979, 1, 1379–1383. 10.1039/p19790001379.

[ref42] Wiggins L. F. 4. The acetone derivatives of hexahydric alcohols. Part I. Triacetone mannitol and its conversion into d-arabinose. J. Chem. Soc. 1946, 13–14. 10.1039/jr9460000013. 21015764

[ref43] Williams D. R.; Klingler F. D.; Allen E. E.; Lichtenthaler F. W. Bromine as an Oxidant for Direct Conversion of Aldehydes to Esters. Tetrahedron Lett. 1988, 29 (40), 5087–5090. 10.1016/S0040-4039(00)80686-3.

[ref44] Fringuelli F.; Piermatti O.; Pizzo F.; Vaccaro L. Ring Opening of Epoxides with Sodium Azide in Water. A Regioselective pH-Controlled Reaction. J. Org. Chem. 1999, 64 (16), 6094–6096. 10.1021/jo990368i.

[ref45] Gajda T.; Koziara A.; Osowska-Pacewicka K.; Zawadzki S.; Zwierzak A. A Convergent One-Pot Synthesis of Secondary Amines via Aza-Wittig Reaction. Synth. Commun. 1992, 22 (13), 1929–1938. 10.1080/00397919208021323.

[ref46] Abdel-Magid A. F.; Carson K. G.; Harris B. D.; Maryanoff C. A.; Shah R. D. Reductive Amination of Aldehydes and Ketones with Sodium Triacetoxyborohydride. Studies on Direct and Indirect Reductive Amination Procedures. J. Org. Chem. 1996, 61 (11), 3849–3862. 10.1021/jo960057x. 11667239

[ref47] Burés J.; Martín M.; Urpí F.; Vilarrasa J. Catalytic Staudinger-Vilarrasa Reaction for the Direct Ligation of Carboxylic Acids and Azides. J. Org. Chem. 2009, 74 (5), 2203–2206. 10.1021/jo802825e. 19203231

[ref48] Clements J. M.; Coignard F.; Johnson I.; Chandler S.; Palan S.; Waller A.; Wijkmans J.; Hunter M. G. Antibacterial activities and characterization of novel inhibitors of LpxC. Antimicrob. Agents Chemother. 2002, 46 (6), 1793–1799. 10.1128/AAC.46.6.1793-1799.2002. 12019092 PMC127247

[ref49] Roth M. Fluorescence Reaction for Amino Acids. Anal. Chem. 1971, 43 (7), 880–882. 10.1021/ac60302a020. 5576608

[ref50] Hernick M.; Gattis S. G.; Penner-Hahn J. E.; Fierke C. A. Activation of Escherichia coli UDP-3-O-[(R)-3-hydroxymyristoyl]-N-acetylglucosamine Deacetylase by Fe^2+^ Yields a More Efficient Enzyme with Altered Ligand Affinity. Biochemistry 2010, 49 (10), 2246–2255. 10.1021/bi902066t. 20136146 PMC2884013

[ref51] Jackman J. E.; Raetz C. R.; Fierke C. A. UDP-3-*O*-(*R*-3-Hydroxymyristoyl)-*N*-acetylglucosamine Deacetylase of Escherichia coli Is a Zinc Metalloenzyme. Biochemistry 1999, 38 (6), 1902–1911. 10.1021/bi982339s. 10026271

[ref52] Barb A. W.; McClerren A. L.; Snehelatha K.; Reynolds C. M.; Zhou P.; Raetz C. R. Inhibition of Lipid A Biosynthesis as the Primary Mechanism of CHIR-090 Antibiotic Activity in Escherichia coli. Biochemistry 2007, 46 (12), 3793–3802. 10.1021/bi6025165. 17335290 PMC2709454

[ref53] Normark S.; Boman H. G.; Matsson E. Mutant of Escherichia coli with Anomalous Cell Division and Ability to Decrease Episomally and Chromosomally Mediated Resistance to Ampicillin and Several Other Antibiotics. J. Bacteriol. 1969, 97 (3), 1334–1342. 10.1128/jb.97.3.1334-1342.1969. 4887513 PMC249852

[ref54] Cala O.; Krimm I. Ligand-Orientation Based Fragment Selection in STD NMR Screening. J. Med. Chem. 2015, 58 (21), 8739–8742. 10.1021/acs.jmedchem.5b01114. 26492576

[ref55] Mayer M.; Meyer B. Group epitope mapping by saturation transfer difference NMR to identify segments of a ligand in direct contact with a protein receptor. J. Am. Chem. Soc. 2001, 123 (25), 6108–6117. 10.1021/ja0100120. 11414845

[ref56] Rice L. B. Federal funding for the study of antimicrobial resistance in nosocomial pathogens: no ESKAPE. J. Infect. Dis. 2008, 197 (8), 1079–1081. 10.1086/533452. 18419525

[ref57] Tacconelli E.; Carrara E.; Savoldi A.; Harbarth S.; Mendelson M.; Monnet D. L.; Pulcini C.; Kahlmeter G.; Kluytmans J.; Carmeli Y.; Ouellette M.; Outterson K.; Patel J.; Cavaleri M.; Cox E. M.; Houchens C. R.; Grayson M. L.; Hansen P.; Singh N.; Theuretzbacher U.; Magrini N.; et al. Discovery, research, and development of new antibiotics: the WHO priority list of antibiotic-resistant bacteria and tuberculosis. Lancet Infect. Dis. 2018, 18 (3), 318–327. 10.1016/S1473-3099(17)30753-3. 29276051

[ref58] Smith E. W.; Zhang X.; Behzadi C.; Andrews L. D.; Cohen F.; Chen Y. Structures of Pseudomonas aeruginosa LpxA Reveal the Basis for Its Substrate Selectivity. Biochemistry 2015, 54 (38), 5937–5948. 10.1021/acs.biochem.5b00720. 26352800

[ref59] Williamson J. M.; Anderson M. S.; Raetz C. R. Acyl-acyl carrier protein specificity of UDP-GlcNAc acyltransferases from gram-negative bacteria: relationship to lipid A structure. J. Bacteriol. 1991, 173 (11), 3591–3596. 10.1128/jb.173.11.3591-3596.1991. 1904441 PMC207978

[ref60] Coggins B. E.; Li X.; McClerren A. L.; Hindsgaul O.; Raetz C. R.; Zhou P. Structure of the LpxC deacetylase with a bound substrate-analog inhibitor. Nat. Struct. Mol. Biol. 2003, 10 (8), 645–651. 10.1038/nsb948. PMC6783277 12833153

[ref61] Hyland S. A.; Eveland S. S.; Anderson M. S. Cloning, Expression, and Purification of UDP-3-O-acyl-GlcNAc Deacetylase from Pseudomonas aeruginosa: a Metalloamidase of the Lipid A Biosynthesis Pathway. J. Bacteriol. 1997, 179 (6), 2029–2037. 10.1128/jb.179.6.2029-2037.1997. 9068651 PMC178929

[ref62] Lee C. J.; Liang X.; Gopalaswamy R.; Najeeb J.; Ark E. D.; Toone E. J.; Zhou P. Structural Basis of the Promiscuous Inhibitor Susceptibility of Escherichia coli LpxC. ACS Chem. Biol. 2014, 9 (1), 237–246. 10.1021/cb400067g. 24117400 PMC3947053

[ref63] Everett M. J.; Davies D. T. Pseudomonas aeruginosa elastase (LasB) as a therapeutic target. Drug Discov. Today 2021, 26 (9), 2108–2123. 10.1016/j.drudis.2021.02.026. 33676022

[ref64] Horna G.; Ruiz J. Type 3 secretion system of Pseudomonas aeruginosa. Microbiol. Res. 2021, 246, 126719 10.1016/j.micres.2021.126719. 33582609

[ref65] Bastaert F.; Kheir S.; Saint-Criq V.; Villeret B.; Dang P. M.; El-Benna J.; Sirard J. C.; Voulhoux R.; Sallenave J. M. Pseudomonas aeruginosa LasB Subverts Alveolar Macrophage Activity by Interfering With Bacterial Killing Through Downregulation of Innate Immune Defense, Reactive Oxygen Species Generation, and Complement Activation. Front. Immunol. 2018, 9, 1675 10.3389/fimmu.2018.01675. 30083156 PMC6064941

[ref66] Kany A. M.; Sikandar A.; Haupenthal J.; Yahiaoui S.; Maurer C. K.; Proschak E.; Kohnke J.; Hartmann R. W. Binding Mode Characterization and Early in Vivo Evaluation of Fragment-Like Thiols as Inhibitors of the Virulence Factor LasB from Pseudomonas aeruginosa. ACS Infect. Dis. 2018, 4 (6), 988–997. 10.1021/acsinfecdis.8b00010. 29485268

[ref67] Richardson S. J.; Bai A.; Kulkarni A. A.; Moghaddam M. F. Efficiency in Drug Discovery: Liver S9 Fraction Assay As a Screen for Metabolic Stability. Drug Metab. Lett. 2016, 10 (2), 83–90. 10.2174/1872312810666160223121836. 26902079 PMC5405623

[ref68] Wang X.; He B.; Shi J.; Li Q.; Zhu H. J. Comparative Proteomics Analysis of Human Liver Microsomes and S9 Fractions. Drug Metab. Dispos. 2020, 48 (1), 31–40. 10.1124/dmd.119.089235. 31699809 PMC6918043

[ref69] O’Shea R.; Moser H. E. Physicochemical properties of antibacterial compounds: implications for drug discovery. J. Med. Chem. 2008, 51 (10), 2871–2878. 10.1021/jm700967e. 18260614

[ref70] Brown D. G.; May-Dracka T. L.; Gagnon M. M.; Tommasi R. Trends and exceptions of physical properties on antibacterial activity for Gram-positive and Gram-negative pathogens. J. Med. Chem. 2014, 57 (23), 10144–10161. 10.1021/jm501552x. 25402200

[ref71] Zhao J.; Cochrane C. S.; Najeeb J.; Gooden D.; Sciandra C.; Fan P.; Lemaitre N.; Newns K.; Nicholas R. A.; Guan Z.; Thaden J. T.; Fowler V. G.; Spasojevic I.; Sebbane F.; Toone E. J.; Duncan C.; Gammans R.; Zhou P. Preclinical safety and efficacy characterization of an LpxC inhibitor against Gram-negative pathogens. Sci. Transl. Med. 2023, 15 (708), eadf5668 10.1126/scitranslmed.adf5668. 37556556 PMC10785772

[ref72] Ushiyama F.; Takashima H.; Matsuda Y.; Ogata Y.; Sasamoto N.; Kurimoto-Tsuruta R.; Ueki K.; Tanaka-Yamamoto N.; Endo M.; Mima M.; Fujita K.; Takata I.; Tsuji S.; Yamashita H.; Okumura H.; Otake K.; Sugiyama H. Lead optimization of 2-hydroxymethyl imidazoles as non-hydroxamate LpxC inhibitors: Discovery of TP0586532. Bioorg. Med. Chem. 2021, 30, 115964 10.1016/j.bmc.2020.115964. 33385955

[ref73] Bertani G. Studies on lysogenesis. I. The mode of phage liberation by lysogenic Escherichia coli. J. Bacteriol. 1951, 62 (3), 293–300. 10.1128/jb.62.3.293-300.1951. 14888646 PMC386127

[ref74] Jackman J. E.; Fierke C. A.; Tumey L. N.; Pirrung M.; Uchiyama T.; Tahir S. H.; Hindsgaul O.; Raetz C. R. Antibacterial agents that target lipid A biosynthesis in gram-negative bacteria. Inhibition of diverse UDP-3-*O*-(*R*-3-hydroxymyristoyl)-*N*-acetylglucosamine deacetylases by substrate analogs containing zinc binding motifs. J. Biol. Chem. 2000, 275 (15), 11002–11009. 10.1074/jbc.275.15.11002. 10753902

[ref75] Finney D. J. Probit Analysis: A Statistical Treatment of the Sigmoid Response Curve, 2nd ed.; Cambridge University Press: New York, NY, US, 1952.

[ref76] Yung-Chi C.; Prusoff W. H. Relationship between the inhibition constant (*K*_I_) and the concentration of inhibitor which causes 50% inhibition (*I*_50_) of an enzymatic reaction. Biochem. Pharmacol. 1973, 22 (23), 3099–3108. 10.1016/0006-2952(73)90196-2. 4202581

[ref77] Haupenthal J.; Baehr C.; Zeuzem S.; Piiper A. RNAse A-like enzymes in serum inhibit the anti-neoplastic activity of siRNA targeting polo-like kinase 1. Int. J. Cancer 2007, 121 (1), 206–210. 10.1002/ijc.22665. 17351902

[ref78] Mala P.; Siebs E.; Meiers J.; Rox K.; Varrot A.; Imberty A.; Titz A. Discovery of *N*-β-l-Fucosyl Amides as High-Affinity Ligands for the Pseudomonas aeruginosa Lectin LecB. J. Med. Chem. 2022, 65 (20), 14180–14200. 10.1021/acs.jmedchem.2c01373. 36256875 PMC9620277

[ref79] Dreger A.; Hoff K.; Agoglitta O.; Bülbül E. F.; Melesina J.; Sippl W.; Holl R. Synthesis, biological evaluation, and molecular docking studies of deoxygenated C-glycosides as LpxC inhibitors. Bioorg. Chem. 2021, 117, 105403 10.1016/j.bioorg.2021.105403. 34758434

[ref80] Berman H. M.; Westbrook J.; Feng Z.; Gilliland G.; Bhat T. N.; Weissig H.; Shindyalov I. N.; Bourne P. E. The Protein Data Bank. Nucleic Acids Res. 2000, 28 (1), 235–242. 10.1093/nar/28.1.235. 10592235 PMC102472

[ref81] Schrödinger Release 2019-1: Maestro, Protein Preparation Wizard, Prime, Epik, Ligprep, Confgen, Glide; Schrödinger LLC, New York, NY (USA), 2021.

[ref82] Schrödinger Release 2019-1; Desmond Molecular Dynamics System; D.E. Shaw Research: New York, NY, USA, 2019.

[ref83] Maestro-Desmond Interoperability Tools; Schrödinger: New York, NY, USA, 2019.

