# Supplementary material for: Development of Fragment-Based Inhibitors of the Bacterial Deacetylase LpxC with Low Nanomolar Activity
Source: J Med Chem. 2024 Sep 20;67(19):17363–91. doi: 10.1021/acs.jmedchem.4c01262 (PMC11472313; doi:10.1021/acs.jmedchem.4c01262)
Supplement: Supplementary file 2 — jm4c01262_si_002.pdf [file jm4c01262_si_002.pdf]

Supporting Information

Development of Fragment-Based Inhibitors of the Bacterial Deacetylase LpxC with Low Nanomolar Activity

Sebastian Mielniczuk,[a,b] Katharina Hoff,[a,b] Fady Baselious,[c] Yunki Li,[d,e] Jörg Haupenthal,[f] Andreas M. Kany,[f] Maria Riedner,[g] Holger Rohde,[b,h] Katharina Rox,[i,j] Anna K. H. Hirsch,[f,k,l] Isabelle Krimm,[d] Wolfgang Sippl,[c] and Ralph Holl[a,b,*]

[a]Institute of Organic Chemistry, Universität Hamburg, Martin-Luther-King-Platz 6, 20146 Hamburg, Germany

[b]German Center for Infection Research (DZIF), partner site Hamburg-Lübeck-Borstel-Riems, 20146 Hamburg, Germany

[c]Institute of Pharmacy, Martin-Luther-University of Halle-Wittenberg, Kurt-Mothes-Str. 3, 06120 Halle (Saale), Germany

[d]Team «Small Molecules for Biological Targets», Institut Convergence Plascan, Centre de Recherche en Cancérologie de Lyon, INSERM U1052-CNRS UMR5286, Centre Léon Bérard, Université de Lyon, Université Claude Bernard Lyon1, 69008 Lyon, France

[e]Shanghai Key Laboratory of Regulatory Biology, The Institute of Biomedical Sciences, School of Life Sciences, East China Normal University, Shanghai 200241, China

[f]Helmholtz Institute for Pharmaceutical Research Saarland (HIPS), Helmholtz Centre for Infection Research (HZI), Campus E8.1, 66123 Saarbrücken, Germany

[g]Technology Platform Mass Spectrometry, Universität Hamburg, Mittelweg 177, 20148 Hamburg, Germany

[h]Institute of Medical Microbiology, Virology and Hygiene, University Medical Center Hamburg-Eppendorf, Martinistr. 52, 20246 Hamburg, Germany

[i]Department of Chemical Biology, Helmholtz Centre for Infection Research (HZI), Inhoffenstr. 7, 38124 Braunschweig, Germany

[j]German Center for Infection Research (DZIF), partner site Hannover-Braunschweig, 38124 Braunschweig, Germany

[k]Helmholtz International Lab for Anti-infectives, Campus E8.1, 66123 Saarbrücken, Germany

[l]Department of Pharmacy, Saarland University, Campus E8.1, 66123 Saarbrücken, Germany

*To whom correspondence should be addressed. Tel.: +49-40-42838-2825; Fax: +49-40-42838-4325; Email: ralph.holl@uni-hamburg.de

Contents

Figure S1	S3
Figure S2	S3
Figure S3	S4
Figure S4	S4
Figure S5	S5
Figure S6	S5
Figure S7	S6
Figure S8	S7
Figure S9	S7
Table S1	S8
Table S2	S12
Table S3	S13
Determination of enzymatic parameters	S14
Synthetic procedures and analytical data of compounds (*S*)-**38b–n**, (*R*)-**38b–h,j,m,n**, (*S*)-**39b–n**, (*R*)-**39–h,j,m,n**, (*S*)-**13b–n**, and (*R*)-**13b–h,j,m,n**	S17
^{1}H and ^{13}C NMR spectra of representative compounds	S93
HPLC chromatograms of the test compounds	S159
References	S175

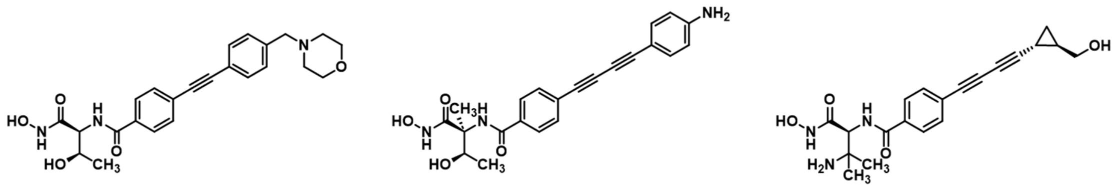

CHIR-090 LPC-138 ACHN-975

Figure S1: Structures of reported LpxC inhibitors.[1,2]

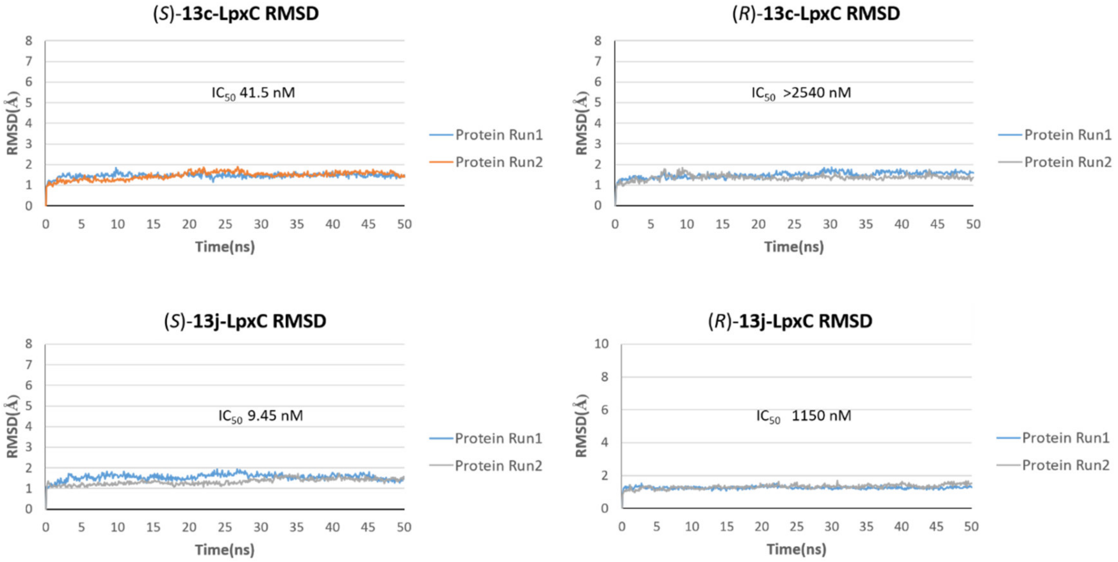

Figure S2: RMSD plots of the protein backbone heavy atoms for two independent 50 ns MD runs of LpxC-inhibitor complexes.

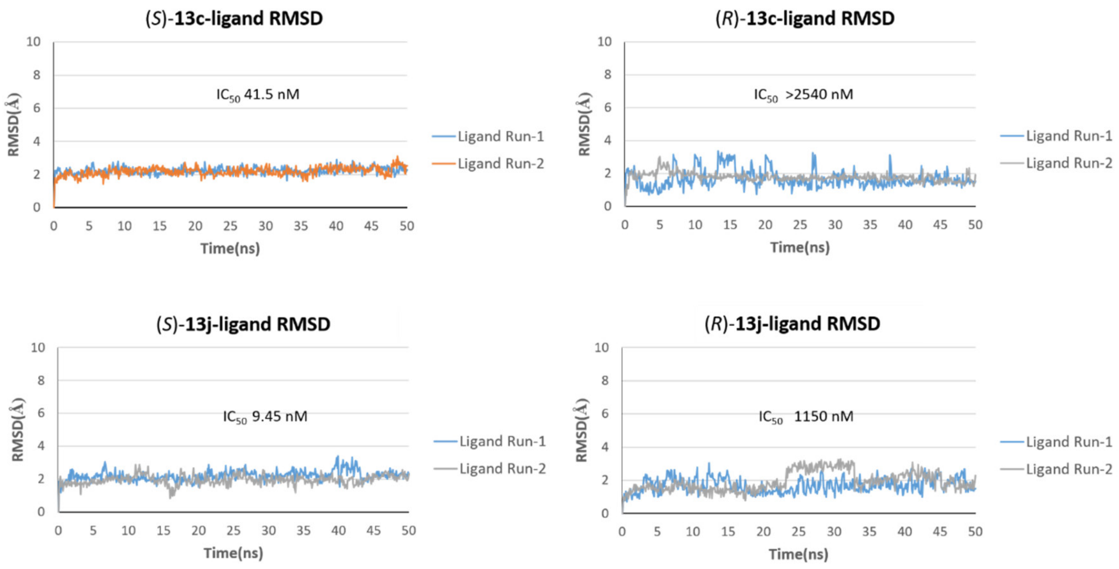

Figure S3: RMSD plots of the ligand heavy atoms for two independent 50 ns MD runs of LpxC-inhibitor complexes.

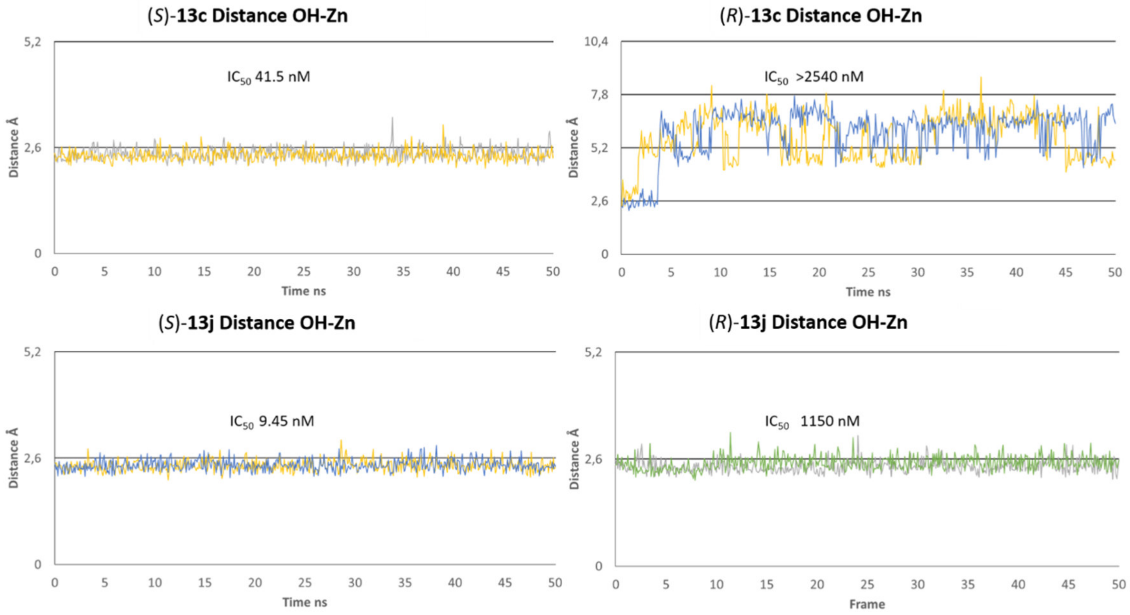

Figure S4: Plots of the measured distance between the inhibitor hydroxamic acid group (OH) and the catalytic Zn^{2+} ion from two independent 50 ns MD runs of LpxC-inhibitor complexes.

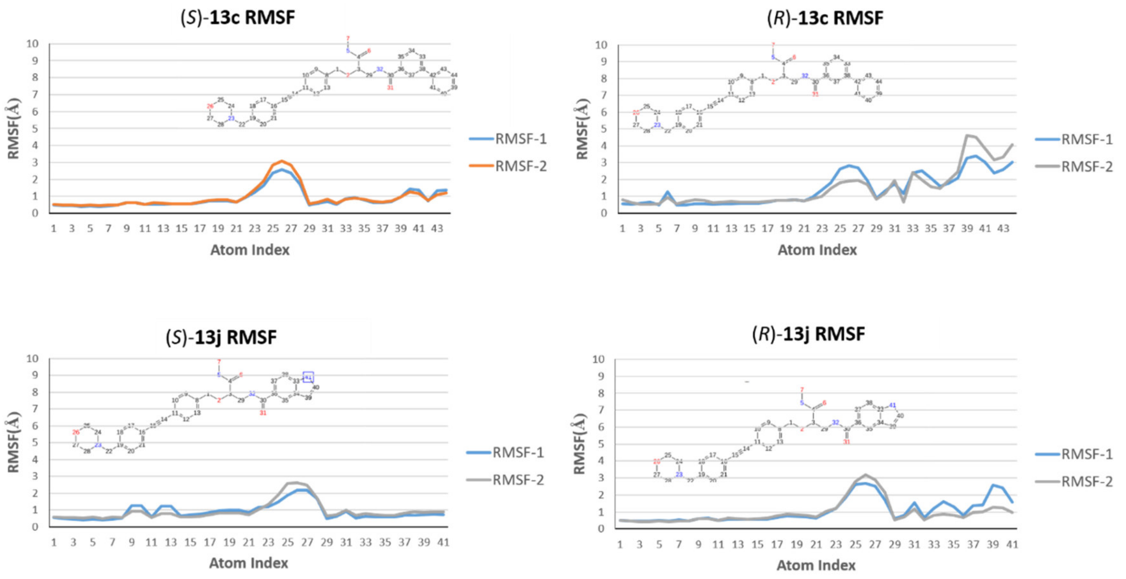

Figure S5: Fluctuation of the inhibitor atoms (given as RMSF values) from two independent 50 ns MD runs of LpxC-inhibitor complexes.

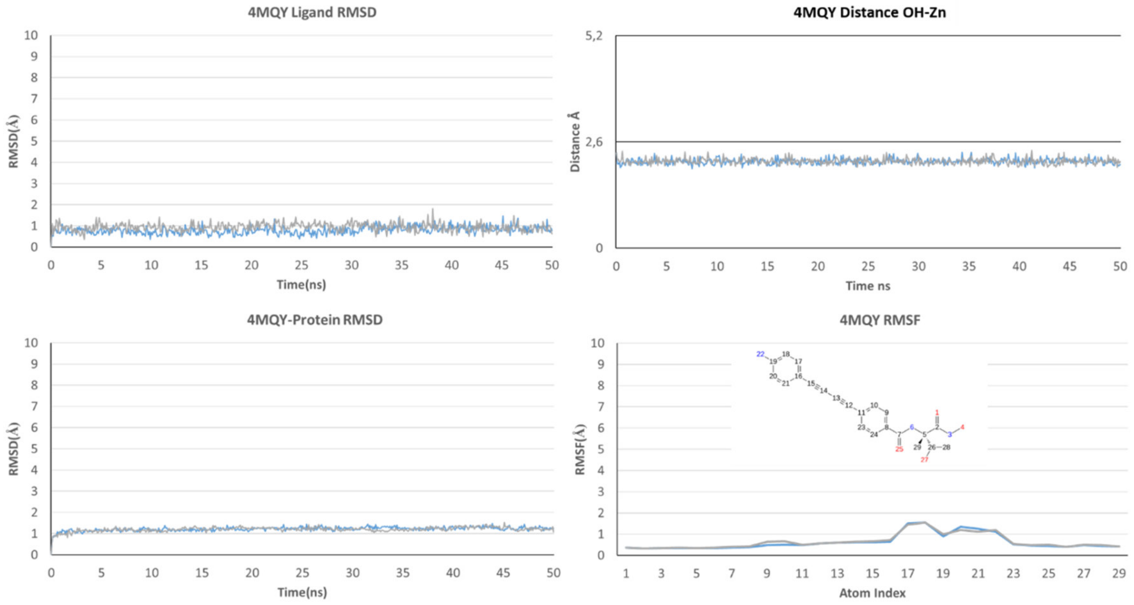

Figure S6: Protein and ligand RMDS values of two independent 50 ns simulations of the *E. coli* LpxC/LPC-138 complex X-ray structure (PDB ID 4MQY), distance between hydroxamic acid and the catalytic Zn^{2+} ion and ligand RMSF plot.

**LPC-138** (4MQY) H-bond occupancy (%)				
	Cys63	Glu78	Lys239	His265
MD-1	89.6	100.0	46.9	87.0
MD-2	93.2	100.0	43.1	85.6

(*S*)-**13j** H-bond occupancy (%)					
	Glu78	Thr191	Phe192	Asp197	His265
MD-1	100.0	96.0	9.4	88.2	10.0
MD-2	99.8	97.6	28.5	86.8	6.6

(*R*)-**13j** H-bond occupancy (%)			
	Glu78	Thr191	Asp197
MD-1	99.6	41.3	36.3
MD-2	99.8	11.2	72.1

(*S*)-**13c** H-bond occupancy (%)					
	Glu78	Thr191	Phe192	Gln202	His265
MD-1	99.2	98.4	24.4	41.5	2.8
MD-2	99.8	97.8	31.1	40.7	18.0

(*R*)-**13c** H-bond occupancy (%)			
	Glu78	Phe192	Gln202
MD-1	0	58.9	10.8
MD-2	0	82.2	30.3

Figure S7: Protein-ligand hydrogen-bond occupancy observed in two independent 50 ns MD runs.

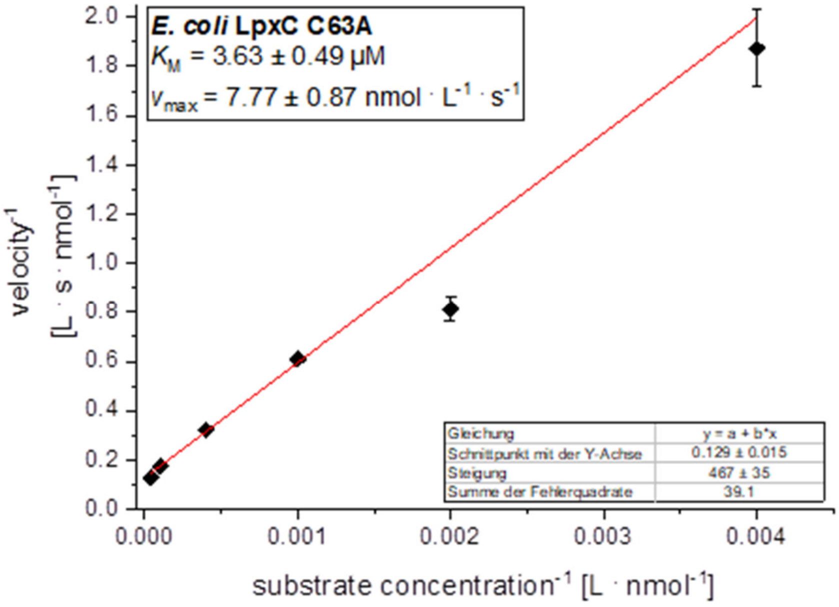

Figure S8: Lineweaver-Burk Plot for the determination of K_M and v_{max} of *E. coli* LpxC C63A. K_M = 3.63 ± 0.49 µM, v_{max} = 7.77 ± 0.87 nmol · L^{-1} · s^{-1}.

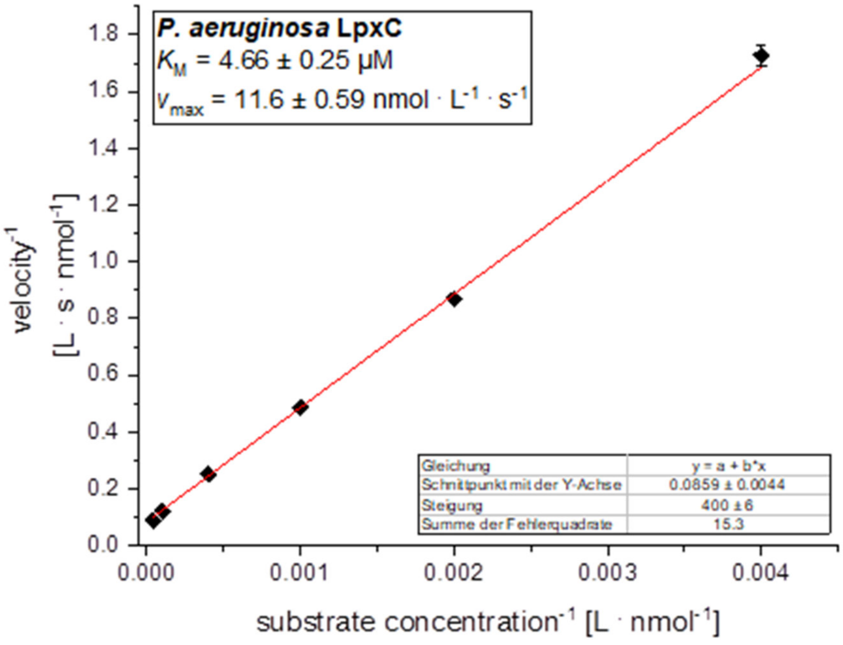

Figure S9: Lineweaver-Burk Plot for the determination of K_M and v_{max} of *P. aeruginosa* LpxC. K_M = 4.66 ± 0.25 µM, v_{max} = 11.6 ± 0.59 nmol · L^{-1} · s^{-1}.

Table S1: LpxC inhibitory activities and GLIDE SP docking scores of the studied inhibitors.

compound		K_i [µM]		Glide SP docking scores	
	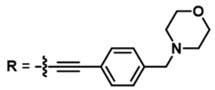	***E. coli*** **LpxC C63A**	***P. aeruginosa*** **LpxC**	**PDB ID: 4MQY (*E. coli* LpxC in complex with LPC-138)**	**PDB ID: 5VWM (*P. aeruginosa* LpxC in complex with CHIR-090)**
(*S*)-**8**	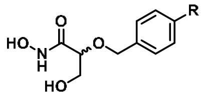	0.41 ± 0.074	n.d.	-8.51	-9.29
(*R*)-**8**		0.24 ± 0.046	n.d.	-8.54	-8.86
**9**	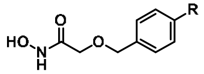	1.5 ± 0.37	n.d.	-8.39	-8.69
(2*S*,3*S*)-**10**	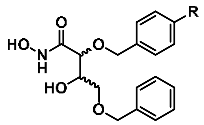	0.28 ± 0.21	n.d.	-9.24	-10.09
(2*R*,3*S*)-**10**		0.87 ± 0.29	n.d.	-9.24	-9.08
(*S*)-**11a**	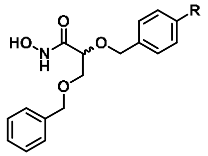	0.606 ± 0.356	0.725 ± 0.335	-8.50	-9.19
(*R*)-**11a**		3.62 ± 0.64	n.d.	-8.30	-8.91

(*S*)-**12a**	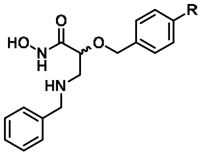	4.59 ± 1.26	0.862 ± 0.070	-8.84	-9.06
(*R*)-**12a**		5.90 ± 2.02	n.d.	-8.80	-9.07
(*S*)-**37**	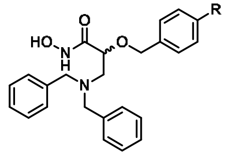	>2.54	>3.25	-8.97	-9.49
(*R*)-**37**		>2.54	n.d.	-8.99	-7.64
**compound**	**R =**	**K_i [nM]**		**Glide SP docking scores**	
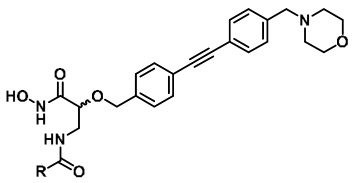		***E. coli*** **LpxC C63A**	***P. aeruginosa*** **LpxC**	**PDB ID: 4MQY (*E. coli* LpxC in complex with LPC-138)**	**PDB ID: 5VWM (*P. aeruginosa* LpxC in complex with CHIR-090)**
(*S*)-**13a**	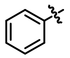	149 ± 56	58.2 ± 0.1	-9.31	-10.30
(*R*)-**13a**		2560 ± 1080	n.d.	-8.69	-9.57
(*S*)-**13b**	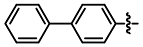	>2540	n.d.	-9.79	-8.75
(*R*)-**13b**		>2540	n.d.	-9.04	-8.20

(*S*)-**13c**	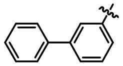	41.5 ± 5.7	149 ± 14	-11.08	-10.11
(*R*)-**13c**		>2540	n.d.	-9.73	-9.94
(*S*)-**13d**	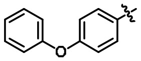	323 ± 126	196 ± 18	-10.86	-10.63
(*R*)-**13d**		>2540	n.d.	-10.92	-9.56
(*S*)-**13e**	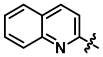	377 ± 83	168 ± 8	-9.97	-10.67
(*R*)-**13e**		>2540	n.d.	-9.67	-9.49
(*S*)-**13f**	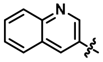	117 ± 21	91.1 ± 17.5	-10.26	-10.46
(*R*)-**13f**		>2540	n.d.	-10.18	-9.65
(*S*)-**13g**	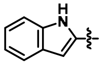	87.0 ± 58.5	46.0 ± 16.4	-10.66	-10.35
(*R*)-**13g**		>2540	n.d.	-8.89	-9.52
(*S*)-**13h**	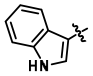	10.7 ± 1.2	5.91 ± 1.47	-9.61	-10.57
(*R*)-**13h**		1580 ± 216	631 ± 198	-9.55	-9.85
(*S*)-**13i**	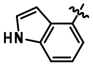	15.3 ± 3.6	10.3 ± 2.0	-10.29	-11.47

(*S*)-**13j**	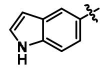	9.45 ± 1.01	5.58 ± 0.53	-11.37	-11.53
(*R*)-**13j**		1150 ± 317	n.d.	-9.80	-10.70
(*S*)-**13k**	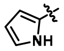	97.1 ± 9.4	29.0 ± 7.6	-9.94	-11.39
(*S*)-**13l**	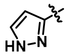	80.9 ± 9.0	18.0 ± 2.9	-9.10	-10.25
(*S*)-**13m**	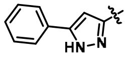	85.2 ± 21.7	41.9 ± 18.5	-10.38	-9.93
(*R*)-**13m**		>2540	n.d.	-10.02	-9.43
(*S*)-**13n**	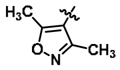	127 ± 40	80.4 ± 9.7	-9.17	-10.14
(*R*)-**13n**		>2540	n.d.	-8.81	-9.47

Table S2: Parameters of the MS/MS-method using the triple quadrupole mass spectrometer QTRAP 5500 (AB Sciex LLC).

scan time	12 – 19 min
curtain gas (CUR)	20 psi
collision gas (CAD)	medium
ion spray voltage (IS)	–4500 V
temperature (TEM)	450 °C
ion source gas 1	60 psi
ion source gas 2	50 psi
declustering potential (DP)	–170 V
entrance potential (EP)	–10 V
dwell time	150 ms

Table S3: Mass transitions of the internal standard (caffeine) and the compounds.

	Q1 mass	***Q3 mass***	***DP [V]***	***CE [V]***	***CXP [V]***
caffeine	195.024	138.0	130	25.0	14.0
caffeine	195.024	110.0	130	31.0	18.0
(*S*)-**13g**	553.141	320.1	1	33	40
(*S*)-**13g**	553.141	290.2	1	23	20
(*S*)-**13h**	553.178	290.1	1	23	14
(*S*)-**13h**	553.178	204.1	1	67	22
(*S*)-**13i**	553.038	290.0	1	23	18
(*S*)-**13i**	553.038	466.2	1	21	30
(*S*)-**13j**	553.214	290.1	1	27	18
(*S*)-**13j**	553.214	204.2	1	71	18
(*S*)-**13l**	504.150	290.2	1	19	14
(*S*)-**13l**	504.150	417.3	1	23	16
(*S*)-**13n**	533.132	446.1	1	27	24
(*S*)-**13n**	533.132	219.0	1	41	24

DP: declustering potential; CE: collision energy; CXP: collision cell exit potential.

Determination of enzymatic parameters

The values of K_M and v_{max} for the *E. coli* LpxC C63A- as well as the *P. aeruginosa* LpxC-catalyzed deacetylation of UDP-3-*O*-[(*R*)-3-hydroxymyristoyl]-*N*-acetylglucosamine (**1**) were determined using the conditions of the established LC-MS/MS-based enzyme assay.

<u>*E. coli* LpxC C63A</u>

The wells in a black, non-binding, 96-well fluorescence microplate (Greiner Bio One, Frickenhausen) were filled with 93 µL of varying concentrations of UDP-3-*O*-[(*R*)-3-hydroxymyristoyl]-*N*-acetylglucosamine (26.9 µM, 10.8 µM, 2.69 µM, 1.08 µM, 0.538 µM, 0.269 µM) in assay buffer (40 mM sodium morpholinoethanesulfonic acid (pH = 6.0), 80 µM dithiothreitol, 0.02% Brij 35). In order to determine the parameters under the conditions of the inhibition assay, 2 µL of DMSO were added. The addition of 5 µL of a solution of purified *E. coli* LpxC C63A (5 µg · mL^{-1}) in assay buffer led to final concentrations of 0.25 – 25 µM UDP-3-*O*-[(*R*)-3-hydroxymyristoyl]-*N*-acetylglucosamine, 7.4 nM *E. coli* LpxC C63A, and 2% DMSO. Next, the microplate was incubated at 37 °C in a plate shaker, and the reactions were stopped at different times (0 s, 10 s, 20 s, 30 s, 40 s, 50 s, 60 s, 70 s, 90 s) by adding 40 µL of 0.625 M hydrochloric acid. The reaction mixtures were further incubated for at least 10 min, sealed, and stored at –80 °C until analysis.

<u>*P. aeruginosa* LpxC</u>

The wells in a black, non-binding, 96-well fluorescence microplate (Greiner Bio One, Frickenhausen) were filled with 93 µL of varying concentrations of UDP-3-*O*-[(*R*)-3-

hydroxymyristoyl]-*N*-acetylglucosamine (26.9 μM, 10.8 μM, 2.69 μM, 1.08 μM, 0.538 μM, 0.269 μM) in assay buffer (50 mM KH_2PO_4/K_2HPO_4 (pH = 7.5), 80 μM dithiothreitol, 0.02% Brij 35). In order to determine the parameters under the conditions of the inhibition assay, 2 μL of DMSO were added. The addition of 5 μL of a solution of purified *P. aeruginosa* LpxC (50 μg · mL^{-1}) in assay buffer led to final concentrations of 0.25 – 25 μM UDP-3-*O*-[(*R*)-3-hydroxymyristoyl]-*N*-acetylglucosamine, 75 nM LpxC, and 2% DMSO. Next, the microplate was incubated at 37 °C in a plate shaker, and the reactions were stopped at different times (0 s, 10 s, 20 s, 30 s, 40 s, 50 s, 60 s, 70 s, 90 s) by adding 40 μL of 0.625 M hydrochloric acid. The reaction mixtures were further incubated for at least 10 min, sealed, and stored at –80 °C until analysis.

LC-MS/MS-analysis

The reaction mixtures were separated by ultra-high performance liquid-chromatography (1290 Infinity II UHPLC, Agilent Technologies), and the eluted compounds were analyzed by mass spectrometry using electrospray ionization in the negative ion mode with a triple quadrupole linear ion trap mass spectrometer (QTRAP 5500, AB Sciex LLC).

UHPLC method: column: Nucleodur® C18 Gravity-SB (Ø = 3 mm, *h* = 100 mm, Macherey-Nagel), coupled to a Universal RP-guard column (Ø = 2 mm, *h* = 4 mm, Macherey-Nagel); flow rate: 0.3 mL · min^{-1}; injection volume: 3.0 μL; solvents: A: 20 mM ammonium formate in water; B: 1 mM ammonium formate in acetonitrile/isopropanol/water (47.5/42.75/9.75); gradient elution: (B%): 0 – 1 min: 30%, 1 – 16 min: gradient from 30% to 90%, 16 – 17 min: 90%, 17 – 17.5 min: gradient from 90% to 30%, 17.5 – 21.5 min: 30%; detection: 12 – 19 min; t_R (**1**) = 12.2 min, t_R (**2**) = 13.0 min.

To analyze the eluted compounds by mass spectrometry, an MRM method was applied. The specific parameters of this method are given in Table S1. After detection and selection of the precursor ions (**1**: *m*/*z* 832; **2**: *m*/*z* 790), both analytes were fragmented, leading to three identical product ions (*m*/*z* (product 1) 385, collision energy = –60 V; *m*/*z* (product 2) 159, collision energy = –80 V; *m*/*z* (product 3) 79, collision energy = –140 V). The mass transitions 832 → 79 (substrate **1**) and 790 → 79 (product **2**) were used as quantifiers, the other mass transitions were used as qualifiers. The final concentrations of substrate **1** and product **2** were quantified by comparing the peak areas of the quantifiers.

Subsequently, the product concentrations were plotted against the respective reaction times and the initial velocities were calculated using a linear regression with the aid of the software Origin. Next, the reciprocal initial velocities were plotted against the reciprocal initial substrate concentrations leading to the double reciprocal Lineweaver–Burk plot. Last, a linear regression with the aid of the software Origin yielded K_M and v_{max} (Figures S9 and S10).

Synthetic procedures and analytical data

Methyl (*S*)-3-([1,1'-biphenyl]-4-carboxamido)-2-[(4-iodobenzyl)oxy]propanoate ((*S*)-38b)

Under N_2 atmosphere, a 1.0 M solution of trimethylphosphane in toluene (2.0 mL, 2.0 mmol) was added to an ice-cooled mixture of biphenyl-4-carboxylic acid (180 mg, 0.92 mmol), (*S*)-**28** (300 mg, 0.84 mmol), and 2,2'-dithiodipyridine (110 mg, 0.50 mmol). After stirring the reaction mixture for 15 min at 0 °C, the ice-bath was removed and the mixture was stirred for 72 h at ambient temperature. Then, water (1.0 mL) was added and the mixture was stirred for 20 min. Afterwards, the mixture was diluted with dichloromethane and washed with a saturated aqueous solution of $NaHCO_3$, an ice-cold solution of 1.0 M HCl, and water. The combined organic layers were dried (Na_2SO_4), filtered, and the solvent was removed *in vacuo*. The residue was purified by flash column chromatography (Ø = 4 cm, h = 24 cm, V = 30 mL, petroleum ether/ethyl acetate = 2/1, R_f = 0.27) to give (*S*)-**38b** as colorless solid (320 mg, 0.62 mmol, 74%). m.p.: 163 °C; $[\alpha]_D^{20}$ = -26.5 (2.6, methanol); HPLC (method 1): t_R = 25.5 min, purity 99.8%.

Methyl (*R*)-3-([1,1'-biphenyl]-4-carboxamido)-2-[(4-iodobenzyl)oxy]propanoate ((*R*)-38b)

Under N_2 atmosphere, a 1.0 M solution of trimethylphosphane in toluene (2.0 mL, 2.0 mmol) was added to an ice-cooled mixture of biphenyl-4-carboxylic acid (200 mg, 0.98 mmol), (*R*)-**28** (300 mg, 0.83 mmol), and 2,2'-dithiodipyridine (180 mg, 0.81 mmol). After stirring the reaction mixture for 15 min at 0 °C, the ice-bath was

removed and the mixture was stirred for 48 h at ambient temperature. Then, water (1.0 mL) was added and the mixture was stirred for 20 min. Afterwards, the mixture was diluted with dichloromethane and washed with a saturated aqueous solution of $NaHCO_3$, an ice-cold solution of 1.0 M HCl, and water. The combined organic layers were dried (Na_2SO_4), filtered, and the solvent was removed *in vacuo*. The residue was purified by flash column chromatography (Ø = 4 cm, h = 20 cm, V = 30 mL, petroleum ether/ethyl acetate = 2/1, R_f = 0.27) to give (*R*)-**38b** as colorless solid (260 mg, 0.51 mmol, 61%). m.p.: 164 °C; $[\alpha]_D^{20}$ = +22.5 (1.2, methanol); HPLC (method 1): t_R = 25.3 min, purity 98.0%.

Spectroscopic data of (*S*)-**38b** *and* (*R*)-**38b**:

^{1}H NMR (DMSO-d_6): δ [ppm] = 3.48 – 3.56 (m, 1H, OCHCH_2NH), 3.63 – 3.70 (m, 1H, OCHCH_2NH), 3.68 (s, 3H, CO_2CH_3), 4.22 (dd, *J* = 7.3/5.1 Hz, 1H, OC*H*CH_2NH), 4.42 (d, *J* = 12.3 Hz, 1H, OCH_2Ar), 4.59 (d, *J* = 12.3 Hz, 1H, OCH_2Ar), 7.13 – 7.17 (m, 2H, 2''-$H_{4\text{-iodophenyl}}$, 6''-$H_{4\text{-iodophenyl}}$), 7.39 – 7.43 (m, 1H, 4'-$H_{[1,1'\text{-biphenyl}]\text{-4-yl}}$), 7.47 – 7.53 (m, 2H, 3'-$H_{[1,1'\text{-biphenyl}]\text{-4-yl}}$, 5'-$H_{[1,1'\text{-biphenyl}]\text{-4-yl}}$), 7.60 – 7.65 (m, 2H, 3''-$H_{4\text{-iodophenyl}}$, 5''-$H_{4\text{-iodophenyl}}$), 7.71 – 7.76 (m, 2H, 2'-$H_{[1,1'\text{-biphenyl}]\text{-4-yl}}$, 6'-$H_{[1,1'\text{-biphenyl}]\text{-4-yl}}$), 7.76 – 7.80 (m, 2H, 2-$H_{[1,1'\text{-biphenyl}]\text{-4-yl}}$, 6-$H_{[1,1'\text{-biphenyl}]\text{-4-yl}}$), 7.88 – 7.92 (m, 2H, 3-$H_{[1,1'\text{-biphenyl}]\text{-4-yl}}$, 5-$H_{[1,1'\text{-biphenyl}]\text{-4-yl}}$), 8.71 (t, *J* = 5.9 Hz, 1H, CON*H*); ^{13}C NMR (DMSO-d_6): δ [ppm] = 41.4 (1C, OCH*C*H_2NH), 51.9 (1C, CO_2CH_3), 70.7 (1C, O*C*H_2Ar), 76.5 (1C, O*C*HCH_2NH), 93.6 (1C, C-4''$_{4\text{-iodophenyl}}$), 126.5 (2C, C-$2_{[1,1'\text{-biphenyl}]\text{-4-yl}}$, C-$6_{[1,1'\text{-biphenyl}]\text{-4-yl}}$), 126.9 (2C, C-2'$_{[1,1'\text{-biphenyl}]\text{-4-yl}}$, C-6'$_{[1,1'\text{-biphenyl}]\text{-4-yl}}$), 127.9 (2C, C-$3_{[1,1'\text{-biphenyl}]\text{-4-yl}}$, C-$5_{[1,1'\text{-biphenyl}]\text{-4-yl}}$), 128.0 (1C, C-4'$_{[1,1'\text{-biphenyl}]\text{-4-yl}}$), 129.0 (2C, C-3'$_{[1,1'\text{-biphenyl}]\text{-4-yl}}$, C-5'$_{[1,1'\text{-biphenyl}]\text{-4-yl}}$), 129.9 (2C, C-2''$_{4\text{-iodophenyl}}$, C-6''$_{4\text{-iodophenyl}}$), 133.0 (1C, C-$4_{[1,1'\text{-biphenyl}]\text{-4-yl}}$), 136.9 (2C, C-3''$_{4\text{-iodophenyl}}$, C-5''$_{4\text{-iodophenyl}}$), 137.5 (1C, C-1''$_{4\text{-iodophenyl}}$), 139.2 (1C, C-1'$_{[1,1'\text{-biphenyl}]\text{-4-yl}}$), 142.8 (1C, C-$1_{[1,1'\text{-}}$

$_{biphenyl]-4-yl}$), 166.2 (1C, *C*ONH), 171.0 (1C, *C*O_2CH_3); IR (neat): $\tilde{\nu}$ [cm^{-1}] = 3300, 2939, 1739, 1636, 1537, 1484, 1294, 1208, 1131, 1005, 853, 803, 748, 731, 685, 445; HRMS (*m*/*z*): $[M+H]^+$ calcd for $C_{24}H_{23}INO_4$: 516.0666, found: 516.0684.

Methyl (*S*)-3-([1,1'-biphenyl]-3-carboxamido)-2-[(4-iodobenzyl)oxy]propanoate ((*S*)-38c)

Under N_2 atmosphere, a 1.0 M solution of trimethylphosphane in toluene (2.2 mL, 2.2 mmol) was added to an ice-cooled mixture of biphenyl-3-carboxylic acid (200 mg, 1.0 mmol), (*S*)-**28** (330 mg, 0.91 mmol), and 2,2'-dithiodipyridine (100 mg, 0.47 mmol). After stirring the reaction mixture for 20 min at 0 °C, the ice-bath was removed and the mixture was stirred for 48 h at ambient temperature. Then, water (1.5 mL) was added and the mixture was stirred for 20 min. Afterwards, the mixture was diluted with dichloromethane and washed with a saturated aqueous solution of $NaHCO_3$, an ice-cold solution of 1.0 M HCl, and water. The combined organic layers were dried (Na_2SO_4), filtered, and the solvent was removed *in vacuo*. The residue was purified by flash column chromatography (Ø = 4 cm, h = 24 cm, V = 30 mL, petroleum ether/ethyl acetate = 2/1, R_f = 0.27) to give (*S*)-**38c** as colorless solid (360 mg, 0.69 mmol, 76%). m.p.: 99 °C; $[\alpha]_D^{20}$ = -28.6 (2.2, methanol); HPLC (method 1): t_R = 25.6 min, purity 97.7%.

Methyl (*R*)-3-([1,1'-biphenyl]-3-carboxamido)-2-[(4-iodobenzyl)oxy]propanoate ((*R*)-38c)

Under N_2 atmosphere, a 1.0 M solution of trimethylphosphane in toluene (2.1 mL, 2.1 mmol) was added to an ice-cooled mixture of biphenyl-3-carboxylic acid (180 mg,

0.90 mmol), (*R*)-**28** (320 mg, 0.88 mmol), and 2,2'-dithiodipyridine (99 mg, 0.45 mmol). After stirring the reaction mixture for 20 min at 0 °C, the ice-bath was removed and the mixture was stirred for 36 h at ambient temperature. Then, water (1.0 mL) was added and the mixture was stirred for 20 min. Afterwards, the mixture was diluted with dichloromethane and washed with a saturated aqueous solution of $NaHCO_3$, an ice-cold solution of 1.0 M HCl, and water. The combined organic layers were dried (Na_2SO_4), filtered, and the solvent was removed *in vacuo*. The residue was purified by flash column chromatography (Ø = 4 cm, h = 21 cm, V = 30 mL, petroleum ether/ethyl acetate = 2/1, R_f = 0.27) to give (*R*)-**38c** as colorless solid (270 mg, 0.53 mmol, 61%). m.p.: 99 °C; $[\alpha]_D^{20}$ = +28.7 (1.5, methanol); HPLC (method 1): t_R = 25.4 min, purity 98.8%.

Spectroscopic data of (*S*)-**38c** *and* (*R*)-**38c**:

^{1}H NMR (DMSO-d_6): δ [ppm] = 3.47 – 3.57 (m, 1H, OCHCH_2NH), 3.63 – 3.73 (m, 1H, OCHCH_2NH), 3.68 (s, 3H, CO_2CH_3), 4.21 (dd, *J* = 7.4/4.9 Hz, 1H, OC*H*CH_2NH), 4.42 (d, *J* = 12.3 Hz, 1H, OCH_2Ar), 4.60 (d, *J* = 12.3 Hz, 1H, OCH_2Ar), 7.12 – 7.18 (m, 2H, 2''-$H_{4\text{-iodophenyl}}$, 6''-$H_{4\text{-iodophenyl}}$), 7.38 – 7.45 (m, 1H, 4'-$H_{[1,1'\text{-biphenyl]-3-yl}}$), 7.47 – 7.54 (m, 2H, 3'-$H_{[1,1'\text{-biphenyl]-3-yl}}$, 5'-$H_{[1,1'\text{-biphenyl]-3-yl}}$), 7.54 – 7.62 (m, 3H, 3''-$H_{4\text{-iodophenyl}}$, 5''-$H_{4\text{-iodophenyl}}$, 5-$H_{[1,1'\text{-biphenyl]-3-yl}}$), 7.70 – 7.76 (m, 2H, 2'-$H_{[1,1'\text{-biphenyl]-3-yl}}$, 6'-$H_{[1,1'\text{-biphenyl]-3-yl}}$), 7.77 – 7.82 (m, 1H, 4-$H_{[1,1'\text{-biphenyl]-3-yl}}$), 7.82 – 7.86 (m, 1H, 6-$H_{[1,1'\text{-biphenyl]-3-yl}}$), 8.08 – 8.12 (m, 1H, 2-$H_{[1,1'\text{-biphenyl]-3-yl}}$), 8.84 (t, *J* = 5.9 Hz, 1H, CON*H*); ^{13}C NMR (DMSO-d_6): δ [ppm] = 41.4 (1C, OCH*C*H_2NH), 52.0 (1C, CO_2CH_3), 70.7 (1C, O*C*H_2Ar), 76.5 (1C, O*C*HCH_2NH), 93.6 (1C, C-4''$_{4\text{-iodophenyl}}$), 125.4 (1C, C-2$_{[1,1'\text{-biphenyl]-3-yl}}$), 126.5 (1C, C-4$_{[1,1'\text{-biphenyl]-3-yl}}$), 126.9 (2C, C-2'$_{[1,1'\text{-biphenyl]-3-yl}}$, C-6'$_{[1,1'\text{-biphenyl]-3-yl}}$), 127.8 (1C, C-4'$_{[1,1'\text{-biphenyl]-3-yl}}$), 129.0 (2C, C-3'$_{[1,1'\text{-biphenyl]-3-yl}}$, C-5'$_{[1,1'\text{-biphenyl]-3-yl}}$), 129.1 (1C, C-5$_{[1,1'\text{-biphenyl]-3-yl}}$), 129.5

(1C, C-6$_{[1,1'\text{-biphenyl}]\text{-3-yl}}$), 129.9 (2C, C-2''$_{4\text{-iodophenyl}}$, C-6''$_{4\text{-iodophenyl}}$), 134.8 (1C, C-3$_{[1,1'\text{-biphenyl}]\text{-3-yl}}$), 136.9 (2C, C-3''$_{4\text{-iodophenyl}}$, C-5''$_{4\text{-iodophenyl}}$), 137.5 (1C, C-1''$_{4\text{-iodophenyl}}$), 139.6 (1C, C-1'$_{[1,1'\text{-biphenyl}]\text{-3-yl}}$), 140.2 (1C, C-1$_{[1,1'\text{-biphenyl}]\text{-3-yl}}$), 166.4 (1C, *C*ONH), 171.0 (1C, *C*O$_2$CH$_3$); IR (neat): $\tilde{\nu}$ [cm^{-1}] = 3264, 2951, 1739, 1636, 1533, 1322, 1215, 1137, 1046, 1005, 800, 750, 697, 464; HRMS (*m/z*): [M+H]$^+$ calcd for $C_{24}H_{23}INO_4$: 516.0666, found: 516.0658.

Methyl (*S*)-2-[(4-iodobenzyl)oxy]-3-(4-phenoxybenzamido)propanoate ((*S*)-38d)

Under N$_2$ atmosphere, a 1.0 M solution of trimethylphosphane in toluene (2.0 mL, 2.0 mmol) was added to an ice-cooled mixture of 4-phenoxybenzoic acid (210 mg, 0.96 mmol), (*S*)-**28** (310 mg, 0.85 mmol), and 2,2'-dithiodipyridine (98 mg, 0.44 mmol). After stirring the reaction mixture for 20 min at 0 °C, the ice-bath was removed and the mixture was stirred for 48 h at ambient temperature. Then, water (1.5 mL) was added and the mixture was stirred for 20 min. Afterwards, the mixture was diluted with dichloromethane and washed with a saturated aqueous solution of NaHCO$_3$, an ice-cold solution of 1.0 M HCl, and water. The combined organic layers were dried (Na$_2$SO$_4$), filtered, and the solvent was removed *in vacuo*. The residue was purified by flash column chromatography (Ø = 4 cm, h = 24 cm, V = 30 mL, petroleum ether/ethyl acetate = 2/1, R_f = 0.27) to give (*S*)-**38d** as colorless solid (350 mg, 0.66 mmol, 77%). m.p.: 126 °C; $[\alpha]_D^{20}$ = -14.3 (2.3, methanol); HPLC (method 1): t_R = 25.2 min, purity 100%.

Methyl (*R*)-2-[(4-iodobenzyl)oxy]-3-(4-phenoxybenzamido)propanoate ((*R*)-38d)

Under N_2 atmosphere, a 1.0 M solution of trimethylphosphane in toluene (2.2 mL, 2.2 mmol) was added to an ice-cooled mixture of 4-phenoxybenzoic acid (220 mg, 1.0 mmol), (*R*)-**28** (330 mg, 0.92 mmol), and 2,2'-dithiodipyridine (100 mg, 0.46 mmol). After stirring the reaction mixture for 20 min at 0 °C, the ice-bath was removed and the mixture was stirred for 48 h at ambient temperature. Then, water (1.0 mL) was added and the mixture was stirred for 20 min. Afterwards, the mixture was diluted with dichloromethane and washed with a saturated aqueous solution of $NaHCO_3$, an ice-cold solution of 1.0 M HCl, and water. The combined organic layers were dried (Na_2SO_4), filtered, and the solvent was removed *in vacuo*. The residue was purified by flash column chromatography (Ø = 4 cm, h = 21 cm, V = 30 mL, petroleum ether/ethyl acetate = 2/1, R_f = 0.27) to give (*R*)-**38d** as colorless solid (330 mg, 0.62 mmol, 68%). m.p.: 125 °C; $[\alpha]_D^{20}$ = +12.2 (1.2, methanol); HPLC (method 1): t_R = 25.2 min, purity 99.3%.

Spectroscopic data of (*S*)-**38d** *and* (*R*)-**38d**:

^{1}H NMR (DMSO-d_6): δ [ppm] = 3.44 – 3.51 (m, 1H, OCHCH_2NH), 3.60 – 3.66 (m, 1H, OCHCH_2NH), 3.67 (s, 3H, CO_2CH_3), 4.18 (dd, *J* = 7.4/5.0 Hz, 1H, OC*H*CH_2NH), 4.40 (d, *J* = 12.3 Hz, 1H, OCH_2Ar), 4.58 (d, *J* = 12.3 Hz, 1H, OCH_2Ar), 7.01 – 7.06 (m, 2H, 3''-$H_{benzoyl}$, 5''-$H_{benzoyl}$), 7.08 – 7.12 (m, 2H, 2'''-$H_{phenoxy}$, 6'''-$H_{phenoxy}$), 7.12 – 7.16 (m, 2H, 2'-$H_{4\text{-iodophenyl}}$, 6'-$H_{4\text{-iodophenyl}}$), 7.19 – 7.24 (m, 1H, 4'''-$H_{phenoxy}$), 7.42 – 7.47 (m, 2H, 3'''-$H_{phenoxy}$, 5'''-$H_{phenoxy}$), 7.59 – 7.63 (m, 2H, 3'-$H_{4\text{-iodophenyl}}$, 5'-$H_{4\text{-iodophenyl}}$), 7.82 – 7.86 (m, 2H, 2''-$H_{benzoyl}$, 6''-$H_{benzoyl}$), 8.61 (t, *J* = 5.9 Hz, 1H, CON*H*); ^{13}C NMR (DMSO-d_6): δ [ppm] = 41.4 (1C, OCH*C*H_2NH), 51.9 (1C, CO_2CH_3), 70.7 (1C, O*C*H_2Ar), 76.5 (1C, O*C*HCH_2NH), 93.6 (1C, C-4'$_{4\text{-iodophenyl}}$), 117.4 (2C, C-3''$_{benzoyl}$, C-5''$_{benzoyl}$), 119.5

(2C, C-2'''$_{phenoxy}$, C-6'''$_{phenoxy}$), 124.3 (1C, C-4'''$_{phenoxy}$), 128.8 (1C, C-1''$_{benzoyl}$), 129.4 (2C, C-2''$_{benzoyl}$, C-6''$_{benzoyl}$), 129.9 (2C, C-2'$_{4\text{-iodophenyl}}$, C-6'$_{4\text{-iodophenyl}}$), 130.2 (2C, C-3'''$_{phenoxy}$, C-5'''$_{phenoxy}$), 136.9 (2C, C-3'$_{4\text{-iodophenyl}}$, C-5'$_{4\text{-iodophenyl}}$), 137.5 (1C, C-1'$_{4\text{-iodophenyl}}$), 155.6 (1C, C-1'''$_{phenoxy}$), 159.5 (1C, C-4''$_{benzoyl}$), 165.7 (1C, *C*ONH), 171.0 (1C, *C*O_2CH_3); IR (neat): $\tilde{\nu}$ [cm^{-1}] = 3273, 3040, 2948, 2873, 1741, 1628, 1586, 1540, 1486, 1241, 1213, 1098, 1002, 988, 841, 689, 626, 536, 477; HRMS (*m*/*z*): $[M+H]^+$ calcd for $C_{24}H_{23}INO_5$: 532.0615, found: 532.0618.

Methyl (*S*)-2-[(4-iodobenzyl)oxy]-3-(quinoline-2-carboxamido)propanoate ((*S*)-38e)

Under N_2 atmosphere, a 1.0 M solution of trimethylphosphane in toluene (2.3 mL, 2.3 mmol) was added to an ice-cooled mixture of quinaldic acid (210 mg, 1.2 mmol), (*S*)-**28** (340 mg, 0.94 mmol), and 2,2'-dithiodipyridine (100 mg, 0.47 mmol). After stirring the reaction mixture for 20 min at 0 °C, the ice-bath was removed and the mixture was stirred for 72 h at ambient temperature. Then, water (1.5 mL) was added and the mixture was stirred for 20 min. Afterwards, the mixture was diluted with dichloromethane and washed with a saturated aqueous solution of $NaHCO_3$, an ice-cold solution of 1.0 M HCl, and water. The combined organic layers were dried (Na_2SO_4), filtered, and the solvent was removed *in vacuo*. The residue was purified by flash column chromatography (Ø = 4 cm, h = 23 cm, V = 30 mL, petroleum ether/ethyl acetate = 1/1, R_f = 0.60) to give (*S*)-**38e** as yellow solid (420 mg, 0.86 mmol, 92%). m.p.: 75 °C; $[\alpha]_D^{20}$ = -18.2 (2.3, methanol); HPLC (method 1): t_R = 25.0 min, purity 98.6%.

Methyl (*R*)-2-[(4-iodobenzyl)oxy]-3-(quinoline-2-carboxamido)propanoate ((*R*)-38e)

Under N_2 atmosphere, a 1.0 M solution of trimethylphosphane in toluene (0.8 mL, 0.8 mmol) was added to an ice-cooled mixture of quinaldic acid (51 mg, 0.29 mmol), (*R*)-**28** (84 mg, 0.23 mmol), and 2,2'-dithiodipyridine (36 mg, 0.16 mmol). After stirring the reaction mixture for 20 min at 0 °C, the ice-bath was removed and the mixture was stirred for 24 h at ambient temperature. Then, water (1.5 mL) was added and the mixture was stirred for 20 min. Afterwards, the mixture was diluted with dichloromethane and washed with a saturated aqueous solution of $NaHCO_3$, an ice-cold solution of 1.0 M HCl, and water. The combined organic layers were dried (Na_2SO_4), filtered, and the solvent was removed *in vacuo*. The residue was purified by flash column chromatography (Ø = 3 cm, h = 19 cm, V = 20 mL, petroleum ether/ethyl acetate = 2/1, R_f = 0.27) to give (*R*)-**38e** as colorless solid (88 mg, 0.18 mmol, 77%). m.p.: 75 °C; $[\alpha]_D^{20}$ = +20.0 (2.5, methanol); HPLC (method 1): t_R = 25.0 min, purity 99.4%.

Spectroscopic data of (*S*)-**38e** *and* (*R*)-**38e**:

^{1}H NMR (DMSO-d_6): δ [ppm] = 3.62 – 3.68 (m, 1H, OCHCH_2NH), 3.69 (s, 3H, CO_2CH_3), 3.78 (ddd, J = 13.6/6.1/5.0 Hz, 1H, OCHCH_2NH), 4.31 (dd, J = 7.3/4.9 Hz, 1H, OCHCH$_2$NH), 4.45 (d, J = 12.3 Hz, 1H, OCH_2Ar), 4.63 (d, J = 12.3 Hz, 1H, OCH_2Ar), 7.13 – 7.19 (m, 2H, 2'-$H_{4\text{-iodophenyl}}$, 6'-$H_{4\text{-iodophenyl}}$), 7.53 – 7.60 (m, 2H, 3'-$H_{4\text{-iodophenyl}}$, 5'-$H_{4\text{-iodophenyl}}$), 7.72 – 7.76 (m, 1H, 6''-$H_{quinoline}$), 7.88 – 7.92 (m, 1H, 7''-$H_{quinoline}$), 8.08 – 8.11 (m, 1H, 5''-$H_{quinoline}$), 8.11 – 8.15 (m, 2H, 3''-$H_{quinoline}$, 8''-$H_{quinoline}$), 8.58 (d, J = 8.4 Hz, 1H, 4''-$H_{quinoline}$), 8.96 (t, J = 6.2 Hz, 1H, CONH); ^{13}C NMR (DMSO-d_6): δ [ppm] = 41.0 (1C, OCHCH$_2$NH), 52.0 (1C, CO_2CH_3), 70.7 (1C, OCH$_2$Ar), 76.4

(1C, O*C*HCH$_2$NH), 93.6 (1C, C-4'$_{4\text{-iodophenyl}}$), 118.6 (1C, C-3''$_{\text{quinoline}}$), 128.15 (1C, C$_{\text{arom.}}$), 128.18 (1C, C$_{\text{arom.}}$), 128.9 (1C, C-4a''$_{\text{quinoline}}$), 129.2 (1C, C-8''$_{\text{quinoline}}$), 129.9 (2C, C-2'$_{4\text{-iodophenyl}}$, C-6'$_{4\text{-iodophenyl}}$), 130.6 (1C, C-7''$_{\text{quinoline}}$), 136.9 (2C, C-3'$_{4\text{-iodophenyl}}$, C-5'$_{4\text{-iodophenyl}}$), 137.5 (1C, C-1'$_{4\text{-iodophenyl}}$), 138.0 (1C, C-4''$_{\text{quinoline}}$), 146.0 (1C, C-8a''$_{\text{quinoline}}$), 149.7 (1C, C-2''$_{\text{quinoline}}$), 164.1 (1C, *C*ONH), 170.9 (1C, *C*O$_2$CH$_3$); IR (neat): $\tilde{\nu}$ [cm^{-1}] = 3362, 2873, 1749, 1665, 1524, 1501, 1456, 1429, 1381, 1203, 1132, 1005, 845, 772, 733, 676,631, 469; HRMS (*m*/*z*): [M+H]$^+$ calcd for $C_{21}H_{20}IN_2O_4$: 491.0462, found: 491.0465.

Methyl (*S*)-2-[(4-iodobenzyl)oxy]-3-(quinoline-3-carboxamido)propanoate ((*S*)-38f)

Under N_2 atmosphere, a 1.0 M solution of trimethylphosphane in toluene (2.6 mL, 2.6 mmol) was added to an ice-cooled mixture of quinoline-3-carboxylic acid (180 mg, 1.0 mmol), (*S*)-**28** (340 mg, 0.93 mmol), and 2,2'-dithiodipyridine (100 mg, 0.46 mmol). After stirring the reaction mixture for 20 min at 0 °C, the ice-bath was removed and the mixture was stirred for 36 h at ambient temperature. Then, water (1.5 mL) was added and the mixture was stirred for 20 min. Afterwards, the mixture was diluted with dichloromethane and washed with a saturated aqueous solution of $NaHCO_3$, an ice-cold solution of 1.0 M HCl, and water. The combined organic layers were dried (Na_2SO_4), filtered, and the solvent was removed *in vacuo*. The residue was purified by flash column chromatography (Ø = 4 cm, h = 23 cm, V = 30 mL, 100% ethyl acetate, R_f = 0.53) to give (*S*)-**38f** as yellow solid (370 mg, 0.76 mmol, 82%). m.p.: 99 °C; $[\alpha]_D^{20}$ = -14.2 (3.7, methanol); HPLC (method 1): t_R = 20.3 min, purity 98.7%.

Methyl (*R*)-2-[(4-iodobenzyl)oxy]-3-(quinoline-3-carboxamido)propanoate ((*R*)-38f)

Under N_2 atmosphere, a 1.0 M solution of trimethylphosphane in toluene (2.1 mL, 2.1 mmol) was added to an ice-cooled mixture of quinoline-3-carboxylic acid (170 mg, 0.98 mmol), (*R*)-**28** (320 mg, 0.88 mmol), and 2,2'-dithiodipyridine (96 mg, 0.44 mmol). After stirring the reaction mixture for 20 min at 0 °C, the ice-bath was removed and the mixture was stirred for 36 h at ambient temperature. Then, water (1.5 mL) was added and the mixture was stirred for 20 min. Afterwards, the mixture was diluted with dichloromethane and washed with a saturated aqueous solution of $NaHCO_3$, an ice-cold solution of 1.0 M HCl, and water. The combined organic layers were dried (Na_2SO_4), filtered, and the solvent was removed *in vacuo*. The residue was purified by flash column chromatography (Ø = 4 cm, h = 20 cm, V = 30 mL, 100% ethyl acetate, R_f = 0.53) to give (*R*)-**38f** as colorless solid (320 mg, 0.65 mmol, 73%). m.p.: 99 °C; $[\alpha]_D^{20}$ = +16.4 (1.1, methanol); HPLC (method 1): t_R = 20.3 min, purity 99.6%.

Spectroscopic data of (*S*)-**38f** *and* (*R*)-**38f**:

^{1}H NMR (DMSO-d_6): δ [ppm] = 3.53 – 3.62 (m, 1H, $OCHCH_2NH$), 3.69 – 3.77 (m, 1H, $OCHCH_2NH$), 3.70 (s, 3H, CO_2CH_3), 4.25 (dd, *J* = 7.3/4.9 Hz, 1H, $OCHCH_2NH$), 4.44 (d, *J* = 12.2 Hz, 1H, OCH_2Ar), 4.61 (d, *J* = 12.2 Hz, 1H, OCH_2Ar), 7.11 – 7.22 (m, 2H, 2'-$H_{4\text{-iodophenyl}}$, 6'-$H_{4\text{-iodophenyl}}$), 7.56 – 7.65 (m, 2H, 3'-$H_{4\text{-iodophenyl}}$, 5'-$H_{4\text{-iodophenyl}}$), 7.67 – 7.74 (m, 1H, 6''-$H_{quinoline}$), 7.84 – 7.91 (m, 1H, 7''-$H_{quinoline}$), 8.05 – 8.16 (m, 2H, 5''-$H_{quinoline}$, 8''-$H_{quinoline}$), 8.77 (d, *J* = 2.0 Hz, 1H, 4''-$H_{quinoline}$), 9.05 (t, *J* = 5.9 Hz, 1H, CON*H*), 9.24 (d, *J* = 2.2 Hz, 1H, 2''-$H_{quinoline}$); ^{13}C NMR (DMSO-d_6): δ [ppm] = 41.4 (1C, $OCHCH_2NH$), 52.0 (1C, CO_2CH_3), 70.8 (1C, OCH_2Ar), 76.4 (1C, $OCHCH_2NH$), 93.6 (1C, C-4'$_{4\text{-iodophenyl}}$), 126.5 (1C, C-4a''$_{quinoline}$), 126.8 (1C, C-3''$_{quinoline}$), 127.5 (1C, C-

6''$_{quinoline}$), 128.8 (1C, C-8''$_{quinoline}$), 129.2 (1C, C-5''$_{quinoline}$), 130.0 (2C, C-2'$_{4\text{-iodophenyl}}$, C-6'$_{4\text{-iodophenyl}}$), 131.3 (1C, C-7''$_{quinoline}$), 135.6 (1C, C-4''$_{quinoline}$), 136.9 (2C, C-3'$_{4\text{-iodophenyl}}$, C-5'$_{4\text{-iodophenyl}}$), 137.5 (1C, C-1'$_{4\text{-iodophenyl}}$), 148.5 (1C, C-8a''$_{quinoline}$), 148.8 (1C, C-2''$_{quinoline}$), 165.2 (1C, *C*ONH), 170.9 (1C, *C*O_2CH_3); IR (neat): $\tilde{\nu}$ [cm^{-1}] = 3258, 3049, 2937, 1736, 1634, 1541, 1428, 1300, 1201, 1097, 1002, 801, 784, 758, 740, 695, 627, 483; HRMS (*m*/*z*): $[M+H]^+$ calcd for $C_{21}H_{20}IN_2O_4$: 491.0462, found: 491.0496.

Methyl (*S*)-3-(1*H*-indole-2-carboxamido)-2-[(4-iodobenzyl)oxy]propanoate ((*S*)-38g)

Under N_2 atmosphere, a 1.0 M solution of trimethylphosphane in toluene (2.3 mL, 2.3 mmol) was added to an ice-cooled mixture of indole-2-carboxylic acid (180 mg, 1.1 mmol), (*S*)-**28** (350 mg, 0.98 mmol), and 2,2'-dithiodipyridine (110 mg, 0.52 mmol). After stirring the reaction mixture for 20 min at 0 °C, the ice-bath was removed and the mixture was stirred for 4 d at ambient temperature. Then, water (1.5 mL) was added and the mixture was stirred for 20 min. Afterwards, the mixture was diluted with dichloromethane and washed with a saturated aqueous solution of $NaHCO_3$, an ice-cold solution of 1.0 M HCl, and water. The combined organic layers were dried (Na_2SO_4), filtered, and the solvent was removed *in vacuo*. The residue was purified by flash column chromatography (Ø = 4 cm, h = 24 cm, V = 30 mL, petroleum ether/ethyl acetate = 2/1, R_f = 0.22) to give (*S*)-**38g** as orange solid (360 mg, 0.75 mmol, 76%). m.p.: 120 °C; $[\alpha]_D^{20}$ = -15.3 (1.9, methanol); HPLC (method 1): t_R = 23.6 min, purity 99.1%.

Methyl (*R*)-3-(1*H*-indole-2-carboxamido)-2-[(4-iodobenzyl)oxy]propanoate ((*R*)-38g)

Under N_2 atmosphere, a 1.0 M solution of trimethylphosphane in toluene (2.3 mL, 2.3 mmol) was added to an ice-cooled mixture of indole-2-carboxylic acid (180 mg, 1.1 mmol), (*R*)-**28** (350 mg, 0.96 mmol), and 2,2'-dithiodipyridine (110 mg, 0.52 mmol). After stirring the reaction mixture for 20 min at 0 °C, the ice-bath was removed and the mixture was stirred for 4 d at ambient temperature. Then, water (1.0 mL) was added and the mixture was stirred for 20 min. Afterwards, the mixture was diluted with dichloromethane and washed with a saturated aqueous solution of $NaHCO_3$, an ice-cold solution of 1.0 M HCl, and water. The combined organic layers were dried (Na_2SO_4), filtered, and the solvent was removed *in vacuo*. The residue was purified by flash column chromatography (Ø = 4 cm, h = 19 cm, V = 30 mL, petroleum ether/ethyl acetate = 2/1 → 1/1) to give (*R*)-**38g** as orange solid (350 mg, 0.74 mmol, 77%). m.p.: 120 °C; R_f = 0.22 (petroleum ether/ethyl acetate = 2/1); $[\alpha]_D^{20}$ = +17.5 (2.9, methanol); HPLC (method 1): t_R = 23.5 min, purity 99.3%.

Spectroscopic data of (*S*)-**38g** *and* (*R*)-**38g**:

^{1}H NMR (DMSO-*d*$_6$): δ [ppm] = 3.51 – 3.59 (m, 1H, OCHC*H*$_2$NH), 3.64 – 3.72 (m, 4H, OCHC*H*$_2$NH (1H), CO_2CH_3), 4.21 (dd, *J* = 7.2/5.0 Hz, 1H, OC*H*CH$_2$NH), 4.44 (d, *J* = 12.3 Hz, 1H, OC*H*$_2$Ar), 4.60 (d, *J* = 12.3 Hz, 1H, OC*H*$_2$Ar), 7.01 – 7.06 (m, 1H, 5''-H_{indole}), 7.12 – 7.21 (m, 4H, 2'-$H_{4\text{-iodophenyl}}$, 6'-$H_{4\text{-iodophenyl}}$, 3''-H_{indole}, 6''-H_{indole}), 7.41 – 7.45 (m, 1H, 7''-H_{indole}), 7.60 – 7.64 (m, 3H, 3'-$H_{4\text{-iodophenyl}}$, 5'-$H_{4\text{-iodophenyl}}$, 4''-H_{indole}), 8.66 (t, *J* = 6.0 Hz, 1H, CON*H*), 11.53 – 11.59 (m, 1H, 1''-H_{indole}); ^{13}C NMR (DMSO-*d*$_6$): δ [ppm] = 41.0 (1C, OCH*C*H$_2$NH), 51.9 (1C, CO_2CH_3), 70.7 (1C, O*C*H$_2$Ar), 76.7 (1C, O*C*HCH$_2$NH), 93.5 (1C, C-4'$_{4\text{-iodophenyl}}$), 102.8 (1C, C-3''$_{indole}$), 112.3 (1C, C-7''$_{indole}$),

119.7 (1C, C-5''$_{indole}$), 121.5 (1C, C-4''$_{indole}$), 123.3 (1C, C-6''$_{indole}$), 127.0 (1C, C-3a''$_{indole}$), 129.9 (2C, C-2'$_{4\text{-iodophenyl}}$, C-6'$_{4\text{-iodophenyl}}$), 131.3 (1C, C-2''$_{indole}$), 136.5 (1C, C-7a''$_{indole}$), 136.9 (2C, C-3'$_{4\text{-iodophenyl}}$, C-5'$_{4\text{-iodophenyl}}$), 137.5 (1C, C-1'$_{4\text{-iodophenyl}}$), 161.3 (1C, *C*ONH), 170.9 (1C, *C*O_2CH_3); IR (neat): $\tilde{\nu}$ [cm^{-1}] = 3277, 1744, 1641, 1542, 1421, 1341, 1308, 1244, 1203, 1135, 1006, 802, 773, 745, 480, 433, 412; HRMS (*m*/*z*): $[M+Na]^+$ calcd for $C_{20}H_{19}IN_2NaO_4$: 501.0282, found: 501.0254.

Methyl (*S*)-3-(1*H*-indole-3-carboxamido)-2-[(4-iodobenzyl)oxy]propanoate ((*S*)-38h)

Under N_2 atmosphere, a 1.0 M solution of trimethylphosphane in toluene (2.4 mL, 2.4 mmol) was added to an ice-cooled mixture of indole-3-carboxylic acid (180 mg, 1.1 mmol), (*S*)-**28** (340 mg, 0.94 mmol), and 2,2'-dithiodipyridine (110 mg, 0.49 mmol). After stirring the reaction mixture for 20 min at 0 °C, the ice-bath was removed and the mixture was stirred for 7 d at ambient temperature. Then, water (1.5 mL) was added and the mixture was stirred for 20 min. Afterwards, the mixture was diluted with dichloromethane and washed with a saturated aqueous solution of $NaHCO_3$, an ice-cold solution of 1.0 M HCl, and water. The combined organic layers were dried (Na_2SO_4), filtered, and the solvent was removed *in vacuo*. The residue was purified by flash column chromatography (Ø = 4 cm, h = 24 cm, V = 30 mL, dichloromethane/isopropanol = 40/1 → 20/1) to give (*S*)-**38h** as yellow solid (300 mg, 0.62 mmol, 65%). m.p.: 154 °C; R_f = 0.33 (dichloromethane/isopropanol = 40/1); $[\alpha]_D^{20}$ = -39.7 (2.2, methanol); HPLC (method 1): t_R = 22.0 min, purity 97.2%.

Methyl (*R*)-3-(1*H*-indole-3-carboxamido)-2-[(4-iodobenzyl)oxy]propanoate ((*R*)-38h)

Under N_2 atmosphere, a 1.0 M solution of trimethylphosphane in toluene (2.3 mL, 2.3 mmol) was added to an ice-cooled mixture of indole-3-carboxylic acid (190 mg, 1.2 mmol), (*R*)-**28** (340 mg, 0.94 mmol), and 2,2'-dithiodipyridine (170 mg, 0.75 mmol). After stirring the reaction mixture for 20 min at 0 °C, the ice-bath was removed and the mixture was stirred for 7 d at ambient temperature. Then, water (1.0 mL) was added and the mixture was stirred for 20 min. Afterwards, the mixture was diluted with dichloromethane and washed with a saturated aqueous solution of $NaHCO_3$, an ice-cold solution of 1.0 M HCl, and water. The combined organic layers were dried (Na_2SO_4), filtered, and the solvent was removed *in vacuo*. The residue was purified by flash column chromatography (Ø = 4 cm, h = 25 cm, V = 30 mL, dichloromethane/isopropanol = 40/1 → 20/1) to give (*R*)-**38h** as yellow solid (330 mg, 0.69 mmol, 73%). m.p.: 154 °C; R_f = 0.33 (dichloromethane/isopropanol = 40/1); $[\alpha]_D^{20}$ = +42.9 (1.4, methanol); HPLC (method 1): t_R = 22.0 min, purity 97.6%.

Spectroscopic data of (*S*)-**38h** *and* (*R*)-**38h**:

^{1}H NMR (DMSO-d_6): δ [ppm] = 3.44 – 3.52 (m, 1H, OCHCH_2NH), 3.62 – 3.71 (m, 4H, OCHCH_2NH (1H), CO_2CH_3), 4.21 (dd, *J* = 7.2/5.1 Hz, 1H, OC*H*CH$_2$NH), 4.43 (d, *J* = 12.3 Hz, 1H, OCH_2Ar), 4.59 (d, *J* = 12.3 Hz, 1H, OCH_2Ar), 7.06 – 7.19 (m, 4H, 2'-$H_{4\text{-iodophenyl}}$, 6'-$H_{4\text{-iodophenyl}}$, 5''-H_{indole}, 6''-H_{indole}), 7.40 – 7.45 (m, 1H, 7''-H_{indole}), 7.59 – 7.65 (m, 2H, 3'-$H_{4\text{-iodophenyl}}$, 5'-$H_{4\text{-iodophenyl}}$), 8.01 (d, *J* = 2.9 Hz, 1H, 2''-H_{indole}), 8.06 (t, *J* = 6.0 Hz, 1H, CON*H*), 8.08 – 8.13 (m, 1H, 4''-H_{indole}), 11.49 – 11.58 (m, 1H, 1''-H_{indole}); ^{13}C NMR (DMSO-d_6): δ [ppm] = 40.8 (1C, OCH*C*H$_2$NH), 51.8 (1C, CO_2CH_3), 70.7 (1C, O*C*H$_2$Ar), 77.1 (1C, O*C*HCH$_2$NH), 93.5 (1C, C-4'$_{4\text{-iodophenyl}}$), 110.3 (1C, C-3''$_{indole}$), 111.8

(1C, C-7''$_{indole}$), 120.3 (1C, C-5''$_{indole}$), 120.9 (1C, C-4''$_{indole}$), 121.8 (1C, C-6''$_{indole}$), 126.1 (1C, C-3a''$_{indole}$), 127.9 (1C, C-2''$_{indole}$), 129.9 (2C, C-2'$_{4\text{-iodophenyl}}$, C-6'$_{4\text{-iodophenyl}}$), 136.1 (1C, C-7a''$_{indole}$), 136.9 (2C, C-3'$_{4\text{-iodophenyl}}$, C-5'$_{4\text{-iodophenyl}}$), 137.6 (1C, C-1'$_{4\text{-iodophenyl}}$), 164.7 (1C, *C*ONH), 171.2 (1C, *C*O_2CH_3); IR (neat): $\tilde{\nu}$ [cm^{-1}] = 3377, 3115, 2947, 2920, 2865, 1739, 1598, 1539, 1433, 1318, 1199, 1093, 1005, 796, 749, 570, 506, 483, 433, 402; HRMS (*m/z*): $[M+H]^+$ calcd for $C_{20}H_{20}IN_2O_4$: 479.0462, found: 479.0469.

Methyl (*S*)-3-(1*H*-indole-4-carboxamido)-2-[(4-iodobenzyl)oxy]propanoate ((*S*)-38i)

Under N_2 atmosphere, a 1.0 M solution of trimethylphosphane in toluene (2.4 mL, 2.4 mmol) was added to an ice-cooled mixture of indole-4-carboxylic acid (180 mg, 1.1 mmol), (*S*)-**28** (360 mg, 1.0 mmol), and 2,2'-dithiodipyridine (110 mg, 0.50 mmol). After stirring the reaction mixture for 20 min at 0 °C, the ice-bath was removed and the mixture was stirred for 5 d at ambient temperature. Then, water (1.5 mL) was added and the mixture was stirred for 20 min. Afterwards, the mixture was diluted with dichloromethane and washed with a saturated aqueous solution of $NaHCO_3$, an ice-cold solution of 1.0 M HCl, and water. The combined organic layers were dried (Na_2SO_4), filtered, and the solvent was removed *in vacuo*. The residue was purified by flash column chromatography (Ø = 4 cm, h = 25 cm, V = 30 mL, petroleum ether/ethyl acetate = 1/1, R_f = 0.29) to give (*S*)-**38i** as yellow solid (310 mg, 0.65 mmol, 64%). m.p.: 119 °C; $[\alpha]_D^{20}$ = -38.1 (2.0, methanol); ^1H NMR (DMSO-d_6): δ [ppm] = 3.55 – 3.62 (m, 1H, OCHCH_2NH), 3.65 – 3.71 (m, 4H, OCHCH_2NH (1H), CO_2CH_3), 4.27 (dd, *J* = 6.9/5.3 Hz, 1H, OC*H*CH_2NH), 4.45 (d, *J* = 12.2 Hz, 1H, OCH_2Ar), 4.60 (d, *J* = 12.2 Hz, 1H, OCH_2Ar), 6.80 – 6.83 (m, 1H, 3''-H$_{indole}$), 7.11 – 7.15 (m, 1H, 6''-H$_{indole}$), 7.16 – 7.19 (m, 2H, 2'-H$_{4\text{-iodophenyl}}$, 6'-H$_{4\text{-iodophenyl}}$), 7.35 – 7.38 (m, 1H, 5''-H$_{indole}$), 7.42 (t, *J* = 2.8 Hz,

1H, 2''-H_{indole}), 7.52 – 7.56 (m, 1H, 7''-H_{indole}), 7.62 – 7.67 (m, 2H, 3'-$H_{4\text{-iodophenyl}}$, 5'-$H_{4\text{-iodophenyl}}$), 8.30 (t, *J* = 5.9 Hz, 1H, CON*H*), 11.28 (s br, 1H, 1''-H_{indole}); ^{13}C NMR (DMSO-*d*$_6$): δ [ppm] = 41.2 (1C, OCH*C*H_2NH), 51.9 (1C, CO_2*C*H_3), 70.6 (1C, O*C*H_2Ar), 76.8 (1C, O*C*HCH_2NH), 93.5 (1C, C-4'$_{4\text{-iodophenyl}}$), 101.7 (1C, C-3''$_{indole}$), 114.2 (1C, C-7''$_{indole}$), 118.5 (1C, C-5''$_{indole}$), 120.1 (1C, C-6''$_{indole}$), 125.8 (1C, C-3a''$_{indole}$), 126.3 (1C, C-4''$_{indole}$), 126.4 (1C, C-2''$_{indole}$), 129.9 (2C, C-2'$_{4\text{-iodophenyl}}$, C-6'$_{4\text{-iodophenyl}}$), 136.5 (1C, C-7a''$_{indole}$), 136.9 (2C, C-3'$_{4\text{-iodophenyl}}$, C-5'$_{4\text{-iodophenyl}}$), 137.6 (1C, C-1'$_{4\text{-iodophenyl}}$), 168.0 (1C, *C*ONH), 171.1 (1C, *C*O_2CH_3); IR (neat): $\tilde{\nu}$ [cm^{-1}] = 3379, 2945, 2863, 1726, 1637, 1605, 1514, 1494, 1441, 1341, 1285, 1219, 1098, 1006, 797, 764, 651, 549, 455; HRMS (*m*/*z*): $[M+H]^+$ calcd for $C_{20}H_{20}IN_2O_4$: 479.0462, found: 479.0456; HPLC (method 1): t_R = 21.9 min, purity 99.1%.

Methyl (*S*)-3-(1*H*-indole-5-carboxamido)-2-[(4-iodobenzyl)oxy]propanoate ((*S*)-38j)

Under N_2 atmosphere, a 1.0 M solution of trimethylphosphane in toluene (2.4 mL, 2.4 mmol) was added to an ice-cooled mixture of indole-5-carboxylic acid (170 mg, 1.0 mmol), (*S*)-**28** (340 mg, 0.95 mmol), and 2,2'-dithiodipyridine (110 mg, 0.48 mmol). After stirring the reaction mixture for 20 min at 0 °C, the ice-bath was removed and the mixture was stirred for 7 d at ambient temperature. Then, water (2.0 mL) was added and the mixture was stirred for 20 min. Afterwards, the mixture was diluted with dichloromethane and washed with a saturated aqueous solution of $NaHCO_3$, an ice-cold solution of 1.0 M HCl, and water. The combined organic layers were dried (Na_2SO_4), filtered, and the solvent was removed *in vacuo*. The residue was dissolved in a mixture of petroleum ether and ethyl acetate (1/2) and filtered through a short silica gel column. The solvent was removed *in vacuo* and the residue was purified by

automatic flash column chromatography using a Biotage® Isolera™ One system (10% → 80% ACN in H_2O, Biotage® SNAP Ultra C18 30 g). Fractions containing the desired product were combined and subjected to lyophilization to give (*S*)-**38j** as yellow solid (150 mg, 0.32 mmol, 34%). m.p.: 61 °C; R_f = 0.53 (petroleum ether/ethyl acetate = 1/2); $[\alpha]_D^{20}$ = -22.4 (1.7, methanol); HPLC (method 1): t_R = 21.8 min, purity 99.8%.

Methyl (*R*)-3-(1*H*-indole-5-carboxamido)-2-[(4-iodobenzyl)oxy]propanoate ((*R*)-38j)

Under N_2 atmosphere, a 1.0 M solution of trimethylphosphane in toluene (2.24 mL, 2.2 mmol) was added to an ice-cooled mixture of indole-5-carboxylic acid (170 mg, 1.0 mmol), (*R*)-**28** (330 mg, 0.92 mmol), and 2,2'-dithiodipyridine (100 mg, 0.46 mmol). After stirring the reaction mixture for 20 min at 0 °C, the ice-bath was removed and the mixture was stirred for 5 d at ambient temperature. Then, water (1.0 mL) was added and the mixture was stirred for 20 min. Afterwards, the mixture was diluted with dichloromethane and washed with a saturated aqueous solution of $NaHCO_3$, an ice-cold solution of 1.0 M HCl, and water. The combined organic layers were dried (Na_2SO_4), filtered, and the solvent was removed *in vacuo*. The residue was purified by flash column chromatography (Ø = 4 cm, h = 27 cm, V = 30 mL, petroleum ether/ethyl acetate = 1/2, R_f = 0.53) to give (*R*)-**38j** as yellow solid (200 mg, 0.42 mmol, 45%). m.p.: 63 °C; $[\alpha]_D^{20}$ = +23.3 (1.2, methanol); HPLC (method 1): t_R = 21.8 min, purity 98.0%.

Spectroscopic data of (*S*)-**38j** *and* (*R*)-**38j**:

^{1}H NMR (DMSO-d_6): δ [ppm] = 3.48 – 3.55 (m, 1H, OCHCH_2NH), 3.65 (dt, *J* = 13.5/5.5 Hz, 1H, OCHCH_2NH), 3.67 (s, 3H, CO_2CH_3), 4.22 (dd, *J* = 7.3/5.2 Hz, 1H, OC*H*CH_2NH), 4.43 (d, *J* = 12.3 Hz, 1H, OCH_2Ar), 4.58 (d, *J* = 12.3 Hz, 1H, OCH_2Ar), 6.51 – 6.55 (m, 1H, 3''-H_{indole}), 7.13 – 7.17 (m, 2H, 2'-$H_{4\text{-iodophenyl}}$, 6'-$H_{4\text{-iodophenyl}}$), 7.40 – 7.44 (m, 2H, 2''-H_{indole}, 7''-H_{indole}), 7.59 (dd, *J* = 8.5/1.6 Hz, 1H, 6''-H_{indole}), 7.61 – 7.64 (m, 2H, 3'-$H_{4\text{-iodophenyl}}$, 5'-$H_{4\text{-iodophenyl}}$), 8.08 – 8.10 (m, 1H, 4''-H_{indole}), 8.48 (t, *J* = 5.9 Hz, 1H, CON*H*), 11.32 (s br, 1H, 1''-H_{indole}); ^{13}C NMR (DMSO-d_6): δ [ppm] = 41.4 (1C, OCH*C*H_2NH), 51.9 (1C, CO_2CH_3), 70.6 (1C, O*C*H_2Ar), 76.7 (1C, O*C*HCH_2NH), 93.5 (1C, C-4'$_{4\text{-iodophenyl}}$), 102.1 (1C, C-3''$_{indole}$), 110.9 (1C, C-7''$_{indole}$), 120.0 (1C, C-4''$_{indole}$), 120.5 (1C, C-6''$_{indole}$), 125.2 (1C, C-5''$_{indole}$), 126.7 (1C, C-2''$_{indole}$), 127.0 (1C, C-3a''$_{indole}$), 129.9 (2C, C-2'$_{4\text{-iodophenyl}}$, C-6'$_{4\text{-iodophenyl}}$), 136.9 (2C, C-3'$_{4\text{-iodophenyl}}$, C-5'$_{4\text{-iodophenyl}}$), 137.4 (1C, C-7a''$_{indole}$), 137.6 (1C, C-1'$_{4\text{-iodophenyl}}$), 167.6 (1C, *C*ONH), 171.1 (1C, *C*O_2CH_3); IR (neat): $\tilde{\nu}$ [cm^{-1}] = 3270, 2925, 2851, 1735, 1634, 1610, 1525, 1302, 1202, 1115, 1006, 894, 799, 752, 726, 473, 423; HRMS (*m*/*z*): [M+H]$^+$ calcd for $C_{20}H_{20}IN_2O_4$: 479.0462, found: 479.0470.

Methyl (*S*)-2-[(4-iodobenzyl)oxy]-3-(1*H*-pyrrole-2-carboxamido)propanoate ((*S*)-38k)

Under N_2 atmosphere, a 1.0 M solution of trimethylphosphane in toluene (2.0 mL, 2.0 mmol) was added to an ice-cooled mixture of pyrrole-2-carboxylic acid (100 mg, 0.92 mmol), (*S*)-**28** (300 mg, 0.83 mmol), and 2,2'-dithiodipyridine (66 mg, 0.30 mmol). After stirring the reaction mixture for 20 min at 0 °C, the ice-bath was removed and the mixture was stirred for 48 h at ambient temperature. Then, water (2.0 mL) was added and the mixture was stirred for 20 min. Afterwards, the mixture was diluted with

dichloromethane and washed with a saturated aqueous solution of $NaHCO_3$, an ice-cold solution of 1.0 M HCl, and water. The combined organic layers were dried (Na_2SO_4), filtered, and the solvent was removed *in vacuo*. The residue was purified by flash column chromatography (Ø = 3 cm, h = 20 cm, V = 20 mL, petroleum ether/ethyl acetate = 1/1, R_f = 0.32) to give (*S*)-**38k** as brown oil (120 mg, 0.27 mmol, 33%). $[\alpha]_D^{20}$ = -52.6 (1.3, methanol); ^{1}H NMR (DMSO-d_6): δ [ppm] = 3.39 – 3.49 (m, 1H, OCHCH_2NH), 3.56 – 3.64 (m, 1H, OCHCH_2NH), 3.67 (s, 3H, CO_2CH_3), 4.14 (dd, *J* = 7.2/5.0 Hz, 1H, OC*H*CH_2NH), 4.41 (d, *J* = 12.3 Hz, 1H, OCH_2Ar), 4.58 (d, *J* = 12.3 Hz, 1H, OCH_2Ar), 6.06 – 6.10 (m, 1H, 4''-$H_{pyrrole}$), 6.75 – 6.81 (m, 1H, 3''-$H_{pyrrole}$), 6.82 – 6.88 (m, 1H, 5''-$H_{pyrrole}$), 7.11 – 7.18 (m, 2H, 2'-$H_{4\text{-iodophenyl}}$, 6'-$H_{4\text{-iodophenyl}}$), 7.59 – 7.66 (m, 2H, 3'-$H_{4\text{-iodophenyl}}$, 5'-$H_{4\text{-iodophenyl}}$), 8.16 (t, *J* = 6.0 Hz, 1H, CON*H*), 11.42 (s br, 1H, 1''-$H_{pyrrole}$); ^{13}C NMR (DMSO-d_6): δ [ppm] = 40.8 (1C, OCH*C*H_2NH), 51.9 (1C, CO_2CH_3), 70.6 (1C, O*C*H_2Ar), 76.8 (1C, O*C*HCH_2NH), 93.5 (1C, C-4'$_{4\text{-iodophenyl}}$), 108.5 (1C, C-4''$_{pyrrole}$), 110.2 (1C, C-3''$_{pyrrole}$), 121.4 (1C, C-5''$_{pyrrole}$), 126.0 (1C, C-2''$_{pyrrole}$), 129.8 (2C, C-2'$_{4\text{-iodophenyl}}$, C-6'$_{4\text{-iodophenyl}}$), 136.9 (2C, C-3'$_{4\text{-iodophenyl}}$, C-5'$_{4\text{-iodophenyl}}$), 137.5 (1C, C-1'$_{4\text{-iodophenyl}}$), 160.8 (1C, *C*ONH), 171.0 (1C, *C*O_2CH_3); IR (neat): $\tilde{\nu}$ [cm^{-1}] = 3254, 2949, 2870, 1738, 1628, 1558, 1516, 1483, 1433, 1406, 1321, 1200, 1115, 1035, 1005, 834, 793, 738, 605, 507, 473; HRMS (*m/z*): $[M+Na]^+$ calcd for $C_{16}H_{17}IN_2NaO_4$: 451.0125, found: 451.0116; HPLC (method 1): t_R = 21.5 min, purity 98.3%.

Methyl (*S*)-2-[(4-iodobenzyl)oxy]-3-(1*H*-pyrazole-3-carboxamido)propanoate ((*S*)-38l)

Under N_2 atmosphere, a 1.0 M solution of trimethylphosphane in toluene (1.4 mL, 1.4 mmol) was added to an ice-cooled mixture of 1*H*-pyrazole-3-carboxylic acid (82 mg, 0.73 mmol), (*S*)-**28** (210 mg, 0.57 mmol), and 2,2'-dithiodipyridine (76 mg,

0.34 mmol). After stirring the reaction mixture for 20 min at 0 °C, the ice-bath was removed and the mixture was stirred for 5 d at ambient temperature. Then, water (2.0 mL) was added and the mixture was stirred for 20 min. Afterwards, the mixture was diluted with dichloromethane and washed with a saturated aqueous solution of $NaHCO_3$, an ice-cold solution of 1.0 M HCl, and water. The combined organic layers were dried (Na_2SO_4), filtered, and the solvent was removed *in vacuo*. The residue was purified by flash column chromatography (Ø = 3 cm, h = 20 cm, V = 18 mL, petroleum ether/ethyl acetate = 1/2 → 1/4) to give (*S*)-**38l** as yellow oil (120 mg, 0.29 mmol, 50%). R_f = 0.33 (petroleum ether/ethyl acetate = 1/4); $[\alpha]_D^{20}$ = -31.4 (1.1, methanol); ^{1}H NMR (DMSO-d_6): δ [ppm] = 3.46 – 3.55 (m, 1H, OCHCH_2NH), 3.57 – 3.65 (m, 1H, OCHCH_2NH), 3.66 (s, 3H, CO_2CH_3), 4.20 (dd, *J* = 6.9/5.1 Hz, 1H, OC*H*CH_2NH), 4.42 (d, *J* = 12.3 Hz, 1H, OCH_2Ar), 4.58 (d, *J* = 12.3 Hz, 1H, OCH_2Ar), 6.65 (s br, 1H, 4''-$H_{pyrazole}$), 7.11 – 7.19 (m, 2H, 2'-$H_{4\text{-iodophenyl}}$, 6'-$H_{4\text{-iodophenyl}}$), 7.60 – 7.68 (m, 2H, 3'-$H_{4\text{-iodophenyl}}$, 5'-$H_{4\text{-iodophenyl}}$), 7.73 – 7.88 (m, 1H, 5''-$H_{pyrazole}$), 8.16 (s br, 1H, CON*H*), 13.26 (s br, 1H, 1''-$H_{pyrazole}$); ^{13}C NMR (DMSO-d_6): δ [ppm] = 40.5 (1C, OCH*C*H_2NH), 51.9 (1C, CO_2CH_3), 70.6 (1C, O*C*H_2Ar), 76.6 (1C, O*C*HCH_2NH), 93.5 (1C, C-4'$_{4\text{-iodophenyl}}$), 105.0 (1C, C-4''$_{pyrazole}$), 129.8 (2C, C-2'$_{4\text{-iodophenyl}}$, C-6'$_{4\text{-iodophenyl}}$), 136.9 (2C, C-3'$_{4\text{-iodophenyl}}$, C-5'$_{4\text{-iodophenyl}}$), 137.5 (1C, C-1'$_{4\text{-iodophenyl}}$), 170.9 (1C, *C*O_2CH_3), the signals for C-3''$_{pyrazole}$, C-5''$_{pyrazole}$, and *C*ONH cannot be observed in the spectrum; IR (neat): $\tilde{\nu}$ [cm^{-1}] = 3214, 2951, 1734, 1647, 1543, 1206, 1117, 1006, 795, 761, 613, 508, 474; HRMS (*m/z*): $[M+H]^+$ calcd for $C_{15}H_{17}IN_3O_4$: 430.0258, found: 430.0235; HPLC (method 1): t_R = 19.4 min, purity 98.2%.

Methyl (*S*)-2-[(4-iodobenzyl)oxy]-3-(3-phenyl-1*H*-pyrazole-5-carboxamido)propanoate ((*S*)-38m)

Under N_2 atmosphere, a 1.0 M solution of trimethylphosphane in toluene (2.3 mL, 2.3 mmol) was added to an ice-cooled mixture of 3-phenyl-1*H*-pyrazole-5-carboxylic acid (220 mg, 1.2 mmol), (*S*)-**28** (340 mg, 0.94 mmol), and 2,2'-dithiodipyridine (110 mg, 0.50 mmol). After stirring the reaction mixture for 20 min at 0 °C, the ice-bath was removed and the mixture was stirred for 72 h at ambient temperature. Then, water (1.5 mL) was added and the mixture was stirred for 20 min. Afterwards, the mixture was diluted with dichloromethane and washed with a saturated aqueous solution of $NaHCO_3$, an ice-cold solution of 1.0 M HCl, and water. The combined organic layers were dried (Na_2SO_4), filtered, and the solvent was removed *in vacuo*. The residue was purified by flash column chromatography (Ø = 4 cm, h = 23 cm, V = 30 mL, petroleum ether/ethyl acetate = 1/1, R_f = 0.27) to give (*S*)-**38m** as colorless solid (313 mg, 0.62 mmol, 66%). m.p.: 159 °C; $[\alpha]_D^{20}$ = -26.2 (1.3, methanol); HPLC (method 1): t_R = 22.7 min, purity 99.0%.

Methyl (*R*)-2-[(4-iodobenzyl)oxy]-3-(3-phenyl-1*H*-pyrazole-5-carboxamido)propanoate ((*R*)-38m)

Under N_2 atmosphere, a 1.0 M solution of trimethylphosphane in toluene (2.3 mL, 2.3 mmol) was added to an ice-cooled mixture of 3-phenyl-1*H*-pyrazole-5-carboxylic acid (190 mg, 1.0 mmol), (*R*)-**28** (330 mg, 0.92 mmol), and 2,2'-dithiodipyridine (100 mg, 0.46 mmol). After stirring the reaction mixture for 20 min at 0 °C, the ice-bath was removed and the mixture was stirred for 48 h at ambient temperature. Then, water (1.0 mL) was added and the mixture was stirred for 20 min. Afterwards, the mixture was diluted with dichloromethane and washed with a saturated aqueous solution of

$NaHCO_3$, an ice-cold solution of 1.0 M HCl, and water. The combined organic layers were dried (Na_2SO_4), filtered, and the solvent was removed *in vacuo*. The residue was purified by flash column chromatography (Ø = 4 cm, h = 16 cm, V = 30 mL, petroleum ether/ethyl acetate = 1/1, R_f = 0.27) to give (*R*)-**38m** as colorless solid (290 mg, 0.58 mmol, 62%). m.p.: 159 °C; $[\alpha]_D^{20}$ = +27.5 (1.2, methanol); HPLC (method 1): t_R = 22.7 min, purity 99.6%.

Spectroscopic data of (*S*)-**38m** *and* (*R*)-**38m**:

^{1}H NMR (DMSO-d_6): δ [ppm] = 3.47 – 3.74 (m, 5H, OCHCH_2NH, CO_2CH_3), 4.15 – 4.28 (m, 1H, OC*H*CH_2NH), 4.44 (d, *J* = 12.2 Hz, 1H, OCH_2Ar), 4.56 – 4.64 (m, 1H, OCH_2Ar), 7.06 (d, *J* = 2.0 Hz, 0.7H, 4''-$H_{pyrazole}$), 7.11 – 7.21 (m, 2H, 2'-$H_{4\text{-iodophenyl}}$, 6'-$H_{4\text{-iodophenyl}}$), 7.25 (d, *J* = 1.6 Hz, 0.3H, 4''-$H_{pyrazole}$), 7.28 – 7.53 (m, 3H, 3'''-H_{phenyl}, 4'''-H_{phenyl}, 5'''-H_{phenyl}), 7.59 – 7.69 (m, 2H, 3'-$H_{4\text{-iodophenyl}}$, 5'-$H_{4\text{-iodophenyl}}$), 7.74 – 7.85 (m, 2H, 2'''-H_{phenyl}, 6'''-H_{phenyl}), 8.17 (t, *J* = 6.0 Hz, 0.7H, CON*H*), 8.68 (t, *J* = 5.8 Hz, 0.3H, CON*H*), 13.59 – 13.64 (m, 0.3H, 1''-$H_{pyrazole}$), 13.64 – 13.69 (m, 0.7H, 1''-$H_{pyrazole}$), two annular tautomers exist in the ratio 70:30; ^{13}C NMR (DMSO-d_6): δ [ppm] = 40.5 (1C, OCH*C*H_2NH), 51.9 (1C, CO_2CH_3), 70.6 (1C, O*C*H_2Ar), 76.6 (1C, O*C*HCH_2NH), 93.5 (1C, C-4'$_{4\text{-iodophenyl}}$), 102.6 (1C, C-4''$_{pyrazole}$), 125.3 (2C, C-2'''$_{phenyl}$, C-6'''$_{phenyl}$), 128.5 (1C, C-4'''$_{phenyl}$), 128.7 (1C, C-1'''$_{phenyl}$), 129.0 (2C, C-3'''$_{phenyl}$, C-5'''$_{phenyl}$), 129.8 (2C, C-2'$_{4\text{-iodophenyl}}$, C-6'$_{4\text{-iodophenyl}}$), 136.9 (2C, C-3'$_{4\text{-iodophenyl}}$, C-5'$_{4\text{-iodophenyl}}$), 137.6 (1C, C-1'$_{4\text{-iodophenyl}}$), 143.5 (1C, C-3''$_{pyrazole}$), 147.5 (1C, C-5''$_{pyrazole}$), 161.8 (1C, *C*ONH), 170.9 (1C, *C*O_2CH_3), two annular tautomers exist in the ratio 70:30, the signals of the major tautomer are given; IR (neat): $\tilde{\nu}$ [cm^{-1}] = 3433, 3192, 1742, 1649, 1546, 1462, 1204, 1129, 1006, 801, 761, 687, 556, 479; HRMS (*m*/*z*): $[M+H]^+$ calcd for $C_{21}H_{21}IN_3O_4$: 506.0571, found: 506.0540.

Methyl (*S*)-3-(3,5-dimethylisoxazole-4-carboxamido)-2-[(4-iodobenzyl)oxy]propanoate ((*S*)-38n)

Under N_2 atmosphere, a 1.0 M solution of trimethylphosphane in toluene (2.0 mL, 2.0 mmol) was added to an ice-cooled mixture of 3,5-dimethylisoxazole-4-carboxylic acid (130 mg, 0.90 mmol), (*S*)-**28** (290 mg, 0.81 mmol), and 2,2'-dithiodipyridine (72 mg, 0.33 mmol). After stirring the reaction mixture for 20 min at 0 °C, the ice-bath was removed and the mixture was stirred for 72 h at ambient temperature. Then, water (2.0 mL) was added and the mixture was stirred for 20 min. Afterwards, the mixture was diluted with dichloromethane and washed with a saturated aqueous solution of $NaHCO_3$, an ice-cold solution of 1.0 M HCl, and water. The combined organic layers were dried (Na_2SO_4), filtered, and the solvent was removed *in vacuo*. The residue was purified by automatic flash column chromatography using an Interchim puriFlash® XS 420 system (petroleum ether/ethyl acetate = 1/1, Biotage® SNAP Ultra HP-Sphere™ 25 g) to give (*S*)-**38n** as colorless solid (310 mg, 0.68 mmol, 83%). m.p.: 119 °C; R_f = 0.36 (petroleum ether/ethyl acetate = 1/1); $[\alpha]_D^{20}$ = -19.9 (1.5, methanol); HPLC (method 1): t_R = 21.7 min, purity 98.4%.

Methyl (*R*)-3-(3,5-dimethylisoxazole-4-carboxamido)-2-[(4-iodobenzyl)oxy]propanoate ((*R*)-38n)

Under N_2 atmosphere, a 1.0 M solution of trimethylphosphane in toluene (2.0 mL, 2.0 mmol) was added to an ice-cooled mixture of 3,5-dimethylisoxazole-4-carboxylic acid (130 mg, 0.91 mmol), (*R*)-**28** (290 mg, 0.80 mmol), and 2,2'-dithiodipyridine (71 mg, 0.32 mmol). After stirring the reaction mixture for 20 min at 0 °C, the ice-bath was removed and the mixture was stirred for 72 h at ambient temperature. Then, water (2.0 mL) was added and the mixture was stirred for 20 min. Afterwards, the mixture was diluted with dichloromethane and washed with a saturated aqueous solution of $NaHCO_3$, an ice-cold solution of 1.0 M HCl, and water. The combined organic layers were dried (Na_2SO_4), filtered, and the solvent was removed *in vacuo*.

The residue was purified by automatic flash column chromatography using an Interchim puriFlash® XS 420 system (petroleum ether/ethyl acetate = 3/2 → 1/1, Biotage® SNAP Ultra HP-Sphere™ 25 g) to give (*R*)-**38n** as colorless solid (310 mg, 0.68 mmol, 84%). m.p.: 120 °C; R_f = 0.36 (petroleum ether/ethyl acetate = 1/1); $[\alpha]_D^{20}$ = +20.0 (1.2, methanol); HPLC (method 1): t_R = 21.8 min, purity 99.8%.

Spectroscopic data of (*S*)-**38n** *and* (*R*)-**38n**:

^{1}H NMR (DMSO-d_6): δ [ppm] = 2.23 (s, 3H, H_3CC-3''$_{isoxazole}$), 2.44 (s, 3H, H_3CC-5''$_{isoxazole}$), 3.48 – 3.62 (m, 2H, OCHCH_2NH), 3.67 (s, 3H, CO_2CH_3), 4.16 – 4.21 (m, 1H, OC*H*CH$_2$NH), 4.43 (d, *J* = 12.0 Hz, 1H, OCH_2Ar), 4.57 (d, *J* = 12.0 Hz, 1H, OCH_2Ar), 7.14 – 7.21 (m, 2H, 2'-H$_{4\text{-iodophenyl}}$, 6'-H$_{4\text{-iodophenyl}}$), 7.66 – 7.73 (m, 2H, 3'-H$_{4\text{-iodophenyl}}$, 5'-H$_{4\text{-iodophenyl}}$), 8.17 (t, *J* = 6.0 Hz, 1H, CON*H*); ^{13}C NMR (DMSO-d_6): δ [ppm] = 10.4 (1C, H$_3$*C*C-3''$_{isoxazole}$), 11.9 (1C, H$_3$*C*C-5''$_{isoxazole}$), 40.7 (1C, OCH*C*H$_2$NH), 51.9 (1C, CO$_2$*C*H$_3$), 70.6 (1C, O*C*H$_2$Ar), 76.5 (1C, O*C*HCH$_2$NH), 93.7 (1C, C-4'$_{4\text{-iodophenyl}}$), 112.7 (1C, C-4''$_{isoxazole}$), 130.0 (2C, C-2'$_{4\text{-iodophenyl}}$, C-6'$_{4\text{-iodophenyl}}$), 137.0 (2C, C-3'$_{4\text{-iodophenyl}}$, C-5'$_{4\text{-iodophenyl}}$), 137.5 (1C, C-1'$_{4\text{-iodophenyl}}$), 158.2 (1C, C-3''$_{isoxazole}$), 161.6 (1C, *C*ONH), 169.5 (1C, C-5''$_{isoxazole}$), 170.9 (1C, *C*O$_2$CH$_3$); IR (neat): $\tilde{\nu}$ [cm^{-1}] = 3332, 2958, 1738, 1641, 1618, 1608, 1537, 1417, 1297, 1207, 1163, 1126, 1044, 1008, 802, 685, 639, 430; HRMS (*m*/*z*): $[M+H]^+$ calcd for $C_{17}H_{20}IN_2O_5$: 459.0411, found: 459.0413.

Methyl (*S*)-3-([1,1'-biphenyl]-4-carboxamido)-2-[(4-{[4-(morpholinomethyl)phenyl]ethynyl}benzyl)oxy]propanoate ((*S*)-39b)

Under N_2 atmosphere, copper(I) iodide (10 mg, 0.053 mmol), bis(triphenylphosphine)palladium(II) chloride (37 mg, 0.053 mmol), and diisopropylamine (3 mL) were added to a solution of (*S*)-**38b** (260 mg, 0.50 mmol) in dry THF (12 mL) at ambient temperature and the mixture was stirred for 20 min. Then,

4-(4-ethynylbenzyl)morpholine (300 mg, 1.5 mmol) was added in two portions at an interval of 30 min. After stirring the reaction mixture for 48 h at ambient temperature, the solvent was removed *in vacuo*. The residue was dissolved in a mixture of dichloromethane and methanol (20/1) and filtered through a short silica gel column. The solvent was removed *in vacuo* and the residue was purified by flash column chromatography (Ø = 4 cm, h = 27 cm, V = 30 mL, dichloromethane/methanol = 20/1, R_f = 0.44) to give (*S*)-**39b** as colorless solid (150 mg, 0.25 mmol, 50%). m.p.: 188 °C; $[\alpha]_D^{20}$ = -30.4 (1.2, dimethyl sulfoxide); HPLC (method 1): t_R = 21.4 min, purity 97.9%.

Methyl (*R*)-3-([1,1'-biphenyl]-4-carboxamido)-2-[(4-^{3}benzyl)oxy]propanoate ((*R*)-39b)

Under N_2 atmosphere, copper(I) iodide (8 mg, 0.042 mmol), tetrakis(triphenylphosphine)palladium(0) (47 mg, 0.041 mmol), and diisopropylamine (2 mL) were added to a solution of (*R*)-**38b** (190 mg, 0.36 mmol) in dry THF (6 mL) at ambient temperature and the mixture was stirred for 20 min. Then, 4-(4-ethynylbenzyl)morpholine (250 mg, 1.2 mmol) was added. After heating the reaction mixture to reflux overnight, the solvent was removed *in vacuo*. The residue was dissolved in a mixture of dichloromethane and methanol (10/1) and filtered through a short silica gel column. The solvent was removed *in vacuo* and the residue was purified by flash column chromatography (Ø = 4 cm, h = 12 cm, V = 30 mL, dichloromethane/methanol = 20/1, R_f = 0.44) to give (*R*)-**39b** as colorless solid (160 mg, 0.26 mmol, 74%). m.p.: 188 °C; $[\alpha]_D^{20}$ = +27.0 (1.0, dimethyl sulfoxide); HPLC (method 1): t_R = 21.2 min, purity 97.3%.

*Spectroscopic data of (S)-***39b** *and (R)-***39b***:*

^{1}H NMR (DMSO-d_6): δ [ppm] = 2.30 – 2.40 (m, 4H, N(CH_2CH$_2$)$_2$O), 3.49 (s, 2H, NCH_2Ar), 3.49 – 3.61 (m, 5H, N(CH$_2$CH_2)$_2$O, OCHCH_2NH (1H)), 3.65 – 3.73 (m, 1H, OCHCH_2NH), 3.70 (s, 3H, CO$_2$CH_3), 4.25 (dd, *J* = 7.4/4.9 Hz, 1H, OC*H*CH$_2$NH), 4.50 (d, *J* = 12.5 Hz, 1H, OCH_2Ar), 4.68 (d, *J* = 12.5 Hz, 1H, OCH_2Ar), 7.32 – 7.52 (m, 11H, 3'-$H_{[1,1'\text{-biphenyl}]\text{-4-yl}}$, 4'-$H_{[1,1'\text{-biphenyl}]\text{-4-yl}}$, 5'-$H_{[1,1'\text{-biphenyl}]\text{-4-yl}}$, 2''-$H_{4\text{-\{[4-(morpholinomethyl)phenyl]ethynyl\}phenyl}}$, 3''-$H_{4\text{-\{[4-(morpholinomethyl)phenyl]ethynyl\}phenyl}}$, 5''-$H_{4\text{-\{[4-(morpholinomethyl)phenyl]ethynyl\}phenyl}}$, 6''-$H_{4\text{-\{[4-(morpholinomethyl)phenyl]ethynyl\}phenyl}}$, 2'''-$H_{4\text{-(morpholinomethyl)phenyl}}$, 3'''-$H_{4\text{-(morpholinomethyl)phenyl}}$, 5'''-$H_{4\text{-(morpholinomethyl)phenyl}}$, 6'''-$H_{4\text{-(morpholinomethyl)phenyl}}$), 7.70 – 7.75 (m, 2H, 2'-$H_{[1,1'\text{-biphenyl}]\text{-4-yl}}$, 6'-$H_{[1,1'\text{-biphenyl}]\text{-4-yl}}$), 7.75 – 7.81 (m, 2H, 2-$H_{[1,1'\text{-biphenyl}]\text{-4-yl}}$, 6-$H_{[1,1'\text{-biphenyl}]\text{-4-yl}}$), 7.88 – 7.93 (m, 2H, 3-$H_{[1,1'\text{-biphenyl}]\text{-4-yl}}$, 5-$H_{[1,1'\text{-biphenyl}]\text{-4-yl}}$), 8.74 (t, *J* = 5.9 Hz, 1H, CON*H*); ^{13}C NMR (DMSO-d_6): δ [ppm] = 41.4 (1C, OCH*C*H$_2$NH), 51.9 (1C, CO$_2$*C*H$_3$), 53.1 (2C, N(*C*H$_2$CH$_2$)$_2$O), 62.0 (1C, Ar*C*H$_2$N), 66.2 (2C, N(CH$_2$*C*H$_2$)$_2$O), 71.0 (1C, O*C*H$_2$Ar), 76.6 (1C, O*C*HCH$_2$NH), 89.0 (1C, *C*≡C), 89.3 (1C, *C*≡C), 120.8 (1C, C-1'''$_{4\text{-(morpholinomethyl)phenyl}}$), 121.5 (1C, C-4''$_{4\text{-\{[4-(morpholinomethyl)phenyl]ethynyl\}phenyl}}$), 126.5 (2C, C-2$_{[1,1'\text{-biphenyl}]\text{-4-yl}}$, C-6$_{[1,1'\text{-biphenyl}]\text{-4-yl}}$), 126.9 (2C, C-2'$_{[1,1'\text{-biphenyl}]\text{-4-yl}}$, C-6'$_{[1,1'\text{-biphenyl}]\text{-4-yl}}$), 127.9 (4C, C-2''$_{4\text{-\{[4-(morpholinomethyl)phenyl]ethynyl\}phenyl}}$, C-6''$_{4\text{-\{[4-(morpholinomethyl)phenyl]ethynyl\}phenyl}}$, C-3$_{[1,1'\text{-biphenyl}]\text{-4-yl}}$, C-5$_{[1,1'\text{-biphenyl}]\text{-4-yl}}$), 128.0 (1C, C-4'$_{[1,1'\text{-biphenyl}]\text{-4-yl}}$), 129.0 (2C, C$_{\text{arom.}}$), 129.1 (2C, C$_{\text{arom.}}$), 131.17 (2C, C$_{\text{arom.}}$), 131.21 (2C, C$_{\text{arom.}}$), 133.0 (1C, C-4$_{[1,1'\text{-biphenyl}]\text{-4-yl}}$), 138.4 (1C, C-1''$_{4\text{-\{[4-(morpholinomethyl)phenyl]ethynyl\}phenyl}}$), 138.8 (1C, C-4'''$_{4\text{-(morpholinomethyl)phenyl}}$), 139.2 (1C, C-1'$_{[1,1'\text{-biphenyl}]\text{-4-yl}}$), 142.8 (1C, C-1$_{[1,1'\text{-biphenyl}]\text{-4-yl}}$), 166.2 (1C, *C*ONH), 171.0 (1C, *C*O$_2$CH$_3$); IR (neat): $\tilde{\nu}$ [cm^{-1}] = 3284, 2957, 2816, 1740, 1626, 1535, 1318, 1207, 1113, 1098, 1005, 913, 849, 694, 507, 483; HRMS (*m*/*z*): [M+H]$^+$ calcd for $C_{37}H_{37}N_2O_5$: 589.2697, found: 589.2690.

Methyl (*S*)-3-([1,1'-biphenyl]-3-carboxamido)-2-[(4-{[4-(morpholinomethyl)phenyl]ethynyl}benzyl)oxy]propanoate ((*S*)-39c)

Under N_2 atmosphere, copper(I) iodide (14 mg, 0.071 mmol), bis(triphenylphosphine)palladium(II) chloride (35 mg, 0.050 mmol), and diisopropylamine (3 mL) were added to a solution of (*S*)-**38c** (220 mg, 0.42 mmol) in dry THF (10 mL) at ambient temperature and the mixture was stirred for 20 min. Then, 4-(4-ethynylbenzyl)morpholine (360 mg, 1.8 mmol) was added in two portions at an interval of 30 min. After stirring the reaction mixture for 48 h at ambient temperature, the solvent was removed *in vacuo*. The residue was dissolved in a mixture of petroleum ether and ethyl acetate (1/2) and filtered through a short silica gel column. The solvent was removed *in vacuo* and the residue was purified by flash column chromatography (Ø = 3 cm, h = 27 cm, V = 18 mL, dichloromethane/methanol = 20/1, R_f = 0.44) to give (*S*)-**39c** as yellow solid (220 mg, 0.37 mmol, 88%). m.p.: 56 °C; $[\alpha]_D^{20}$ = -52.3 (0.7, dimethyl sulfoxide); HPLC (method 1): t_R = 21.7 min, purity 98.7%.

Methyl (*R*)-3-([1,1'-biphenyl]-3-carboxamido)-2-[(4-{[4-(morpholinomethyl)phenyl]ethynyl}benzyl)oxy]propanoate ((*R*)-39c)

Under N_2 atmosphere, copper(I) iodide (10 mg, 0.054 mmol), bis(triphenylphosphine)palladium(II) chloride (28 mg, 0.040 mmol), and diisopropylamine (3 mL) were added to a solution of (*R*)-**38c** (150 mg, 0.30 mmol) in dry THF (10 mL) at ambient temperature and the mixture was stirred for 20 min. Then, 4-(4-ethynylbenzyl)morpholine (200 mg, 0.98 mmol) was added in two portions at an interval of 30 min. After stirring the reaction mixture for 48 h at ambient temperature, the solvent was removed *in vacuo*. The residue was dissolved in a mixture of dichloromethane and methanol (20/1) and filtered through a short silica gel column.

The solvent was removed *in vacuo* and the residue was purified by flash column chromatography (Ø = 3 cm, h = 25 cm, V = 18 mL, dichloromethane/methanol = 20/1, R_f = 0.44) to give (*R*)-**39c** as yellow solid (150 mg, 0.26 mmol, 87%). m.p.: 112 °C; $[\alpha]_D^{20}$ = +32.6 (1.9, dimethyl sulfoxide); HPLC (method 1): t_R = 21.3 min, purity 98.2%.

Spectroscopic data of (*S*)-**39c** *and* (*R*)-**39c**:

^{1}H NMR (DMSO-d_6): δ [ppm] = 2.28 – 2.41 (m, 4H, N(CH_2CH$_2$)$_2$O), 3.49 (s, 2H, NCH_2Ar), 3.51 – 3.62 (m, 5H, N(CH$_2$CH_2)$_2$O, OCHCH_2NH (1H)), 3.65 – 3.76 (m, 1H, OCHCH_2NH), 3.70 (s, 3H, CO$_2$CH_3), 4.25 (dd, *J* = 7.4/4.9 Hz, 1H, OC*H*CH$_2$NH), 4.50 (d, *J* = 12.5 Hz, 1H, OCH_2Ar), 4.68 (d, *J* = 12.5 Hz, 1H, OCH_2Ar), 7.32 – 7.44 (m, 7H, 4'-H$_{[1,1'\text{-biphenyl}]\text{-3-yl}}$, 2''-H$_{4\text{-}\{[4\text{-(morpholinomethyl)phenyl}]\text{ethynyl}\}\text{phenyl}}$, 3''-H$_{4\text{-}\{[4\text{-(morpholinomethyl)phenyl}]\text{ethynyl}\}\text{phenyl}}$, 5''-H$_{4\text{-}\{[4\text{-(morpholinomethyl)phenyl}]\text{ethynyl}\}\text{phenyl}}$, 6''-H$_{4\text{-}\{[4\text{-(morpholinomethyl)phenyl}]\text{ethynyl}\}\text{phenyl}}$, 3'''-H$_{4\text{-(morpholinomethyl)phenyl}}$, 5'''-H$_{4\text{-(morpholinomethyl)phenyl}}$), 7.45 – 7.55 (m, 4H, 3'-H$_{[1,1'\text{-biphenyl}]\text{-3-yl}}$, 5'-H$_{[1,1'\text{-biphenyl}]\text{-3-yl}}$, 2'''-H$_{4\text{-(morpholinomethyl)phenyl}}$, 6'''-H$_{4\text{-(morpholinomethyl)phenyl}}$), 7.55 – 7.60 (m, 1H, 5-H$_{[1,1'\text{-biphenyl}]\text{-3-yl}}$), 7.70 – 7.77 (m, 2H, 2'-H$_{[1,1'\text{-biphenyl}]\text{-3-yl}}$, 6'-H$_{[1,1'\text{-biphenyl}]\text{-3-yl}}$), 7.79 – 7.87 (m, 2H, 4-H$_{[1,1'\text{-biphenyl}]\text{-3-yl}}$, 6-H$_{[1,1'\text{-biphenyl}]\text{-3-yl}}$), 8.09 – 8.13 (m, 1H, 2-H$_{[1,1'\text{-biphenyl}]\text{-3-yl}}$), 8.86 (t, *J* = 5.9 Hz, 1H, CON*H*); ^{13}C NMR (DMSO-d_6): δ [ppm] = 41.5 (1C, OCH*C*H$_2$NH), 52.0 (1C, CO$_2$*C*H$_3$), 53.2 (2C, N(*C*H$_2$CH$_2$)$_2$O), 62.0 (1C, Ar*C*H$_2$N), 66.2 (2C, N(CH$_2$*C*H$_2$)$_2$O), 71.0 (1C, O*C*H$_2$Ar), 76.6 (1C, O*C*HCH$_2$NH), 89.0 (1C, *C*≡C), 89.3 (1C, *C*≡C), 120.8 (1C, C-1'''$_{4\text{-(morpholinomethyl)phenyl}}$), 121.5 (1C, C-4''$_{4\text{-}\{[4\text{-(morpholinomethyl)phenyl}]\text{ethynyl}\}\text{phenyl}}$), 125.4 (1C, C-2$_{[1,1'\text{-biphenyl}]\text{-3-yl}}$), 126.5 (1C, C-4$_{[1,1'\text{-biphenyl}]\text{-3-yl}}$), 126.8 (2C, C-2'$_{[1,1'\text{-biphenyl}]\text{-3-yl}}$, C-6'$_{[1,1'\text{-biphenyl}]\text{-3-yl}}$), 127.78 (1C, C-4'$_{[1,1'\text{-biphenyl}]\text{-3-yl}}$), 127.84 (2C, C-2''$_{4\text{-}\{[4\text{-(morpholinomethyl)phenyl}]\text{ethynyl}\}\text{phenyl}}$, C-6''$_{4\text{-}\{[4\text{-(morpholinomethyl)phenyl}]\text{ethynyl}\}\text{phenyl}}$), 128.98 (2C, C-3'$_{[1,1'\text{-biphenyl}]\text{-3-yl}}$, C-5'$_{[1,1'\text{-biphenyl}]\text{-3-yl}}$), 129.04 (1C, C-5$_{[1,1'\text{-biphenyl}]\text{-3-yl}}$), 129.2 (2C, C-3'''$_{4\text{-(morpholinomethyl)phenyl}}$, C-5'''$_{4\text{-}}$

(morpholinomethyl)phenyl), 129.5 (1C, C-6[1,1'-biphenyl]-3-yl), 131.15 (2C, Carom.), 131.22 (2C, Carom.), 134.8 (1C, C-3[1,1'-biphenyl]-3-yl), 138.5 (1C, C-1''4-{[4-(morpholinomethyl)phenyl]ethynyl}phenyl), 138.9 (1C, C-4'''4-(morpholinomethyl)phenyl), 139.5 (1C, C-1'[1,1'-biphenyl]-3-yl), 140.2 (1C, C-1[1,1'-biphenyl]-3-yl), 166.4 (1C, *C*ONH), 171.0 (1C, *C*O_2CH_3); IR (neat): $\tilde{\nu}$ [cm^{-1}] = 3325, 2950, 2854, 2807, 1745, 1646, 1517, 1308, 1259, 1205, 1113, 1006, 913, 864, 818, 741, 697, 539; HRMS (*m*/*z*): $[M+H]^+$ calcd for $C_{37}H_{37}N_2O_5$: 589.2697, found: 589.2679.

Methyl (*S*)-2-[(4-{[4-(morpholinomethyl)phenyl]ethynyl}benzyl)oxy]-3-(4-phenoxybenzamido)propanoate ((*S*)-39d)

Under N_2 atmosphere, copper(I) iodide (15 mg, 0.079 mmol), bis(triphenylphosphine)palladium(II) chloride (52 mg, 0.074 mmol), and diisopropylamine (5 mL) were added to a solution of (*S*)-**38d** (280 mg, 0.53 mmol) in dry THF (12 mL) at ambient temperature and the mixture was stirred for 20 min. Then, 4-(4-ethynylbenzyl)morpholine (250 mg, 1.2 mmol) was added in two portions at an interval of 30 min. After stirring the reaction mixture for 72 h at ambient temperature, the solvent was removed *in vacuo*. The residue was dissolved in a mixture of petroleum ether and ethyl acetate (1/4) and filtered through a short silica gel column. The solvent was removed *in vacuo* and the residue was purified by flash column chromatography (Ø = 4 cm, h = 14 cm, V = 30 mL, petroleum ether/ethyl acetate = 1/4, R_f = 0.38) to give (*S*)-**39d** as colorless solid (290 mg, 0.47 mmol, 90%). m.p.: 127 °C; $[\alpha]_D^{20}$ = -8.1 (1.4, methanol); HPLC (method 1): t_R = 21.4 min, purity 99.5%.

Methyl (*R*)-2-[(4-{[4-(morpholinomethyl)phenyl]ethynyl}benzyl)oxy]-3-(4-phenoxybenzamido)propanoate ((*R*)-39d)

Under N_2 atmosphere, copper(I) iodide (19 mg, 0.10 mmol), bis(triphenylphosphine)palladium(II) chloride (59 mg, 0.084 mmol), and diisopropylamine (5 mL) were added to a solution of (*R*)-**38d** (290 mg, 0.55 mmol) in dry THF (12 mL) at ambient temperature and the mixture was stirred for 20 min. Then, 4-(4-ethynylbenzyl)morpholine (260 mg, 1.3 mmol) was added in two portions at an interval of 30 min. After stirring the reaction mixture for 24 h at ambient temperature, the solvent was removed *in vacuo*. The residue was dissolved in a mixture of dichloromethane and methanol (50/1) and filtered through a short silica gel column. The solvent was removed *in vacuo* and the residue was purified by flash column chromatography (Ø = 3 cm, h = 23 cm, V = 20 mL, dichloromethane/methanol = 50/1 → 20/1) to give (*R*)-**39d** as colorless solid (310 mg, 0.51 mmol, 93%). m.p.: 127 °C; R_f = 0.18 (dichloromethane/methanol = 50/1); $[\alpha]_D^{20}$ = +9.2 (1.3, methanol); HPLC (method 1): t_R = 21.4 min, purity 98.6%.

Spectroscopic data of (*S*)-**39d** *and* (*R*)-**39d**:

^{1}H NMR (DMSO-d_6): δ [ppm] = 2.29 – 2.40 (m, 4H, N(CH_2CH$_2$)$_2$O), 3.46 – 3.55 (m, 3H, NCH_2Ar, OCHCH_2NH (1H)), 3.55 – 3.61 (m, 4H, N(CH$_2$CH_2)$_2$O), 3.61 – 3.71 (m, 4H, OCHCH_2NH (1H), CO$_2$CH_3), 4.22 (dd, *J* = 7.3/5.0 Hz, 1H, OC*H*CH$_2$NH), 4.49 (d, *J* = 12.5 Hz, 1H, OCH_2Ar), 4.66 (d, *J* = 12.5 Hz, 1H, OCH_2Ar), 7.01 – 7.06 (m, 2H, 3'''-H$_{benzoyl}$, 5'''-H$_{benzoyl}$), 7.06 – 7.11 (m, 2H, 2''''-H$_{phenoxy}$, 6''''-H$_{phenoxy}$), 7.16 – 7.22 (m, 1H, 4''''-H$_{phenoxy}$), 7.33 – 7.46 (m, 8H, 2'-H$_{4\text{-}\{[4\text{-(morpholinomethyl)phenyl]ethynyl}\}phenyl}$, 3'-H$_{4\text{-}\{[4\text{-(morpholinomethyl)phenyl]ethynyl}\}phenyl}$, 5'-H$_{4\text{-}\{[4\text{-(morpholinomethyl)phenyl]ethynyl}\}phenyl}$, 6'-H$_{4\text{-}\{[4\text{-(morpholinomethyl)phenyl]ethynyl}\}phenyl}$, 3''-H$_{4\text{-(morpholinomethyl)phenyl}}$, 5''-H$_{4\text{-(morpholinomethyl)phenyl}}$, 3''''-

H$_{phenoxy}$, 5''''-H$_{phenoxy}$), 7.47 – 7.52 (m, 2H, 2''-H$_{4-(morpholinomethyl)phenyl}$, 6''-H$_{4-(morpholinomethyl)phenyl}$), 7.82 – 7.88 (m, 2H, 2'''-H$_{benzoyl}$, 6'''-H$_{benzoyl}$), 8.62 (t, *J* = 5.9 Hz, 1H, CON*H*); ^{13}C NMR (DMSO-*d*$_6$): δ [ppm] = 41.4 (1C, OCH*C*H$_2$NH), 51.9 (1C, CO$_2$*C*H$_3$), 53.1 (2C, N(*C*H$_2$CH$_2$)$_2$O), 62.0 (1C, Ar*C*H$_2$N), 66.2 (2C, N(CH$_2$*C*H$_2$)$_2$O), 70.9 (1C, O*C*H$_2$Ar), 76.6 (1C, O*C*HCH$_2$NH), 89.0 (1C, *C*≡C), 89.3 (1C, *C*≡C), 117.4 (2C, C-3'''$_{benzoyl}$, C-5'''$_{benzoyl}$), 119.4 (2C, C-2''''$_{phenoxy}$, C-6''''$_{phenoxy}$), 120.8 (1C, C-1''$_{4-(morpholinomethyl)phenyl}$), 121.5 (1C, C-4'$_{4-\{[4-(morpholinomethyl)phenyl]ethynyl\}phenyl}$), 124.2 (1C, C-4''''$_{phenoxy}$), 127.9 (2C, C-2'$_{4-\{[4-(morpholinomethyl)phenyl]ethynyl\}phenyl}$, C-6'$_{4-\{[4-(morpholinomethyl)phenyl]ethynyl\}phenyl}$), 128.9 (1C, C-1'''$_{benzoyl}$), 129.1 (2C, C-3''$_{4-(morpholinomethyl)phenyl}$, C-5''$_{4-(morpholinomethyl)phenyl}$), 129.4 (2C, C-2'''$_{benzoyl}$, C-6'''$_{benzoyl}$), 130.2 (2C, C-3''''$_{phenoxy}$, C-5''''$_{phenoxy}$), 131.1 (2C, C$_{arom.}$), 131.2 (2C, C$_{arom.}$), 138.4 (1C, C-1'$_{4-\{[4-(morpholinomethyl)phenyl]ethynyl\}phenyl}$), 138.8 (1C, C-4''$_{4-(morpholinomethyl)phenyl}$), 155.6 (1C, C-1''''$_{phenoxy}$), 159.5 (1C, C-4'''$_{benzoyl}$), 165.8 (1C, *C*ONH), 171.0 (1C, *C*O$_2$CH$_3$); IR (neat): $\tilde{\nu}$ [cm^{-1}] = 3306, 2956, 2811, 1742, 1630, 1587, 1539, 1487, 1247, 1212, 1109, 1038, 1006, 844, 689, 539, 476; HRMS (*m*/*z*): [M+H]$^+$ calcd for $C_{37}H_{37}N_2O_6$: 605.2646, found: 605.2681.

Methyl (*S*)-2-[(4-{[4-(morpholinomethyl)phenyl]ethynyl}benzyl)oxy]-3-(quinoline-2-carboxamido)propanoate ((*S*)-39e)

Under N_2 atmosphere, copper(I) iodide (18 mg, 0.095 mmol), bis(triphenylphosphine)palladium(II) chloride (58 mg, 0.083 mmol), and diisopropylamine (3 mL) were added to a solution of (*S*)-**38e** (310 mg, 0.64 mmol) in dry THF (10 mL) at ambient temperature and the mixture was stirred for 20 min. Then, 4-(4-ethynylbenzyl)morpholine (460 mg, 2.3 mmol) was added in two portions at an interval of 30 min. After stirring the reaction mixture for 16 h at ambient temperature,

the solvent was removed *in vacuo*. The residue was dissolved in a mixture of dichloromethane and isopropanol (20/1) and filtered through a short silica gel column. The solvent was removed *in vacuo* and the residue was purified by flash column chromatography (Ø = 4 cm, h = 28 cm, V = 30 mL, dichloromethane/isopropanol = 40/1, R_f = 0.24) to give (*S*)-**39e** as yellow oil (360 mg, 0.63 mmol, 99%). $[\alpha]_D^{20}$ = +3.1 (4.9, methanol); HPLC (method 1): t_R = 20.8 min, purity 97.0%.

Methyl (*R*)-2-[(4-{[4-(morpholinomethyl)phenyl]ethynyl}benzyl)oxy]-3-(quinoline-2-carboxamido)propanoate ((*R*)-39e)

Under N_2 atmosphere, copper(I) iodide (19 mg, 0.10 mmol), bis(triphenylphosphine)palladium(II) chloride (68 mg, 0.097 mmol), and diisopropylamine (3 mL) were added to a solution of (*R*)-**38e** (340 mg, 0.69 mmol) in dry THF (10 mL) at ambient temperature and the mixture was stirred for 20 min. Then, 4-(4-ethynylbenzyl)morpholine (510 mg, 2.5 mmol) was added in two portions at an interval of 30 min. After stirring the reaction mixture for 16 h at ambient temperature, the solvent was removed *in vacuo*. The residue was dissolved in a mixture of dichloromethane and methanol (20/1) and filtered through a short silica gel column. The solvent was removed *in vacuo* and the residue was purified by flash column chromatography (Ø = 4 cm, h = 26 cm, V = 30 mL, dichloromethane/isopropanol = 50/1 → 20/1) to give (*R*)-**39e** as yellow oil (310 mg, 0.54 mmol, 79%). R_f = 0.24 (dichloromethane/isopropanol = 40/1); $[\alpha]_D^{20}$ = -7.3 (1.1, methanol); HPLC (method 1): t_R = 20.8 min, purity 99.8%.

Spectroscopic data of (*S*)-**39e** *and* (*R*)-**39e**:

^{1}H NMR (DMSO-d_6): δ [ppm] = 2.29 – 2.40 (m, 4H, N(CH_2CH$_2$)$_2$O), 3.48 (s, 2H, NCH_2Ar), 3.54 – 3.61 (m, 4H, N(CH$_2$CH_2)$_2$O), 3.64 – 3.74 (m, 1H, OCHCH_2NH), 3.70 (s, 3H, CO$_2$CH_3), 3.75 – 3.85 (m, 1H, OCHCH_2NH), 4.35 (dd, *J* = 7.1/4.9 Hz, 1H, OC*H*CH$_2$NH), 4.53 (d, *J* = 12.4 Hz, 1H, OCH_2Ar), 4.71 (d, *J* = 12.4 Hz, 1H, OCH_2Ar), 7.32 – 7.38 (m, 2H, 3''-H$_{4\text{-(morpholinomethyl)phenyl}}$, 5''-H$_{4\text{-(morpholinomethyl)phenyl}}$), 7.40 (s, 4H, 2'-H$_{4\text{-\{[4-(morpholinomethyl)phenyl]ethynyl\}phenyl}}$, 3'-H$_{4\text{-\{[4-(morpholinomethyl)phenyl]ethynyl\}phenyl}}$, 5'-H$_{4\text{-\{[4-(morpholinomethyl)phenyl]ethynyl\}phenyl}}$, 6'-H$_{4\text{-\{[4-(morpholinomethyl)phenyl]ethynyl\}phenyl}}$), 7.46 – 7.51 (m, 2H, 2''-H$_{4\text{-(morpholinomethyl)phenyl}}$, 6''-H$_{4\text{-(morpholinomethyl)phenyl}}$), 7.70 – 7.76 (m, 1H, 6'''-H$_{\text{quinoline}}$), 7.86 – 7.93 (m, 1H, 7'''-H$_{\text{quinoline}}$), 8.07 – 8.12 (m, 1H, 5'''-H$_{\text{quinoline}}$), 8.12 – 8.18 (m, 2H, 3'''-H$_{\text{quinoline}}$, 8'''-H$_{\text{quinoline}}$), 8.58 (d, *J* = 8.5 Hz, 1H, 4'''-H$_{\text{quinoline}}$), 8.99 (t, *J* = 6.2 Hz, 1H, CON*H*); ^{13}C NMR (DMSO-d_6): δ [ppm] = 41.0 (1C, OCH*C*H$_2$NH), 52.0 (1C, CO$_2$*C*H$_3$), 53.2 (2C, N(*C*H$_2$CH$_2$)$_2$O), 62.0 (1C, Ar*C*H$_2$N), 66.2 (2C, N(CH$_2$*C*H$_2$)$_2$O), 71.0 (1C, O*C*H$_2$Ar), 76.5 (1C, O*C*HCH$_2$NH), 88.9 (1C, *C*≡C), 89.3 (1C, *C*≡C), 118.6 (1C, C-3'''$_{\text{quinoline}}$), 120.8 (1C, C-1''$_{4\text{-(morpholinomethyl)phenyl}}$), 121.5 (1C, C-4'$_{4\text{-\{[4-(morpholinomethyl)phenyl]ethynyl\}phenyl}}$), 127.9 (2C, C-2'$_{4\text{-\{[4-(morpholinomethyl)phenyl]ethynyl\}phenyl}}$, C-6'$_{4\text{-\{[4-(morpholinomethyl)phenyl]ethynyl\}phenyl}}$), 128.1 (1C, C$_{\text{arom.}}$), 128.2 (1C, C$_{\text{arom.}}$), 128.9 (1C, C-4a'''$_{\text{quinoline}}$), 129.16 (2C, C-3''$_{4\text{-(morpholinomethyl)phenyl}}$, C-5''$_{4\text{-(morpholinomethyl)phenyl}}$), 129.18 (1C, C-8'''$_{\text{quinoline}}$), 130.6 (1C, C-7'''$_{\text{quinoline}}$), 131.15 (2C, C$_{\text{arom.}}$), 131.24 (2C, C$_{\text{arom.}}$), 138.0 (1C, C-4'''$_{\text{quinoline}}$), 138.4 (1C, C-1'$_{4\text{-\{[4-(morpholinomethyl)phenyl]ethynyl\}phenyl}}$), 138.9 (1C, C-4''$_{4\text{-(morpholinomethyl)phenyl}}$), 146.0 (1C, C-8a'''$_{\text{quinoline}}$), 149.7 (1C, C-2'''$_{\text{quinoline}}$), 164.1 (1C, *C*ONH), 170.9 (1C, *C*O$_2$CH$_3$); IR (neat): $\tilde{\nu}$ [cm^{-1}] = 3390, 2853, 2806, 1746, 1674, 1519, 1499, 1206, 1113, 1006, 844, 773, 624, 539; HRMS (*m*/*z*): [M+H]$^+$ calcd for $C_{34}H_{34}N_3O_5$: 564.2493, found: 564.2467.

Methyl (*S*)-2-[(4-{[4-(morpholinomethyl)phenyl]ethynyl}benzyl)oxy]-3-(quinoline-3-carboxamido)propanoate ((*S*)-39f)

Under N_2 atmosphere, copper(I) iodide (14 mg, 0.074 mmol), bis(triphenylphosphine)palladium(II) chloride (41 mg, 0.058 mmol), and diisopropylamine (3 mL) were added to a solution of (*S*)-**38f** (280 mg, 0.57 mmol) in dry THF (12 mL) at ambient temperature and the mixture was stirred for 20 min. Then, 4-(4-ethynylbenzyl)morpholine (330 mg, 1.6 mmol) was added in two portions at an interval of 30 min. After stirring the reaction mixture for 48 h at ambient temperature, the solvent was removed *in vacuo*. The residue was dissolved in a mixture of dichloromethane and methanol (20/1) and filtered through a short silica gel column. The solvent was removed *in vacuo* and the residue was purified by flash column chromatography (Ø = 3 cm, h = 25 cm, V = 20 mL, dichloromethane/methanol = 20/1, R_f = 0.34) to give (*S*)-**39f** as yellow solid (250 mg, 0.44 mmol, 78%). m.p.: 106 °C; $[\alpha]_D^{20}$ = -3.1 (2.0, methanol); HPLC (method 1): t_R = 17.4 min, purity 99.8%.

Methyl (*R*)-2-[(4-{[4-(morpholinomethyl)phenyl]ethynyl}benzyl)oxy]-3-(quinoline-3-carboxamido)propanoate ((*R*)-39f)

Under N_2 atmosphere, copper(I) iodide (17 mg, 0.089 mmol), bis(triphenylphosphine)palladium(II) chloride (41 mg, 0.058 mmol), and diisopropylamine (3 mL) were added to a solution of (*R*)-**38f** (290 mg, 0.59 mmol) in dry THF (20 mL) at ambient temperature and the mixture was stirred for 20 min. Then, 4-(4-ethynylbenzyl)morpholine (360 mg, 1.8 mmol) was added in two portions at an interval of 30 min. After stirring the reaction mixture for 24 h at ambient temperature, the solvent was removed *in vacuo*. The residue was dissolved in a mixture of dichloromethane and methanol (20/1) and filtered through a short silica gel column.

The solvent was removed *in vacuo* and the residue was purified by flash column chromatography (Ø = 3 cm, h = 25 cm, V = 20 mL, dichloromethane/methanol = 20/1, R_f = 0.34) to give (*R*)-**39f** as orange solid (290 mg, 0.52 mmol, 88%). m.p.: 108 °C; $[\alpha]_D^{20}$ = +3.0 (1.0, methanol); HPLC (method 1): t_R = 17.1 min, purity 97.0%.

Spectroscopic data of (*S*)-**39f** *and* (*R*)-**39f**:

^{1}H NMR (DMSO-d_6): δ [ppm] = 2.29 – 2.41 (m, 4H, N(CH_2CH$_2$)$_2$O), 3.48 (s, 2H, NCH_2Ar), 3.53 – 3.65 (m, 5H, OCHCH_2NH (1H), N(CH$_2$CH_2)$_2$O), 3.71 (s, 3H, CO$_2$CH_3), 3.72 – 3.78 (m, 1H, OCHCH_2NH), 4.29 (dd, *J* = 7.3/4.9 Hz, 1H, OC*H*CH$_2$NH), 4.53 (d, *J* = 12.5 Hz, 1H, OCH_2Ar), 4.69 (d, *J* = 12.5 Hz, 1H, OCH_2Ar), 7.33 – 7.37 (m, 2H, 3''-$H_{4\text{-(morpholinomethyl)phenyl}}$, 5''-$H_{4\text{-(morpholinomethyl)phenyl}}$), 7.38 – 7.45 (m, 4H, 2'-$H_{4\text{-\{[4-(morpholinomethyl)phenyl]ethynyl\}phenyl}}$, 3'-$H_{4\text{-\{[4-(morpholinomethyl)phenyl]ethynyl\}phenyl}}$, 5'-$H_{4\text{-\{[4-(morpholinomethyl)phenyl]ethynyl\}phenyl}}$, 6'-$H_{4\text{-\{[4-(morpholinomethyl)phenyl]ethynyl\}phenyl}}$), 7.45 – 7.50 (m, 2H, 2''-$H_{4\text{-(morpholinomethyl)phenyl}}$, 6''-$H_{4\text{-(morpholinomethyl)phenyl}}$), 7.66 – 7.71 (m, 1H, 6'''-$H_{\text{quinoline}}$), 7.84 – 7.89 (m, 1H, 7'''-$H_{\text{quinoline}}$), 8.07 – 8.13 (m, 2H, 5'''-$H_{\text{quinoline}}$, 8'''-$H_{\text{quinoline}}$), 8.78 (d, *J* = 2.0 Hz, 1H, 4'''-$H_{\text{quinoline}}$), 9.07 (t, *J* = 5.9 Hz, 1H, CON*H*), 9.27 (s, 1H, 2'''-$H_{\text{quinoline}}$); ^{13}C NMR (DMSO-d_6): δ [ppm] = 41.5 (1C, OCH*C*H$_2$NH), 52.0 (1C, CO$_2$*C*H$_3$), 53.1 (2C, N(*C*H$_2$CH$_2$)$_2$O), 62.0 (1C, Ar*C*H$_2$N), 66.2 (2C, N(CH$_2$*C*H$_2$)$_2$O), 71.0 (1C, O*C*H$_2$Ar), 76.5 (1C, O*C*HCH$_2$NH), 88.9 (1C, *C*≡C), 89.3 (1C, *C*≡C), 120.8 (1C, C-1''$_{4\text{-(morpholinomethyl)phenyl}}$), 121.5 (1C, C-4'$_{4\text{-\{[4-(morpholinomethyl)phenyl]ethynyl\}phenyl}}$), 126.5 (1C, C-4a'''$_{\text{quinoline}}$), 126.8 (1C, C-3'''$_{\text{quinoline}}$), 127.4 (1C, C-6'''$_{\text{quinoline}}$), 127.9 (2C, C-2'$_{4\text{-\{[4-(morpholinomethyl)phenyl]ethynyl\}phenyl}}$, C-6'$_{4\text{-\{[4-(morpholinomethyl)phenyl]ethynyl\}phenyl}}$), 128.8 (1C, C-8'''$_{\text{quinoline}}$), 129.1 (3C, C-3''$_{4\text{-(morpholinomethyl)phenyl}}$, C-5''$_{4\text{-(morpholinomethyl)phenyl}}$, C-5'''$_{\text{quinoline}}$), 131.1 (2C, C-3'$_{4\text{-\{[4-(morpholinomethyl)phenyl]ethynyl\}phenyl}}$, C-5'$_{4\text{-\{[4-(morpholinomethyl)phenyl]ethynyl\}phenyl}}$), 131.2 (3C, C-2''$_{4\text{-(morpholinomethyl)phenyl}}$, C-6''$_{4\text{-(morpholinomethyl)phenyl}}$,C-7'''$_{\text{quinoline}}$), 135.6 (1C, C-

4‴$_{quinoline}$), 138.4 (1C, C-1′$_{4-\{[4-(morpholinomethyl)phenyl]ethynyl\}phenyl}$), 138.8 (1C, C-4″$_{4-(morpholinomethyl)phenyl}$), 148.5 (1C, C-8a‴$_{quinoline}$), 148.8 (1C, C-2‴$_{quinoline}$), 165.2 (1C, *C*ONH), 170.9 (1C, *C*O_2CH_3); IR (neat): $\tilde{\nu}$ [cm^{-1}] = 3308, 2949, 2805, 1741, 1655, 1517, 1302, 1203, 1113, 1006, 864, 788, 513; HRMS (*m*/*z*): $[M+H]^+$ calcd for $C_{34}H_{34}N_3O_5$: 564.2493, found: 564.2491.

Methyl (*S*)-3-(1*H*-indole-2-carboxamido)-2-[(4-{[4-(morpholinomethyl)phenyl]ethynyl}benzyl)oxy]propanoate ((*S*)-39g)

Under N_2 atmosphere, copper(I) iodide (20 mg, 0.11 mmol), bis(triphenylphosphine)palladium(II) chloride (43 mg, 0.061 mmol), and diisopropylamine (3 mL) were added to a solution of (*S*)-**38g** (270 mg, 0.57 mmol) in dry THF (20 mL) at ambient temperature and the mixture was stirred for 20 min. Then, 4-(4-ethynylbenzyl)morpholine (340 mg, 1.7 mmol) was added in two portions at an interval of 30 min. After stirring the reaction mixture for 72 h at ambient temperature, the solvent was removed *in vacuo*. The residue was dissolved in ethyl acetate and filtered through a short silica gel column. The solvent was removed *in vacuo* and the residue was purified by flash column chromatography (Ø = 4 cm, h = 19 cm, V = 30 mL, ethyl acetate, R_f = 0.37) to give (*S*)-**39g** as colorless solid (280 mg, 0.50 mmol, 87%). m.p.: 175 °C; $[\alpha]_D^{20}$ = -9.5 (1.1, methanol); HPLC (method 1): t_R = 19.8 min, purity 98.4%.

Methyl (*R*)-3-(1*H*-indole-2-carboxamido)-2-[(4-{[4-(morpholinomethyl)phenyl]ethynyl}benzyl)oxy]propanoate ((*R*)-39g)

Under N_2 atmosphere, copper(I) iodide (20 mg, 0.11 mmol), bis(triphenylphosphine)palladium(II) chloride (40 mg, 0.057 mmol), and diisopropylamine (3 mL) were added to a solution of (*R*)-**38g** (250 mg, 0.53 mmol) in dry THF (20 mL) at ambient temperature and the mixture was stirred for 20 min. Then, 4-(4-ethynylbenzyl)morpholine (310 mg, 1.6 mmol) was added in two portions at an interval of 30 min. After stirring the reaction mixture for 72 h at ambient temperature, the solvent was removed *in vacuo*. The residue was dissolved in ethyl acetate and filtered through a short silica gel column. The solvent was removed *in vacuo* and the residue was purified by flash column chromatography (Ø = 4 cm, h = 19 cm, V = 30 mL, ethyl acetate, R_f = 0.37) to give (*R*)-**39g** as colorless solid (270 mg, 0.49 mmol, 91%). m.p.: 175 °C; $[\alpha]_D^{20}$ = +10.4 (1.2, methanol); HPLC (method 1): t_R = 19.8 min, purity 98.5%.

Spectroscopic data of (*S*)-**39g** *and* (*R*)-**39g**:

^{1}H NMR (DMSO-d_6): δ [ppm] = 2.30 – 2.40 (m, 4H, N(CH_2CH$_2$)$_2$O), 3.48 (s, 2H, NCH_2Ar), 3.52 – 3.62 (m, 5H, OCHCH_2NH (1H), N(CH$_2$CH_2)$_2$O), 3.66 – 3.75 (m, 4H, OCHCH_2NH (1H), CO$_2$CH_3), 4.25 (dd, *J* = 7.1/5.0 Hz, 1H, OC*H*CH$_2$NH), 4.52 (d, *J* = 12.5 Hz, 1H, OCH_2Ar), 4.68 (d, *J* = 12.5 Hz, 1H, OCH_2Ar), 7.00 – 7.07 (m, 1H, 5'''-H_{indole}), 7.12 – 7.21 (m, 2H, 3'''-H_{indole}, 6'''-H_{indole}), 7.32 – 7.38 (m, 2H, 3''-$H_{4\text{-(morpholinomethyl)phenyl}}$, 5''-$H_{4\text{-(morpholinomethyl)phenyl}}$), 7.38 – 7.46 (m, 5H, 2'-$H_{4\text{-\{[4-(morpholinomethyl)phenyl]ethynyl\}phenyl}}$, 3'-$H_{4\text{-\{[4-(morpholinomethyl)phenyl]ethynyl\}phenyl}}$, 5'-$H_{4\text{-\{[4-(morpholinomethyl)phenyl]ethynyl\}phenyl}}$, 6'-$H_{4\text{-\{[4-(morpholinomethyl)phenyl]ethynyl\}phenyl}}$, 7'''-$H_{indole}$), 7.46 – 7.51 (m, 2H, 2''-$H_{4\text{-(morpholinomethyl)phenyl}}$, 6''-$H_{4\text{-(morpholinomethyl)phenyl}}$), 7.59 – 7.65 (m, 1H, 4'''-

H_{indole}), 8.69 (t, *J* = 6.1 Hz, 1H, CON*H*), 11.54 – 11.60 (m, 1H, 1'''-H_{indole}); ^{13}C NMR (DMSO-*d*$_6$): δ [ppm] = 41.0 (1C, OCH*C*H_2NH), 51.9 (1C, CO_2*C*H_3), 53.1 (2C, N(*C*$H_2CH_2)_2$O), 62.0 (1C, Ar*C*H_2N), 66.2 (2C, N(CH_2*C*$H_2)_2$O), 71.0 (1C, O*C*H_2Ar), 76.8 (1C, O*C*HCH$_2$NH), 89.0 (1C, *C*≡C), 89.3 (1C, *C*≡C), 102.8 (1C, C-3'''$_{indole}$), 112.3 (1C, C-7'''$_{indole}$), 119.7 (1C, C-5'''$_{indole}$), 120.8 (1C, C-1''$_{4\text{-(morpholinomethyl)phenyl}}$), 121.46 (1C, C-4'$_{4\text{-\{[4-(morpholinomethyl)phenyl]ethynyl\}phenyl}}$), 121.52 (1C, C-4'''$_{indole}$), 123.3 (1C, C-6'''$_{indole}$), 127.0 (1C, C-3a'''$_{indole}$), 127.8 (2C, C-2'$_{4\text{-\{[4-(morpholinomethyl)phenyl]ethynyl\}phenyl}}$, C-6'$_{4\text{-\{[4-(morpholinomethyl)phenyl]ethynyl\}phenyl}}$), 129.1 (2C, C-3''$_{4\text{-(morpholinomethyl)phenyl}}$, C-5''$_{4\text{-(morpholinomethyl)phenyl}}$), 131.15 (2C, $C_{arom.}$), 131.21 (2C, $C_{arom.}$), 131.4 (1C, C-2'''$_{indole}$), 136.5 (1C, C-7a'''$_{indole}$), 138.4 (1C, C-1'$_{4\text{-\{[4-(morpholinomethyl)phenyl]ethynyl\}phenyl}}$), 138.8 (1C, C-4''$_{4\text{-(morpholinomethyl)phenyl}}$), 161.3 (1C, *C*ONH), 171.0 (1C, *C*O_2CH_3); IR (neat): $\tilde{\nu}$ [cm^{-1}] = 3248, 1746, 1643, 1557, 1421, 1310, 1261, 1202, 1130, 1114, 1009, 868, 817, 776, 744, 517, 434; HRMS (*m*/*z*): [M+H]$^+$ calcd for $C_{33}H_{34}N_3O_5$: 552.2493, found: 552.2504.

Methyl (*S*)-3-(1*H*-indole-3-carboxamido)-2-[(4-{[4-(morpholinomethyl)phenyl]ethynyl}benzyl)oxy]propanoate ((*S*)-39h)

Under N_2 atmosphere, copper(I) iodide (18 mg, 0.095 mmol), bis(triphenylphosphine)palladium(II) chloride (33 mg, 0.047 mmol), and diisopropylamine (3 mL) were added to a solution of (*S*)-**38h** (230 mg, 0.48 mmol) in dry THF (20 mL) at ambient temperature and the mixture was stirred for 20 min. Then, 4-(4-ethynylbenzyl)morpholine (280 mg, 1.4 mmol) was added in two portions at an interval of 30 min. After stirring the reaction mixture for 72 h at ambient temperature, the solvent was removed *in vacuo*. The residue was dissolved in a mixture of dichloromethane and methanol (20/1) and filtered through a short silica gel column. The solvent was removed *in vacuo* and the residue was purified by flash column

chromatography (Ø = 3 cm, h = 24 cm, V = 20 mL, dichloromethane/methanol = 20/1, R_f = 0.31) to give (*S*)-**39h** as yellow solid (260 mg, 0.47 mmol, 98%). m.p.: 141 °C; $[\alpha]_D^{20}$ = -38.2 (1.7, methanol); HPLC (method 1): t_R = 18.6 min, purity 96.0%.

Methyl (*R*)-3-(1*H*-indole-3-carboxamido)-2-[(4-{[4-(morpholinomethyl)phenyl]ethynyl}benzyl)oxy]propanoate ((*R*)-39h)

Under N_2 atmosphere, copper(I) iodide (10 mg, 0.053 mmol), bis(triphenylphosphine)palladium(II) chloride (34 mg, 0.048 mmol), and diisopropylamine (3 mL) were added to a solution of (*R*)-**38h** (210 mg, 0.43 mmol) in dry THF (15 mL) at ambient temperature and the mixture was stirred for 20 min. Then, 4-(4-ethynylbenzyl)morpholine (280 mg, 1.4 mmol) was added in two portions at an interval of 30 min. After stirring the reaction mixture for 72 h at ambient temperature, the solvent was removed *in vacuo*. The residue was dissolved in a mixture of dichloromethane and methanol (20/1) and filtered through a short silica gel column. The solvent was removed *in vacuo* and the residue was purified by flash column chromatography (Ø = 3 cm, h = 27 cm, V = 20 mL, dichloromethane/methanol = 20/1, R_f = 0.31) to give (*R*)-**39h** as yellow solid (220 mg, 0.40 mmol, 93%). m.p.: 141 °C; $[\alpha]_D^{20}$ = +40.9 (1.2, methanol); HPLC (method 1): t_R = 18.6 min, purity 95.3%.

Spectroscopic data of (*S*)-**39h** *and* (*R*)-**39h**:

^{1}H NMR (DMSO-d_6): δ [ppm] = 2.29 – 2.40 (m, 4H, $N(CH_2CH_2)_2O$), 3.44 – 3.62 (m, 7H, NCH_2Ar, $OCHCH_2NH$ (1H), $N(CH_2CH_2)_2O$), 3.63 – 3.74 (m, 4H, $OCHCH_2NH$ (1H), CO_2CH_3), 4.24 (dd, *J* = 7.2/5.1 Hz, 1H, $OCHCH_2NH$), 4.52 (d, *J* = 12.4 Hz, 1H, OCH_2Ar), 4.67 (d, *J* = 12.4 Hz, 1H, OCH_2Ar), 7.06 – 7.19 (m, 2H, 5'''-H_{indole}, 6'''-H_{indole}),

7.32 – 7.53 (m, 9H, 2'-$H_{4\text{-}\{[4\text{-(morpholinomethyl)phenyl]ethynyl}\}phenyl}$, 3'-$H_{4\text{-}\{[4\text{-(morpholinomethyl)phenyl]ethynyl}\}phenyl}$, 5'-$H_{4\text{-}\{[4\text{-(morpholinomethyl)phenyl]ethynyl}\}phenyl}$, 6'-$H_{4\text{-}\{[4\text{-(morpholinomethyl)phenyl]ethynyl}\}phenyl}$, 2''-$H_{4\text{-(morpholinomethyl)phenyl}}$, 3''-$H_{4\text{-(morpholinomethyl)phenyl}}$, 5''-$H_{4\text{-(morpholinomethyl)phenyl}}$, 6''-$H_{4\text{-(morpholinomethyl)phenyl}}$, 7'''-$H_{indole}$), 8.04 (d, *J* = 2.9 Hz, 1H, 2'''-H_{indole}), 8.09 – 8.16 (m, 2H, CON*H*, 4'''-H_{indole}), 11.51 – 11.59 (m, 1H, 1'''-H_{indole}); ^{13}C NMR (DMSO-*d$_6$*): δ [ppm] = 40.9 (1C, OCH*C*H_2NH), 51.9 (1C, CO_2*C*H_3), 53.2 (2C, N(*C*$H_2CH_2)_2$O), 62.0 (1C, Ar*C*H_2N), 66.2 (2C, N(CH_2*C*$H_2)_2$O), 71.0 (1C, O*C*H_2Ar), 77.2 (1C, O*C*HCH_2NH), 89.0 (1C, *C*≡C), 89.3 (1C, *C*≡C), 110.3 (1C, C-3'''$_{indole}$), 111.8 (1C, C-7'''$_{indole}$), 120.4 (1C, C-5'''$_{indole}$), 120.8 (1C, C-1''$_{4\text{-(morpholinomethyl)phenyl}}$), 121.0 (1C, C-4'''$_{indole}$), 121.4 (1C, C-4'$_{4\text{-}\{[4\text{-(morpholinomethyl)phenyl]ethynyl}\}phenyl}$), 121.9 (1C, C-6'''$_{indole}$), 126.1 (1C, C-3a'''$_{indole}$), 127.8 (2C, C-2'$_{4\text{-}\{[4\text{-(morpholinomethyl)phenyl]ethynyl}\}phenyl}$, C-6'$_{4\text{-}\{[4\text{-(morpholinomethyl)phenyl]ethynyl}\}phenyl}$), 128.0 (1C, C-2'''$_{indole}$), 129.2 (2C, C-3''$_{4\text{-(morpholinomethyl)phenyl}}$, C-5''$_{4\text{-(morpholinomethyl)phenyl}}$), 131.2 (2C, $C_{arom.}$), 131.3 (2C, $C_{arom.}$), 136.1 (1C, C-7a'''$_{indole}$), 138.5 (1C, C-1'$_{4\text{-}\{[4\text{-(morpholinomethyl)phenyl]ethynyl}\}phenyl}$), 138.9 (1C, C-4''$_{4\text{-(morpholinomethyl)phenyl}}$), 164.8 (1C, *C*ONH), 171.2 (1C, *C*O_2CH_3); IR (neat): $\tilde{\nu}$ [cm^{-1}] = 3168, 2854, 2800, 1737, 1599, 1537, 1437, 1200, 1113, 1099, 1006, 865, 830, 789, 749, 512; HRMS (*m*/*z*): $[M+H]^+$ calcd for $C_{33}H_{34}N_3O_5$: 552.2493, found: 552.2482.

Methyl (*S*)-3-(1*H*-indole-4-carboxamido)-2-[(4-{[4-(morpholinomethyl)phenyl]ethynyl}benzyl)oxy]propanoate ((*S*)-39i)

Under N_2 atmosphere, copper(I) iodide (13 mg, 0.068 mmol), bis(triphenylphosphine)palladium(II) chloride (32 mg, 0.046 mmol), and diisopropylamine (3 mL) were added to a solution of (*S*)-**38i** (210 mg, 0.45 mmol) in dry THF (10 mL) at ambient temperature and the mixture was stirred for 20 min. Then, 4-(4-ethynylbenzyl)morpholine (330 mg, 1.6 mmol) was added in two portions at an

interval of 30 min. After stirring the reaction mixture for 24 h at ambient temperature, the solvent was removed *in vacuo*. The residue was dissolved in a mixture of petroleum ether and ethyl acetate (1/4) and filtered through a short silica gel column. The solvent was removed *in vacuo* and the residue was purified by flash column chromatography (Ø = 3 cm, h = 8 cm, V = 20 mL, petroleum ether/ethyl acetate = 1/4, R_f = 0.20) to give (*S*)-**39i** as orange solid (160 mg, 0.28 mmol, 64%). m.p.: 65 °C; $[\alpha]_D^{20}$ = -29.8 (2.0, methanol); ^{1}H NMR (DMSO-d_6): δ [ppm] = 2.29 – 2.40 (m, 4H, N(CH_2CH$_2$)$_2$O), 3.49 (s, 2H, NCH_2Ar), 3.55 – 3.64 (m, 5H, OCHCH_2NH (1H), N(CH$_2$CH_2)$_2$O), 3.67 – 3.73 (m, 4H, OCHCH_2NH (1H), CO$_2$CH_3), 4.30 (dd, *J* = 6.9/5.3 Hz, 1H, OC*H*CH$_2$NH), 4.54 (d, *J* = 12.3 Hz, 1H, OCH_2Ar), 4.67 (d, *J* = 12.3 Hz, 1H, OCH_2Ar), 6.81 – 6.84 (m, 1H, 3'''-H$_{indole}$), 7.12 – 7.16 (m, 1H, 6'''-H$_{indole}$), 7.34 – 7.37 (m, 2H, 3''-H$_{4\text{-(morpholinomethyl)phenyl}}$, 5''-H$_{4\text{-(morpholinomethyl)phenyl}}$), 7.37 – 7.40 (m, 1H, 5'''-H$_{indole}$), 7.40 – 7.44 (m, 3H, 2'-H$_{4\text{-\{[4-(morpholinomethyl)phenyl]ethynyl\}phenyl}}$, 6'-H$_{4\text{-\{[4-(morpholinomethyl)phenyl]ethynyl\}phenyl}}$, 2'''-H$_{indole}$), 7.45 – 7.49 (m, 2H, 3'-H$_{4\text{-\{[4-(morpholinomethyl)phenyl]ethynyl\}phenyl}}$, 5'-H$_{4\text{-\{[4-(morpholinomethyl)phenyl]ethynyl\}phenyl}}$), 7.49 – 7.52 (m, 2H, 2''-H$_{4\text{-(morpholinomethyl)phenyl}}$, 6''-H$_{4\text{-(morpholinomethyl)phenyl}}$), 7.53 – 7.56 (m, 1H, 7'''-H$_{indole}$), 8.34 (t, *J* = 5.9 Hz, 1H, CON*H*), 11.29 (s br, 1H, 1'''-H$_{indole}$); ^{13}C NMR (DMSO-d_6): δ [ppm] = 41.2 (1C, OCH*C*H$_2$NH), 51.9 (1C, CO$_2$*C*H$_3$), 53.2 (2C, N(*C*H$_2$CH$_2$)$_2$O), 62.0 (1C, Ar*C*H$_2$N), 66.2 (2C, N(CH$_2$*C*H$_2$)$_2$O), 70.9 (1C, O*C*H$_2$Ar), 76.9 (1C, O*C*HCH$_2$NH), 89.0 (1C, *C*≡C), 89.3 (1C, *C*≡C), 101.7 (1C, C-3'''$_{indole}$), 114.2 (1C, C-7'''$_{indole}$), 118.6 (1C, C-5'''$_{indole}$), 120.1 (1C, C-6'''$_{indole}$), 120.8 (1C, C-1''$_{4\text{-(morpholinomethyl)phenyl}}$), 121.5 (1C, C-4'$_{4\text{-\{[4-(morpholinomethyl)phenyl]ethynyl\}phenyl}}$), 125.8 (1C, C-3a'''$_{indole}$), 126.4 (1C, C-4'''$_{indole}$), 126.5 (1C, C-2'''$_{indole}$), 127.8 (2C, C-2'$_{4\text{-\{[4-(morpholinomethyl)phenyl]ethynyl\}phenyl}}$, C-6'$_{4\text{-\{[4-(morpholinomethyl)phenyl]ethynyl\}phenyl}}$), 129.2 (2C, C-3''$_{4\text{-(morpholinomethyl)phenyl}}$, C-5''$_{4\text{-(morpholinomethyl)phenyl}}$), 131.19 (2C, C$_{arom.}$), 131.24 (2C, C$_{arom.}$), 136.5 (1C, C-7a'''$_{indole}$), 138.5 (1C, C-1'$_{4\text{-\{[4-(morpholinomethyl)phenyl]ethynyl\}phenyl}}$), 138.9 (1C, C-

4''$_{4-(morpholinomethyl)phenyl}$), 168.1 (1C, *C*ONH), 171.1 (1C, *C*O_2CH_3); IR (neat): $\tilde{\nu}$ [cm^{-1}] = 3272, 2855, 2810, 1737, 1642, 1606, 1517, 1344, 1291, 1195, 1111, 1005, 863, 819, 759, 514; HRMS (*m*/*z*): [M+H]$^+$ calcd for $C_{33}H_{34}N_3O_5$: 552.2493, found: 552.2468; HPLC (method 1): t_R = 18.6 min, purity 95.8%.

Methyl (*S*)-3-(1*H*-indole-5-carboxamido)-2-[(4-{[4-(morpholinomethyl)phenyl]ethynyl}benzyl)oxy]propanoate ((*S*)-39j)

Under N_2 atmosphere, copper(I) iodide (8.1 mg, 0.043 mmol), bis(triphenylphosphine)palladium(II) chloride (21 mg, 0.030 mmol), and diisopropylamine (3 mL) were added to a solution of (*S*)-**38j** (130 mg, 0.28 mmol) in dry THF (10 mL) at ambient temperature and the mixture was stirred for 20 min. Then, 4-(4-ethynylbenzyl)morpholine (140 mg, 0.70 mmol) was added in two portions at an interval of 30 min. After stirring the reaction mixture for 24 h at ambient temperature, the solvent was removed *in vacuo*. The residue was dissolved in a mixture of dichloromethane and methanol (20/1) and filtered through a short silica gel column. The solvent was removed *in vacuo* and the residue was purified by flash column chromatography (Ø = 3 cm, h = 24 cm, V = 20 mL, dichloromethane/methanol = 20/1, R_f = 0.36) to give (*S*)-**39j** as yellow solid (130 mg, 0.23 mmol, 83%). m.p.: 72 °C; $[\alpha]_D^{20}$ = -17.0 (1.2, methanol); HPLC (method 1): t_R = 18.5 min, purity 98.8%.

Methyl (*R*)-3-(1*H*-indole-5-carboxamido)-2-[(4-{[4-(morpholinomethyl)phenyl]ethynyl}benzyl)oxy]propanoate ((*R*)-39j)

Under N_2 atmosphere, copper(I) iodide (7.0 mg, 0.037 mmol), bis(triphenylphosphine)palladium(II) chloride (23 mg, 0.033 mmol), and

diisopropylamine (3 mL) were added to a solution of (*R*)-**38j** (150 mg, 0.31 mmol) in dry THF (12 mL) at ambient temperature and the mixture was stirred for 20 min. Then, 4-(4-ethynylbenzyl)morpholine (150 mg, 0.74 mmol) was added in two portions at an interval of 30 min. After stirring the reaction mixture for 24 h at ambient temperature, the solvent was removed *in vacuo*. The residue was dissolved in a mixture of dichloromethane and methanol (50/1) and filtered through a short silica gel column. The solvent was removed *in vacuo* and the residue was purified by flash column chromatography (Ø = 3 cm, h = 23 cm, V = 20 mL, dichloromethane/methanol = 50/1, R_f = 0.11) to give (*R*)-**39j** as yellow solid (130 mg, 0.23 mmol, 76%). m.p.: 72 °C; $[\alpha]_D^{20}$ = +17.1 (4.2, methanol); HPLC (method 1): t_R = 18.5 min, purity 98.9%.

Spectroscopic data of (*S*)-**39j** *and* (*R*)-**39j**:

^{1}H NMR (DMSO-d_6): δ [ppm] = 2.29 – 2.41 (m, 4H, N(CH_2CH$_2$)$_2$O), 3.49 (s, 2H, NCH_2Ar), 3.51 – 3.60 (m, 5H, OCHCH_2NH (1H), N(CH$_2$CH_2)$_2$O), 3.64 – 3.71 (m, 4H, OCHCH_2NH (1H), CO$_2$CH_3), 4.26 (dd, *J* = 7.2/5.3 Hz, 1H, OC*H*CH$_2$NH), 4.51 (d, *J* = 12.5 Hz, 1H, OCH_2Ar), 4.66 (d, *J* = 12.5 Hz, 1H, OCH_2Ar), 6.52 – 6.55 (m, 1H, 3'''-H_{indole}), 7.34 – 7.37 (m, 2H, 3''-$H_{4\text{-(morpholinomethyl)phenyl}}$, 5''-$H_{4\text{-(morpholinomethyl)phenyl}}$), 7.38 – 7.41 (m, 2H, 2'-$H_{4\text{-\{[4-(morpholinomethyl)phenyl]ethynyl\}phenyl}}$, 6'-$H_{4\text{-\{[4-(morpholinomethyl)phenyl]ethynyl\}phenyl}}$), 7.41 – 7.44 (m, 2H, 2'''-H_{indole}, 7'''-H_{indole}), 7.44 – 7.47 (m, 2H, 3'-$H_{4\text{-\{[4-(morpholinomethyl)phenyl]ethynyl\}phenyl}}$, 5'-$H_{4\text{-\{[4-(morpholinomethyl)phenyl]ethynyl\}phenyl}}$), 7.48 – 7.51 (m, 2H, 2''-$H_{4\text{-(morpholinomethyl)phenyl}}$, 6''-$H_{4\text{-(morpholinomethyl)phenyl}}$), 7.61 (dd, *J* = 8.5/1.5 Hz, 1H, 6'''-H_{indole}), 8.09 – 8.12 (m, 1H, 4'''-H_{indole}), 8.50 (t, *J* = 5.9 Hz, 1H, CON*H*), 11.32 (s br, 1H, 1'''-H_{indole}); ^{13}C NMR (DMSO-d_6): δ [ppm] = 41.5 (1C, OCH*C*H$_2$NH), 51.9 (1C, CO$_2$*C*H$_3$), 53.2 (2C, N(*C*H$_2$CH$_2$)$_2$O), 62.0 (1C, Ar*C*H$_2$N), 66.2 (2C, N(CH$_2$*C*H$_2$)$_2$O), 70.9 (1C, O*C*H$_2$Ar), 76.9 (1C, O*C*HCH$_2$NH), 89.0 (1C, *C*≡C), 89.3 (1C, *C*≡C), 102.1 (1C, C-

3'''$_{indole}$), 110.9 (1C, C-7'''$_{indole}$), 120.0 (1C, C-4'''$_{indole}$), 120.5 (1C, C-6'''$_{indole}$), 120.8 (1C, C-1''$_{4\text{-(morpholinomethyl)phenyl}}$), 121.4 (1C, C-4'$_{4\text{-\{[4-(morpholinomethyl)phenyl]ethynyl\}phenyl}}$), 125.2 (1C, C-5'''$_{indole}$), 126.7 (1C, C-2'''$_{indole}$), 127.0 (1C, C-3a'''$_{indole}$), 127.8 (2C, C-2'$_{4\text{-\{[4-(morpholinomethyl)phenyl]ethynyl\}phenyl}}$, C-6'$_{4\text{-\{[4-(morpholinomethyl)phenyl]ethynyl\}phenyl}}$), 129.2 (2C, C-3''$_{4\text{-(morpholinomethyl)phenyl}}$, C-5''$_{4\text{-(morpholinomethyl)phenyl}}$), 131.17 (2C, $C_{arom.}$), 131.23 (2C, $C_{arom.}$), 137.4 (1C, C-7a'''$_{indole}$), 138.5 (1C, C-1'$_{4\text{-\{[4-(morpholinomethyl)phenyl]ethynyl\}phenyl}}$), 138.8 (1C, C-4''$_{4\text{-(morpholinomethyl)phenyl}}$), 167.6 (1C, *C*ONH), 171.2 (1C, *C*O_2CH_3); IR (neat): $\tilde{\nu}$ [cm^{-1}] = 3260, 2856, 2808, 1737, 1636, 1611, 1518, 1203, 1110, 1005, 863, 818, 753, 726, 513; HRMS (*m*/*z*): $[M+H]^+$ calcd for $C_{33}H_{34}N_3O_5$: 552.2493, found: 552.2487.

Methyl (*S*)-2-[(4-{[4-(morpholinomethyl)phenyl]ethynyl}benzyl)oxy]-3-(1*H*-pyrrole-2-carboxamido)propanoate ((*S*)-39k)

Under N_2 atmosphere, copper(I) iodide (6 mg, 0.032 mmol), bis(triphenylphosphine)palladium(II) chloride (26 mg, 0.037 mmol), and diisopropylamine (2.5 mL) were added to a solution of (*S*)-**38k** (110 mg, 0.26 mmol) in dry THF (10 mL) at ambient temperature and the mixture was stirred for 20 min. Then, 4-(4-ethynylbenzyl)morpholine (120 mg, 0.61 mmol) was added in two portions at an interval of 30 min. After stirring the reaction mixture for 24 h at ambient temperature, the solvent was removed *in vacuo*. The residue was dissolved in a mixture of dichloromethane and methanol (20/1) and filtered through a short silica gel column. The solvent was removed *in vacuo* and the residue was purified by flash column chromatography (Ø = 2 cm, h = 26 cm, V = 10 mL, dichloromethane/methanol = 20/1, R_f = 0.29) to give (*S*)-**39k** as colorless solid (100 mg, 0.20 mmol, 78%). m.p.: 135 °C; $[\alpha]_D^{20}$ = -42.1 (1.5, methanol); ^{1}H NMR (DMSO-*d*$_6$): δ [ppm] = 2.31 – 2.39 (m, 4H, N(C*H*$_2$CH$_2$)$_2$O), 3.44 – 3.51 (m, 3H, OCHC*H*$_2$NH (1H), NC*H*$_2$Ar), 3.55 – 3.65 (m, 5H,

OCHCH_2NH (1H), N(CH_2CH_2)$_2$O), 3.68 (s, 3H, CO_2CH_3), 4.18 (dd, *J* = 7.2/5.0 Hz, 1H, OC*H*CH_2NH), 4.50 (d, *J* = 12.5 Hz, 1H, OCH_2Ar), 4.65 (d, *J* = 12.5 Hz, 1H, OCH_2Ar), 6.07 – 6.11 (m, 1H, 4'''-$H_{pyrrole}$), 6.78 – 6.81 (m, 1H, 3'''-$H_{pyrrole}$), 6.84 – 6.87 (m, 1H, 5'''-$H_{pyrrole}$), 7.34 – 7.37 (m, 2H, 3''-$H_{4-(morpholinomethyl)phenyl}$, 5''-$H_{4-(morpholinomethyl)phenyl}$), 7.37 – 7.41 (m, 2H, 2'-$H_{4-\{[4-(morpholinomethyl)phenyl]ethynyl\}phenyl}$, 6'-$H_{4-\{[4-(morpholinomethyl)phenyl]ethynyl\}phenyl}$), 7.43 – 7.47 (m, 2H, 3'-$H_{4-\{[4-(morpholinomethyl)phenyl]ethynyl\}phenyl}$, 5'-$H_{4-\{[4-(morpholinomethyl)phenyl]ethynyl\}phenyl}$), 7.48 – 7.52 (m, 2H, (m, 4H, 2''-$H_{4-(morpholinomethyl)phenyl}$, 6''-$H_{4-(morpholinomethyl)phenyl}$), 8.20 (t, *J* = 6.0 Hz, 1H, CON*H*), 11.44 (s br, 1H, 1'''-$H_{pyrrole}$); ^{13}C NMR (DMSO-*d$_6$*): δ [ppm] = 40.8 (1C, OCH*C*H_2NH), 51.9 (1C, CO_2CH_3), 53.2 (2C, N(CH_2CH_2)$_2$O), 62.0 (1C, Ar*C*H_2N), 66.2 (2C, N(CH_2CH_2)$_2$O), 70.9 (1C, O*C*H_2Ar), 77.0 (1C, O*C*HCH_2NH), 89.0 (1C, *C*≡C), 89.3 (1C, *C*≡C), 108.6 (1C, C-4'''$_{pyrrole}$), 110.3 (1C, C-3'''$_{pyrrole}$), 120.8 (1C, C-1''$_{4-(morpholinomethyl)phenyl}$), 121.4 (2C, C-4'$_{4-\{[4-(morpholinomethyl)phenyl]ethynyl\}phenyl}$, C-5'''$_{pyrrole}$), 126.0 (1C, C-2'''$_{pyrrole}$), 127.8 (2C, C-2'$_{4-\{[4-(morpholinomethyl)phenyl]ethynyl\}phenyl}$, C-6'$_{4-\{[4-(morpholinomethyl)phenyl]ethynyl\}phenyl}$), 129.2 (2C, C-3''$_{4-(morpholinomethyl)phenyl}$, C-5''$_{4-(morpholinomethyl)phenyl}$), 131.15 (2C, $C_{arom.}$), 131.24 (2C, $C_{arom.}$), 138.5 (1C, C-1'$_{4-\{[4-(morpholinomethyl)phenyl]ethynyl\}phenyl}$), 138.9 (1C, C-4''$_{4-(morpholinomethyl)phenyl}$), 160.8 (1C, *C*ONH), 171.1 (1C, *C*O_2CH_3); IR (neat): $\tilde{\nu}$ [cm^{-1}] = 3241, 2947, 2813, 1744, 1633, 1565, 1533, 1410, 1320, 1201, 1125, 1109, 1034, 1007, 857, 823, 788, 747, 614, 505; HRMS (*m*/*z*): [M+H]$^+$ calcd for $C_{29}H_{32}N_3O_5$: 502.2336, found: 502.2362; HPLC (method 1): t_R = 18.0 min, purity 96.1%.

Methyl (*S*)-2-[(4-{[4-(morpholinomethyl)phenyl]ethynyl}benzyl)oxy]-3-(1*H*-pyrazole-3-carboxamido)propanoate ((*S*)-39l)

Under N_2 atmosphere, copper(I) iodide (7 mg, 0.037 mmol), bis(triphenylphosphine)palladium(II) chloride (20 mg, 0.028 mmol), and

diisopropylamine (3 mL) were added to a solution of (*S*)-**38l** (110 mg, 0.27 mmol) in dry THF (10 mL) at ambient temperature and the mixture was stirred for 20 min. Then, 4-(4-ethynylbenzyl)morpholine (160 mg, 0.80 mmol) was added in two portions at an interval of 30 min. After stirring the reaction mixture for 16 h at ambient temperature, the solvent was removed *in vacuo*. The residue was dissolved in a mixture of dichloromethane and methanol (20/1) and filtered through a short silica gel column. The solvent was removed *in vacuo* and the residue was purified by automatic flash column chromatography using a Biotage® Isolera™ One system (30% → 80% ACN in H_2O, Biotage® SNAP C18 12 g). Fractions containing the desired product were combined and subjected to lyophilization to give (*S*)-**39l** as colorless solid (100 mg, 0.21 mmol, 78%). m.p.: 62 °C; R_f = 0.29 (dichloromethane/methanol = 20/1); $[\alpha]_D^{20}$ = -36.0 (1.3, methanol); ^{1}H NMR (DMSO-d_6): δ [ppm] = 2.29 – 2.41 (m, 4H, $N(CH_2CH_2)_2O$), 3.49 (s, 2H, NCH_2Ar), 3.49 – 3.67 (m, 6H, $N(CH_2CH_2)_2O$, $OCHCH_2NH$), 3.67 (s, 3H, CO_2CH_3), 4.19 – 4.28 (m, 1H, $OCHCH_2NH$), 4.50 (d, *J* = 12.5 Hz, 1H, OCH_2Ar), 4.65 (d, *J* = 12.5 Hz, 1H, OCH_2Ar), 6.61 – 6.67 (m, 1H, 4'''-$H_{pyrazole}$), 7.33 – 7.42 (m, 4H, 2'-$H_{4\text{-}\{[4\text{-}(morpholinomethyl)phenyl]ethynyl\}phenyl}$, 6'-$H_{4\text{-}\{[4\text{-}(morpholinomethyl)phenyl]ethynyl\}phenyl}$, 3''-$H_{4\text{-}(morpholinomethyl)phenyl}$, 5''-$H_{4\text{-}(morpholinomethyl)phenyl}$), 7.43 – 7.54 (m, 4H, 3'-$H_{4\text{-}\{[4\text{-}(morpholinomethyl)phenyl]ethynyl\}phenyl}$, 5'-$H_{4\text{-}\{[4\text{-}(morpholinomethyl)phenyl]ethynyl\}phenyl}$, 2''-$H_{4\text{-}(morpholinomethyl)phenyl}$, 6''-$H_{4\text{-}(morpholinomethyl)phenyl}$), 7.80 – 7.85 (m, 1H, 5'''-$H_{pyrazole}$), 8.12 – 8.20 (m, 1H, CON*H*), 13.26 (s br, 1H, 1'''-$H_{pyrazole}$); ^{13}C NMR (DMSO-d_6): δ [ppm] = 40.5 (1C, $OCHCH_2NH$), 51.9 (1C, CO_2CH_3), 53.1 (2C, $N(CH_2CH_2)_2O$), 62.0 (1C, $ArCH_2N$), 66.2 (2C, $N(CH_2CH_2)_2O$), 70.9 (1C, OCH_2Ar), 76.7 (1C, $OCHCH_2NH$), 89.0 (1C, *C*≡C), 89.3 (1C, *C*≡C), 105.0 (1C, C-4'''$_{pyrazole}$), 120.8 (1C, $C_{arom.}$), 121.4 (1C, $C_{arom.}$), 127.7 (2C, C-2'$_{4\text{-}\{[4\text{-}(morpholinomethyl)phenyl]ethynyl\}phenyl}$, C-6'$_{4\text{-}\{[4\text{-}(morpholinomethyl)phenyl]ethynyl\}phenyl}$), 129.1 (2C, C-3''$_{4\text{-}(morpholinomethyl)phenyl}$, C-5''$_{4\text{-}}$

(morpholinomethyl)phenyl), 131.1 (2C, $C_{arom.}$), 131.2 (2C, $C_{arom.}$), 138.5 (1C, C-1'$_{4\text{-}\{[4\text{-}(morpholinomethyl)phenyl]ethynyl\}phenyl}$), 138.8 (1C, C-4''$_{4\text{-}(morpholinomethyl)phenyl}$), 171.0 (1C, CO_2CH_3), the signals for C-3'''$_{pyrazole}$, C-5'''$_{pyrazole}$, and *C*ONH cannot be observed in the spectrum; IR (neat): $\tilde{\nu}$ [cm^{-1}] = 3207, 2951, 1744, 1651, 1545, 1207, 1114, 1006, 864, 820, 794, 763, 616, 540, 516; HRMS (*m*/*z*): $[M+H]^+$ calcd for $C_{28}H_{31}N_4O_5$: 503.2289, found: 503.2294; HPLC (method 1): t_R = 16.5 min, purity 97.1%.

Methyl (*S*)-2-[(4-{[4-(morpholinomethyl)phenyl]ethynyl}benzyl)oxy]-3-(3-phenyl-1*H*-pyrazole-5-carboxamido)propanoate ((*S*)-39m)

Under N_2 atmosphere, copper(I) iodide (17 mg, 0.089 mmol), bis(triphenylphosphine)palladium(II) chloride (32 mg, 0.046 mmol), and diisopropylamine (3 mL) were added to a solution of (*S*)-**38m** (230 mg, 0.46 mmol) in dry THF (20 mL) at ambient temperature and the mixture was stirred for 20 min. Then, 4-(4-ethynylbenzyl)morpholine (270 mg, 1.3 mmol) was added in two portions at an interval of 30 min. After stirring the reaction mixture for 16 h at ambient temperature, the solvent was removed *in vacuo*. The residue was dissolved in a mixture of dichloromethane and methanol (20/1) and filtered through a short silica gel column. The solvent was removed *in vacuo* and the residue was purified by flash column chromatography (Ø = 3 cm, h = 27 cm, V = 20 mL, dichloromethane/methanol = 50/1 → 20/1) to give (*S*)-**39m** as yellow solid (240 mg, 0.41 mmol, 88%). m.p.: 144 °C; R_f = 0.34 (dichloromethane/methanol = 20/1); $[\alpha]_D^{20}$ = -21.8 (1.1, methanol); HPLC (method 1): t_R = 19.2 min, purity 98.0%.

Methyl (*R*)-2-[(4-{[4-(morpholinomethyl)phenyl]ethynyl}benzyl)oxy]-3-(3-phenyl-1*H*-pyrazole-5-carboxamido)propanoate ((*R*)-39m)

Under N_2 atmosphere, copper(I) iodide (15 mg, 0.078 mmol), bis(triphenylphosphine)palladium(II) chloride (27 mg, 0.038 mmol), and diisopropylamine (3 mL) were added to a solution of (*R*)-**38m** (190 mg, 0.37 mmol) in dry THF (20 mL) at ambient temperature and the mixture was stirred for 20 min. Then, 4-(4-ethynylbenzyl)morpholine (210 mg, 1.1 mmol) was added in two portions at an interval of 30 min. After stirring the reaction mixture for 24 h at ambient temperature, the solvent was removed *in vacuo*. The residue was dissolved in a mixture of dichloromethane and methanol (20/1) and filtered through a short silica gel column. The solvent was removed *in vacuo* and the residue was purified by flash column chromatography (Ø = 4 cm, h = 25 cm, V = 30 mL, dichloromethane/methanol = 50/1 → 20/1) to give (*R*)-**39m** as yellow solid (210 mg, 0.35 mmol, 96%). m.p.: 143 °C; R_f = 0.34 (dichloromethane/methanol = 20/1); $[\alpha]_D^{20}$ = +25.2 (1.6, methanol); HPLC (method 1): t_R = 19.2 min, purity 98.6%.

Spectroscopic data of (*S*)-**39m** *and* (*R*)-**39m**:

^{1}H NMR (DMSO-d_6): δ [ppm] = 2.29 – 2.41 (m, 4H, N(CH_2CH$_2$)$_2$O), 3.48 (s, 2H, NCH_2Ar), 3.49 – 3.61 (m, 5H, N(CH$_2$CH_2)$_2$O, OCHCH_2NH (1H)), 3.61 – 3.73 (m, 4H, OCHCH_2NH (1H), CO$_2$CH_3), 4.18 – 4.31 (m, 1H, OCHCH$_2$NH), 4.52 (d, *J* = 12.5 Hz, 1H, OCH_2Ar), 4.68 (d, *J* = 12.5 Hz, 1H, OCH_2Ar), 7.08 (d, *J* = 1.3 Hz, 0.7H, 4'''-$H_{pyrazole}$), 7.24 – 7.29 (m, 0.3H, 4'''-$H_{pyrazole}$), 7.29 – 7.52 (m, 11H, 2'-$H_{4\text{-}\{[4\text{-}(morpholinomethyl)phenyl]ethynyl\}phenyl}$, 3'-$H_{4\text{-}\{[4\text{-}(morpholinomethyl)phenyl]ethynyl\}phenyl}$, 5'-$H_{4\text{-}\{[4\text{-}(morpholinomethyl)phenyl]ethynyl\}phenyl}$, 6'-$H_{4\text{-}\{[4\text{-}(morpholinomethyl)phenyl]ethynyl\}phenyl}$, 2''-$H_{4\text{-}(morpholinomethyl)phenyl}$, 3''-$H_{4\text{-}(morpholinomethyl)phenyl}$, 5''-$H_{4\text{-}(morpholinomethyl)phenyl}$, 6''-H4-

(morpholinomethyl)phenyl, 3''''-H$_{phenyl}$, 4''''-H$_{phenyl}$, 5''''-H$_{phenyl}$), 7.73 – 7.85 (m, 2H, 2''''-H$_{phenyl}$, 6''''-H$_{phenyl}$), 8.23 (t, *J* = 5.8 Hz, 0.7H, CON*H*), 8.68 – 8.78 (m, 0.3H, CON*H*), 13.65 (s br, 0.3H, 1'''-H$_{pyrazole}$), 13.69 (s br, 0.7H, 1'''-H$_{pyrazole}$), two annular tautomers exist in the ratio 70:30; ^{13}C NMR (DMSO-*d$_6$*): δ [ppm] = 40.6 (1C, OCH*C*H$_2$NH), 51.9 (1C, CO$_2$*C*H$_3$), 53.2 (2C, N(*C*H$_2$CH$_2$)$_2$O), 62.0 (1C, Ar*C*H$_2$N), 66.2 (2C, N(CH$_2$*C*H$_2$)$_2$O), 70.9 (1C, O*C*H$_2$Ar), 76.7 (1C, O*C*HCH$_2$NH), 89.0 (1C, *C*≡C), 89.3 (1C, *C*≡C), 102.7 (1C, C-4'''$_{pyrazole}$), 120.8 (1C, C-1''$_{4-(morpholinomethyl)phenyl}$), 121.4 (1C, C-4'$_{4-\{[4-(morpholinomethyl)phenyl]ethynyl\}phenyl}$), 125.3 (2C, C-2''''$_{phenyl}$, C-6''''$_{phenyl}$), 127.8 (2C, C-2'$_{4-\{[4-(morpholinomethyl)phenyl]ethynyl\}phenyl}$, C-6'$_{4-\{[4-(morpholinomethyl)phenyl]ethynyl\}phenyl}$), 128.5 (1C, C-4''''$_{phenyl}$), 128.8 (1C, C-1''''$_{phenyl}$), 129.1 (2C, C-3''''$_{phenyl}$, C-5''''$_{phenyl}$), 129.2 (2C, C-3''$_{4-(morpholinomethyl)phenyl}$, C-5''$_{4-(morpholinomethyl)phenyl}$), 131.19 (2C, C$_{arom.}$), 131.24 (2C, C$_{arom.}$), 138.5 (1C, C-1'$_{4-\{[4-(morpholinomethyl)phenyl]ethynyl\}phenyl}$), 138.8 (1C, C-4''$_{4-(morpholinomethyl)phenyl}$), 143.5 (1C, C-3'''$_{pyrazole}$), 147.5 (1C, C-5'''$_{pyrazole}$), 161.8 (1C, *C*ONH), 171.0 (1C, *C*O$_2$CH$_3$), two annular tautomers exist in the ratio 70:30, the signals of the major tautomer are given; IR (neat): $\tilde{\nu}$ [cm^{-1}] = 3425, 3130, 3013, 2853, 2801, 1746, 1641, 1552, 1396, 1348, 1262, 1201, 1131, 1113, 1007, 912, 829, 764, 688, 673, 566, 540, 511; HRMS (*m*/*z*): [M+H]$^+$ calcd for $C_{34}H_{35}N_4O_5$: 579.2602, found: 579.2652.

Methyl (*S*)-3-(3,5-dimethylisoxazole-4-carboxamido)-2-[(4-{[4-(morpholinomethyl)phenyl]ethynyl}benzyl)oxy]propanoate ((*S*)-39n)

Under N_2 atmosphere, copper(I) iodide (13 mg, 0.068 mmol), bis(triphenylphosphine)palladium(II) chloride (41 mg, 0.058 mmol), and diisopropylamine (3 mL) were added to a solution of (*S*)-**38n** (250 mg, 0.55 mmol) in dry THF (20 mL) at ambient temperature and the mixture was stirred for 20 min. Then, 4-(4-ethynylbenzyl)morpholine (340 mg, 1.7 mmol) was added in two portions at an interval of 30 min. After stirring the

reaction mixture for 16 h at ambient temperature, the solvent was removed *in vacuo*. The residue was dissolved in a mixture of dichloromethane and methanol (20/1) and filtered through a short silica gel column. The solvent was removed *in vacuo* and the residue was purified by automatic flash column chromatography using a Biotage® Isolera™ One system (10% → 80% ACN in H_2O, Biotage® SNAP C18 30 g). Fractions containing the desired product were combined and subjected to lyophilization to give (*S*)-**39n** as colorless solid (260 mg, 0.48 mmol, 87%). m.p.: 106 °C; R_f = 0.37 (dichloromethane/methanol = 20/1); $[\alpha]_D^{20}$ = -19.7 (1.2, methanol); HPLC (method 1): t_R = 18.2 min, purity 100%.

Methyl (*R*)-3-(3,5-dimethylisoxazole-4-carboxamido)-2-[(4-{[4-(morpholinomethyl)phenyl]ethynyl}benzyl)oxy]propanoate ((*R*)-39n)

Under N_2 atmosphere, copper(I) iodide (13 mg, 0.068 mmol), bis(triphenylphosphine)palladium(II) chloride (41 mg, 0.058 mmol), and diisopropylamine (3 mL) were added to a solution of (*R*)-**38n** (260 mg, 0.56 mmol) in dry THF (20 mL) at ambient temperature and the mixture was stirred for 20 min. Then, 4-(4-ethynylbenzyl)morpholine (320 mg, 1.6 mmol) was added in two portions at an interval of 30 min. After stirring the reaction mixture for 16 h at ambient temperature, the solvent was removed *in vacuo*. The residue was dissolved in ethyl acetate and filtered through a short silica gel column. The solvent was removed *in vacuo* and the residue was purified by automatic flash column chromatography using a Biotage® Isolera™ One system (10% → 80% ACN in H_2O, Biotage® SNAP C18 12 g). Fractions containing the desired product were combined and subjected to lyophilization to give (*R*)-**39n** as yellow oil (250 mg, 0.47 mmol, 83%). R_f = 0.37 (dichloromethane/methanol = 20/1); $[\alpha]_D^{20}$ = +16.0 (0.8, methanol); HPLC (method 1): t_R = 18.3 min, purity 95.1%.

Spectroscopic data of (*S*)-**39n** *and* (*R*)-**39n**:

^{1}H NMR (DMSO-*d₆*): δ [ppm] = 2.24 (s, 3H, H_3CC-3''$_{isoxazole}$), 2.32 – 2.39 (m, 4H, N(CH_2CH$_2$)$_2$O), 2.46 (s, 3H, H_3CC-5''$_{isoxazole}$), 3.49 (s, 2H, NCH_2Ar), 3.53 – 3.63 (m, 6H, N(CH$_2$CH_2)$_2$O, OCHCH_2NH), 3.68 (s, 3H, CO$_2$CH_3), 4.21 – 4.24 (m, 1H, OC*H*CH$_2$NH), 4.52 (d, *J* = 12.2 Hz, 1H, OCH_2Ar), 4.65 (d, *J* = 12.2 Hz, 1H, OCH_2Ar), 7.34 – 7.38 (m, 2H, 3''-H$_{4-(morpholinomethyl)phenyl}$, 5''-H$_{4-(morpholinomethyl)phenyl}$), 7.39 – 7.43 (m, 2H, 2'-H$_{4-\{[4-(morpholinomethyl)phenyl]ethynyl\}phenyl}$, 6'-H$_{4-\{[4-(morpholinomethyl)phenyl]ethynyl\}phenyl}$), 7.49 – 7.53 (m, 4H, 3'-H$_{4-\{[4-(morpholinomethyl)phenyl]ethynyl\}phenyl}$, 5'-H$_{4-\{[4-(morpholinomethyl)phenyl]ethynyl\}phenyl}$, 2''-H$_{4-(morpholinomethyl)phenyl}$, 6''-H$_{4-(morpholinomethyl)phenyl}$), 8.20 (t, *J* = 6.0 Hz, 1H, CON*H*); ^{13}C NMR (DMSO-*d₆*): δ [ppm] = 10.4 (1C, H$_3$*C*C-3'''$_{isoxazole}$), 12.0 (1C, H$_3$*C*C-5'''$_{isoxazole}$), 40.8 (1C, OCH*C*H$_2$NH), 52.0 (1C, CO$_2$*C*H$_3$), 53.2 (2C, N(*C*H$_2$CH$_2$)$_2$O), 62.0 (1C, Ar*C*H$_2$N), 66.2 (2C, N(CH$_2$*C*H$_2$)$_2$O), 70.9 (1C, O*C*H$_2$Ar), 76.7 (1C, O*C*HCH$_2$NH), 89.0 (1C, *C*≡C), 89.4 (1C, *C*≡C), 112.8 (1C, C-4'''$_{isoxazole}$), 120.8 (1C, C-1''$_{4-(morpholinomethyl)phenyl}$), 121.5 (1C, C-4'$_{4-\{[4-(morpholinomethyl)phenyl]ethynyl\}phenyl}$), 127.9 (2C, C-2'$_{4-\{[4-(morpholinomethyl)phenyl]ethynyl\}phenyl}$, C-6'$_{4-\{[4-(morpholinomethyl)phenyl]ethynyl\}phenyl}$), 129.2 (2C, C-3''$_{4-(morpholinomethyl)phenyl}$, C-5''$_{4-(morpholinomethyl)phenyl}$), 131.20 (2C, C$_{arom.}$), 131.24 (2C, C$_{arom.}$), 138.4 (1C, C-1'$_{4-\{[4-(morpholinomethyl)phenyl]ethynyl\}phenyl}$), 138.9 (1C, C-4''$_{4-(morpholinomethyl)phenyl}$), 158.2 (1C, C-3'''$_{isoxazole}$), 161.6 (1C, *C*ONH), 169.5 (1C, C-5'''$_{isoxazole}$), 170.9 (1C, *C*O$_2$CH$_3$); IR (neat): $\tilde{\nu}$ [cm^{-1}] = 3306, 2954, 2871, 2803, 1739, 1729, 1649, 1537, 1269, 1226, 1205, 1168, 1111, 1040, 1009, 865, 834, 791; HRMS (*m*/*z*): [M+H]$^+$ calcd for $C_{30}H_{34}N_3O_6$: 532.2442, found: 532.2463.

(*S*)-*N*-{3-(Hydroxyamino)-2-[(4-{[4-(morpholinomethyl)phenyl]ethynyl}benzyl)oxy]-3-oxopropyl}-[1,1'-biphenyl]-4-carboxamide ((*S*)-13b)

Under ice-cooling, an aqueous solution of hydroxylamine (50 wt%, 4 mL) was added to a solution of (*S*)-**39b** (100 mg, 0.17 mmol) in a mixture of THF (5 mL) and isopropanol (5 mL). After stirring the reaction mixture for 5 min at 0 °C, stirring was

continued for 48 h at ambient temperature. Then, the solvent was removed *in vacuo* and the residue was triturated with cold water and cold acetonitrile. The desired product was dried on high vacuum to give (*S*)-**13b** as colorless solid (83 mg, 0.14 mmol, 82%). m.p.: 185 °C; $[\alpha]_D^{20}$ = -38.7 (1.5, dimethyl sulfoxide); HPLC (method 2): t_R = 13.7 min, purity 98.0%.

(*R*)-*N*-{3-(Hydroxyamino)-2-[(4-{[4-(morpholinomethyl)phenyl]ethynyl}benzyl)oxy]-3-oxopropyl}-[1,1'-biphenyl]-4-carboxamide ((*R*)-13b)

Under ice-cooling, an aqueous solution of hydroxylamine (50 wt%, 2 mL) was added to a solution of (*R*)-**39b** (120 mg, 0.21 mmol) in a mixture of THF (5 mL) and isopropanol (5 mL). After stirring the reaction mixture for 5 min at 0 °C, stirring was continued for 48 h at ambient temperature. Then, the solvent was removed *in vacuo* and the residue was triturated with cold water and cold acetonitrile. The desired product was dried on high vacuum to give (*R*)-**13b** as colorless solid (100 mg, 0.17 mmol, 83%). m.p.: 185 °C; $[\alpha]_D^{20}$ = +37.5 (1.2, dimethyl sulfoxide); HPLC (method 2): t_R = 13.7 min, purity 99.4%.

Spectroscopic data of (*S*)-**13b** *and* (*R*)-**13b**:

^{1}H NMR (DMSO-d_6): δ [ppm] = 2.31 – 2.39 (m, 4H, N(CH_2CH$_2$)$_2$O), 3.45 – 3.52 (m, 1H, OCHCH_2NH), 3.48 (s, 2H, NCH_2Ar), 3.54 – 3.62 (m, 5H, N(CH$_2$CH_2)$_2$O, OCHCH_2NH (1H)), 4.02 (dd, *J* = 7.9/4.6 Hz, 1H, OC*H*CH$_2$NH), 4.42 (d, *J* = 12.6 Hz, 1H, OCH_2Ar), 4.63 (d, *J* = 12.6 Hz, 1H, OCH_2Ar), 7.31 – 7.36 (m, 2H, 3'''-H$_{4\text{-(morpholinomethyl)phenyl}}$, 5'''-H$_{4\text{-(morpholinomethyl)phenyl}}$), 7.36 – 7.43 (m, 5H, 4'-H$_{[1,1'\text{-biphenyl]-4-yl}}$, 2''-H$_{4\text{-\{[4-}}$

(morpholinomethyl)phenyl]ethynyl}phenyl, 3''-$H_{4\text{-}\{[4\text{-}(morpholinomethyl)phenyl]ethynyl\}phenyl}$, 5''-$H_{4\text{-}\{[4\text{-}(morpholinomethyl)phenyl]ethynyl\}phenyl}$, 6''-$H_{4\text{-}\{[4\text{-}(morpholinomethyl)phenyl]ethynyl\}phenyl}$), 7.43 – 7.48 (m, 4H, 3'-$H_{[1,1'\text{-}biphenyl]\text{-}4\text{-}yl}$, 5'-$H_{[1,1'\text{-}biphenyl]\text{-}4\text{-}yl}$, 2'''-$H_{4\text{-}(morpholinomethyl)phenyl}$, 6'''-$H_{4\text{-}(morpholinomethyl)phenyl}$), 7.70 – 7.74 (m, 2H, 2'-$H_{[1,1'\text{-}biphenyl]\text{-}4\text{-}yl}$, 6'-$H_{[1,1'\text{-}biphenyl]\text{-}4\text{-}yl}$), 7.75 – 7.79 (m, 2H, 2-$H_{[1,1'\text{-}biphenyl]\text{-}4\text{-}yl}$, 6-$H_{[1,1'\text{-}biphenyl]\text{-}4\text{-}yl}$), 7.88 – 7.92 (m, 2H, 3-$H_{[1,1'\text{-}biphenyl]\text{-}4\text{-}yl}$, 5-$H_{[1,1'\text{-}biphenyl]\text{-}4\text{-}yl}$), 8.64 (t, *J* = 5.7 Hz, 1H, CON*H*CH_2), 8.99 (s br, 1H, CON*H*OH), 10.92 (s br, 1H, CONHO*H*); ^{13}C NMR (DMSO-d_6): δ [ppm] = 41.8 (1C, OCH*C*H_2NH), 53.1 (2C, N(*C*$H_2$$CH_2$)$_2$O), 62.0 (1C, Ar*C*$H_2$N), 66.2 (2C, N($CH_2$*C*$H_2$)$_2$O), 70.7 (1C, O*C*$H_2$Ar), 76.7 (1C, O*C*H$CH_2$NH), 89.0 (1C, *C*≡C), 89.2 (1C, *C*≡C), 120.8 (1C, C-1'''$_{4\text{-}(morpholinomethyl)phenyl}$), 121.4 (1C, C-4''$_{4\text{-}\{[4\text{-}(morpholinomethyl)phenyl]ethynyl\}phenyl}$), 126.5 (2C, C-2$_{[1,1'\text{-}biphenyl]\text{-}4\text{-}yl}$, C-6$_{[1,1'\text{-}biphenyl]\text{-}4\text{-}yl}$), 126.9 (2C, C-2'$_{[1,1'\text{-}biphenyl]\text{-}4\text{-}yl}$, C-6'$_{[1,1'\text{-}biphenyl]\text{-}4\text{-}yl}$), 127.8 (2C, $C_{arom.}$), 127.9 (2C, $C_{arom.}$), 128.0 (1C, C-4'$_{[1,1'\text{-}biphenyl]\text{-}4\text{-}yl}$), 129.0 (2C, C-3'$_{[1,1'\text{-}biphenyl]\text{-}4\text{-}yl}$, C-5'$_{[1,1'\text{-}biphenyl]\text{-}4\text{-}yl}$), 129.1 (2C, C-3'''$_{4\text{-}(morpholinomethyl)phenyl}$, C-5'''$_{4\text{-}(morpholinomethyl)phenyl}$), 131.1 (2C, $C_{arom.}$), 131.2 (2C, $C_{arom.}$), 133.1 (1C, C-4$_{[1,1'\text{-}biphenyl]\text{-}4\text{-}yl}$), 138.6 (1C, C-1''$_{4\text{-}\{[4\text{-}(morpholinomethyl)phenyl]ethynyl\}phenyl}$), 138.8 (1C, C-4'''$_{4\text{-}(morpholinomethyl)phenyl}$), 139.2 (1C, C-1'$_{[1,1'\text{-}biphenyl]\text{-}4\text{-}yl}$), 142.7 (1C, C-1$_{[1,1'\text{-}biphenyl]\text{-}4\text{-}yl}$), 166.1 (1C, *C*ONH$CH_2$), 166.2 (1C, *C*ONHOH); IR (neat): $\tilde{\nu}$ [cm^{-1}] = 3210, 2957, 2815, 1655, 1620, 1533, 1513, 1485, 1330, 1302, 1112, 1095, 1007, 860, 845, 749, 514; HRMS (*m*/*z*): [M+H]$^+$ calcd for $C_{36}H_{36}N_3O_5$: 590.2649, found: 590.2667.

(*S*)-*N*-{3-(Hydroxyamino)-2-[(4-{[4-(morpholinomethyl)phenyl]ethynyl}benzyl)oxy]-3-oxopropyl}-[1,1'-biphenyl]-3-carboxamide ((*S*)-13c)

Under ice-cooling, an aqueous solution of hydroxylamine (50 wt%, 3 mL) was added to a solution of (*S*)-**39c** (100 mg, 0.18 mmol) in a mixture of THF (4 mL) and

isopropanol (4 mL). After stirring the reaction mixture for 5 min at 0 °C, stirring was continued for 48 h at ambient temperature. Then, the solvent was removed *in vacuo* and the residue was triturated with cold water and cold acetonitrile. The desired product was dried on high vacuum to give (*S*)-**13c** as colorless solid (73 mg, 0.12 mmol, 70%). m.p.: 134 °C; $[\alpha]_D^{20}$ = -56.0 (1.0, dimethyl sulfoxide); HPLC (method 2): t_R = 13.7 min, purity 95.5%.

(*R*)-*N*-{3-(Hydroxyamino)-2-[(4-{[4-(morpholinomethyl)phenyl]ethynyl}benzyl)oxy]-3-oxopropyl}-[1,1'-biphenyl]-3-carboxamide ((*R*)-13c)

Under ice-cooling, an aqueous solution of hydroxylamine (50 wt%, 3 mL) was added to a solution of (*R*)-**39c** (110 mg, 0.19 mmol) in a mixture of THF (5 mL) and isopropanol (5 mL). After stirring the reaction mixture for 5 min at 0 °C, stirring was continued for 48 h at ambient temperature. Then, the solvent was removed *in vacuo* and the residue was triturated with cold water and cold acetonitrile. The desired product was dried on high vacuum to give (*R*)-**13c** as colorless solid (42 mg, 0.071 mmol, 38%). m.p.: 134 °C; $[\alpha]_D^{20}$ = +56.7 (1.2, dimethyl sulfoxide); HPLC (method 2): t_R = 13.7 min, purity 97.0%.

Spectroscopic data of (*S*)-**13c** *and* (*R*)-**13c**:

^{1}H NMR (DMSO-d_6): δ [ppm] = 2.31 – 2.40 (m, 4H, N(CH_2CH$_2$)$_2$O), 3.46 – 3.53 (m, 1H, OCHCH_2NH), 3.49 (s, 2H, NCH_2Ar), 3.55 – 3.63 (m, 5H, N(CH$_2$CH_2)$_2$O, OCHCH_2NH (1H)), 4.02 (dd, *J* = 7.9/4.7 Hz, 1H, OC*H*CH$_2$NH), 4.43 (d, *J* = 12.7 Hz, 1H, OCH_2Ar), 4.63 (d, *J* = 12.7 Hz, 1H, OCH_2Ar), 7.33 – 7.42 (m, 7H, 4'-H$_{[1,1'\text{-biphenyl}]\text{-3-yl}}$, 2''-H$_{4\text{-}\{[4\text{-}}$

$_{\text{(morpholinomethyl)phenyl]ethynyl\}phenyl}}$, 3''-H$_{\text{4-\{[4-(morpholinomethyl)phenyl]ethynyl\}phenyl}}$, 5''-H$_{\text{4-\{[4-(morpholinomethyl)phenyl]ethynyl\}phenyl}}$, 6''-H$_{\text{4-\{[4-(morpholinomethyl)phenyl]ethynyl\}phenyl}}$, 3'''-H$_{\text{4-(morpholinomethyl)phenyl}}$, 5'''-H$_{\text{4-(morpholinomethyl)phenyl}}$), 7.45 – 7.52 (m, 4H, 3'-H$_{\text{[1,1'-biphenyl]-3-yl}}$, 5'-H$_{\text{[1,1'-biphenyl]-3-yl}}$, 2'''-H$_{\text{4-(morpholinomethyl)phenyl}}$, 6'''-H$_{\text{4-(morpholinomethyl)phenyl}}$), 7.57 (t, *J* = 7.7 Hz,, 1H, 5-H$_{\text{[1,1'-biphenyl]-3-yl}}$), 7.71 – 7.75 (m, 2H, 2'-H$_{\text{[1,1'-biphenyl]-3-yl}}$, 6'-H$_{\text{[1,1'-biphenyl]-3-yl}}$), 7.81 – 7.85 (m, 2H, 4-H$_{\text{[1,1'-biphenyl]-3-yl}}$, 6-H$_{\text{[1,1'-biphenyl]-3-yl}}$), 8.11 – 8.14 (m, 1H, 2-H$_{\text{[1,1'-biphenyl]-3-yl}}$), 8.79 (t, *J* = 5.7 Hz, 1H, CON*H*CH_2), 8.99 (s br, 1H, CON*H*OH), 10.92 (s br, 1H, CONHO*H*); ^{13}C NMR (DMSO-d_6): δ [ppm] = 41.8 (1C, OCH*C*H_2NH), 53.2 (2C, N(*C*$H_2CH_2)_2$O), 62.0 (1C, Ar*C*H_2N), 66.2 (2C, N(CH_2*C*$H_2)_2$O), 70.6 (1C, O*C*H_2Ar), 76.7 (1C, O*C*HCH_2NH), 89.0 (1C, *C*≡C), 89.2 (1C, *C*≡C), 120.8 (1C, C-1'''$_{\text{4-(morpholinomethyl)phenyl}}$), 121.4 (1C, C-4''$_{\text{4-\{[4-(morpholinomethyl)phenyl]ethynyl\}phenyl}}$), 125.4 (1C, C-2$_{\text{[1,1'-biphenyl]-3-yl}}$), 126.5 (1C, C-4$_{\text{[1,1'-biphenyl]-3-yl}}$), 126.8 (2C, C-2'$_{\text{[1,1'-biphenyl]-3-yl}}$, C-6'$_{\text{[1,1'-biphenyl]-3-yl}}$), 127.7 (2C, C-2''$_{\text{4-\{[4-(morpholinomethyl)phenyl]ethynyl\}phenyl}}$, C-6''$_{\text{4-\{[4-(morpholinomethyl)phenyl]ethynyl\}phenyl}}$), 127.8 (1C, C-4'$_{\text{[1,1'-biphenyl]-3-yl}}$), 128.97 (2C, C-3'$_{\text{[1,1'-biphenyl]-3-yl}}$, C-5'$_{\text{[1,1'-biphenyl]-3-yl}}$), 128.99 (1C, C-5$_{\text{[1,1'-biphenyl]-3-yl}}$), 129.2 (2C, C-3'''$_{\text{4-(morpholinomethyl)phenyl}}$, C-5'''$_{\text{4-(morpholinomethyl)phenyl}}$), 129.4 (1C, C-6$_{\text{[1,1'-biphenyl]-3-yl}}$), 131.1 (2C, $C_{arom.}$), 131.2 (2C, $C_{arom.}$), 135.0 (1C, C-3$_{\text{[1,1'-biphenyl]-3-yl}}$), 138.7 (1C, C-1''$_{\text{4-\{[4-(morpholinomethyl)phenyl]ethynyl\}phenyl}}$), 138.8 (1C, C-4'''$_{\text{4-(morpholinomethyl)phenyl}}$), 139.6 (1C, C-1'$_{\text{[1,1'-biphenyl]-3-yl}}$), 140.1 (1C, C-1$_{\text{[1,1'-biphenyl]-3-yl}}$), 166.2 (1C, *C*ONHOH), 166.4 (1C, *C*ONHCH_2); IR (neat): $\tilde{\nu}$ [cm^{-1}] = 3221, 2932, 2859, 2806, 1661, 1548, 1337, 1115, 1094, 1007, 865, 753, 695, 540, 511; HRMS (*m/z*): $[M+H]^+$ calcd for $C_{36}H_{36}N_3O_5$: 590.2649, found: 590.2664.

(*S*)-*N*-{3-(Hydroxyamino)-2-[(4-{[4-(morpholinomethyl)phenyl]ethynyl}benzyl)oxy]-3-oxopropyl}-4-phenoxybenzamide ((*S*)-13d)

Under ice-cooling, an aqueous solution of hydroxylamine (50 wt%, 4 mL) was added to a solution of (*S*)-**39d** (180 mg, 0.29 mmol) in a mixture of THF (5 mL) and isopropanol (5 mL). After stirring the reaction mixture for 5 min at 0 °C, stirring was continued for 36 h at ambient temperature. Then, the solvent was removed *in vacuo* and the residue was purified by automatic flash column chromatography using a Biotage® Isolera™ One system (10% → 80% ACN in H_2O, Biotage® SNAP Ultra C18 12 g). Fractions containing the desired product were combined and subjected to lyophilization to give (*S*)-**13d** as colorless solid (140 mg, 0.22 mmol, 77%). m.p.: 129 °C; $[\alpha]_D^{20}$ = -25.2 (1.2, methanol); HPLC (method 2): t_R = 13.8 min, purity 99.6%.

(*R*)-*N*-{3-(Hydroxyamino)-2-[(4-{[4-(morpholinomethyl)phenyl]ethynyl}benzyl)oxy]-3-oxopropyl}-4-phenoxybenzamide ((*R*)-13d)

Under ice-cooling, an aqueous solution of hydroxylamine (50 wt%, 5 mL) was added to a solution of (*R*)-**39d** (190 mg, 0.32 mmol) in a mixture of THF (5 mL) and isopropanol (5 mL). After stirring the reaction mixture for 5 min at 0 °C, stirring was continued for 36 h at ambient temperature. Then, the solvent was removed *in vacuo* and the residue was purified by automatic flash column chromatography using a Biotage® Isolera™ One system (10% → 75% ACN in H_2O, Biotage® SNAP Ultra C18 12 g). Fractions containing the desired product were combined and subjected to

lyophilization to give (*R*)-**13d** as colorless solid (120 mg, 0.20 mmol, 63%). m.p.: 142 °C; $[\alpha]_D^{20}$ = +14.3 (4.2, methanol); HPLC (method 2): t_R = 13.8 min, purity 99.7%.

Spectroscopic data of (*S*)-**13d** *and* (*R*)-**13d**:

^{1}H NMR (DMSO-d_6): δ [ppm] = 2.31 – 2.39 (m, 4H, $N(CH_2CH_2)_2O$), 3.41 – 3.49 (m, 1H, $OCHCH_2NH$), 3.49 (s, 2H, NCH_2Ar), 3.52 – 3.60 (m, 5H, $N(CH_2CH_2)_2O$, $OCHCH_2NH$ (1H)), 3.99 (dd, *J* = 8.0/4.7 Hz, 1H, $OCHCH_2NH$), 4.41 (d, *J* = 12.7 Hz, 1H, OCH_2Ar), 4.61 (d, *J* = 12.7 Hz, 1H, OCH_2Ar), 7.01 – 7.05 (m, 2H, 3'''-H_{benzoyl}, 5'''-H_{benzoyl}), 7.05 – 7.09 (m, 2H, 2''''-H_{phenoxy}, 6''''-H_{phenoxy}), 7.16 – 7.21 (m, 1H, 4''''-H_{phenoxy}), 7.33 – 7.44 (m, 8H, 2'-$H_{\text{4-\{[4-(morpholinomethyl)phenyl]ethynyl\}phenyl}}$, 3'-$H_{\text{4-\{[4-(morpholinomethyl)phenyl]ethynyl\}phenyl}}$, 5'-$H_{\text{4-\{[4-(morpholinomethyl)phenyl]ethynyl\}phenyl}}$, 6'-$H_{\text{4-\{[4-(morpholinomethyl)phenyl]ethynyl\}phenyl}}$, 3''-$H_{\text{4-(morpholinomethyl)phenyl}}$, 5''-$H_{\text{4-(morpholinomethyl)phenyl}}$, 3''''-$H_{\text{phenoxy}}$, 5''''-$H_{\text{phenoxy}}$), 7.46 – 7.51 (m, 2H, 2''-$H_{\text{4-(morpholinomethyl)phenyl}}$, 6''-$H_{\text{4-(morpholinomethyl)phenyl}}$), 7.83 – 7.89 (m, 2H, 2'''-H_{benzoyl}, 6'''-H_{benzoyl}), 8.56 (t, *J* = 5.7 Hz, 1H, $CONHCH_2$), 8.99 (s br, 1H, CON*H*OH), 10.90 (s br, 1H, CONHO*H*); ^{13}C NMR (DMSO-d_6): δ [ppm] = 41.7 (1C, $OCHCH_2NH$), 53.1 (2C, $N(CH_2CH_2)_2O$), 62.0 (1C, $ArCH_2N$), 66.2 (2C, $N(CH_2CH_2)_2O$), 70.6 (1C, OCH_2Ar), 76.7 (1C, $OCHCH_2NH$), 89.0 (1C, *C*≡C), 89.2 (1C, *C*≡C), 117.4 (2C, C-3'''$_{\text{benzoyl}}$, C-5'''$_{\text{benzoyl}}$), 119.4 (2C, C-2''''$_{\text{phenoxy}}$, C-6''''$_{\text{phenoxy}}$), 120.8 (1C, C-1''$_{\text{4-(morpholinomethyl)phenyl}}$), 121.3 (1C, C-4'$_{\text{4-\{[4-(morpholinomethyl)phenyl]ethynyl\}phenyl}}$), 124.2 (1C, C-4''''$_{\text{phenoxy}}$), 127.8 (2C, C-2'$_{\text{4-\{[4-(morpholinomethyl)phenyl]ethynyl\}phenyl}}$, C-6'$_{\text{4-\{[4-(morpholinomethyl)phenyl]ethynyl\}phenyl}}$), 129.0 (1C, C-1'''$_{\text{benzoyl}}$), 129.1 (2C, C-3''$_{\text{4-(morpholinomethyl)phenyl}}$, C-5''$_{\text{4-(morpholinomethyl)phenyl}}$), 129.4 (2C, C-2'''$_{\text{benzoyl}}$, C-6'''$_{\text{benzoyl}}$), 130.2 (2C, C-3''''$_{\text{phenoxy}}$, C-5''''$_{\text{phenoxy}}$), 131.1 (2C, $C_{\text{arom.}}$), 131.2 (2C, $C_{\text{arom.}}$), 138.6 (1C, C-1'$_{\text{4-\{[4-(morpholinomethyl)phenyl]ethynyl\}phenyl}}$), 138.8 (1C, C-4''$_{\text{4-(morpholinomethyl)phenyl}}$), 155.7 (1C, C-1''''$_{\text{phenoxy}}$), 159.4 (1C, C-4'''$_{\text{benzoyl}}$), 165.7 (1C, $CONHCH_2$), 166.2 (1C, *C*ONHOH); IR (neat): $\tilde{\nu}$ [cm^{-1}] = 3242, 2852, 1666, 1609,

1553, 1488, 1252, 1092, 1006, 862, 842, 786, 750, 692, 525; HRMS (*m*/*z*): $[M+H]^+$ calcd for $C_{36}H_{36}N_3O_6$: 606.2599, found: 606.2552.

(*S*)-*N*-{3-(Hydroxyamino)-2-[(4-{[4-(morpholinomethyl)phenyl]ethynyl}benzyl)oxy]-3-oxopropyl}quinoline-2-carboxamide ((*S*)-13e)

Under ice-cooling, an aqueous solution of hydroxylamine (50 wt%, 5 mL) was added to a solution of (*S*)-**39e** (250 mg, 0.44 mmol) in a mixture of THF (5 mL) and isopropanol (5 mL). After stirring the reaction mixture for 5 min at 0 °C, stirring was continued for 48 h at ambient temperature. Then, the solvent was removed *in vacuo* and the residue was purified by automatic flash column chromatography using a Biotage® Isolera™ One system (10% → 75% ACN in H_2O, Biotage® SNAP Ultra C18 30 g). Fractions containing the desired product were combined and subjected to lyophilization to give (*S*)-**13e** as colorless solid (170 mg, 0.30 mmol, 68%). m.p.: 104 °C; $[\alpha]_D^{20}$ = +13.3 (0.9, methanol); HPLC (method 2): t_R = 13.3 min, purity 100%.

(*R*)-*N*-{3-(Hydroxyamino)-2-[(4-{[4-(morpholinomethyl)phenyl]ethynyl}benzyl)oxy]-3-oxopropyl}quinoline-2-carboxamide ((*R*)-13e)

Under ice-cooling, an aqueous solution of hydroxylamine (50 wt%, 5 mL) was added to a solution of (*R*)-**39e** (220 mg, 0.39 mmol) in a mixture of THF (5 mL) and isopropanol (5 mL). After stirring the reaction mixture for 5 min at 0 °C, stirring was continued for 48 h at ambient temperature. Then, the solvent was removed *in vacuo* and the residue was purified by automatic flash column chromatography using a

Biotage® Isolera™ One system (10% → 75% ACN in H_2O, Biotage® SNAP Ultra C18 12 g). Fractions containing the desired product were combined and subjected to lyophilization to give (*R*)-**13e** as colorless solid (170 mg, 0.30 mmol, 77%). m.p.: 105 °C; $[\alpha]_D^{20}$ = -15.0 (1.2, methanol); HPLC (method 2): t_R = 13.4 min, purity 100%.

Spectroscopic data of (*S*)-**13e** *and* (*R*)-**13e**:

^{1}H NMR (DMSO-*d*$_6$): δ [ppm] = 2.31 – 2.39 (m, 4H, N(CH_2CH$_2$)$_2$O), 3.48 (s, 2H, NCH_2Ar), 3.55 – 3.60 (m, 4H, N(CH$_2$CH_2)$_2$O), 3.60 – 3.67 (m, 1H, OCHCH_2NH), 3.74 (ddd, *J* = 13.6/6.1/5.0 Hz, 1H, OCHCH_2NH), 4.10 (dd, *J* = 7.3/4.8 Hz, 1H, OC*H*CH$_2$NH), 4.47 (d, *J* = 12.6 Hz, 1H, OCH_2Ar), 4.67 (d, *J* = 12.6 Hz, 1H, OCH_2Ar), 7.33 – 7.39 (m, 4H, 3''-$H_{4\text{-(morpholinomethyl)phenyl}}$, 5''-$H_{4\text{-(morpholinomethyl)phenyl}}$, 3'-$H_{4\text{-\{[4-(morpholinomethyl)phenyl]ethynyl\}phenyl}}$, 5'-$H_{4\text{-\{[4-(morpholinomethyl)phenyl]ethynyl\}phenyl}}$), 7.39 – 7.43 (m, 2H, 2'-$H_{4\text{-\{[4-(morpholinomethyl)phenyl]ethynyl\}phenyl}}$, 6'-$H_{4\text{-\{[4-(morpholinomethyl)phenyl]ethynyl\}phenyl}}$), 7.45 – 7.49 (m, 2H, 2''-$H_{4\text{-(morpholinomethyl)phenyl}}$, 6''-$H_{4\text{-(morpholinomethyl)phenyl}}$), 7.70 – 7.75 (m, 1H, 6'''-$H_{quinoline}$), 7.86 – 7.92 (m, 1H, 7'''-$H_{quinoline}$), 8.06 – 8.10 (m, 1H, 5'''-$H_{quinoline}$), 8.11 – 8.17 (m, 2H, 3'''-$H_{quinoline}$, 8'''-$H_{quinoline}$), 8.58 (d, *J* = 8.5 Hz, 1H, 4'''-$H_{quinoline}$), 8.86 (t, *J* = 6.0 Hz, 1H, CON*H*CH$_2$), 9.03 (s, 1H, CON*H*OH), 10.94 (s, 1H, CONHO*H*); ^{13}C NMR (DMSO-*d*$_6$): δ [ppm] = 41.2 (1C, OCH*C*H$_2$NH), 53.1 (2C, N(*C*H$_2$CH$_2$)$_2$O), 62.0 (1C, Ar*C*H$_2$N), 66.2 (2C, N(CH$_2$*C*H$_2$)$_2$O), 70.7 (1C, O*C*H$_2$Ar), 76.4 (1C, O*C*HCH$_2$NH), 88.9 (1C, *C*≡C), 89.3 (1C, *C*≡C), 118.5 (1C, C-3'''$_{quinoline}$), 120.8 (1C, C-1''$_{4\text{-(morpholinomethyl)phenyl}}$), 121.4 (1C, C-4'$_{4\text{-\{[4-(morpholinomethyl)phenyl]ethynyl\}phenyl}}$), 127.9 (2C, C-2'$_{4\text{-\{[4-(morpholinomethyl)phenyl]ethynyl\}phenyl}}$, C-6'$_{4\text{-\{[4-(morpholinomethyl)phenyl]ethynyl\}phenyl}}$), 128.11 (1C, $C_{arom.}$), 128.14 (1C, $C_{arom.}$), 128.9 (1C, C-4a'''$_{quinoline}$), 129.15 (2C, C-3''$_{4\text{-(morpholinomethyl)phenyl}}$, C-5''$_{4\text{-(morpholinomethyl)phenyl}}$), 129.18 (1C, C-8'''$_{quinoline}$), 130.6 (1C, C-7'''$_{quinoline}$), 131.1 (2C, C-3'$_{4\text{-\{[4-(morpholinomethyl)phenyl]ethynyl\}phenyl}}$, C-5'$_{4\text{-\{[4-}}$

$_{(morpholinomethyl)phenyl]ethynyl\}phenyl}$), 131.2 (2C, C-2''$_{4-(morpholinomethyl)phenyl}$, C-6''$_{4-(morpholinomethyl)phenyl}$), 138.0 (1C, C-4'''$_{quinoline}$), 138.5 (1C, C-1'$_{4-\{[4-(morpholinomethyl)phenyl]ethynyl\}phenyl}$), 138.8 (1C, C-4''$_{4-(morpholinomethyl)phenyl}$), 145.9 (1C, C-8a'''$_{quinoline}$), 149.7 (1C, C-2'''$_{quinoline}$), 164.0 (1C, *C*ONHCH$_2$), 166.1 (1C, *C*ONHOH); IR (neat): $\tilde{\nu}$ [cm^{-1}] = 3210, 2858, 2812, 1660, 1500, 1112, 1005, 844, 773, 624; HRMS (*m*/*z*): [M+H]$^+$ calcd for $C_{33}H_{33}N_4O_5$: 565.2445, found: 565.2451.

(*S*)-*N*-{3-(Hydroxyamino)-2-[(4-{[4-(morpholinomethyl)phenyl]ethynyl}benzyl)oxy]-3-oxopropyl}quinoline-3-carboxamide ((*S*)-13f)

Under ice-cooling, an aqueous solution of hydroxylamine (50 wt%, 3 mL) was added to a solution of (*S*)-**39f** (95 mg, 0.17 mmol) in a mixture of THF (4 mL) and isopropanol (4 mL). After stirring the reaction mixture for 5 min at 0 °C, stirring was continued for 48 h at ambient temperature. Then, the solvent was removed *in vacuo* and the residue was purified by automatic flash column chromatography using a Biotage® Isolera™ One system (10% → 75% ACN in H_2O, Biotage® SNAP Ultra C18 30 g). Fractions containing the desired product were combined and subjected to lyophilization to give (*S*)-**13f** as colorless solid (75 mg, 0.13 mmol, 79%). m.p.: 107 °C; $[\alpha]_D^{20}$ = -19.0 (2.1, methanol); HPLC (method 2): t_R = 12.7 min, purity 100%.

(*R*)-*N*-{3-(Hydroxyamino)-2-[(4-{[4-(morpholinomethyl)phenyl]ethynyl}benzyl)oxy]-3-oxopropyl}quinoline-3-carboxamide ((*R*)-13f)

Under ice-cooling, an aqueous solution of hydroxylamine (50 wt%, 5 mL) was added to a solution of (*R*)-**39f** (210 mg, 0.38 mmol) in a mixture of THF (5 mL) and

isopropanol (5 mL). After stirring the reaction mixture for 5 min at 0 °C, stirring was continued for 24 h at ambient temperature. Then, the solvent was removed *in vacuo* and the residue was purified by automatic flash column chromatography using a Biotage® Isolera™ One system (10% → 75% ACN in H_2O, Biotage® SNAP Ultra C18 12 g). Fractions containing the desired product were combined and subjected to lyophilization to give (*R*)-**13f** as colorless solid (100 mg, 0.18 mmol, 47%). m.p.: 106 °C; $[\alpha]_D^{20}$ = +19.4 (1.7, methanol); HPLC (method 2): t_R = 12.7 min, purity 99.5%.

Spectroscopic data of (*S*)-**13f** *and* (*R*)-**13f**:

^{1}H NMR (DMSO-*d*$_6$): δ [ppm] = 2.28 – 2.42 (m, 4H, N(C*H*$_2$CH$_2$)$_2$O), 3.49 (s, 2H, NC*H*$_2$Ar), 3.51 – 3.61 (m, 5H, OCHC*H*$_2$NH (1H), N(CH$_2$C*H*$_2$)$_2$O), 3.64 (dt *J* = 13.5/5.2 Hz, 1H, OCHC*H*$_2$NH), 4.05 (dd, *J* = 7.9/4.8 Hz, 1H, OC*H*CH$_2$NH), 4.45 (d, *J* = 12.6 Hz, 1H, OC*H*$_2$Ar), 4.64 (d, *J* = 12.6 Hz, 1H, OC*H*$_2$Ar), 7.32 – 7.37 (m, 2H, 3''-H$_{4\text{-(morpholinomethyl)phenyl}}$, 5''-H$_{4\text{-(morpholinomethyl)phenyl}}$), 7.37 – 7.42 (m, 4H, 2'-H$_{4\text{-\{[4-(morpholinomethyl)phenyl]ethynyl\}phenyl}}$, 3'-H$_{4\text{-\{[4-(morpholinomethyl)phenyl]ethynyl\}phenyl}}$, 5'-H$_{4\text{-\{[4-(morpholinomethyl)phenyl]ethynyl\}phenyl}}$, 6'-H$_{4\text{-\{[4-(morpholinomethyl)phenyl]ethynyl\}phenyl}}$), 7.43 – 7.49 (m, 2H, 2''-H$_{4\text{-(morpholinomethyl)phenyl}}$, 6''-H$_{4\text{-(morpholinomethyl)phenyl}}$), 7.65 – 7.71 (m, 1H, 6'''-H$_{\text{quinoline}}$), 7.83 – 7.88 (m, 1H, 7'''-H$_{\text{quinoline}}$), 8.06 – 8.11 (m, 2H, 5'''-H$_{\text{quinoline}}$, 8'''-H$_{\text{quinoline}}$), 8.76 – 8.79 (m, 1H, 4'''-H$_{\text{quinoline}}$), 8.94 – 9.06 (m, 2H, CON*H*CH$_2$, CON*H*OH), 9.26 (d, *J* = 2.2 Hz, 1H, 2'''-H$_{\text{quinoline}}$), 10.94 (s br, 1H, CONHO*H*); ^{13}C NMR (DMSO-*d*$_6$): δ [ppm] = 41.8 (1C, OCH*C*H$_2$NH), 53.1 (2C, N(*C*H$_2$CH$_2$)$_2$O), 62.0 (1C, Ar*C*H$_2$N), 66.2 (2C, N(CH$_2$*C*H$_2$)$_2$O), 70.7 (1C, O*C*H$_2$Ar), 76.7 (1C, O*C*HCH$_2$NH), 88.9 (1C, *C*≡C), 89.2 (1C, *C*≡C), 120.8 (1C, C-1''$_{4\text{-(morpholinomethyl)phenyl}}$), 121.4 (1C, C-4'$_{4\text{-\{[4-(morpholinomethyl)phenyl]ethynyl\}phenyl}}$), 126.5 (1C, C-4a'''$_{\text{quinoline}}$), 126.9 (1C, C-3'''$_{\text{quinoline}}$), 127.4 (1C, C-6'''$_{\text{quinoline}}$), 127.9 (2C, C-2'$_{4\text{-\{[4-(morpholinomethyl)phenyl]ethynyl\}phenyl}}$, C-6'$_{4\text{-\{[4-}}$

(morpholinomethyl)phenyl]ethynyl}phenyl), 128.8 (1C, C-8'''$_{quinoline}$), 129.09 (1C, C-5'''$_{quinoline}$), 129.12 (2C, C-3''$_{4\text{-(morpholinomethyl)phenyl}}$, C-5''$_{4\text{-(morpholinomethyl)phenyl}}$), 131.1 (2C, C-3'$_{4\text{-\{[4-(morpholinomethyl)phenyl]ethynyl\}phenyl}}$, C-5'$_{4\text{-\{[4-(morpholinomethyl)phenyl]ethynyl\}phenyl}}$), 131.15 (1C, C-7'''$_{quinoline}$), 131.20 (2C, C-2''$_{4\text{-(morpholinomethyl)phenyl}}$, C-6''$_{4\text{-(morpholinomethyl)phenyl}}$), 135.5 (1C, C-4'''$_{quinoline}$), 138.6 (1C, C-1'$_{4\text{-\{[4-(morpholinomethyl)phenyl]ethynyl\}phenyl}}$), 138.8 (1C, C-4''$_{4\text{-(morpholinomethyl)phenyl}}$), 148.5 (1C, C-8a'''$_{quinoline}$), 148.9 (1C, C-2'''$_{quinoline}$), 165.1 (1C, *C*ONHCH$_2$), 166.1 (1C, *C*ONHOH); IR (neat): $\tilde{\nu}$ [cm^{-1}] = 3220, 2856, 2810, 1642, 1516, 1306, 1112, 1006, 916, 863, 788, 515, 480; HRMS (*m*/*z*): $[M+H]^+$ calcd for $C_{33}H_{33}N_4O_5$: 565.2445, found: 565.2454.

(*S*)-*N*-{3-(Hydroxyamino)-2-[(4-{[4-(morpholinomethyl)phenyl]ethynyl}benzyl)oxy]-3-oxopropyl}-1*H*-indole-2-carboxamide ((*S*)-13g)

Under ice-cooling, an aqueous solution of hydroxylamine (50 wt%, 4 mL) was added to a solution of (*S*)-**39g** (180 mg, 0.32 mmol) in a mixture of THF (5 mL) and isopropanol (5 mL). After stirring the reaction mixture for 5 min at 0 °C, stirring was continued for 48 h at ambient temperature. Then, the solvent was removed *in vacuo* and the residue was purified by automatic flash column chromatography using a Biotage® Isolera™ One system (10% → 80% ACN in H_2O, Biotage® SNAP Ultra C18 30 g). Fractions containing the desired product were combined and subjected to lyophilization to give (*S*)-**13g** as colorless solid (150 mg, 0.26 mmol, 82%). m.p.: 119 °C; $[\alpha]_D^{20}$ = -6.0 (1.5, methanol); HPLC (method 2): t_R = 13.3 min, purity 100%.

(*R*)-*N*-{3-(Hydroxyamino)-2-[(4-{[4-(morpholinomethyl)phenyl]ethynyl}benzyl)oxy]-3-oxopropyl}-1*H*-indole-2-carboxamide ((*R*)-13g)

Under ice-cooling, an aqueous solution of hydroxylamine (50 wt%, 4 mL) was added to a solution of (*R*)-**39g** (190 mg, 0.34 mmol) in a mixture of THF (5 mL) and isopropanol (5 mL). After stirring the reaction mixture for 5 min at 0 °C, stirring was continued for 36 h at ambient temperature. Then, the solvent was removed *in vacuo* and the residue was purified by automatic flash column chromatography using a Biotage® Isolera™ One system (10% → 80% ACN in H_2O, Biotage® SNAP Ultra C18 12 g). Fractions containing the desired product were combined and subjected to lyophilization to give (*R*)-**13g** as colorless solid (110 mg, 0.20 mmol, 60%). m.p.: 119 °C; $[\alpha]_D^{20}$ = +6.7 (1.8, methanol); HPLC (method 2): t_R = 13.2 min, purity 98.9%.

Spectroscopic data of (*S*)-**13g** *and* (*R*)-**13g**:

^{1}H NMR (DMSO-*d*$_6$): δ [ppm] = 2.30 – 2.40 (m, 4H, N(C*H*$_2$CH$_2$)$_2$O), 3.48 (s, 2H, NC*H*$_2$Ar), 3.48 – 3.55 (m, 1H, OCHC*H*$_2$NH), 3.55 – 3.66 (m, 5H, OCHC*H*$_2$NH (1H), N(CH$_2$C*H*$_2$)$_2$O), 4.01 (dd, *J* = 7.9/4.7 Hz, 1H, OC*H*CH$_2$NH), 4.46 (d, *J* = 12.6 Hz, 1H, OC*H*$_2$Ar), 4.62 (d, *J* = 12.6 Hz, 1H, OC*H*$_2$Ar), 7.00 – 7.06 (m, 1H, 5'''-H_{indole}), 7.13 – 7.20 (m, 2H, 3'''-H_{indole}, 6'''-H_{indole}), 7.32 – 7.37 (m, 2H, 3''-$H_{4\text{-(morpholinomethyl)phenyl}}$, 5''-$H_{4\text{-(morpholinomethyl)phenyl}}$), 7.37 – 7.45 (m, 5H, 2'-$H_{4\text{-\{[4-(morpholinomethyl)phenyl]ethynyl\}phenyl}}$, 3'-$H_{4\text{-\{[4-(morpholinomethyl)phenyl]ethynyl\}phenyl}}$, 5'-$H_{4\text{-\{[4-(morpholinomethyl)phenyl]ethynyl\}phenyl}}$, 6'-$H_{4\text{-\{[4-(morpholinomethyl)phenyl]ethynyl\}phenyl}}$, 7'''-$H_{indole}$), 7.45 – 7.50 (m, 2H, 2''-$H_{4\text{-(morpholinomethyl)phenyl}}$, 6''-$H_{4\text{-(morpholinomethyl)phenyl}}$), 7.60 – 7.66 (m, 1H, 4'''-H_{indole}), 8.62 (t, *J* = 5.8 Hz, 1H, CON*H*CH$_2$), 8.99 (s br, 1H, CON*H*OH), 10.92 (s br, 1H, CONHO*H*), 11.53 – 11.57 (m, 1H, 1'''-H_{indole}); ^{13}C NMR (DMSO-*d*$_6$): δ [ppm] = 41.3 (1C, OCH*C*H$_2$NH), 53.1 (2C,

N(CH_2CH_2)$_2$O), 62.0 (1C, ArCH_2N), 66.2 (2C, N(CH_2CH_2)$_2$O), 70.6 (1C, OCH_2Ar), 76.9 (1C, O*C*HCH_2NH), 89.0 (1C, *C*≡C), 89.2 (1C, *C*≡C), 102.8 (1C, C-3'''$_{indole}$), 112.3 (1C, C-7'''$_{indole}$), 119.7 (1C, C-5'''$_{indole}$), 120.8 (1C, C-1''$_{4\text{-(morpholinomethyl)phenyl}}$), 121.3 (1C, C-4'$_{4\text{-\{[4-(morpholinomethyl)phenyl]ethynyl\}phenyl}}$), 121.5 (1C, C-4'''$_{indole}$), 123.3 (1C, C-6'''$_{indole}$), 127.1 (1C, C-3a'''$_{indole}$), 127.7 (2C, C-2'$_{4\text{-\{[4-(morpholinomethyl)phenyl]ethynyl\}phenyl}}$, C-6'$_{4\text{-\{[4-(morpholinomethyl)phenyl]ethynyl\}phenyl}}$), 129.1 (2C, C-3''$_{4\text{-(morpholinomethyl)phenyl}}$, C-5''$_{4\text{-(morpholinomethyl)phenyl}}$), 131.1 (2C, C-3'$_{4\text{-\{[4-(morpholinomethyl)phenyl]ethynyl\}phenyl}}$, C-5'$_{4\text{-\{[4-(morpholinomethyl)phenyl]ethynyl\}phenyl}}$), 131.2 (2C, C-2''$_{4\text{-(morpholinomethyl)phenyl}}$, C-6''$_{4\text{-(morpholinomethyl)phenyl}}$), 131.5 (1C, C-2'''$_{indole}$), 136.4 (1C, C-7a'''$_{indole}$), 138.6 (1C, C-1'$_{4\text{-\{[4-(morpholinomethyl)phenyl]ethynyl\}phenyl}}$), 138.8 (1C, C-4''$_{4\text{-(morpholinomethyl)phenyl}}$), 161.3 (1C, *C*ONH$CH_2$), 166.1 (1C, *C*ONHOH); IR (neat): $\tilde{\nu}$ [cm^{-1}] = 3254, 2918, 2860, 2810, 1638, 1547, 1416, 1309, 1261, 1110, 1004, 863, 818, 746, 540, 432; HRMS (*m*/*z*): [M+H]$^+$ calcd for $C_{32}H_{33}N_4O_5$: 553.2445, found: 553.2441.

(*S*)-*N*-{3-(Hydroxyamino)-2-[(4-{[4-(morpholinomethyl)phenyl]ethynyl}benzyl)oxy]-3-oxopropyl}-1*H*-indole-3-carboxamide ((*S*)-13h)

Under ice-cooling, an aqueous solution of hydroxylamine (50 wt%, 4 mL) was added to a solution of (*S*)-**39h** (150 mg, 0.27 mmol) in a mixture of THF (5 mL) and isopropanol (5 mL). After stirring the reaction mixture for 5 min at 0 °C, stirring was continued for 36 h at ambient temperature. Then, the solvent was removed *in vacuo* and the residue was purified by automatic flash column chromatography using a Biotage® Isolera™ One system (10% → 80% ACN in H_2O, Biotage® SNAP Ultra C18 30 g). Fractions containing the desired product were combined and subjected to

lyophilization to give (*S*)-**13h** as colorless solid (100 mg, 0.18 mmol, 67%). m.p.: 159 °C; $[\alpha]_D^{20}$ = -52.4 (1.6, methanol); HPLC (method 2): t_R = 12.9 min, purity 99.9%.

(*R*)-*N*-{3-(Hydroxyamino)-2-[(4-{[4-(morpholinomethyl)phenyl]ethynyl}benzyl)oxy]-3-oxopropyl}-1*H*-indole-3-carboxamide ((*R*)-13h)

Under ice-cooling, an aqueous solution of hydroxylamine (50 wt%, 5 mL) was added to a solution of (*R*)-**39h** (170 mg, 0.31 mmol) in a mixture of THF (5 mL) and isopropanol (5 mL). After stirring the reaction mixture for 5 min at 0 °C, stirring was continued for 36 h at ambient temperature. Then, the solvent was removed *in vacuo* and the residue was purified by automatic flash column chromatography using a Biotage® Isolera™ One system (10% → 75% ACN in H_2O, Biotage® SNAP Ultra C18 12 g). Fractions containing the desired product were combined and subjected to lyophilization to give (*R*)-**13h** as colorless solid (90 mg, 0.16 mmol, 53%). m.p.: 159 °C; $[\alpha]_D^{20}$ = +54.3 (1.8, methanol); HPLC (method 2): t_R = 13.0 min, purity 99.6%.

Spectroscopic data of (*S*)-**13h** *and* (*R*)-**13h**:

^1H NMR (DMSO-d_6): δ [ppm] = 2.31 – 2.39 (m, 4H, $N(CH_2CH_2)_2O$), 3.41 – 3.51 (m, 3H, $OCHCH_2NH$ (1H), NCH_2Ar), 3.54 – 3.65 (m, 5H, $OCHCH_2NH$ (1H), $N(CH_2CH_2)_2O$), 4.02 (dd, *J* = 7.6/4.9 Hz, 1H, $OCHCH_2NH$), 4.46 (d, *J* = 12.6 Hz, 1H, OCH_2Ar), 4.61 (d, *J* = 12.6 Hz, 1H, OCH_2Ar), 7.07 – 7.12 (m, 1H, 5'''-H_{indole}), 7.12 – 7.17 (m, 1H, 6'''-H_{indole}), 7.33 – 7.38 (m, 2H, 3''-$H_{4\text{-(morpholinomethyl)phenyl}}$, 5''-$H_{4\text{-(morpholinomethyl)phenyl}}$), 7.38 – 7.45 (m, 5H, 2'-$H_{4\text{-\{[4-(morpholinomethyl)phenyl]ethynyl\}phenyl}}$, 3'-$H_{4\text{-\{[4-(morpholinomethyl)phenyl]ethynyl\}phenyl}}$, 5'-$H_{4\text{-\{[4-(morpholinomethyl)phenyl]ethynyl\}phenyl}}$, 6'-$H_{4\text{-\{[4-(morpholinomethyl)phenyl]ethynyl\}phenyl}}$, 7'''-$H_{indole}$),

7.46 – 7.52 (m, 2H, 2''-$H_{4\text{-(morpholinomethyl)phenyl}}$, 6''-$H_{4\text{-(morpholinomethyl)phenyl}}$), 8.03 (t, *J* = 5.9 Hz, 1H, CON*H*CH_2), 8.06 (d, *J* = 2.9 Hz, 1H, 2'''-H_{indole}), 8.11 – 8.16 (m, 1H, 4'''-H_{indole}), 8.99 (s br, 1H, CON*H*OH), 10.90 (s br, 1H, CONHO*H*), 11.50 – 11.59 (m, 1H, 1'''-H_{indole}); ^{13}C NMR (DMSO-*d*6): δ [ppm] = 41.0 (1C, OCH*C*H_2NH), 53.1 (2C, N(*C*$H_2CH_2)_2$O), 62.0 (1C, Ar*C*H_2N), 66.2 (2C, N(CH_2*C*$H_2)_2$O), 70.6 (1C, O*C*H_2Ar), 77.1 (1C, O*C*HCH_2NH), 89.0 (1C, *C*≡C), 89.2 (1C, *C*≡C), 110.4 (1C, C-3'''$_{indole}$), 111.8 (1C, C-7'''$_{indole}$), 120.3 (1C, C-5'''$_{indole}$), 120.8 (1C, C-1''$_{4\text{-(morpholinomethyl)phenyl}}$), 120.9 (1C, C-4'''$_{indole}$), 121.3 (1C, C-4'$_{4\text{-\{[4-(morpholinomethyl)phenyl]ethynyl\}phenyl}}$), 121.8 (1C, C-6'''$_{indole}$), 126.1 (1C, C-3a'''$_{indole}$), 127.7 (2C, C-2'$_{4\text{-\{[4-(morpholinomethyl)phenyl]ethynyl\}phenyl}}$, C-6'$_{4\text{-\{[4-(morpholinomethyl)phenyl]ethynyl\}phenyl}}$), 128.0 (1C, C-2'''$_{indole}$), 129.1 (2C, C-3''$_{4\text{-(morpholinomethyl)phenyl}}$, C-5''$_{4\text{-(morpholinomethyl)phenyl}}$), 131.1 (2C, $C_{arom.}$), 131.2 (2C, $C_{arom.}$), 136.1 (1C, C-7a'''$_{indole}$), 138.7 (1C, $C_{arom.}$), 138.8 (1C, $C_{arom.}$), 164.8 (1C, *C*ONHCH_2), 166.4 (1C, *C*ONHOH); IR (neat): $\tilde{\nu}$ [cm^{-1}] = 3211, 2861, 2811, 1620, 1537, 1515, 1454, 1204, 1109, 1006, 862, 819, 747; HRMS (*m*/*z*): $[M+H]^+$ calcd for $C_{32}H_{33}N_4O_5$: 553.2445, found: 553.2459.

(*S*)-*N*-{3-(Hydroxyamino)-2-[(4-{[4-(morpholinomethyl)phenyl]ethynyl}benzyl)oxy]-3-oxopropyl}-1*H*-indole-4-carboxamide ((*S*)-13i)

Under ice-cooling, an aqueous solution of hydroxylamine (50 wt%, 3 mL) was added to a solution of (*S*)-**39i** (110 mg, 0.20 mmol) in a mixture of THF (4 mL) and isopropanol (4 mL). After stirring the reaction mixture for 5 min at 0 °C, stirring was continued for 36 h at ambient temperature. Then, the solvent was removed *in vacuo* and the residue was purified by automatic flash column chromatography using a Biotage® Isolera™ One system (10% → 80% ACN in H_2O, Biotage® SNAP Ultra C18 30 g). Fractions

containing the desired product were combined and subjected to lyophilization to give (*S*)-**13i** as colorless solid (69 mg, 0.12 mmol, 63%). m.p.: 107 °C; $[\alpha]_D^{20}$ = -56.8 (1.1, methanol); ^{1}H NMR (DMSO-d_6): δ [ppm] = 2.30 – 2.40 (m, 4H, N(CH_2CH$_2$)$_2$O), 3.46 – 3.67 (m, 8H, OCHCH_2NH, NCH_2Ar, N(CH$_2$CH_2)$_2$O), 4.08 (dd, *J* = 7.6/4.8 Hz, 1H, OC*H*CH$_2$NH), 4.48 (d, *J* = 12.5 Hz, 1H, OCH_2Ar), 4.63 (d, *J* = 12.5 Hz, 1H, OCH_2Ar), 6.83 – 6.88 (m, 1H, 3'''-H_{indole}), 7.11 – 7.17 (m, 1H, 6'''-H_{indole}), 7.33 – 7.38 (m, 2H, 3''-$H_{4\text{-(morpholinomethyl)phenyl}}$, 5''-$H_{4\text{-(morpholinomethyl)phenyl}}$), 7.39 – 7.47 (m, 6H, 2'-$H_{4\text{-\{[4-(morpholinomethyl)phenyl]ethynyl\}phenyl}}$, 3'-$H_{4\text{-\{[4-(morpholinomethyl)phenyl]ethynyl\}phenyl}}$, 5'-$H_{4\text{-\{[4-(morpholinomethyl)phenyl]ethynyl\}phenyl}}$, 6'-$H_{4\text{-\{[4-(morpholinomethyl)phenyl]ethynyl\}phenyl}}$, 2'''-$H_{indole}$, 5'''-$H_{indole}$), 7.48 – 7.52 (m, 2H, 2''-$H_{4\text{-(morpholinomethyl)phenyl}}$, 6''-$H_{4\text{-(morpholinomethyl)phenyl}}$), 7.52 – 7.57 (m, 1H, 7'''-H_{indole}), 8.25 (t, *J* = 5.7 Hz, 1H, CON*H*CH$_2$), 9.02 (s br, 1H, CON*H*OH), 10.93 (s br, 1H, CONHO*H*), 11.30 (s br, 1H, 1'''-H_{indole}); ^{13}C NMR (DMSO-d_6): δ [ppm] = 41.6 (1C, OCH*C*H$_2$NH), 53.2 (2C, N(*C*H$_2$CH$_2$)$_2$O), 62.0 (1C, Ar*C*H$_2$N), 66.2 (2C, N(CH$_2$*C*H$_2$)$_2$O), 70.6 (1C, O*C*H$_2$Ar), 76.8 (1C, O*C*HCH$_2$NH), 89.1 (1C, *C*≡C), 89.3 (1C, *C*≡C), 101.7 (1C, C-3'''$_{indole}$), 114.2 (1C, C-7'''$_{indole}$), 118.7 (1C, C-5'''$_{indole}$), 120.1 (1C, C-6'''$_{indole}$), 120.9 (1C, C-1''$_{4\text{-(morpholinomethyl)phenyl}}$), 121.4 (1C, C-4'$_{4\text{-\{[4-(morpholinomethyl)phenyl]ethynyl\}phenyl}}$), 125.8 (1C, C-3a'''$_{indole}$), 126.4 (1C, C-4'''$_{indole}$), 126.5 (1C, C-2'''$_{indole}$), 127.8 (2C, C-2'$_{4\text{-\{[4-(morpholinomethyl)phenyl]ethynyl\}phenyl}}$, C-6'$_{4\text{-\{[4-(morpholinomethyl)phenyl]ethynyl\}phenyl}}$), 129.2 (2C, C-3''$_{4\text{-(morpholinomethyl)phenyl}}$, C-5''$_{4\text{-(morpholinomethyl)phenyl}}$), 131.2 (2C, $C_{arom.}$), 131.3 (2C, $C_{arom.}$), 136.5 (1C, C-7a'''$_{indole}$), 138.7 (1C, C-1'$_{4\text{-\{[4-(morpholinomethyl)phenyl]ethynyl\}phenyl}}$), 138.9 (1C, C-4''$_{4\text{-(morpholinomethyl)phenyl}}$), 166.4 (1C, *C*ONHOH), 168.0 (1C, *C*ONHCH$_2$); IR (neat): $\tilde{\nu}$ [cm^{-1}] = 3240, 2862, 2815, 1639, 1606, 1518, 1344, 1292, 1194, 1110, 1004, 863, 760; HRMS (*m*/*z*): $[M+H]^+$ calcd for $C_{32}H_{33}N_4O_5$: 553.2445, found: 553.2447; HPLC (method 2): t_R = 12.9 min, purity 99.4%.

(*S*)-*N*-{3-(Hydroxyamino)-2-[(4-{[4-(morpholinomethyl)phenyl]ethynyl}benzyl)oxy]-3-oxopropyl}-1*H*-indole-5-carboxamide ((*S*)-13j)

Under ice-cooling, an aqueous solution of hydroxylamine (50 wt%, 2.5 mL) was added to a solution of (*S*)-**39j** (97 mg, 0.18 mmol) in a mixture of THF (4 mL) and isopropanol (4 mL). After stirring the reaction mixture for 5 min at 0 °C, stirring was continued for 48 h at ambient temperature. Then, the solvent was removed *in vacuo* and the residue was purified by automatic flash column chromatography using a Biotage® Isolera™ One system (10% → 80% ACN in H_2O, Biotage® SNAP Ultra C18 30 g). Fractions containing the desired product were combined and subjected to lyophilization to give (*S*)-**13j** as colorless solid (84 mg, 0.15 mmol, 86%). m.p.: 109 °C; $[\alpha]_D^{20}$ = -18.8 (1.7, methanol); HPLC (method 2): t_R = 13.0 min, purity 99.8%.

(*R*)-*N*-{3-(Hydroxyamino)-2-[(4-{[4-(morpholinomethyl)phenyl]ethynyl}benzyl)oxy]-3-oxopropyl}-1*H*-indole-5-carboxamide ((*R*)-13j)

Under ice-cooling, an aqueous solution of hydroxylamine (50 wt%, 3 mL) was added to a solution of (*R*)-**39j** (100 mg, 0.19 mmol) in a mixture of THF (5 mL) and isopropanol (5 mL). After stirring the reaction mixture for 5 min at 0 °C, stirring was continued for 48 h at ambient temperature. Then, the solvent was removed *in vacuo* and the residue was purified by automatic flash column chromatography using a Biotage® Isolera™ One system (10% → 80% ACN in H_2O, Biotage® SNAP Ultra C18 12 g). Fractions containing the desired product were combined and subjected to lyophilization to give

(R)-**13j** as colorless solid (56 mg, 0.10 mmol, 54%). m.p.: 109 °C; $[\alpha]_D^{20}$ = +19.0 (1.0, methanol); HPLC (method 2): t_R = 12.9 min, purity 100%.

Spectroscopic data of (S)-**13j** *and* (R)-**13j**:

^{1}H NMR (DMSO-d_6): δ [ppm] = 2.31 – 2.39 (m, 4H, N(CH_2CH$_2$)$_2$O), 3.45 – 3.53 (m, 3H, OCHCH_2NH (1H), NCH_2Ar), 3.53 – 3.62 (m, 5H, OCHCH_2NH (1H), N(CH$_2$CH_2)$_2$O), 4.04 (dd, J = 7.8/4.8 Hz, 1H, OCHCH$_2$NH), 4.45 (d, J = 12.6 Hz, 1H, OCH_2Ar), 4.62 (d, J = 12.6 Hz, 1H, OCH_2Ar), 6.52 – 6.55 (m, 1H, 3'''-H_{indole}), 7.33 – 7.37 (m, 2H, 3''-$H_{4\text{-(morpholinomethyl)phenyl}}$, 5''-$H_{4\text{-(morpholinomethyl)phenyl}}$), 7.37 – 7.41 (m, 2H, 2'-$H_{4\text{-\{[4-(morpholinomethyl)phenyl]ethynyl\}phenyl}}$, 6'-$H_{4\text{-\{[4-(morpholinomethyl)phenyl]ethynyl\}phenyl}}$), 7.41 – 7.44 (m, 4H, 3'-$H_{4\text{-\{[4-(morpholinomethyl)phenyl]ethynyl\}phenyl}}$, 5'-$H_{4\text{-\{[4-(morpholinomethyl)phenyl]ethynyl\}phenyl}}$, 2'''-$H_{indole}$, 7'''-$H_{indole}$), 7.46 – 7.51 (m, 2H, 2''-$H_{4\text{-(morpholinomethyl)phenyl}}$, 6''-$H_{4\text{-(morpholinomethyl)phenyl}}$), 7.60 – 7.64 (m, 1H, 6'''-H_{indole}), 8.09 – 8.13 (m, 1H, 4'''-H_{indole}), 8.40 (t, J = 5.7 Hz, 1H, CONHCH$_2$), 8.98 (s br, 1H, CONHOH), 10.83 (s br, 1H, CONHOH), 11.31 (s br, 1H, 1'''-H_{indole}); ^{13}C NMR (DMSO-d_6): δ [ppm] = 41.8 (1C, OCHCH$_2$NH), 53.2 (2C, N(CH$_2$CH$_2$)$_2$O), 62.0 (1C, ArCH$_2$N), 66.2 (2C, N(CH$_2$$CH_2$)$_2$O), 70.6 (1C, O$CH_2$Ar), 76.9 (1C, O$CHCH_2$NH), 89.0 (1C, C≡C), 89.2 (1C, C≡C), 102.1 (1C, C-3'''$_{indole}$), 110.8 (1C, C-7'''$_{indole}$), 120.0 (1C, C-4'''$_{indole}$), 120.6 (1C, C-6'''$_{indole}$), 120.8 (1C, C-1''$_{4\text{-(morpholinomethyl)phenyl}}$), 121.3 (1C, C-4'$_{4\text{-\{[4-(morpholinomethyl)phenyl]ethynyl\}phenyl}}$), 125.4 (1C, C-5'''$_{indole}$), 126.6 (1C, C-2'''$_{indole}$), 127.0 (1C, C-3a'''$_{indole}$), 127.7 (2C, C-2'$_{4\text{-\{[4-(morpholinomethyl)phenyl]ethynyl\}phenyl}}$, C-6'$_{4\text{-\{[4-(morpholinomethyl)phenyl]ethynyl\}phenyl}}$), 129.2 (2C, C-3''$_{4\text{-(morpholinomethyl)phenyl}}$, C-5''$_{4\text{-(morpholinomethyl)phenyl}}$), 131.1 (2C, C-3'$_{4\text{-\{[4-(morpholinomethyl)phenyl]ethynyl\}phenyl}}$, C-5'$_{4\text{-\{[4-(morpholinomethyl)phenyl]ethynyl\}phenyl}}$), 131.2 (2C, C-2''$_{4\text{-(morpholinomethyl)phenyl}}$, C-6''$_{4\text{-(morpholinomethyl)phenyl}}$), 137.4 (1C, C-7a'''$_{indole}$), 138.75 (1C, C-1'$_{4\text{-\{[4-(morpholinomethyl)phenyl]ethynyl\}phenyl}}$), 138.82 (1C, C-4''$_{4\text{-(morpholinomethyl)phenyl}}$), 166.3 (1C,

*C*ONHOH), 167.6 (1C, *C*ONHCH$_2$); IR (neat): $\tilde{\nu}$ [cm^{-1}] = 3243, 2859, 2815, 1636, 1520, 1350, 1306, 1110, 1005, 863, 819, 754, 727, 514, 424; HRMS (*m*/*z*): [M+H]$^+$ calcd for $C_{32}H_{33}N_4O_5$: 553.2445, found: 553.2396.

(*S*)-*N*-{3-(Hydroxyamino)-2-[(4-{[4-(morpholinomethyl)phenyl]ethynyl}benzyl)oxy]-3-oxopropyl}-1*H*-pyrrole-2-carboxamide ((*S*)-13k)

Under ice-cooling, an aqueous solution of hydroxylamine (50 wt%, 2 mL) was added to a solution of (*S*)-**39k** (65 mg, 0.13 mmol) in a mixture of THF (3 mL) and isopropanol (3 mL). After stirring the reaction mixture for 5 min at 0 °C, stirring was continued for 48 h at ambient temperature. Then, the solvent was removed *in vacuo* and the residue was purified by automatic flash column chromatography using a Biotage® Isolera™ One system (10% → 100% ACN in H_2O, Biotage® SNAP Ultra C18 30 g). Fractions containing the desired product were combined and subjected to lyophilization to give (*S*)-**13k** as colorless solid (49 mg, 0.097 mmol, 75%). m.p.: 97 °C; $[\alpha]_D^{20}$ = -55.2 (1.3, methanol); ^{1}H NMR (DMSO-*d$_6$*): δ [ppm] = 2.30 – 2.39 (m, 4H, N(C*H$_2$*CH$_2$)$_2$O), 3.37 – 3.46 (m, 1H, OCHC*H$_2$*NH), 3.46 – 3.62 (m, 7H, OCHC*H$_2$*NH (1H), NC*H$_2$*Ar, N(CH$_2$C*H$_2$*)$_2$O), 3.94 (dd, *J* = 7.7/4.8 Hz, 1H, OC*H*CH$_2$NH), 4.43 (d, *J* = 12.6 Hz, 1H, OC*H$_2$*Ar), 4.60 (d, *J* = 12.6 Hz, 1H, OC*H$_2$*Ar), 6.05 – 6.12 (m, 1H, 4'''-H$_{pyrrole}$), 6.78 – 6.83 (m, 1H, 3'''-H$_{pyrrole}$), 6.83 – 6.88 (m, 1H, 5'''-H$_{pyrrole}$), 7.33 – 7.41 (m, 4H, 2'-H$_{4\text{-}\{[4\text{-}(morpholinomethyl)phenyl]ethynyl\}phenyl}$, 6'-H$_{4\text{-}\{[4\text{-}(morpholinomethyl)phenyl]ethynyl\}phenyl}$, 3''-H$_{4\text{-}(morpholinomethyl)phenyl}$, 5''-H$_{4\text{-}(morpholinomethyl)phenyl}$), 7.41 – 7.45 (m, 2H, 3'-H$_{4\text{-}\{[4\text{-}(morpholinomethyl)phenyl]ethynyl\}phenyl}$, 5'-H$_{4\text{-}\{[4\text{-}(morpholinomethyl)phenyl]ethynyl\}phenyl}$), 7.47 – 7.53 (m, 4H, 2''-H$_{4\text{-}(morpholinomethyl)phenyl}$, 6''-H$_{4\text{-}(morpholinomethyl)phenyl}$), 8.14 (t, *J* = 5.9 Hz, 1H, CON*H*CH$_2$), 8.98 (s br, 1H, CON*H*OH), 10.82 (s br, 1H, CONHO*H*), 11.39 – 11.46 (m, 1H, 1'''-

$H_{pyrrole}$); ^{13}C NMR (DMSO-d_6): δ [ppm] = 41.0 (1C, OCHCH_2NH), 53.2 (2C, N(CH_2CH_2)$_2$O), 62.0 (1C, ArCH_2N), 66.2 (2C, N(CH_2CH_2)$_2$O), 70.6 (1C, OCH_2Ar), 77.1 (1C, O*C*HCH_2NH), 89.1 (1C, *C*≡C), 89.2 (1C, *C*≡C), 108.5 (1C, C-4'''$_{pyrrole}$), 110.3 (1C, C-3'''$_{pyrrole}$), 120.8 (1C, C-1''$_{4\text{-(morpholinomethyl)phenyl}}$), 121.3 (1C, C-4'$_{4\text{-\{[4-(morpholinomethyl)phenyl]ethynyl\}phenyl}}$), 121.4 (1C, C-5'''$_{pyrrole}$), 126.1 (1C, C-2'''$_{pyrrole}$), 127.7 (2C, C-2'$_{4\text{-\{[4-(morpholinomethyl)phenyl]ethynyl\}phenyl}}$, C-6'$_{4\text{-\{[4-(morpholinomethyl)phenyl]ethynyl\}phenyl}}$), 129.2 (2C, C-3''$_{4\text{-(morpholinomethyl)phenyl}}$, C-5''$_{4\text{-(morpholinomethyl)phenyl}}$), 131.1 (2C, $C_{arom.}$), 131.2 (2C, $C_{arom.}$), 138.7 (1C, C-1'$_{4\text{-\{[4-(morpholinomethyl)phenyl]ethynyl\}phenyl}}$), 138.8 (1C, C-4''$_{4\text{-(morpholinomethyl)phenyl}}$), 160.8 (1C, *C*ONH$CH_2$), 166.2 (1C, *C*ONHOH); IR (neat): $\tilde{\nu}$ [cm^{-1}] = 3236, 2859, 2812, 1629, 1560, 1516, 1409, 1329, 1204, 1111, 1005, 863, 820, 742, 540, 515; HRMS (*m*/*z*): $[M+H]^+$ calcd for $C_{28}H_{31}N_4O_5$: 503.2289, found: 503.2240; HPLC (method 2): t_R = 12.8 min, purity 99.8%.

(*S*)-*N*-{3-(Hydroxyamino)-2-[(4-{[4-(morpholinomethyl)phenyl]ethynyl}benzyl)oxy]-3-oxopropyl}-1*H*-pyrazole-3-carboxamide ((*S*)-13l)

Under ice-cooling, an aqueous solution of hydroxylamine (50 wt%, 2 mL) was added to a solution of (*S*)-**39l** (64 mg, 0.13 mmol) in a mixture of THF (4 mL) and isopropanol (4 mL). After stirring the reaction mixture for 5 min at 0 °C, stirring was continued for 48 h at ambient temperature. Then, the solvent was removed *in vacuo* and the residue was purified by automatic flash column chromatography using a Biotage® Isolera™ One system (10% → 100% ACN in H_2O, Biotage® SNAP Ultra C18 30 g). Fractions containing the desired product were combined and subjected to lyophilization to give (*S*)-**13l** as colorless solid (31 mg, 0.062 mmol, 48%). m.p.: 131 °C; $[\alpha]_D^{20}$ = -53.3 (1.1, methanol); ^1H NMR (DMSO-d_6): δ [ppm] = 2.31 – 2.39 (m, 4H, N(CH_2CH_2)$_2$O), 3.44 –

3.53 (m, 3H, NCH_2Ar, OCHCH_2NH (1H)), 3.53 – 3.62 (m, 5H, OCHCH_2NH (1H), N(CH_2CH_2)$_2$O), 3.96 – 4.04 (m, 1H, OC*H*CH_2NH), 4.45 (d, *J* = 12.6 Hz, 1H, OCH_2Ar), 4.61 (d, *J* = 12.6 Hz, 1H, OCH_2Ar), 6.64 (s br, 1H, 4'''-$H_{pyrazole}$), 7.32 – 7.41 (m, 4H, 2'-$H_{4\text{-}\{[4\text{-}(morpholinomethyl)phenyl]ethynyl\}phenyl}$, 6'-$H_{4\text{-}\{[4\text{-}(morpholinomethyl)phenyl]ethynyl\}phenyl}$, 3''-$H_{4\text{-}(morpholinomethyl)phenyl}$, 5''-$H_{4\text{-}(morpholinomethyl)phenyl}$), 7.41 – 7.47 (m, 2H, 3'-$H_{4\text{-}\{[4\text{-}(morpholinomethyl)phenyl]ethynyl\}phenyl}$, 5'-$H_{4\text{-}\{[4\text{-}(morpholinomethyl)phenyl]ethynyl\}phenyl}$), 7.47 – 7.53 (m, 2H, 2''-$H_{4\text{-}(morpholinomethyl)phenyl}$, 6''-$H_{4\text{-}(morpholinomethyl)phenyl}$), 7.82 (s br, 1H, 5'''-$H_{pyrazole}$), 7.98 (s br, 1H, CON*H*CH_2), 8.98 (s br, 1H, CON*H*OH), 10.86 (s br, 1H, CONHO*H*), 13.24 (s br, 1H, 1'''-$H_{pyrazole}$); ^{13}C NMR (DMSO-d_6): δ [ppm] = 40.2 (1C, OCH*C*H_2NH), 53.1 (2C, N(*C*$H_2$$CH_2$)$_2$O), 62.0 (1C, Ar*C*$H_2$N), 66.2 (2C, N($CH_2$*C*$H_2$)$_2$O), 70.5 (1C, O*C*$H_2$Ar), 76.6 (1C, O*C*H$CH_2$NH), 89.0 (1C, *C*≡C), 89.2 (1C, *C*≡C), 105.0 (1C, C-4'''$_{pyrazole}$), 120.8 (1C, C-1''$_{4\text{-}(morpholinomethyl)phenyl}$), 121.3 (1C, C-4'$_{4\text{-}\{[4\text{-}(morpholinomethyl)phenyl]ethynyl\}phenyl}$), 127.7 (2C, C-2'$_{4\text{-}\{[4\text{-}(morpholinomethyl)phenyl]ethynyl\}phenyl}$, C-6'$_{4\text{-}\{[4\text{-}(morpholinomethyl)phenyl]ethynyl\}phenyl}$), 129.2 (2C, C-3''$_{4\text{-}(morpholinomethyl)phenyl}$, C-5''$_{4\text{-}(morpholinomethyl)phenyl}$), 131.1 (2C, C-3'$_{4\text{-}\{[4\text{-}(morpholinomethyl)phenyl]ethynyl\}phenyl}$, C-5'$_{4\text{-}\{[4\text{-}(morpholinomethyl)phenyl]ethynyl\}phenyl}$), 131.2 (2C, C-2''$_{4\text{-}(morpholinomethyl)phenyl}$, C-6''$_{4\text{-}(morpholinomethyl)phenyl}$), 138.6 (1C, C-1'$_{4\text{-}\{[4\text{-}(morpholinomethyl)phenyl]ethynyl\}phenyl}$), 138.8 (1C, C-4''$_{4\text{-}(morpholinomethyl)phenyl}$), the signals for C-3'''$_{pyrazole}$, C-5'''$_{pyrazole}$, *C*ONHCH_2, and *C*ONHOH cannot be observed in the spectrum; IR (neat): $\tilde{\nu}$ [cm^{-1}] = 3198, 2950, 2862, 1649, 1547, 1456, 1352, 1111, 1005, 863, 819, 793, 764, 616, 540, 511; HRMS (*m*/*z*): [M+H]$^+$ calcd for $C_{27}H_{30}N_5O_5$: 504.2241, found: 504.2229; HPLC (method 2): t_R = 12.3 min, purity 100%.

(*S*)-*N*-{3-(Hydroxyamino)-2-[(4-{[4-(morpholinomethyl)phenyl]ethynyl}benzyl)oxy]-3-oxopropyl}-3-phenyl-1*H*-pyrazole-5-carboxamide ((*S*)-13m)

Under ice-cooling, an aqueous solution of hydroxylamine (50 wt%, 3 mL) was added to a solution of (*S*)-**39m** (120 mg, 0.21 mmol) in a mixture of THF (4 mL) and isopropanol (4 mL). After stirring the reaction mixture for 5 min at 0 °C, stirring was continued for 48 h at ambient temperature. Then, the solvent was removed *in vacuo* and the residue was purified by automatic flash column chromatography using a Biotage® Isolera™ One system (10% → 75% ACN in H_2O, Biotage® SNAP Ultra C18 30 g). Fractions containing the desired product were combined and subjected to lyophilization to give (*S*)-**13m** as colorless solid (94 mg, 0.16 mmol, 76%). m.p.: 139 °C; $[\alpha]_D^{20}$ = -24.8 (1.5, methanol); HPLC (method 2): t_R = 13.0 min, purity 99.7%.

(*R*)-*N*-{3-(Hydroxyamino)-2-[(4-{[4-(morpholinomethyl)phenyl]ethynyl}benzyl)oxy]-3-oxopropyl}-3-phenyl-1*H*-pyrazole-5-carboxamide ((*R*)-13m)

Under ice-cooling, an aqueous solution of hydroxylamine (50 wt%, 3.5 mL) was added to a solution of (*R*)-**39m** (120 mg, 0.21 mmol) in a mixture of THF (5 mL) and isopropanol (5 mL). After stirring the reaction mixture for 5 min at 0 °C, stirring was continued for 72 h at ambient temperature. Then, the solvent was removed *in vacuo* and the residue was purified by automatic flash column chromatography using a Biotage® Isolera™ One system (10% → 75% ACN in H_2O, Biotage® SNAP Ultra C18 12 g). Fractions containing the desired product were combined and subjected to

lyophilization to give (*R*)-**13m** as colorless solid (83 mg, 0.14 mmol, 69%). m.p.: 140 °C; $[\alpha]_D^{20}$ = +24.7 (2.0, methanol); HPLC (method 2): t_R = 13.1 min, purity 100%.

Spectroscopic data of (*S*)-**13m** *and* (*R*)-**13m**:

^{1}H NMR (DMSO-d_6): δ [ppm] = 2.29 – 2.40 (m, 4H, N(CH_2CH$_2$)$_2$O), 3.48 (s, 2H, NCH_2Ar), 3.48 – 3.53 (m, 1H, OCHCH_2NH), 3.55 – 3.63 (m, 5H, N(CH$_2$CH_2)$_2$O, OCHCH_2NH (1H)), 3.94 – 4.07 (m, 1H, OC*H*CH$_2$NH), 4.46 (d, *J* = 12.6 Hz, 1H, OCH_2Ar), 4.63 (d, *J* = 12.6 Hz, 1H, OCH_2Ar), 7.07 (s br, 0.7H, 4'''-$H_{pyrazole}$), 7.22 – 7.52 (11.3H, 4'''-$H_{pyrazole}$ (0.3H), 2'-$H_{4\text{-}\{[4\text{-}(morpholinomethyl)phenyl]ethynyl\}phenyl}$, 3'-$H_{4\text{-}\{[4\text{-}(morpholinomethyl)phenyl]ethynyl\}phenyl}$, 5'-$H_{4\text{-}\{[4\text{-}(morpholinomethyl)phenyl]ethynyl\}phenyl}$, 6'-$H_{4\text{-}\{[4\text{-}(morpholinomethyl)phenyl]ethynyl\}phenyl}$, 2''-$H_{4\text{-}(morpholinomethyl)phenyl}$, 3''-$H_{4\text{-}(morpholinomethyl)phenyl}$, 5''-$H_{4\text{-}(morpholinomethyl)phenyl}$, 6''-$H_{4\text{-}(morpholinomethyl)phenyl}$, 3''''-$H_{phenyl}$, 4''''-$H_{phenyl}$, 5''''-$H_{phenyl}$), 7.75 – 7.84 (m, 2H, 2''''-H_{phenyl}, 6''''-H_{phenyl}), 8.06 (s br, 0.7H, CON*H*CH$_2$), 8.64 (s br, 0.3H, CON*H*CH$_2$), 9.00 (s br, 1H, CON*H*OH), 10.91 (s br, 1H, CONHO*H*), 13.56 – 13.73 (m, 1H, 1'''-$H_{pyrazole}$), two annular tautomers exist in the ratio 70:30; ^{13}C NMR (DMSO-d_6): δ [ppm] = 40.9 (1C, OCH*C*H$_2$NH), 53.1 (2C, N(*C*H$_2$CH$_2$)$_2$O), 62.0 (1C, Ar*C*H$_2$N), 66.2 (2C, N(CH$_2$*C*H$_2$)$_2$O), 70.6 (1C, O*C*H$_2$Ar), 76.7 (1C, O*C*HCH$_2$NH), 89.0 (1C, *C*≡C), 89.2 (1C, *C*≡C), 102.5 (1C, C-4'''$_{pyrazole}$), 120.8 (1C, C-1''$_{4\text{-}(morpholinomethyl)phenyl}$), 121.4 (1C, C-4'$_{4\text{-}\{[4\text{-}(morpholinomethyl)phenyl]ethynyl\}phenyl}$), 125.2 (2C, C-2''''$_{phenyl}$, C-6''''$_{phenyl}$), 127.8 (2C, C-2'$_{4\text{-}\{[4\text{-}(morpholinomethyl)phenyl]ethynyl\}phenyl}$, C-6'$_{4\text{-}\{[4\text{-}(morpholinomethyl)phenyl]ethynyl\}phenyl}$), 128.2 (1C, C-4''''$_{phenyl}$), 129.0 (2C, C-3''''$_{phenyl}$, C-5''''$_{phenyl}$), 129.1 (2C, C-3''$_{4\text{-}(morpholinomethyl)phenyl}$, C-5''$_{4\text{-}(morpholinomethyl)phenyl}$), 131.1 (2C, $C_{arom.}$), 131.2 (2C, $C_{arom.}$), 138.6 (1C, C-1'$_{4\text{-}\{[4\text{-}(morpholinomethyl)phenyl]ethynyl\}phenyl}$), 138.8 (1C, C-4''$_{4\text{-}(morpholinomethyl)phenyl}$), 166.1 (1C, *C*ONHOH), the signals for C-1''''$_{phenyl}$, C-3'''$_{pyrazole}$, C-5'''$_{pyrazole}$, and *C*ONHCH$_2$ cannot be observed in the spectrum; IR (neat): $\tilde{\nu}$ [cm^{-1}] = 3211, 2862, 2816, 1652, 1545, 1457,

1412, 1292, 1111, 1005, 863, 820, 762, 693, 539, 515; HRMS (*m/z*): $[M+H]^+$ calcd for $C_{33}H_{34}N_5O_5$: 580.2554, found: 580.2550.

(*S*)-*N*-{3-(Hydroxyamino)-2-[(4-{[4-(morpholinomethyl)phenyl]ethynyl}benzyl)oxy]-3-oxopropyl}-3,5-dimethylisoxazole-4-carboxamide ((*S*)-13n)

Under ice-cooling, an aqueous solution of hydroxylamine (50 wt%, 3 mL) was added to a solution of (*S*)-**39n** (140 mg, 0.25 mmol) in a mixture of THF (5 mL) and isopropanol (5 mL). After stirring the reaction mixture for 5 min at 0 °C, stirring was continued for 48 h at ambient temperature. Then, the solvent was removed *in vacuo* and the residue was purified by automatic flash column chromatography using a Biotage® Isolera™ One system (10% → 80% ACN in H_2O, Biotage® SNAP Ultra C18 30 g). Fractions containing the desired product were combined and subjected to lyophilization to give (*S*)-**13n** as colorless solid (110 mg, 0.21 mmol, 84%). m.p.: 137 °C; $[\alpha]_D^{20}$ = -49.9 (1.2, methanol); HPLC (method 2): t_R = 12.7 min, purity 99.7%.

(*R*)-*N*-{3-(Hydroxyamino)-2-[(4-{[4-(morpholinomethyl)phenyl]ethynyl}benzyl)oxy]-3-oxopropyl}-3,5-dimethylisoxazole-4-carboxamide ((*R*)-13n)

Under ice-cooling, an aqueous solution of hydroxylamine (50 wt%, 3 mL) was added to a solution of (*R*)-**39n** (140 mg, 0.26 mmol) in a mixture of THF (5 mL) and isopropanol (5 mL). After stirring the reaction mixture for 5 min at 0 °C, stirring was continued for 48 h at ambient temperature. Then, the solvent was removed *in vacuo* and the residue was purified by automatic flash column chromatography using a Biotage® Isolera™ One system (10% → 80% ACN in H_2O, Biotage® SNAP Ultra C18 12 g). Fractions containing the desired product were combined and subjected to lyophilization to give (*R*)-**13n** as colorless solid (42 mg,

0.079 mmol, 31%). m.p.: 134 °C; $[\alpha]_D^{20}$ = +46.7 (1.2, methanol); HPLC (method 2): t_R = 12.7 min, purity 95.7%.

Spectroscopic data of (*S*)-**13n** *and* (*R*)-**13n**:

^{1}H NMR (DMSO-*d*$_6$): δ [ppm] = 2.24 (s, 3H, *H*$_3$CC-3''$_{isoxazole}$), 2.32 – 2.39 (m, 4H, N(C*H*$_2$CH$_2$)$_2$O), 2.45 (s, 3H, *H*$_3$CC-5''$_{isoxazole}$), 3.43 – 3.54 (m, 2H, OCHC*H*$_2$NH), 3.49 (s, 2H, NC*H*$_2$Ar), 3.54 – 3.61 (m, 4H, N(CH$_2$C*H*$_2$)$_2$O), 3.97 (dd, *J* = 7.1/5.3 Hz, 1H, OC*H*CH$_2$NH), 4.45 (d, *J* = 12.4 Hz, 1H, OC*H*$_2$Ar), 4.59 (d, *J* = 12.4 Hz, 1H, OC*H*$_2$Ar), 7.34 – 7.38 (m, 2H, 3''-H$_{4\text{-(morpholinomethyl)phenyl}}$, 5''-H$_{4\text{-(morpholinomethyl)phenyl}}$), 7.39 – 7.43 (m, 2H, 2'-H$_{4\text{-\{[4-(morpholinomethyl)phenyl]ethynyl\}phenyl}}$, 6'-H$_{4\text{-\{[4-(morpholinomethyl)phenyl]ethynyl\}phenyl}}$), 7.48 – 7.53 (m, 4H, 3'-H$_{4\text{-\{[4-(morpholinomethyl)phenyl]ethynyl\}phenyl}}$, 5'-H$_{4\text{-\{[4-(morpholinomethyl)phenyl]ethynyl\}phenyl}}$, 2''-H$_{4\text{-(morpholinomethyl)phenyl}}$, 6''-H$_{4\text{-(morpholinomethyl)phenyl}}$), 8.09 (t, *J* = 5.8 Hz, 1H, CON*H*CH$_2$), 9.01 (s br, 1H, CON*H*OH), 10.90 (s br, 1H, CONHO*H*); ^{13}C NMR (DMSO-*d*$_6$): δ [ppm] = 10.5 (1C, H$_3$*C*C-3'''$_{isoxazole}$), 12.1 (1C, H$_3$*C*C-5'''$_{isoxazole}$), 40.9 (1C, OCH*C*H$_2$NH), 53.2 (2C, N(*C*H$_2$CH$_2$)$_2$O), 62.0 (1C, Ar*C*H$_2$N), 66.2 (2C, N(CH$_2$*C*H$_2$)$_2$O), 70.5 (1C, O*C*H$_2$Ar), 76.5 (1C, O*C*HCH$_2$NH), 89.0 (1C, *C*≡C), 89.3 (1C, *C*≡C), 112.7 (1C, C-4'''$_{isoxazole}$), 120.8 (1C, C-1''$_{4\text{-(morpholinomethyl)phenyl}}$), 121.5 (1C, C-4'$_{4\text{-\{[4-(morpholinomethyl)phenyl]ethynyl\}phenyl}}$), 127.8 (2C, C-2'$_{4\text{-\{[4-(morpholinomethyl)phenyl]ethynyl\}phenyl}}$, C-6'$_{4\text{-\{[4-(morpholinomethyl)phenyl]ethynyl\}phenyl}}$), 129.2 (2C, C-3''$_{4\text{-(morpholinomethyl)phenyl}}$, C-5''$_{4\text{-(morpholinomethyl)phenyl}}$), 131.16 (2C, C$_{arom.}$), 131.24 (2C, C$_{arom.}$), 138.5 (1C, C-1'$_{4\text{-\{[4-(morpholinomethyl)phenyl]ethynyl\}phenyl}}$), 138.9 (1C, C-4''$_{4\text{-(morpholinomethyl)phenyl}}$), 158.2 (1C, C-3'''$_{isoxazole}$), 161.6 (1C, *C*ONHCH$_2$), 166.0 (1C, *C*ONHOH), 169.6 (1C, C-5'''$_{isoxazole}$); IR (neat): $\tilde{\nu}$ [cm^{-1}] = 3288, 2859, 2813, 1645, 1518, 1422, 1309, 1114, 1005, 914, 865, 831, 821, 793, 711, 516; HRMS (*m*/*z*): [M+H]$^+$ calcd for $C_{29}H_{33}N_4O_6$: 533.2395, found: 533.2395.

^{1}H and ^{13}C NMR spectra of representative compounds

Compound **15**

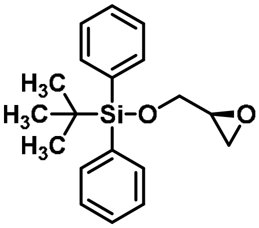

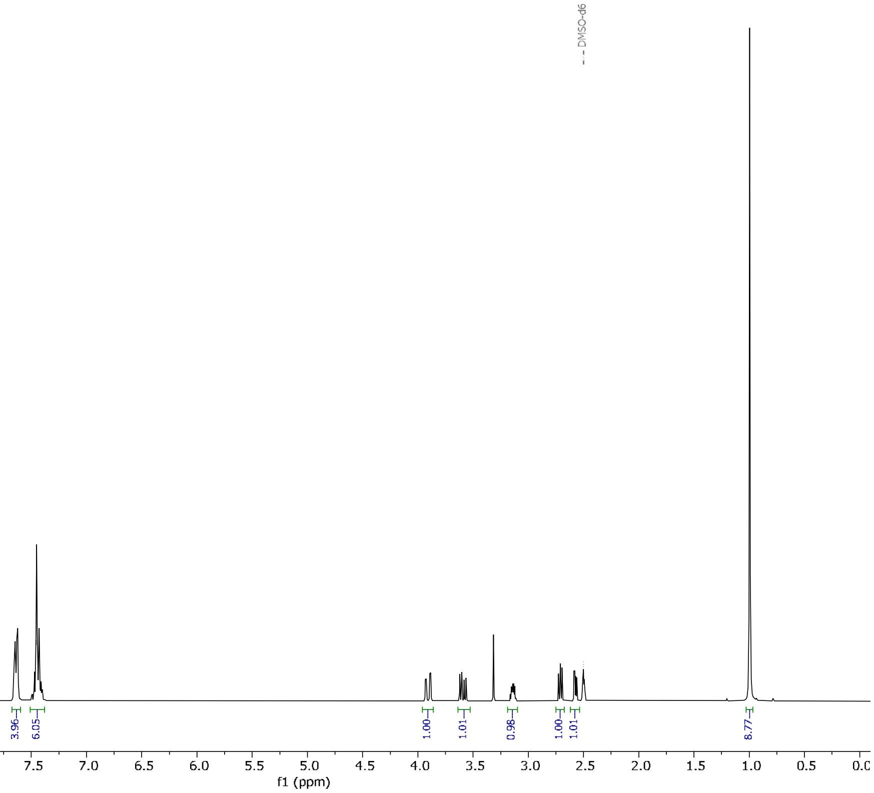

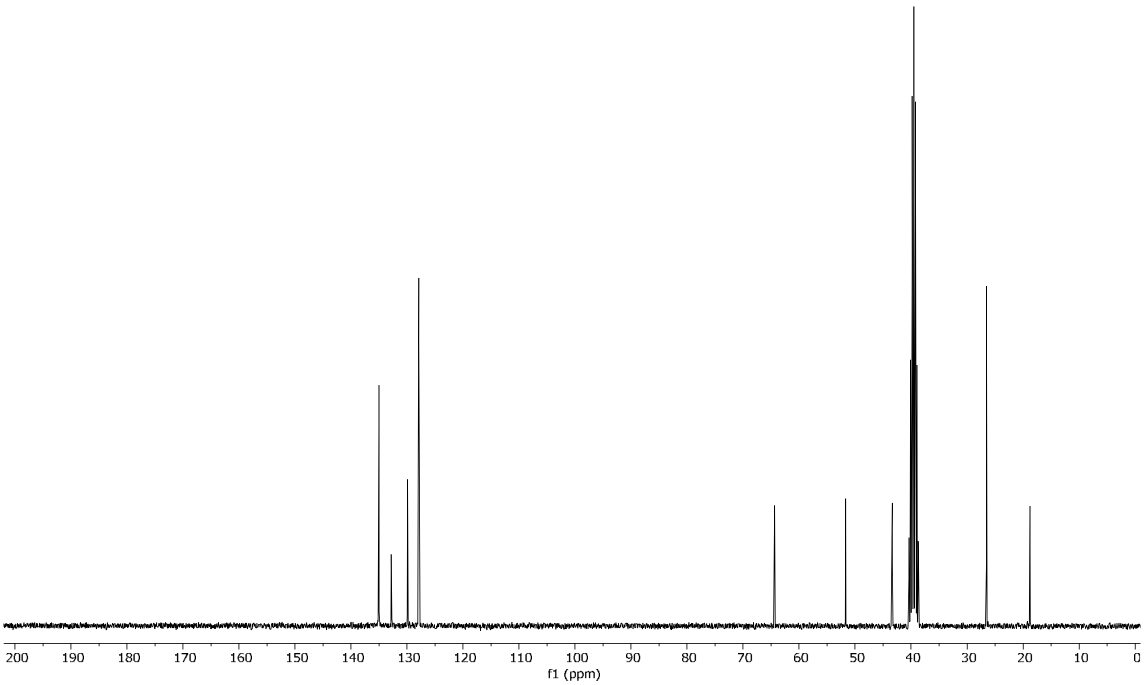

Compound **16**

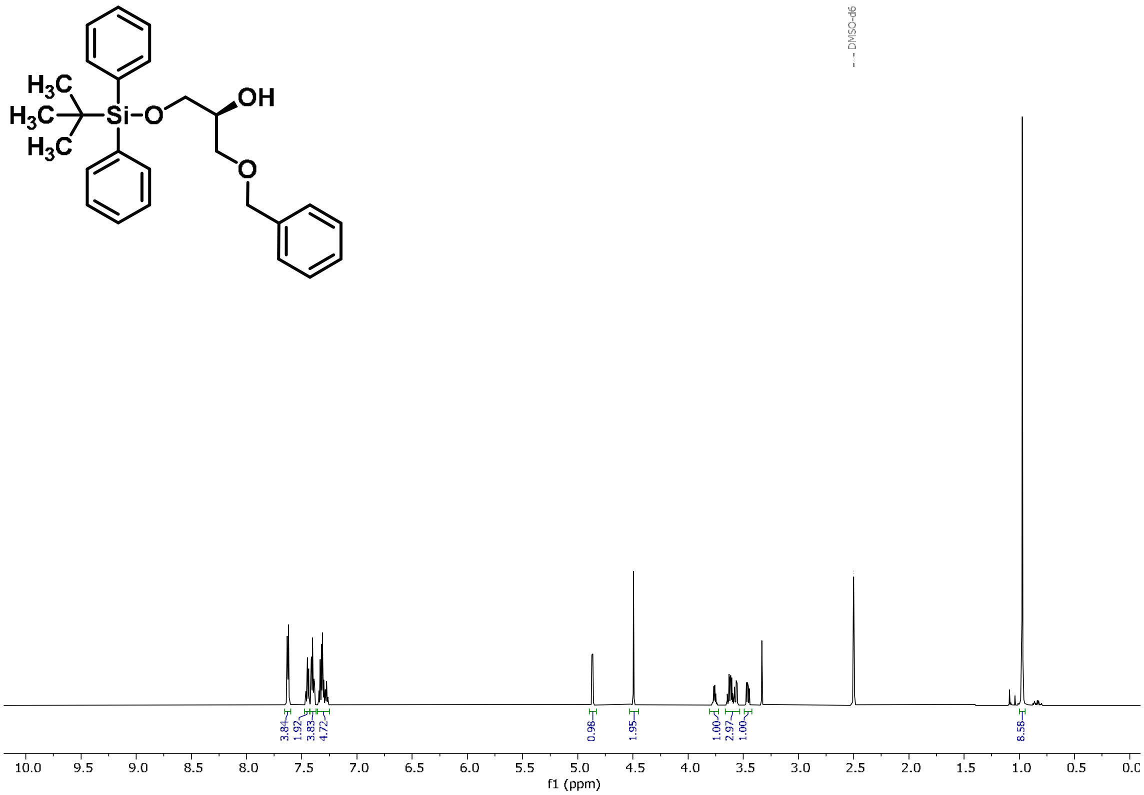

H3C
H3C
H3C
Si
O
OH
O
DMSO-d6
3.84
1.92
3.83
4.72
0.98
1.95
1.00
2.97
1.00
8.58
10.0 9.5 9.0 8.5 8.0 7.5 7.0 6.5 6.0 5.5 5.0 4.5 4.0 3.5 3.0 2.5 2.0 1.5 1.0 0.5 0.0
f1 (ppm)

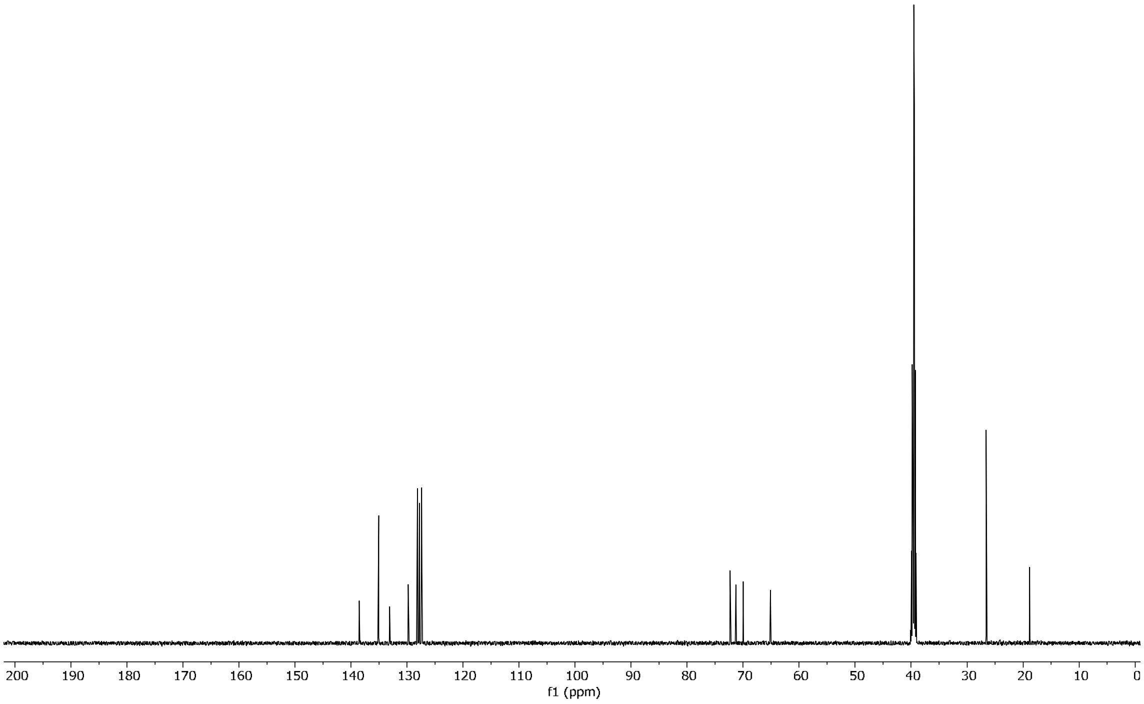

200 190 180 170 160 150 140 130 120 110 100 90 80 70 60 50 40 30 20 10 0
f1 (ppm)

Compound **17**

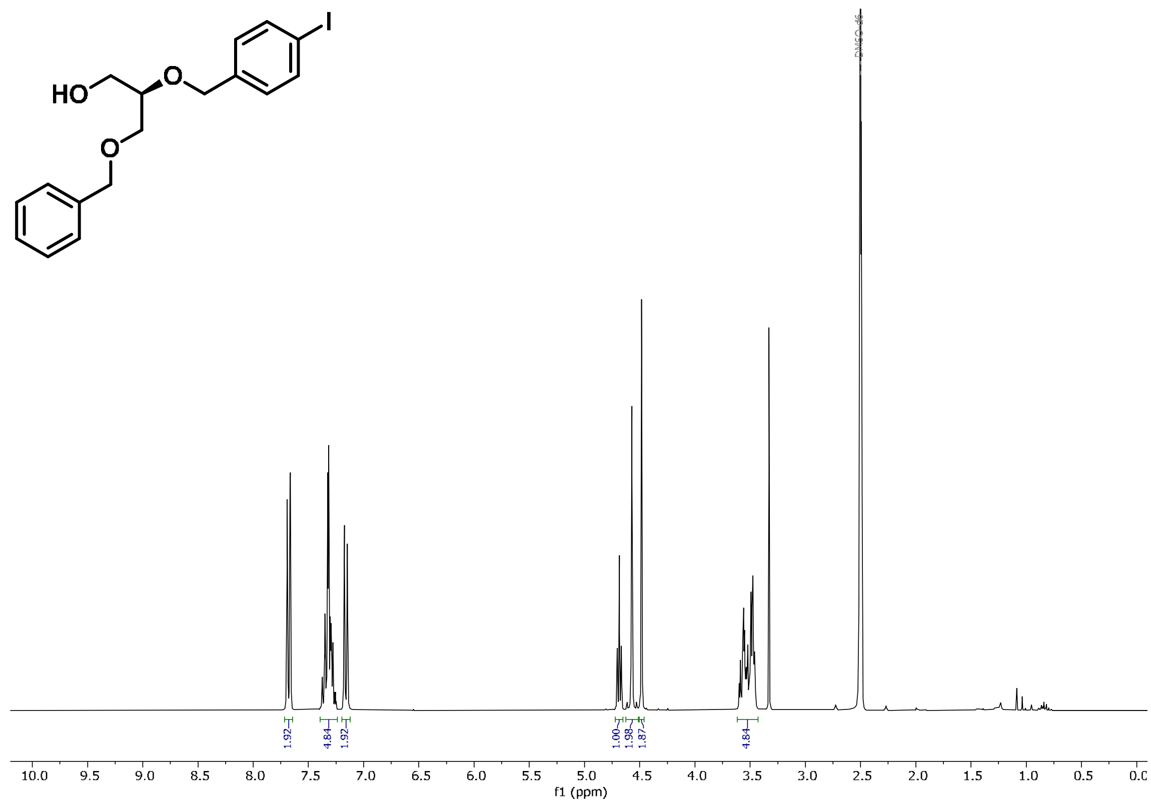
HO
O
O
I
DMSO-d6
1.92
4.84
1.92
1.00
1.98
1.87
4.84
10.0 9.5 9.0 8.5 8.0 7.5 7.0 6.5 6.0 5.5 5.0 4.5 4.0 3.5 3.0 2.5 2.0 1.5 1.0 0.5 0.0
f1 (ppm)

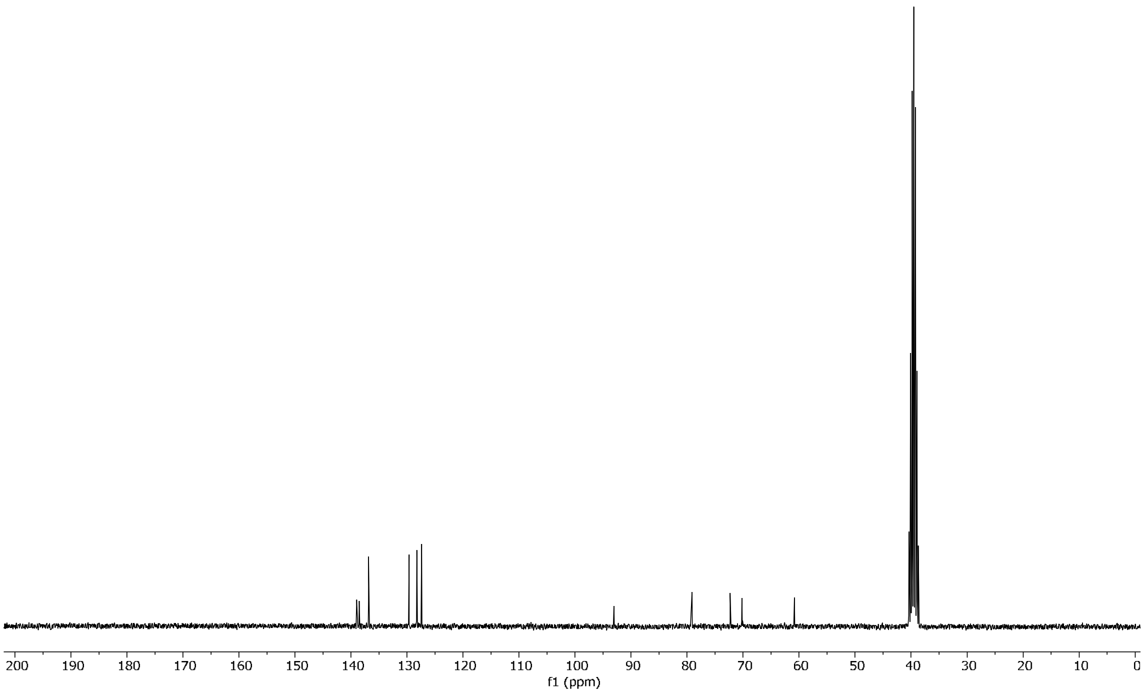
200 190 180 170 160 150 140 130 120 110 100 90 80 70 60 50 40 30 20 10 0
f1 (ppm)

Compound **21**

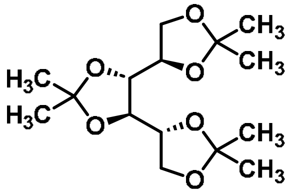

H3C
H3C
CH3
CH3
CH3
CH3
O

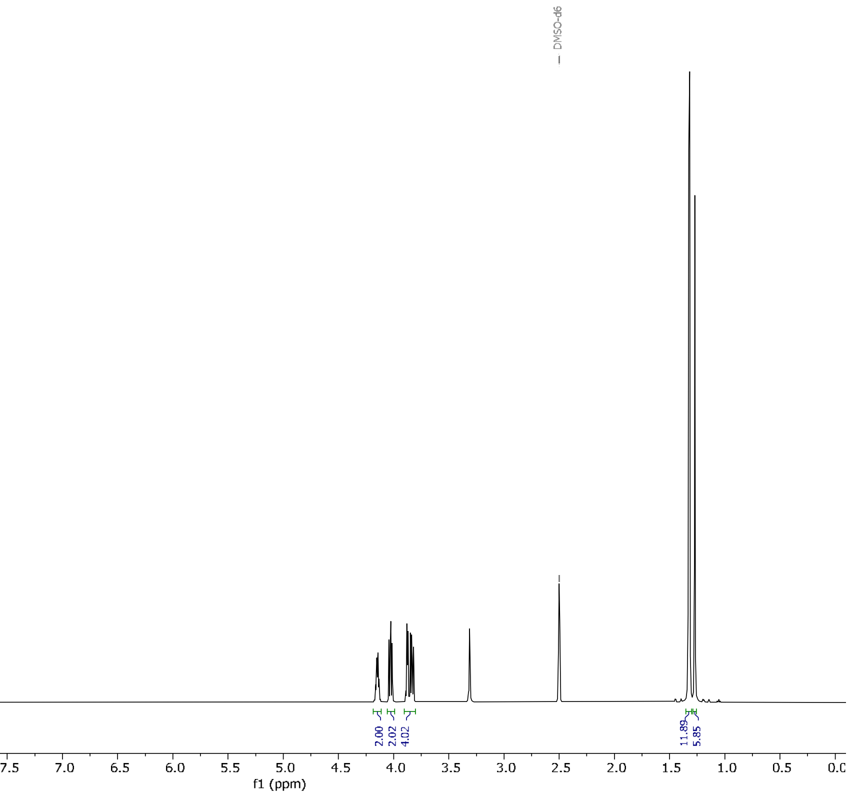

DMSO-d6
2.00
2.02
4.02
11.89
5.85
f1 (ppm)

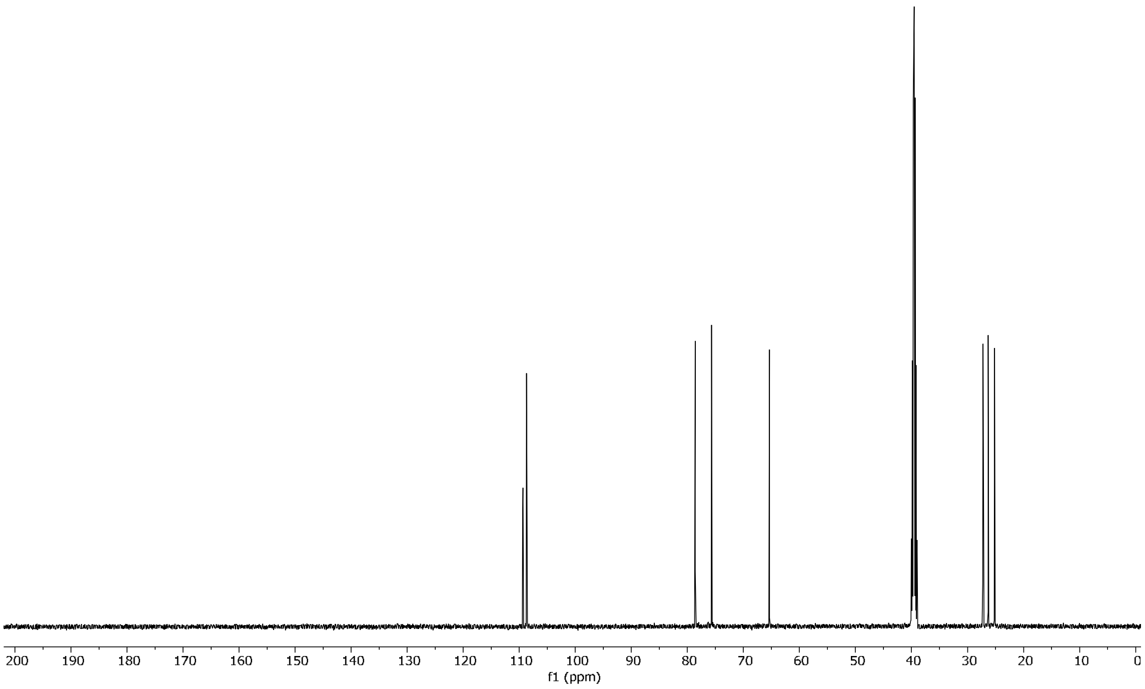

f1 (ppm)

Compound **22**

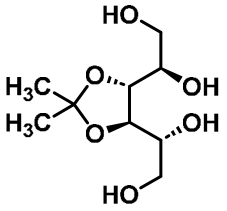
HO
OH
OH
HO
H3C
H3C
O
O

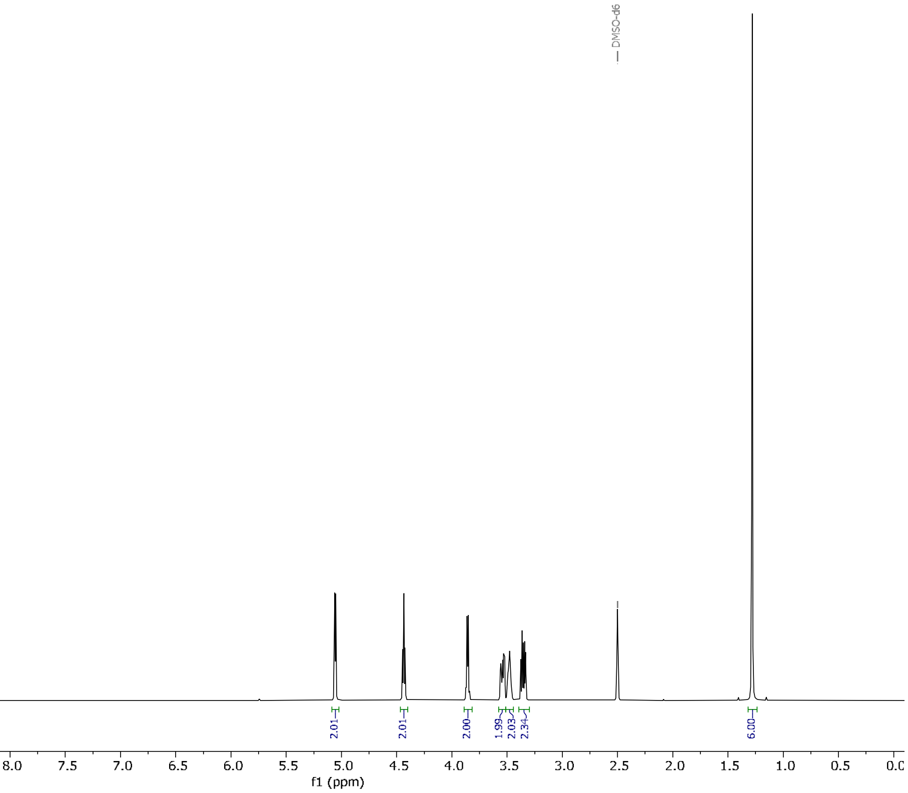
DMSO-d6
2.01
2.01
2.00
1.99
2.03
2.34
6.00
10.0 9.5 9.0 8.5 8.0 7.5 7.0 6.5 6.0 5.5 5.0 4.5 4.0 3.5 3.0 2.5 2.0 1.5 1.0 0.5 0.0
f1 (ppm)

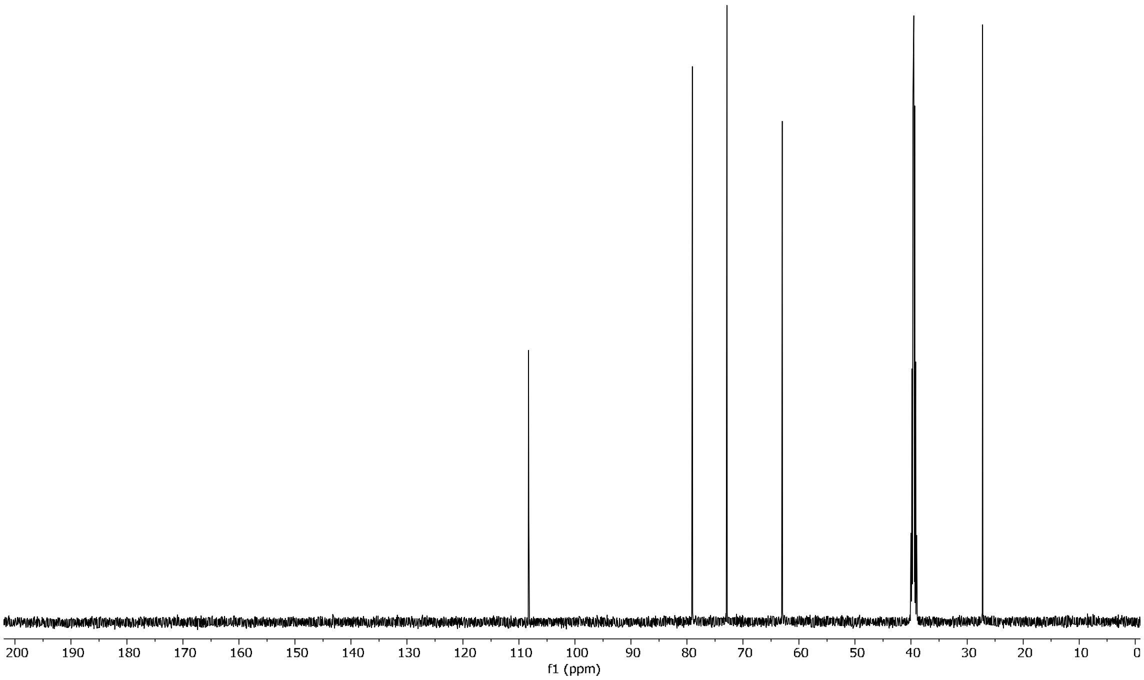
200 190 180 170 160 150 140 130 120 110 100 90 80 70 60 50 40 30 20 10 0
f1 (ppm)

Compound **23**

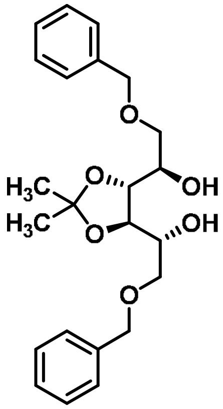
H3C
H3C
O
O
OH
OH
O
O

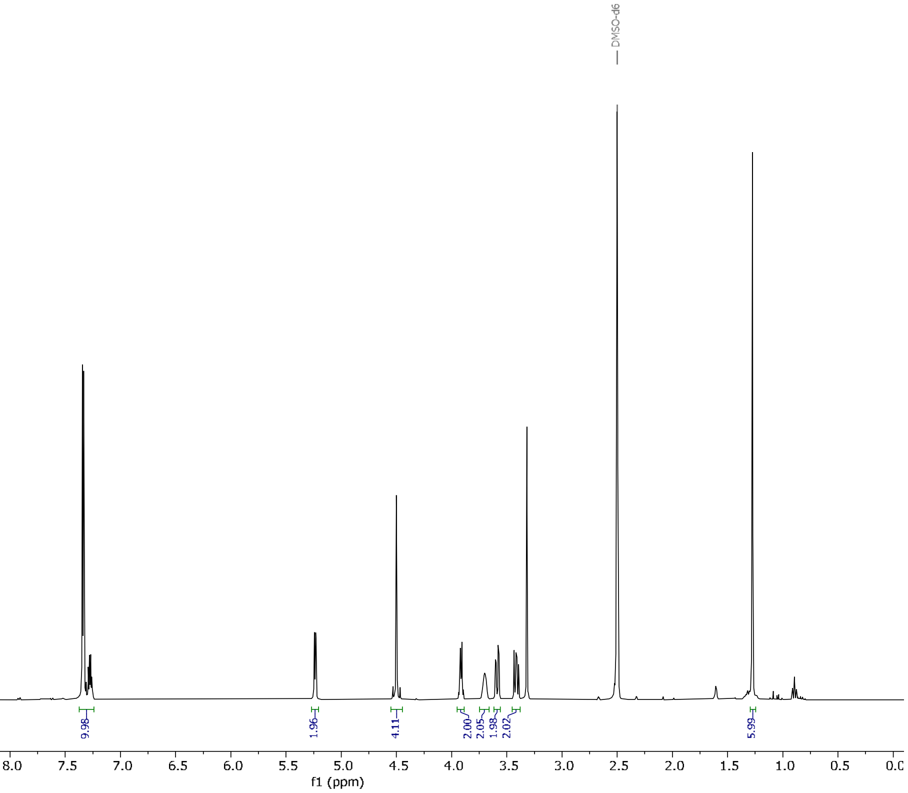
DMSO-d6
9.98
1.96
4.11
2.00
2.05
1.98
2.02
5.99
10.0 9.5 9.0 8.5 8.0 7.5 7.0 6.5 6.0 5.5 5.0 4.5 4.0 3.5 3.0 2.5 2.0 1.5 1.0 0.5 0.0
f1 (ppm)

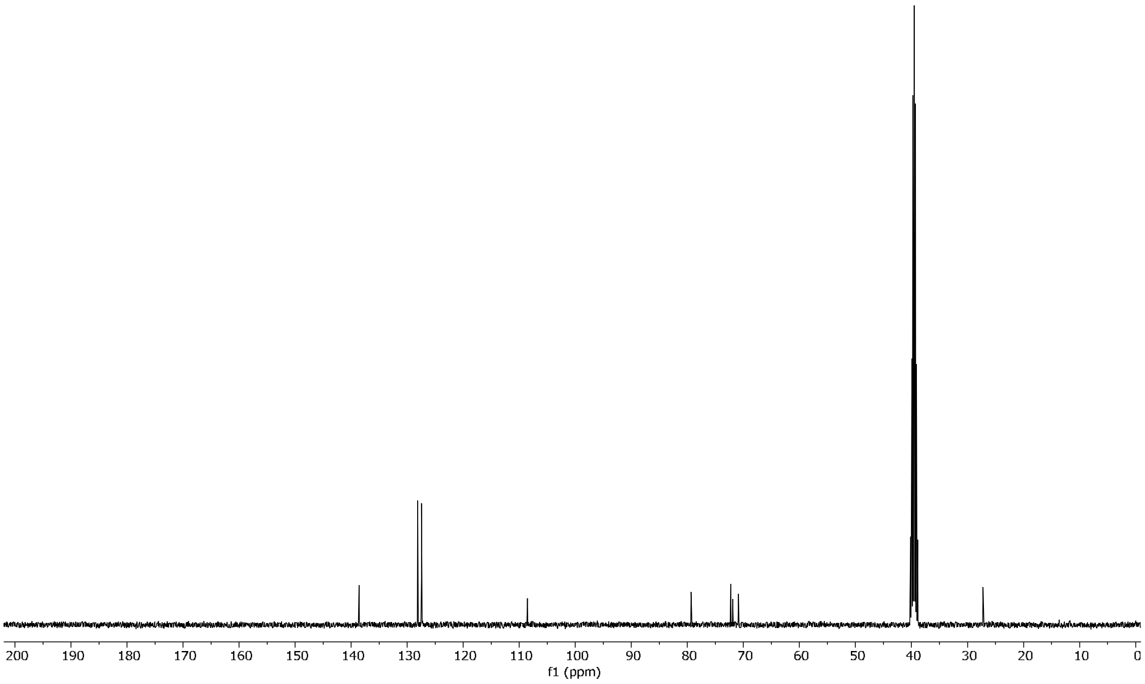
200 190 180 170 160 150 140 130 120 110 100 90 80 70 60 50 40 30 20 10 0
f1 (ppm)

Compound **24**

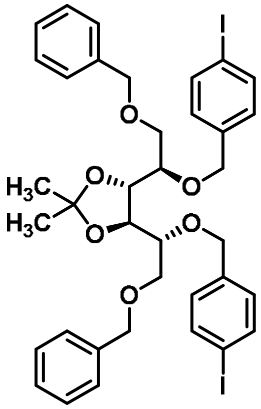

I
O
H3C
H3C
O
O
O
O
O
I

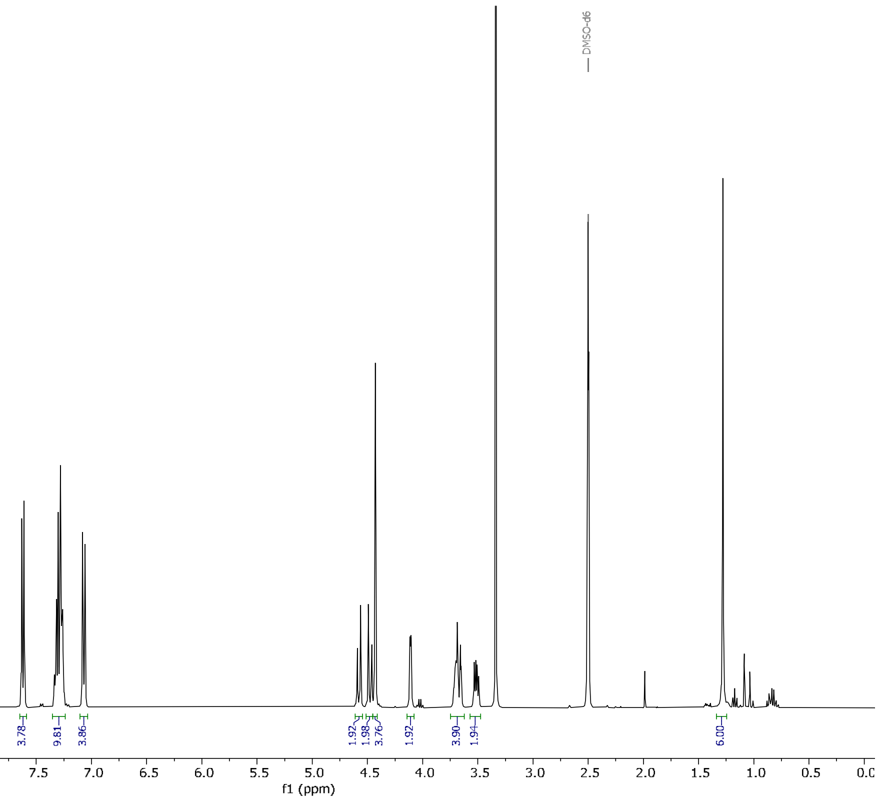

DMSO-d6
3.78
9.81
3.86
1.92
1.98
3.76
1.92
3.90
1.94
6.00
10.0 9.5 9.0 8.5 8.0 7.5 7.0 6.5 6.0 5.5 5.0 4.5 4.0 3.5 3.0 2.5 2.0 1.5 1.0 0.5 0.0
f1 (ppm)

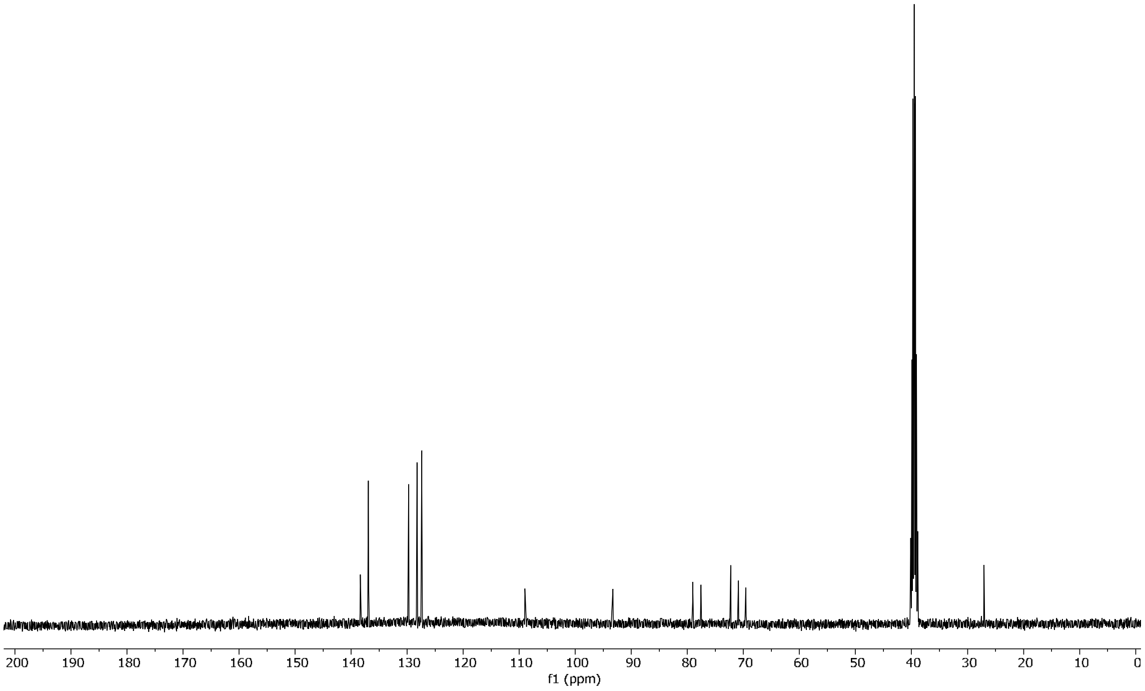

200 190 180 170 160 150 140 130 120 110 100 90 80 70 60 50 40 30 20 10 0
f1 (ppm)

Compound **25**

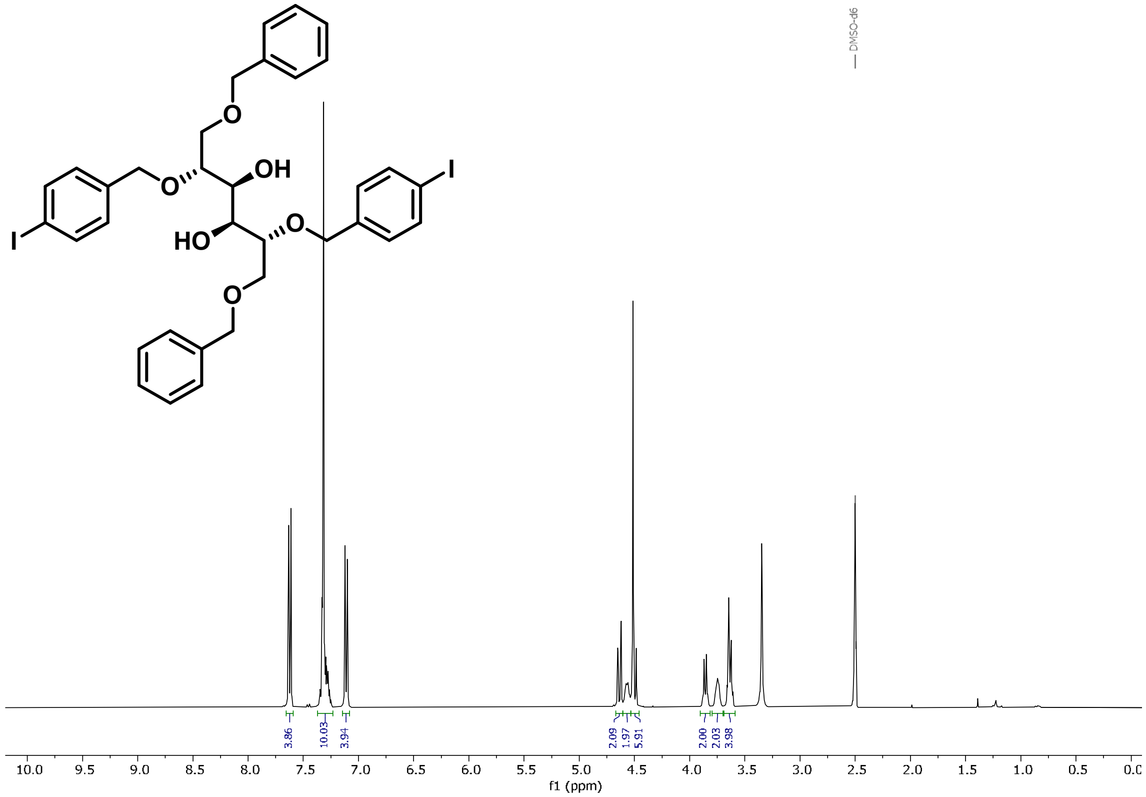

OH
HO
O
I
DMSO-d6
3.86
10.03
3.94
2.09
1.97
5.91
2.00
2.03
3.98
10.0 9.5 9.0 8.5 8.0 7.5 7.0 6.5 6.0 5.5 5.0 4.5 4.0 3.5 3.0 2.5 2.0 1.5 1.0 0.5 0.0
f1 (ppm)

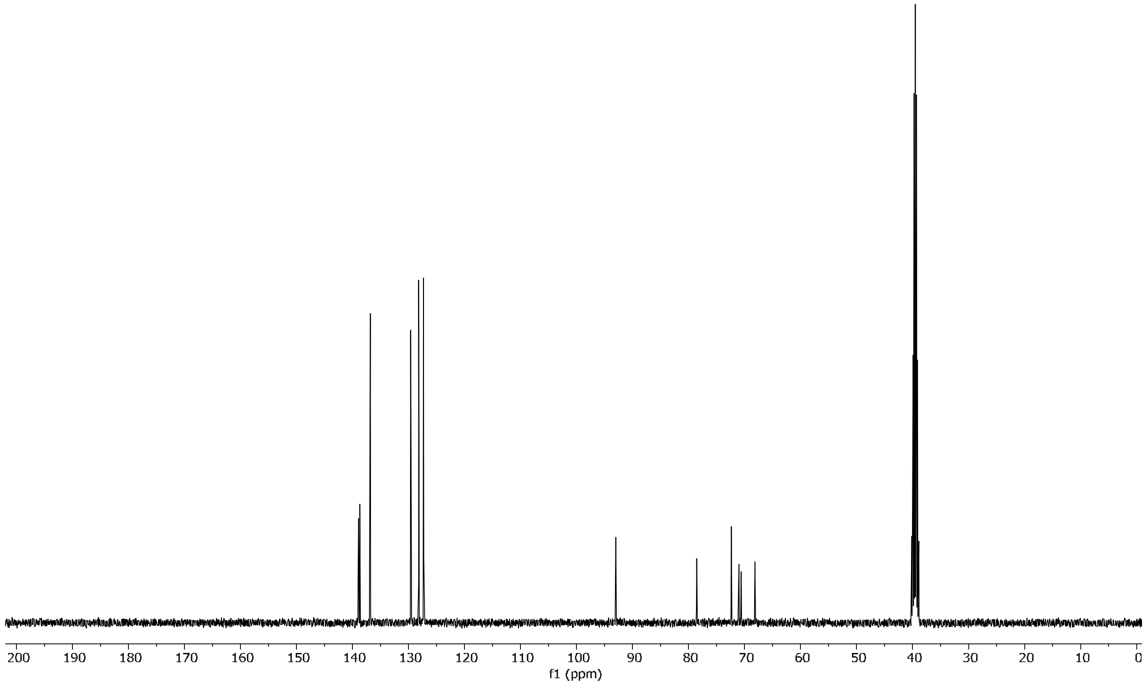

200 190 180 170 160 150 140 130 120 110 100 90 80 70 60 50 40 30 20 10 0
f1 (ppm)

Compound (*S*)-**18**

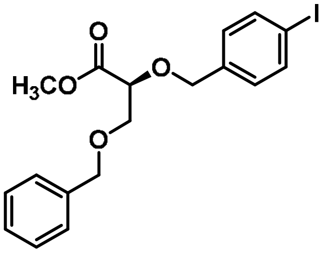

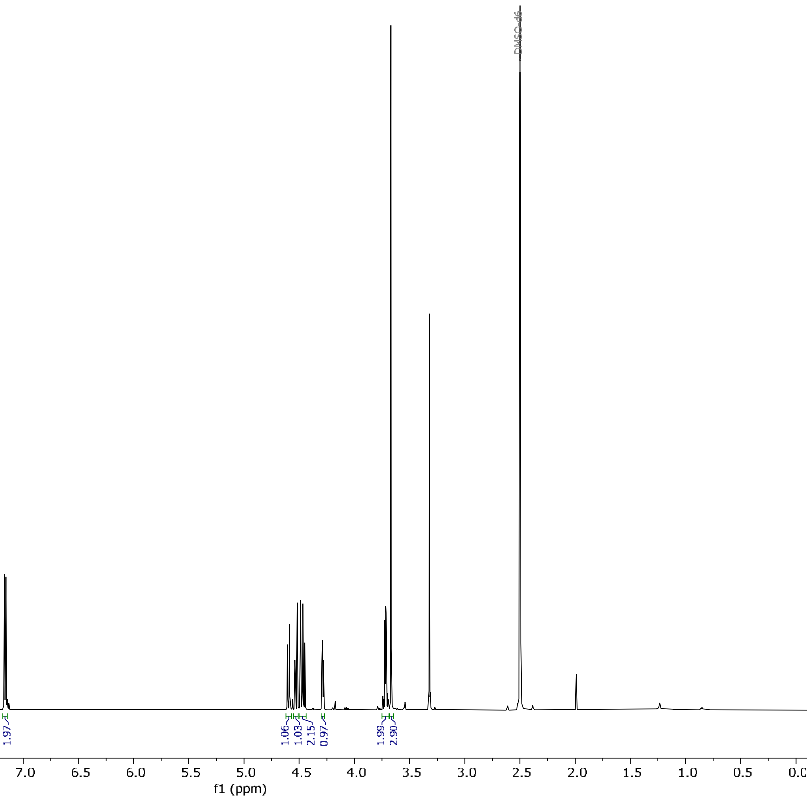

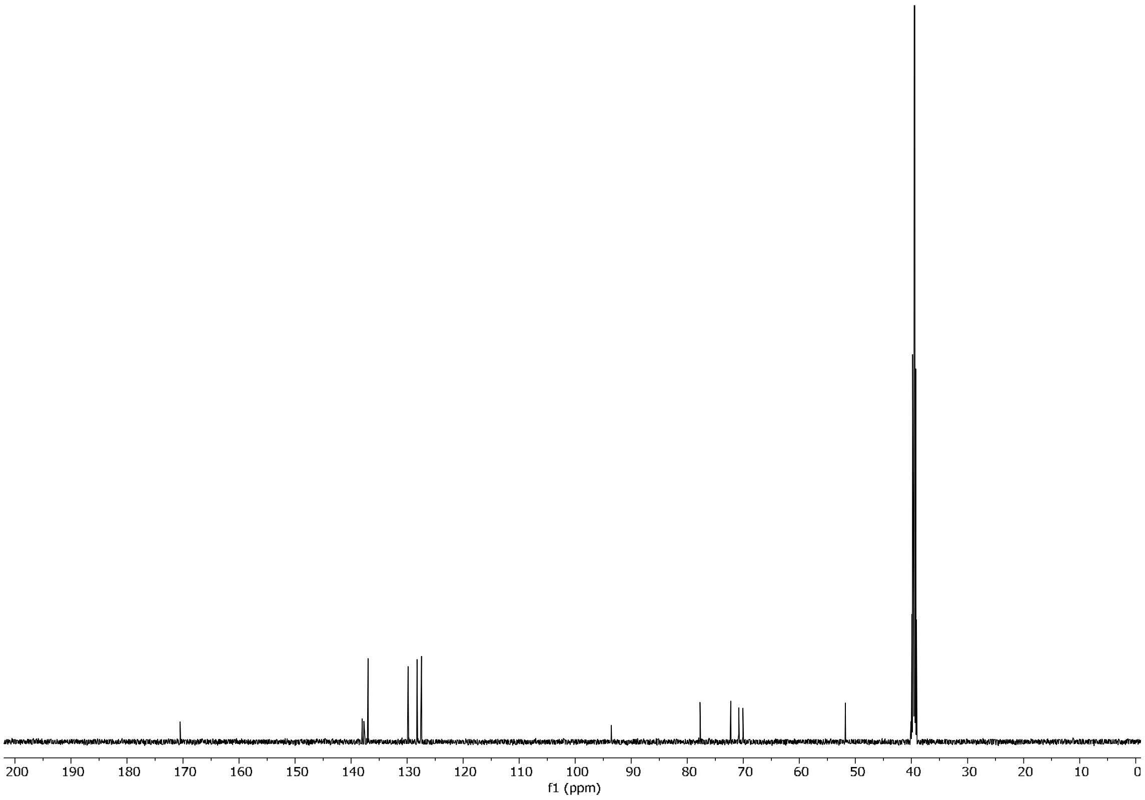

Compound (*S*)-**19**

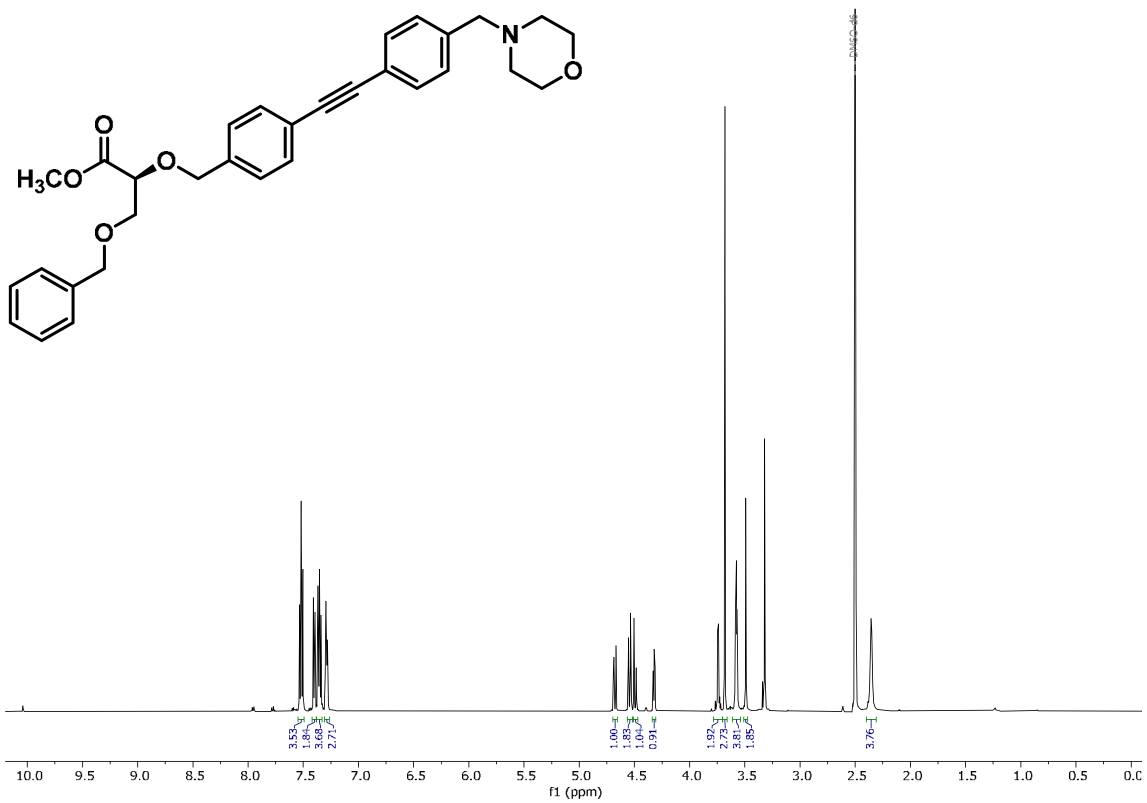

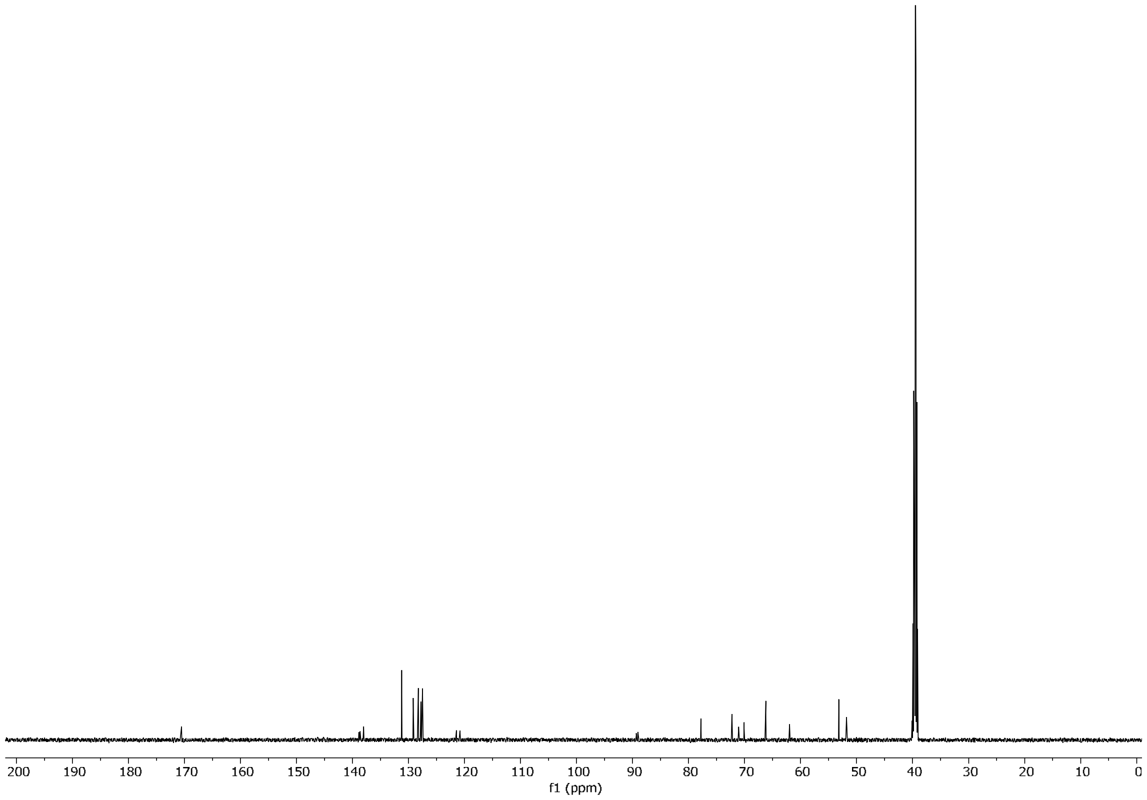

Compound (*S*)-**11a**

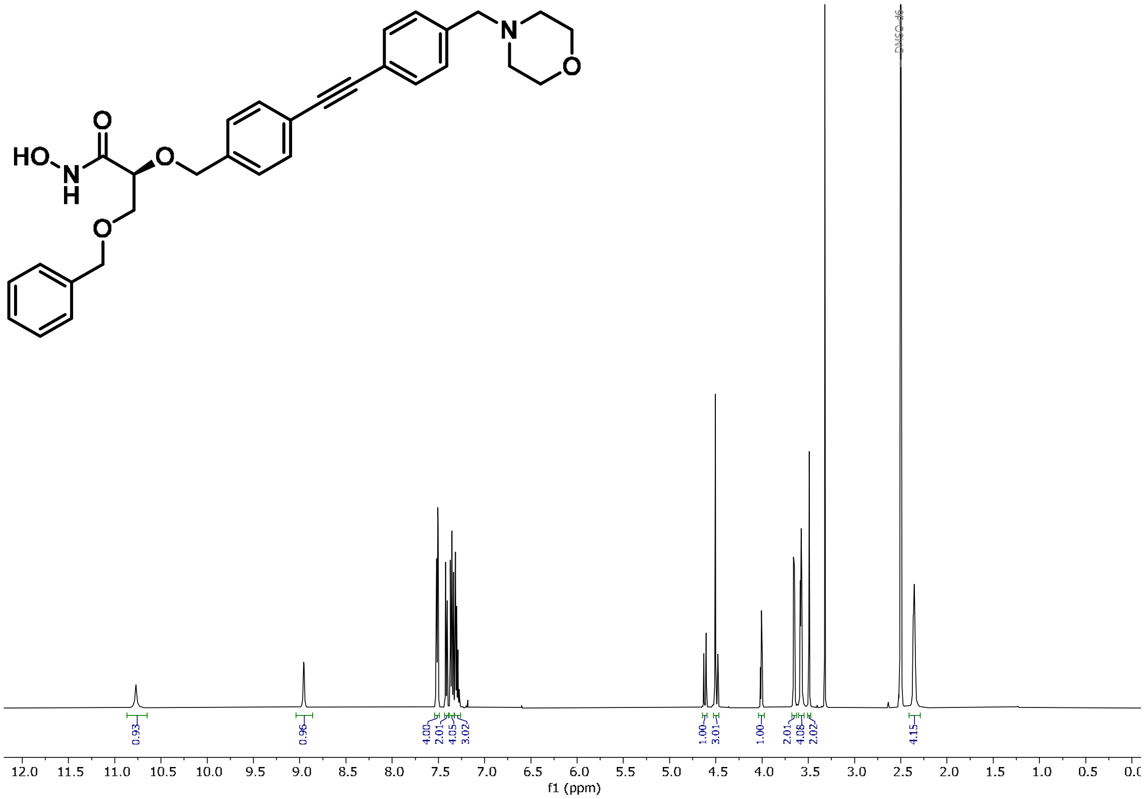

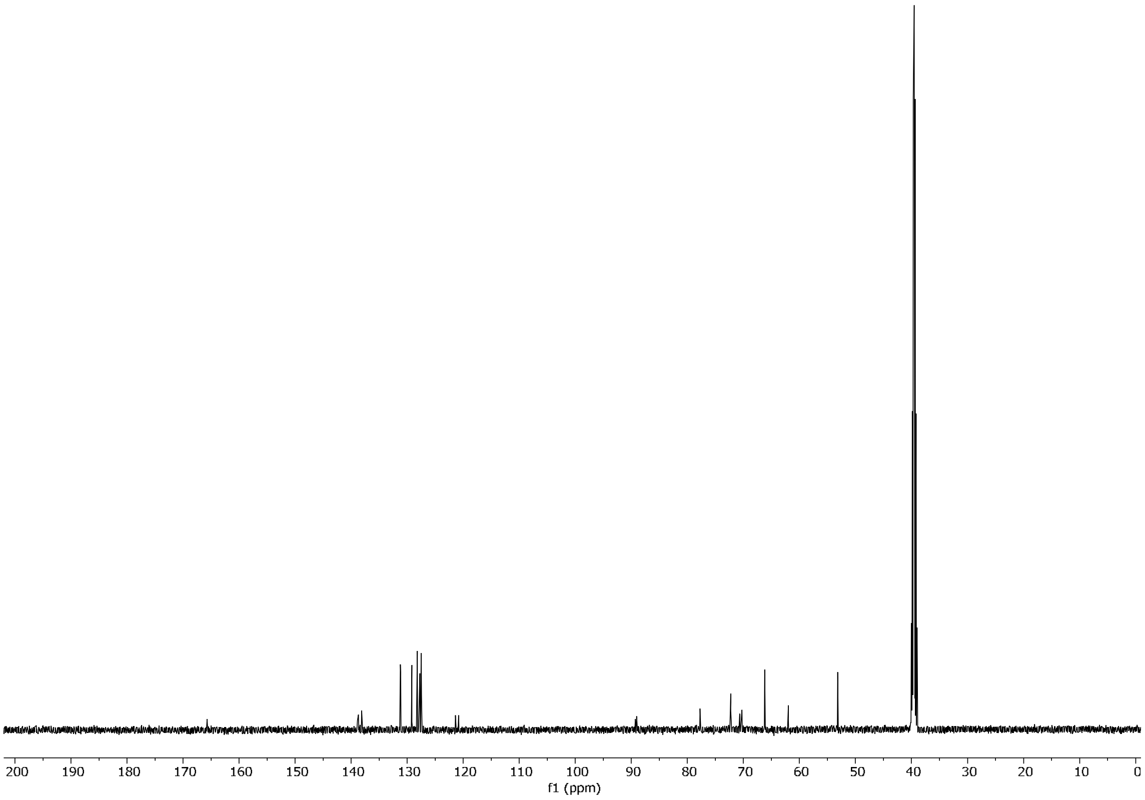

Compound **26**

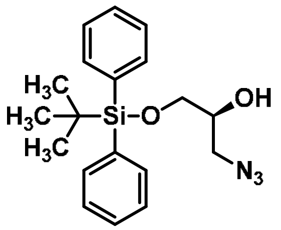
H3C
H3C
H3C
Si
O
OH
N3

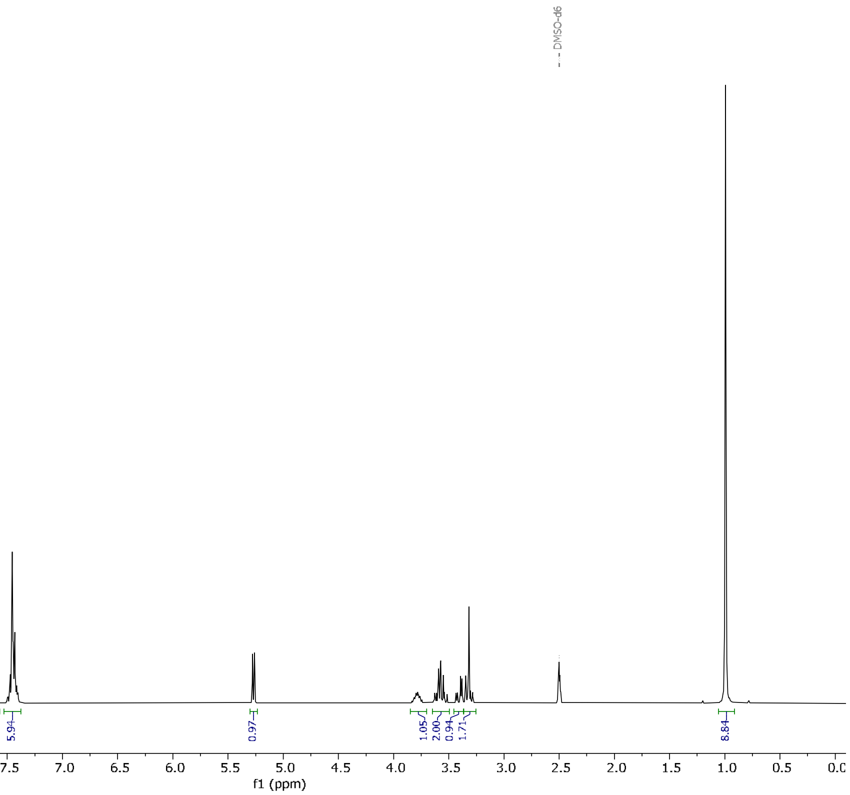
DMSO-d6
3.98
5.91
0.97
1.05
2.00
0.94
1.71
8.84
10.0
9.5
9.0
8.5
8.0
7.5
7.0
6.5
6.0
5.5
5.0
4.5
4.0
3.5
3.0
2.5
2.0
1.5
1.0
0.5
0.0
f1 (ppm)

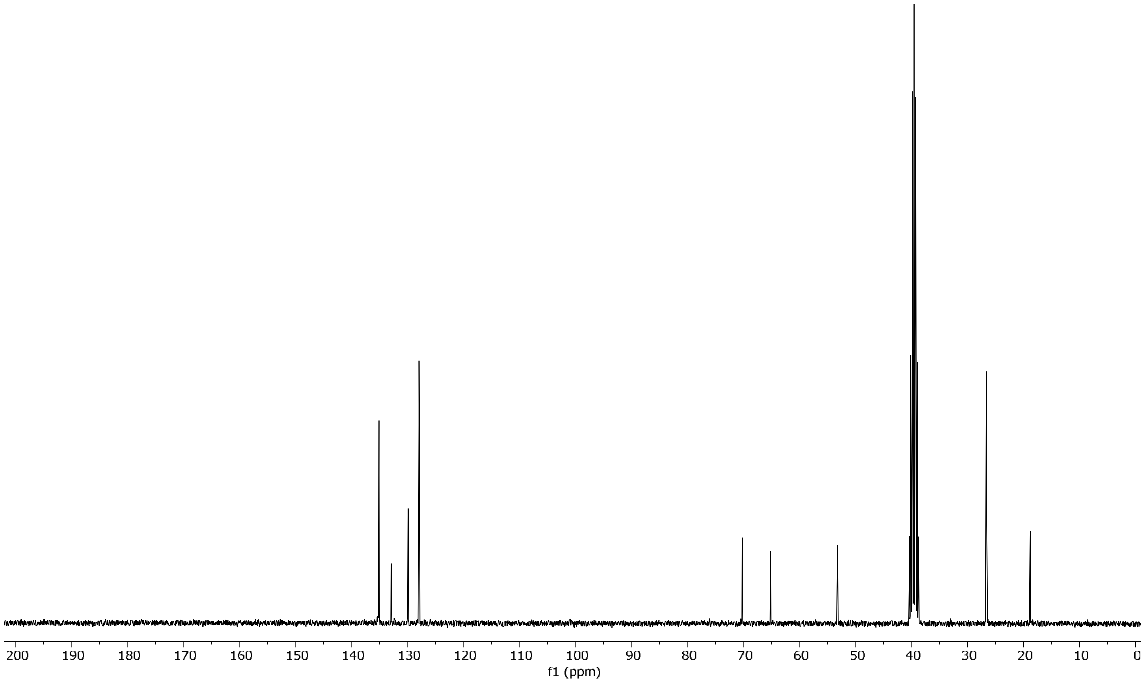
200
190
180
170
160
150
140
130
120
110
100
90
80
70
60
50
40
30
20
10
0
f1 (ppm)

Compound **27**

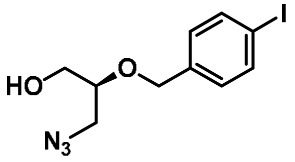

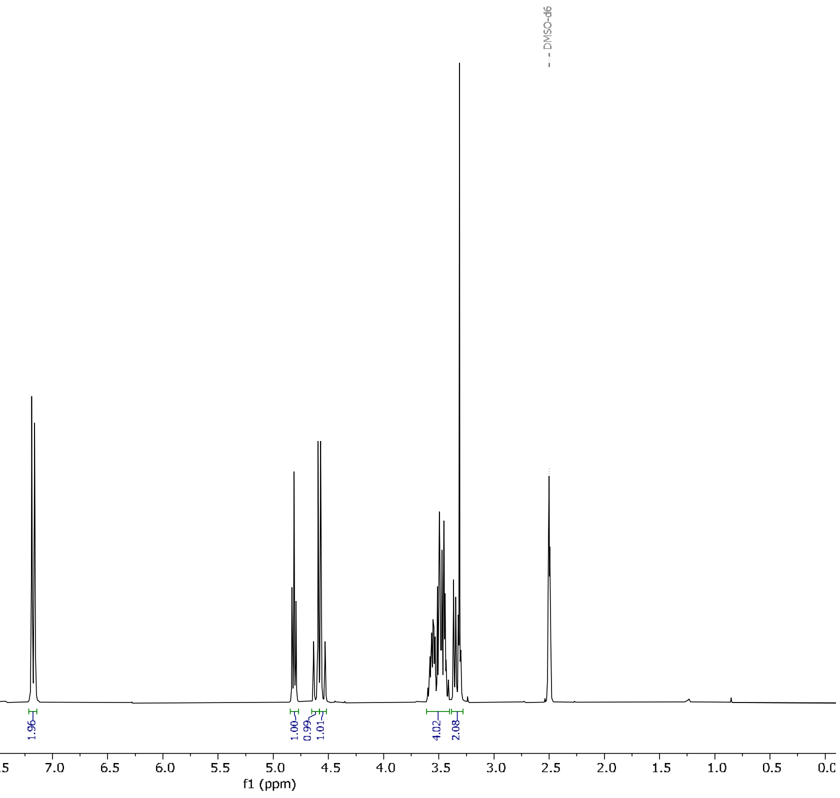
DMSO-d6
1.96
1.96
1.00
0.99
1.01
4.02
2.08
10.0 9.5 9.0 8.5 8.0 7.5 7.0 6.5 6.0 5.5 5.0 4.5 4.0 3.5 3.0 2.5 2.0 1.5 1.0 0.5 0.0
f1 (ppm)

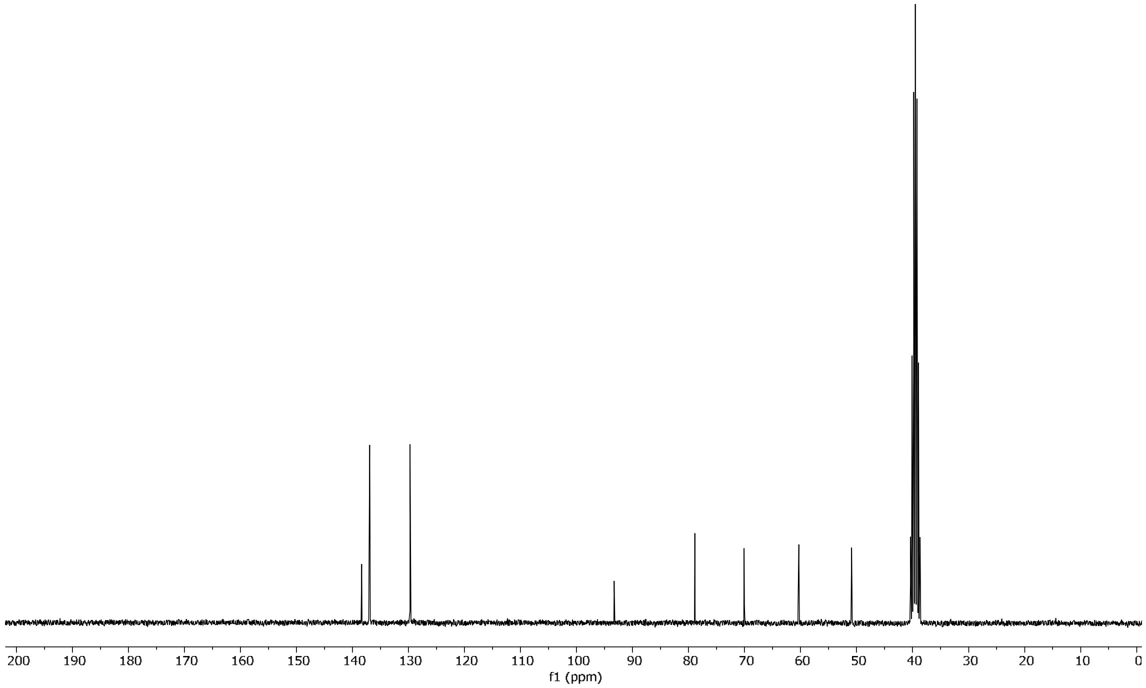
200 190 180 170 160 150 140 130 120 110 100 90 80 70 60 50 40 30 20 10 0
f1 (ppm)

Compound **29**

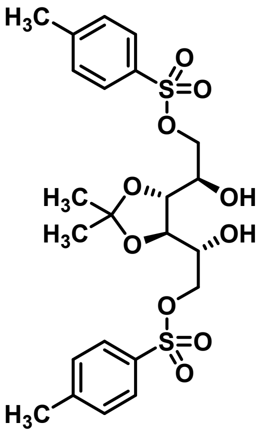
H3C
O
S=O
O
O
OH
H3C
H3C
O
OH
O
S=O
O
H3C

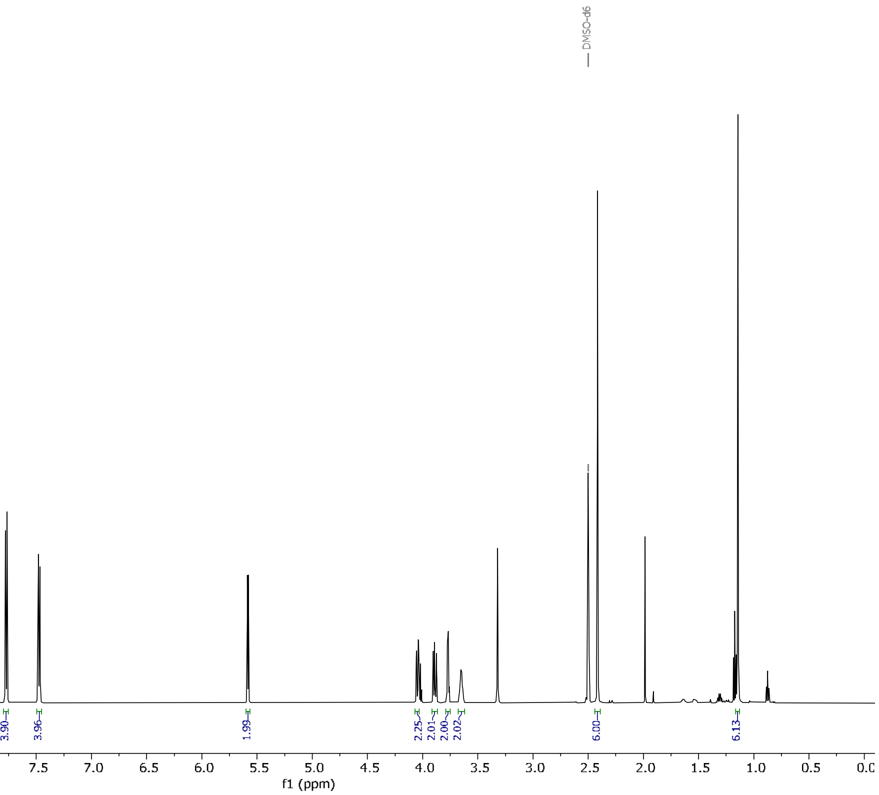
DMSO-d6
3.90
3.96
1.99
2.25
2.01
2.00
2.02
6.00
6.13
10.0 9.5 9.0 8.5 8.0 7.5 7.0 6.5 6.0 5.5 5.0 4.5 4.0 3.5 3.0 2.5 2.0 1.5 1.0 0.5 0.0
f1 (ppm)

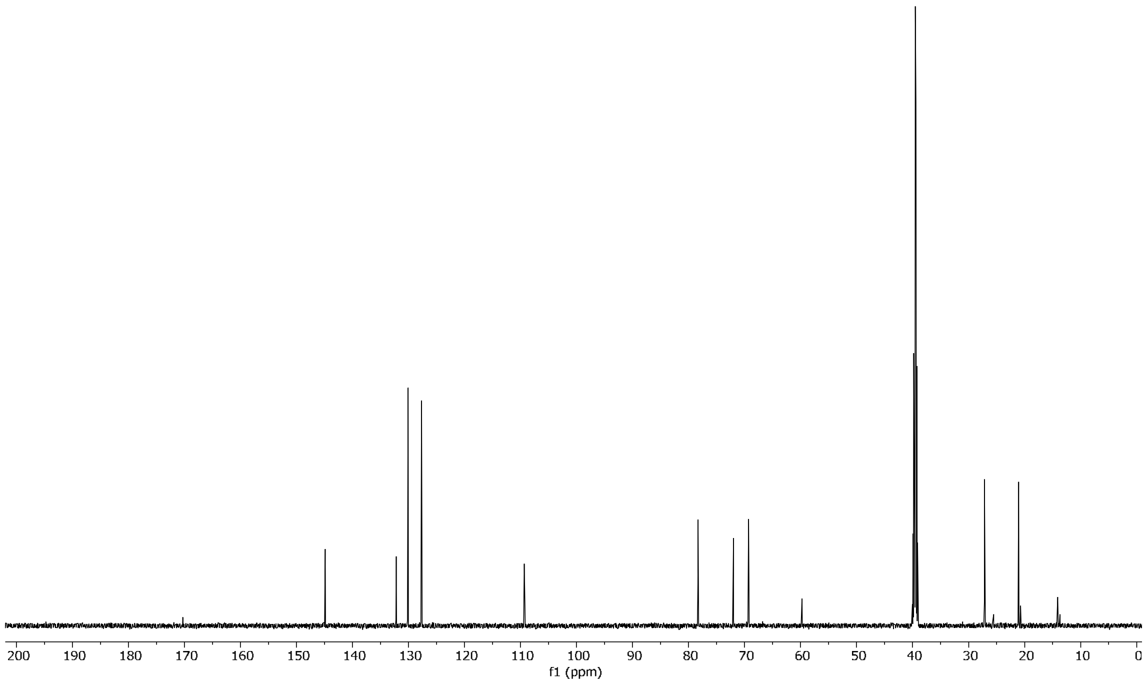
200 190 180 170 160 150 140 130 120 110 100 90 80 70 60 50 40 30 20 10 0
f1 (ppm)

Compound **30**

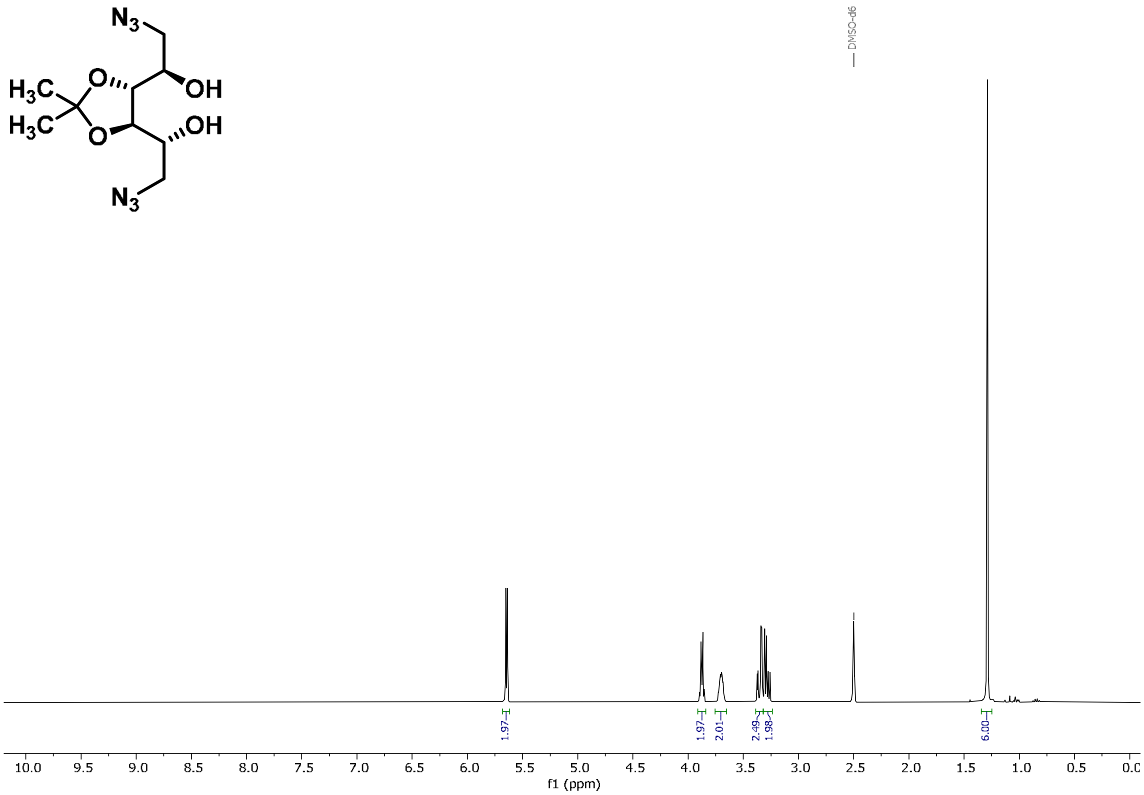

N3
H3C
O
OH
H3C
O
OH
N3
DMSO-d6
1.97
1.97
2.01
2.49
1.98
6.00
10.0 9.5 9.0 8.5 8.0 7.5 7.0 6.5 6.0 5.5 5.0 4.5 4.0 3.5 3.0 2.5 2.0 1.5 1.0 0.5 0.0
f1 (ppm)

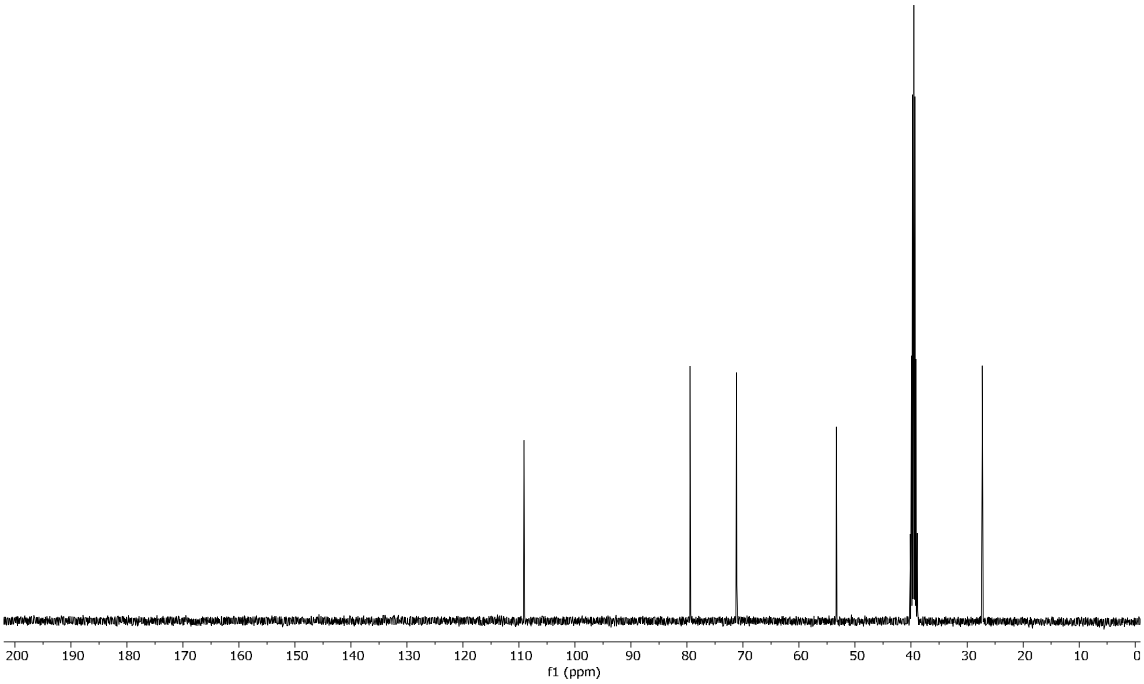

200 190 180 170 160 150 140 130 120 110 100 90 80 70 60 50 40 30 20 10 0
f1 (ppm)

Compound **31**

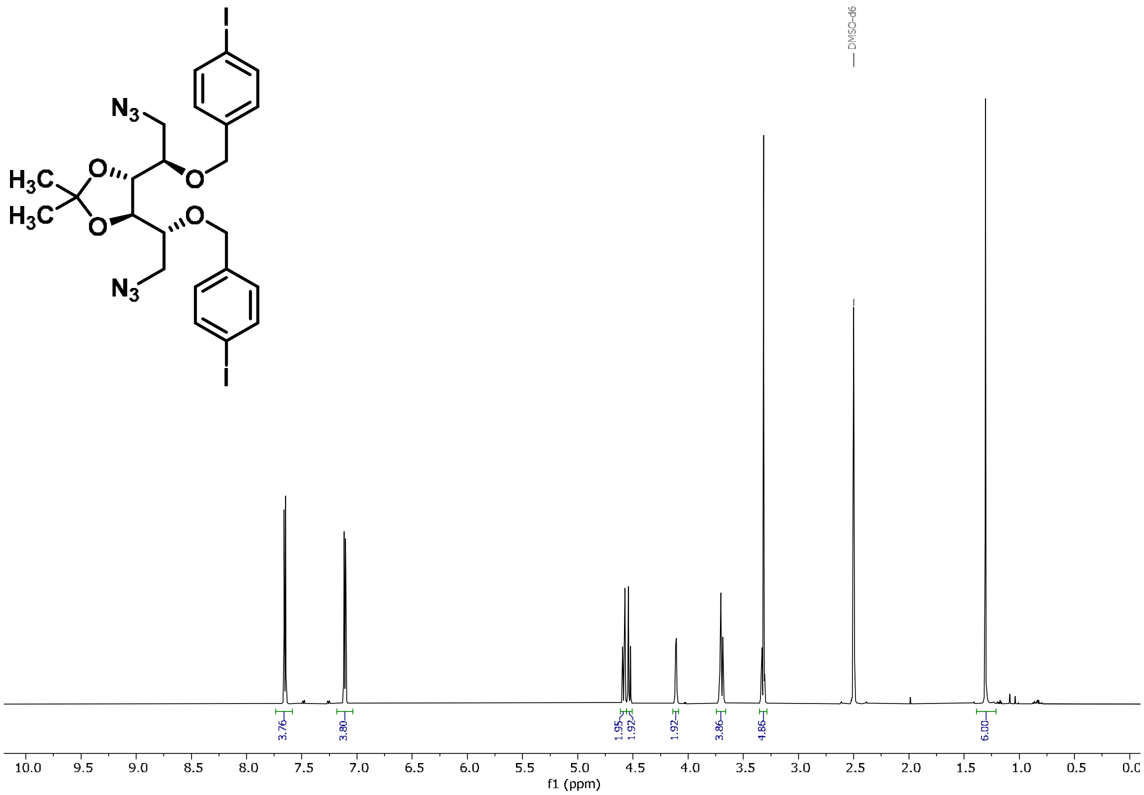

N3
H3C
H3C
O
I
DMSO-d6
3.76
3.80
1.95
1.92
1.92
3.86
4.86
6.00
10.0 9.5 9.0 8.5 8.0 7.5 7.0 6.5 6.0 5.5 5.0 4.5 4.0 3.5 3.0 2.5 2.0 1.5 1.0 0.5 0.0
f1 (ppm)

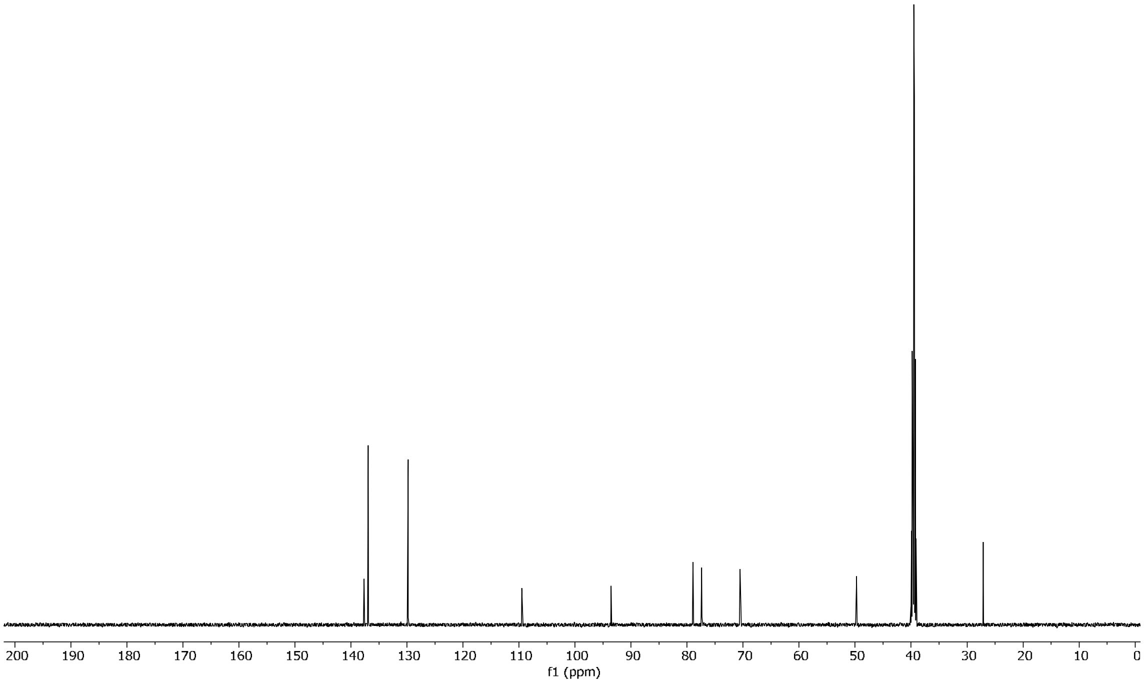

200 190 180 170 160 150 140 130 120 110 100 90 80 70 60 50 40 30 20 10 0
f1 (ppm)

Compound **32**

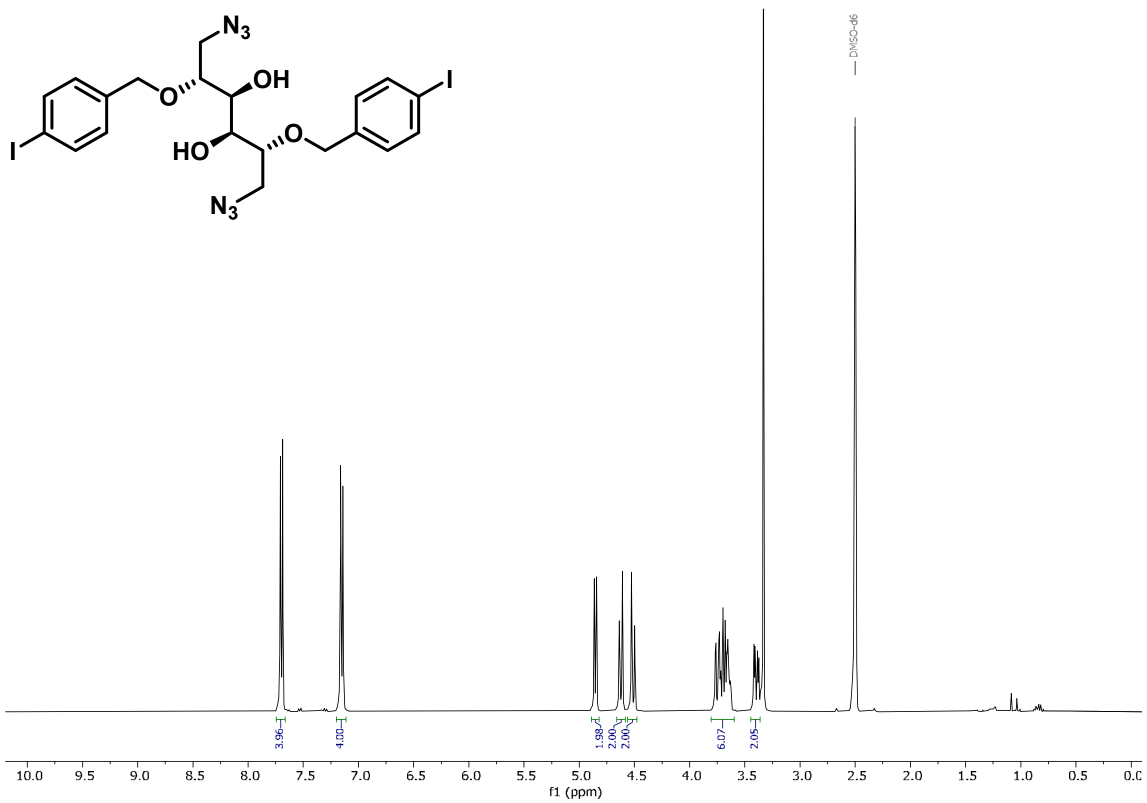

N3
OH
HO
O
O
I
I
N3
DMSO-d6
3.96
4.00
1.98
2.00
2.00
6.07
2.05
10.0 9.5 9.0 8.5 8.0 7.5 7.0 6.5 6.0 5.5 5.0 4.5 4.0 3.5 3.0 2.5 2.0 1.5 1.0 0.5 0.0
f1 (ppm)

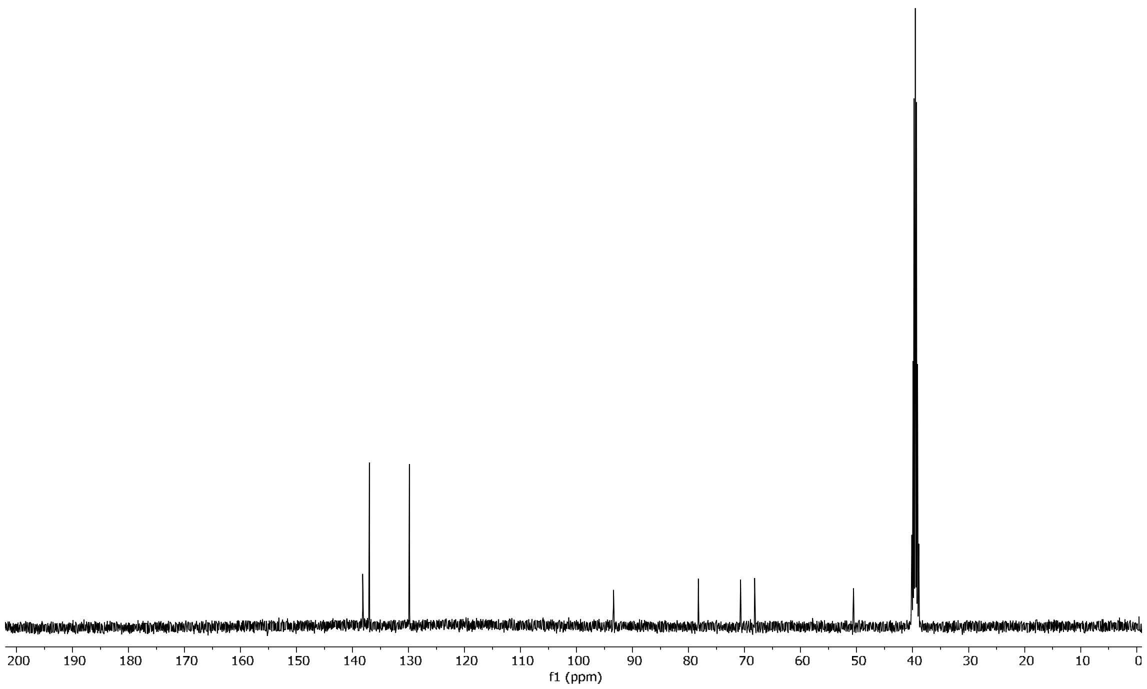

200 190 180 170 160 150 140 130 120 110 100 90 80 70 60 50 40 30 20 10 0
f1 (ppm)

Compound (*R*)-**28**

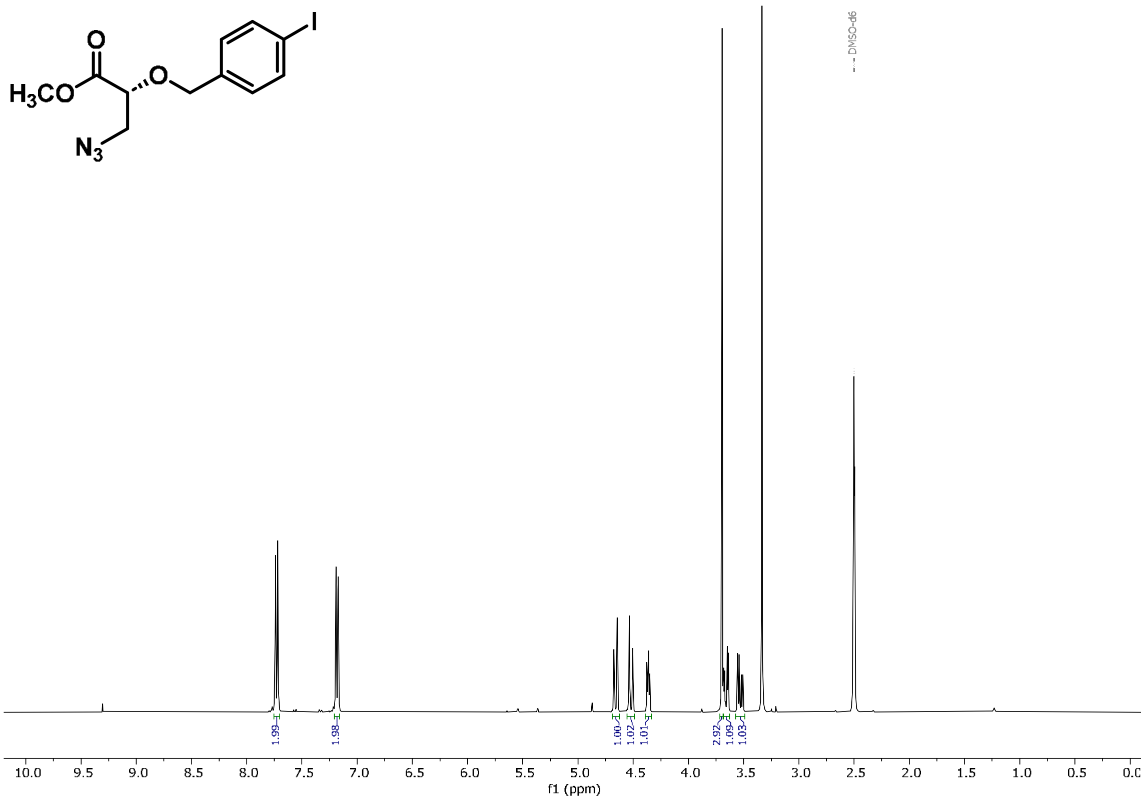

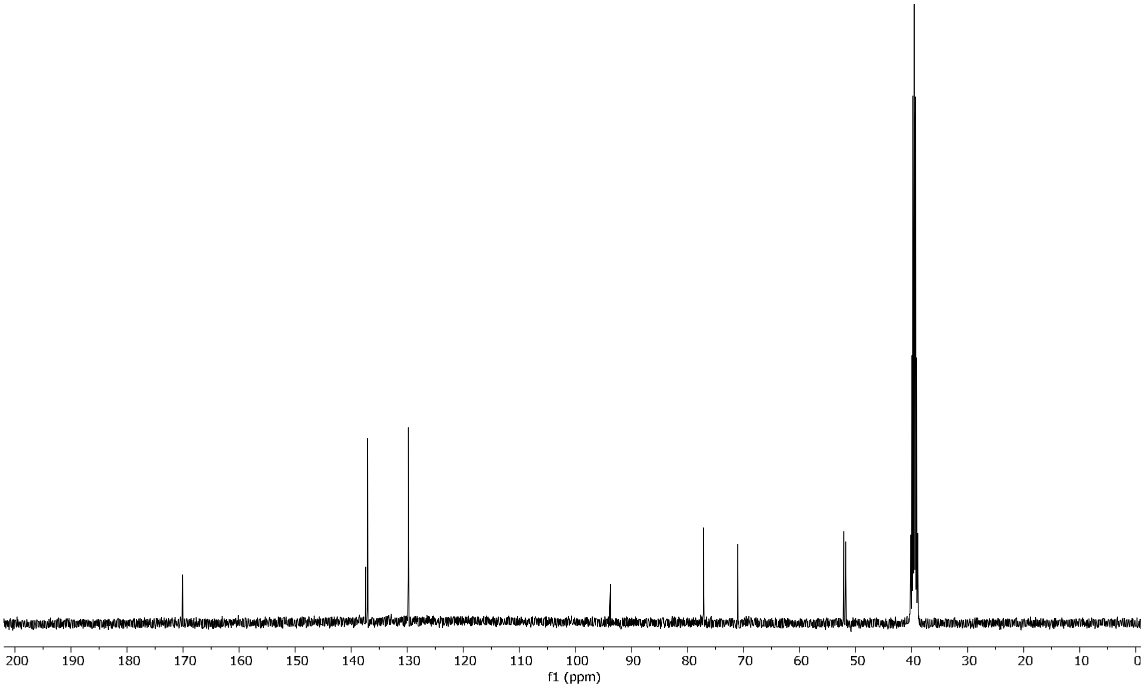

Compound (*R*)-**33**

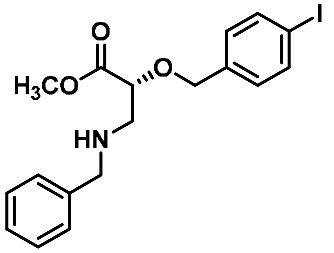

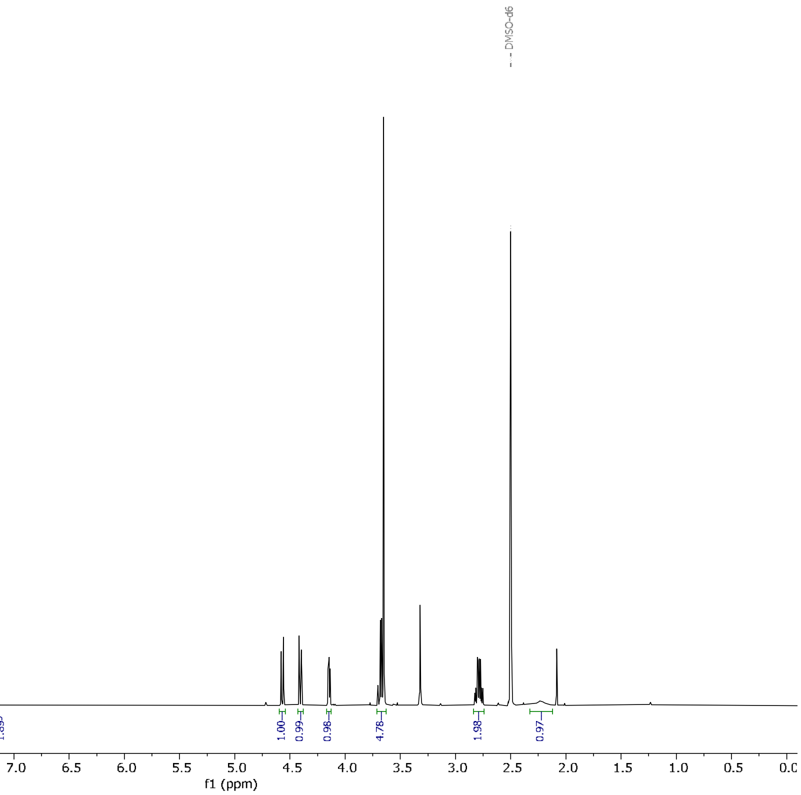

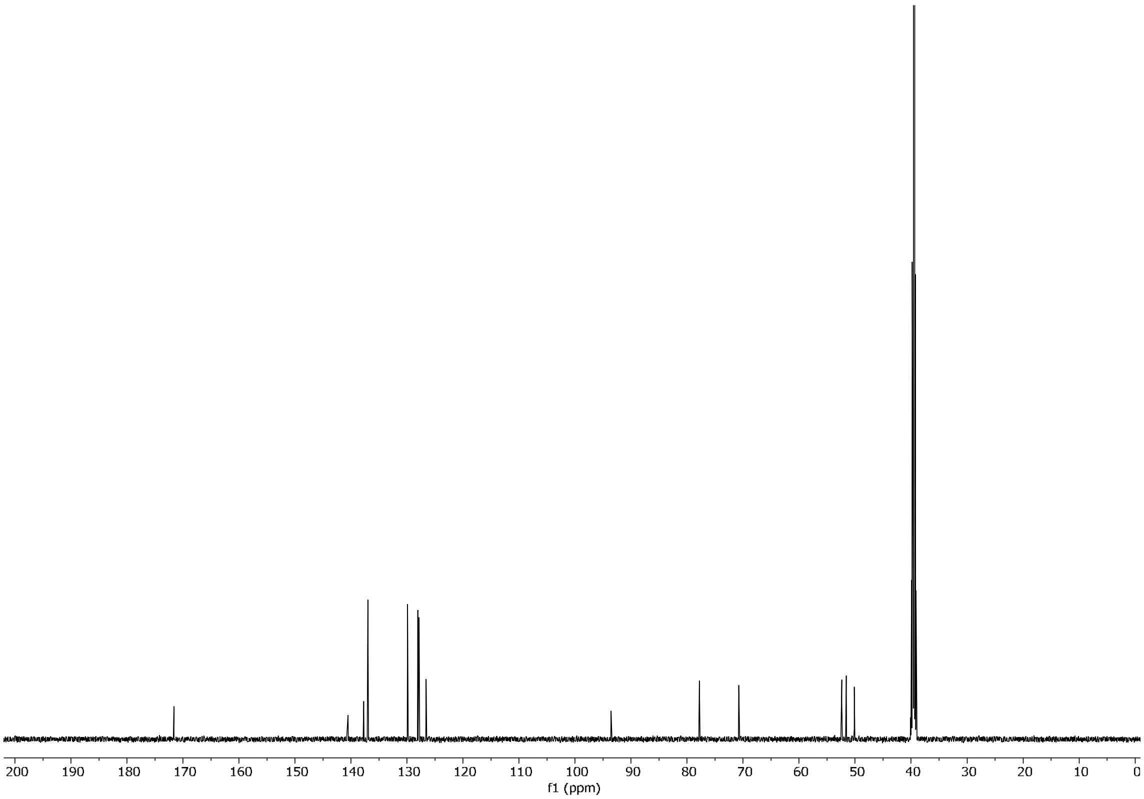

Compound (*R*)-**34**

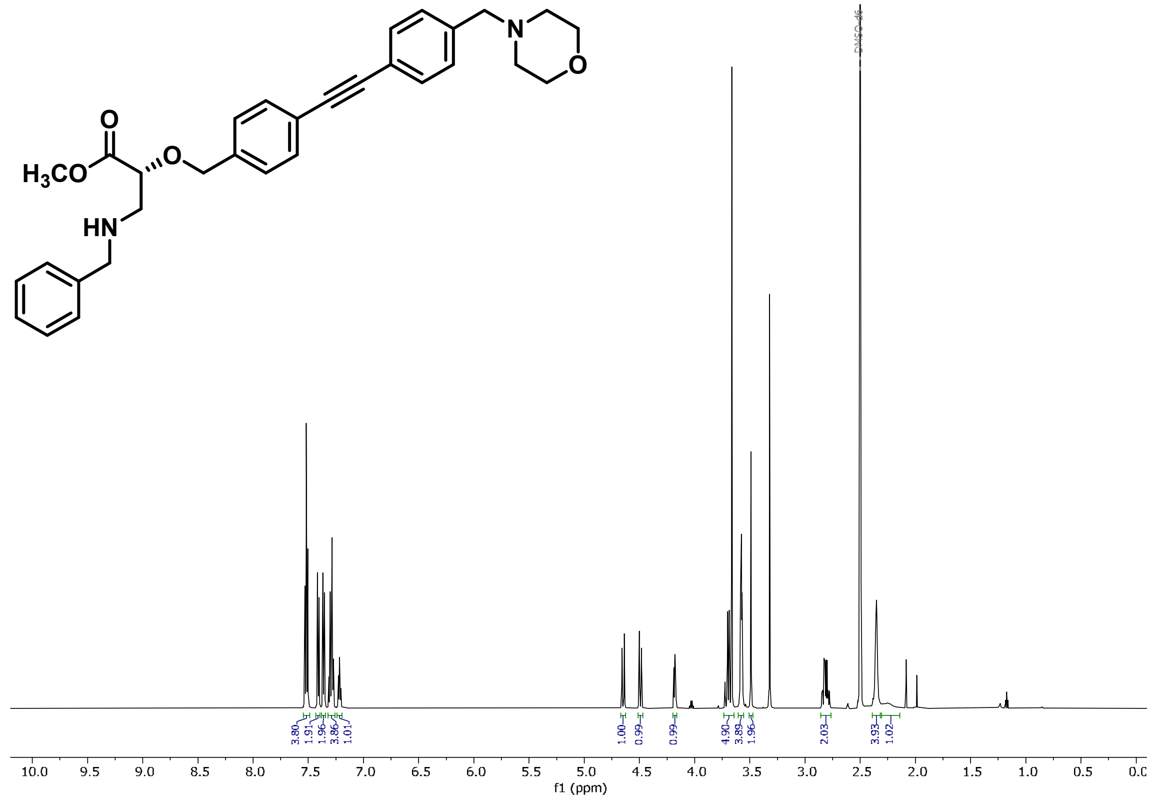

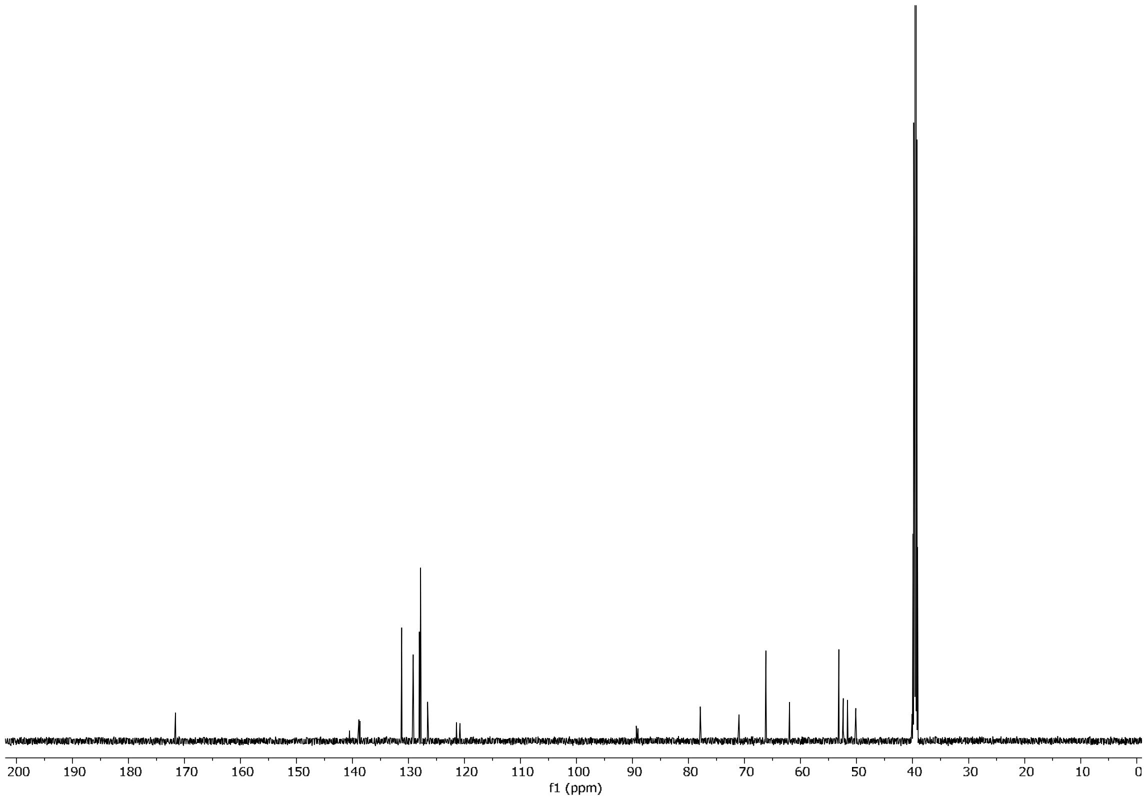

Compound (*S*)-**12a**

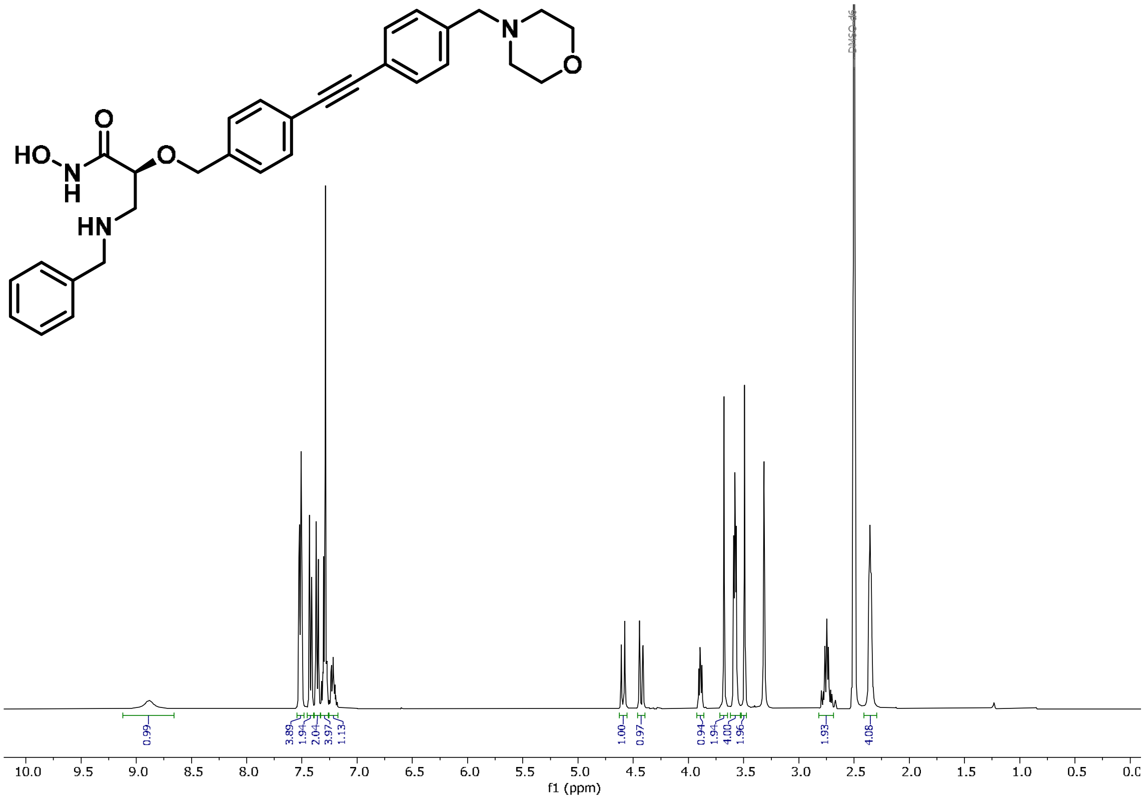

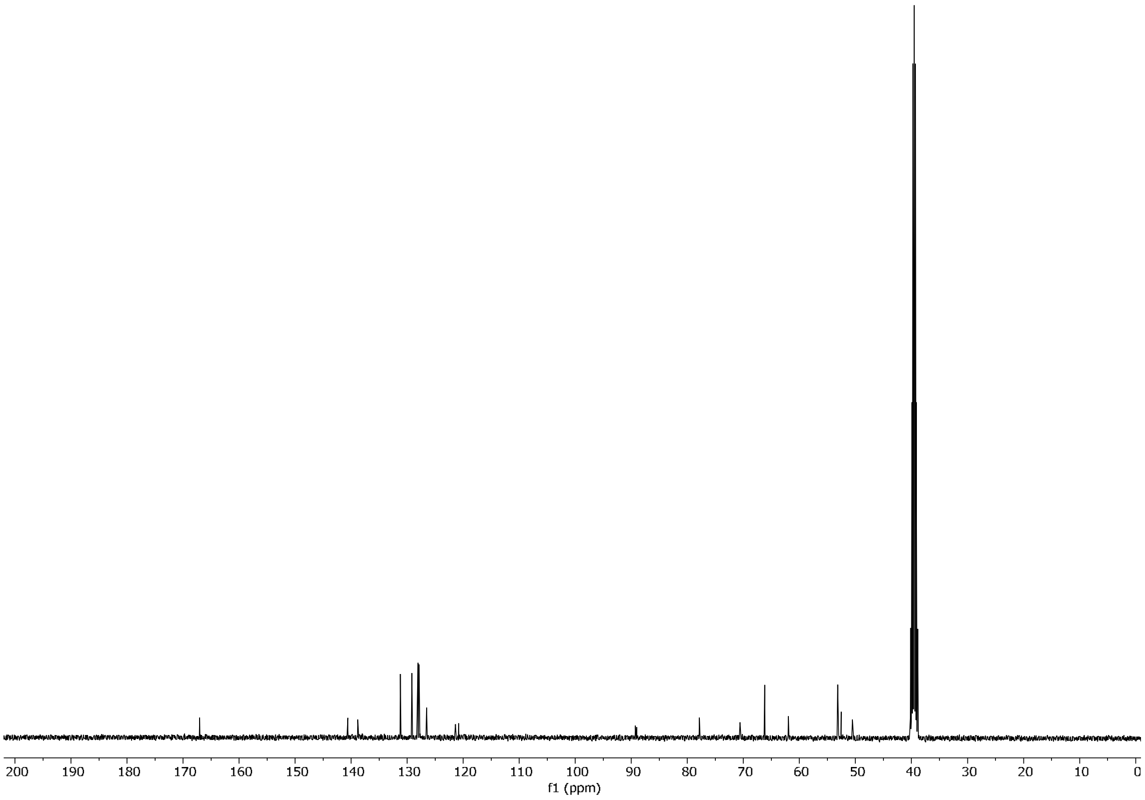

Compound (*R*)-**35**

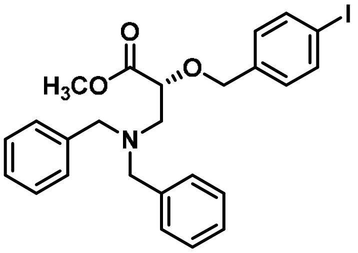

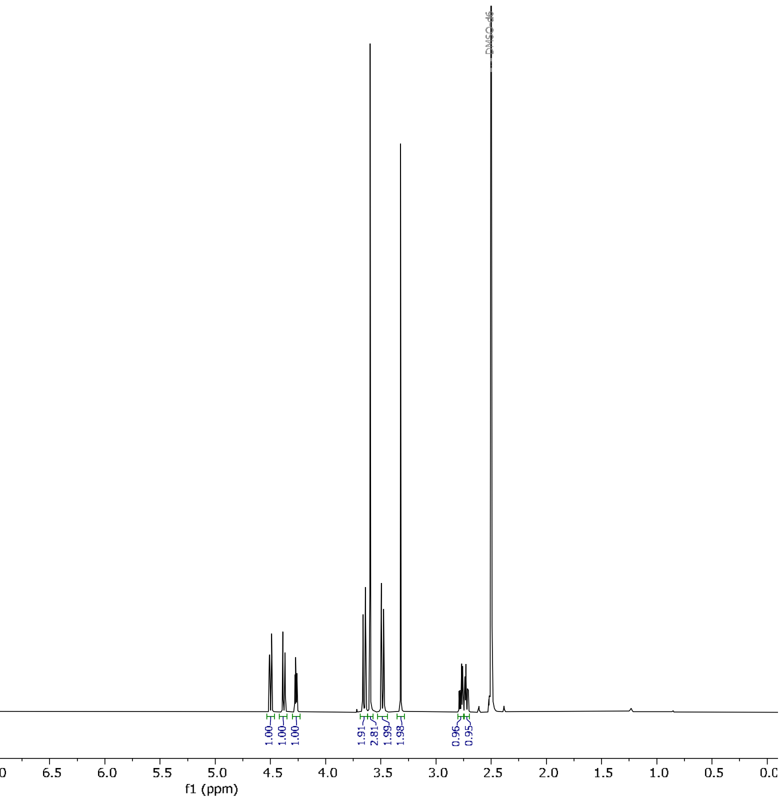

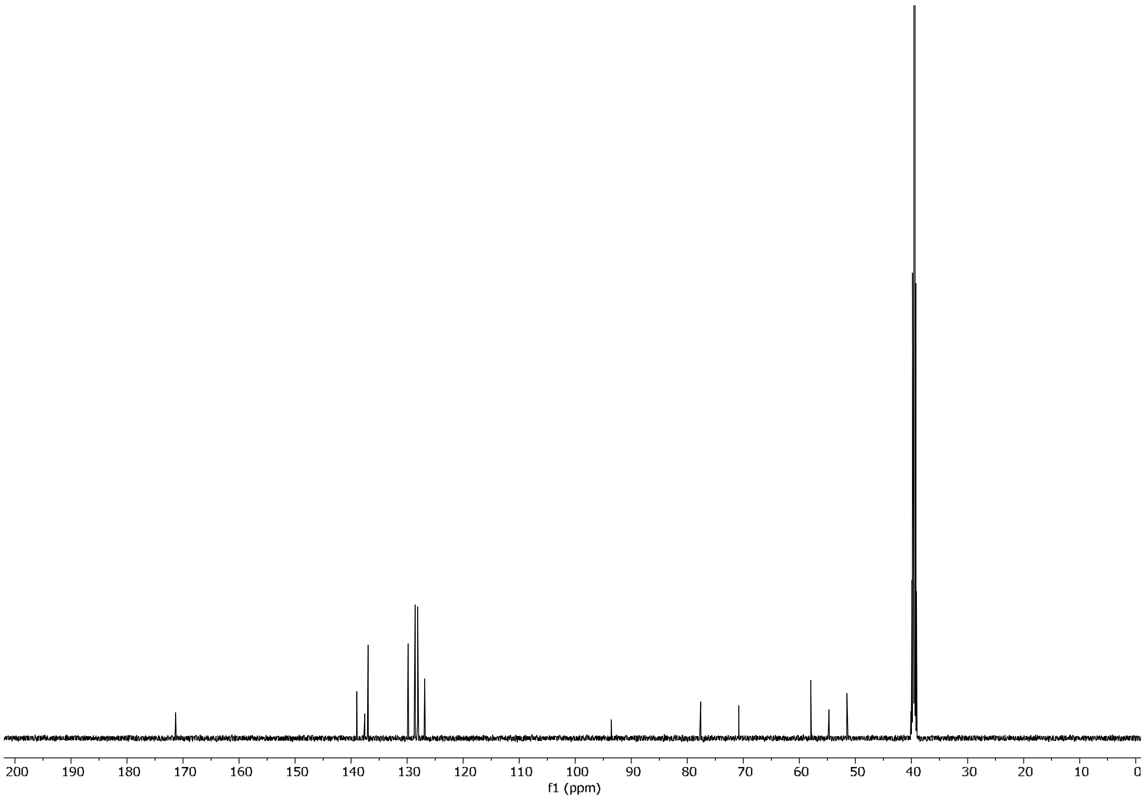

Compound (*R*)-**36**

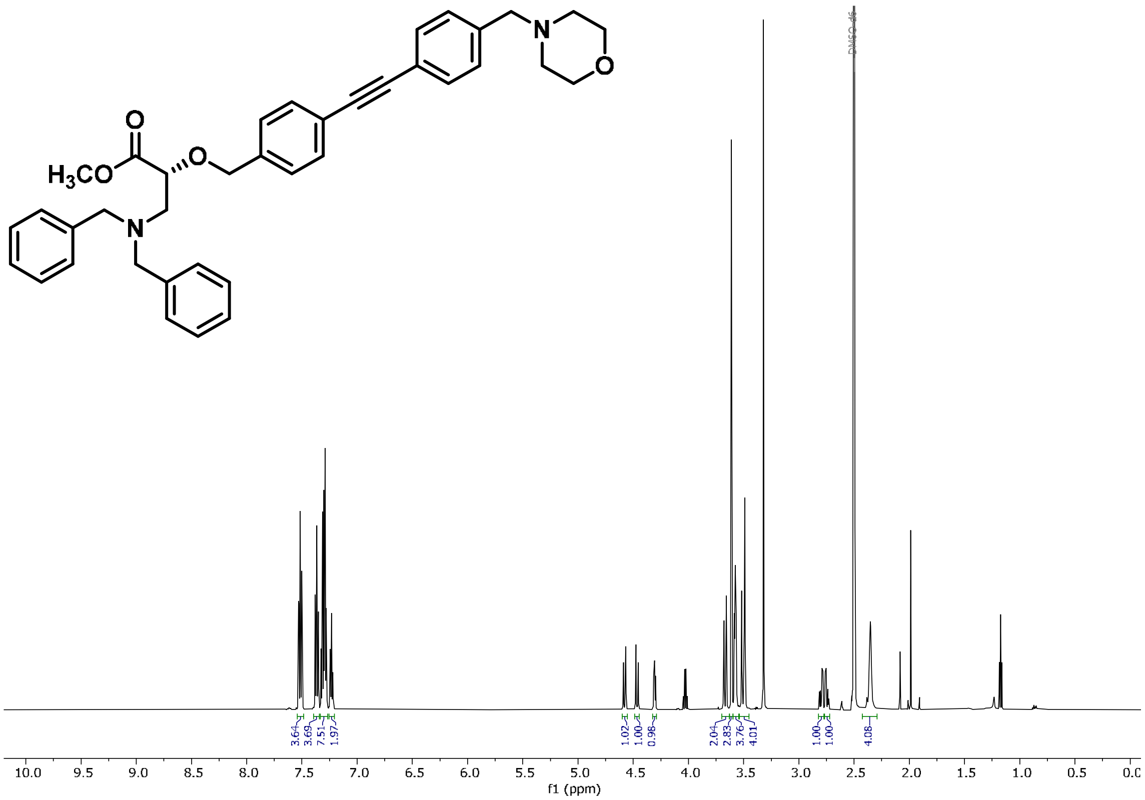

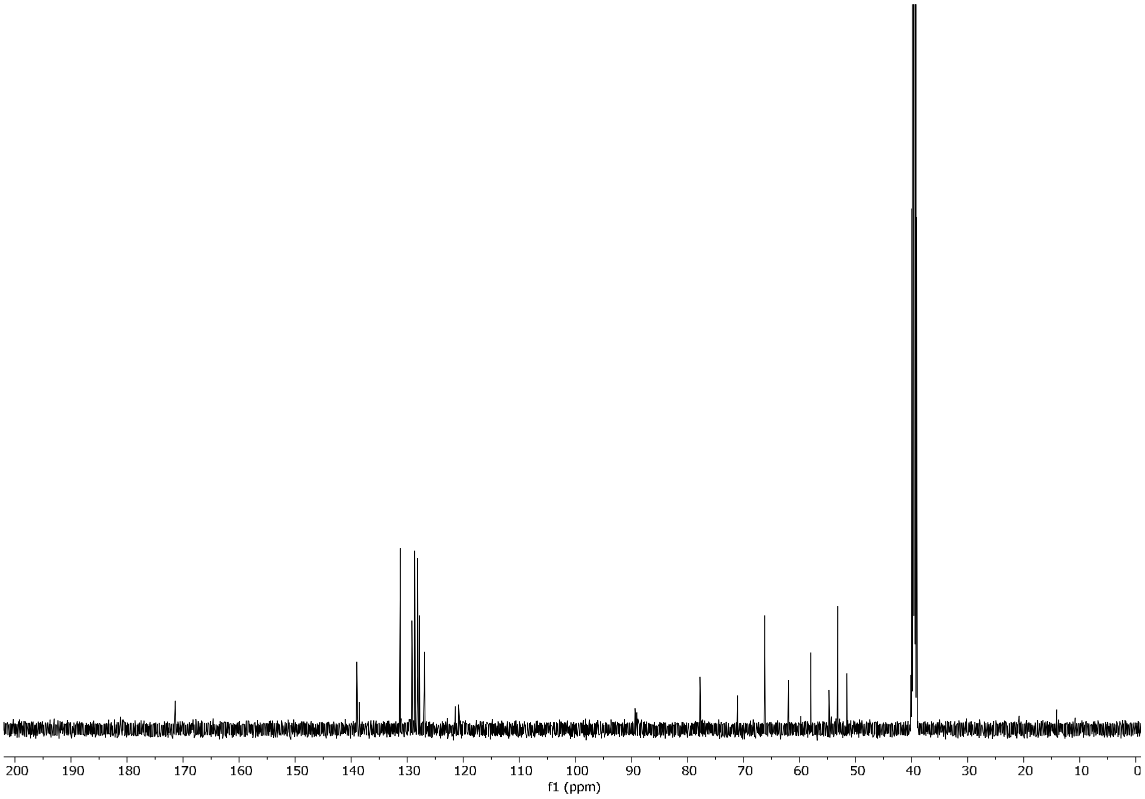

Compound (*S*)-**37**

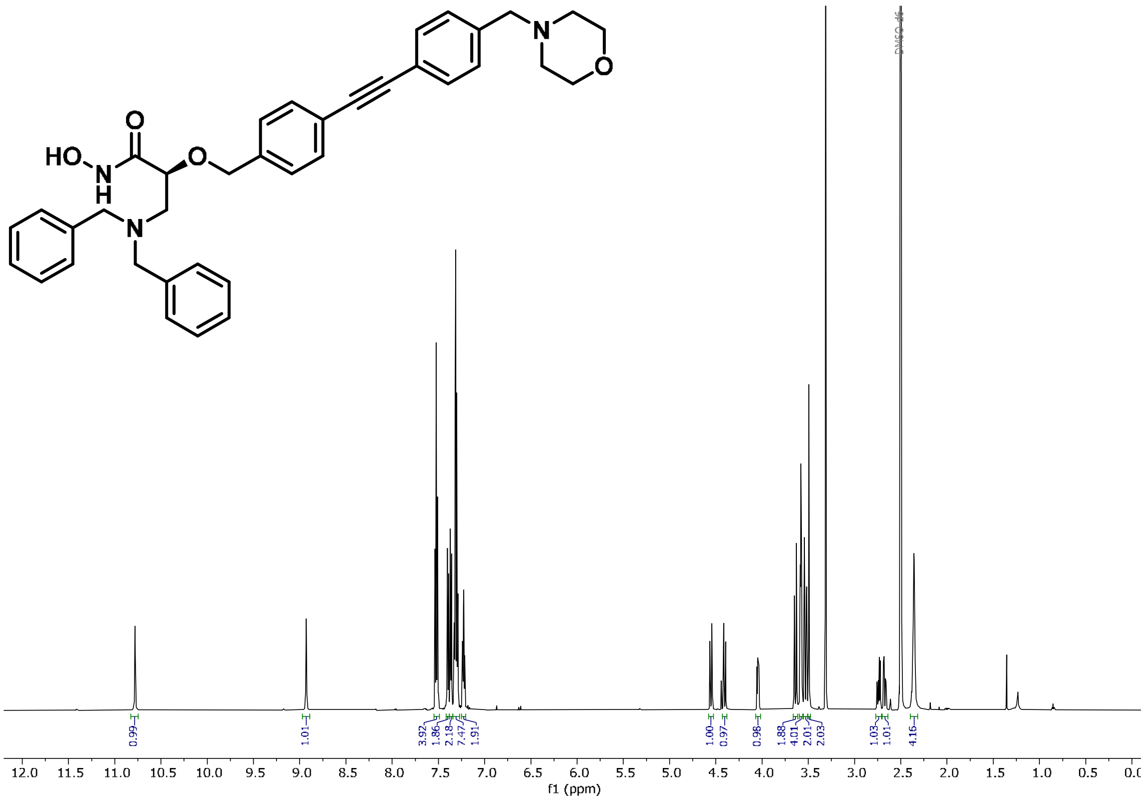

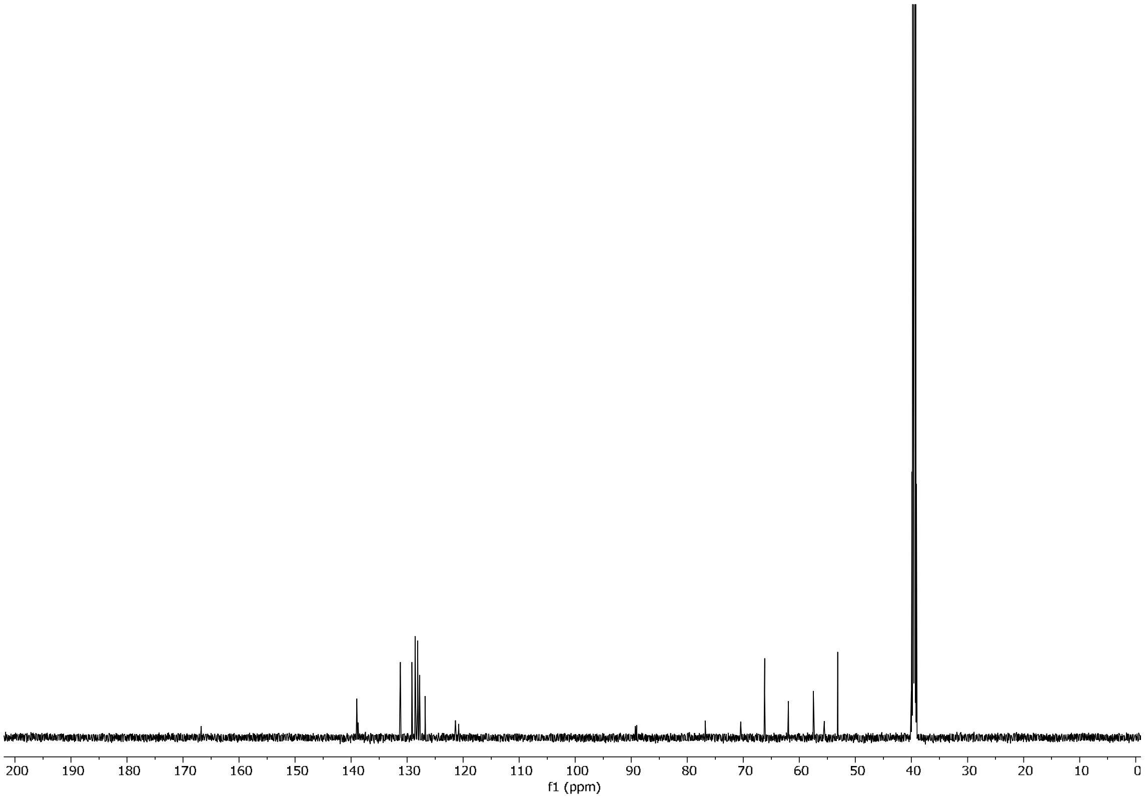

Compound (*S*)-**38a**

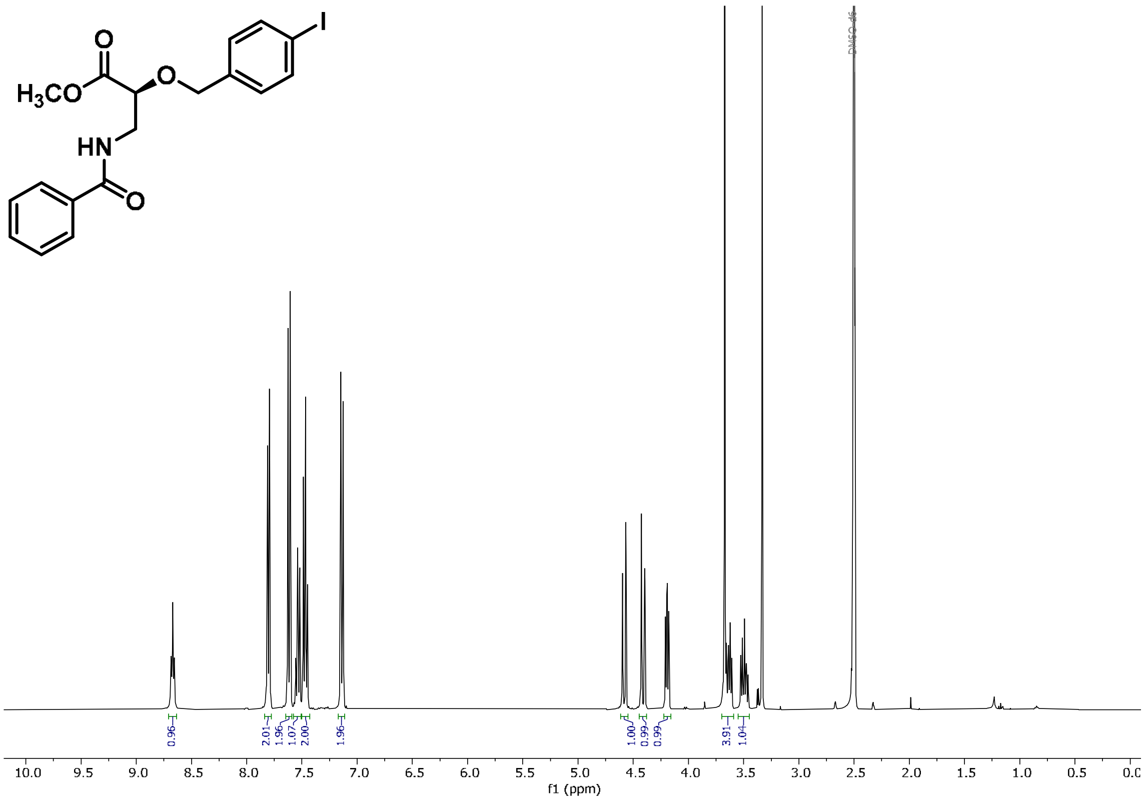

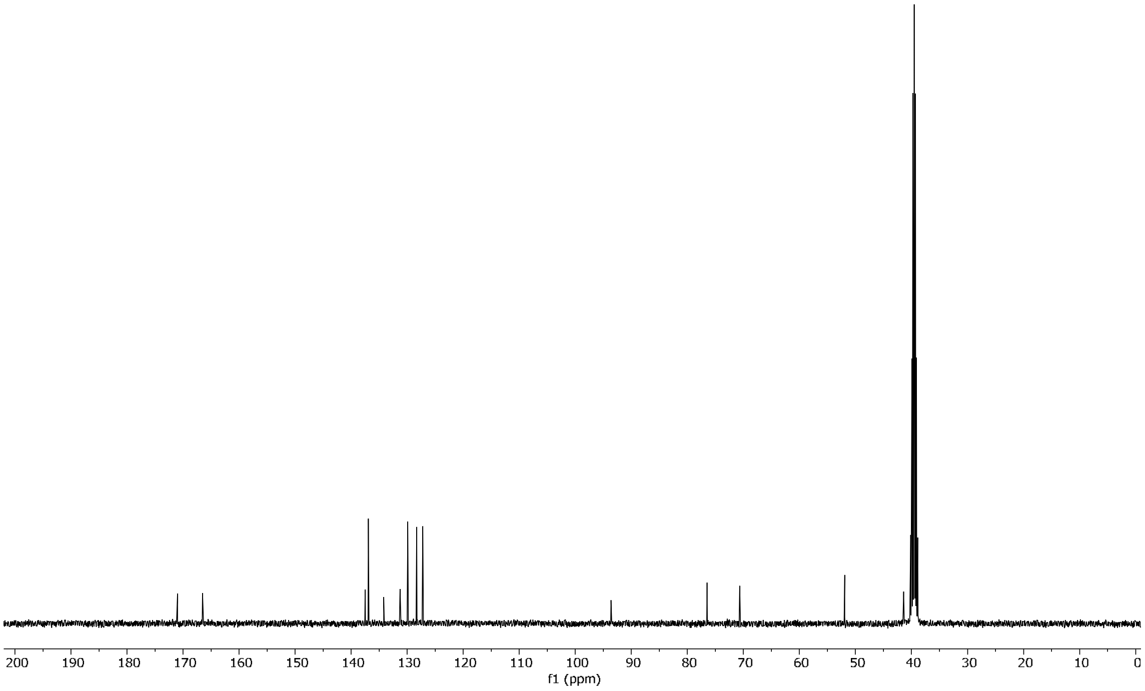

Compound (*S*)-**38b**

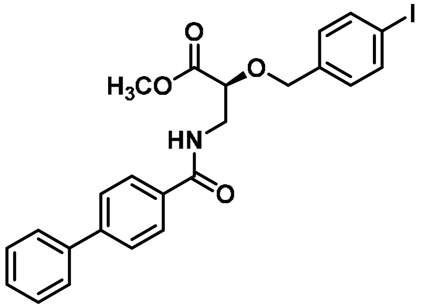

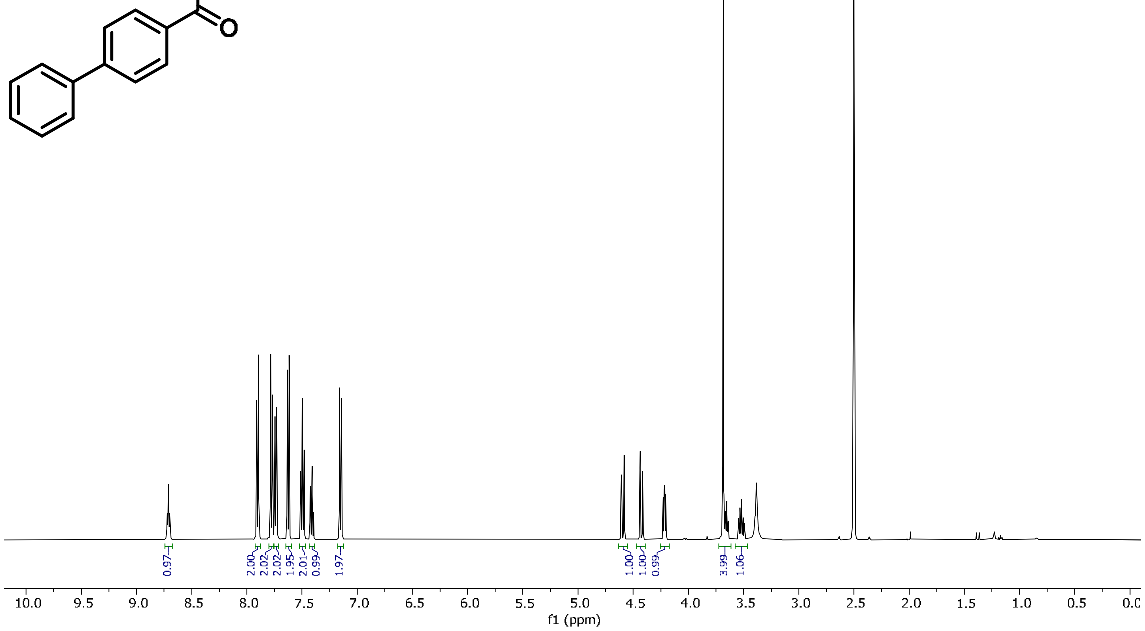

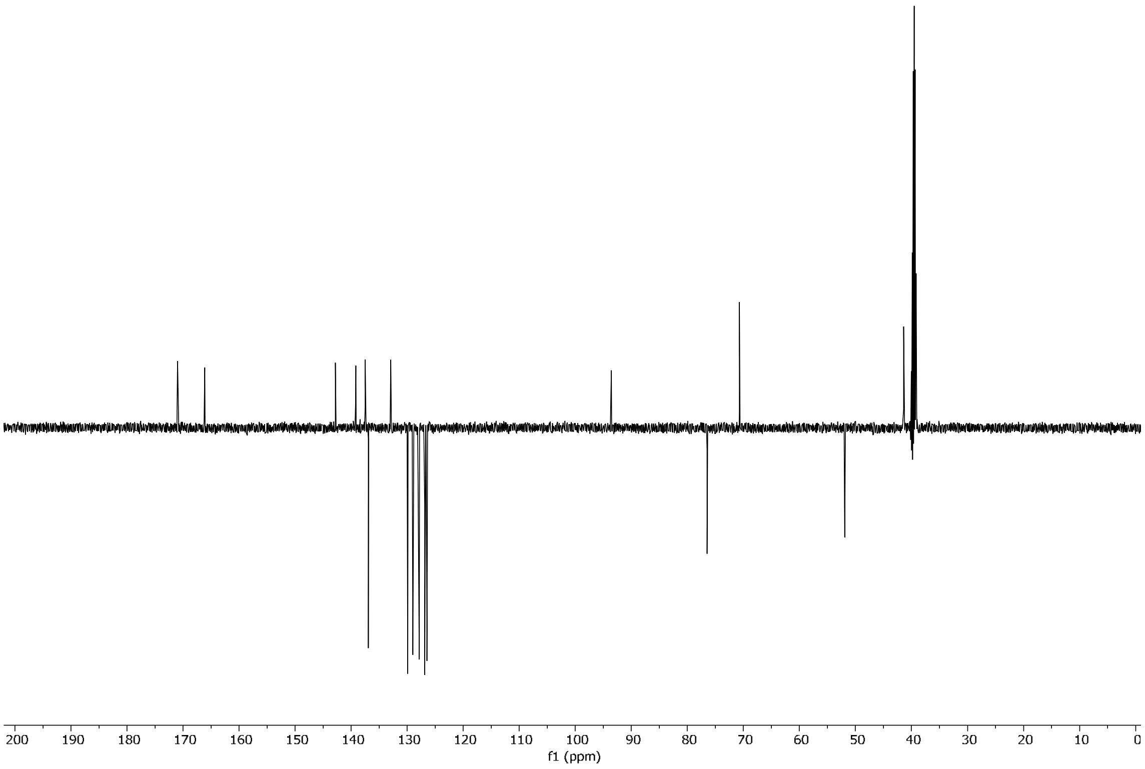

Compound (*R*)-**38c**

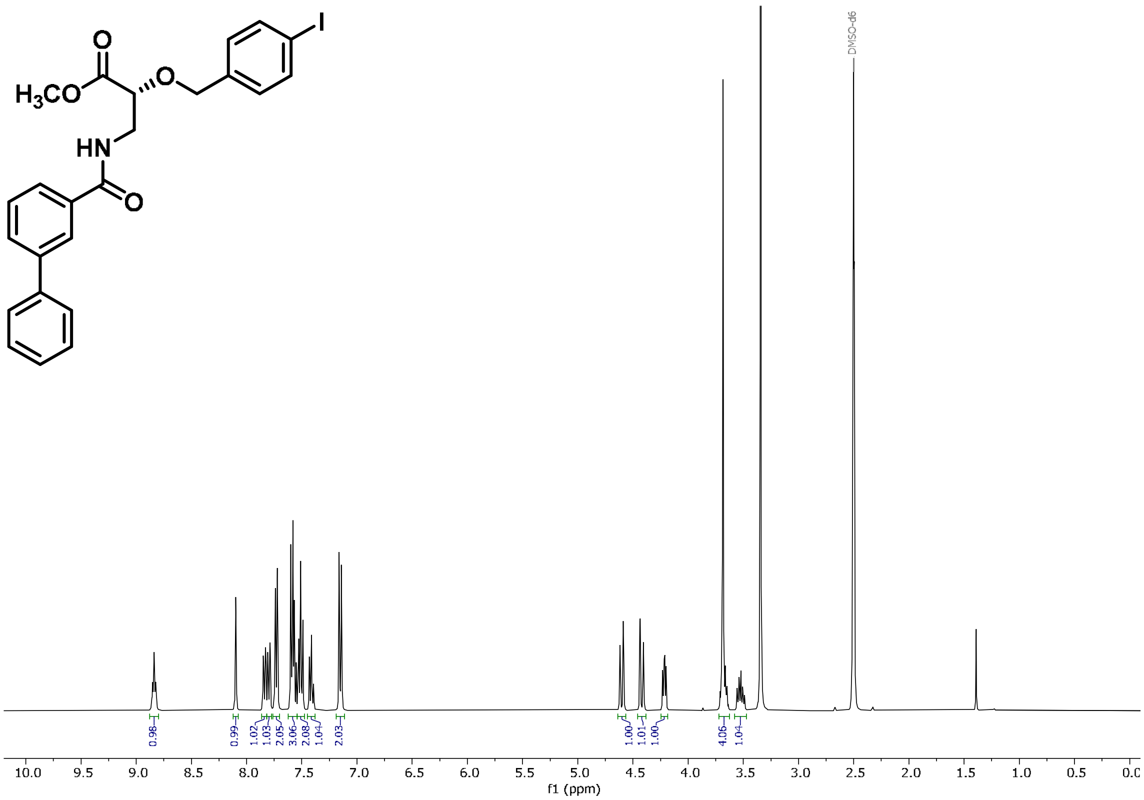

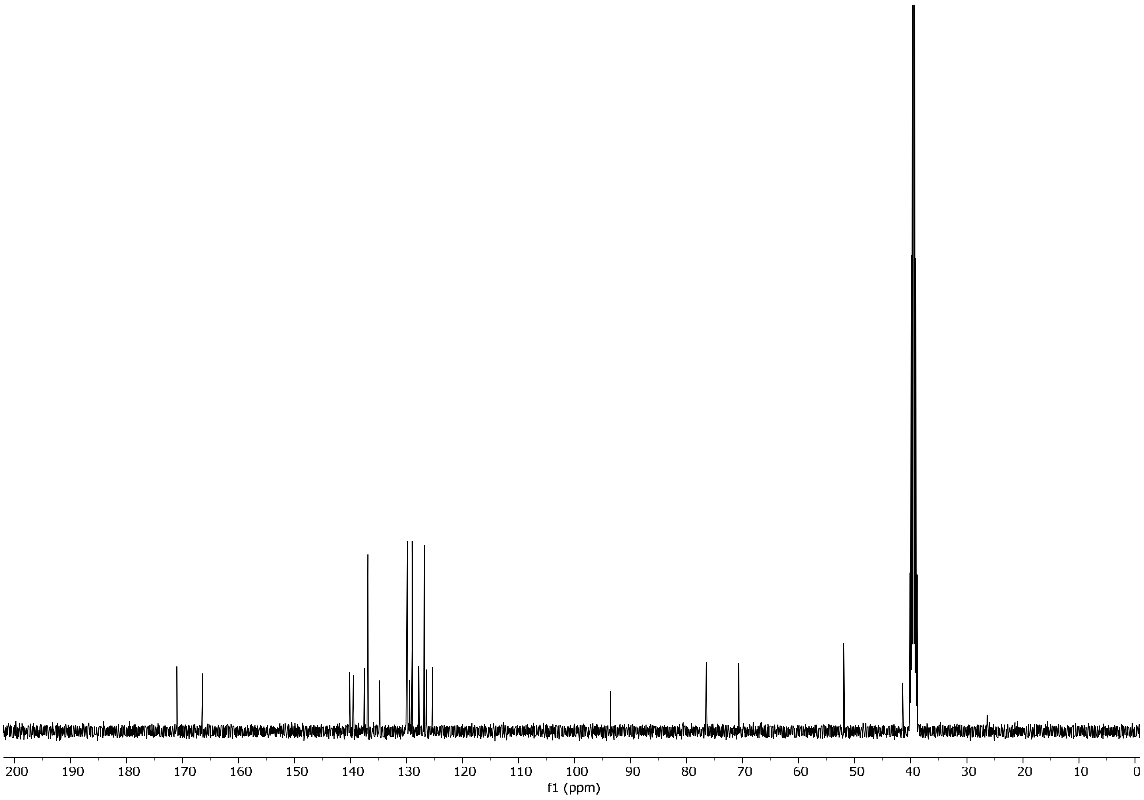

Compound (*S*)-**38d**

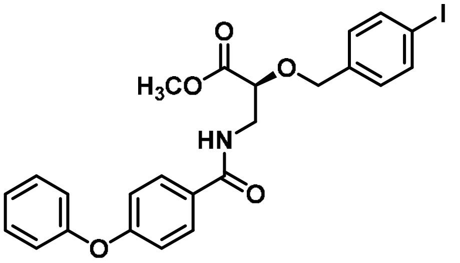

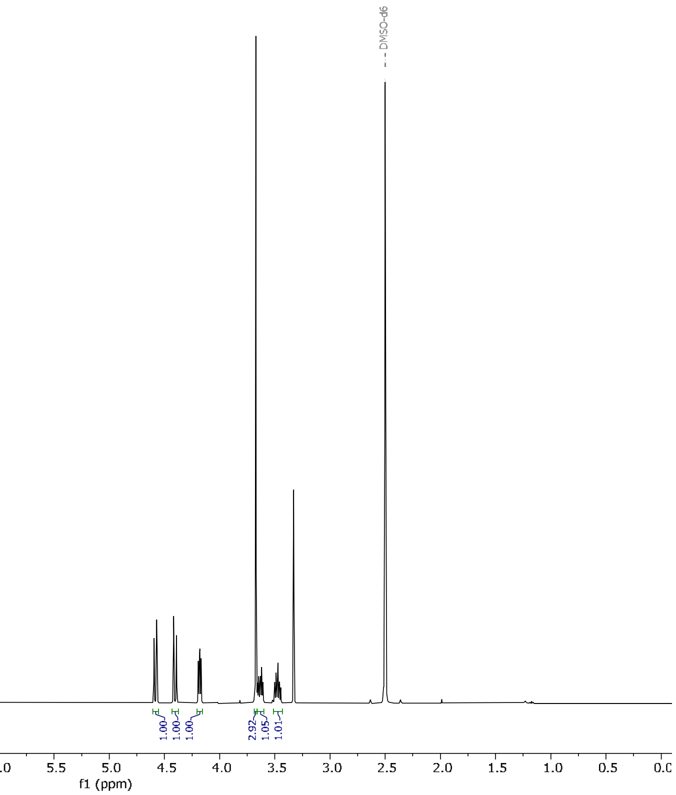

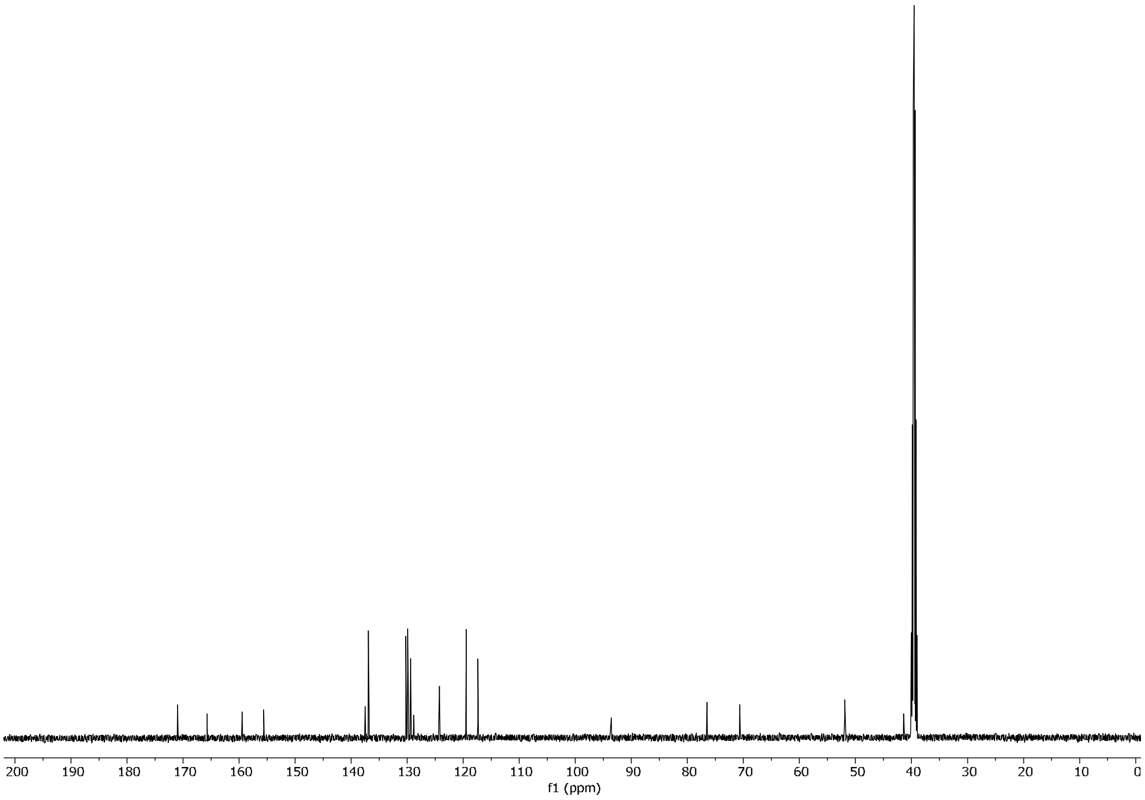

Compound (*R*)-**38e**

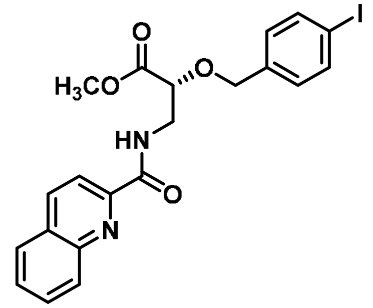

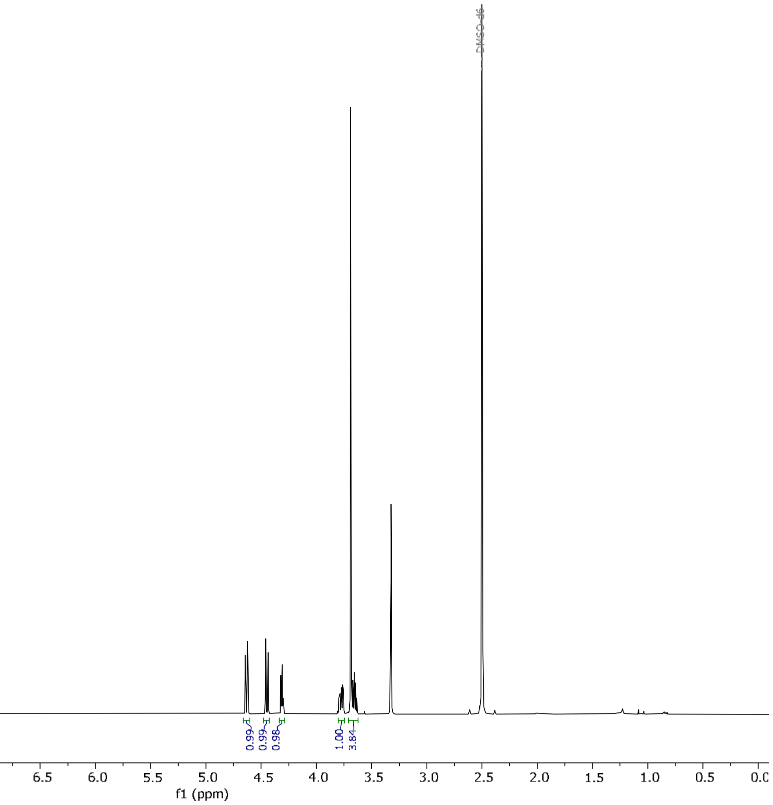

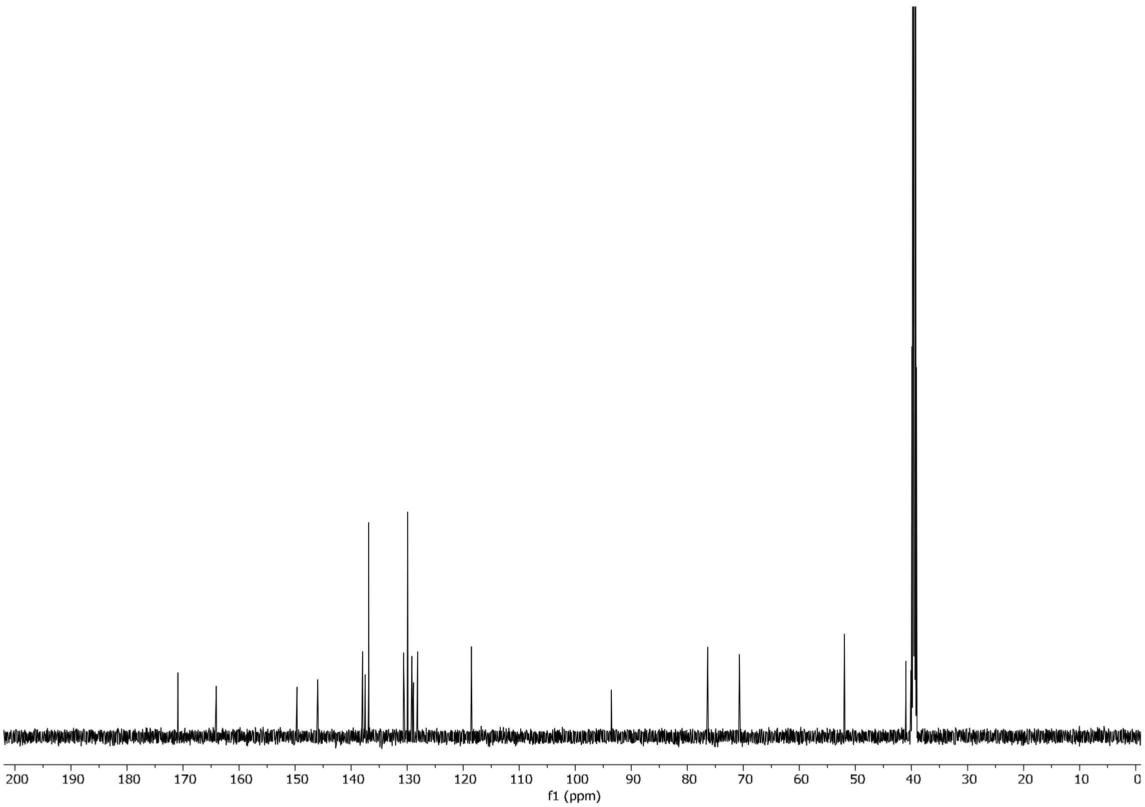

Compound (*R*)-**38f**

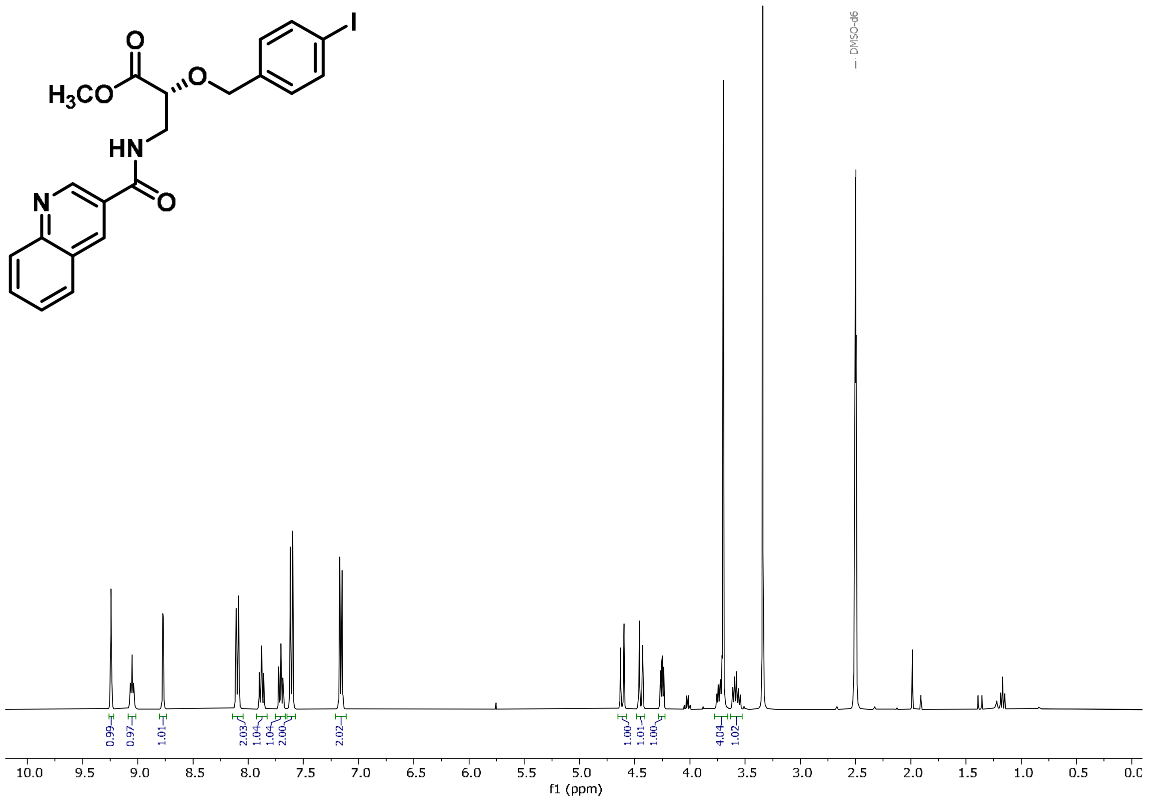

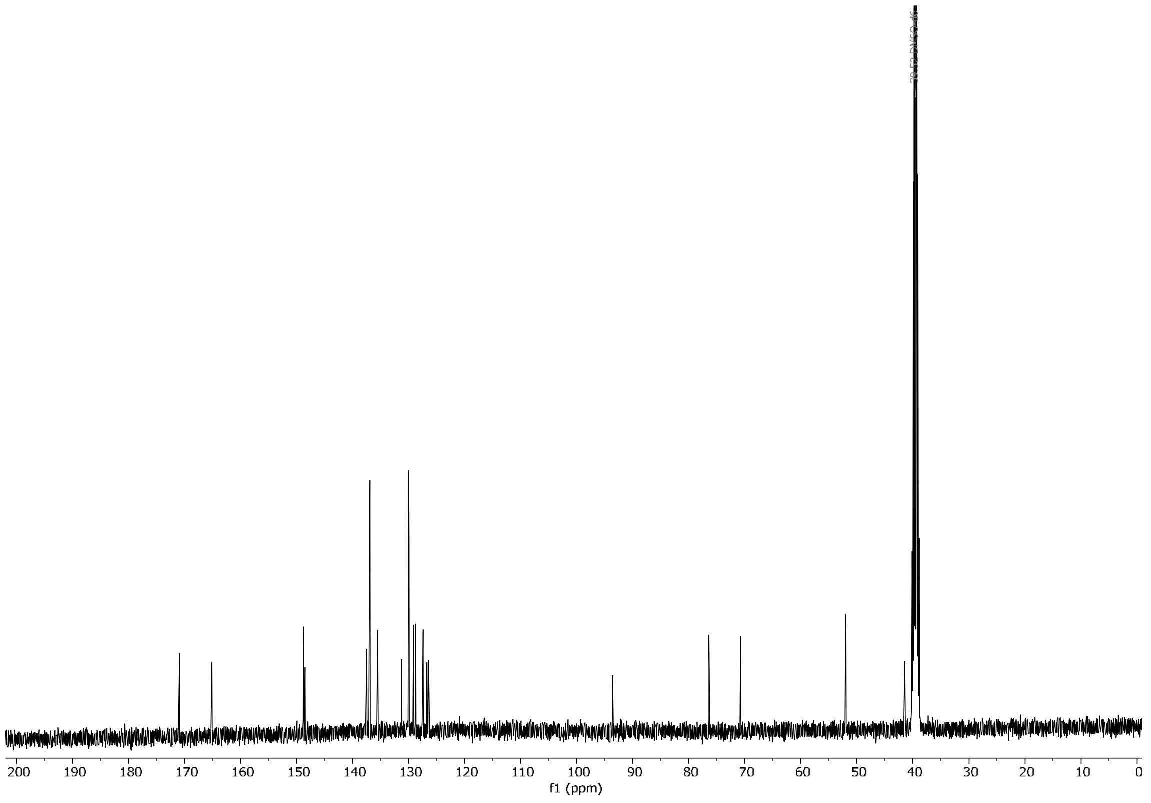

Compound (*R*)-**38g**

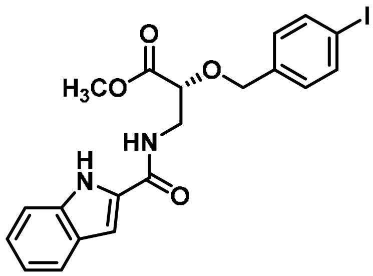

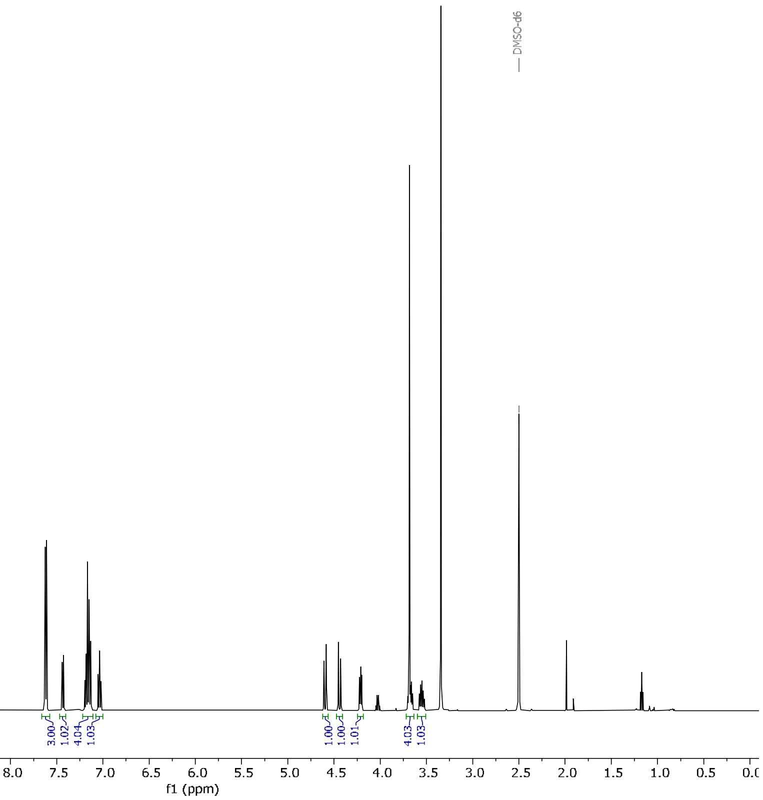

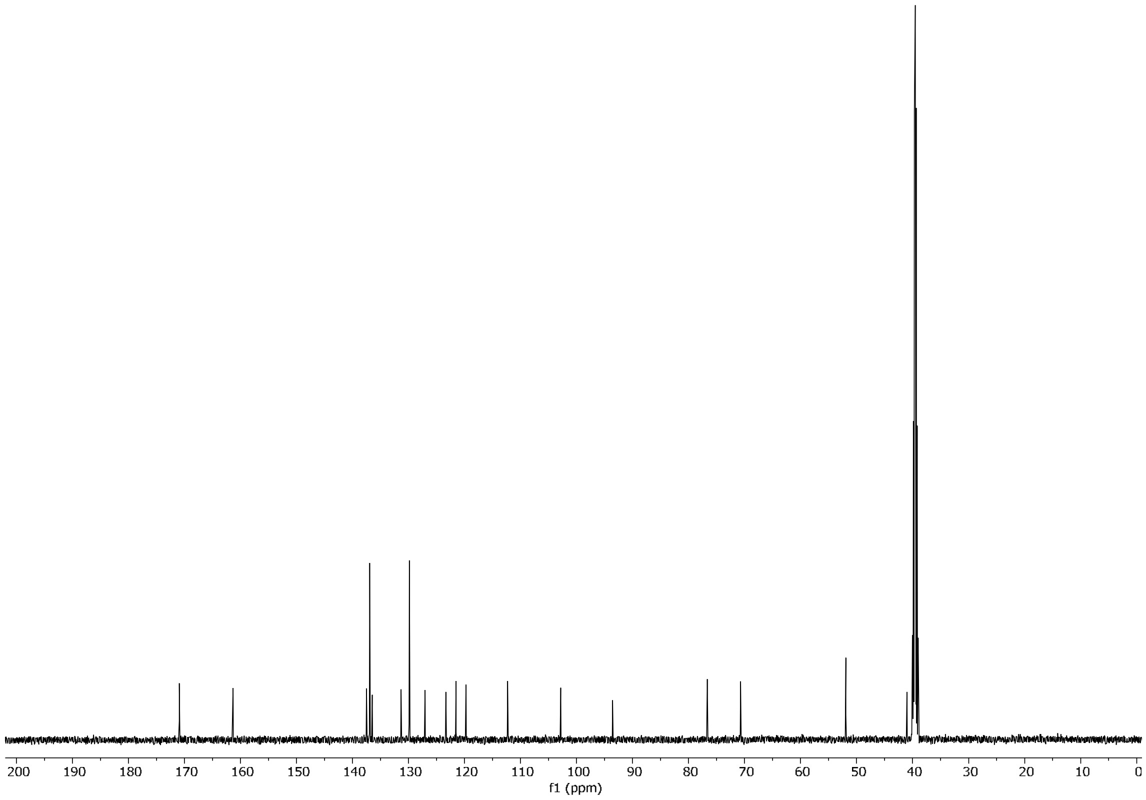

Compound (*S*)-**38h**

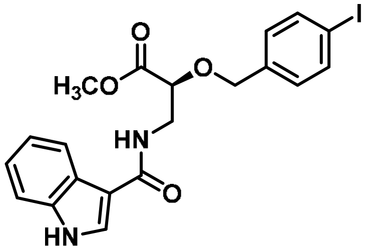

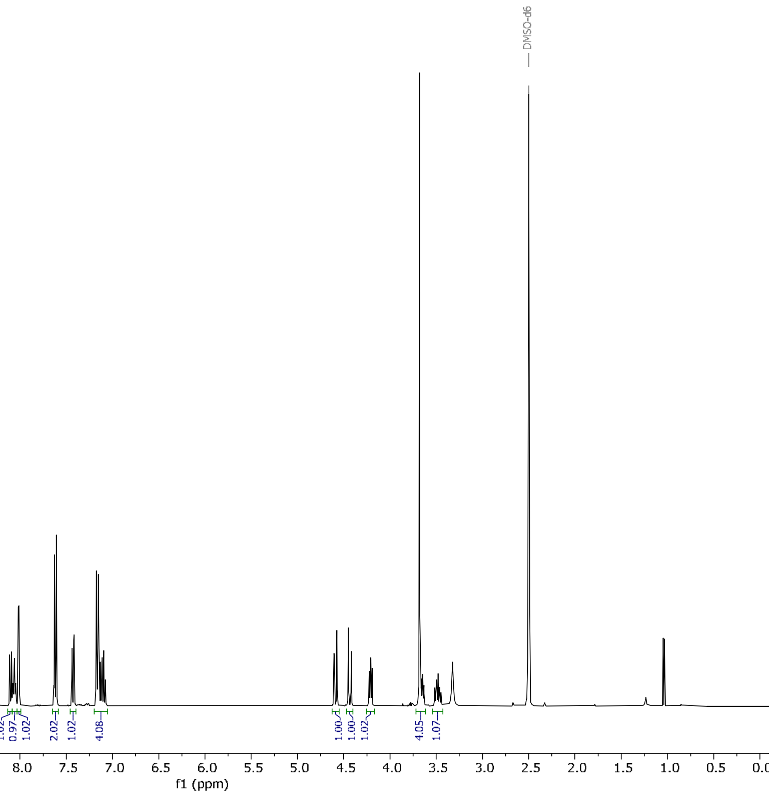

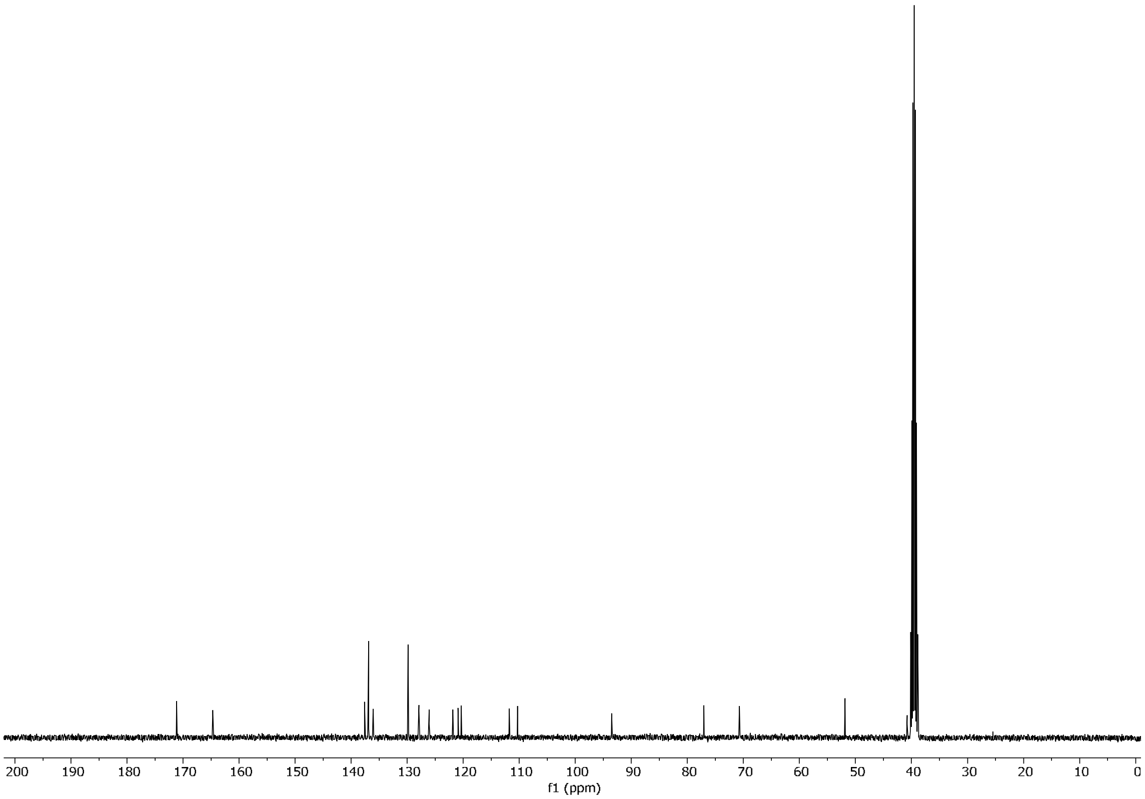

Compound (*S*)-**38i**

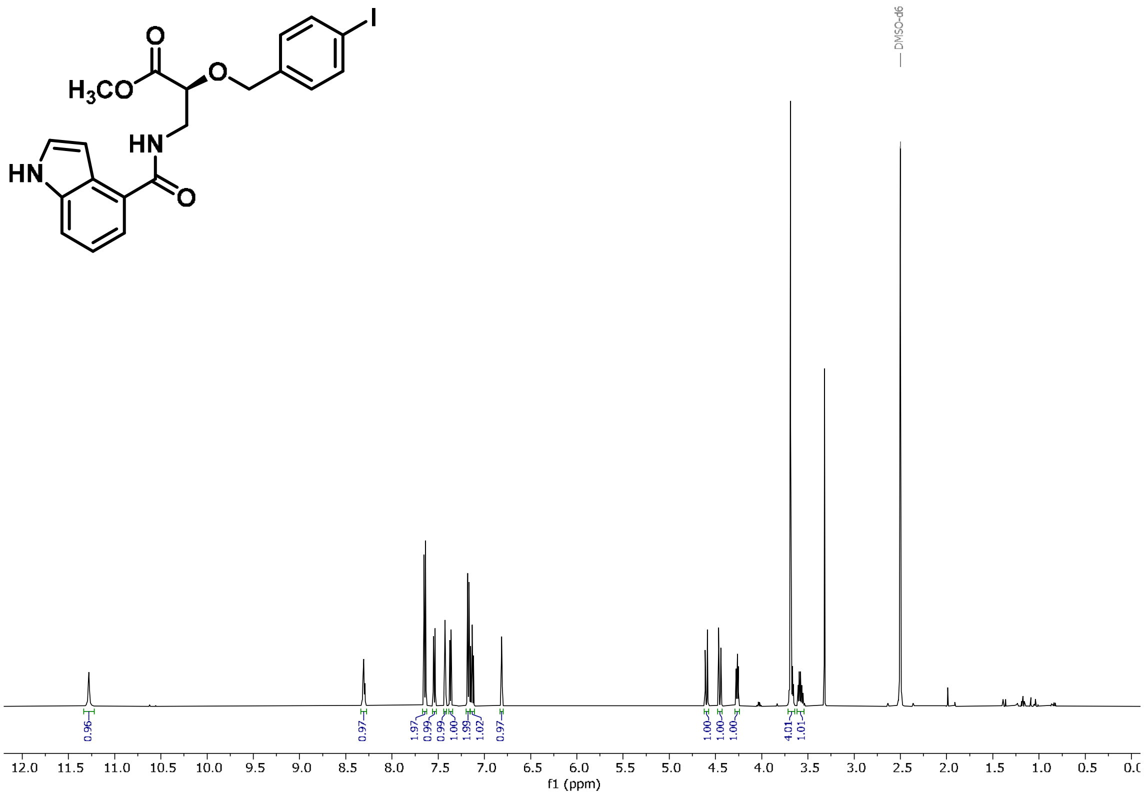

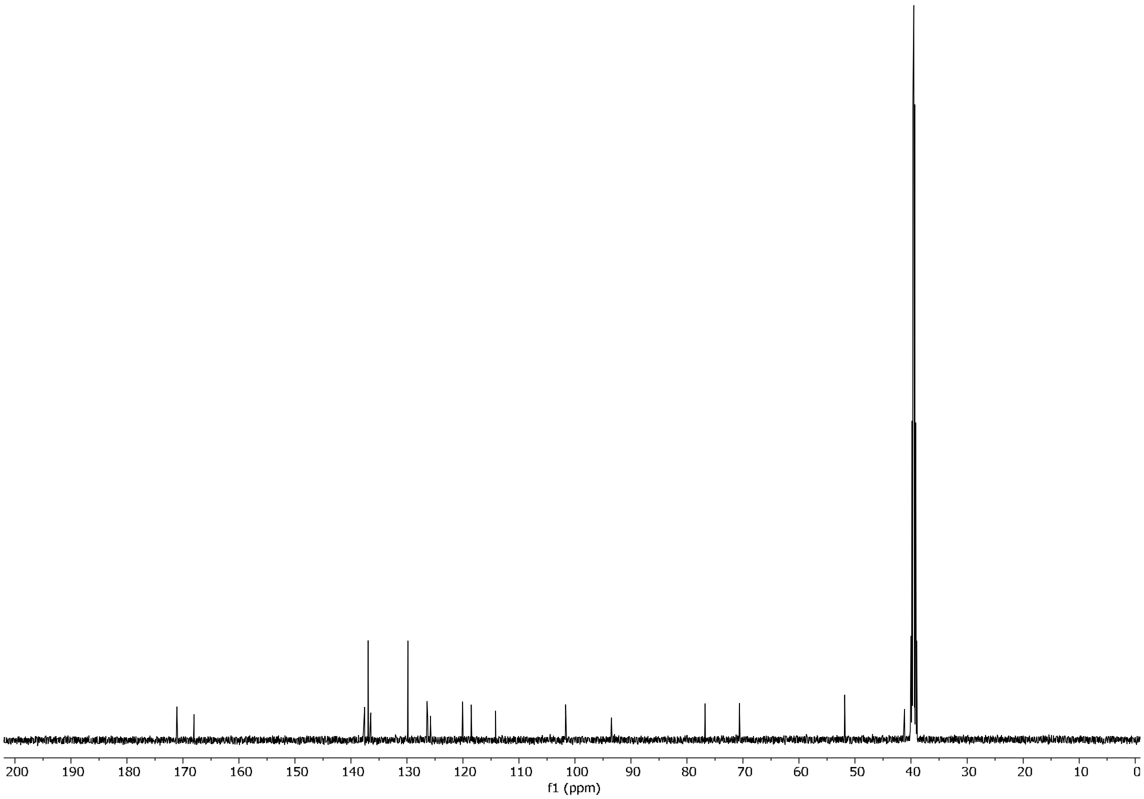

Compound (*S*)-**38j**

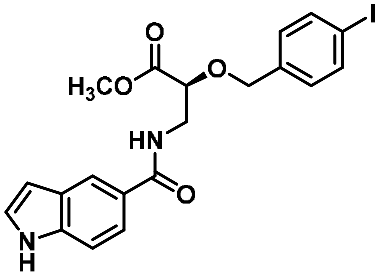

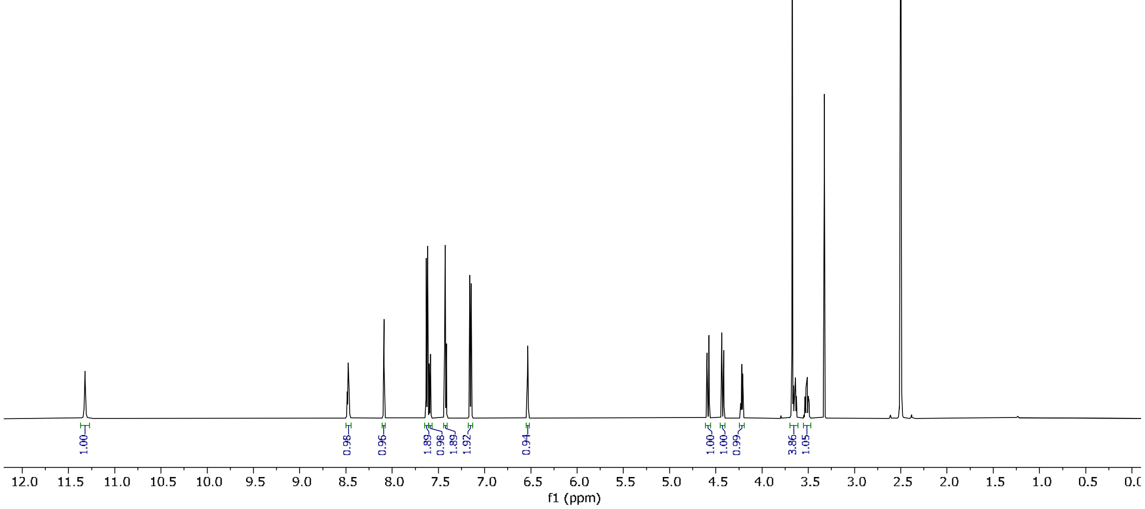

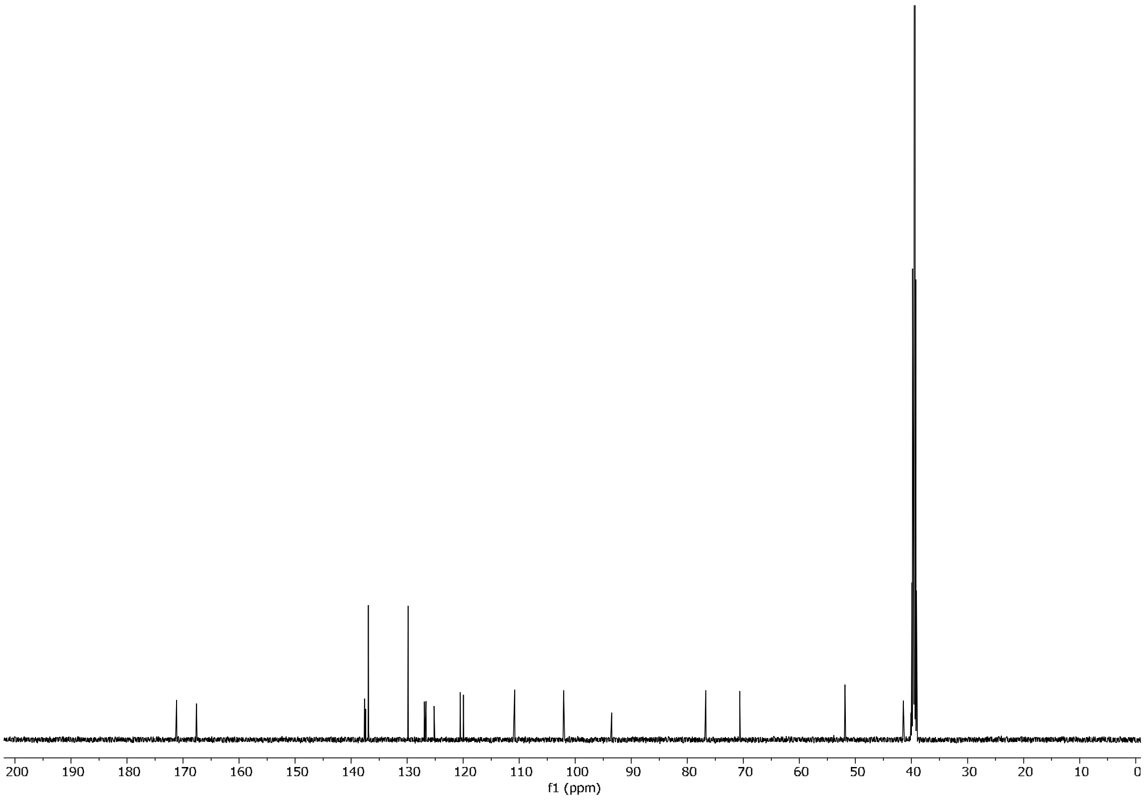

Compound (*S*)-**38k**

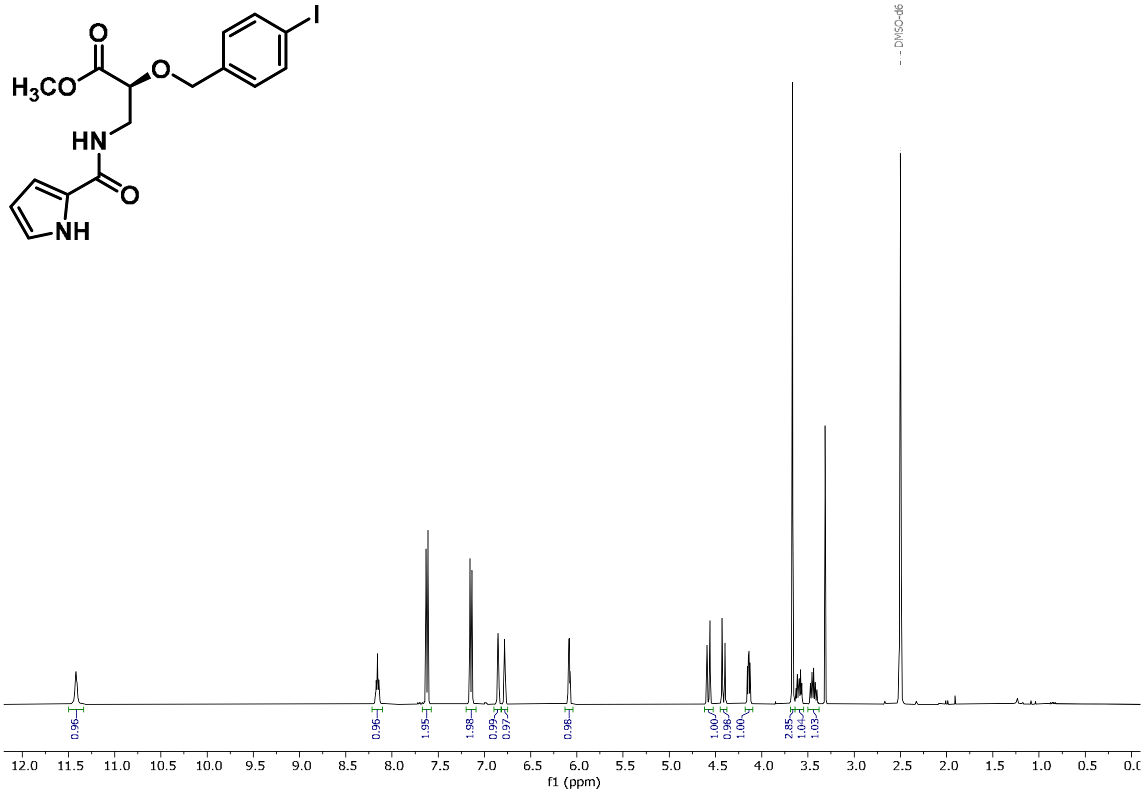

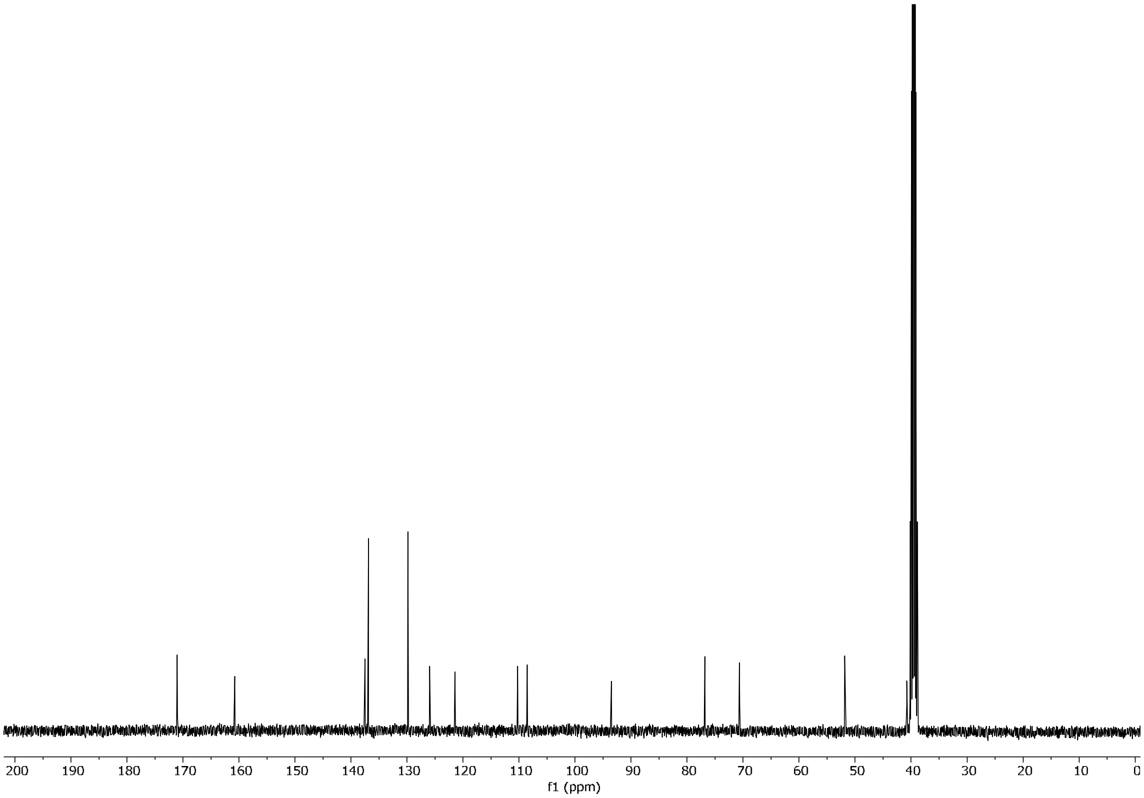

Compound (*S*)-**38l**

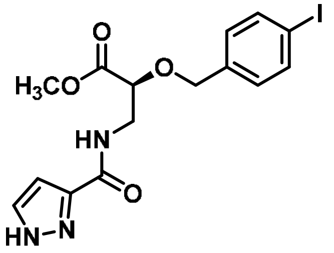

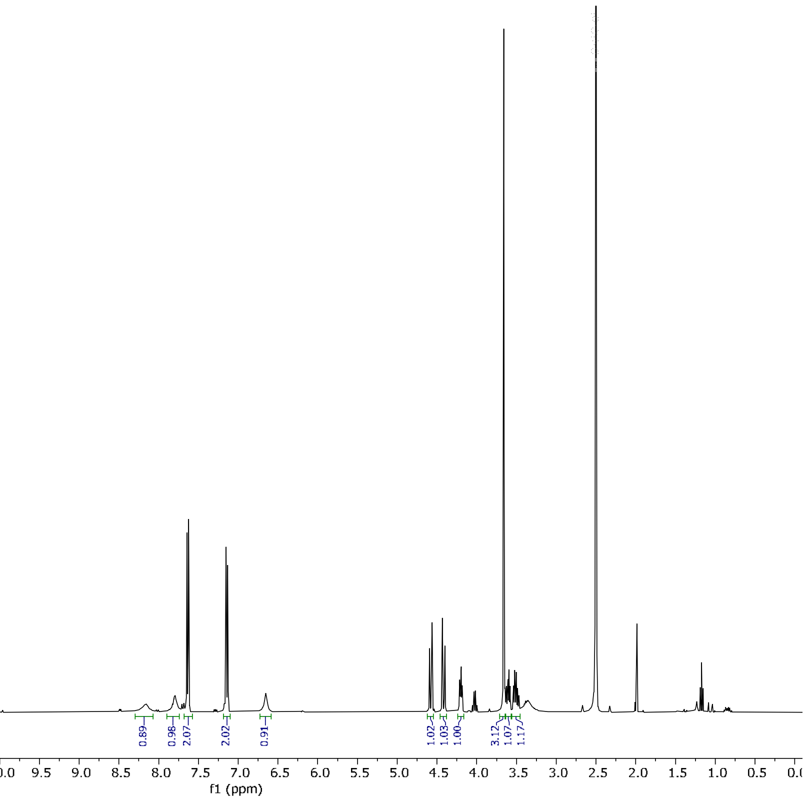

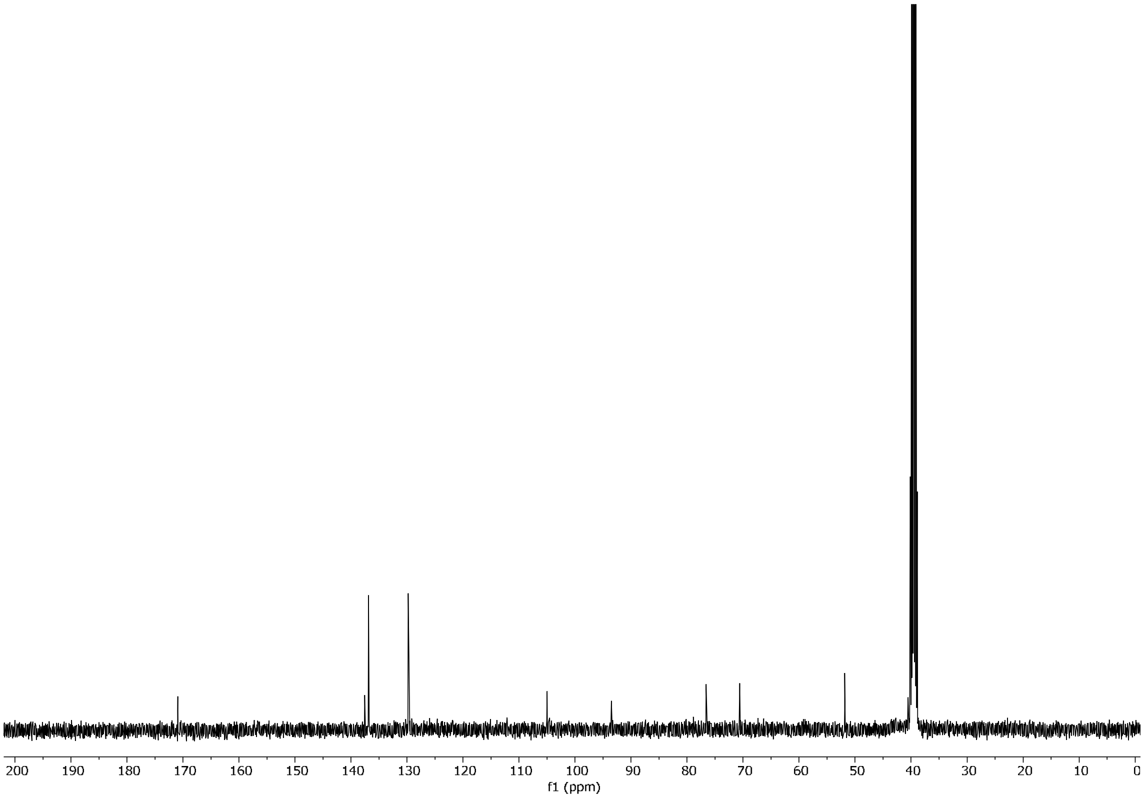

Compound (*S*)-**38m**

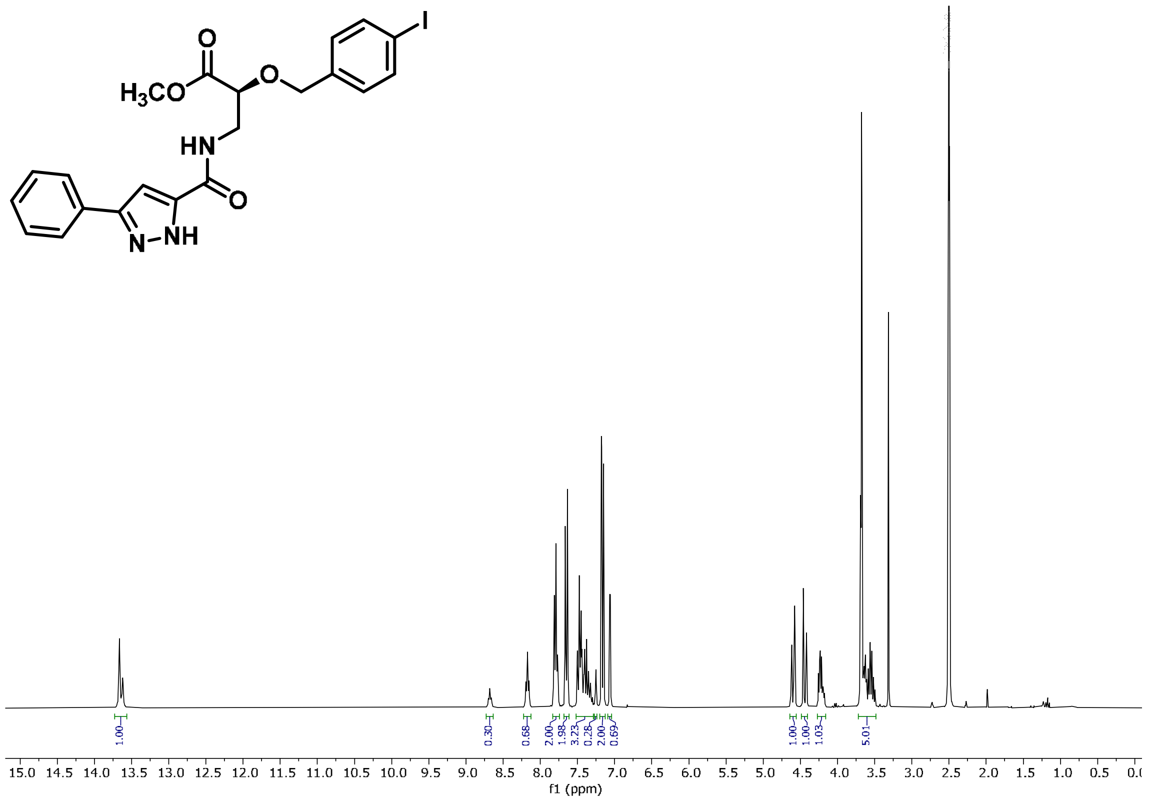

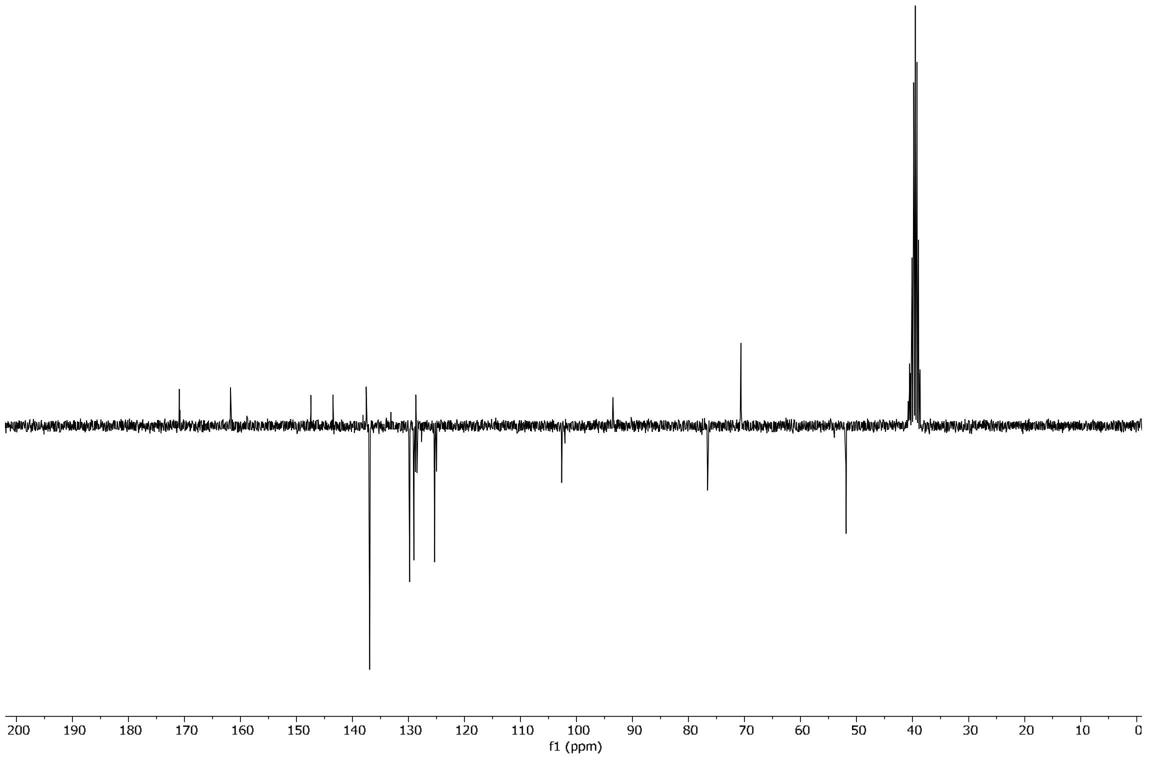

Compound (*S*)-**38n**

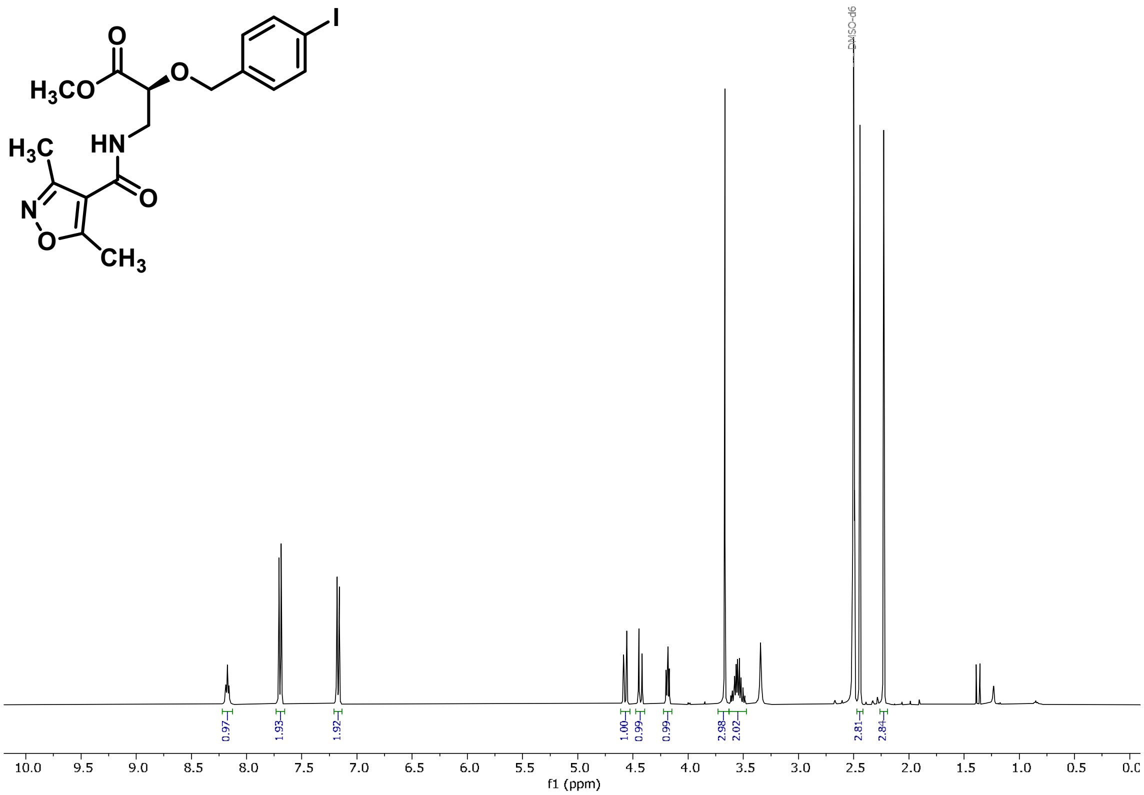

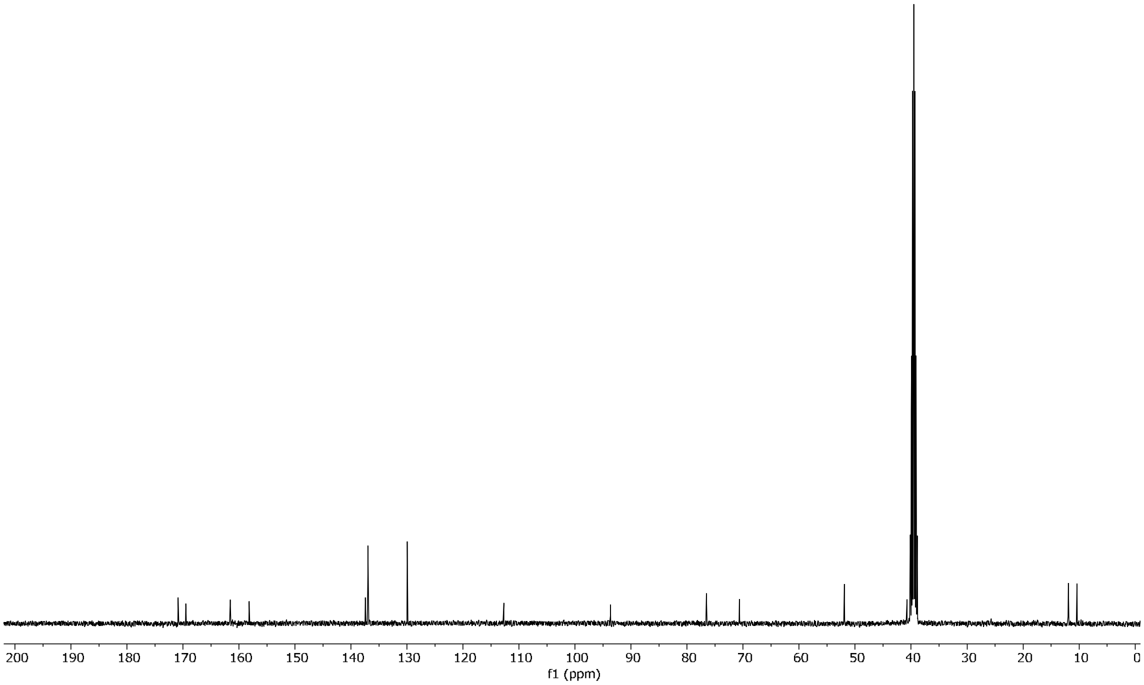

Compound (*S*)-**39a**

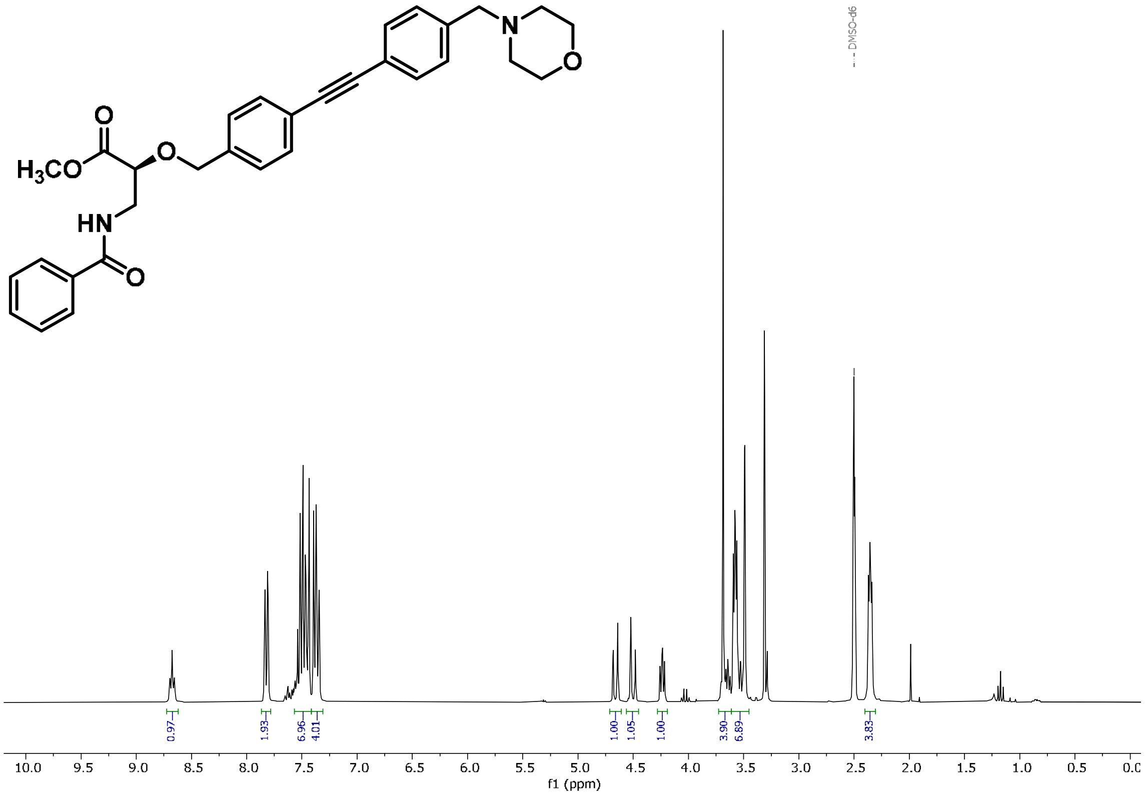

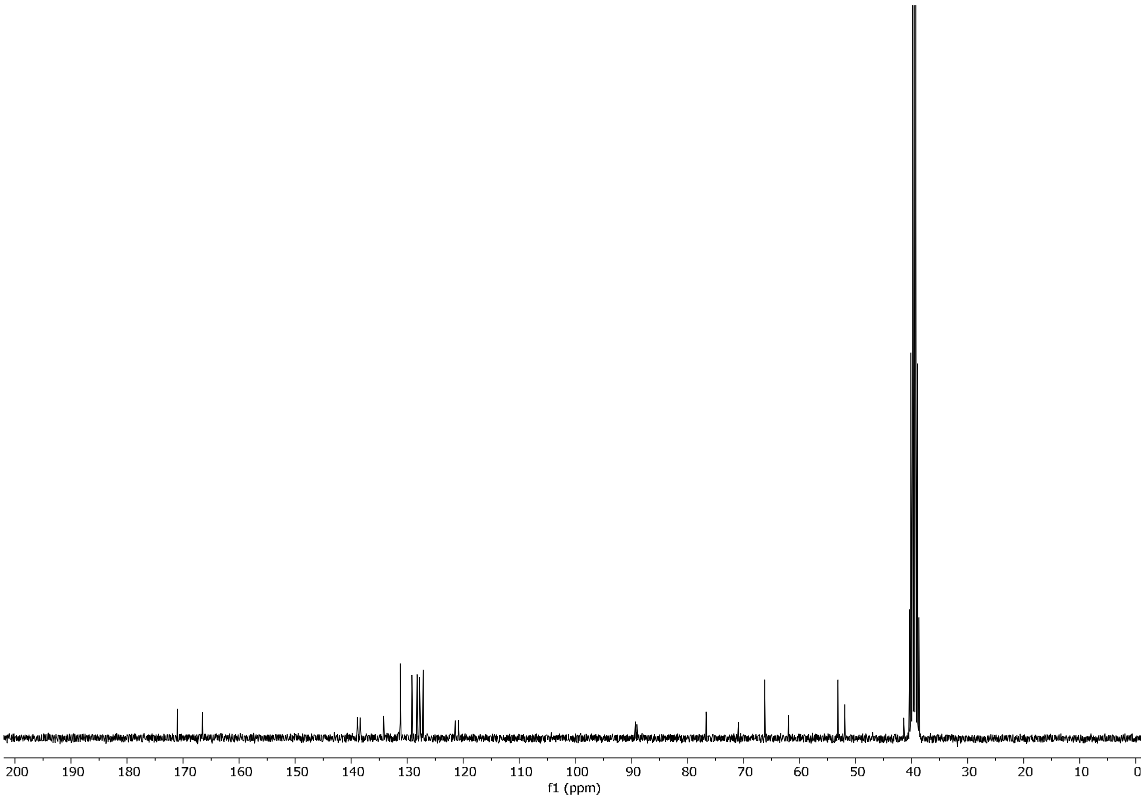

Compound (*S*)-**39b**

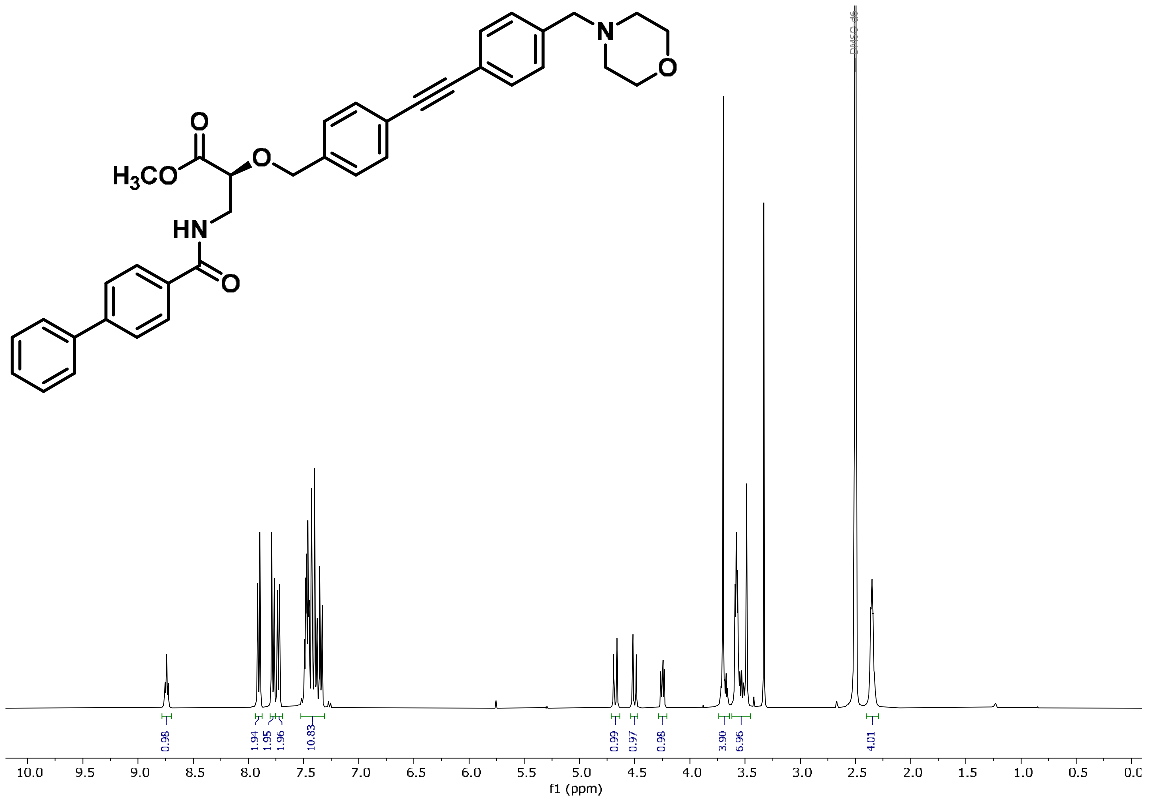

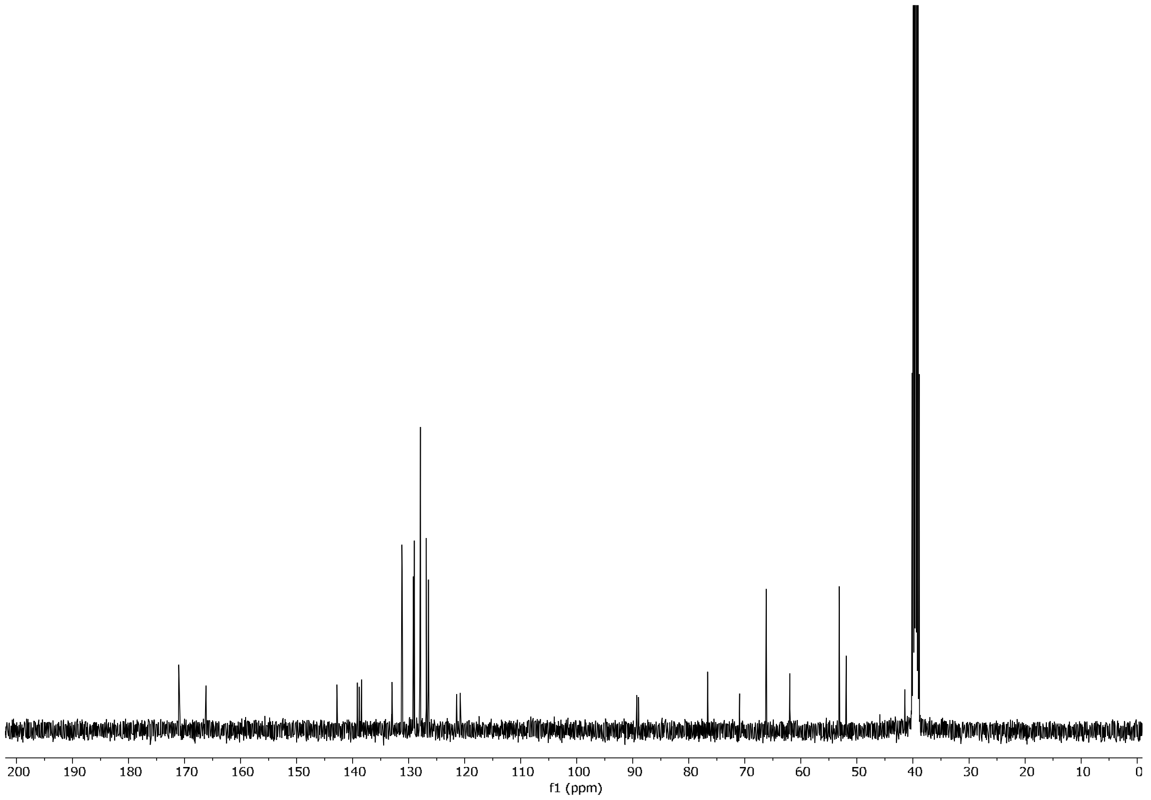

Compound (*S*)-**39c**

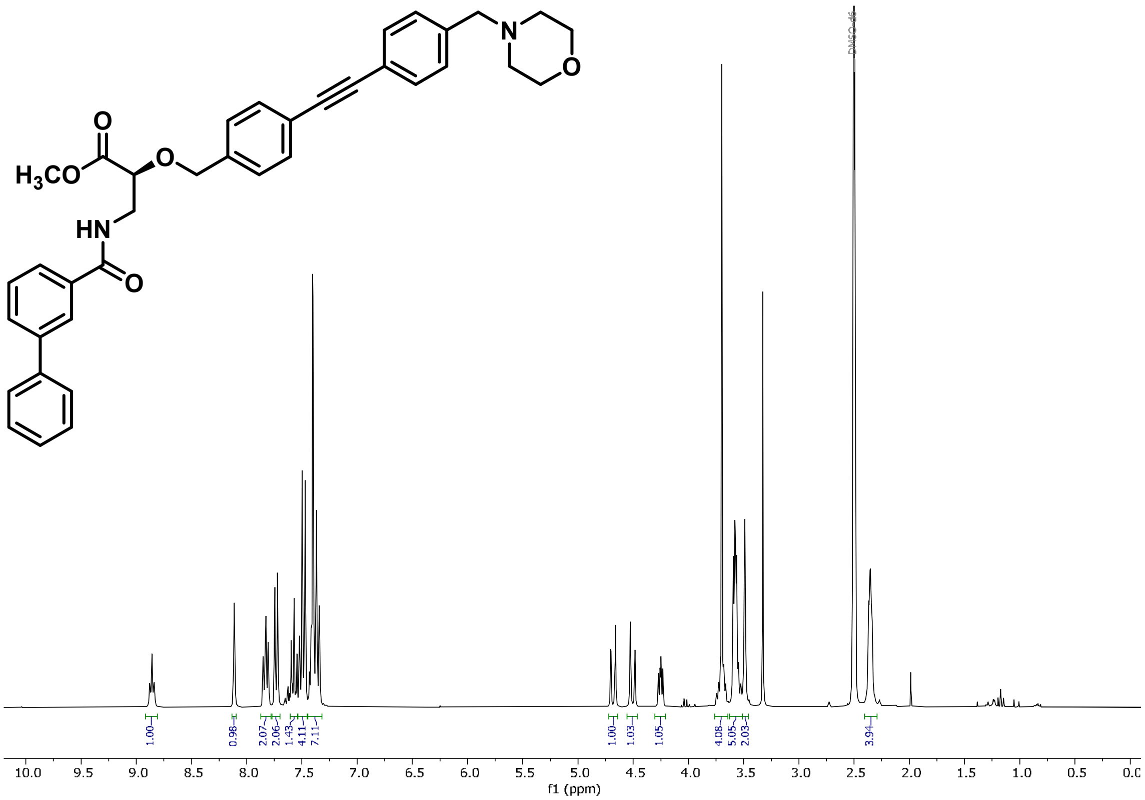

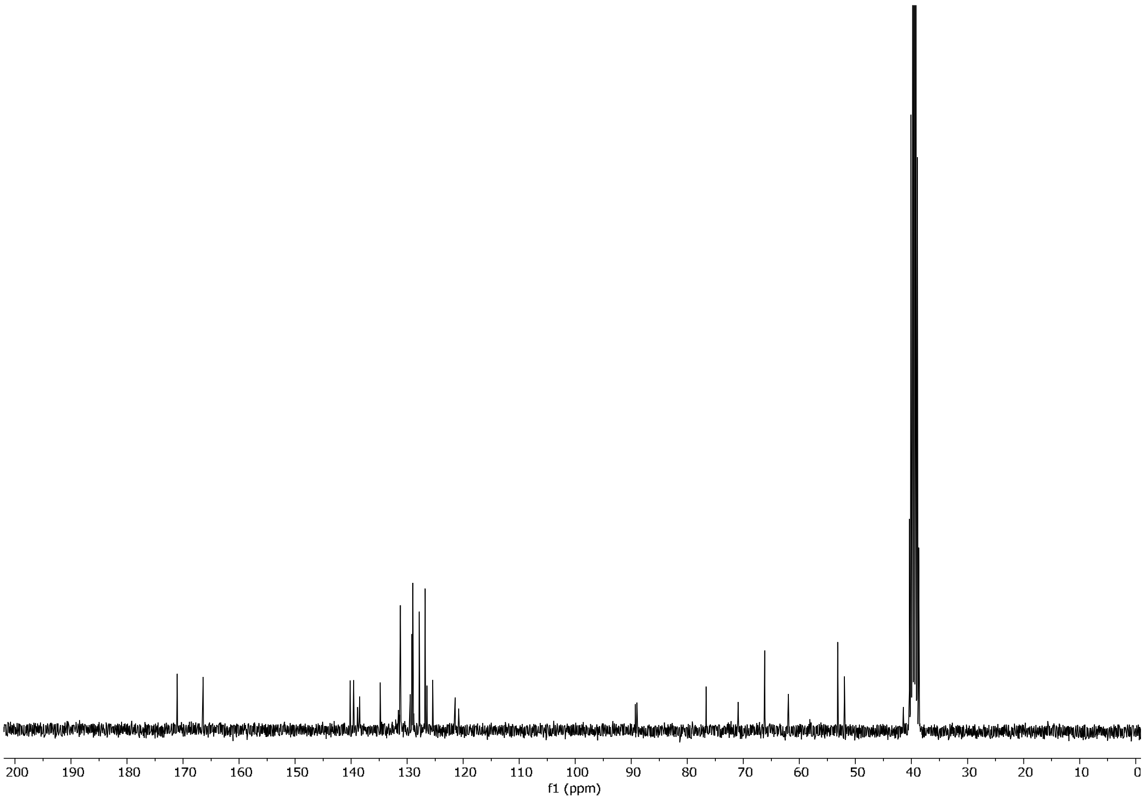

Compound (*R*)-**39d**

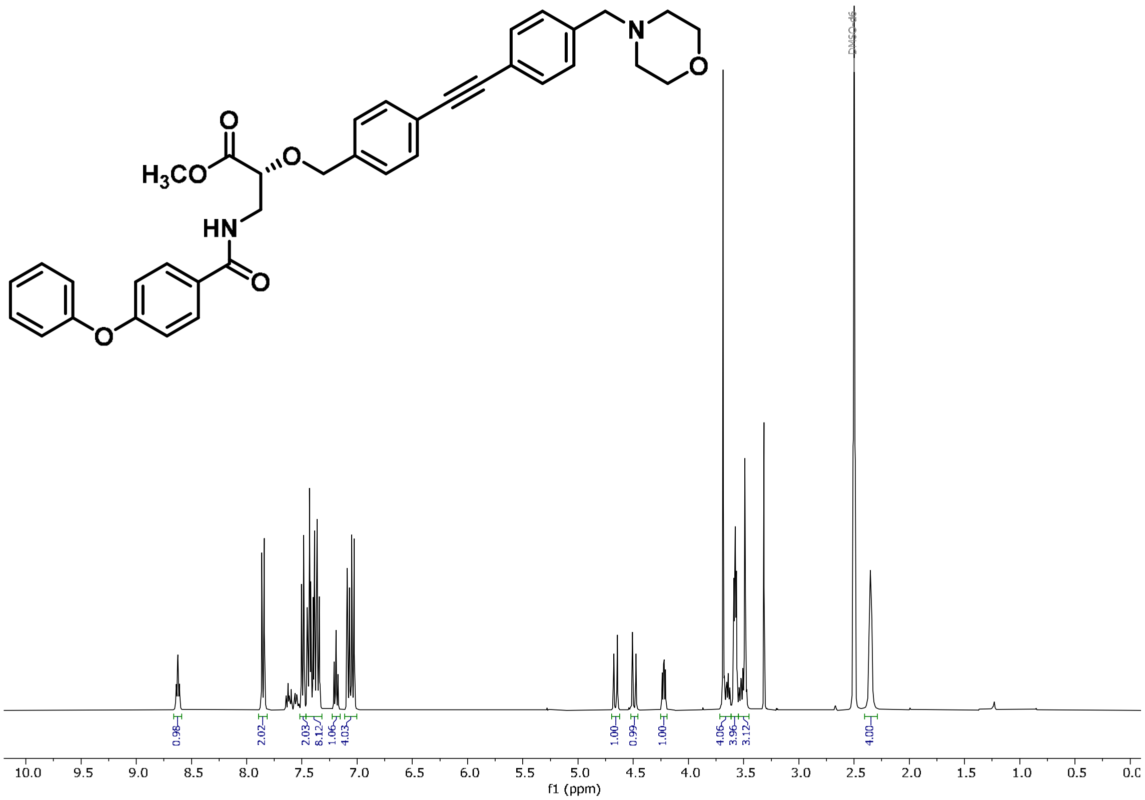

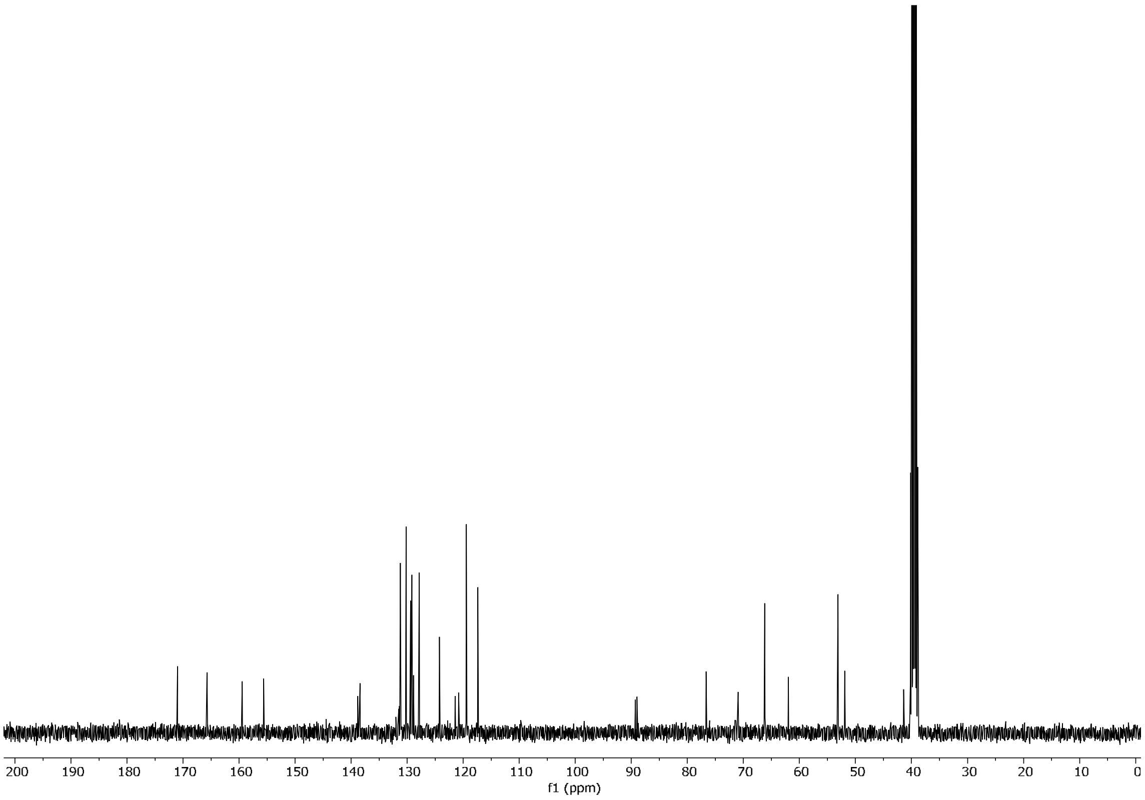

Compound (*R*)-**39e**

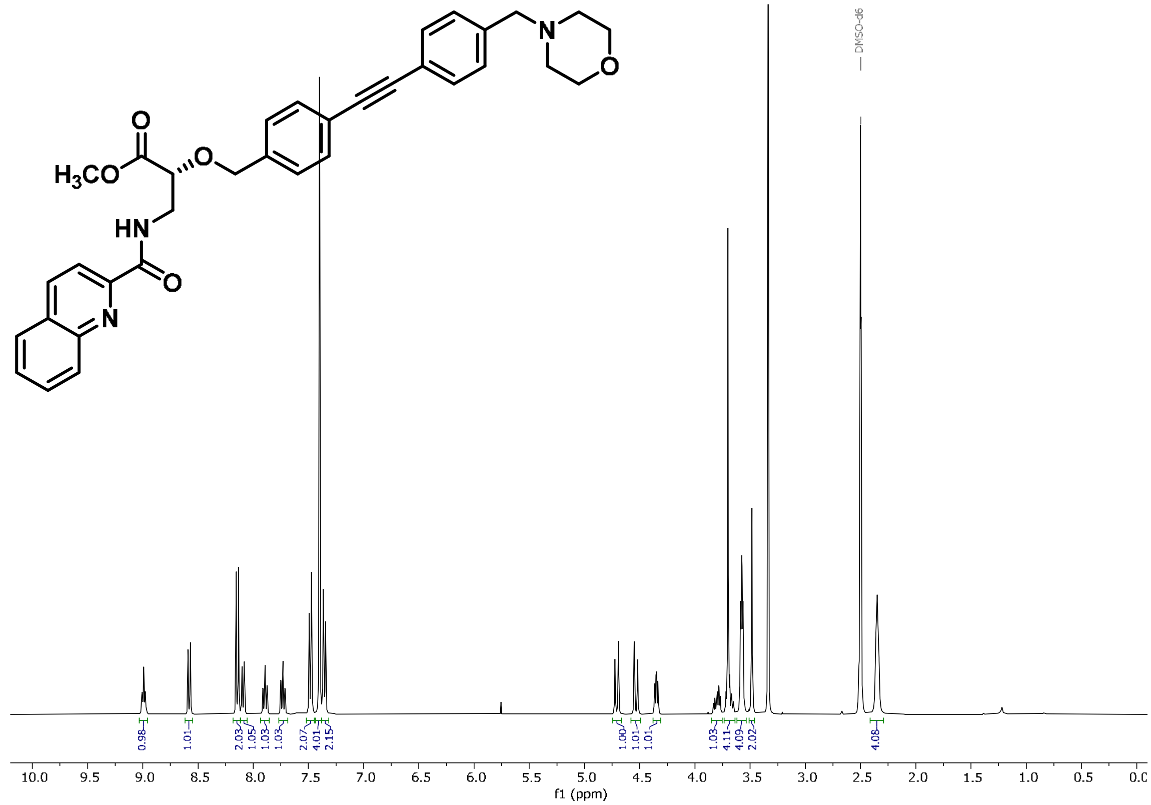

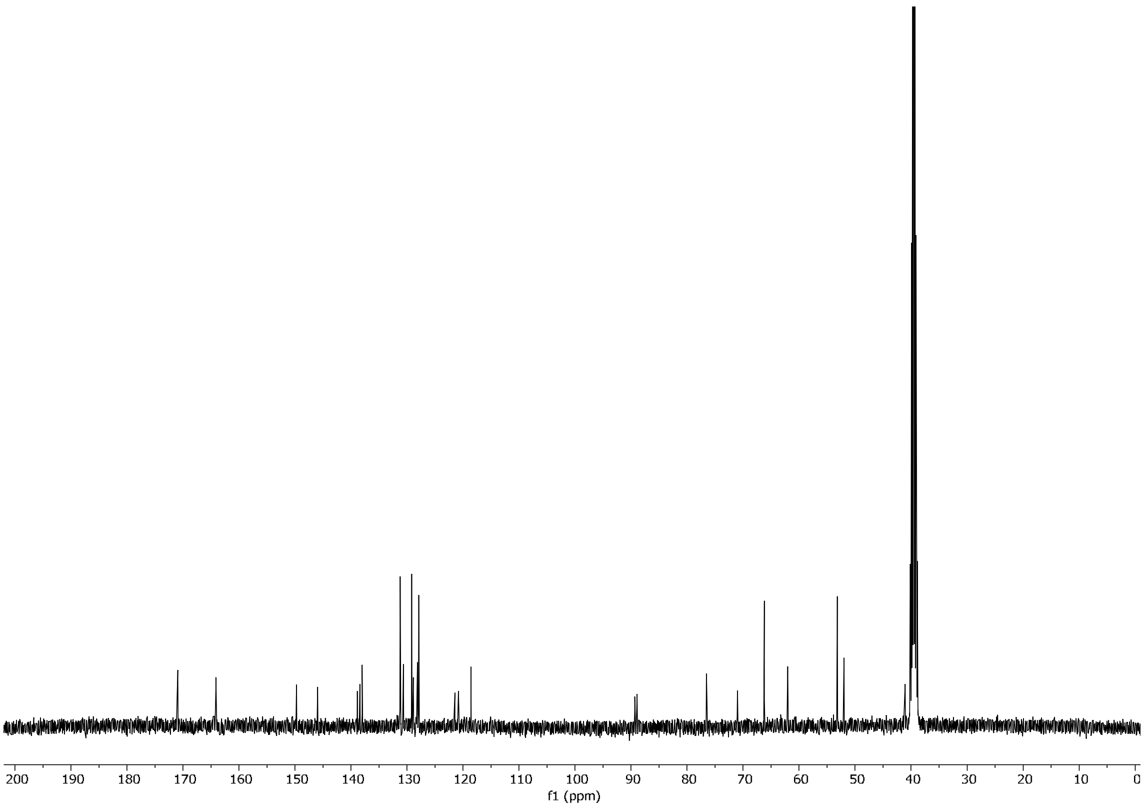

Compound (*S*)-**39f**

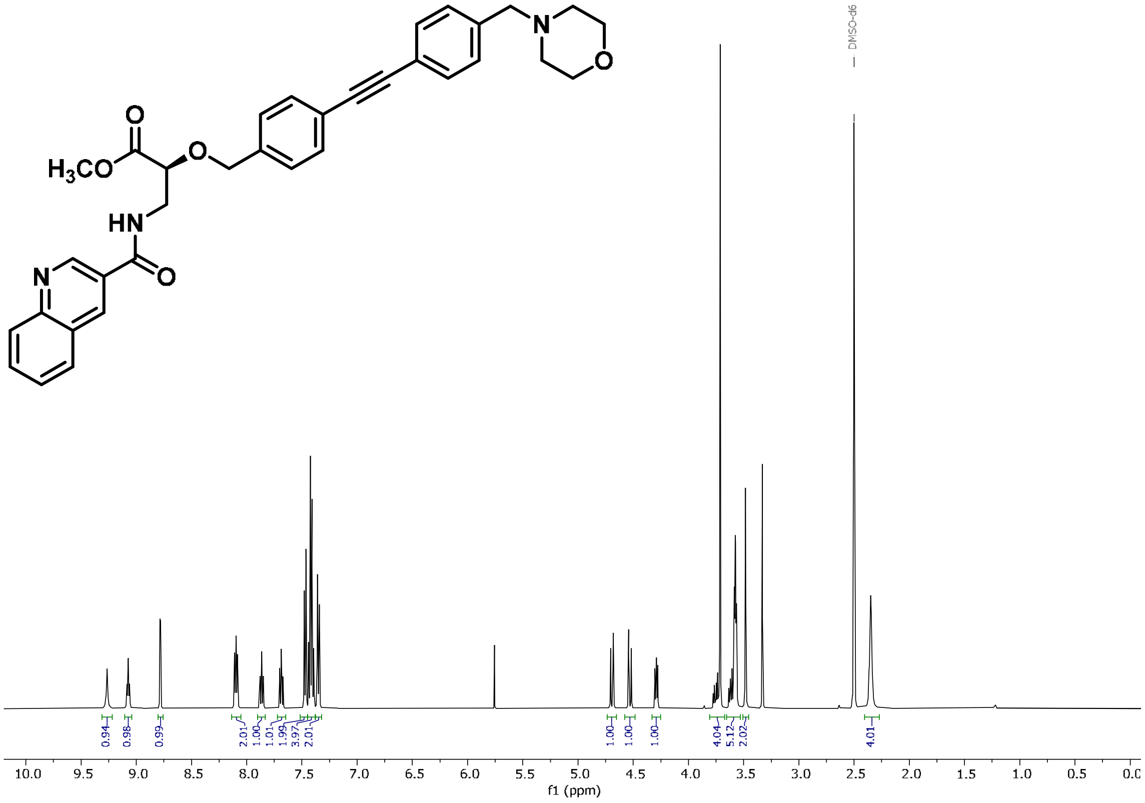

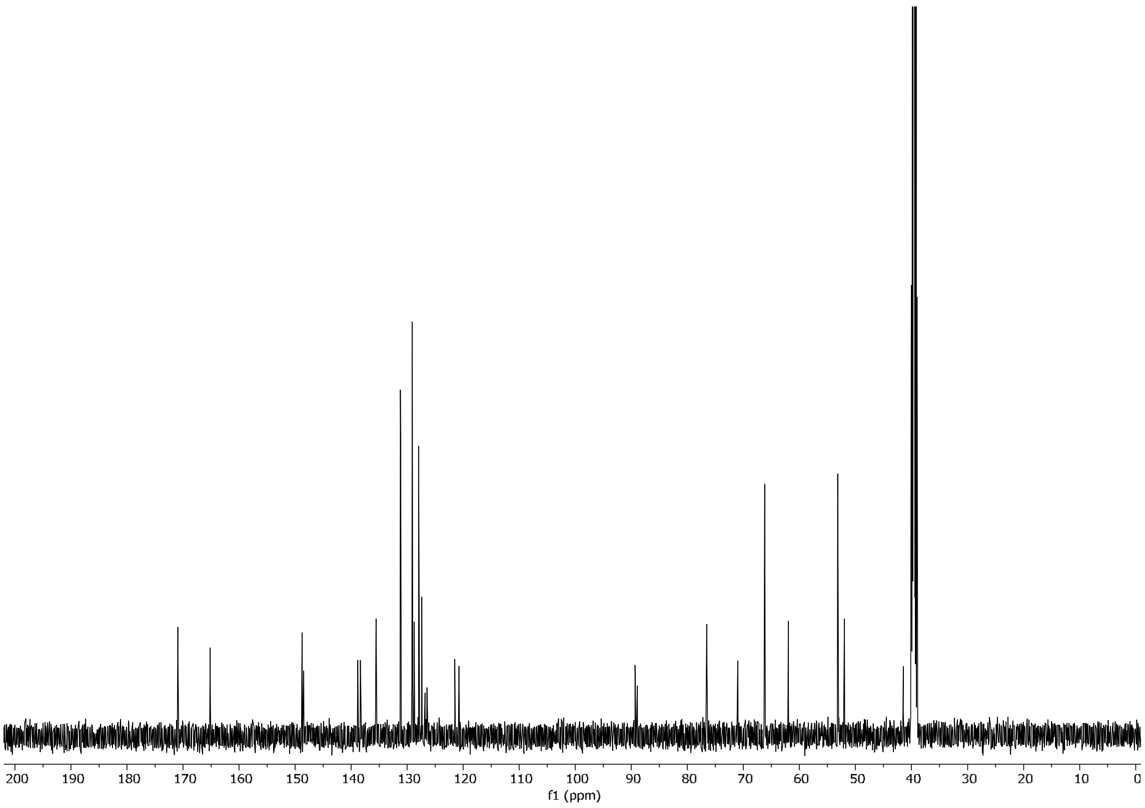

Compound (*S*)-**39g**

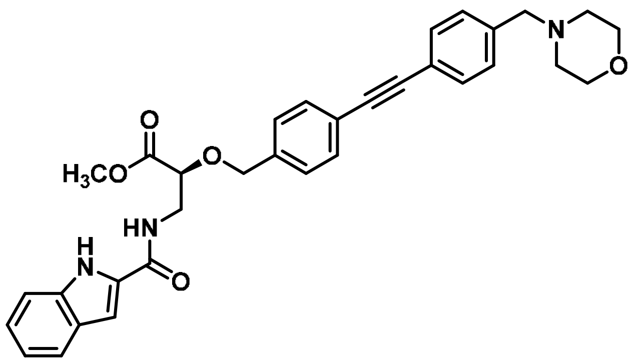

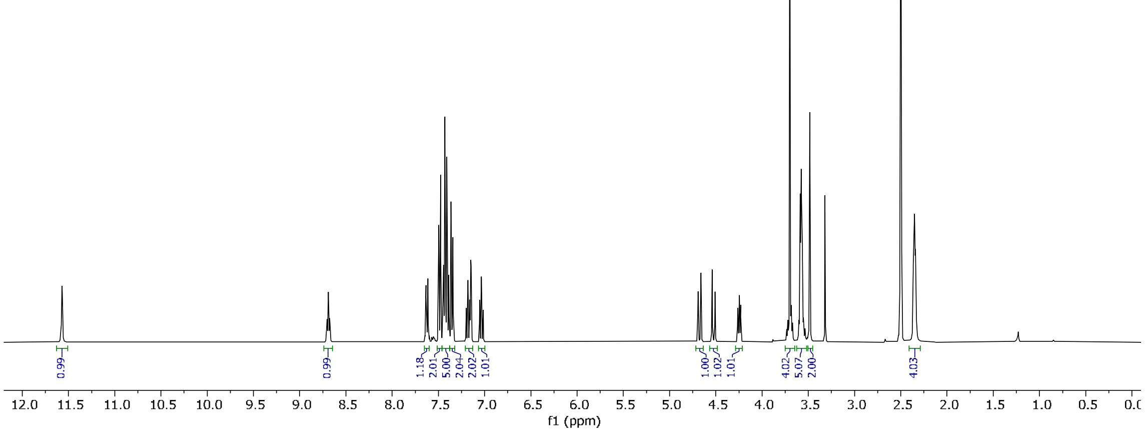

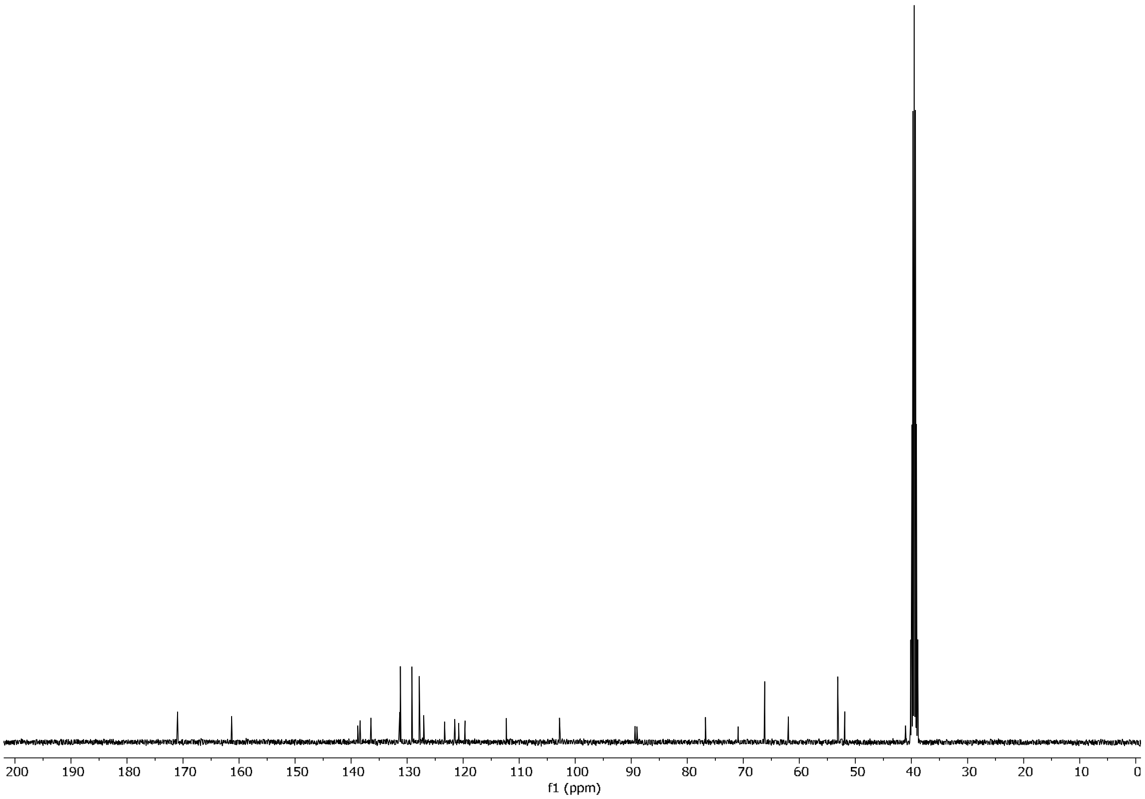

Compound (*S*)-**39h**

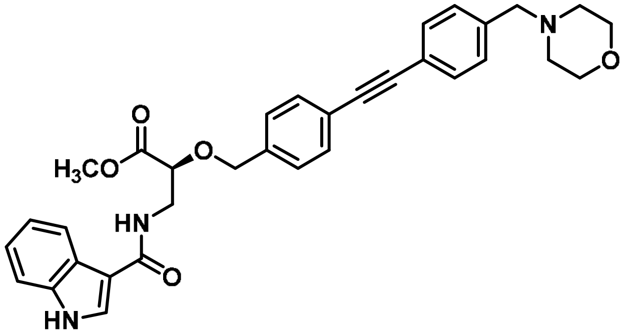

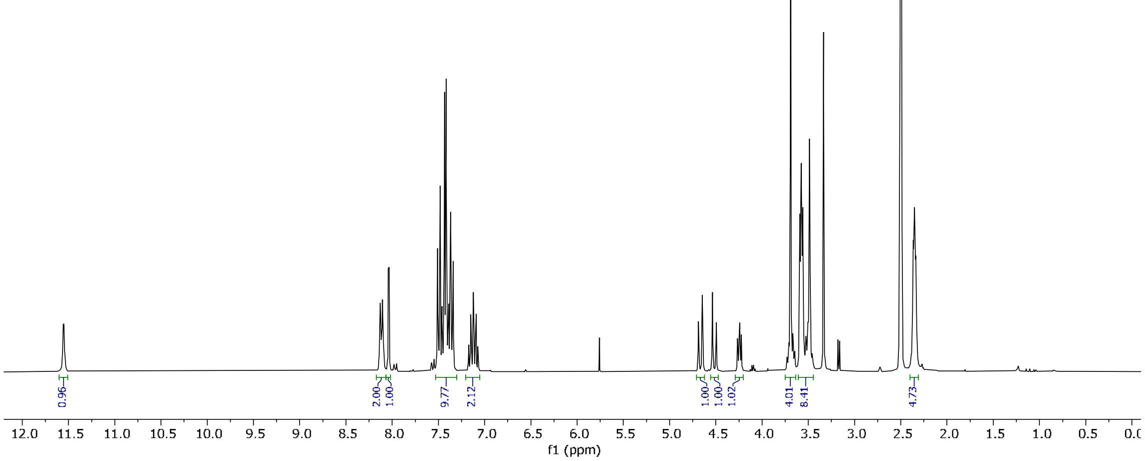

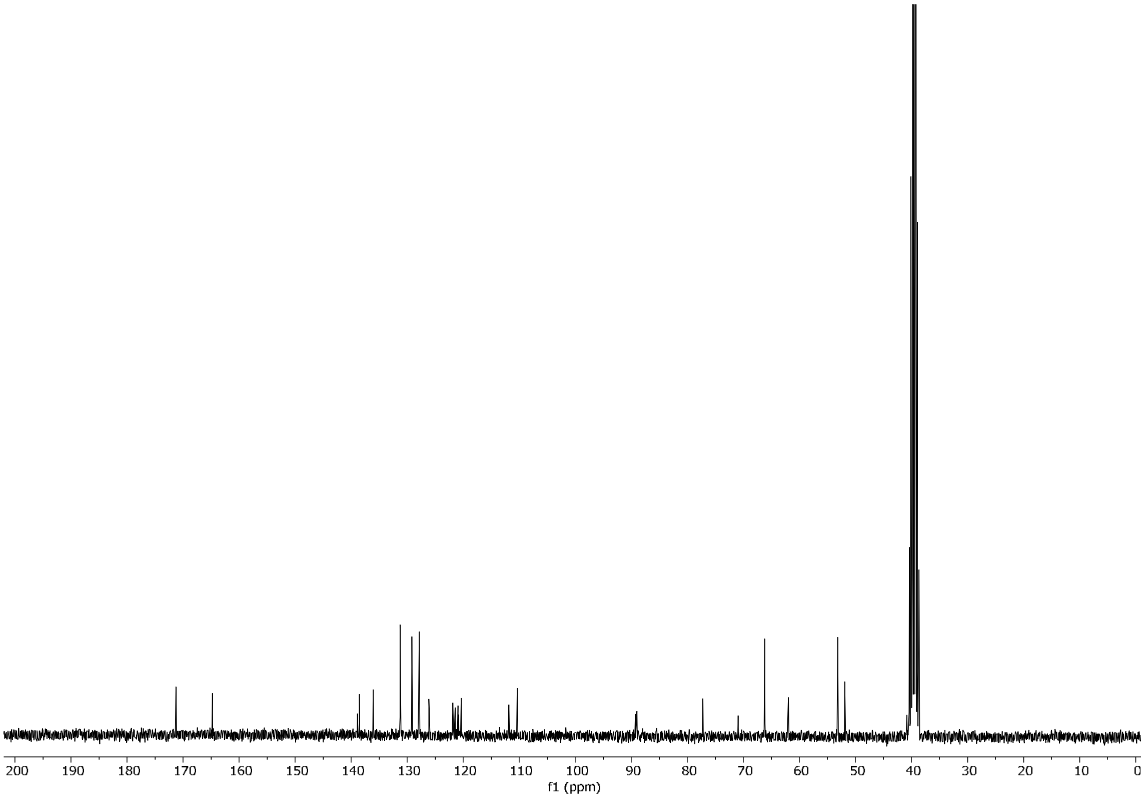

Compound (*S*)-**39i**

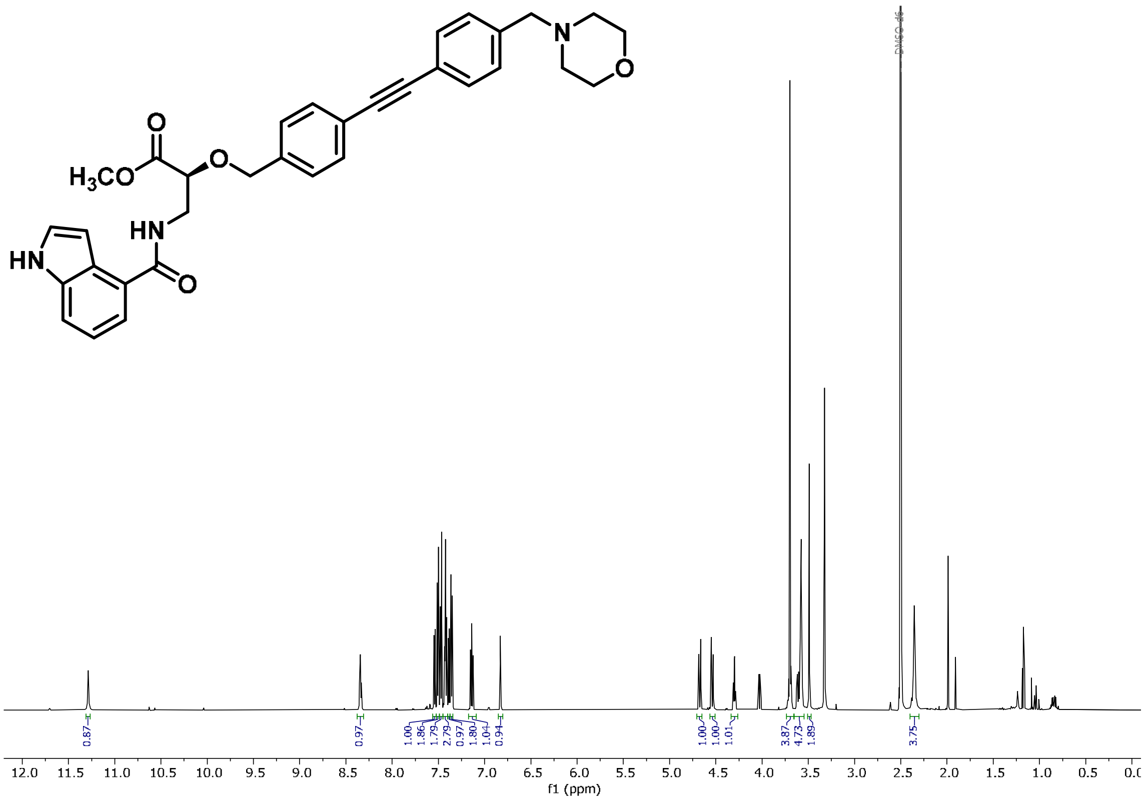

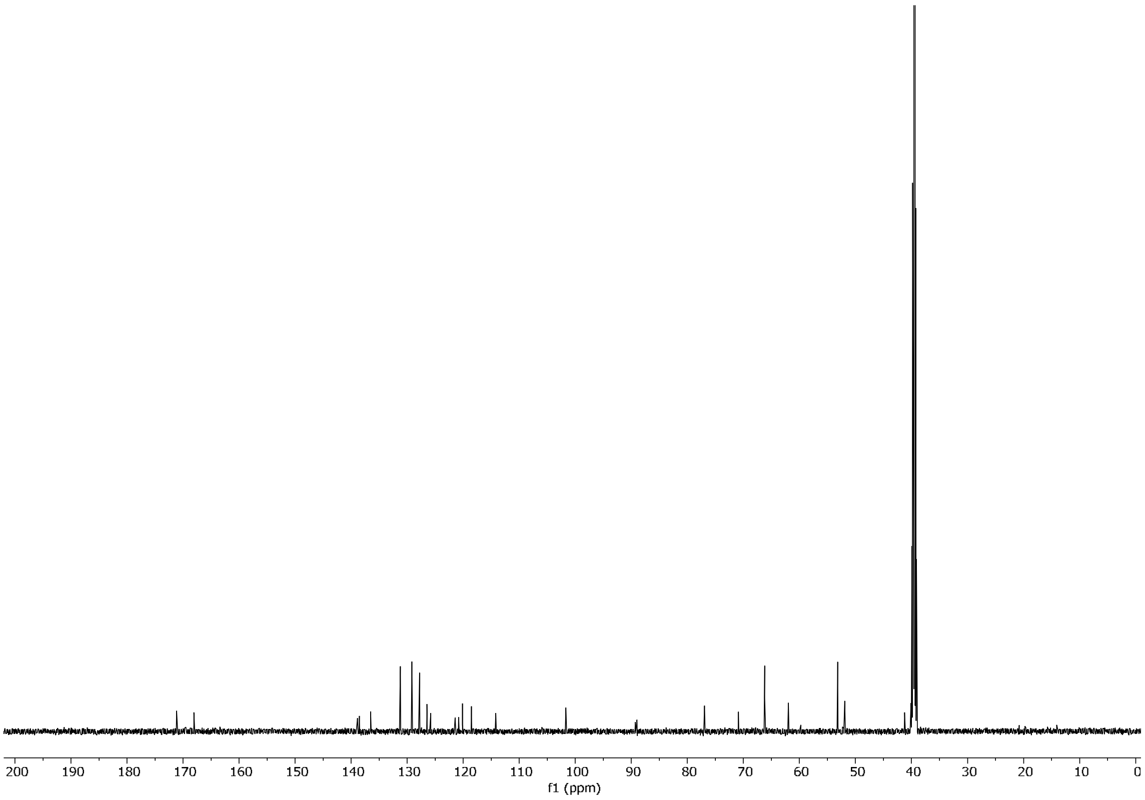

Compound (*R*)-**39j**

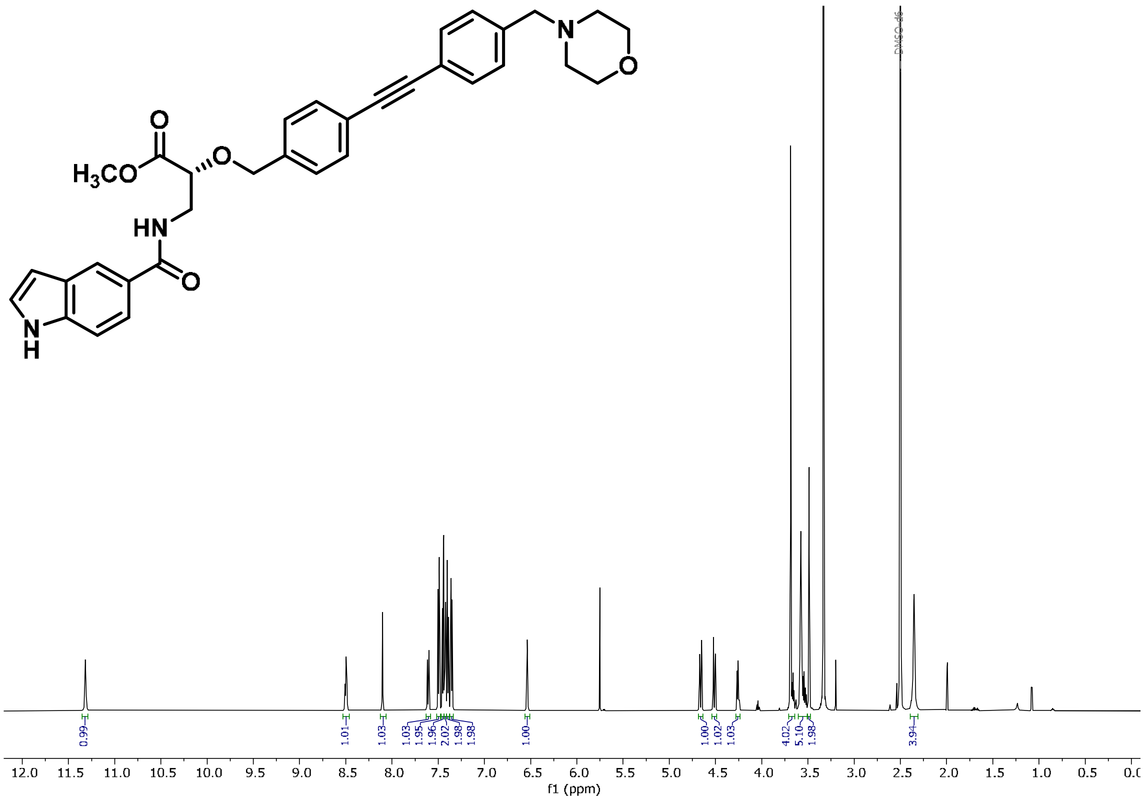

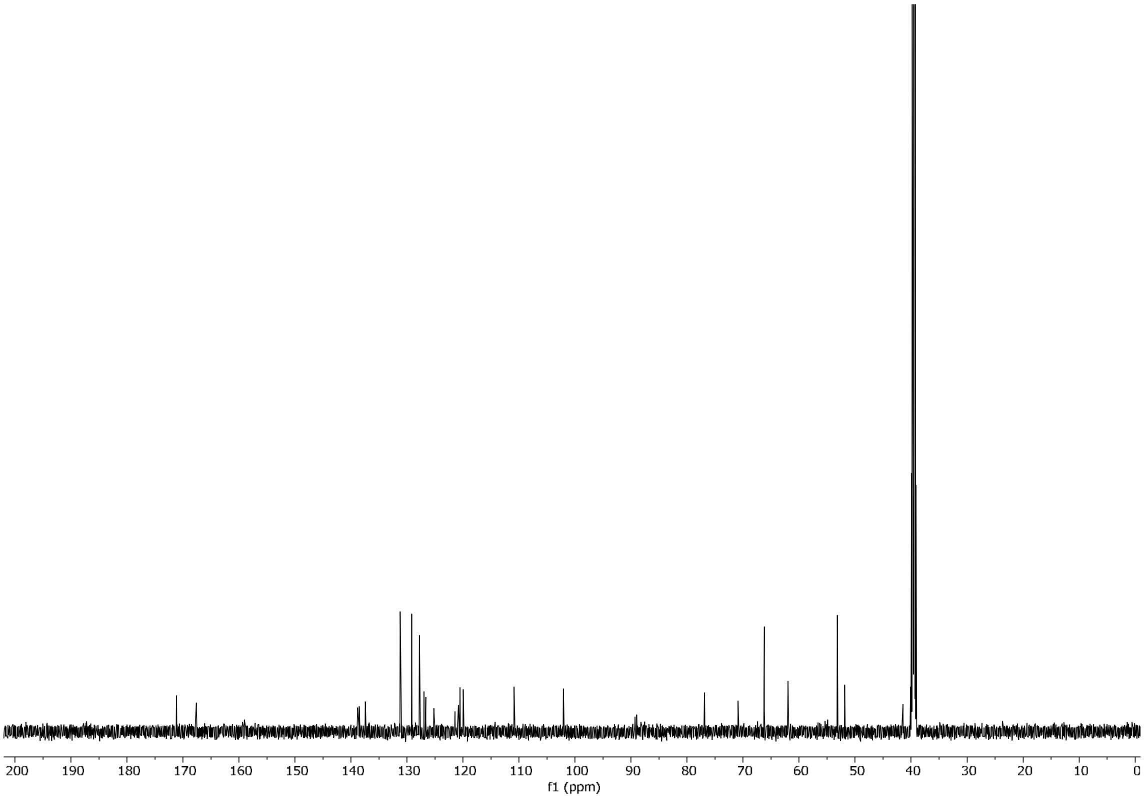

Compound (*S*)-**39k**

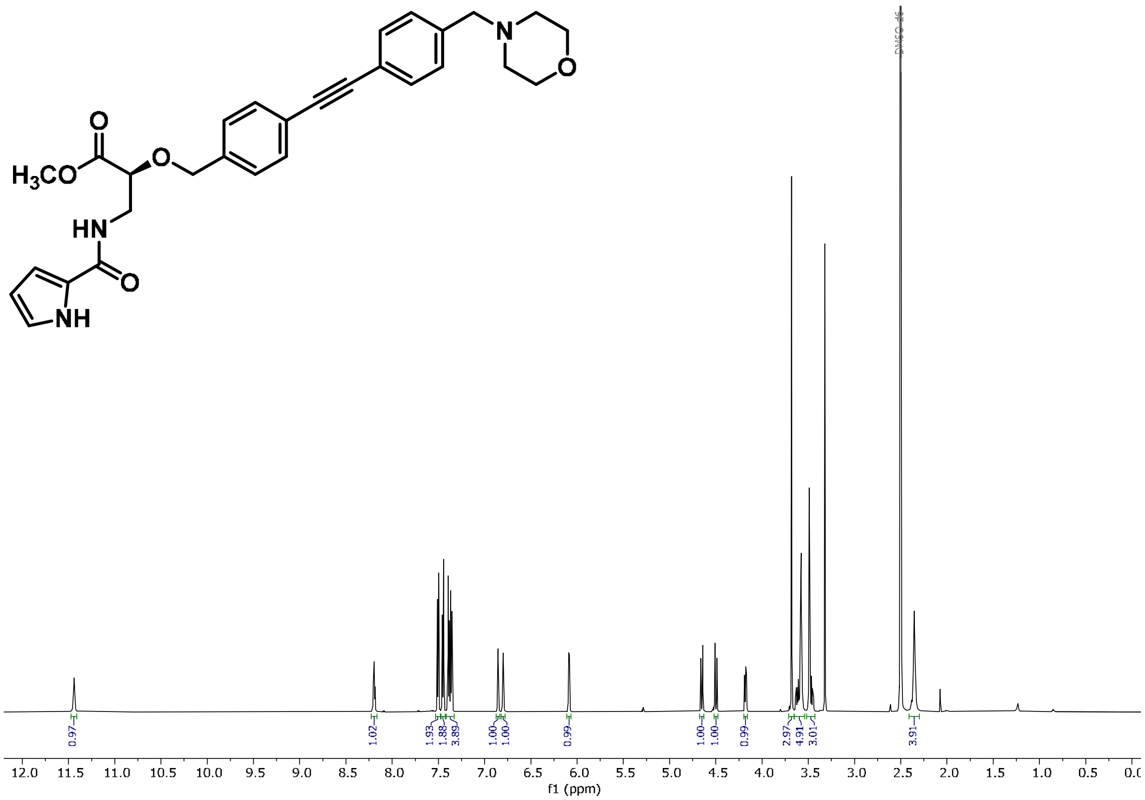

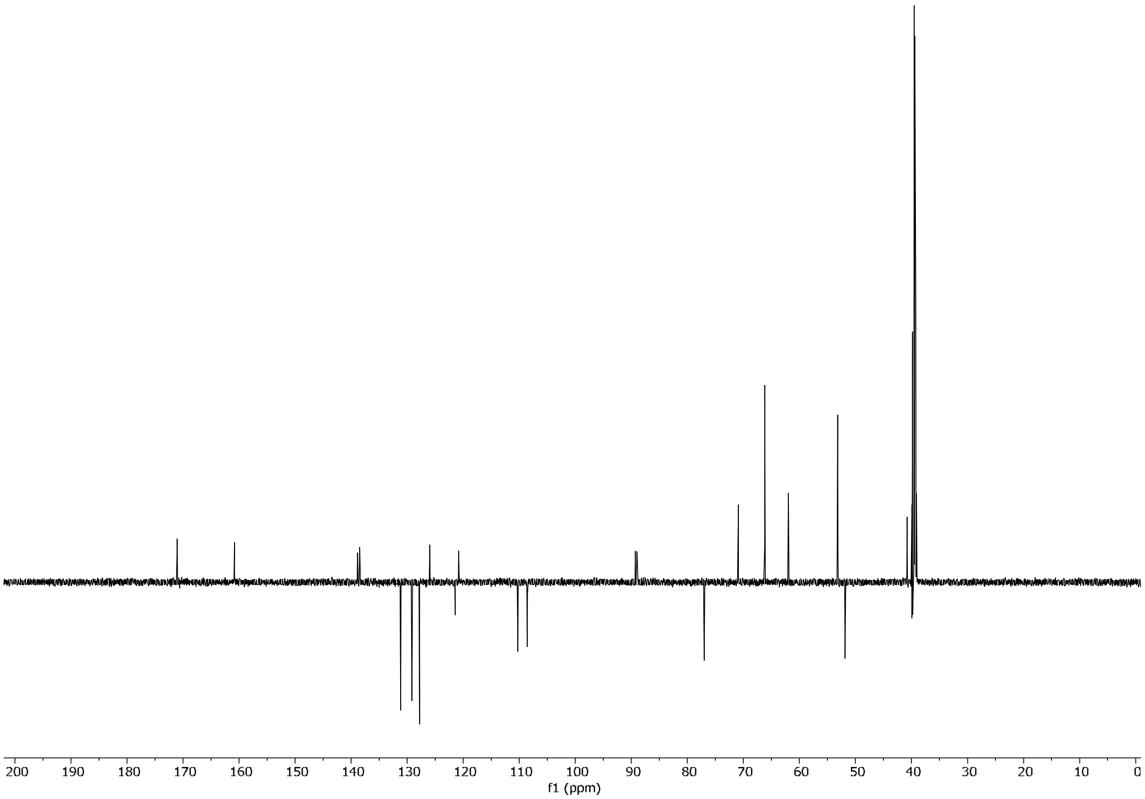

Compound (*S*)-**39l**

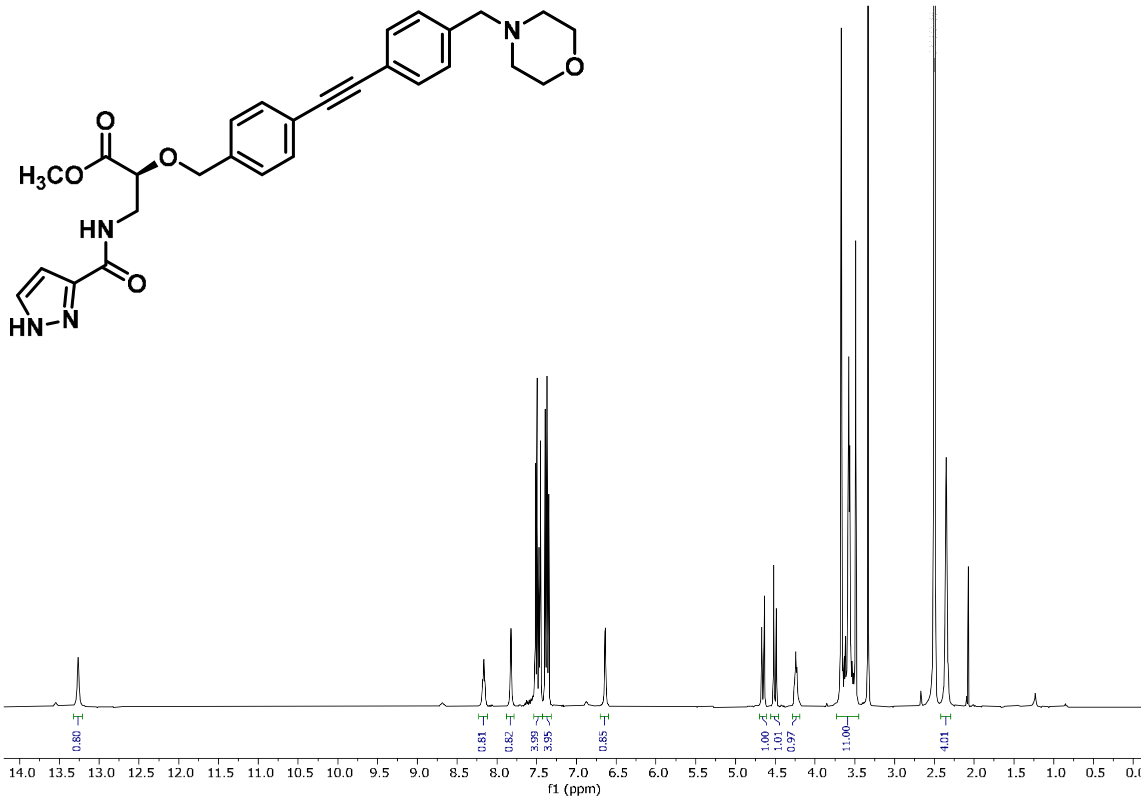

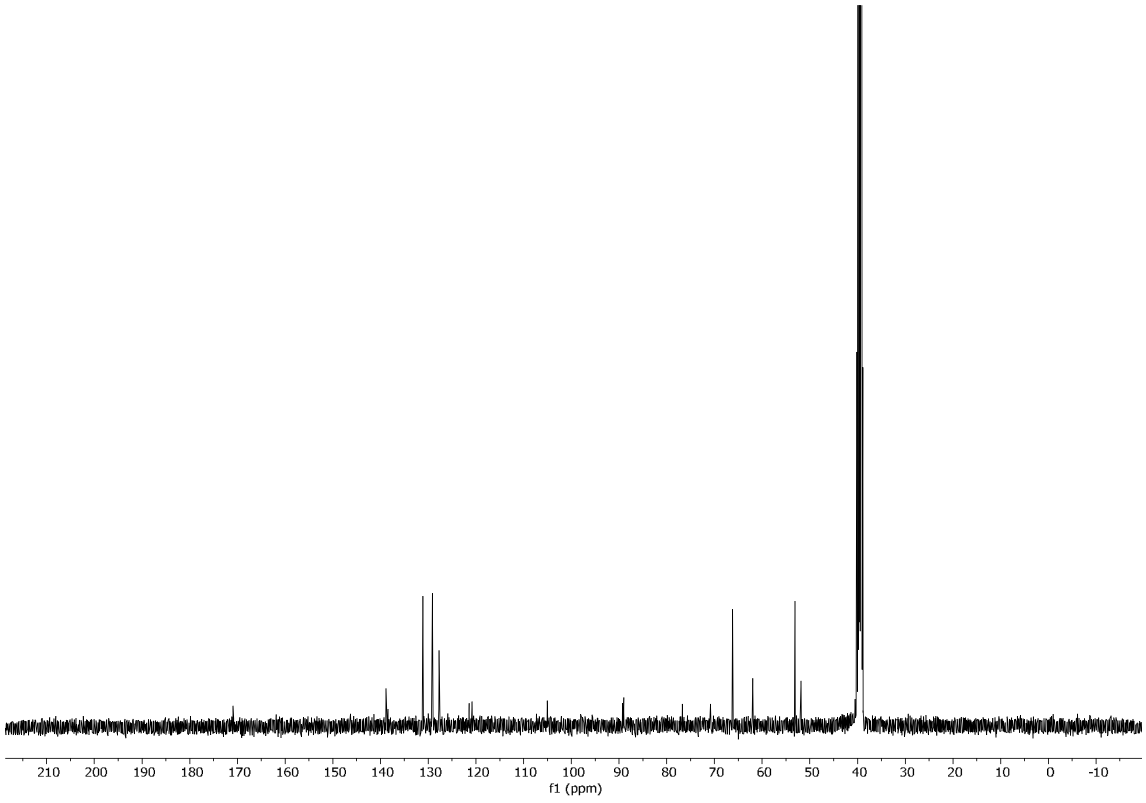

Compound (*S*)-**39m**

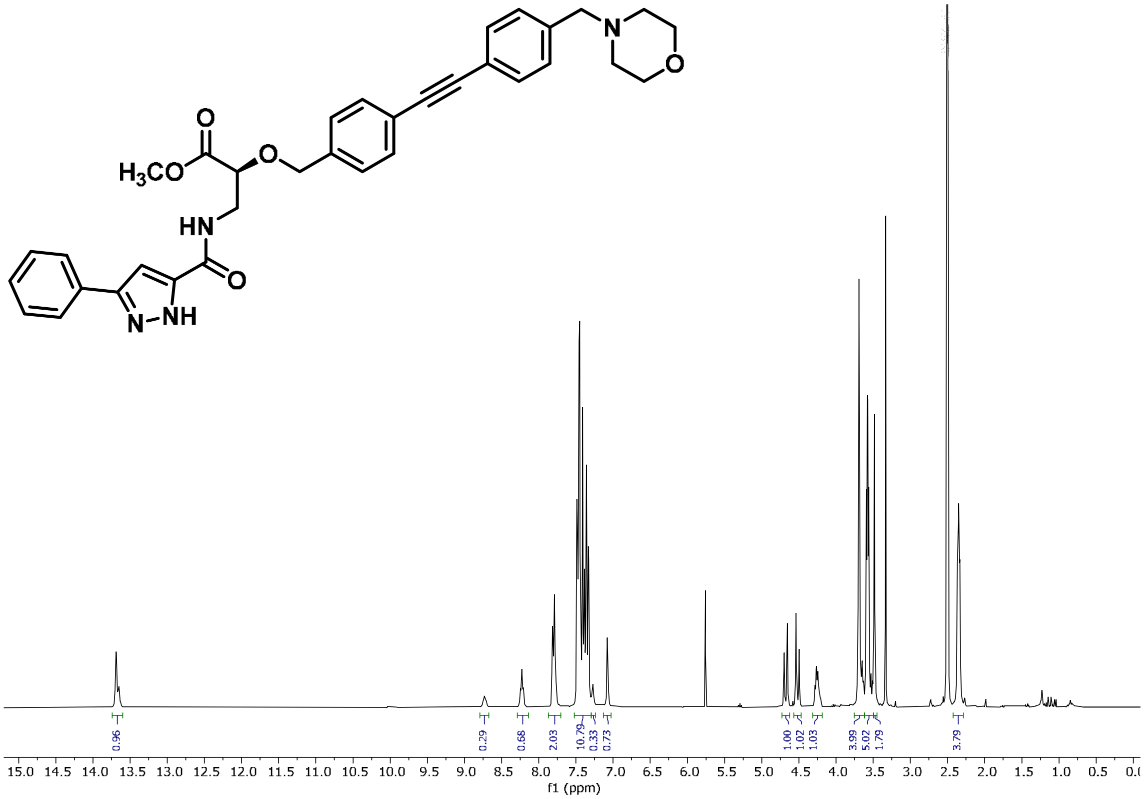

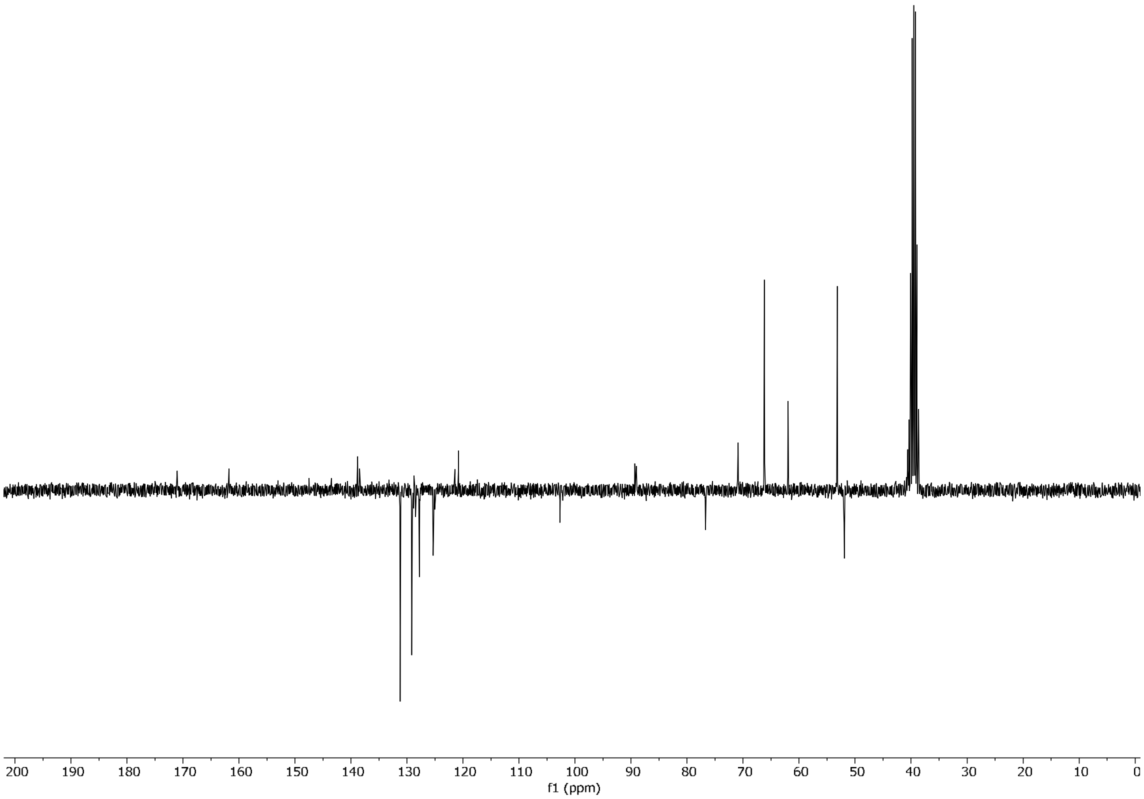

Compound (*S*)-**39n**

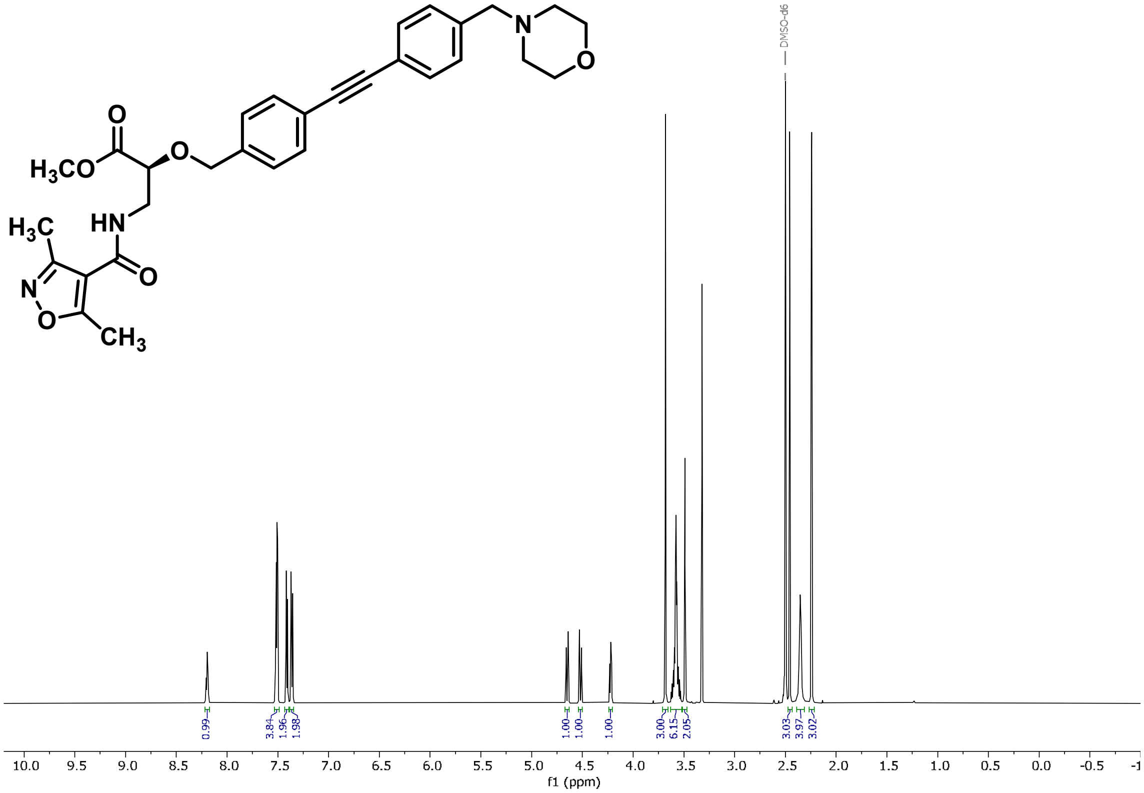

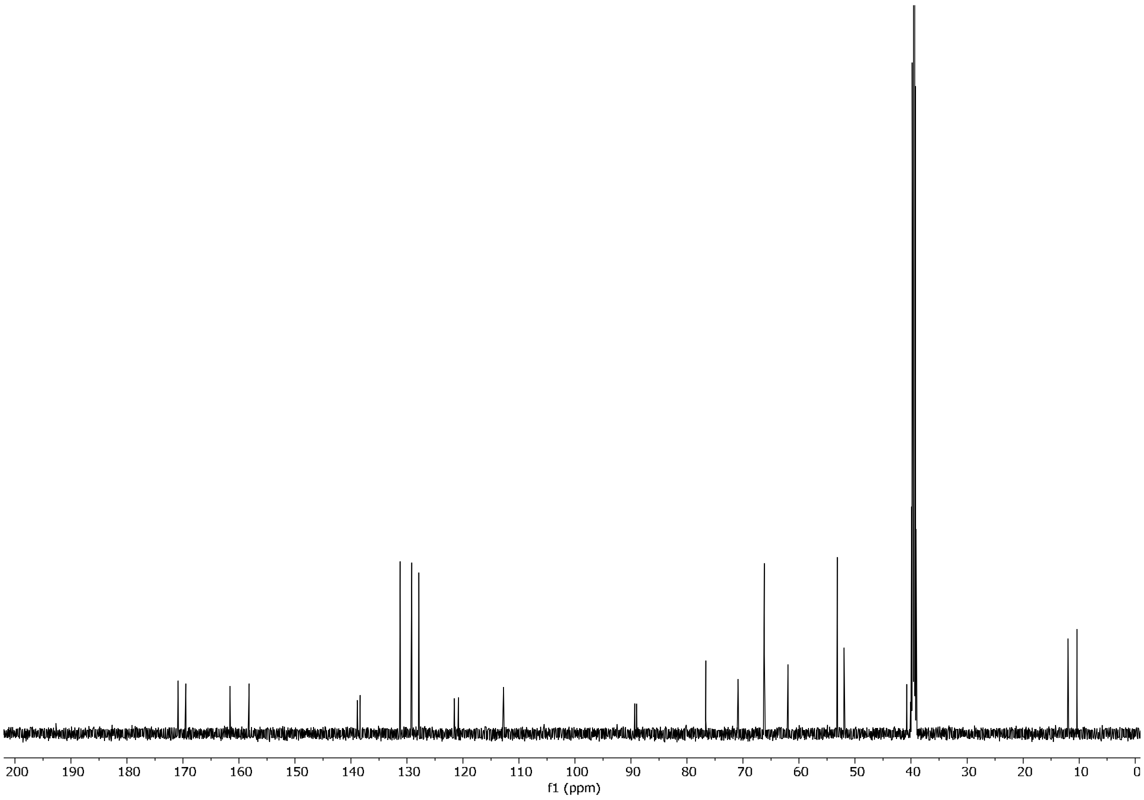

Compound (*R*)-**13a**

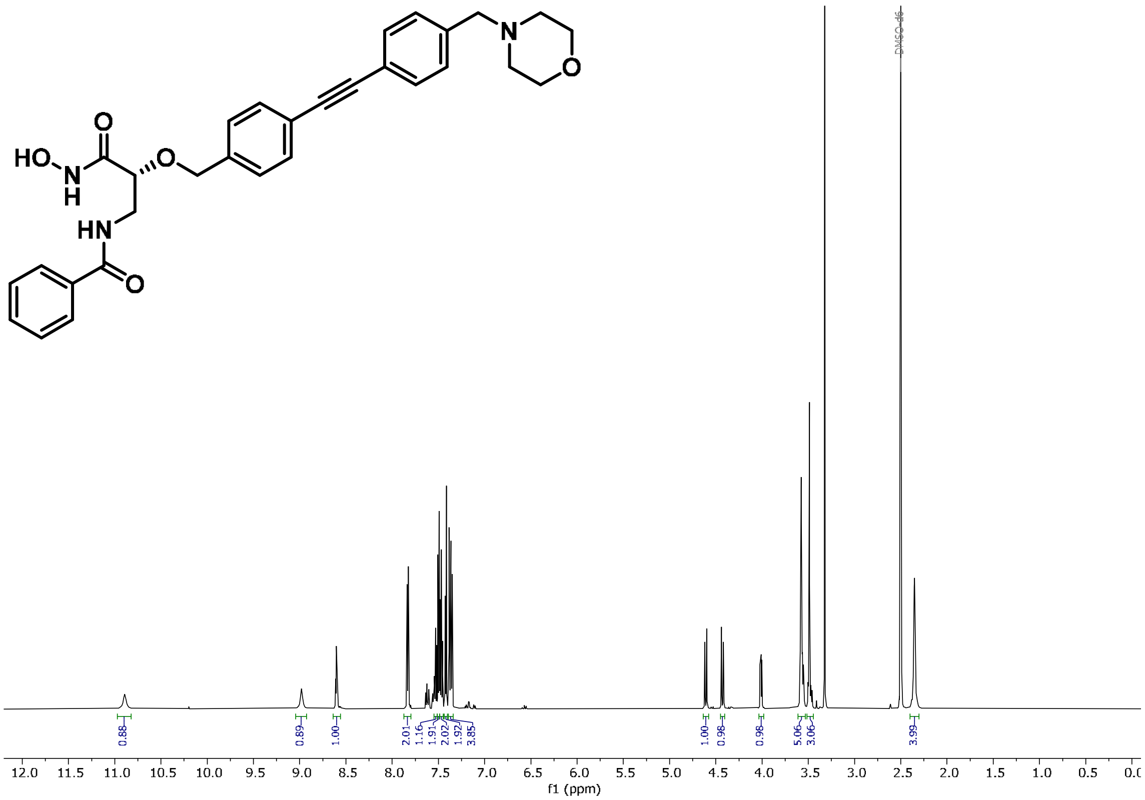

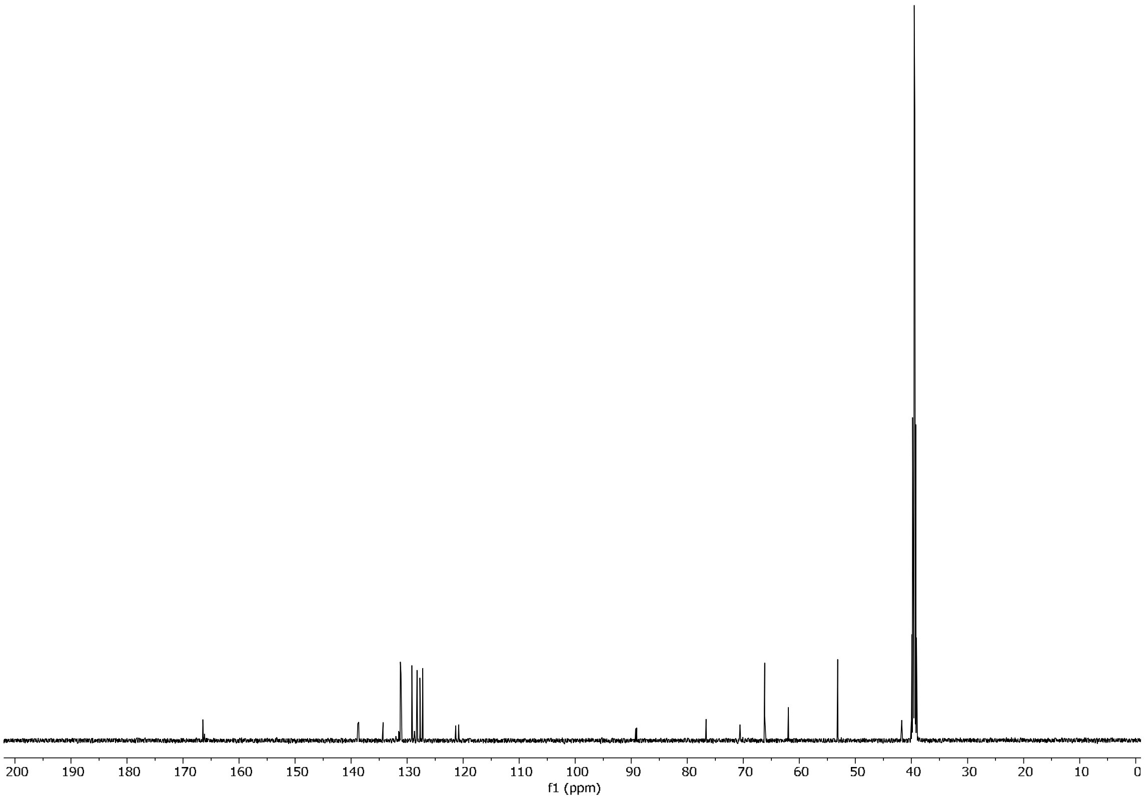

Compound (*R*)-**13b**

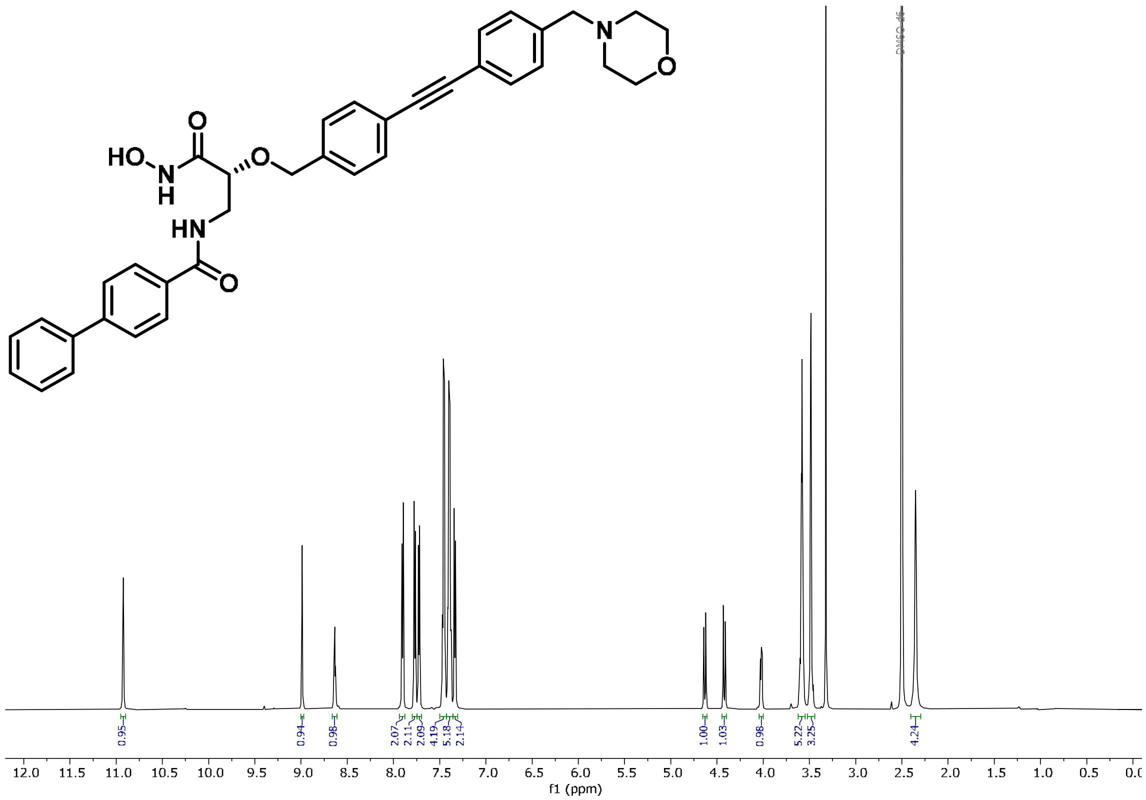

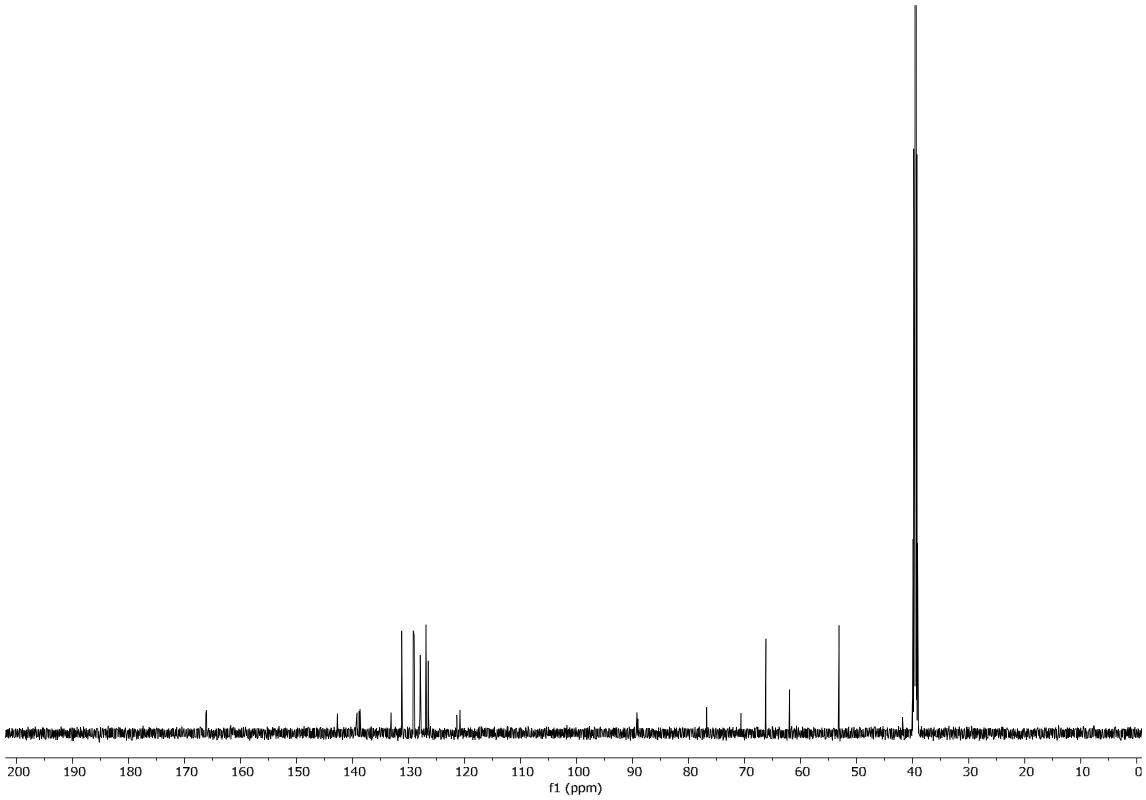

Compound (*R*)-**13c**

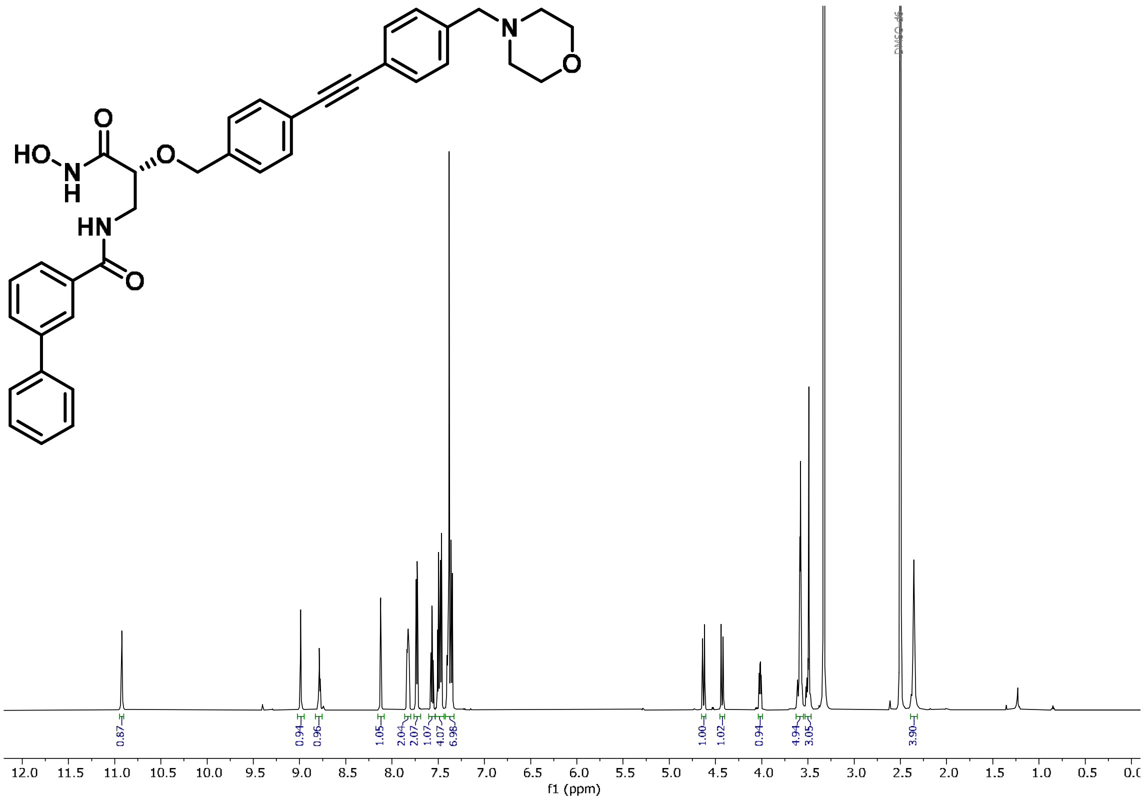

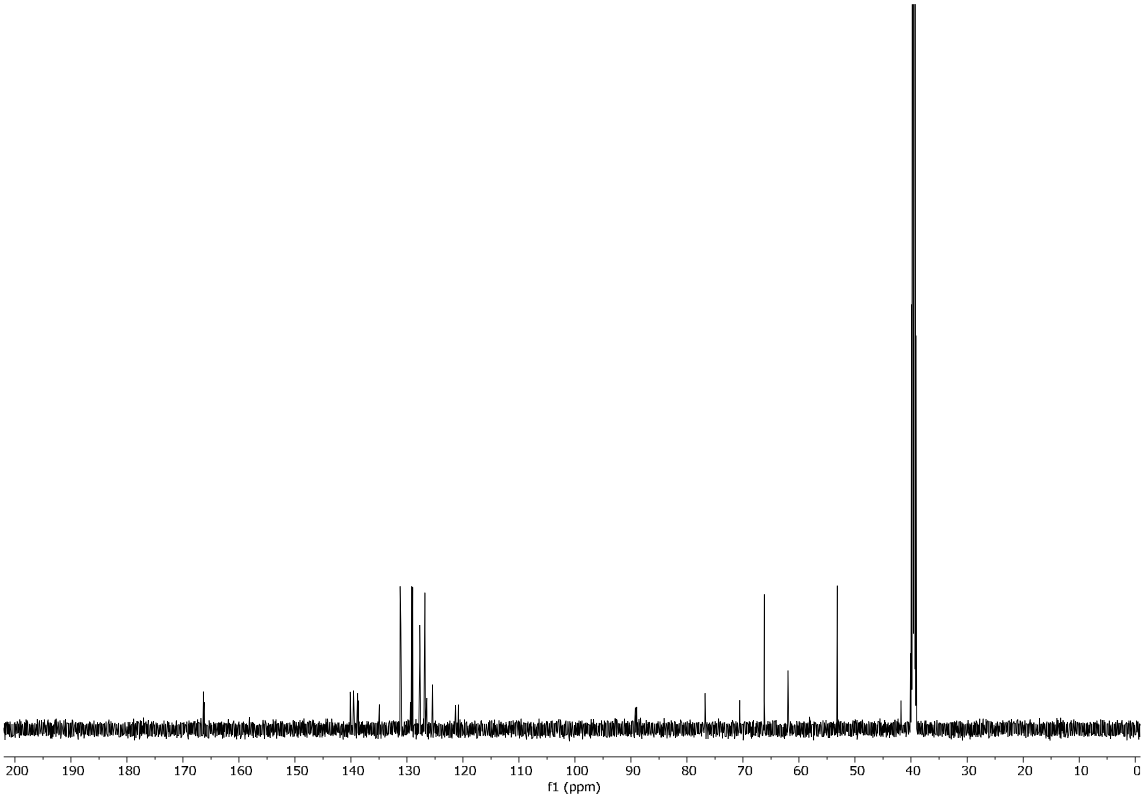

Compound (*S*)-**13d**

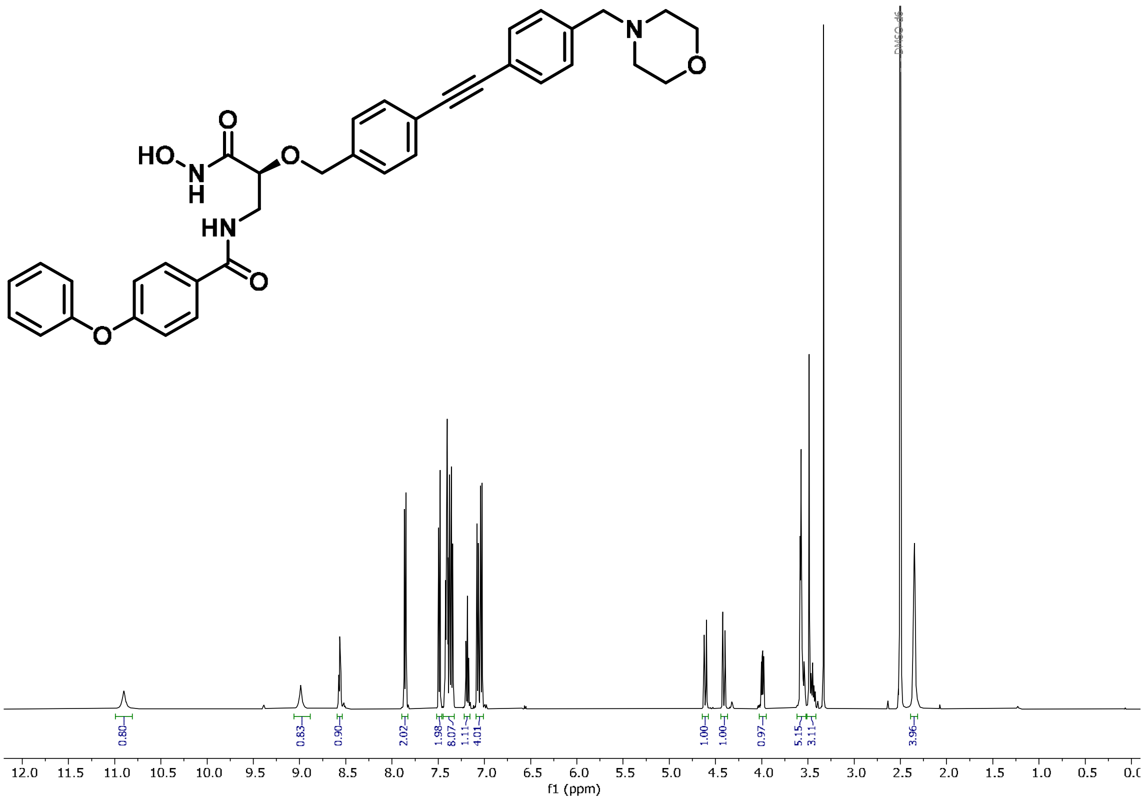

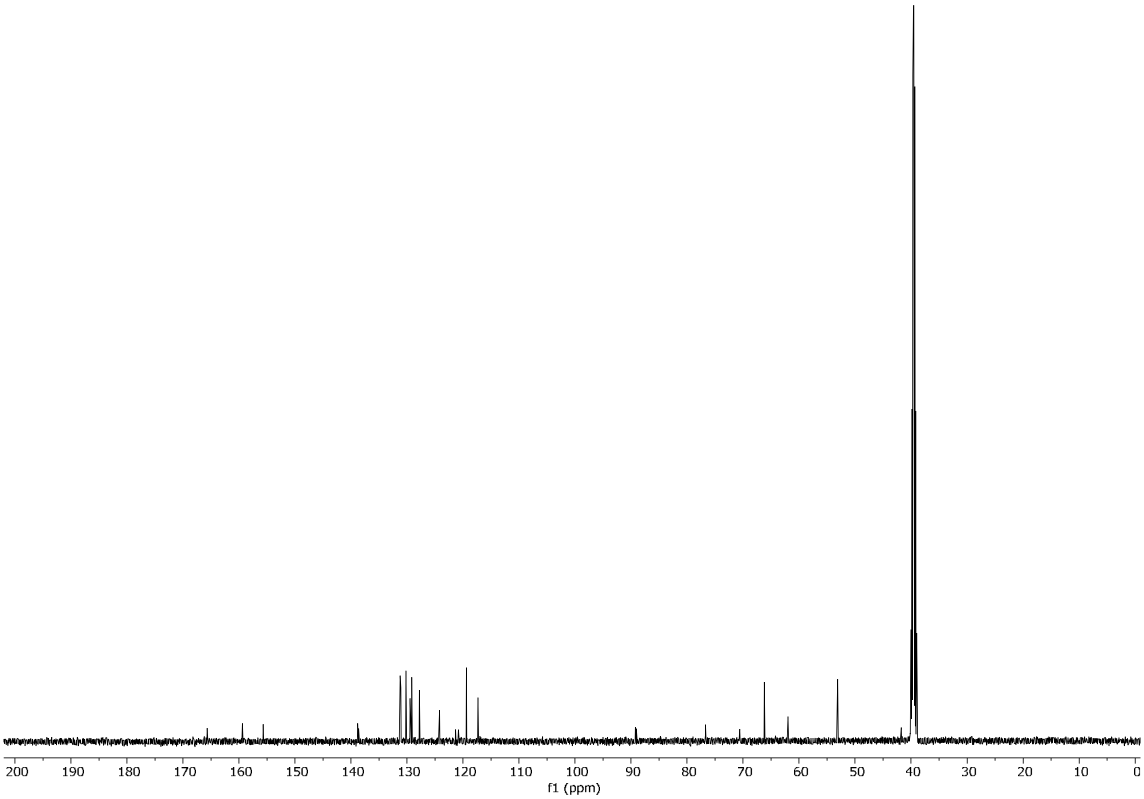

Compound (*S*)-**13e**

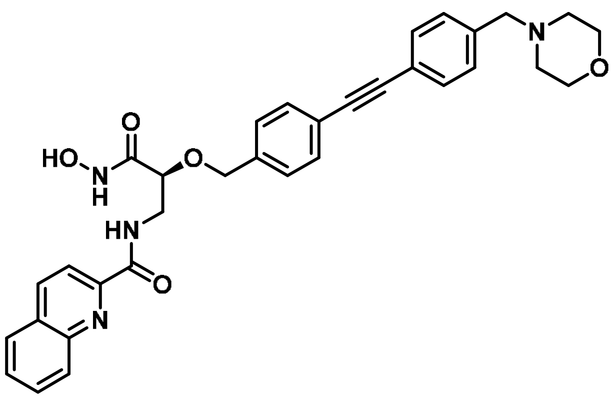

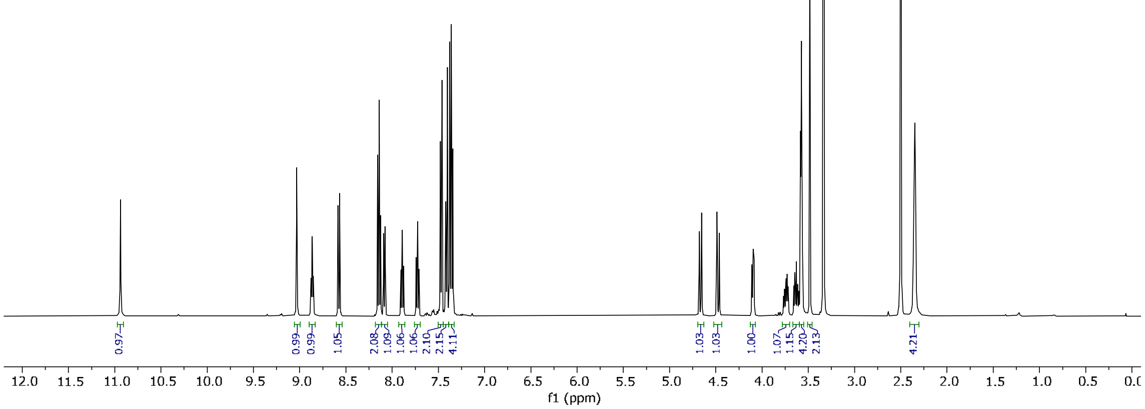

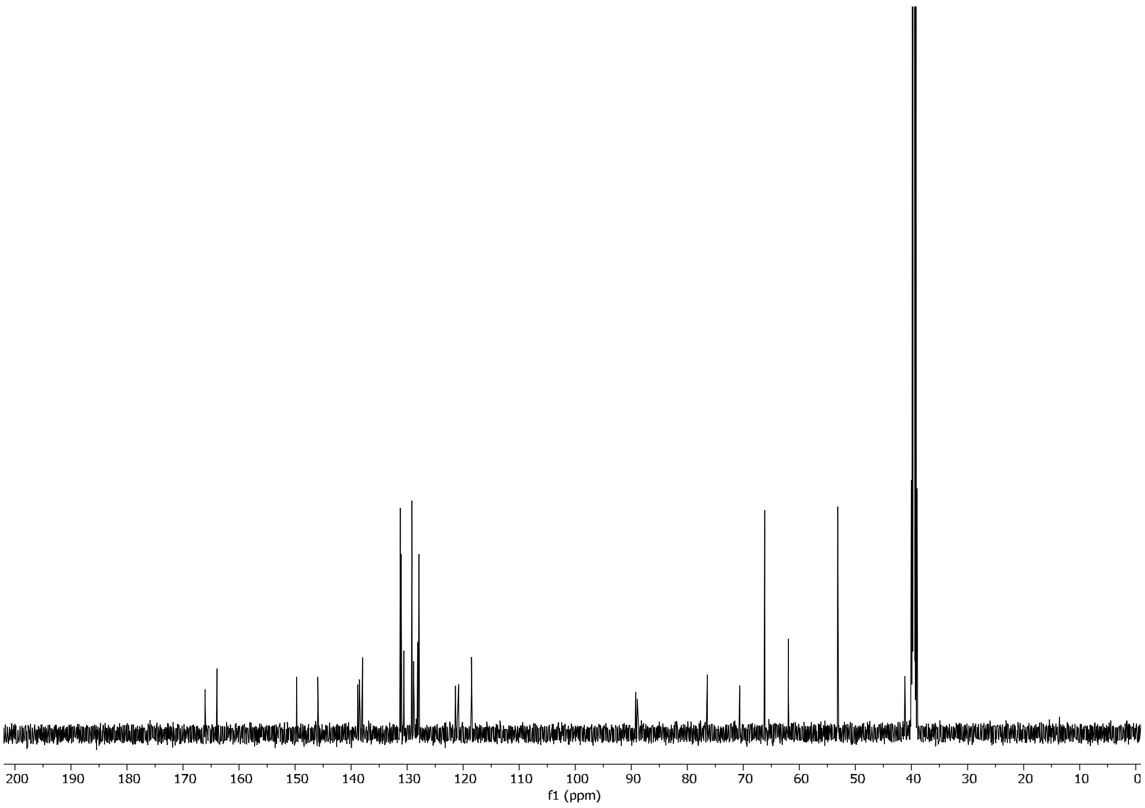

Compound (*S*)-**13f**

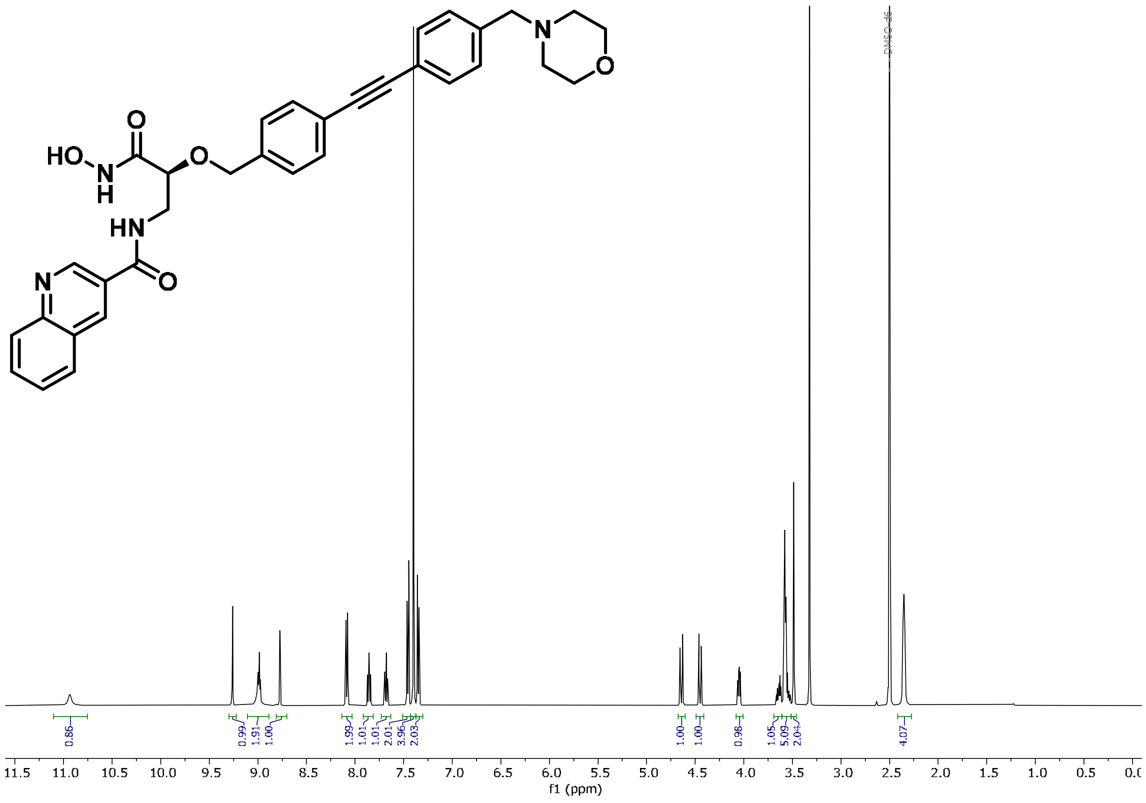

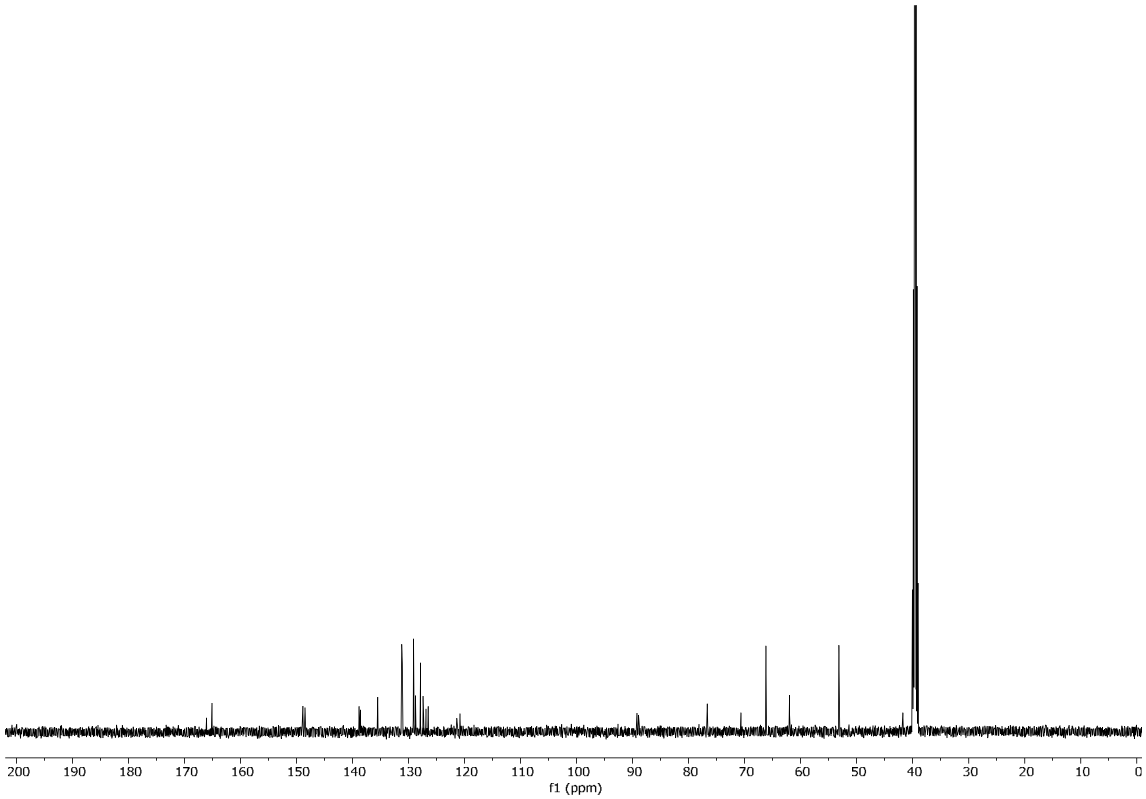

Compound (*S*)-**13g**

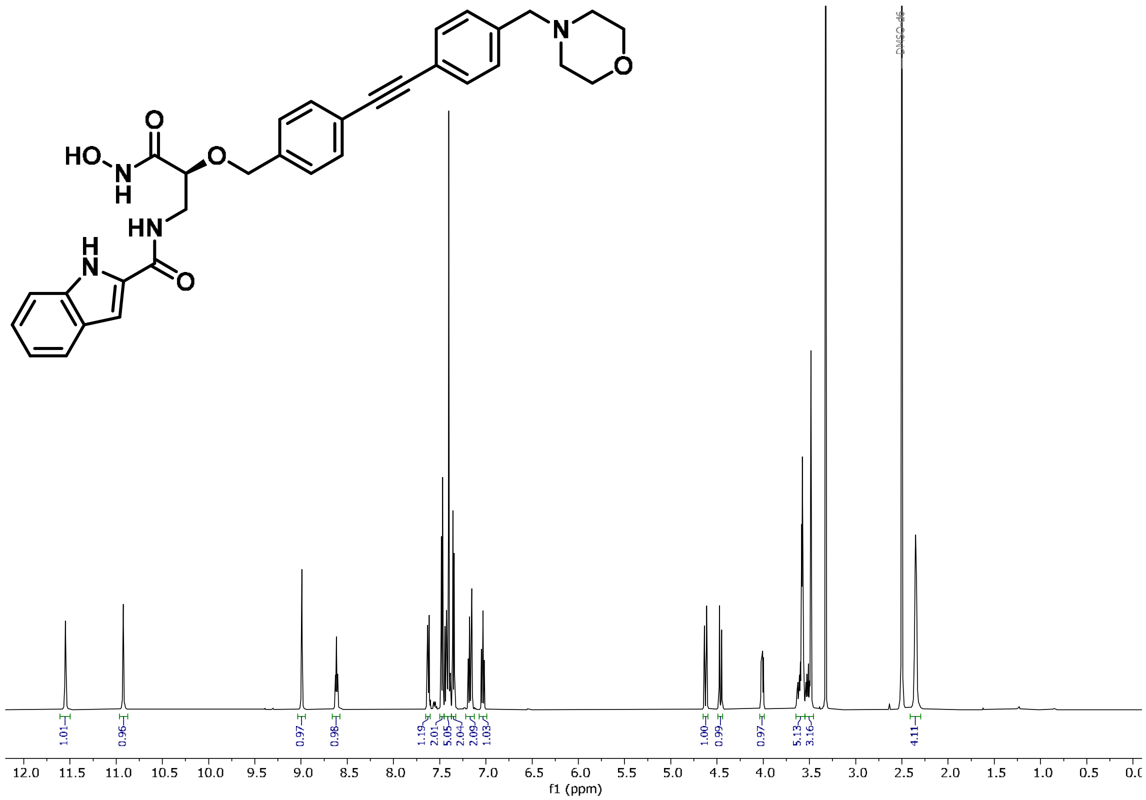

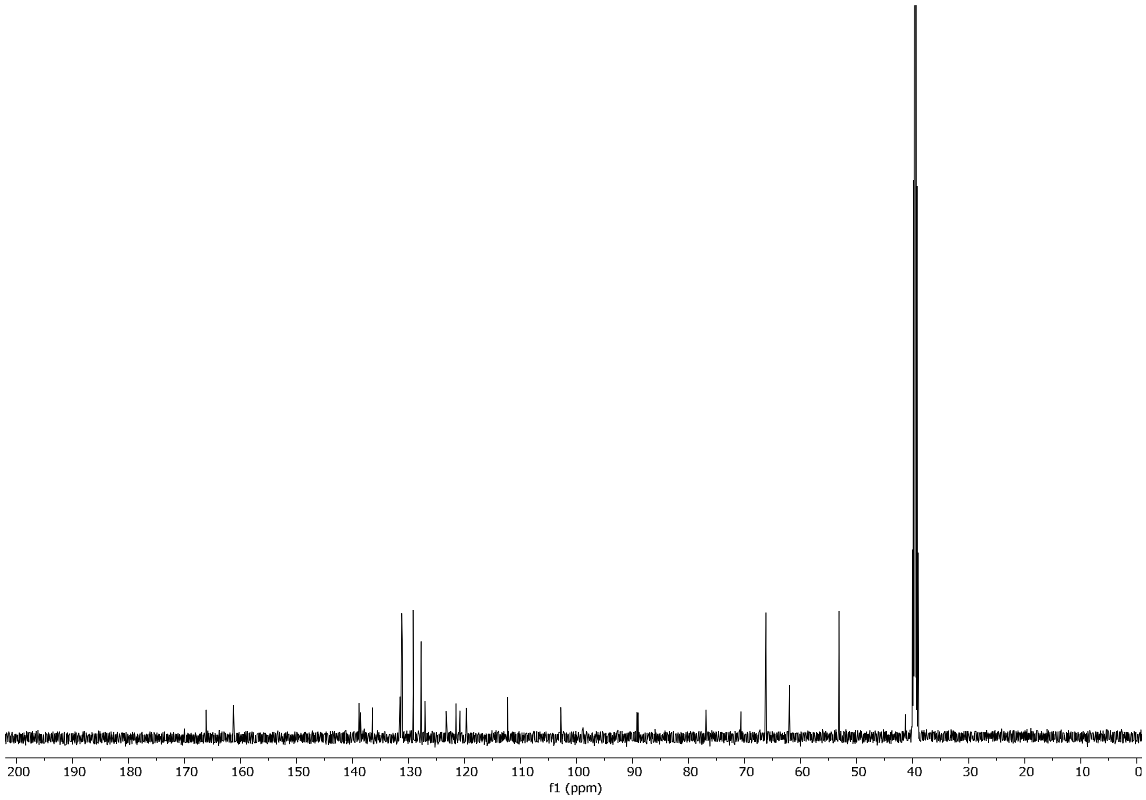

Compound (*S*)-**13h**

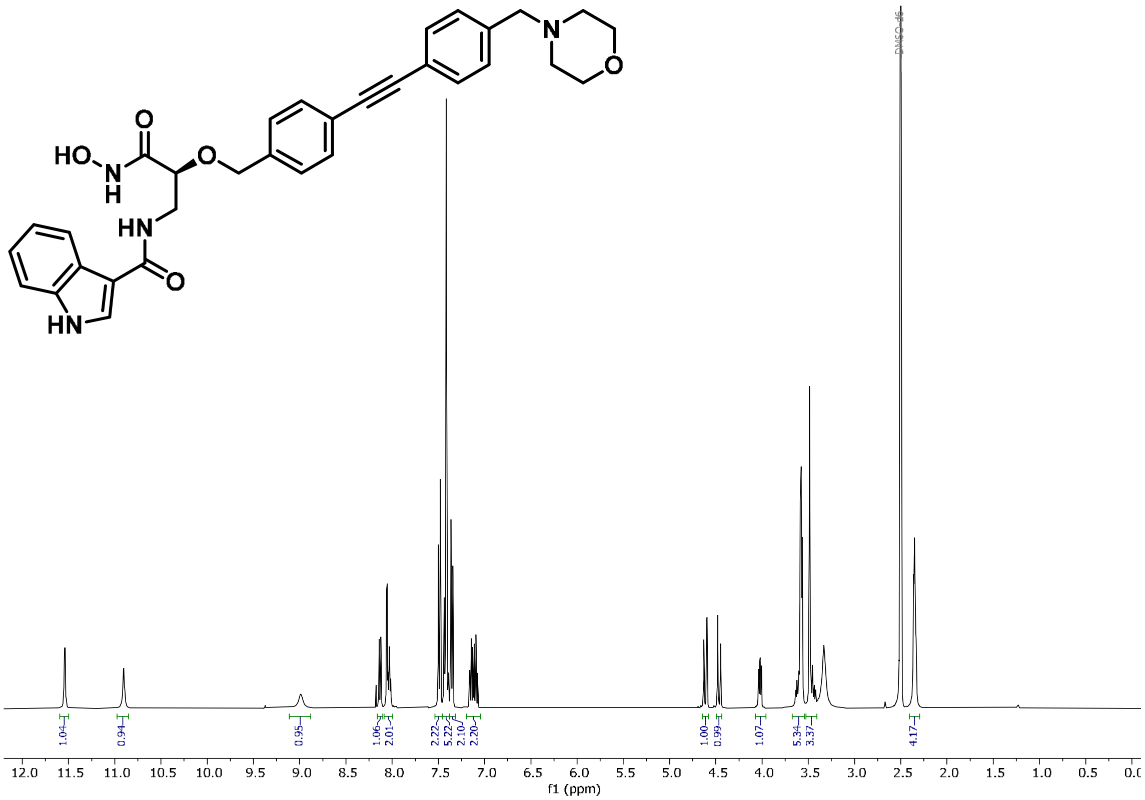

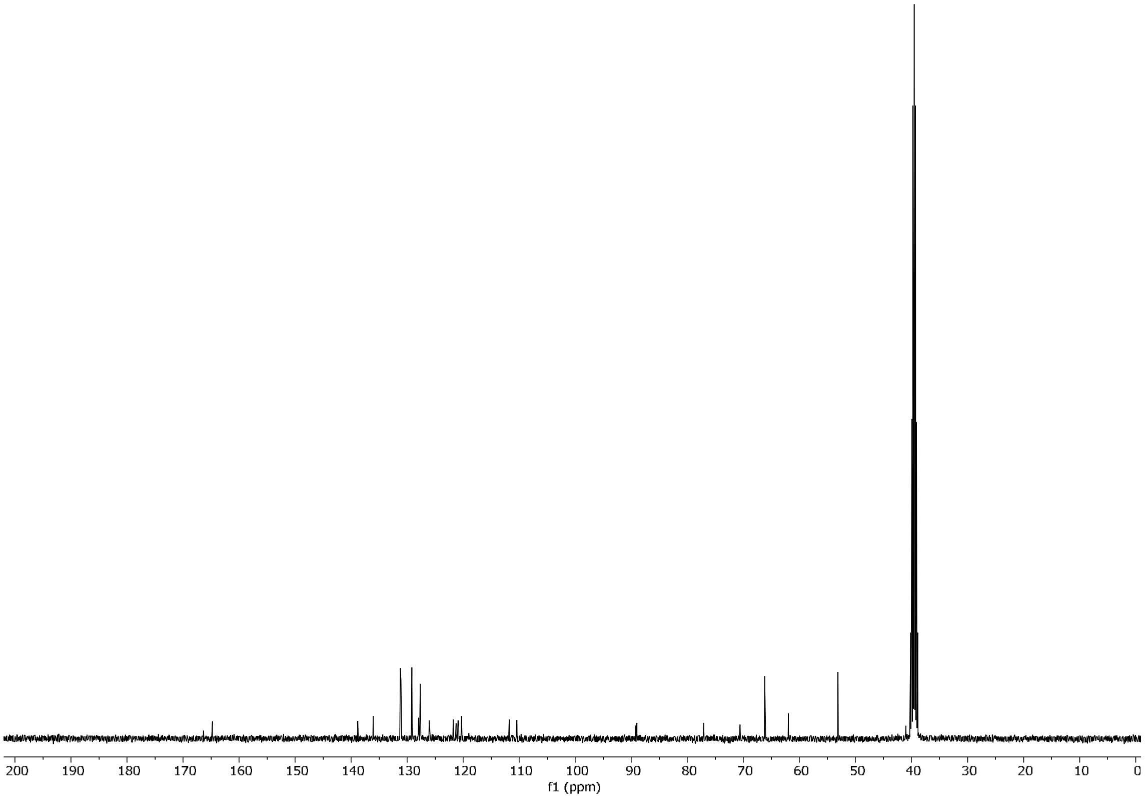

Compound (*S*)-**13i**

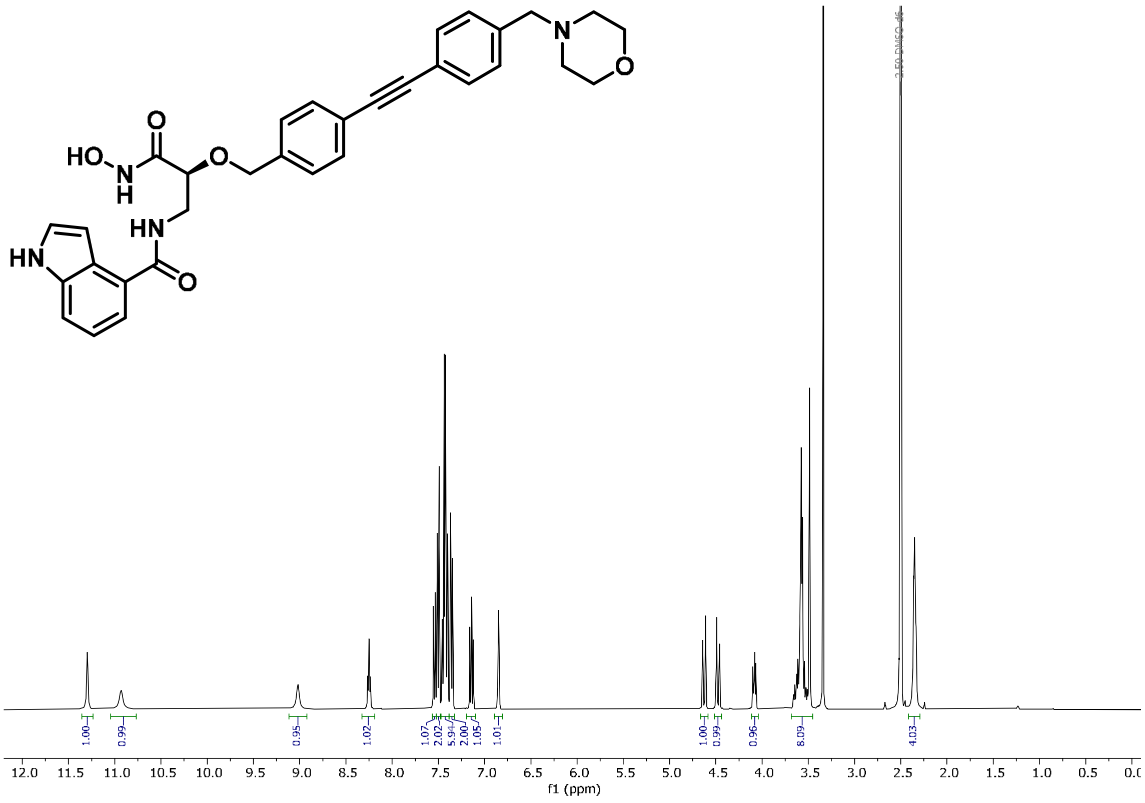

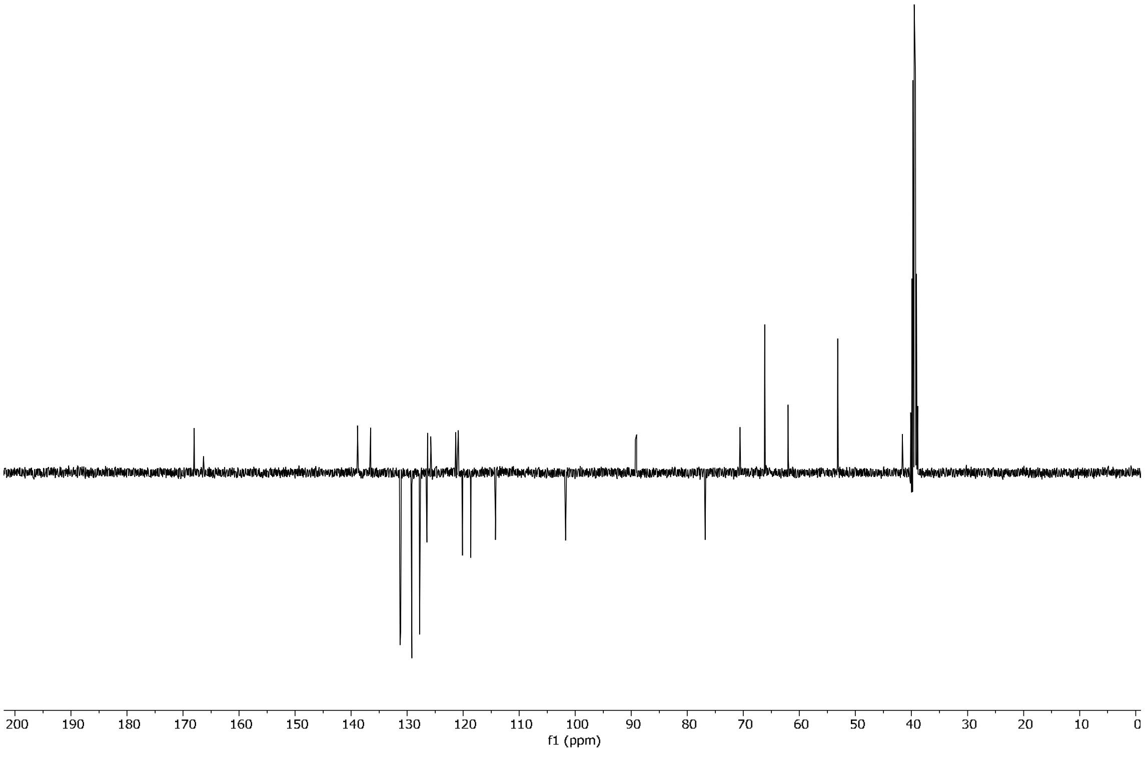

Compound (*R*)-**13j**

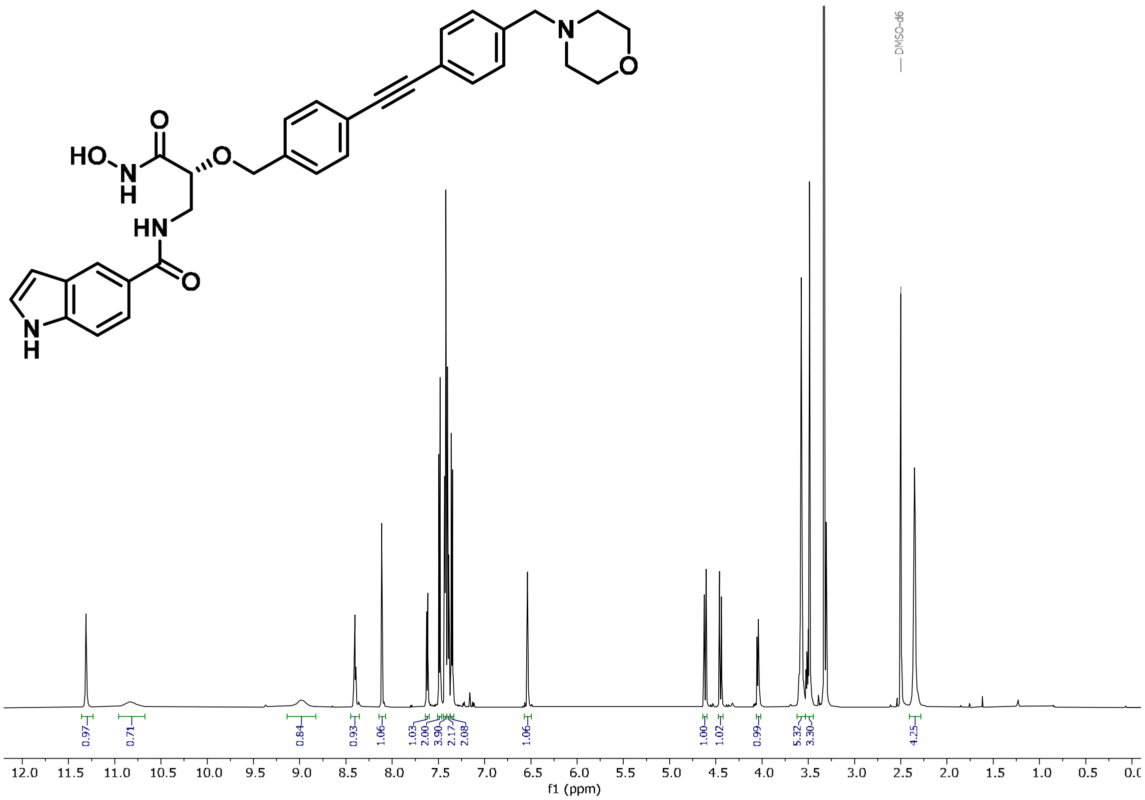

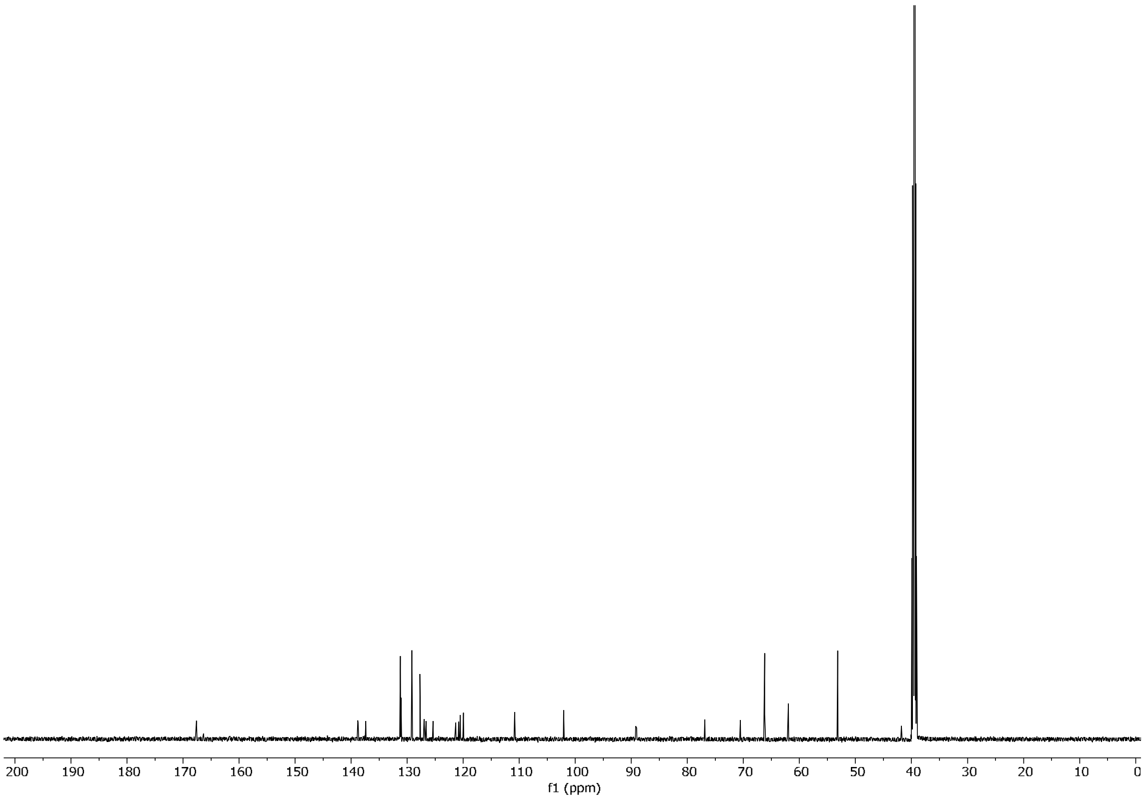

Compound (*S*)-**13k**

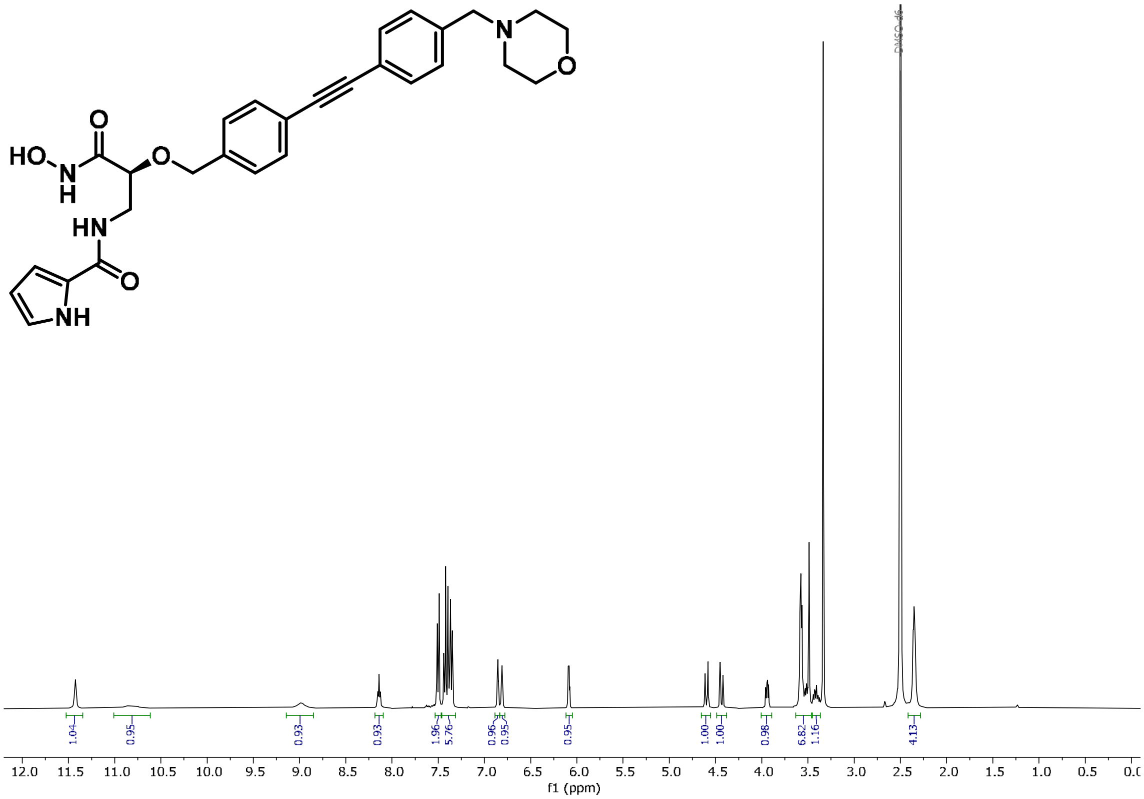

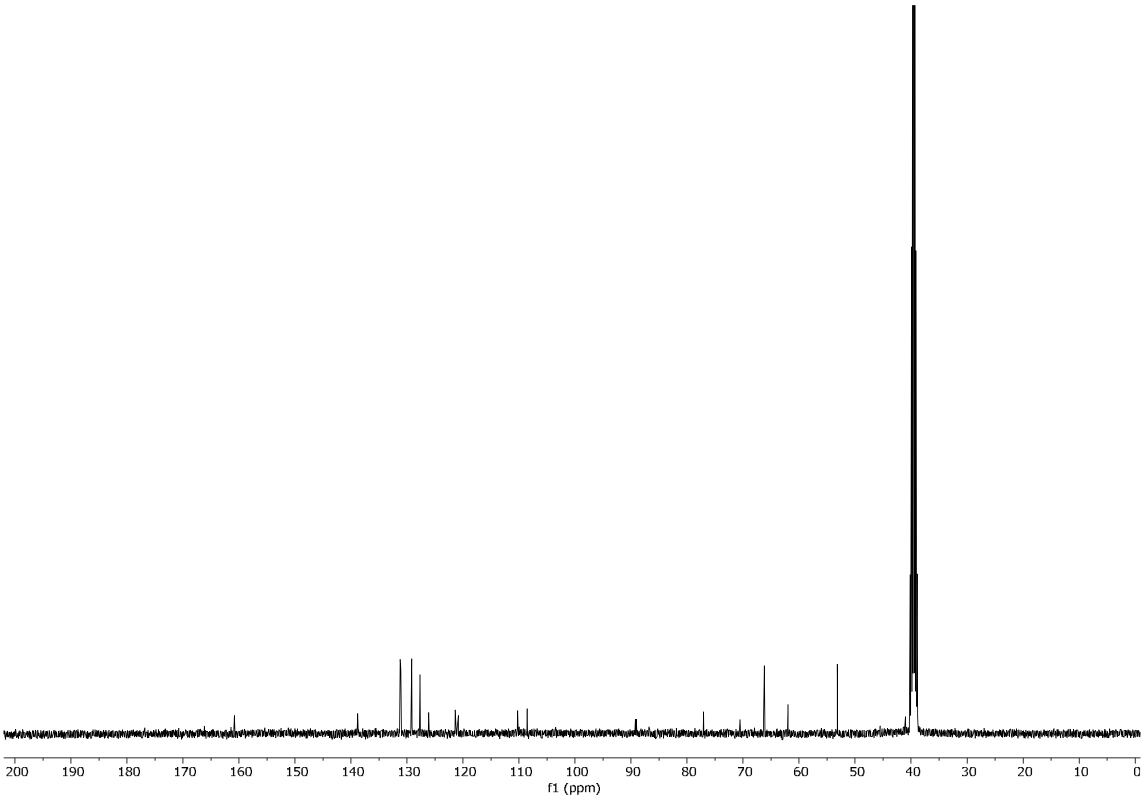

Compound (*S*)-**13l**

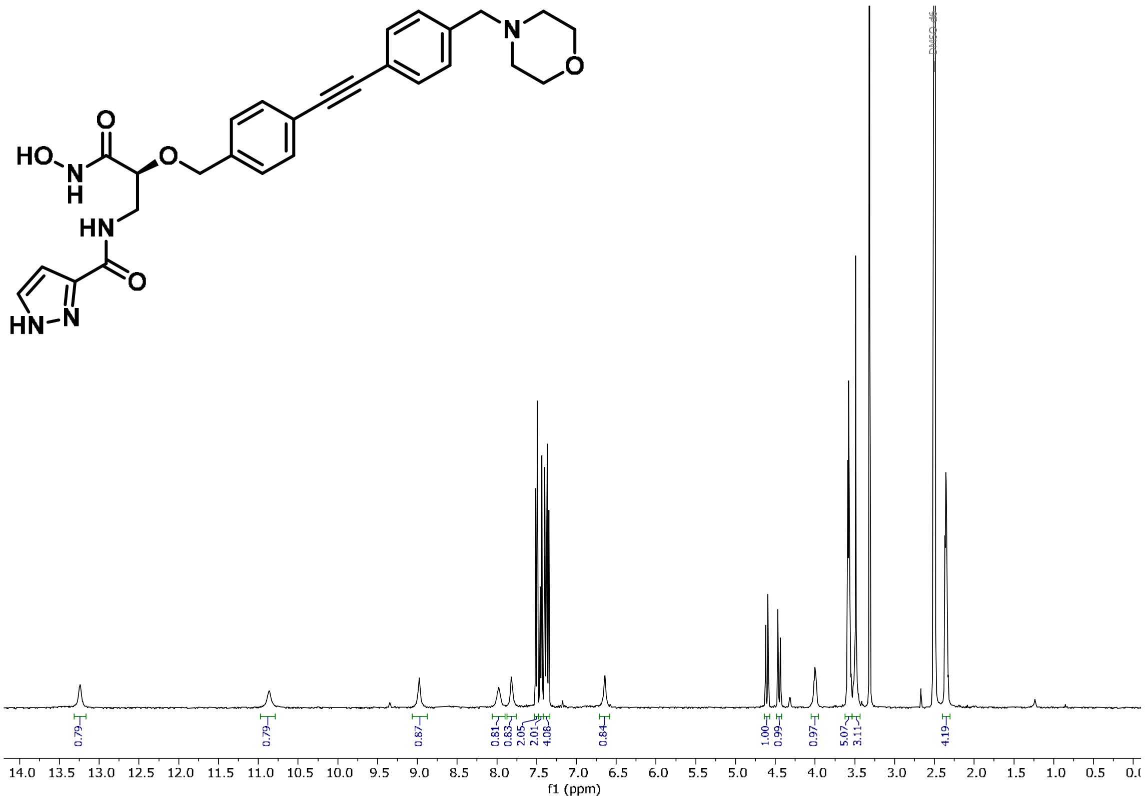

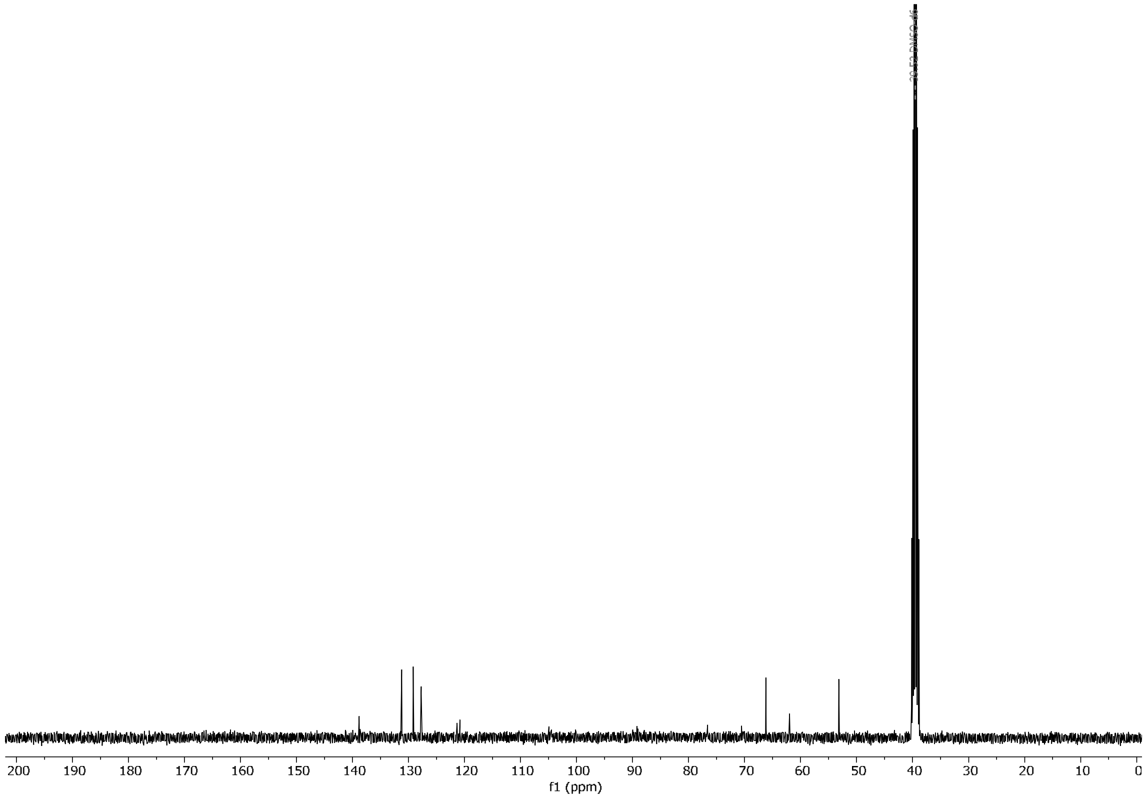

Compound (*S*)-**13m**

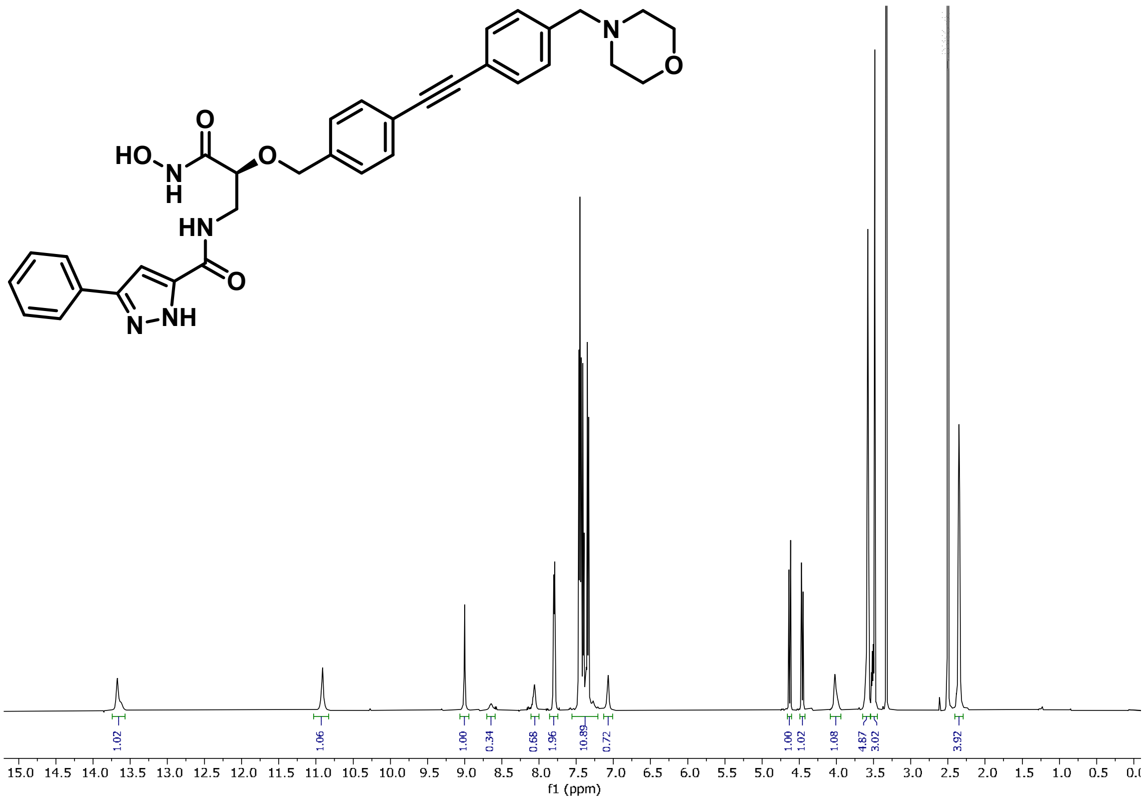

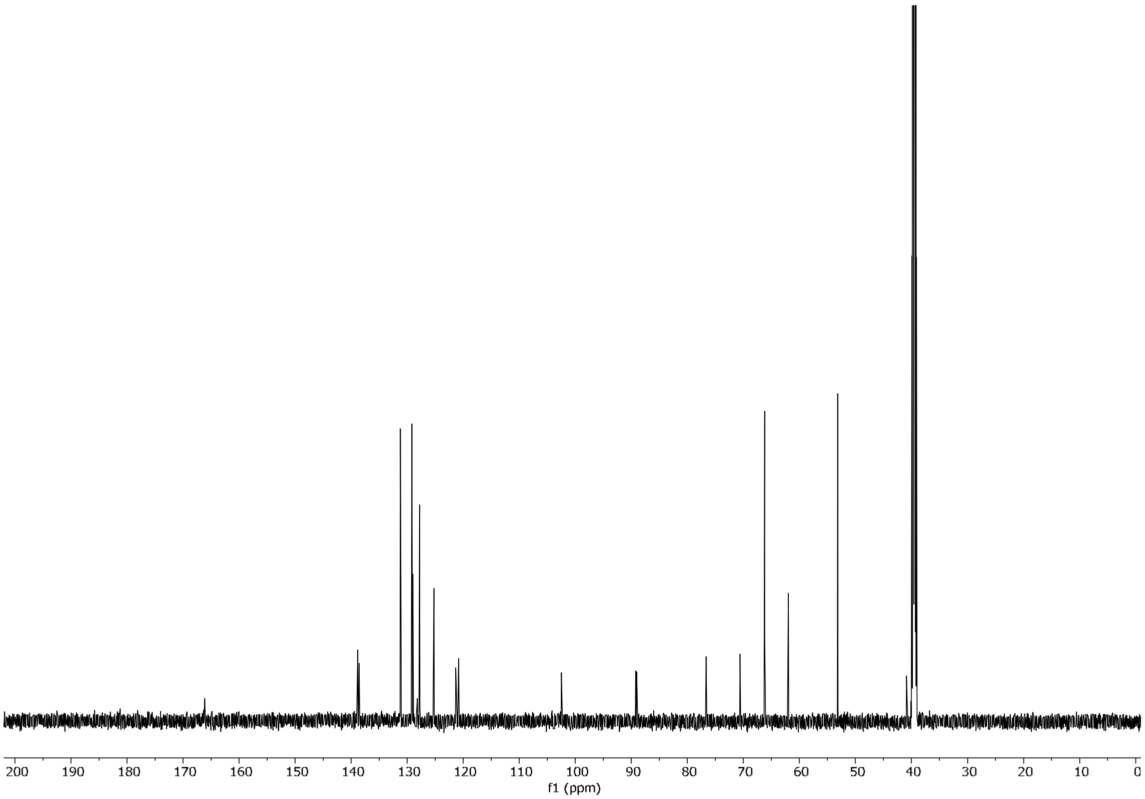

Compound (*S*)-**13n**

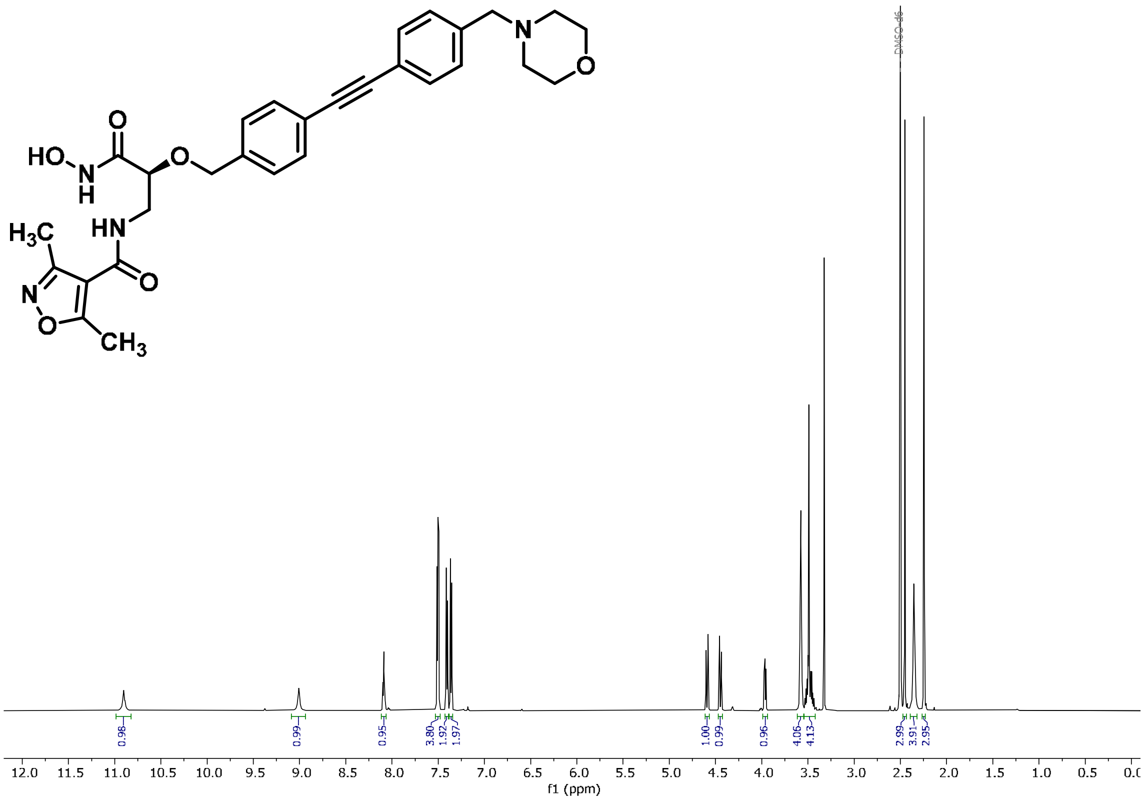

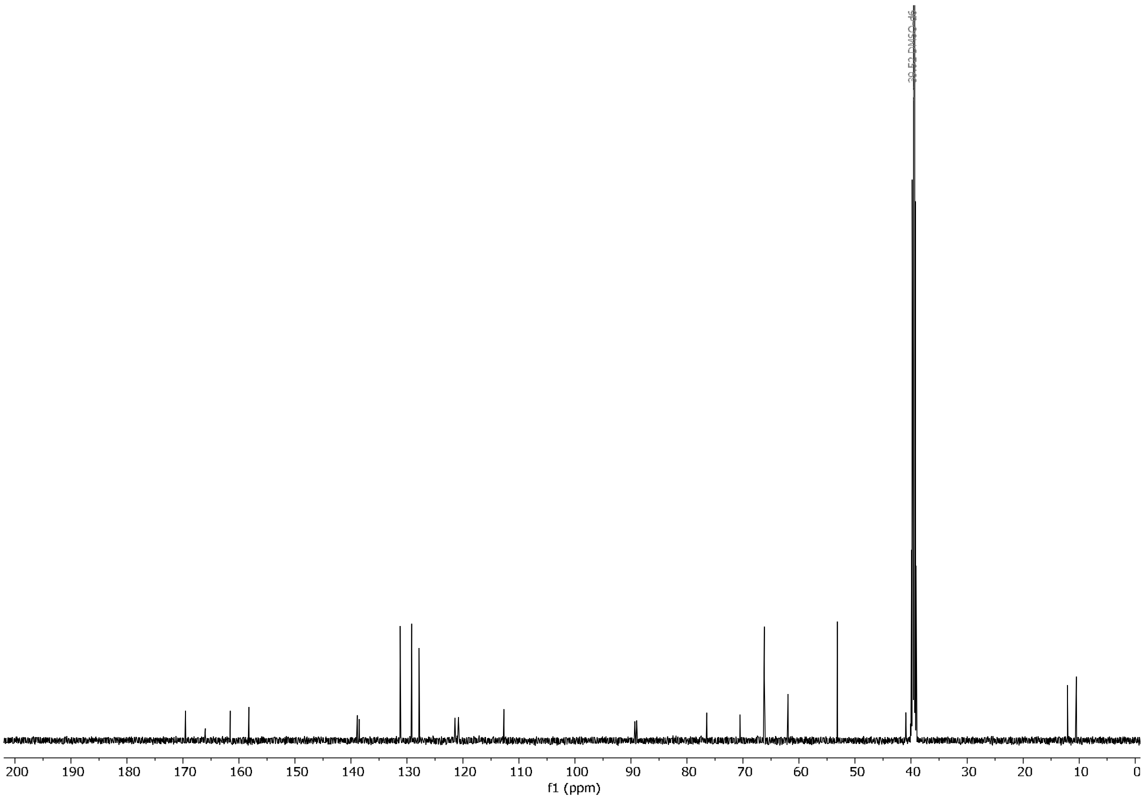

HPLC chromatograms of the test compounds

(*S*)-**11a**

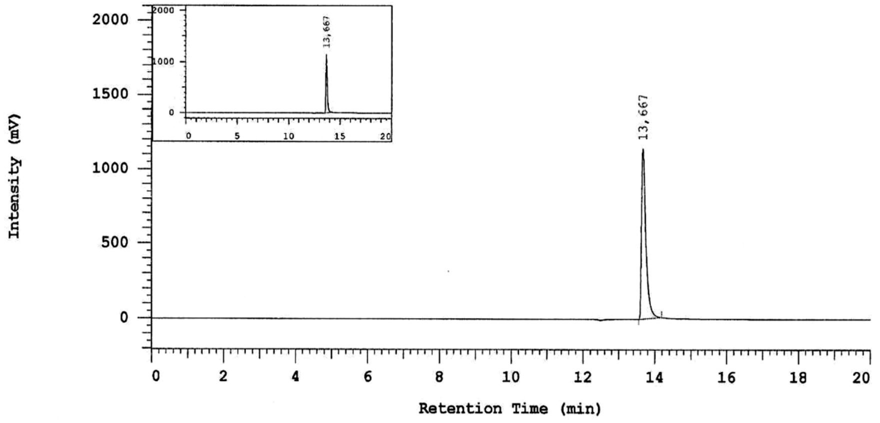

No.	RT	Area	Conc 1	BC
1	13,667	9395614	100,000	BB
		9395614	100,000	

(*R*)-**11a**

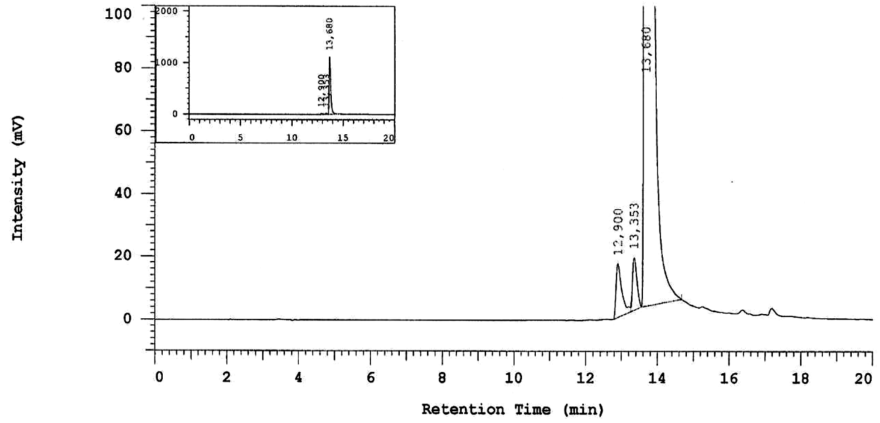

No.	RT	Area	Conc 1	BC
1	12,900	187490	1,649	BV
2	13,353	138636	1,219	VB
3	13,680	11043933	97,132	MC
		11370059	100,000	

(*S*)-**12a**

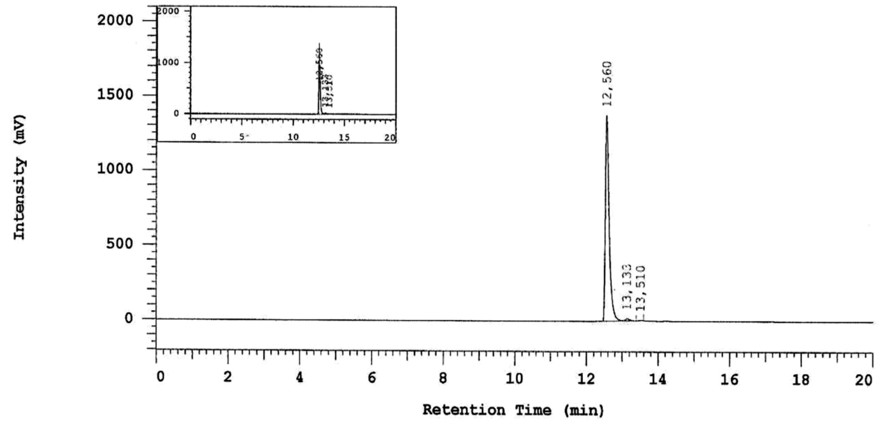

No.	RT	Area	Conc 1	BC
1	12,560	9832768	98,800	BV
2	13,133	101182	1,017	TBB
3	13,510	18291	0,184	BB
		9952241	100,000	

(*R*)-**12a**

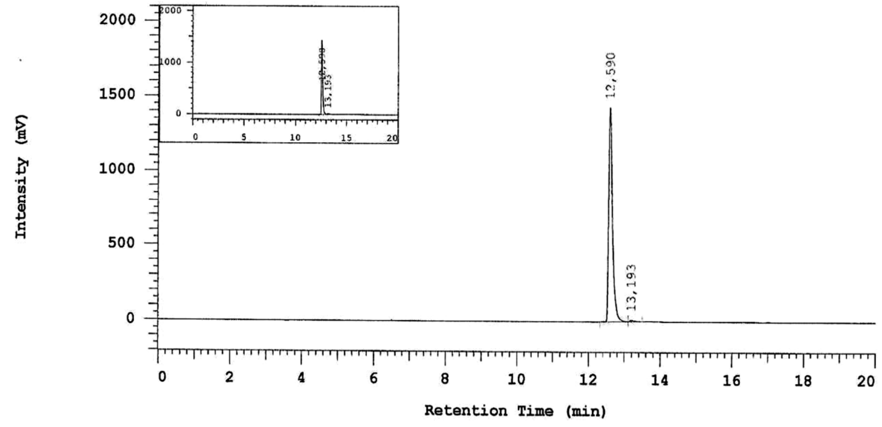

No.	RT	Area	Conc 1	BC
1	12,590	10241715	99,230	BV
2	13,193	79520	0,770	TBB
		10321235	100,000	

(*S*)-**37**

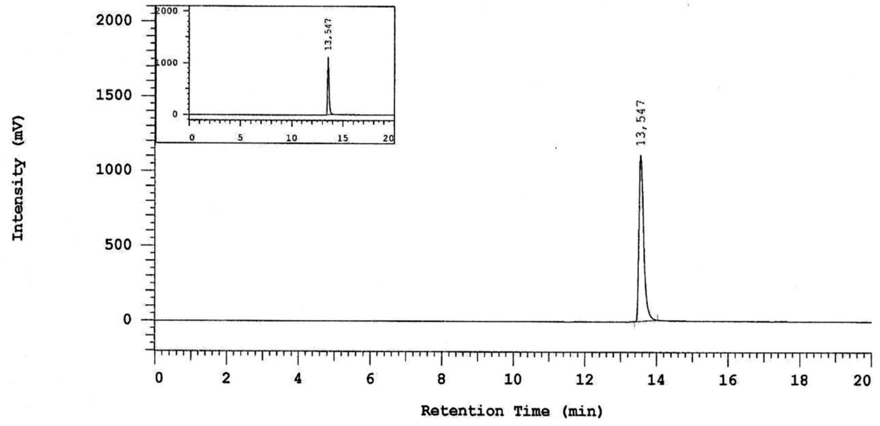

No.	RT	Area	Conc 1	BC
1	13,547	10008191	100,000	BB
		10008191	100,000	

(*R*)-**37**

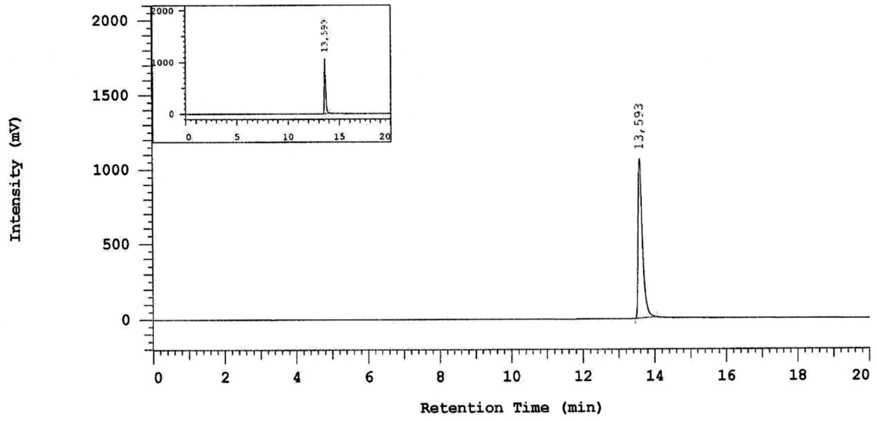

No.	RT	Area	Conc 1	BC
1	13,593	8769599	100,000	BB
		8769599	100,000	

(*S*)-**13a**

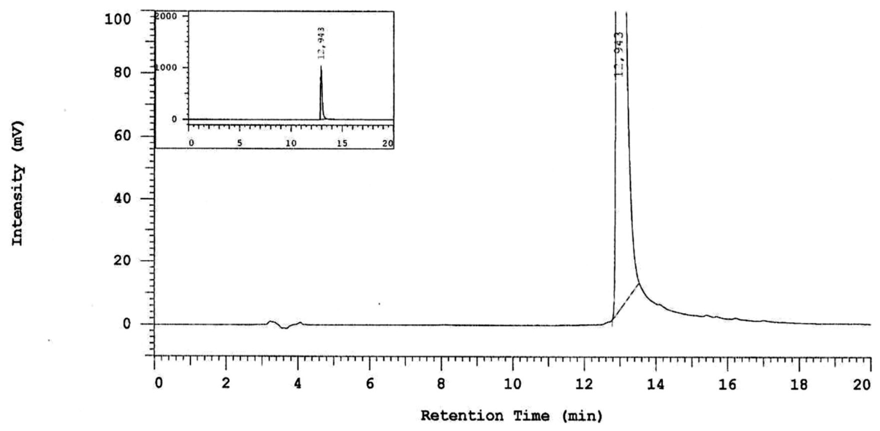

No.	RT	Area	Conc 1	BC
1	12,943	10023566	100,000	BB
		10023566	100,000	

(*R*)-**13a**

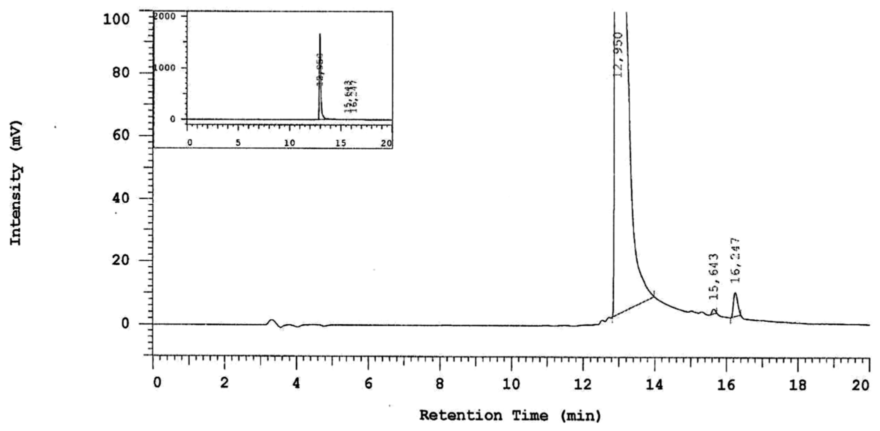

No.	RT	Area	Conc 1	BC
1	12,950	15018694	99,553	MC
2	15,643	8653	0,057	BB
3	16,247	58820	0,390	BB
		15086167	100,000	

(*S*)-**13b**

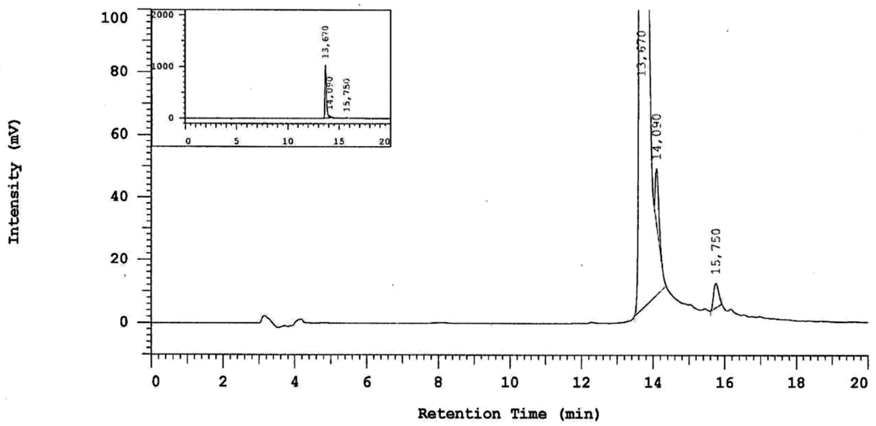

No.	RT	Area	Conc 1	BC
1	13,670	9476283	98,021	BV
2	14,090	118538	1,226	TBB
3	15,750	72735	0,752	BB
		9667556	100,000	

(*R*)-**13b**

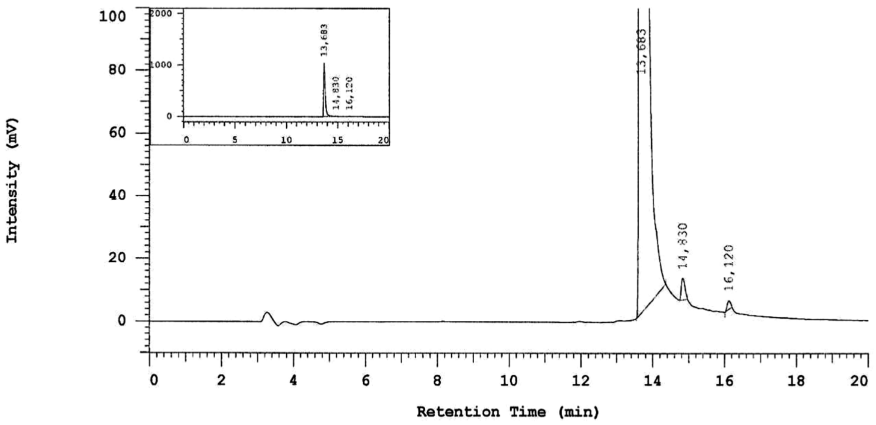

No.	RT	Area	Conc 1	BC
1	13,683	10001844	99,367	MC
2	14,830	46415	0,461	BB
3	16,120	17317	0,172	BB
		10065576	100,000	

(*S*)-**13c**

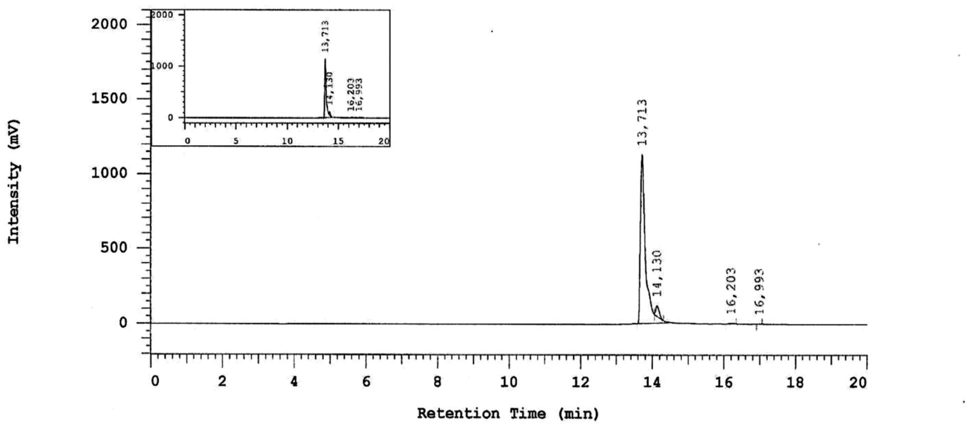

No.	RT	Area	Conc 1	BC
1	13,713	11166306	95,539	BV
2	14,130	474081	4,056	TBB
3	16,203	42025	0,360	BB
4	16,993	5237	0,045	BB
		11687649	100,000	

(*R*)-**13c**

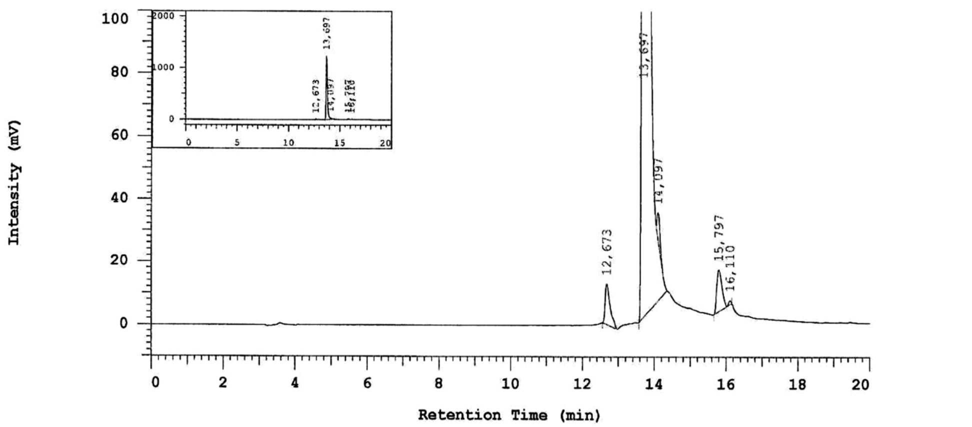

No.	RT	Area	Conc 1	BC
1	12,673	121501	1,159	BB
2	13,697	10176190	97,045	BV
3	14,097	51481	0,491	TBB
4	15,797	130933	1,249	BB
5	16,110	5964	0,057	BB
		10486069	100,000	

(*S*)-**13d**

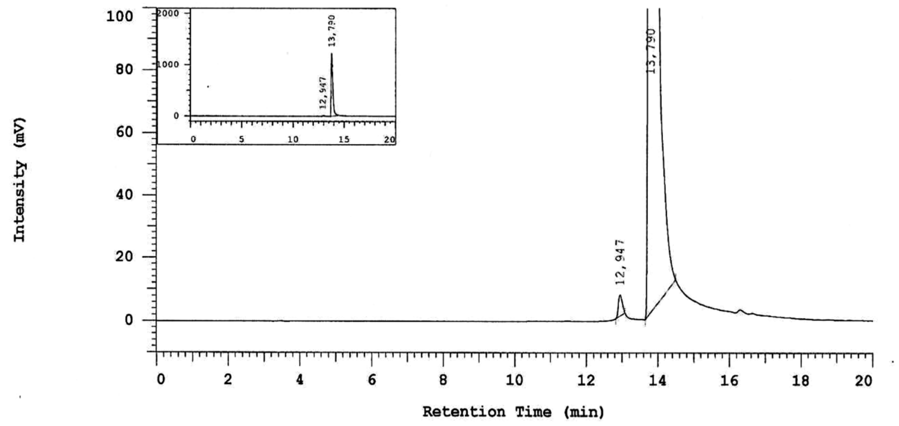

No.	RT	Area	Conc 1	BC
1	12,947	48149	0,373	BB
2	13,790	12877068	99,627	BB
		12925217	100,000	

(*R*)-**13d**

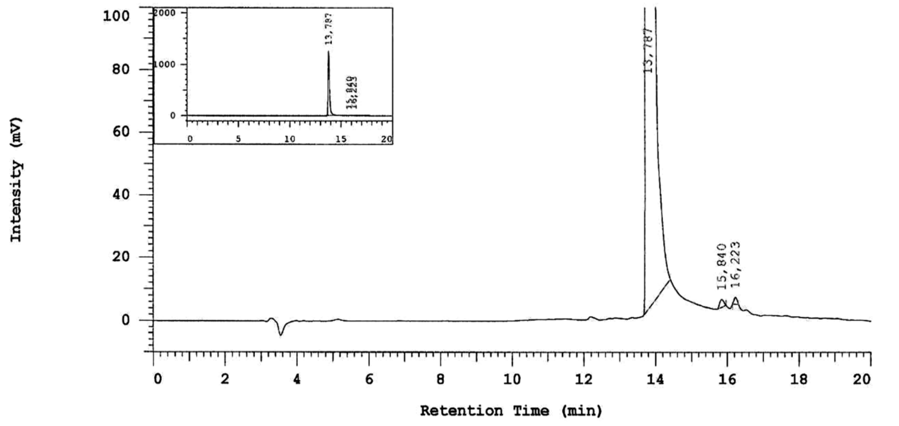

No.	RT	Area	Conc 1	BC
1	13,787	11120591	99,721	BB
2	15,840	18771	0,168	BB
3	16,223	12364	0,111	BB
		11151726	100,000	

(*S*)-**13e**

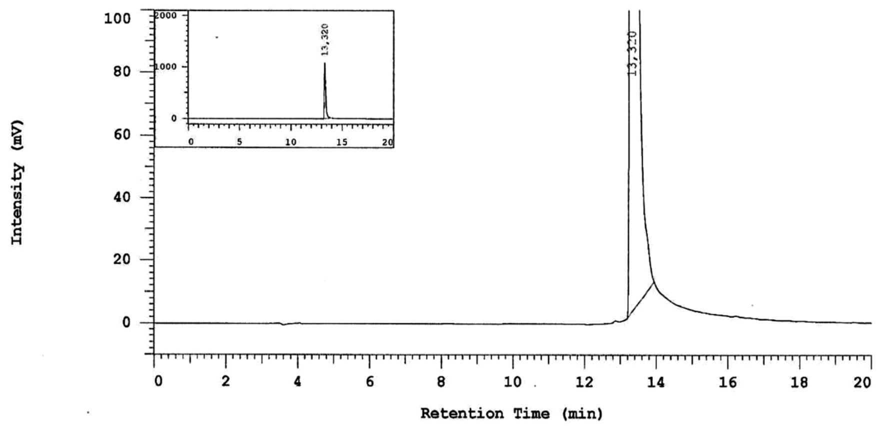

No.	RT	Area	Conc 1	BC
1	13,320	9523456	100,000	BB
		9523456	100,000	

(*R*)-**13e**

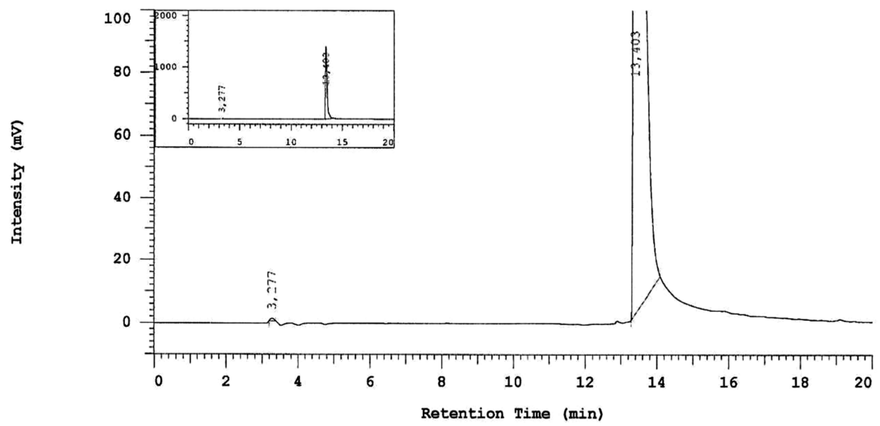

No.	RT	Area	Conc 1	BC
1	3,277	5296	0,037	BB
2	13,403	14171755	99,963	BB
		14177051	100,000	

(*S*)-**13f**

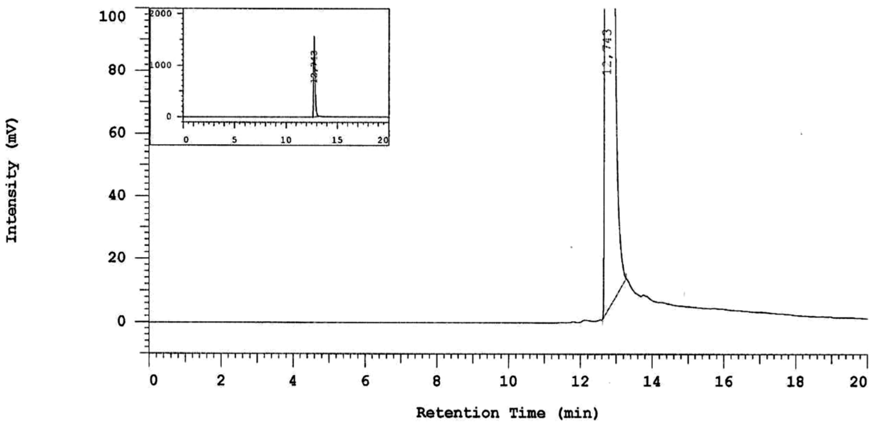

No.	RT	Area	Conc 1	BC
1	12,743	13709461	100,000	BB
		13709461	100,000	

(*R*)-**13f**

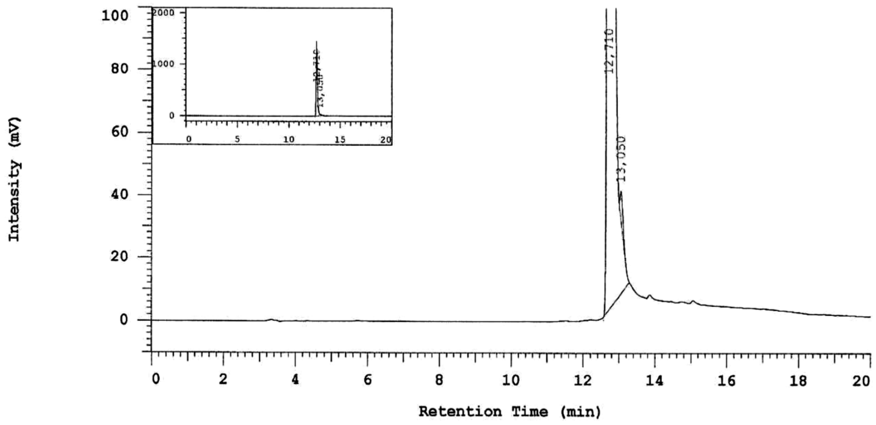

No.	RT	Area	Conc 1	BC
1	12,710	11300099	99,537	BV
2	13,050	52608	0,463	TBB
		11352707	100,000	

(*S*)-**13g**

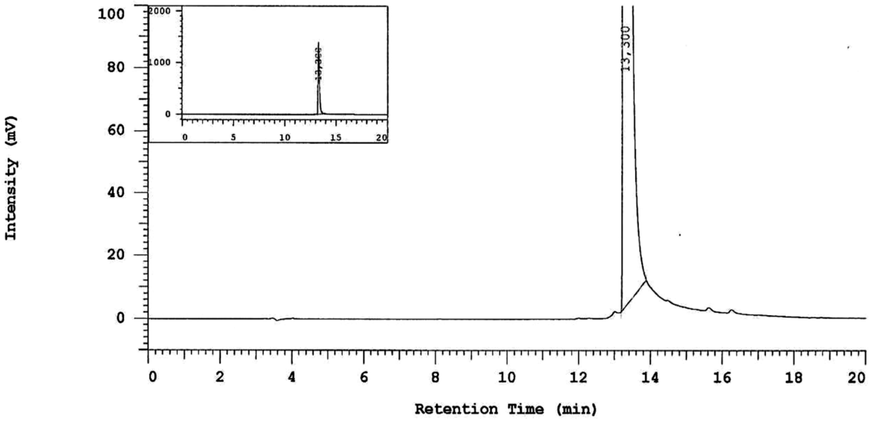

No.	RT	Area	Conc 1	BC
1	13,300	11435088	100,000	BB
		11435088	100,000	

(*R*)-**13g**

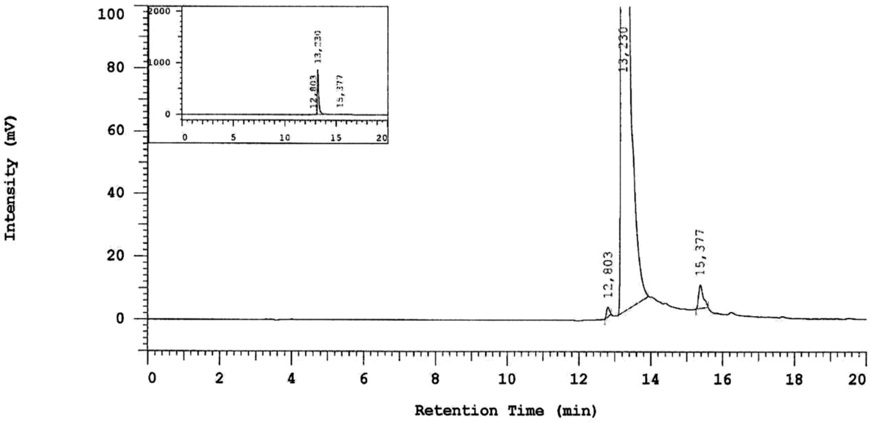

No.	RT	Area	Conc 1	BC
1	12,803	17527	0,231	MC
2	13,230	7500910	98,903	MC
3	15,377	65684	0,866	BB
		7584121	100,000	

(*S*)-**13h**

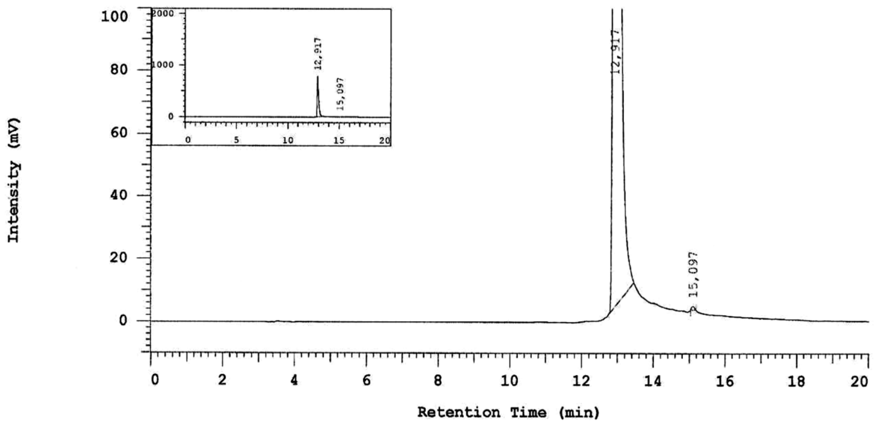

No.	RT	Area	Conc 1	BC
1	12,917	6859384	99,908	BB
2	15,097	6287	0,092	BB
		6865671	100,000	

(*R*)-**13h**

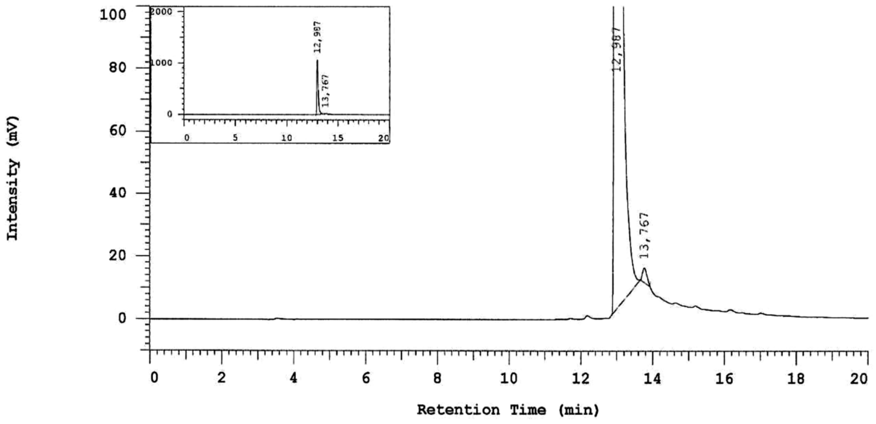

No.	RT	Area	Conc 1	BC
1	12,987	9148176	99,565	MC
2	13,767	39926	0,435	MC
		9188102	100,000	

(*S*)-**13i**

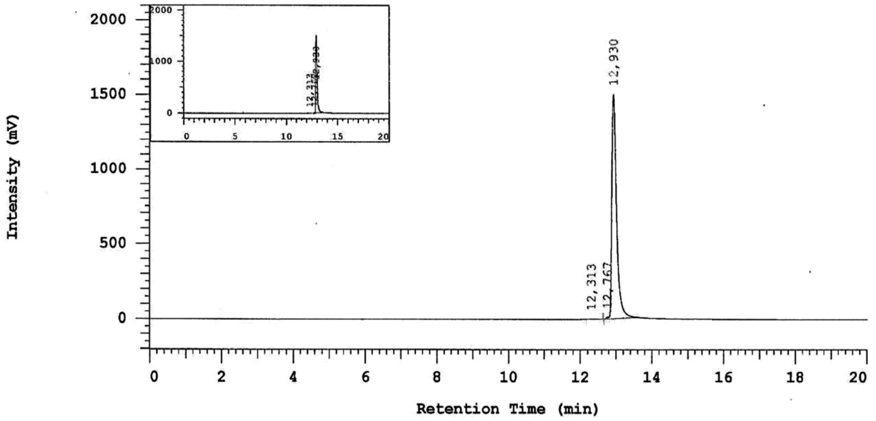

No.	RT	Area	Conc 1	BC
1	12,313	26456	0,196	BB
2	12,767	60609	0,449	BV
3	12,930	13406334	99,355	VB
		13493399	100,000	

(*S*)-**13j**

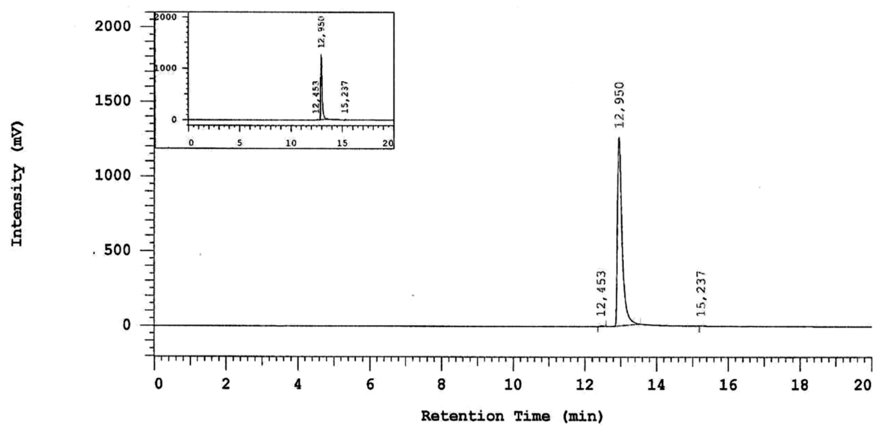

No.	RT	Area	Conc 1	BC
1	12,453	21773	0,196	BB
2	12,950	11055114	99,766	BB
3	15,237	4116	0,037	BB
		11081003	100,000	

(*R*)-**13j**

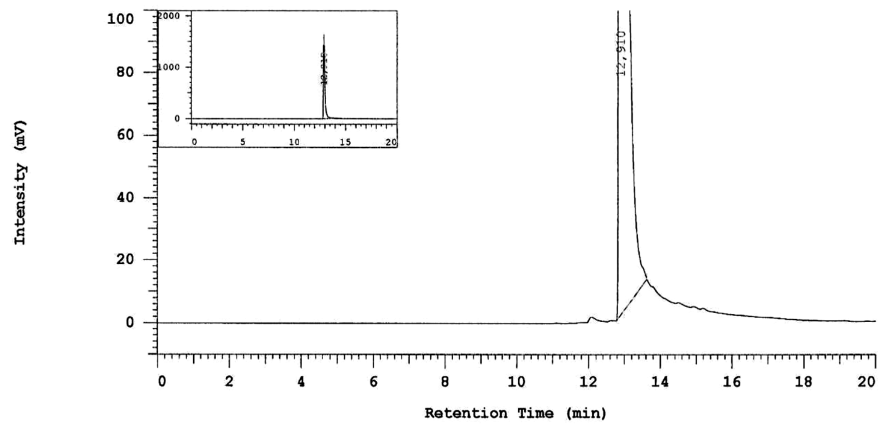

No.	RT	Area	Conc 1	BC
1	12,910	13865646	100,000	BB
		13865646	100,000	

(*S*)-**13k**

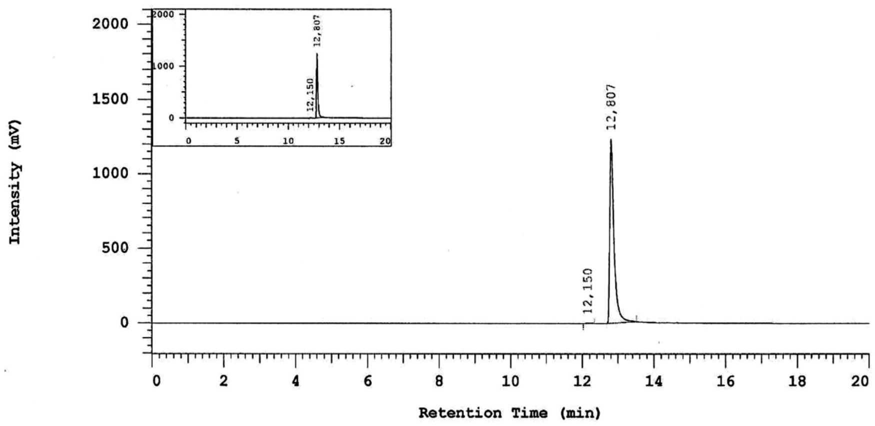

No.	RT	Area	Conc 1	BC
1	12,150	18685	0,182	BB
2	12,807	10271230	99,818	BB
		10289915	100,000	

(*S*)-**13l**

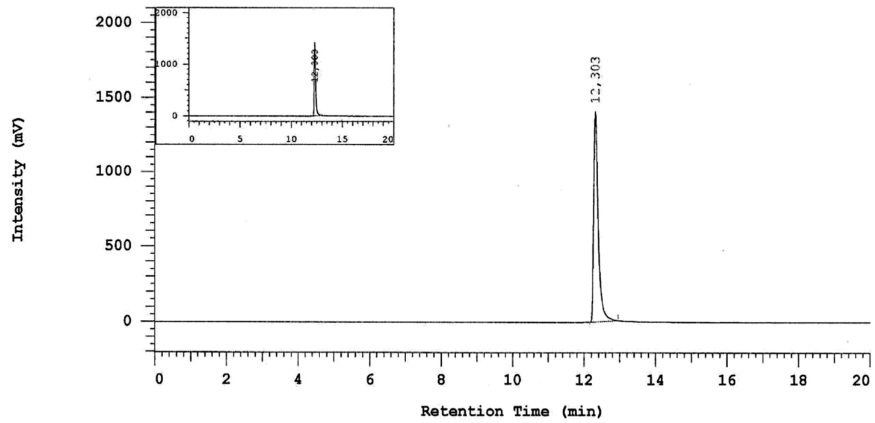

No.	RT	Area	Conc 1	BC
1	12,303	12078018	100,000	BB
		12078018	100,000	

(*S*)-**13m**

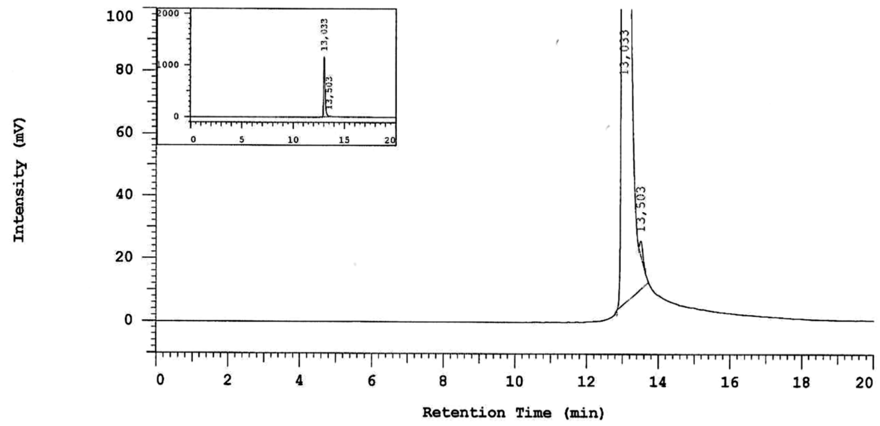

No.	RT	Area	Conc 1	BC
1	13,033	10002122	99,720	BV
2	13,503	28127	0,280	TBB
		10030249	100,000	

(*R*)-**13m**

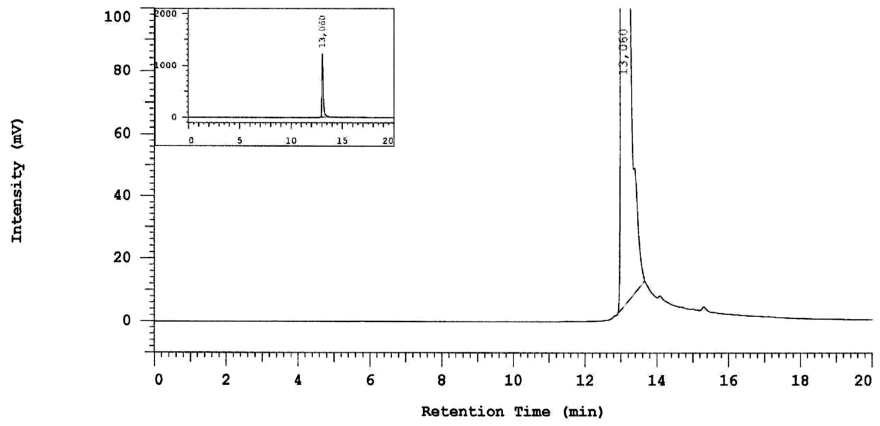

No.	RT	Area	Conc 1	BC
1	13,060	10160022	100,000	BB
		10160022	100,000	

(*S*)-**13n**

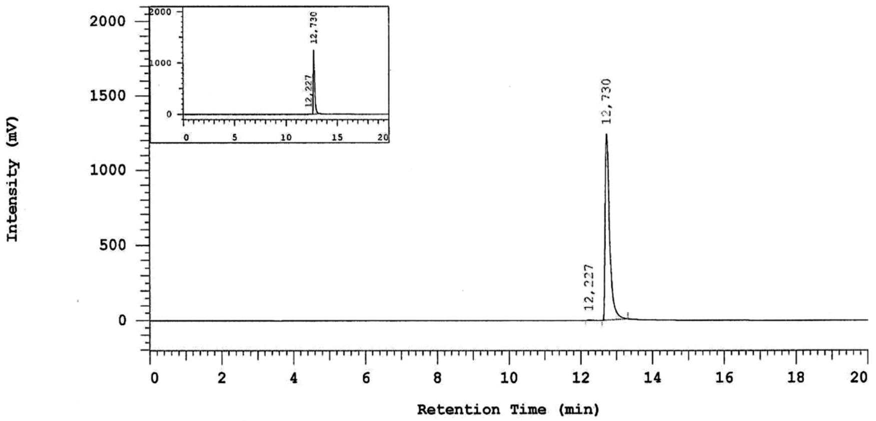

No.	RT	Area	Conc 1	BC
1	12,227	30487	0,283	BB
2	12,730	10735068	99,717	BB
		10765555	100,000	

(*R*)-**13n**

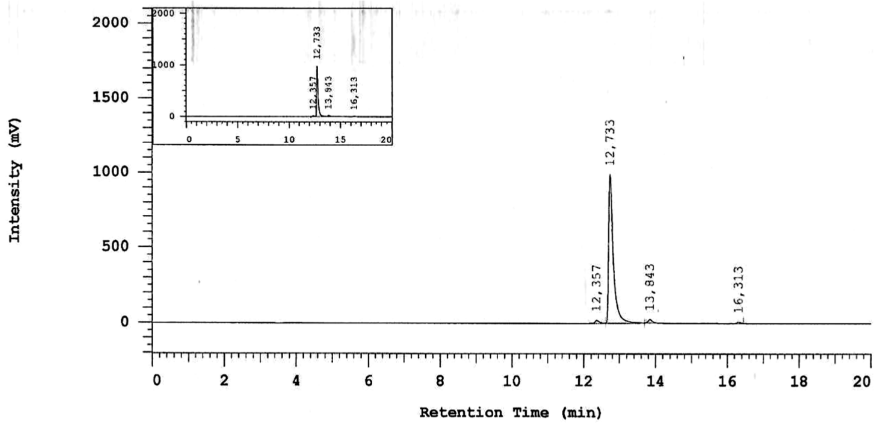

No.	RT	Area	Conc 1	BC
1	12,357	166818	1,776	BV
2	12,733	8994035	95,748	VV
3	13,843	169991	1,810	TBB
4	16,313	62600	0,666	BB
		9393444	100,000	

References

(1) Lee, C. J.; Liang, X.; Gopalaswamy, R.; Najeeb, J.; Ark, E. D.; Toone, E. J.; Zhou, P. Structural basis of the promiscuous inhibitor susceptibility of *Escherichia coli* LpxC. *ACS Chem. Biol.* **2014**, *9* (1), 237-246. DOI: 10.1021/cb400067g

(2) Cohen, F.; Aggen, J. B.; Andrews, L. D.; Assar, Z.; Boggs, J.; Choi, T.; Dozzo, P.; Easterday, A. N.; Haglund, C. M.; Hildebrandt, D. J.; Holt, M. C.; Joly, K.; Jubb, A.; Kamal, Z.; Kane, T. R.; Konradi, A. W.; Krause, K. M.; Linsell, M. S.; Machajewski, T. D.; Miroshnikova, O.; Moser, H. E.; Nieto, V.; Phan, T.; Plato, C.; Serio, A. W.; Seroogy, J.; Shakhmin, A.; Stein, A. J.; Sun, A. D.; Sviridov, S.; Wang, Z.; Wlasichuk, K.; Yang, W.; Zhou, X.; Zhu, H.; Cirz, R. T. Optimization of LpxC Inhibitors for Antibacterial Activity and Cardiovascular Safety. *ChemMedChem* **2019,** *14* (16), 1560-1572. DOI: 10.1002/cmdc.201900287

(3) Dreger, A.; Hoff, K.; Agoglitta, O.; Bülbül, E. F.; Melesina, J.; Sippl, W.; Holl, R. Synthesis, biological evaluation, and molecular docking studies of deoxygenated *C*-glycosides as LpxC inhibitors. *Bioorg. Chem.* **2021**, *117*, 105403. DOI: 10.1016/j.bioorg.2021.105403
